# Supplementary material for: Predictive Model for National Minimal CFR during Spontaneous Initial Outbreak of Emerging Infectious Disease: Lessons from COVID-19 Pandemic in 214 Nations and Regions
Source: Int J Environ Res Public Health. 2022 Dec 29;20(1):594. doi: 10.3390/ijerph20010594 (PMC9819427; doi:10.3390/ijerph20010594)
Supplement: Supplementary file 1 [file ijerph-20-00594-s001.zip › Figure S1 Curve lines of daily case fatality rates for COVID-19 in 40 nations.pdf]

**Figure: S1**

**Curve lines of daily case fatality rates for COVID-19 in 40 nations**

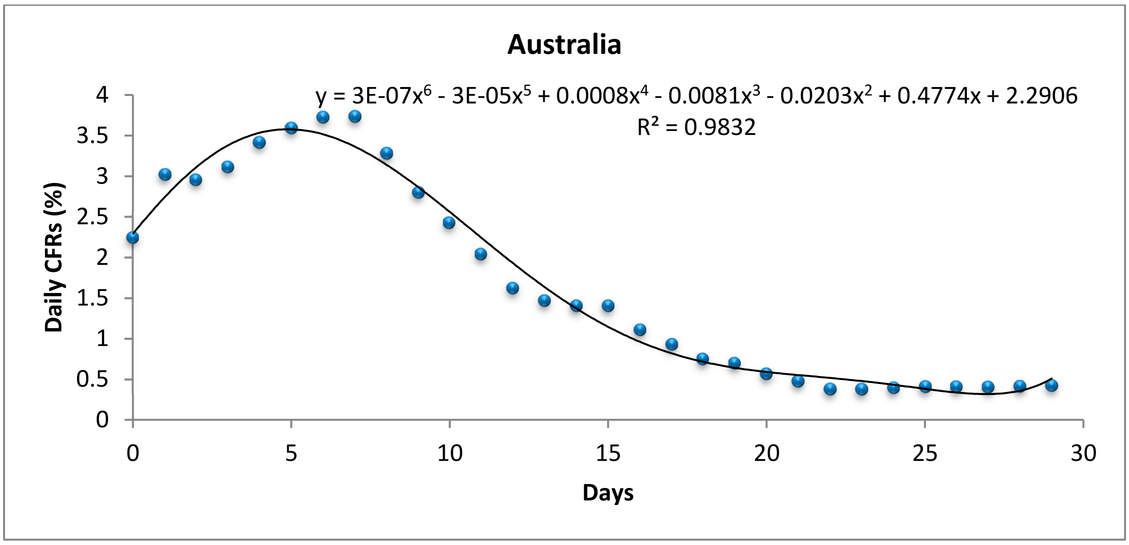


**(1) Daily CFRs since the first death of COVID-19 patient in Australia.**

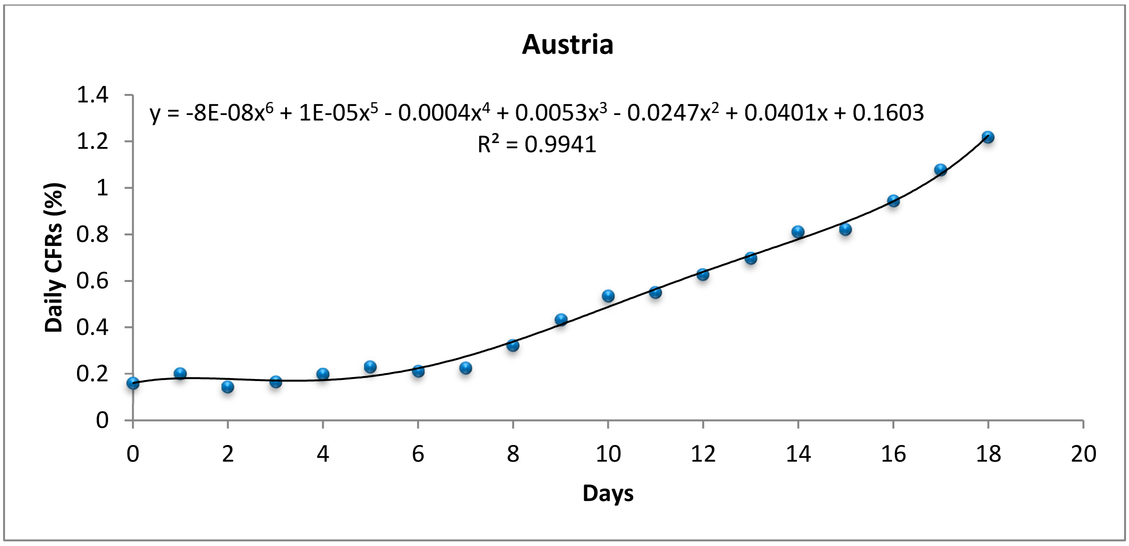


**(2) Daily CFRs since the first death of COVID-19 patient in Austria.**

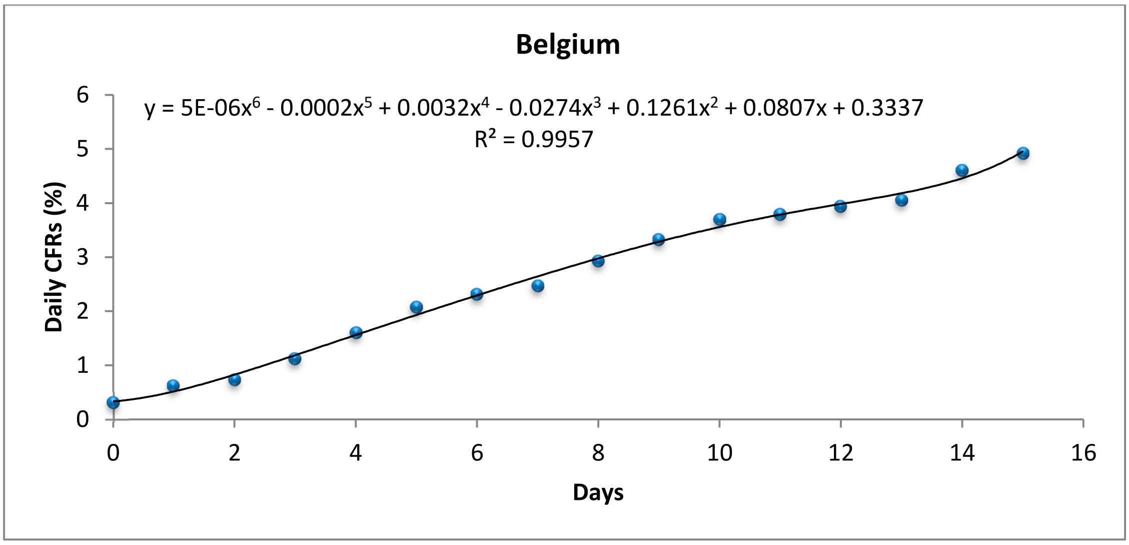


**(3) Daily CFRs since the first death of COVID-19 patient in Belgium.**

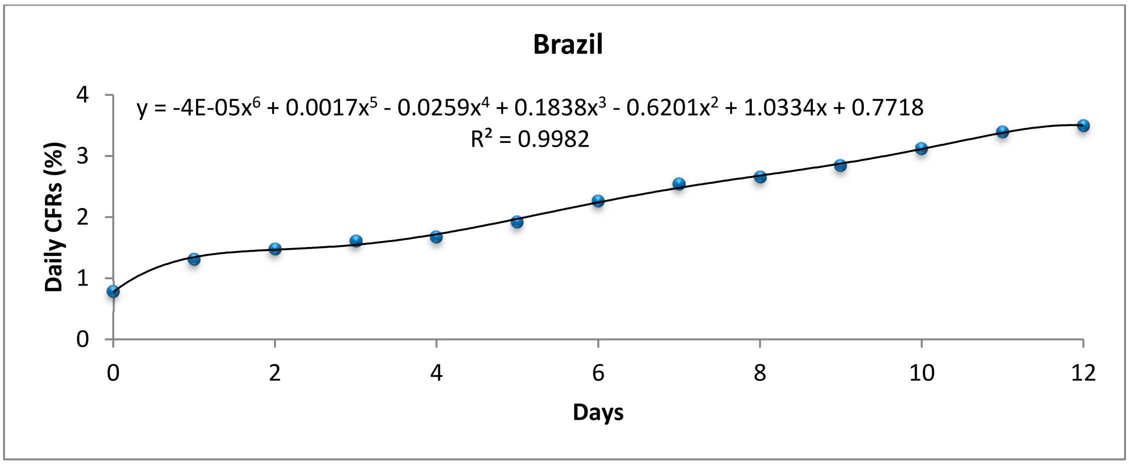


**(4) Daily CFRs since the first death of COVID-19 patient in Brazil.**

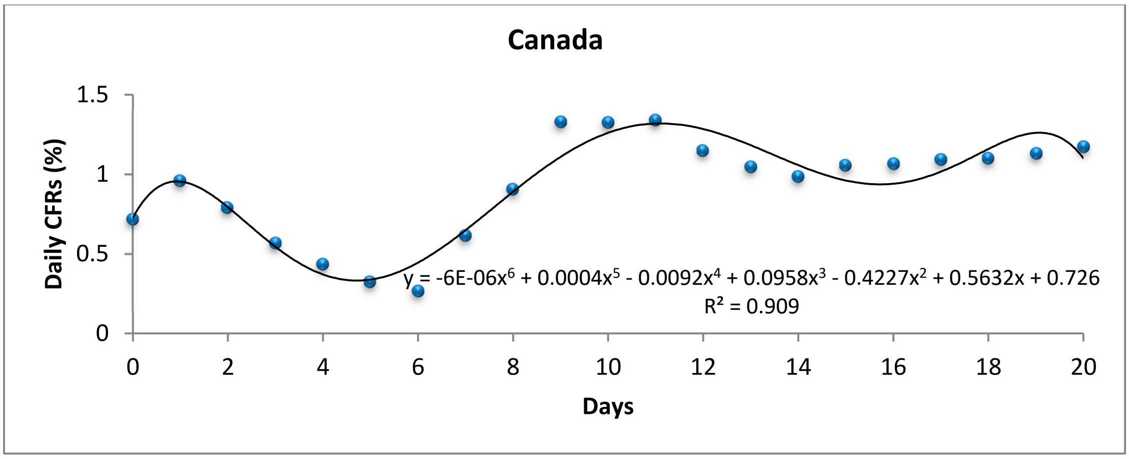


**(5) Daily CFRs since the first death of COVID-19 patient in Canada.**

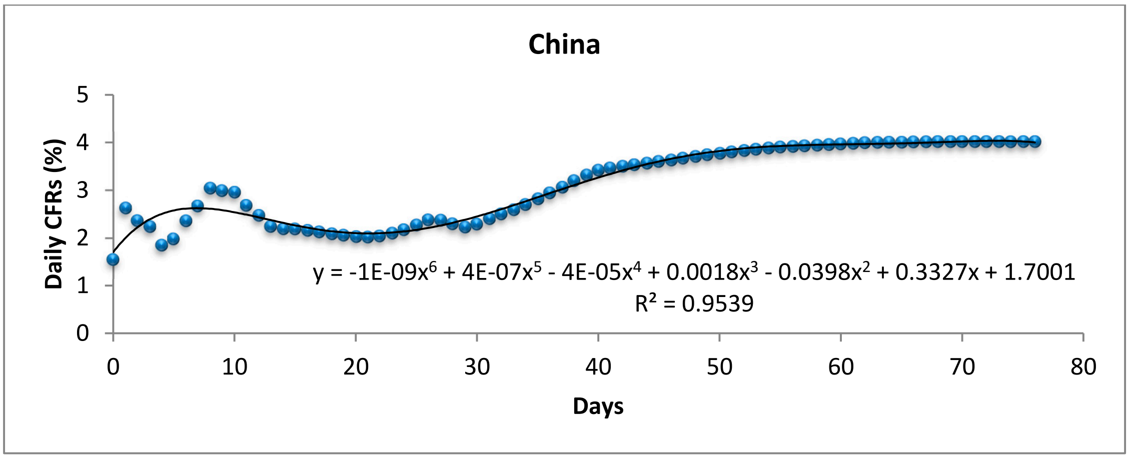


**(6) Daily CFRs since the first death of COVID-19 patient in China.**

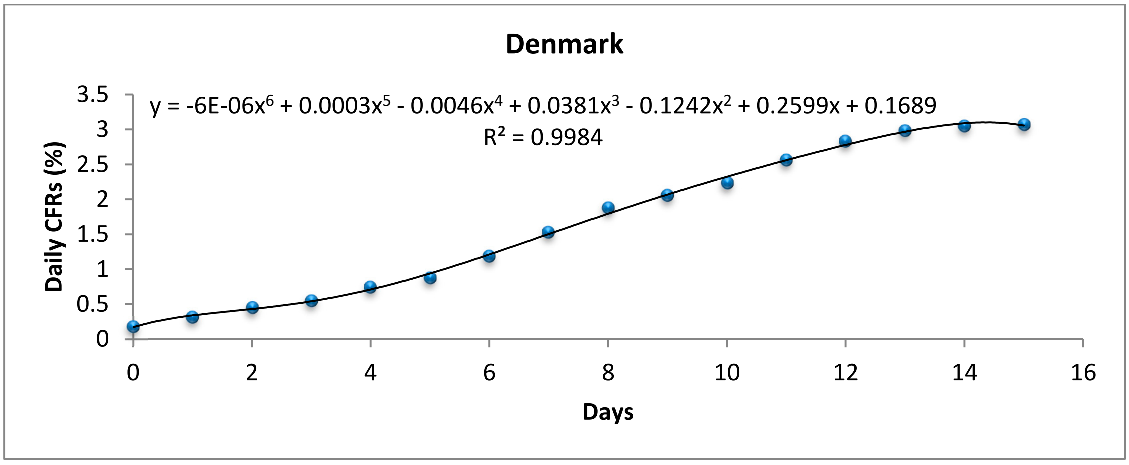


**(7) Daily CFRs since the first death of COVID-19 patient in Denmark.**

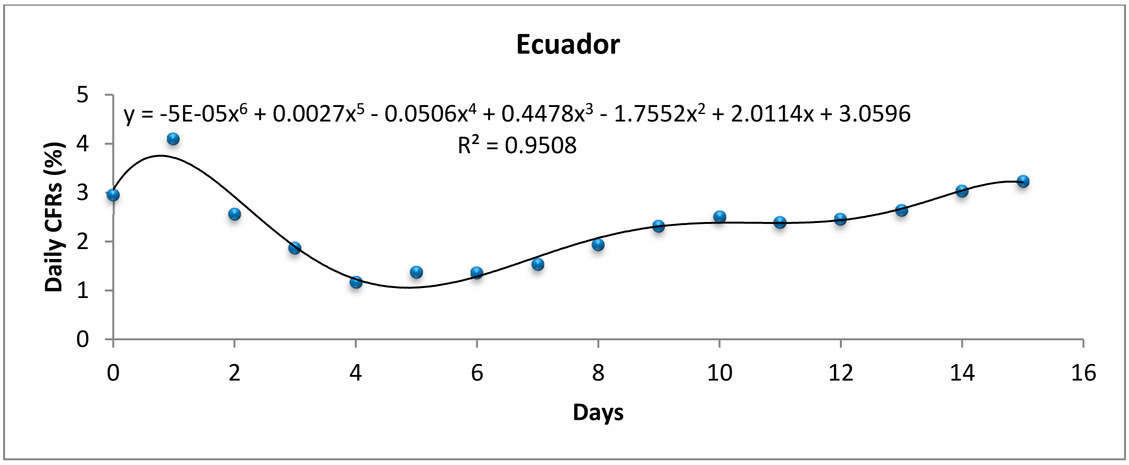


**(8) Daily CFRs since the first death of COVID-19 patient in Ecuador.**

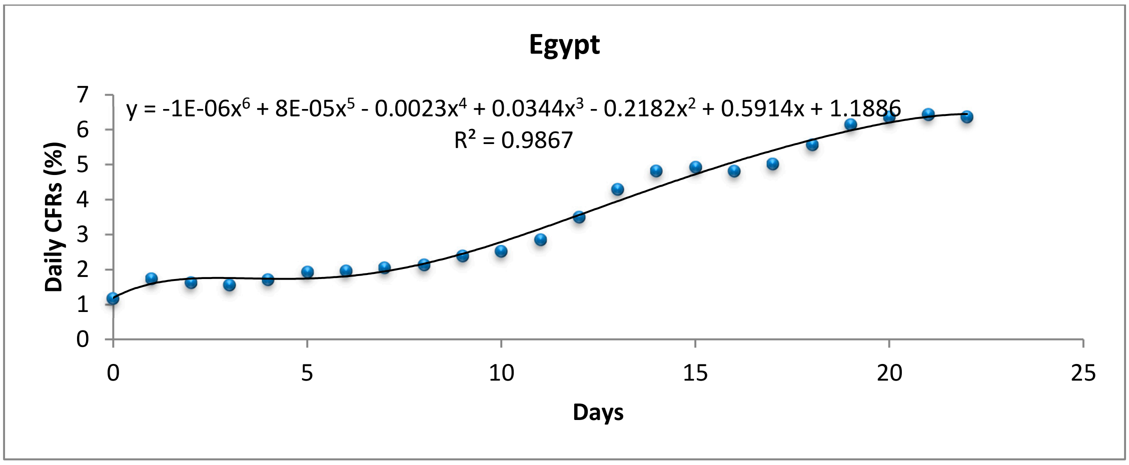


**(9) Daily CFRs since the first death of COVID-19 patient in Egypt.**

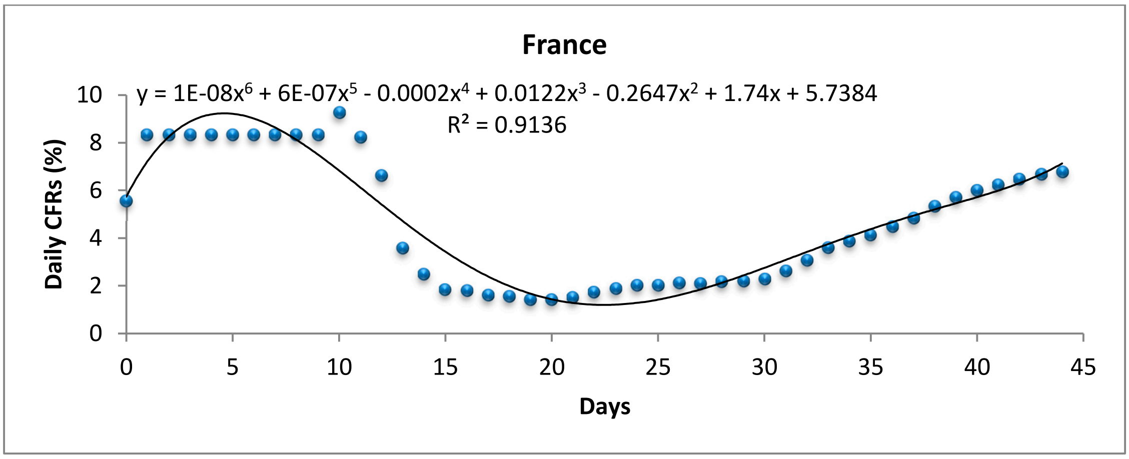


**(10) Daily CFRs since the first death of COVID-19 patient in France.**

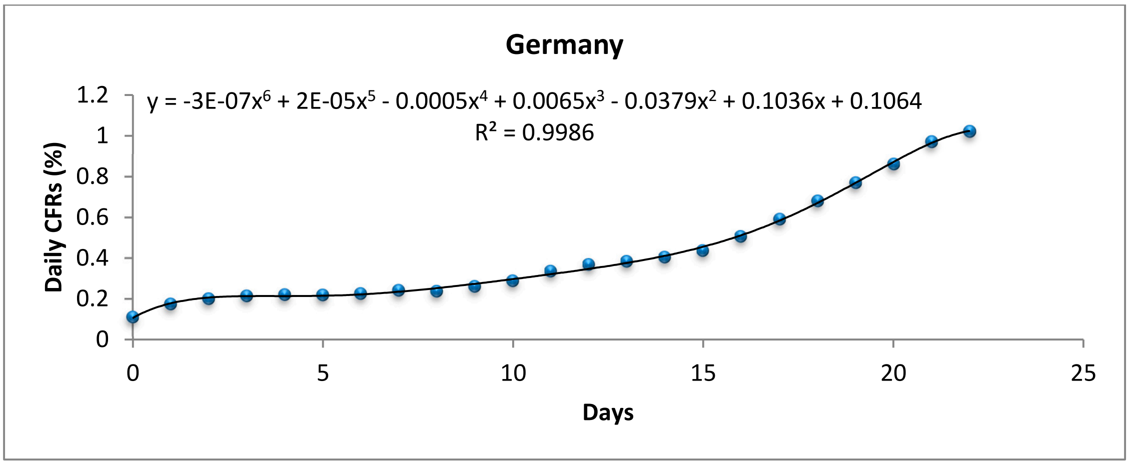


**(11) Daily CFRs since the first death of COVID-19 patient in Germany.**

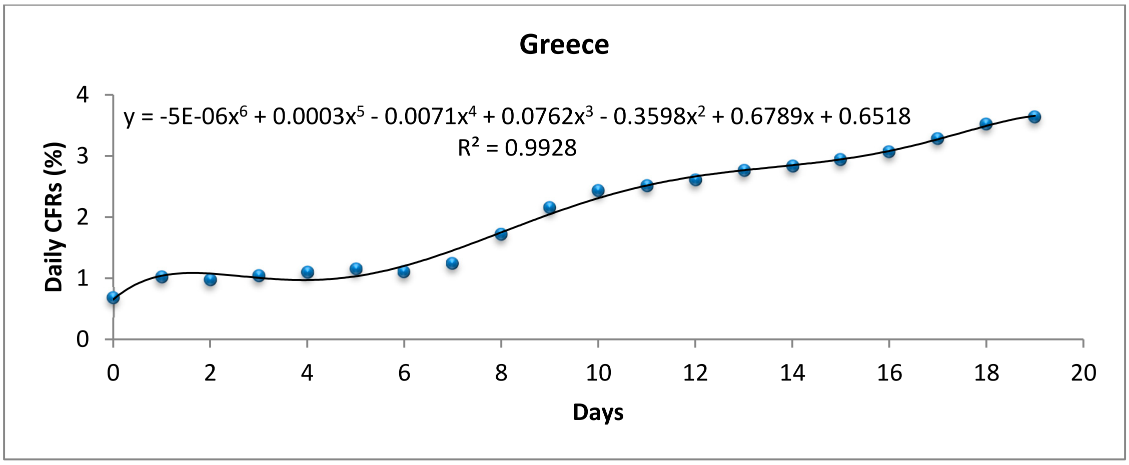


**(12) Daily CFRs since the first death of COVID-19 patient in Greece.**

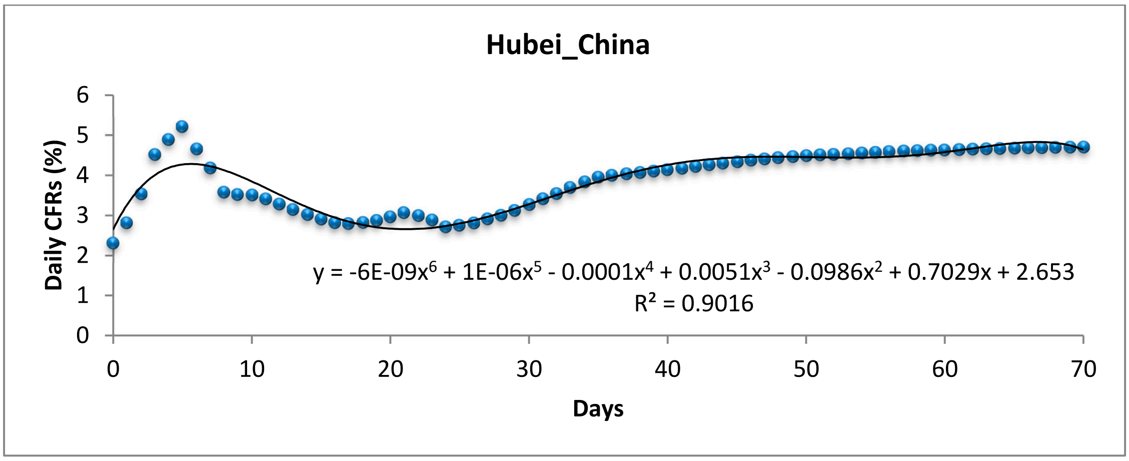


**(13) Daily CFRs since the first death of COVID-19 patient in Hubei_China.**

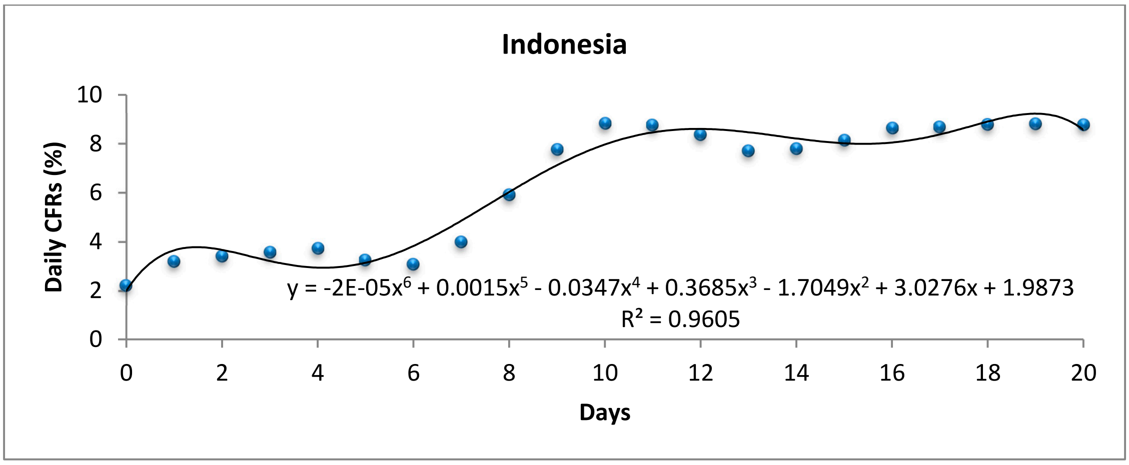


**(14) Daily CFRs since the first death of COVID-19 patient in Indonesia.**

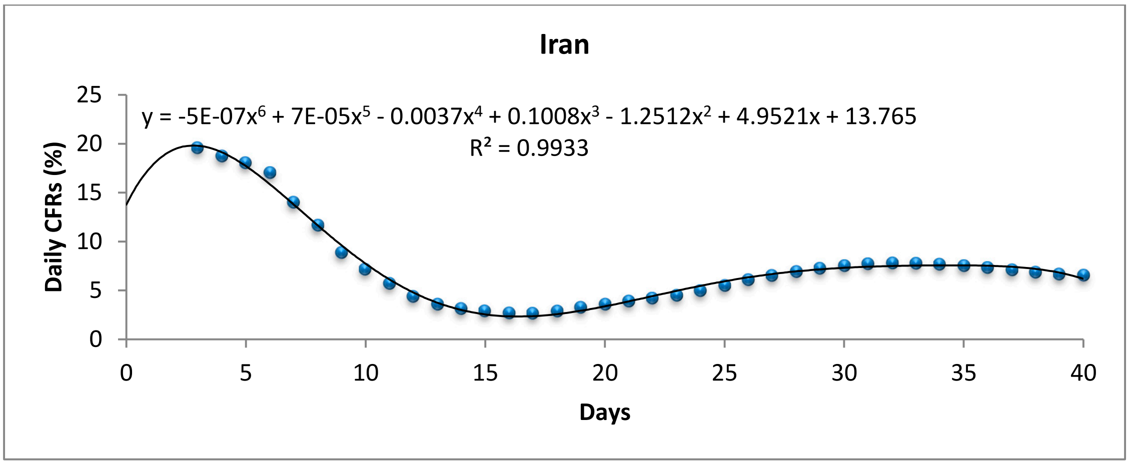


**(15) Daily CFRs since the first death of COVID-19 patient in Iran.**

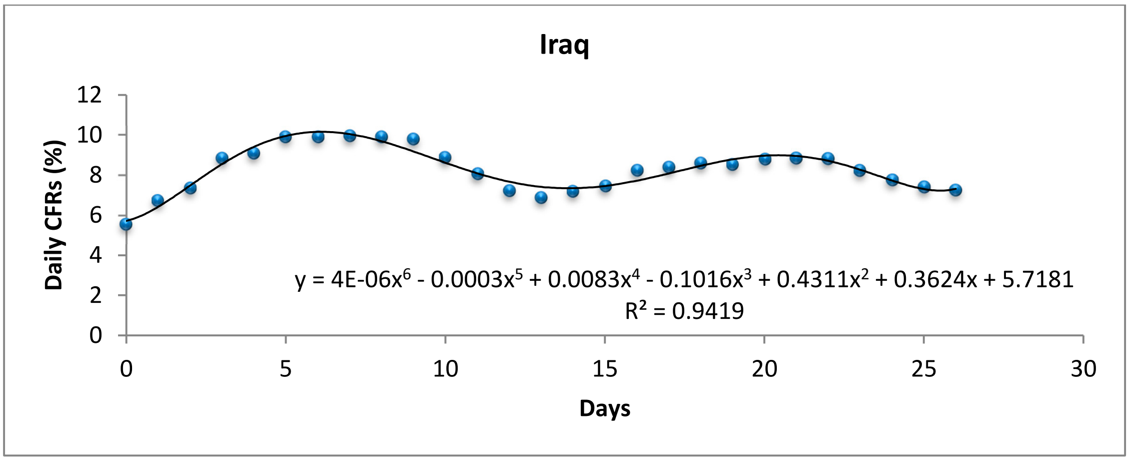


**(16) Daily CFRs since the first death of COVID-19 patient in Iraq.**

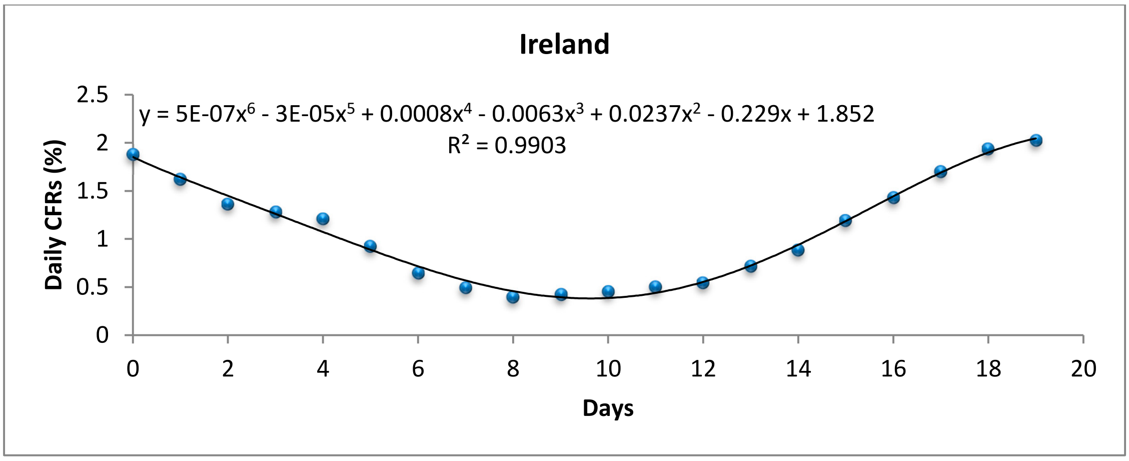


**(17) Daily CFRs since the first death of COVID-19 patient in Ireland.**

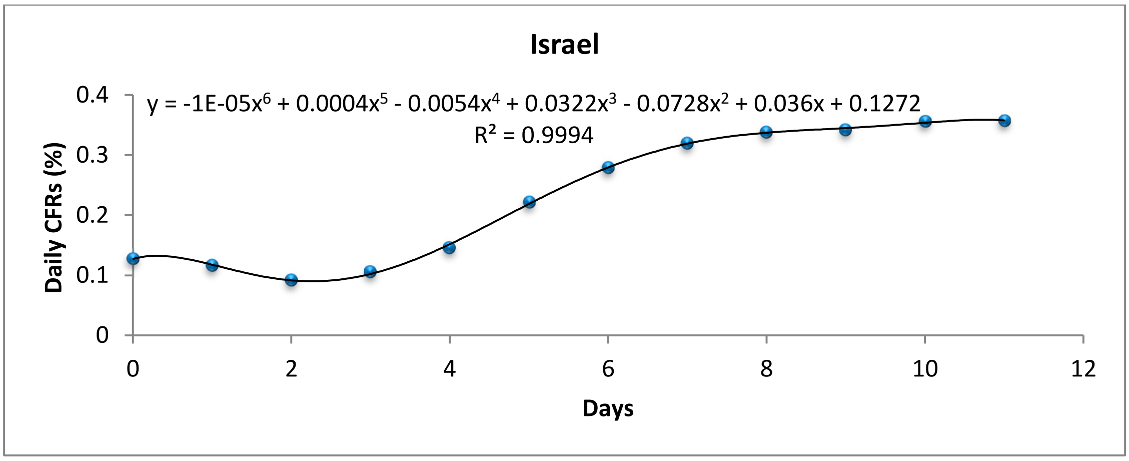


**(18) Daily CFRs since the first death of COVID-19 patient in Israel.**

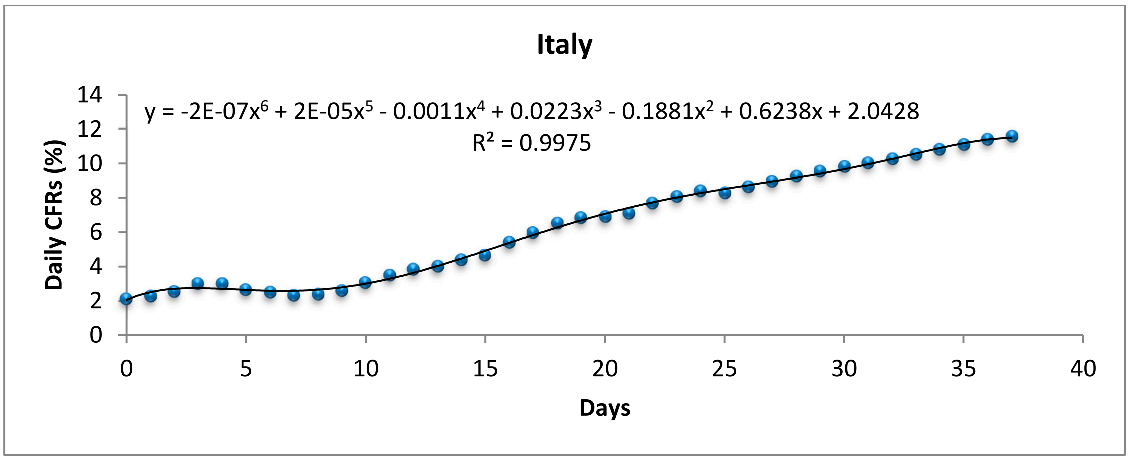


**(19) Daily CFRs since the first death of COVID-19 patient in Italy.**

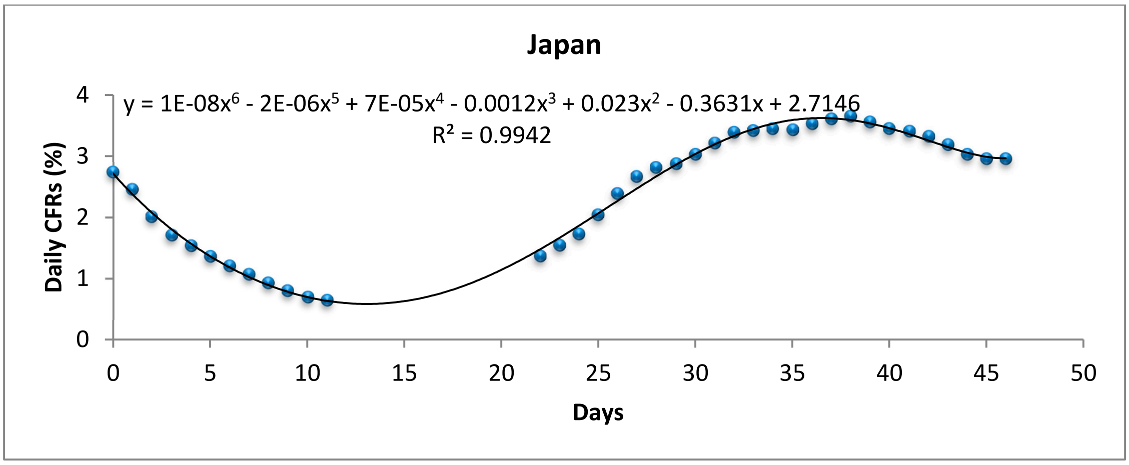


**(20) Daily CFRs since the first death of COVID-19 patient in Japan.**

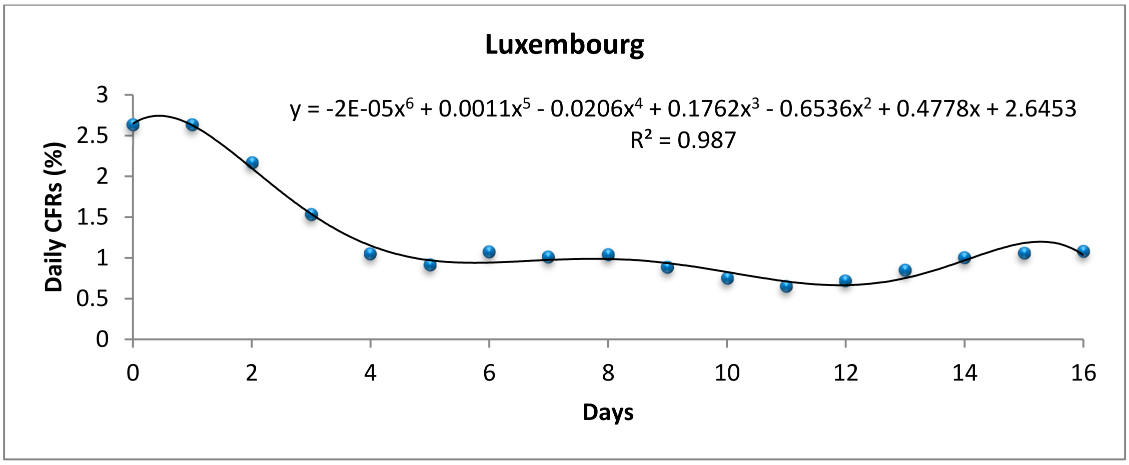


**(21) Daily CFRs since the first death of COVID-19 patient in Luxembourg.**

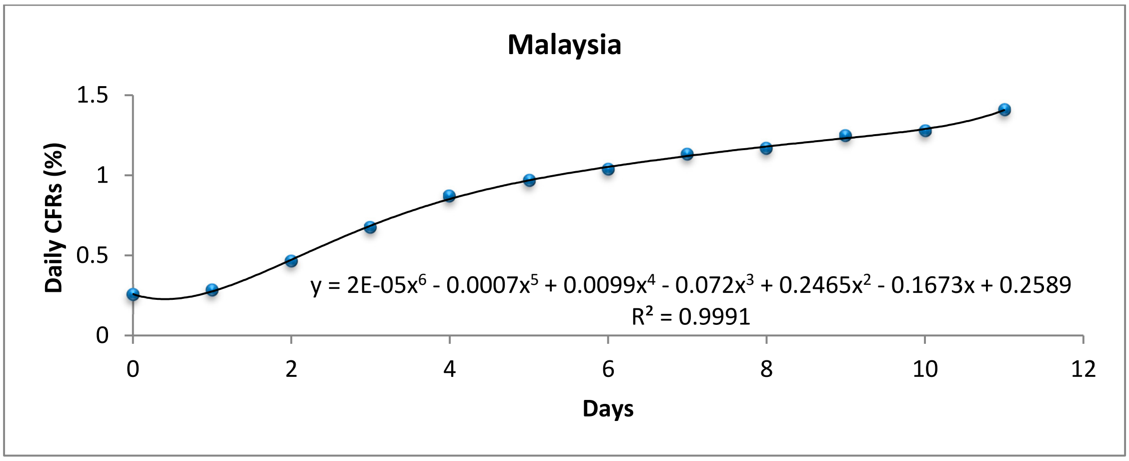


**(22) Daily CFRs since the first death of COVID-19 patient in Malaysia.**

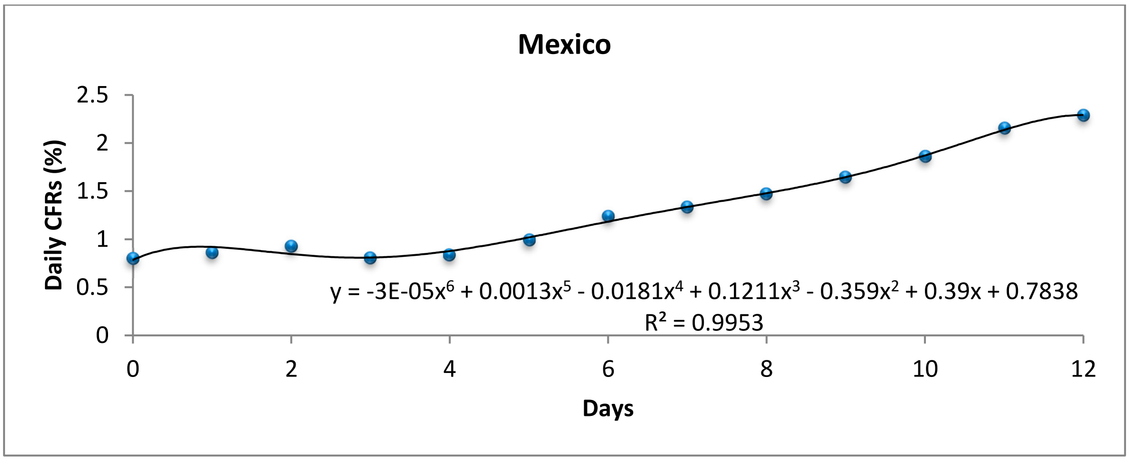


**(23) Daily CFRs since the first death of COVID-19 patient in Mexico.**

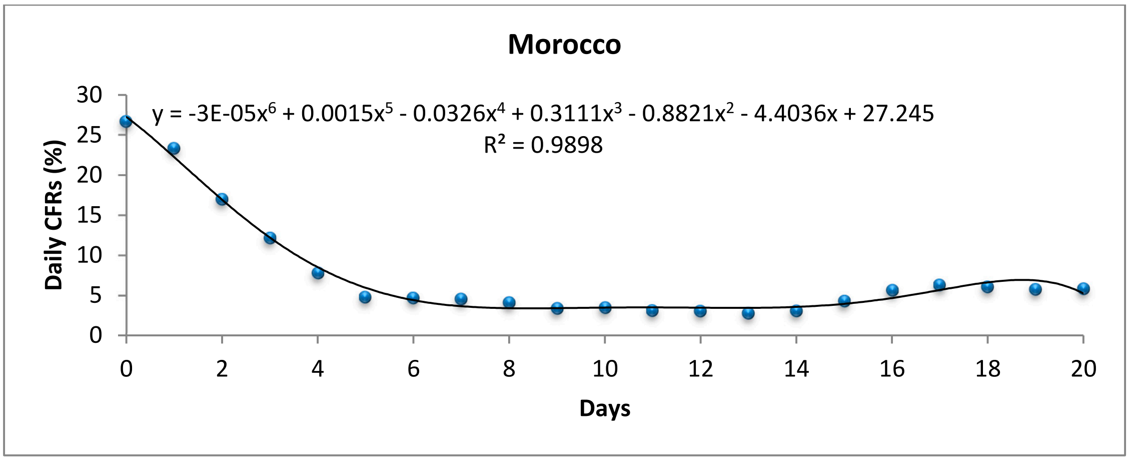


**(24) Daily CFRs since the first death of COVID-19 patient in Morocco.**

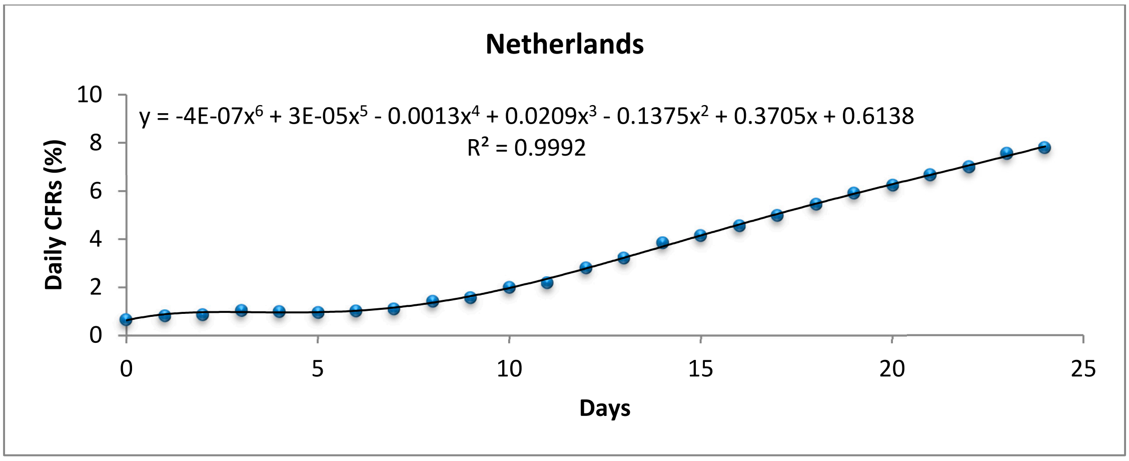


**(25) Daily CFRs since the first death of COVID-19 patient in Netherlands.**

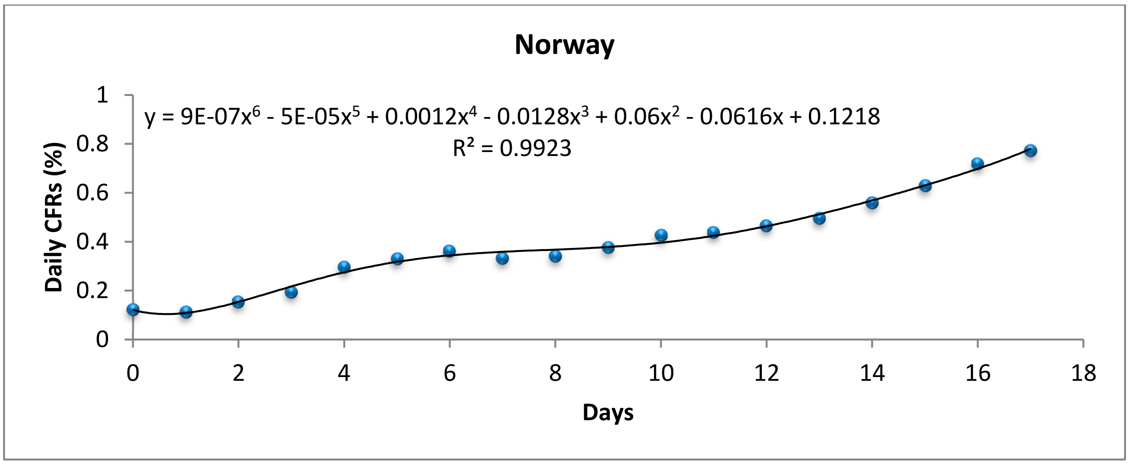


**(26) Daily CFRs since the first death of COVID-19 patient in Norway.**

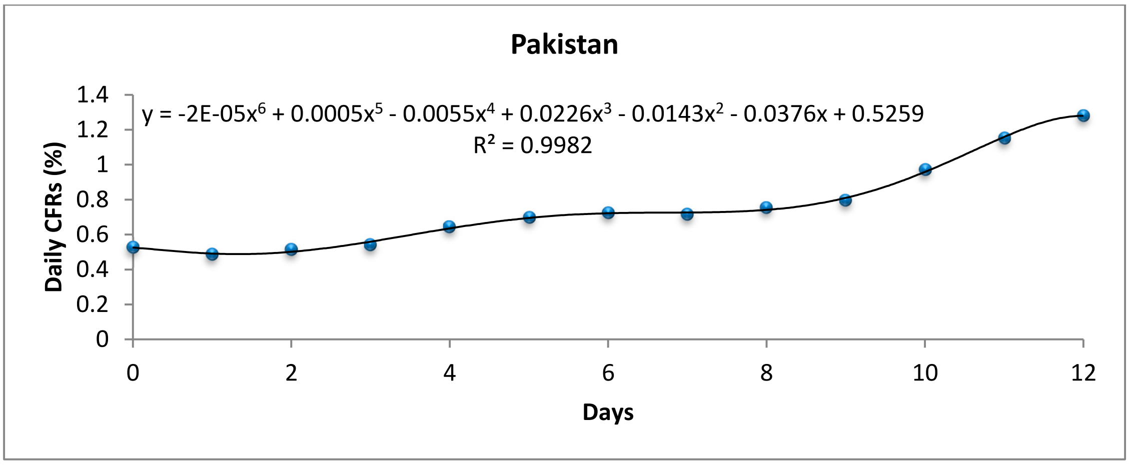


**(27) Daily CFRs since the first death of COVID-19 patient in Pakistan.**

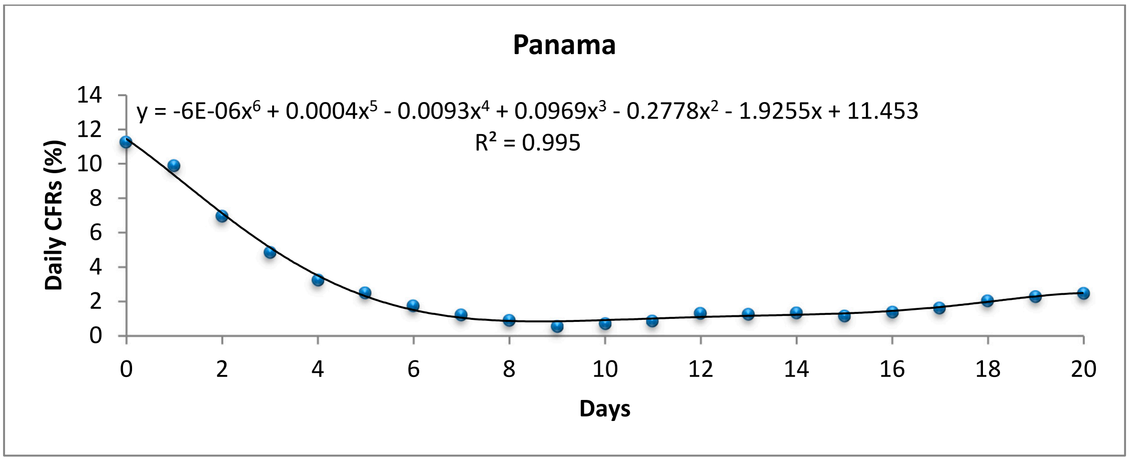


**(28) Daily CFRs since the first death of COVID-19 patient in Panama.**

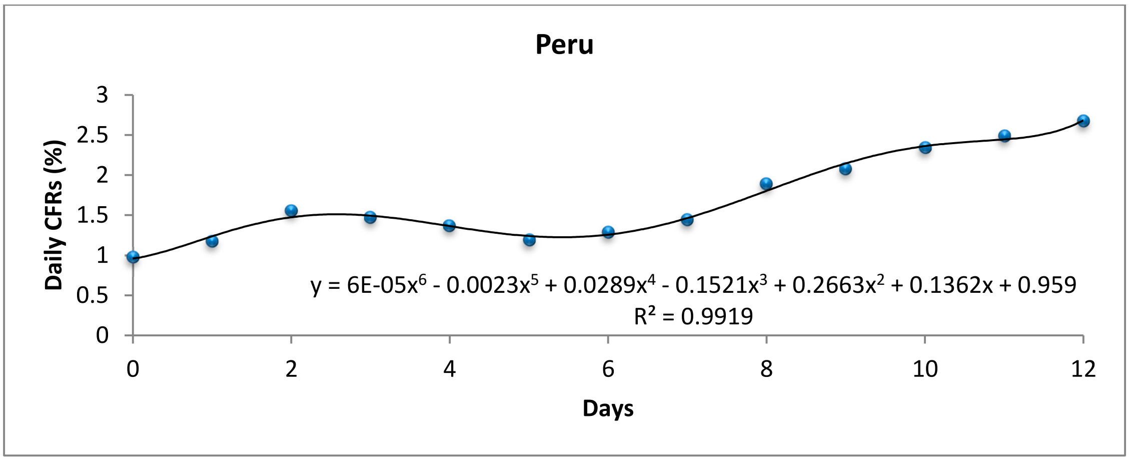


**(29) Daily CFRs since the first death of COVID-19 patient in Peru.**

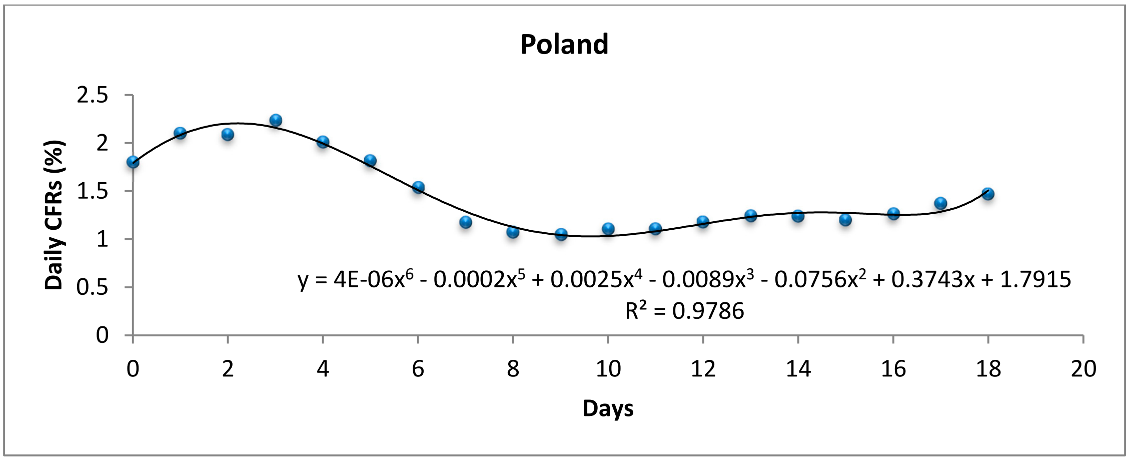


**(30) Daily CFRs since the first death of COVID-19 patient in Poland.**

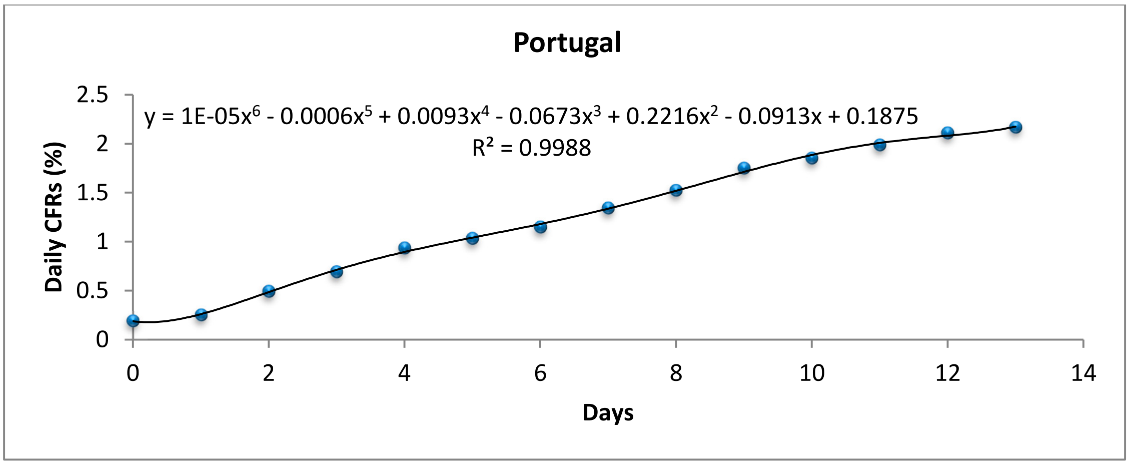


**(31) Daily CFRs since the first death of COVID-19 patient in Portugal.**

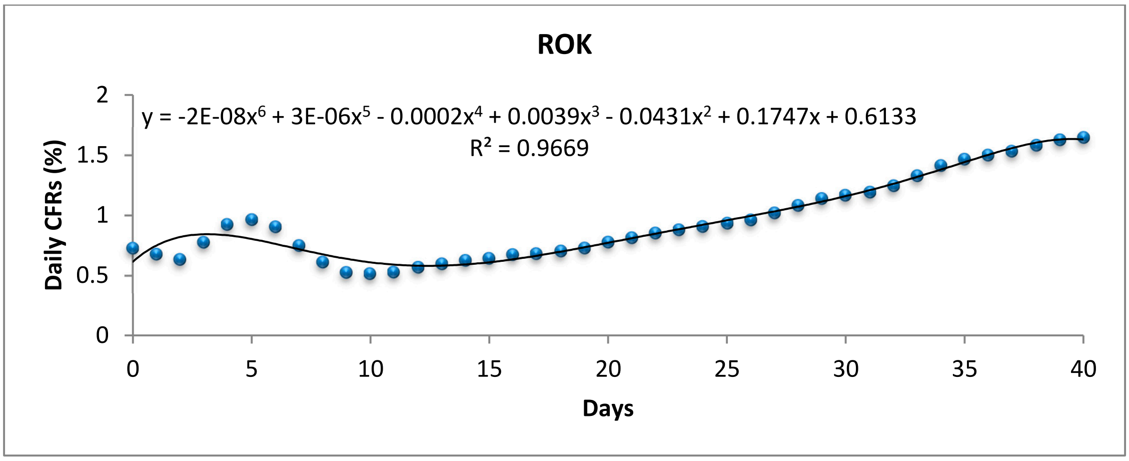


**(32) Daily CFRs since the first death of COVID-19 patient in ROK.**

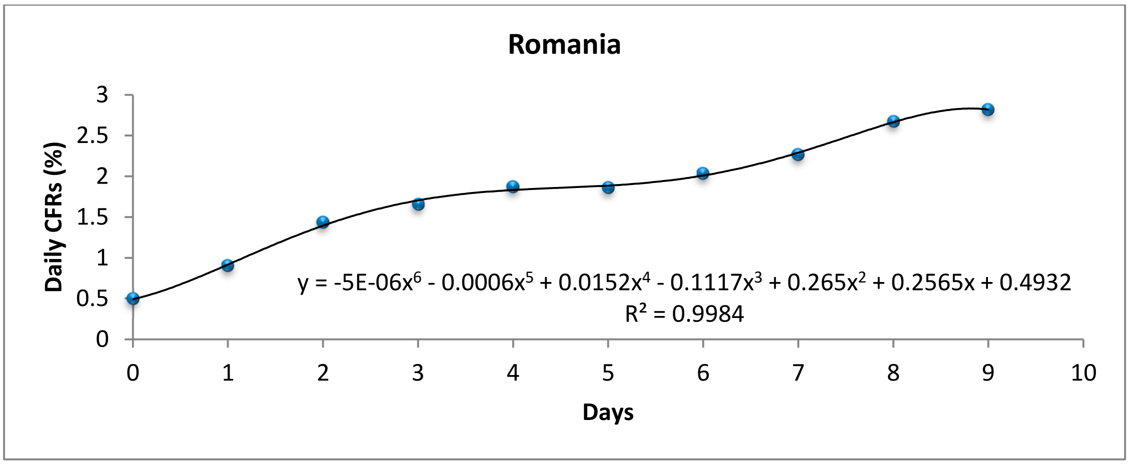


**(33) Daily CFRs since the first death of COVID-19 patient in Romania.**

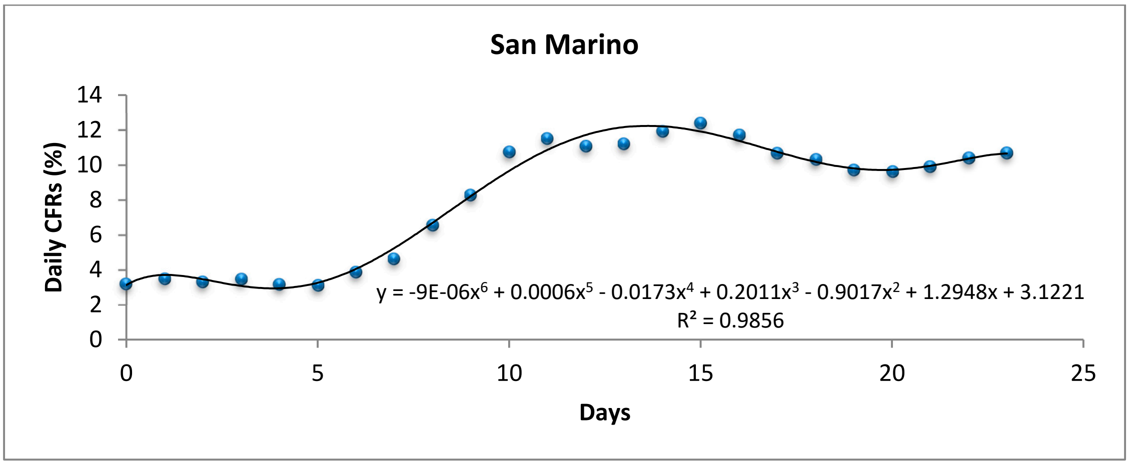


**(34) Daily CFRs since the first death of COVID-19 patient in San Marino.**

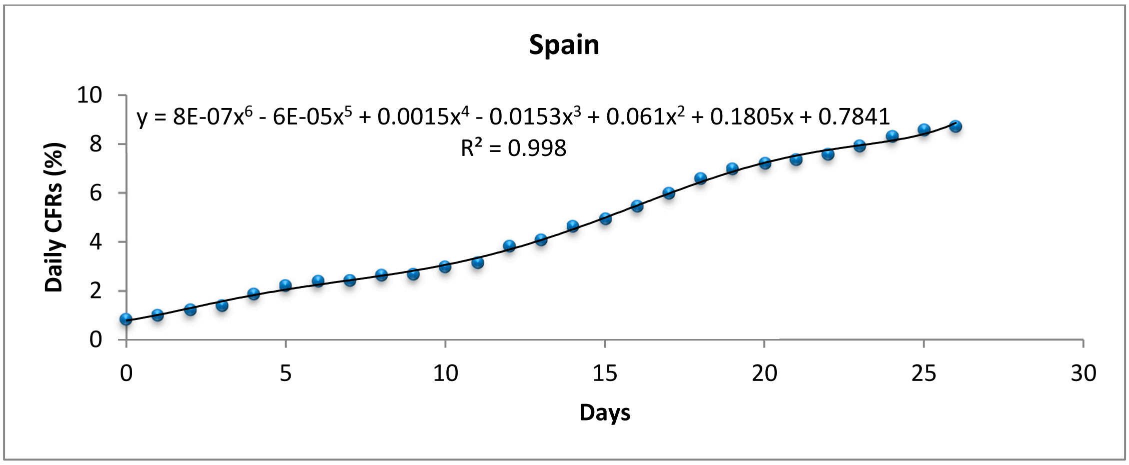


**(35) Daily CFRs since the first death of COVID-19 patient in Spain.**

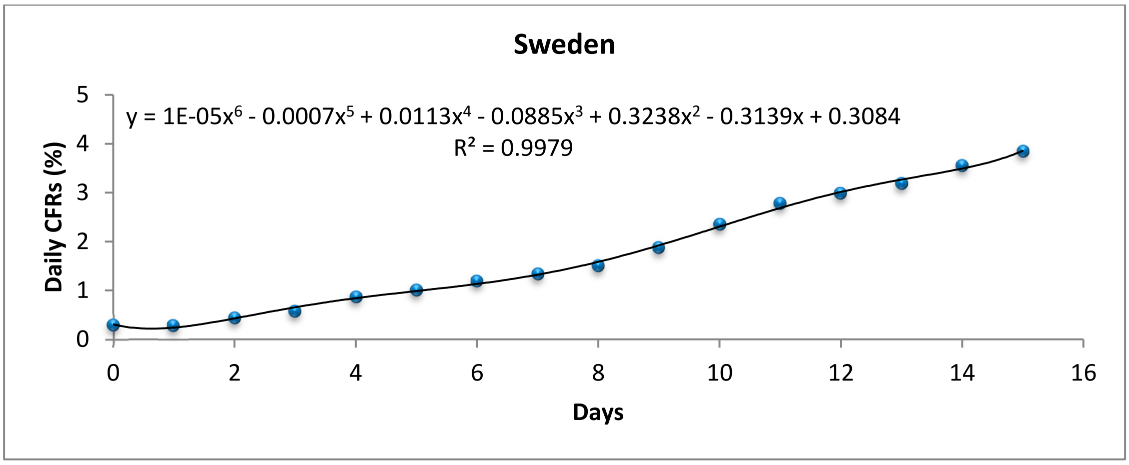


**(36) Daily CFRs since the first death of COVID-19 patient in Sweden.**

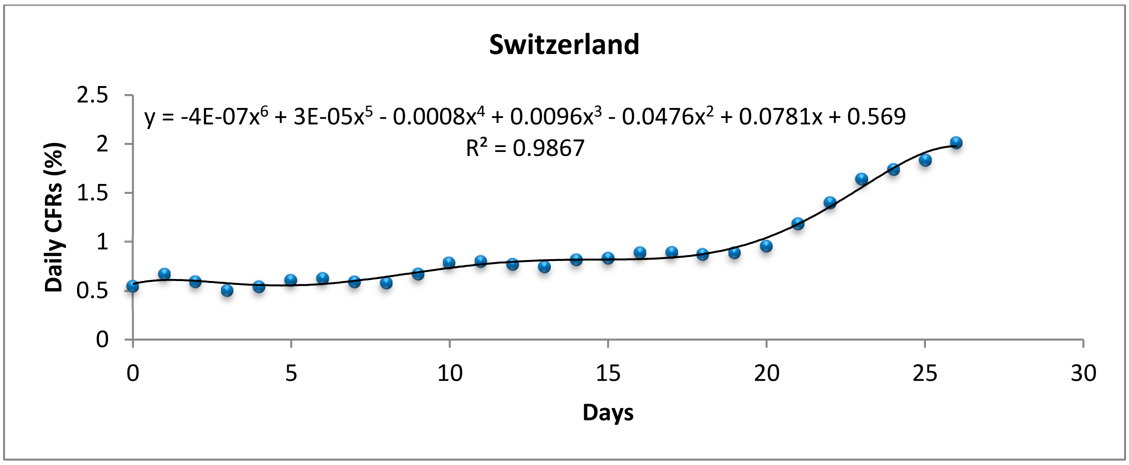


**(37) Daily CFRs since the first death of COVID-19 patient in Switzerland.**

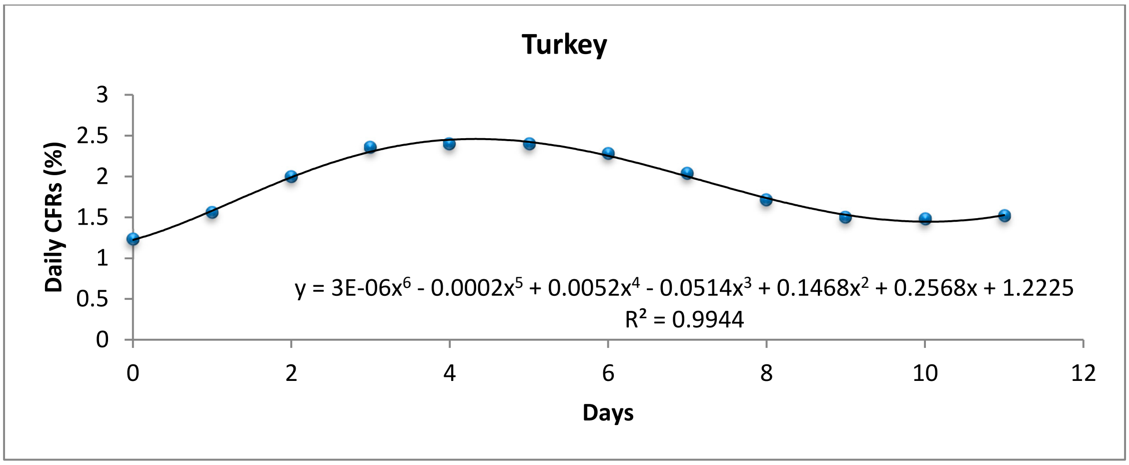


**(38) Daily CFRs since the first death of COVID-19 patient in Turkey.**

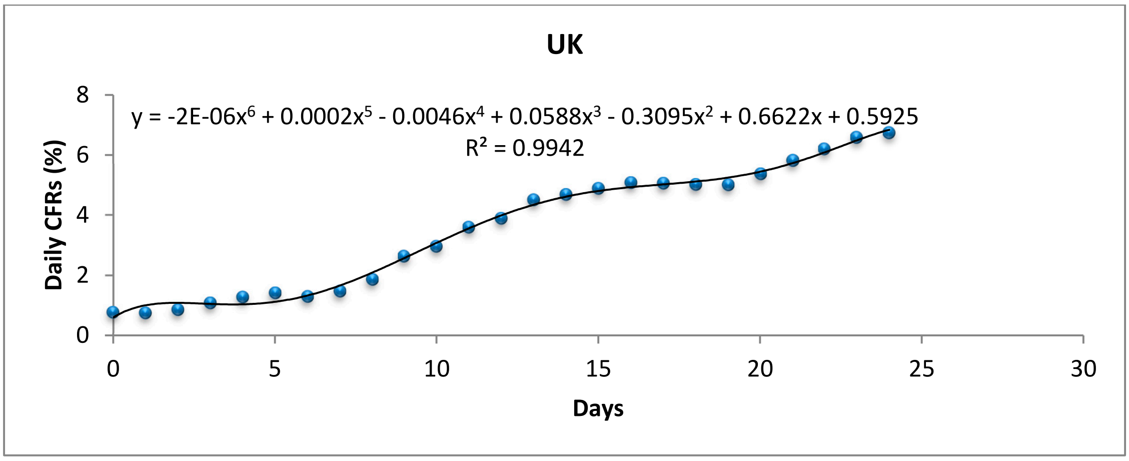


**(39) Daily CFRs since the first death of COVID-19 patient in UK.**

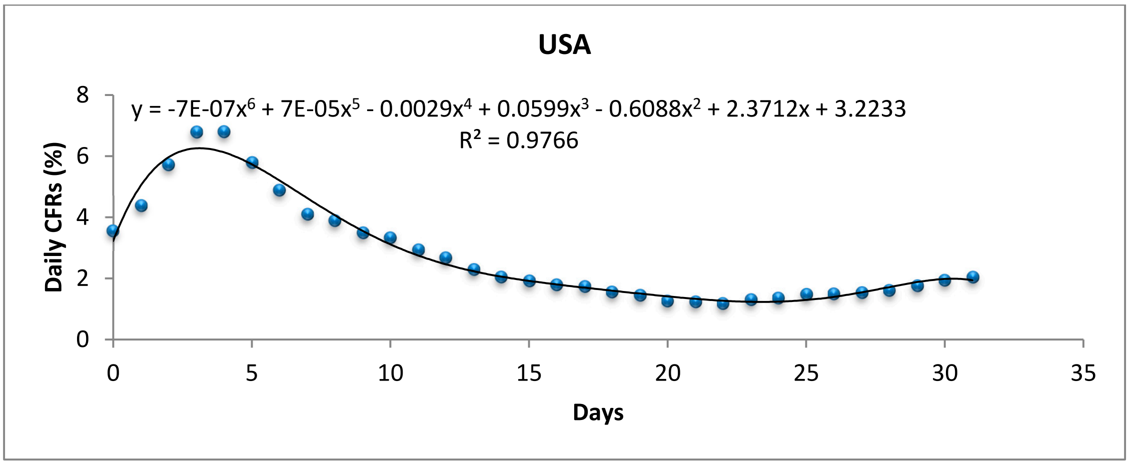


**(40) Daily CFRs since the first death of COVID-19 patient in USA.**
