# Supplementary material for: Predictive Model for National Minimal CFR during Spontaneous Initial Outbreak of Emerging Infectious Disease: Lessons from COVID-19 Pandemic in 214 Nations and Regions
Source: Int J Environ Res Public Health. 2022 Dec 29;20(1):594. doi: 10.3390/ijerph20010594 (PMC9819427; doi:10.3390/ijerph20010594)
Supplement: Supplementary file 1 [file ijerph-20-00594-s001.zip › Figure S2 3DMA CHRs in 92nations.pdf]

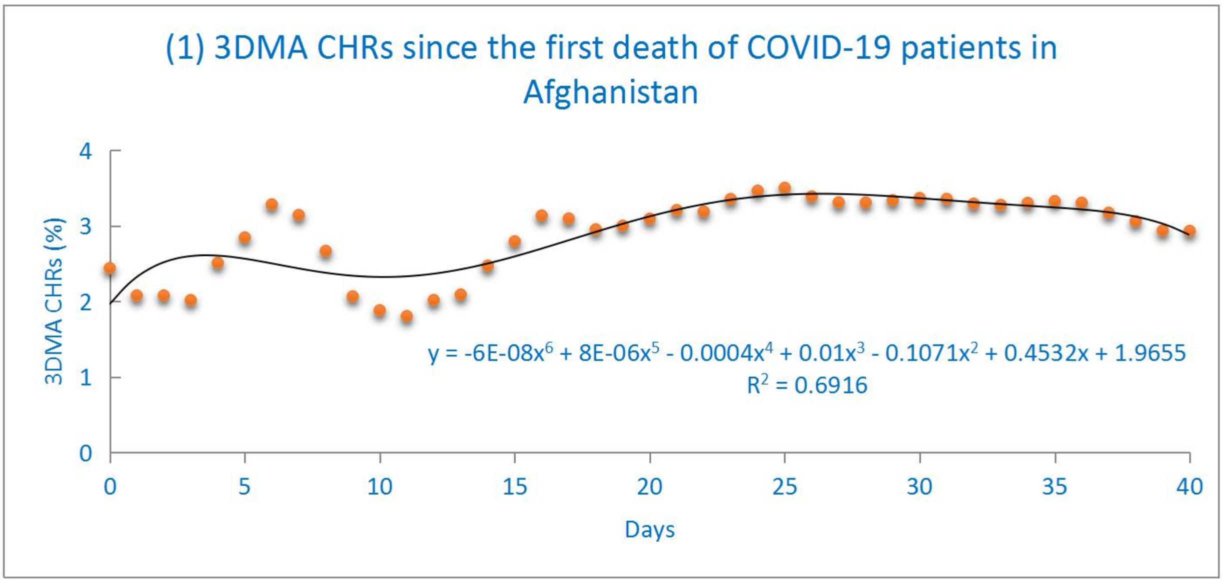
(1) 3DMA CHRs since the first death of COVID-19 patients in Afghanistan
3DMA CHRs (%)
Days
0
1
2
3
4
5
10
15
20
25
30
35
40
y = -6E-08x6 + 8E-06x5 - 0.0004x4 + 0.01x3 - 0.1071x2 + 0.4532x + 1.9655
R2 = 0.6916

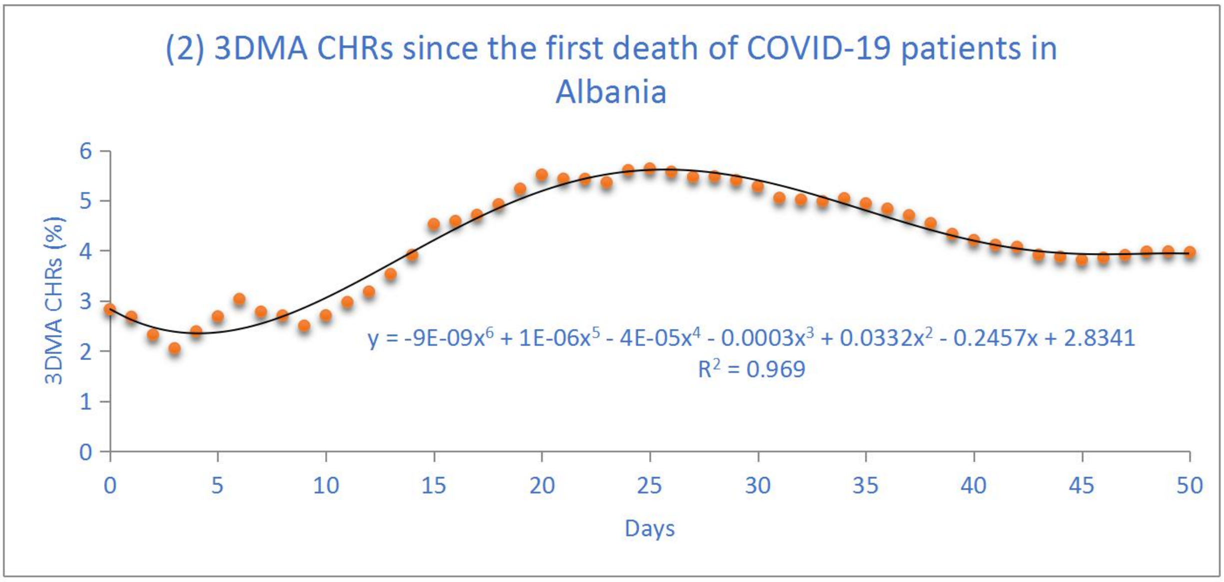
(2) 3DMA CHRs since the first death of COVID-19 patients in Albania
3DMA CHRs (%)
Days
0
1
2
3
4
5
6
10
15
20
25
30
35
40
45
50
y = -9E-09x6 + 1E-06x5 - 4E-05x4 - 0.0003x3 + 0.0332x2 - 0.2457x + 2.8341
R2 = 0.969

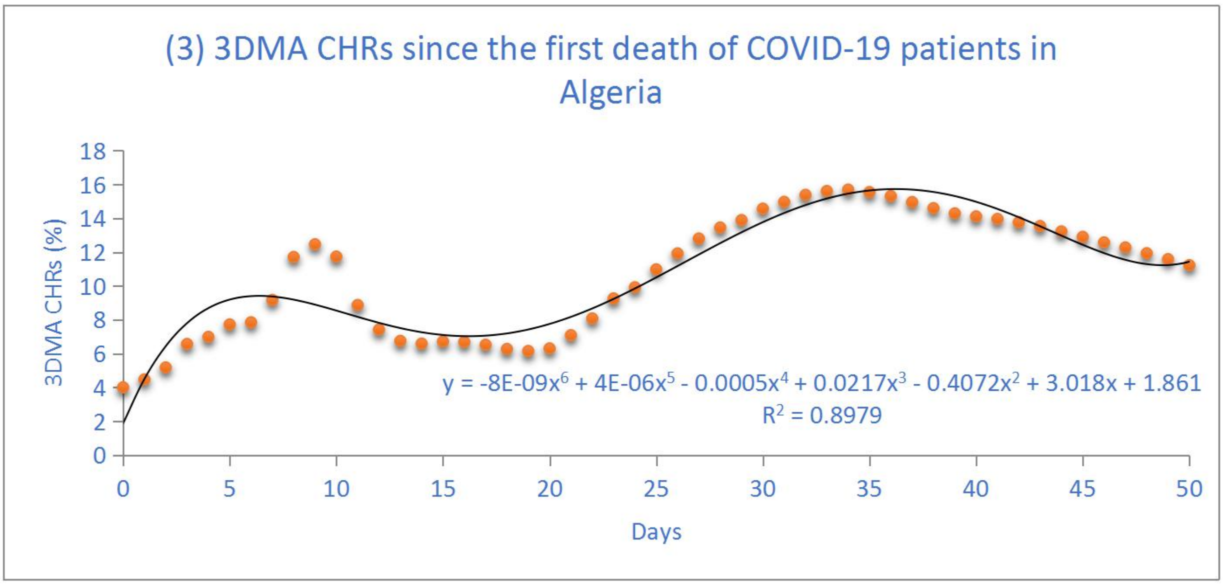
(3) 3DMA CHRs since the first death of COVID-19 patients in Algeria
3DMA CHRs (%)
Days
0
2
4
6
8
10
12
14
16
18
5
15
20
25
30
35
40
45
50
y = -8E-09x6 + 4E-06x5 - 0.0005x4 + 0.0217x3 - 0.4072x2 + 3.018x + 1.861
R2 = 0.8979

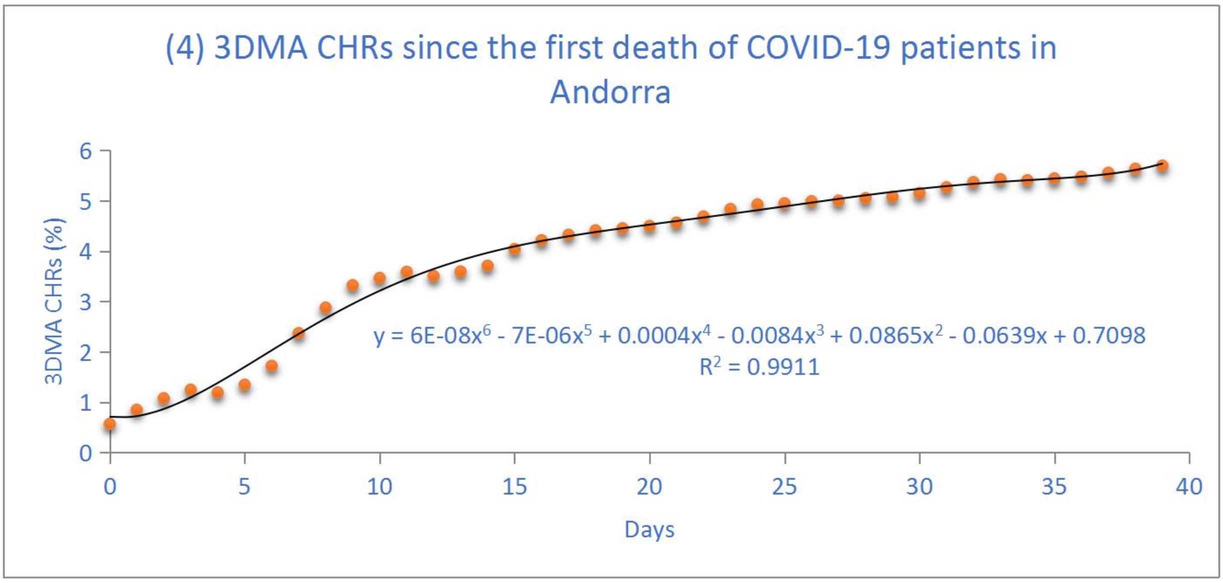
(4) 3DMA CHRs since the first death of COVID-19 patients in Andorra
3DMA CHRs (%)
Days
y = 6E-08x6 - 7E-06x5 + 0.0004x4 - 0.0084x3 + 0.0865x2 - 0.0639x + 0.7098
R2 = 0.9911
0
1
2
3
4
5
6
0
5
10
15
20
25
30
35
40

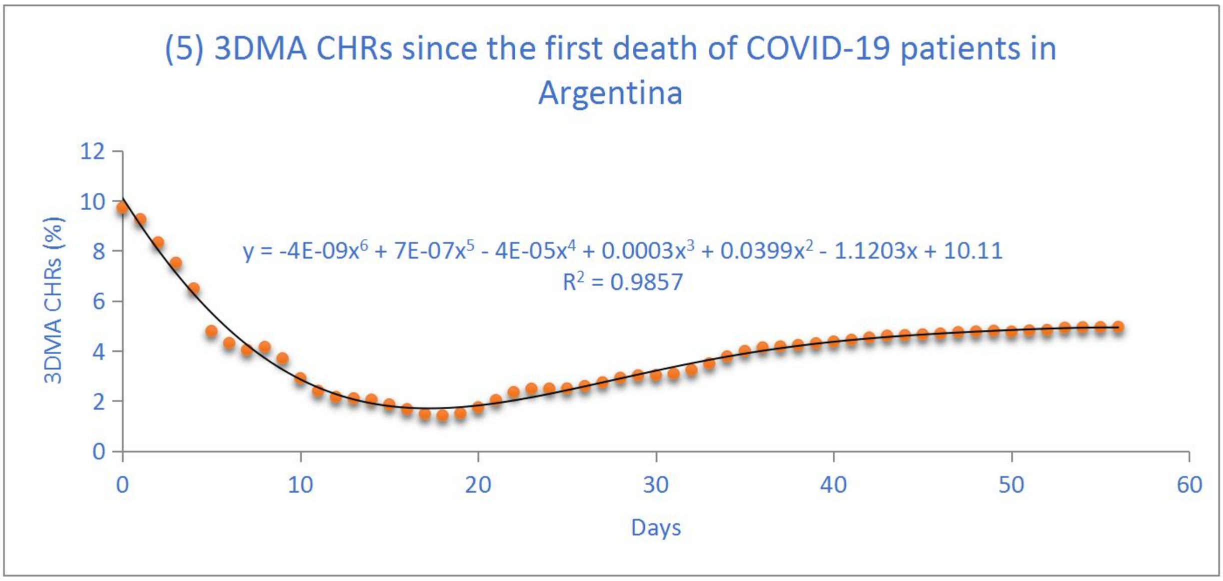
(5) 3DMA CHRs since the first death of COVID-19 patients in Argentina
3DMA CHRs (%)
Days
y = -4E-09x6 + 7E-07x5 - 4E-05x4 + 0.0003x3 + 0.0399x2 - 1.1203x + 10.11
R2 = 0.9857
0
2
4
6
8
10
12
0
10
20
30
40
50
60

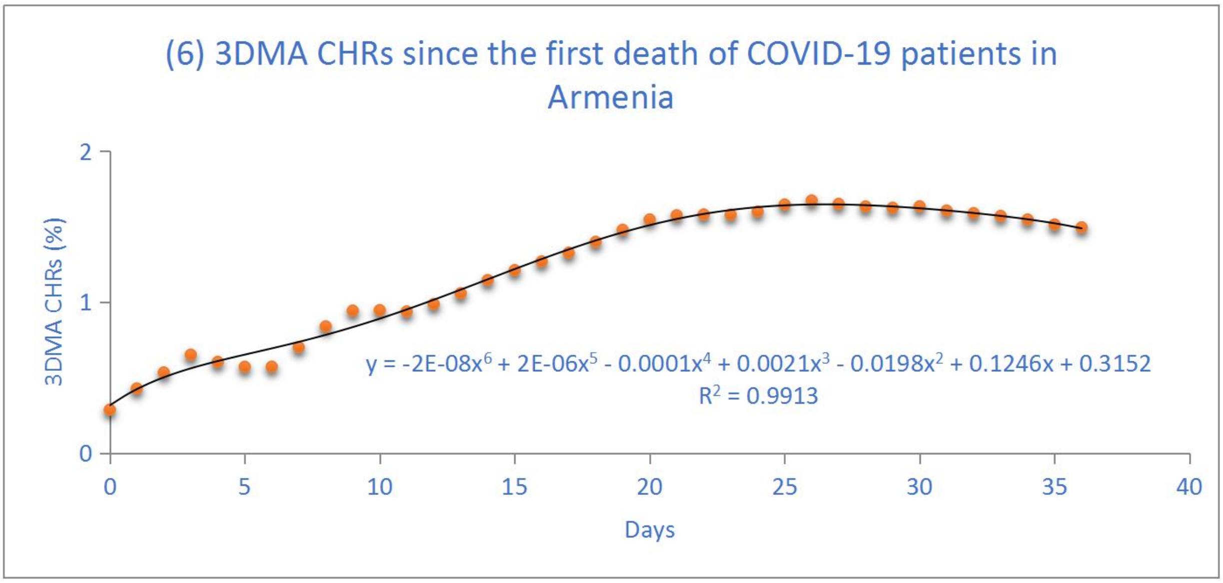
(6) 3DMA CHRs since the first death of COVID-19 patients in Armenia
3DMA CHRs (%)
Days
y = -2E-08x6 + 2E-06x5 - 0.0001x4 + 0.0021x3 - 0.0198x2 + 0.1246x + 0.3152
R2 = 0.9913
0
1
2
0
5
10
15
20
25
30
35
40

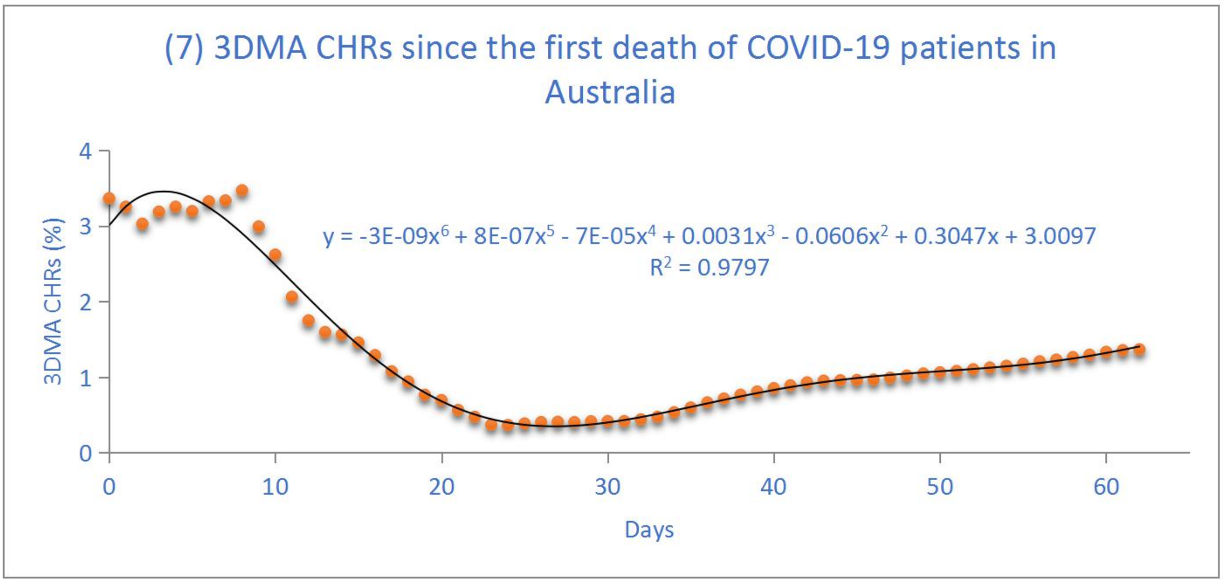

(7) 3DMA CHRs since the first death of COVID-19 patients in Australia
y = -3E-09x6 + 8E-07x5 - 7E-05x4 + 0.0031x3 - 0.0606x2 + 0.3047x + 3.0097
R2 = 0.9797
3DMA CHRs (%)
Days
0
1
2
3
4
10
20
30
40
50
60

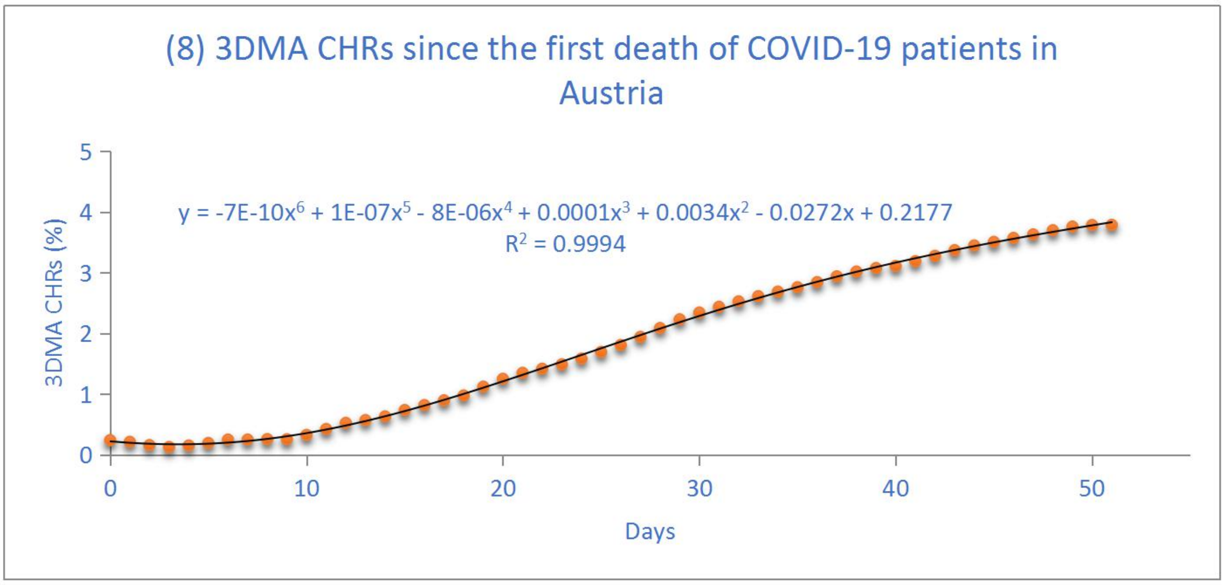

(8) 3DMA CHRs since the first death of COVID-19 patients in Austria
y = -7E-10x6 + 1E-07x5 - 8E-06x4 + 0.0001x3 + 0.0034x2 - 0.0272x + 0.2177
R2 = 0.9994
3DMA CHRs (%)
Days
0
1
2
3
4
5
10
20
30
40
50

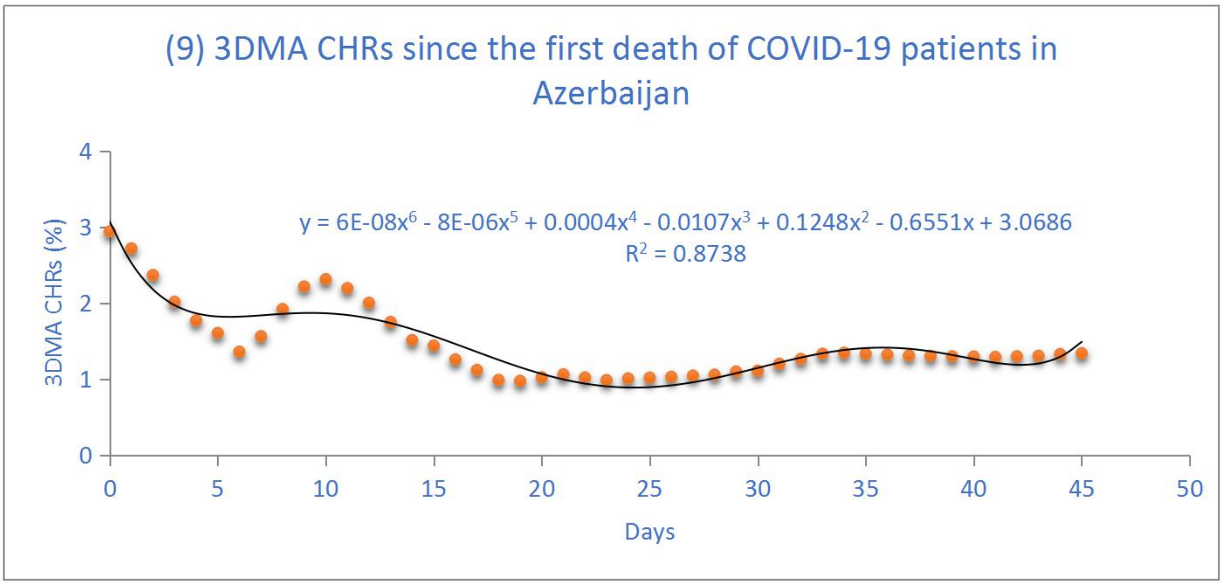

(9) 3DMA CHRs since the first death of COVID-19 patients in Azerbaijan
y = 6E-08x6 - 8E-06x5 + 0.0004x4 - 0.0107x3 + 0.1248x2 - 0.6551x + 3.0686
R2 = 0.8738
3DMA CHRs (%)
Days
0
1
2
3
4
5
10
15
20
25
30
35
40
45
50

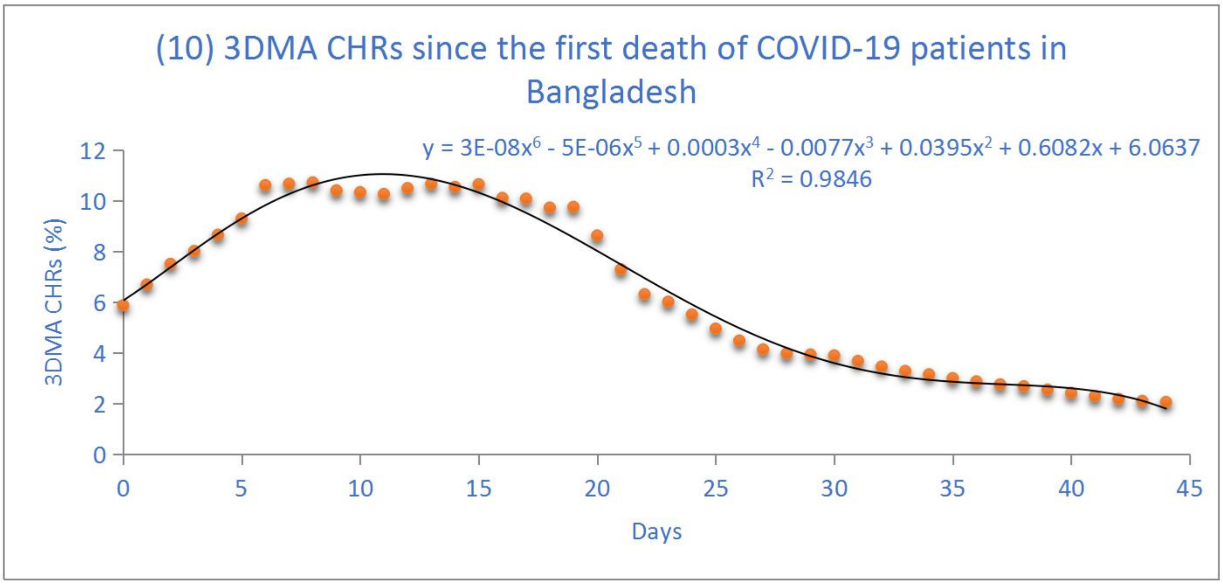

(10) 3DMA CHRs since the first death of COVID-19 patients in Bangladesh
y = 3E-08x6 - 5E-06x5 + 0.0003x4 - 0.0077x3 + 0.0395x2 + 0.6082x + 6.0637
R2 = 0.9846
3DMA CHRs (%)
Days

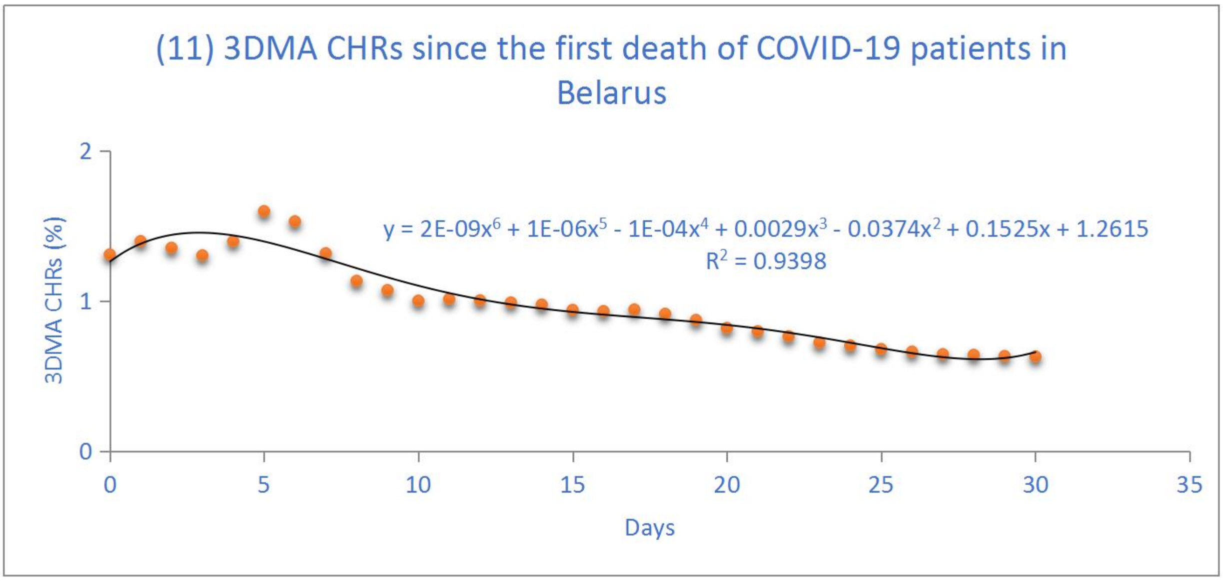

(11) 3DMA CHRs since the first death of COVID-19 patients in Belarus
y = 2E-09x6 + 1E-06x5 - 1E-04x4 + 0.0029x3 - 0.0374x2 + 0.1525x + 1.2615
R2 = 0.9398
3DMA CHRs (%)
Days

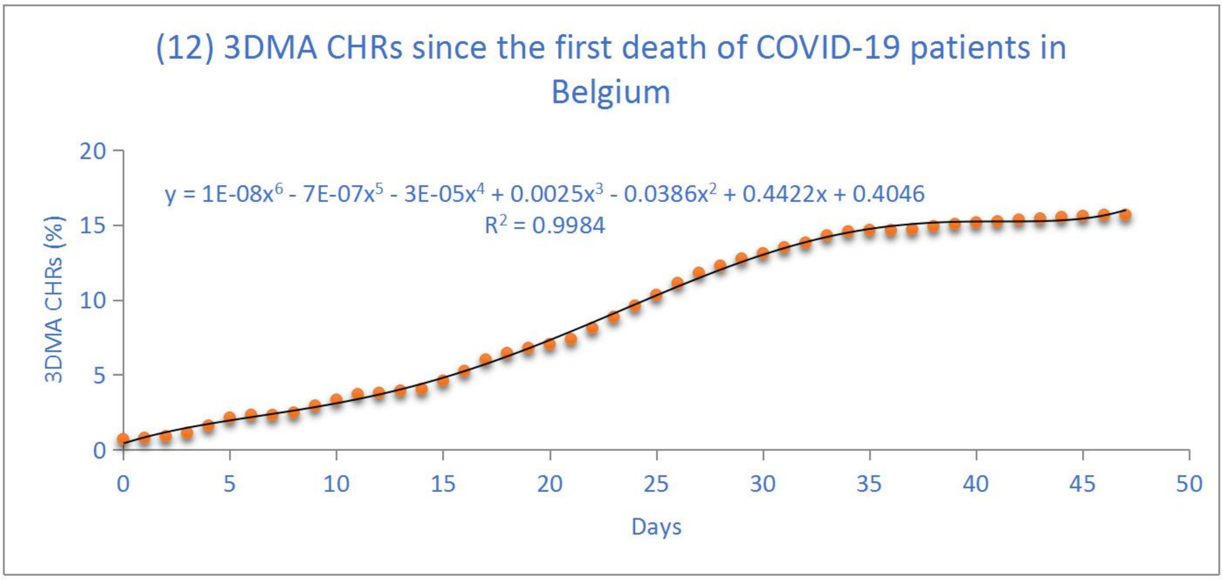

(12) 3DMA CHRs since the first death of COVID-19 patients in Belgium
y = 1E-08x6 - 7E-07x5 - 3E-05x4 + 0.0025x3 - 0.0386x2 + 0.4422x + 0.4046
R2 = 0.9984
3DMA CHRs (%)
Days

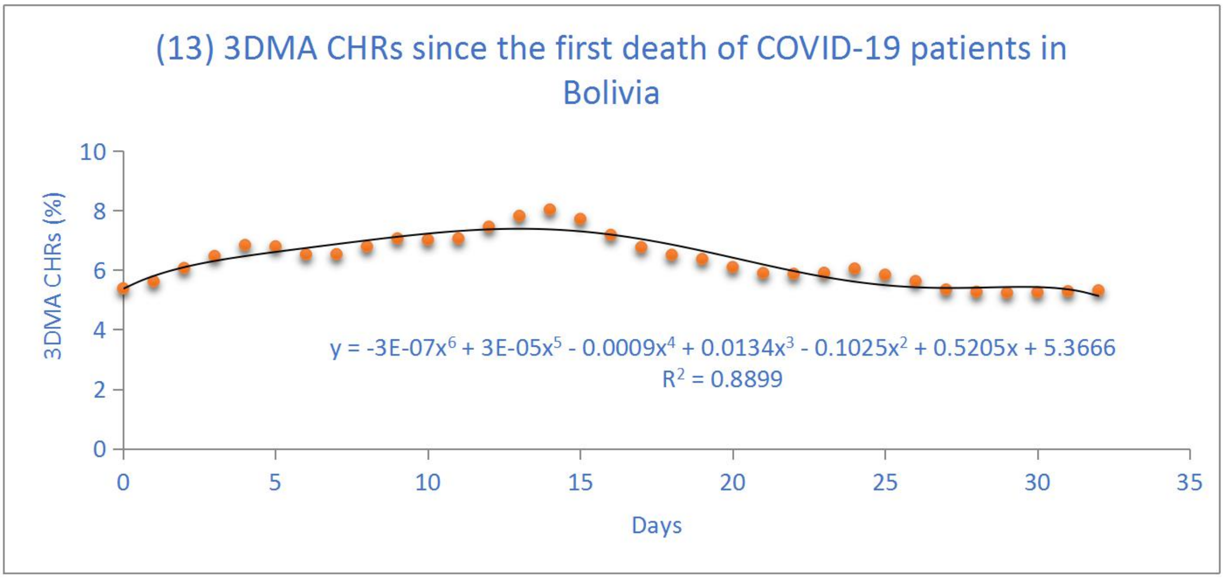
(13) 3DMA CHRs since the first death of COVID-19 patients in Bolivia
3DMA CHRs (%)
Days
$y = -3E-07x^6 + 3E-05x^5 - 0.0009x^4 + 0.0134x^3 - 0.1025x^2 + 0.5205x + 5.3666$
$R^2 = 0.8899$

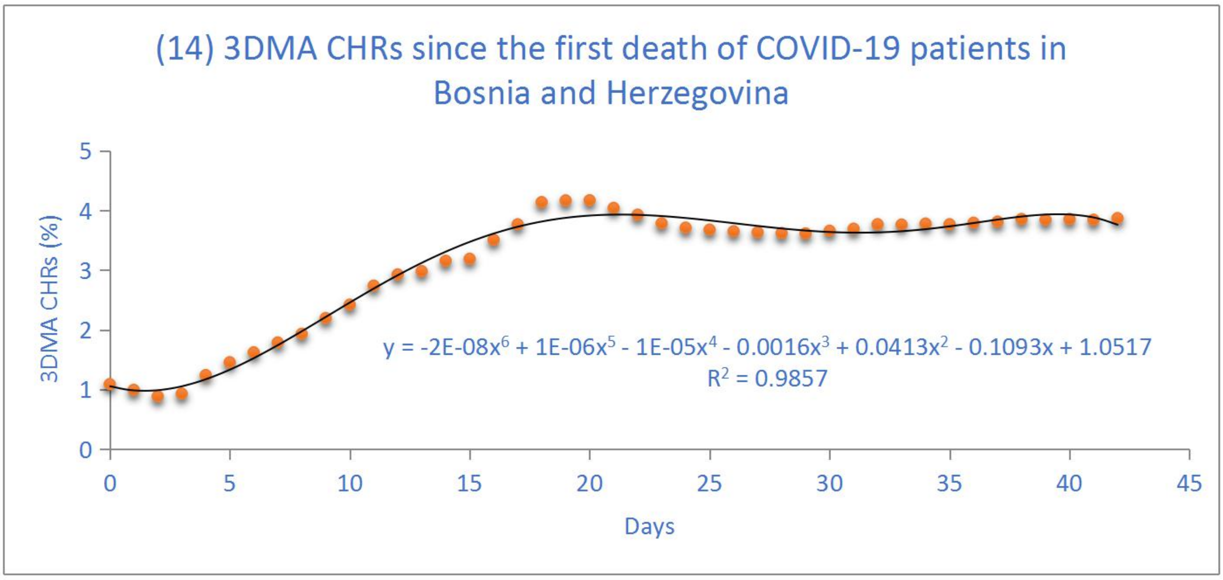
(14) 3DMA CHRs since the first death of COVID-19 patients in Bosnia and Herzegovina
3DMA CHRs (%)
Days
$y = -2E-08x^6 + 1E-06x^5 - 1E-05x^4 - 0.0016x^3 + 0.0413x^2 - 0.1093x + 1.0517$
$R^2 = 0.9857$

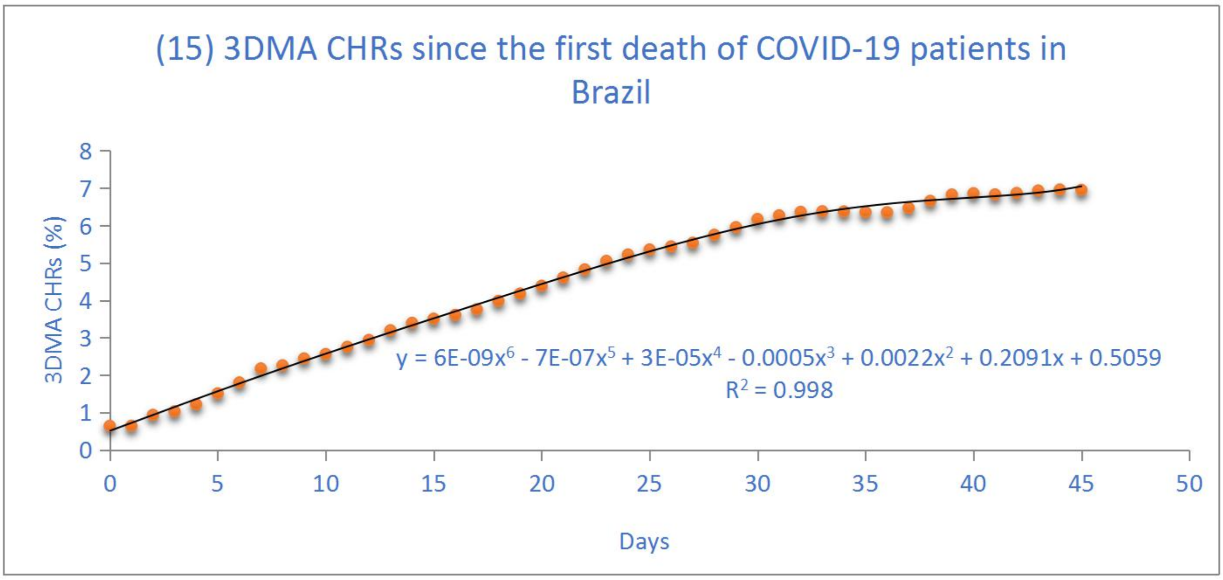
(15) 3DMA CHRs since the first death of COVID-19 patients in Brazil
3DMA CHRs (%)
Days
$y = 6E-09x^6 - 7E-07x^5 + 3E-05x^4 - 0.0005x^3 + 0.0022x^2 + 0.2091x + 0.5059$
$R^2 = 0.998$

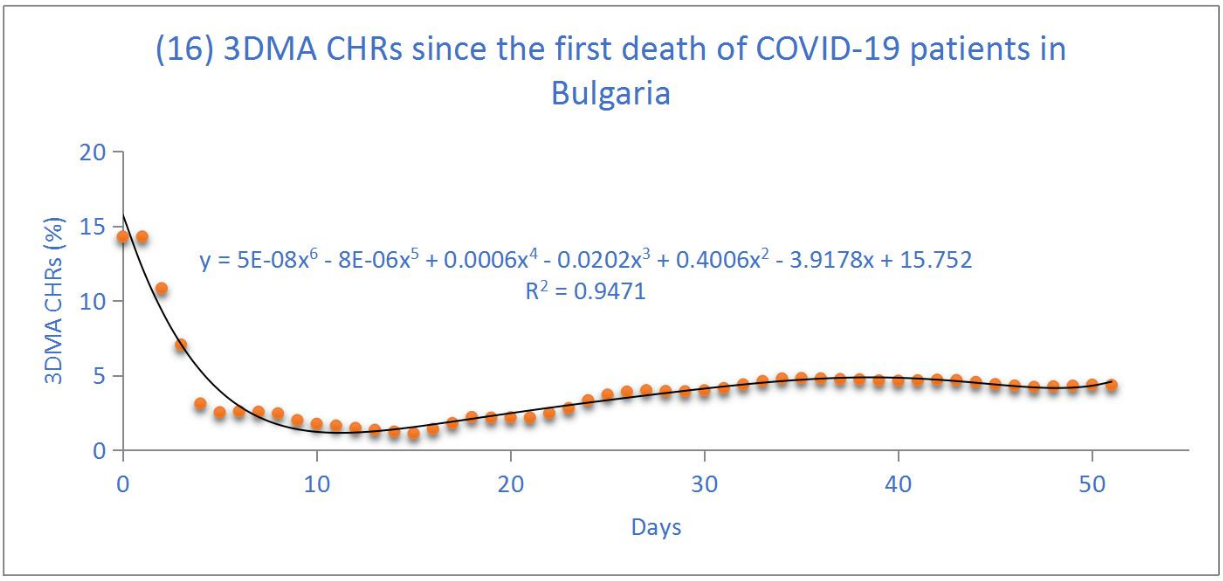
(16) 3DMA CHRs since the first death of COVID-19 patients in Bulgaria
y = 5E-08x6 - 8E-06x5 + 0.0006x4 - 0.0202x3 + 0.4006x2 - 3.9178x + 15.752
R2 = 0.9471
3DMA CHRs (%)
Days
0
5
10
15
20
30
40
50

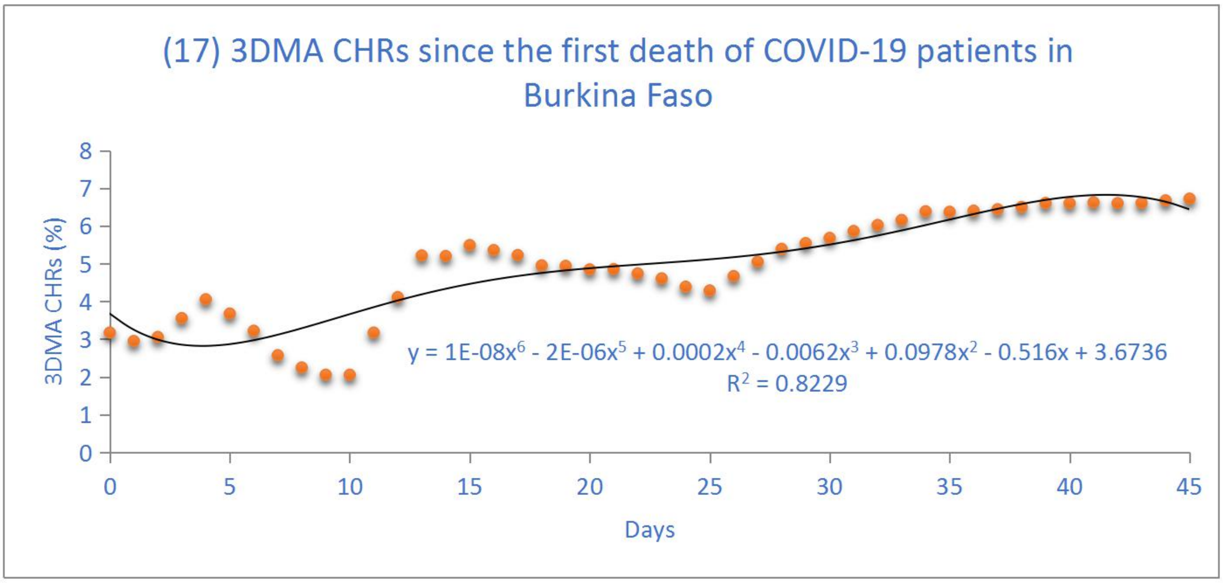
(17) 3DMA CHRs since the first death of COVID-19 patients in Burkina Faso
y = 1E-08x6 - 2E-06x5 + 0.0002x4 - 0.0062x3 + 0.0978x2 - 0.516x + 3.6736
R2 = 0.8229
3DMA CHRs (%)
Days
0
1
2
3
4
5
6
7
8
10
15
20
25
30
35
40
45

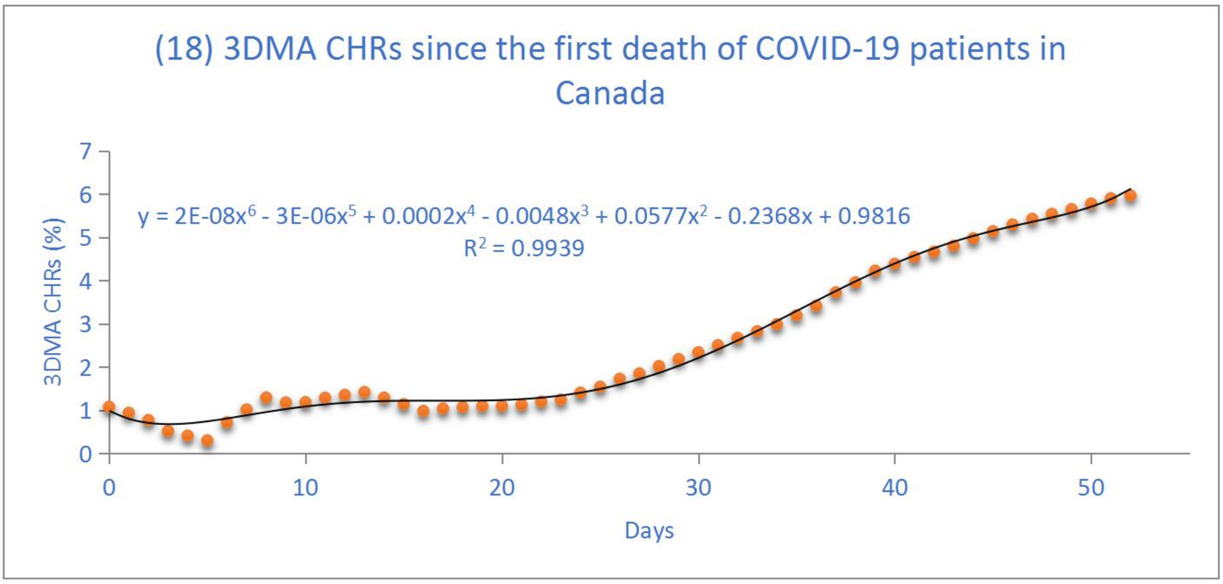
(18) 3DMA CHRs since the first death of COVID-19 patients in Canada
y = 2E-08x6 - 3E-06x5 + 0.0002x4 - 0.0048x3 + 0.0577x2 - 0.2368x + 0.9816
R2 = 0.9939
3DMA CHRs (%)
Days
0
1
2
3
4
5
6
7
10
20
30
40
50

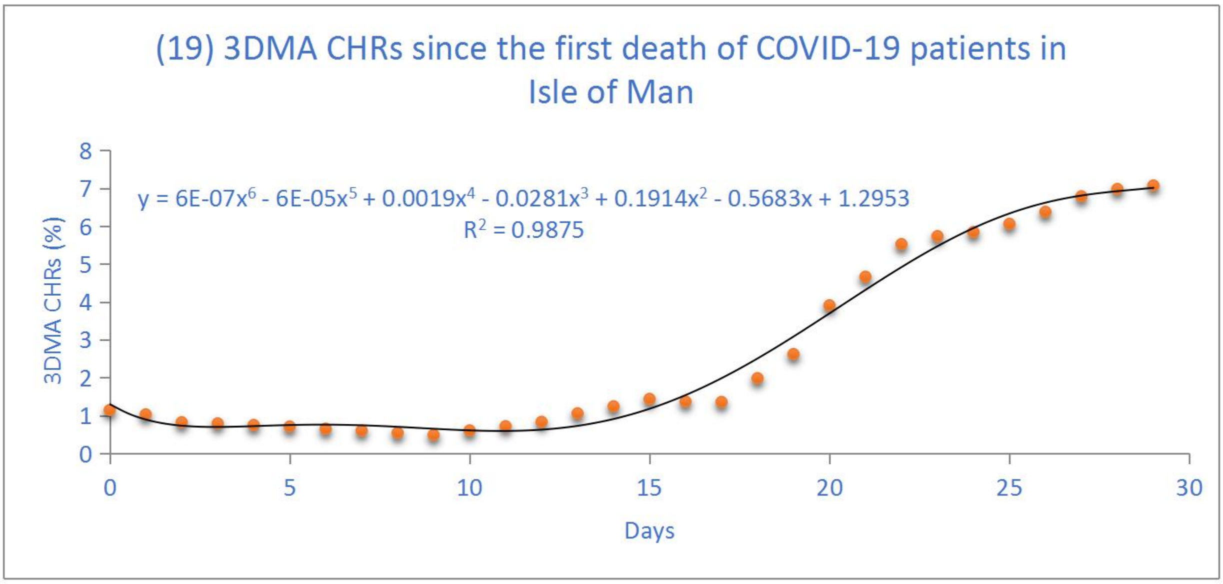
(19) 3DMA CHRs since the first death of COVID-19 patients in Isle of Man
y = 6E-07x6 - 6E-05x5 + 0.0019x4 - 0.0281x3 + 0.1914x2 - 0.5683x + 1.2953
R2 = 0.9875
3DMA CHRs (%)
Days

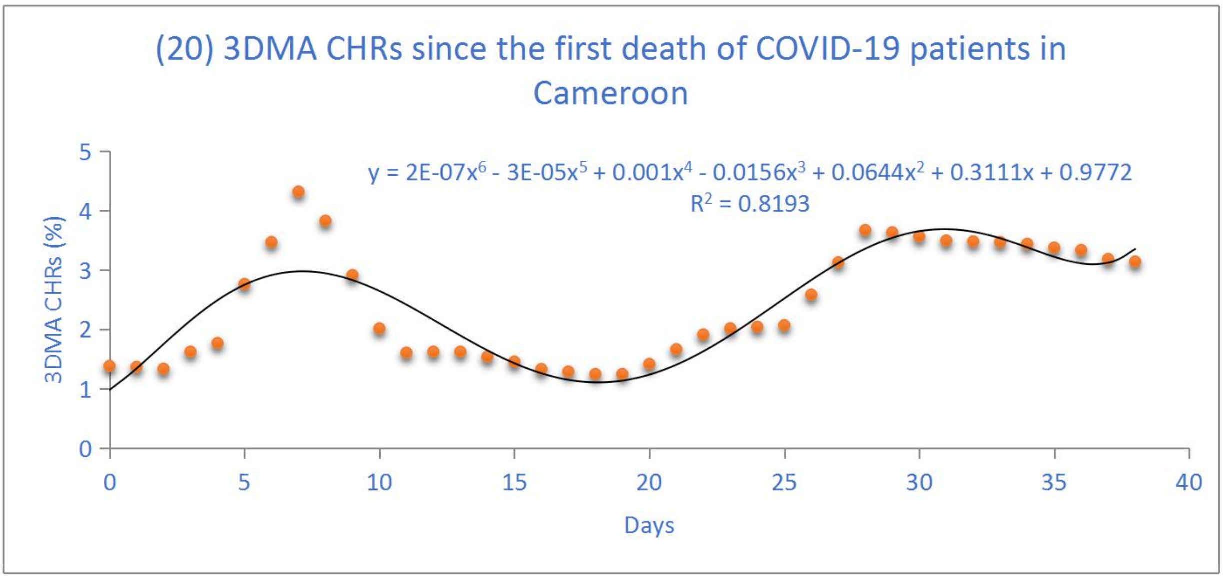
(20) 3DMA CHRs since the first death of COVID-19 patients in Cameroon
y = 2E-07x6 - 3E-05x5 + 0.001x4 - 0.0156x3 + 0.0644x2 + 0.3111x + 0.9772
R2 = 0.8193
3DMA CHRs (%)
Days

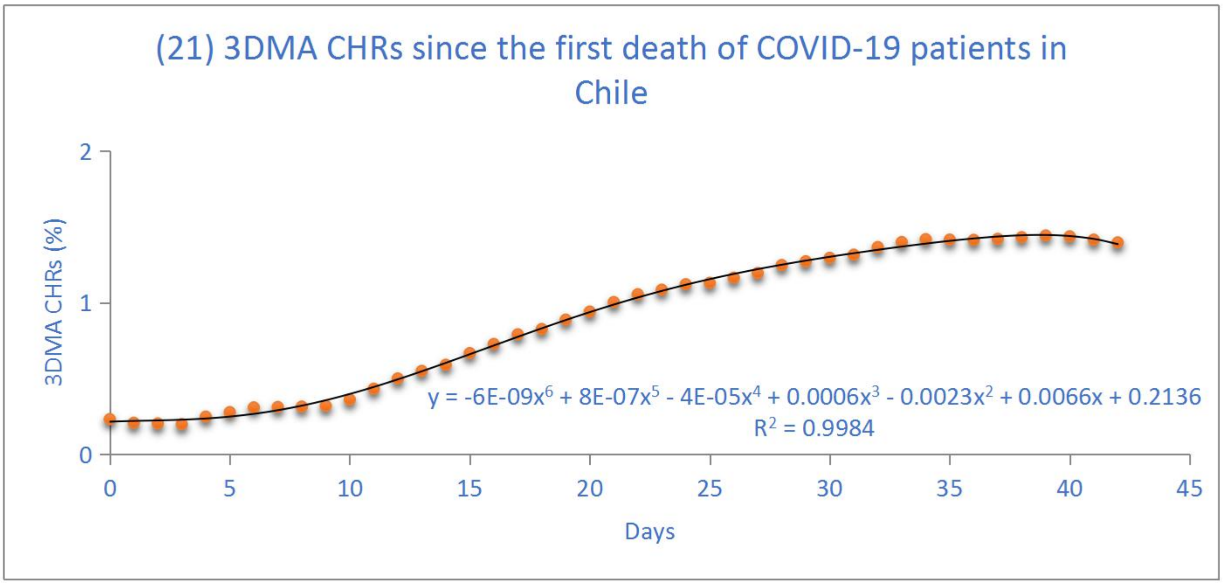
(21) 3DMA CHRs since the first death of COVID-19 patients in Chile
y = -6E-09x6 + 8E-07x5 - 4E-05x4 + 0.0006x3 - 0.0023x2 + 0.0066x + 0.2136
R2 = 0.9984
3DMA CHRs (%)
Days

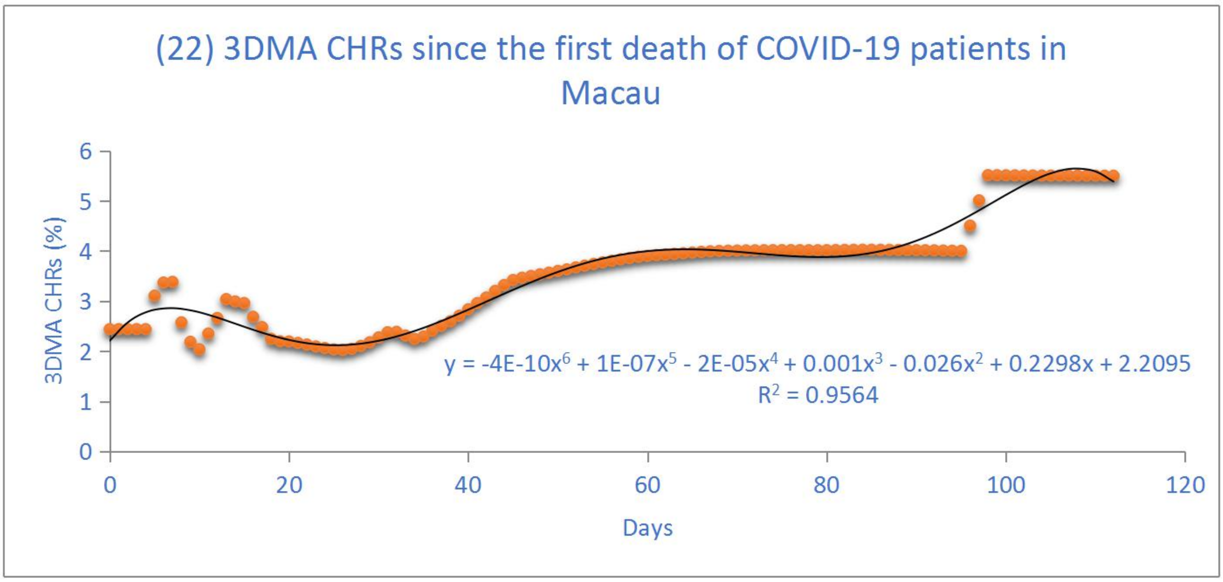
(22) 3DMA CHRs since the first death of COVID-19 patients in Macau
3DMA CHRs (%)
0
1
2
3
4
5
6
0
20
40
60
80
100
120
Days
y = -4E-10x6 + 1E-07x5 - 2E-05x4 + 0.001x3 - 0.026x2 + 0.2298x + 2.2095
R2 = 0.9564

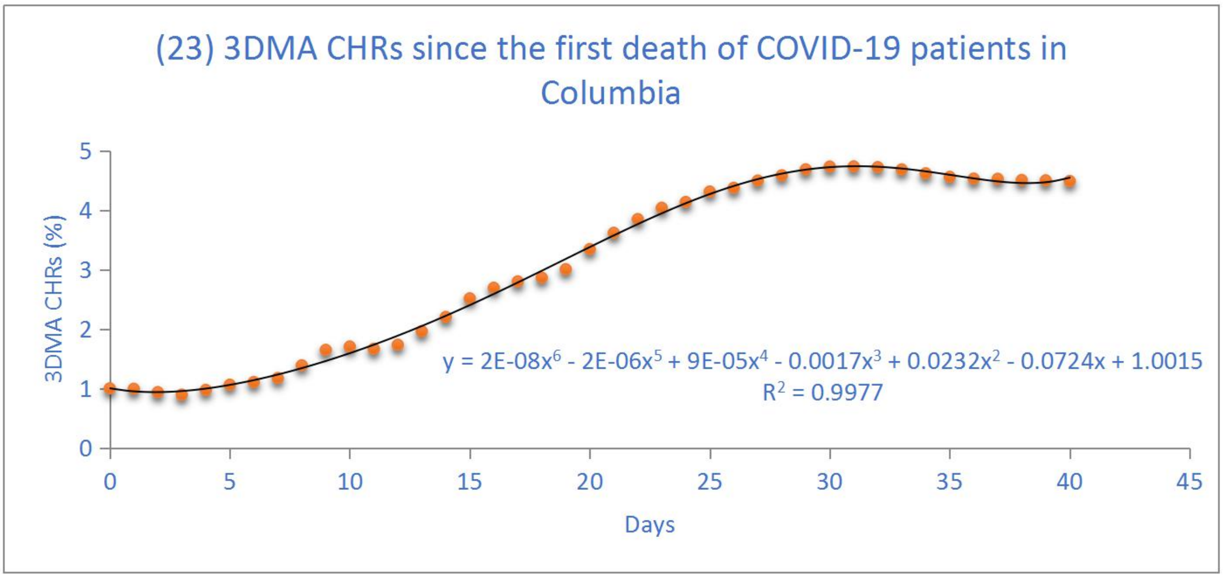
(23) 3DMA CHRs since the first death of COVID-19 patients in Columbia
3DMA CHRs (%)
0
1
2
3
4
5
0
5
10
15
20
25
30
35
40
45
Days
y = 2E-08x6 - 2E-06x5 + 9E-05x4 - 0.0017x3 + 0.0232x2 - 0.0724x + 1.0015
R2 = 0.9977

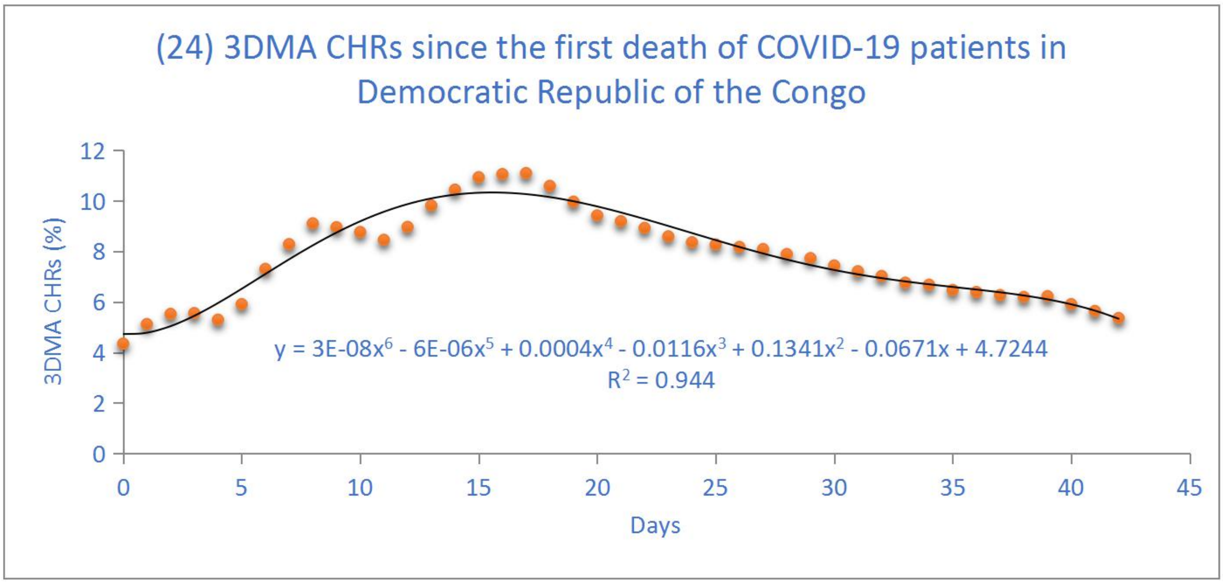
(24) 3DMA CHRs since the first death of COVID-19 patients in Democratic Republic of the Congo
3DMA CHRs (%)
0
2
4
6
8
10
12
0
5
10
15
20
25
30
35
40
45
Days
y = 3E-08x6 - 6E-06x5 + 0.0004x4 - 0.0116x3 + 0.1341x2 - 0.0671x + 4.7244
R2 = 0.944

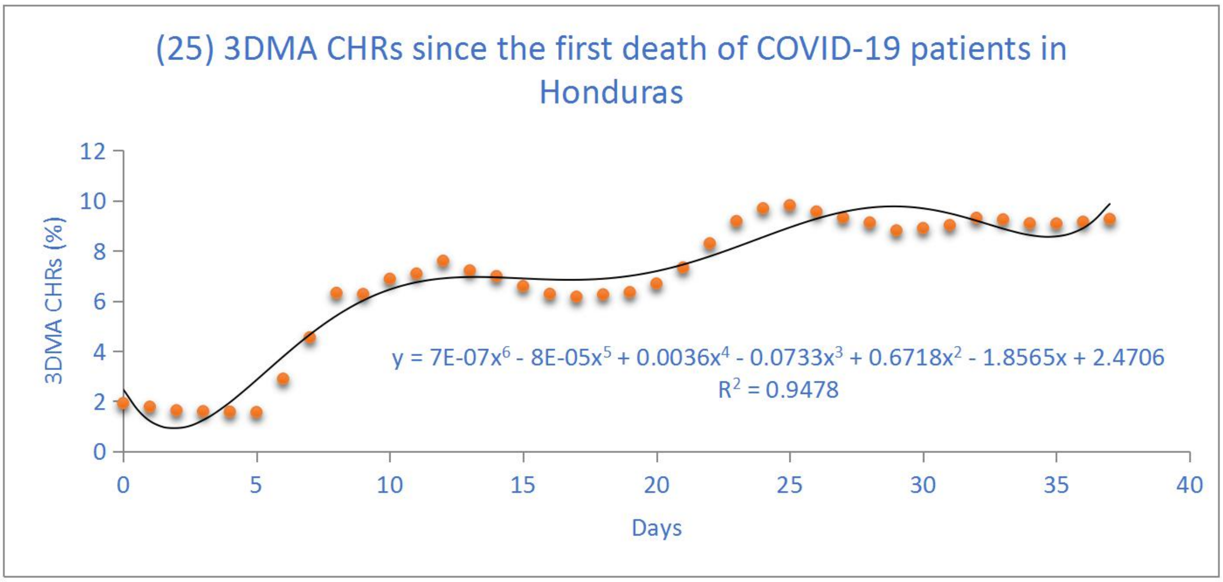
(25) 3DMA CHRs since the first death of COVID-19 patients in Honduras
3DMA CHRs (%)
Days
y = 7E-07x6 - 8E-05x5 + 0.0036x4 - 0.0733x3 + 0.6718x2 - 1.8565x + 2.4706
R2 = 0.9478

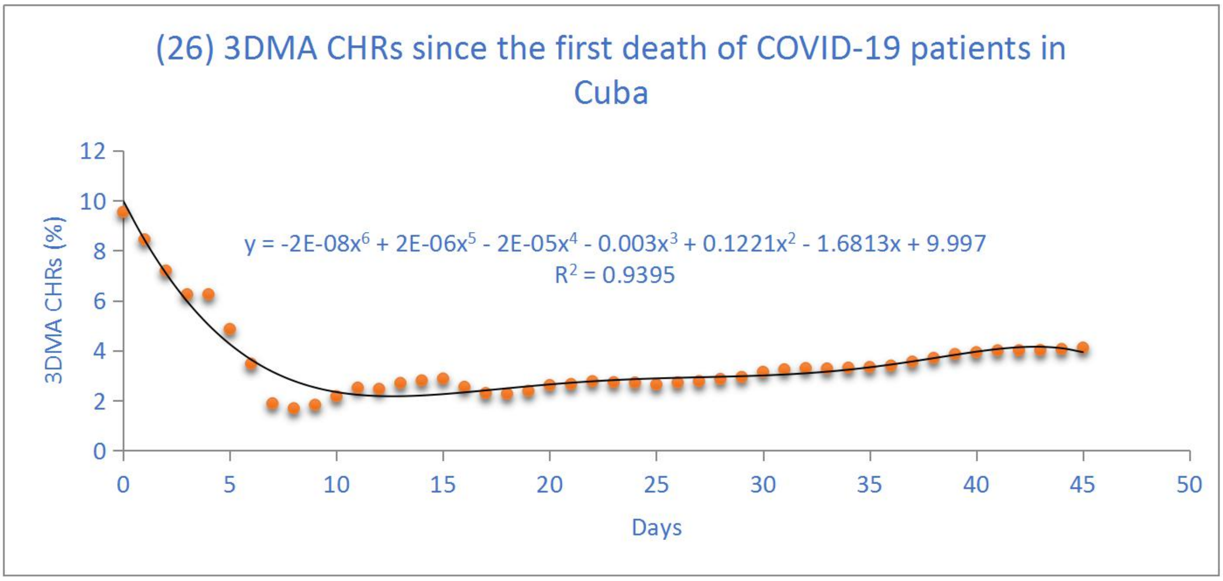
(26) 3DMA CHRs since the first death of COVID-19 patients in Cuba
3DMA CHRs (%)
Days
y = -2E-08x6 + 2E-06x5 - 2E-05x4 - 0.003x3 + 0.1221x2 - 1.6813x + 9.997
R2 = 0.9395

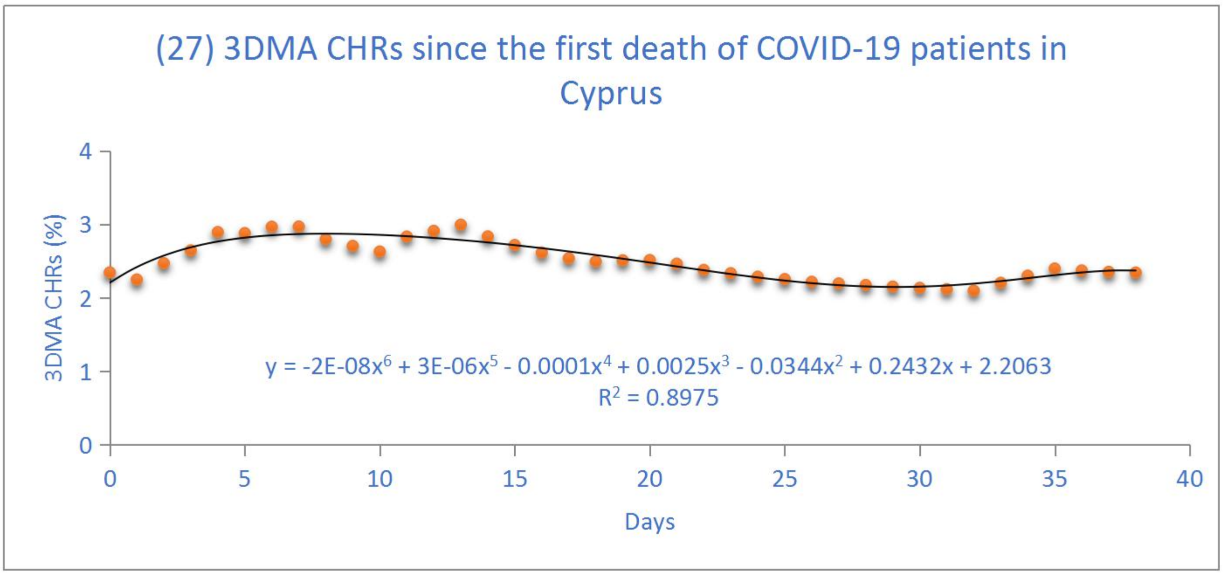
(27) 3DMA CHRs since the first death of COVID-19 patients in Cyprus
3DMA CHRs (%)
Days
y = -2E-08x6 + 3E-06x5 - 0.0001x4 + 0.0025x3 - 0.0344x2 + 0.2432x + 2.2063
R2 = 0.8975

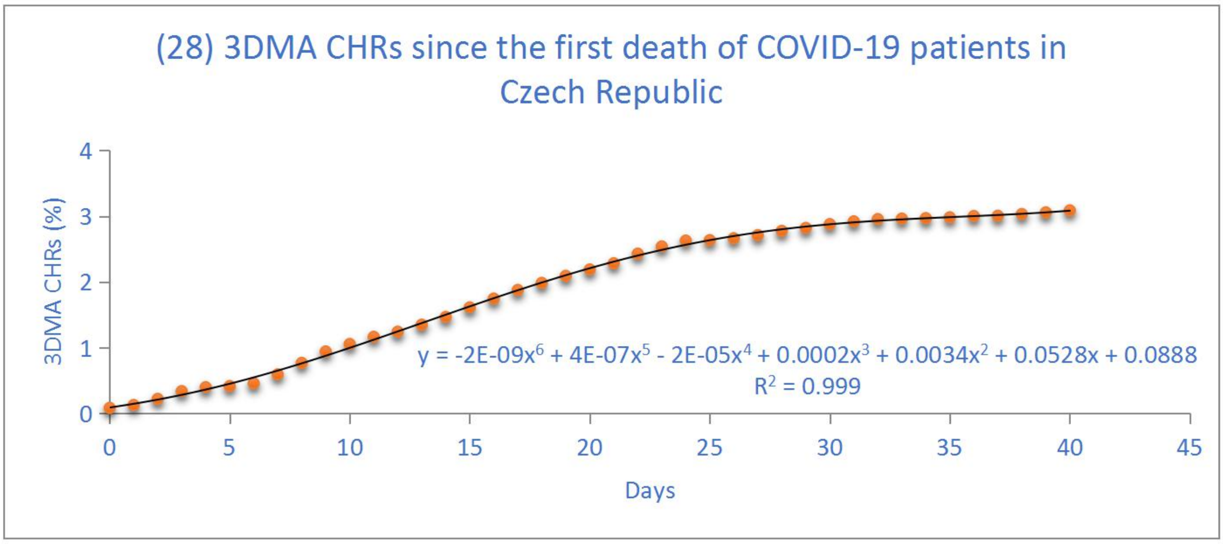

(28) 3DMA CHRs since the first death of COVID-19 patients in Czech Republic
3DMA CHRs (%)
Days
y = -2E-09x⁶ + 4E-07x⁵ - 2E-05x⁴ + 0.0002x³ + 0.0034x² + 0.0528x + 0.0888
R² = 0.999
0
1
2
3
4
5
10
15
20
25
30
35
40
45

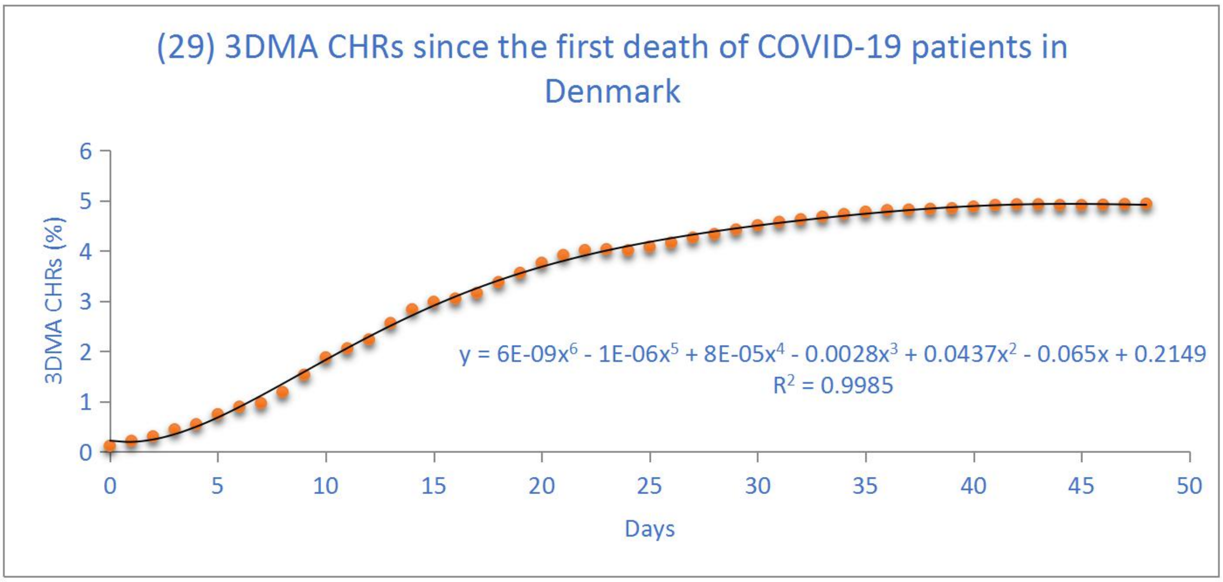

(29) 3DMA CHRs since the first death of COVID-19 patients in Denmark
3DMA CHRs (%)
Days
y = 6E-09x⁶ - 1E-06x⁵ + 8E-05x⁴ - 0.0028x³ + 0.0437x² - 0.065x + 0.2149
R² = 0.9985
0
1
2
3
4
5
6
10
15
20
25
30
35
40
45
50

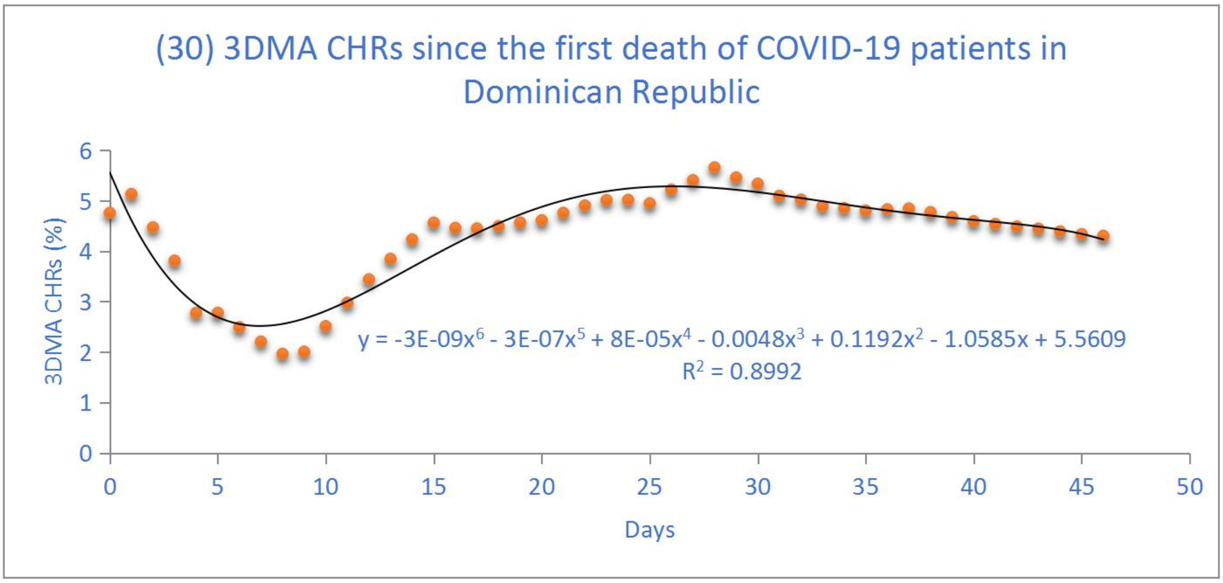

(30) 3DMA CHRs since the first death of COVID-19 patients in Dominican Republic
3DMA CHRs (%)
Days
y = -3E-09x⁶ - 3E-07x⁵ + 8E-05x⁴ - 0.0048x³ + 0.1192x² - 1.0585x + 5.5609
R² = 0.8992
0
1
2
3
4
5
6
10
15
20
25
30
35
40
45
50

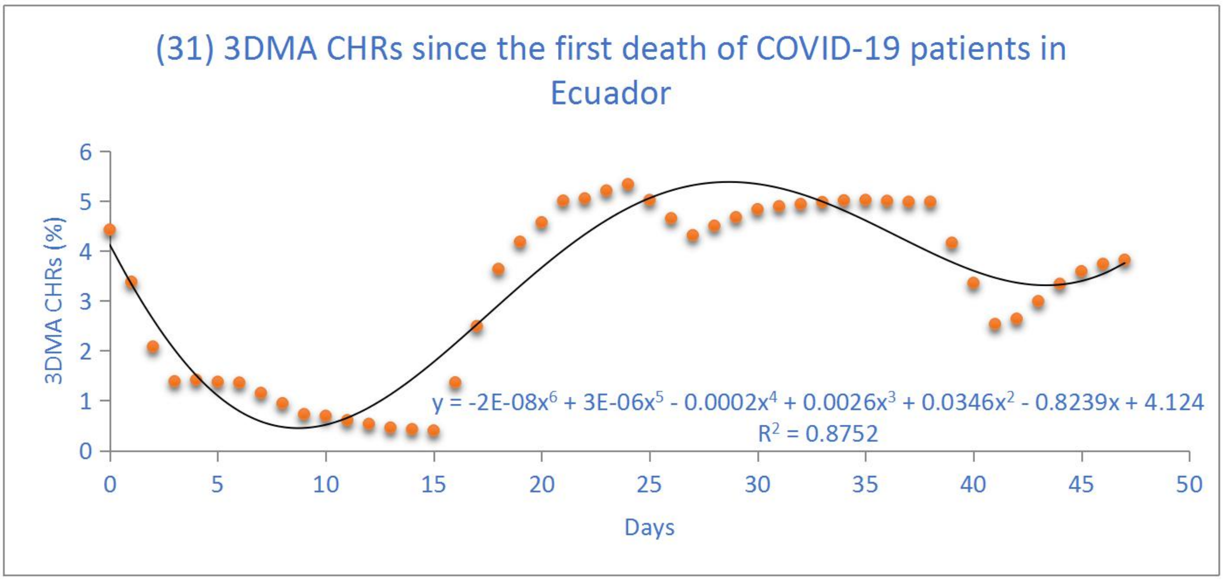

(31) 3DMA CHRs since the first death of COVID-19 patients in Ecuador
3DMA CHRs (%)
Days
y = -2E-08x6 + 3E-06x5 - 0.0002x4 + 0.0026x3 + 0.0346x2 - 0.8239x + 4.124
R2 = 0.8752

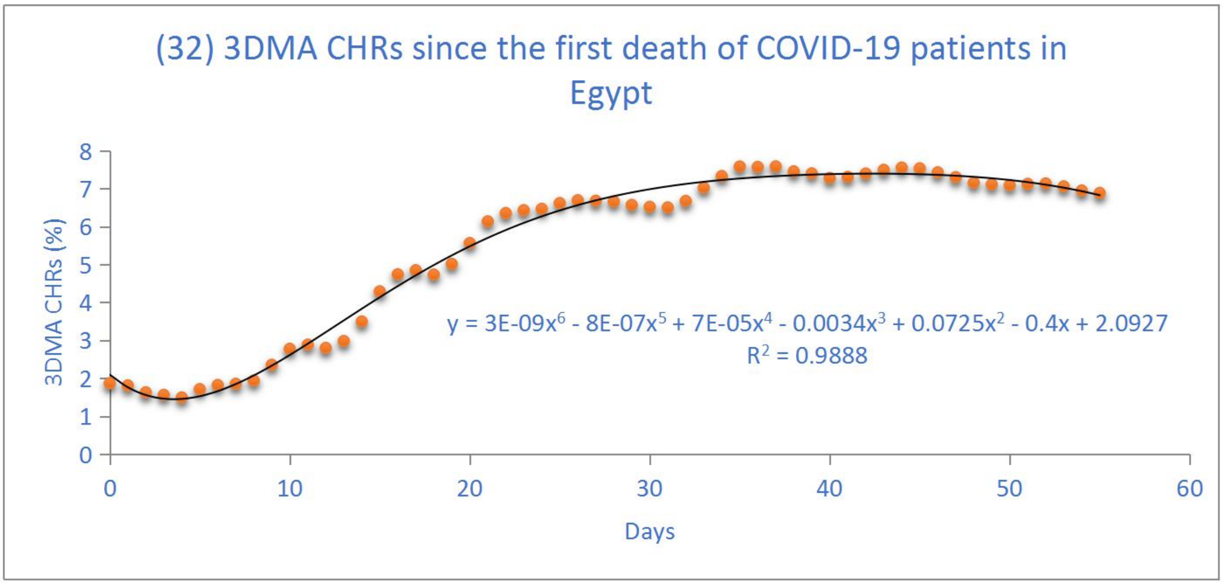

(32) 3DMA CHRs since the first death of COVID-19 patients in Egypt
3DMA CHRs (%)
Days
y = 3E-09x6 - 8E-07x5 + 7E-05x4 - 0.0034x3 + 0.0725x2 - 0.4x + 2.0927
R2 = 0.9888

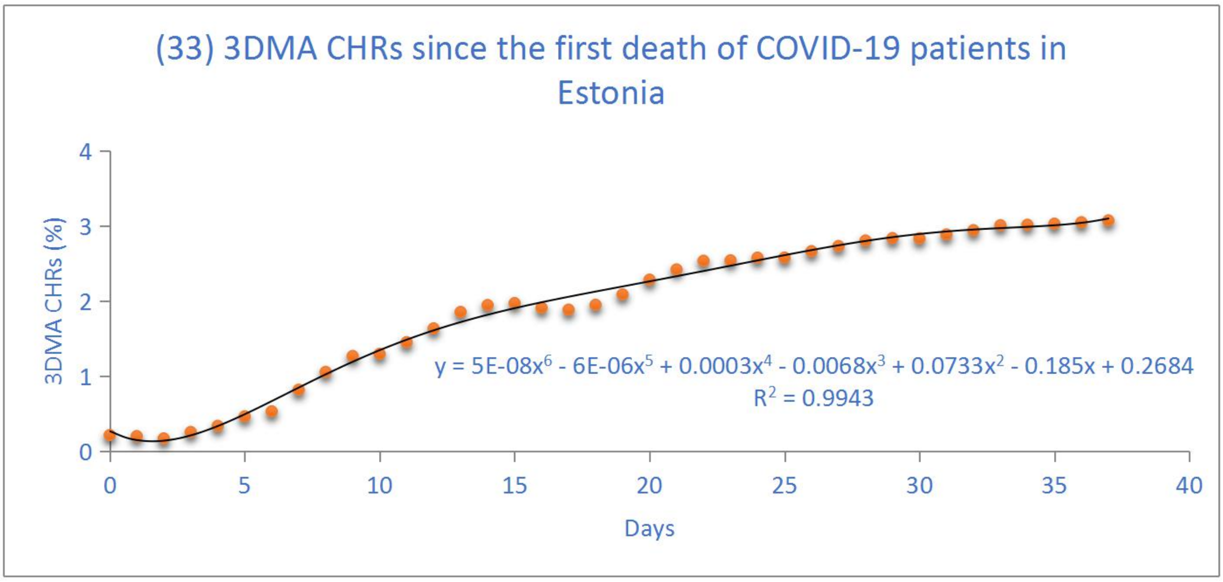

(33) 3DMA CHRs since the first death of COVID-19 patients in Estonia
3DMA CHRs (%)
Days
y = 5E-08x6 - 6E-06x5 + 0.0003x4 - 0.0068x3 + 0.0733x2 - 0.185x + 0.2684
R2 = 0.9943

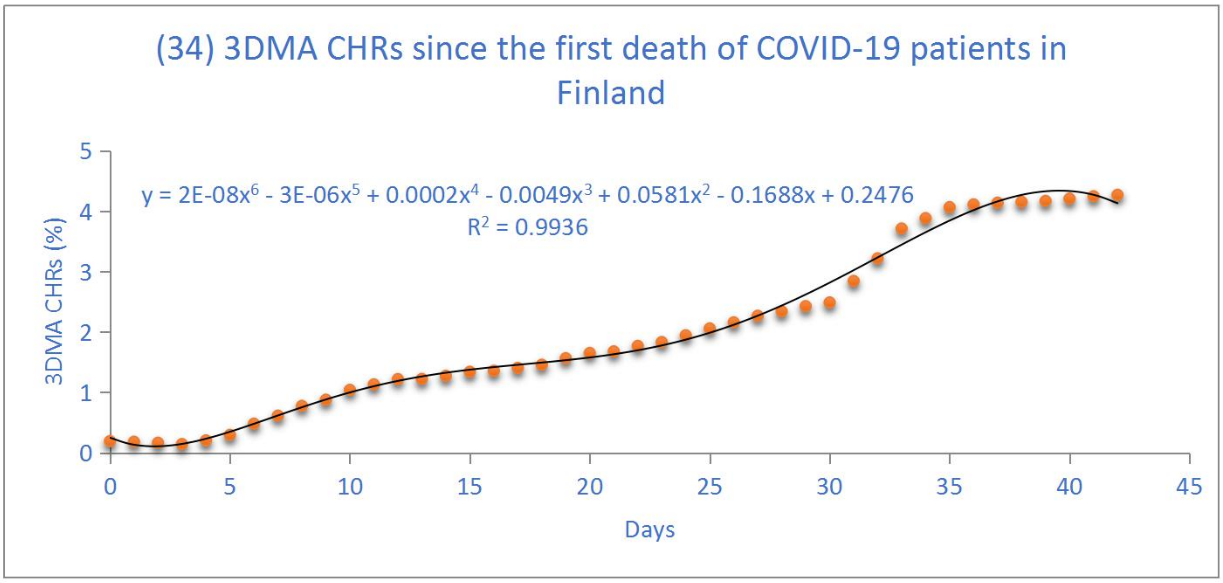
(34) 3DMA CHRs since the first death of COVID-19 patients in Finland
y = 2E-08x6 - 3E-06x5 + 0.0002x4 - 0.0049x3 + 0.0581x2 - 0.1688x + 0.2476
R2 = 0.9936
3DMA CHRs (%)
Days
0
1
2
3
4
5
0
5
10
15
20
25
30
35
40
45

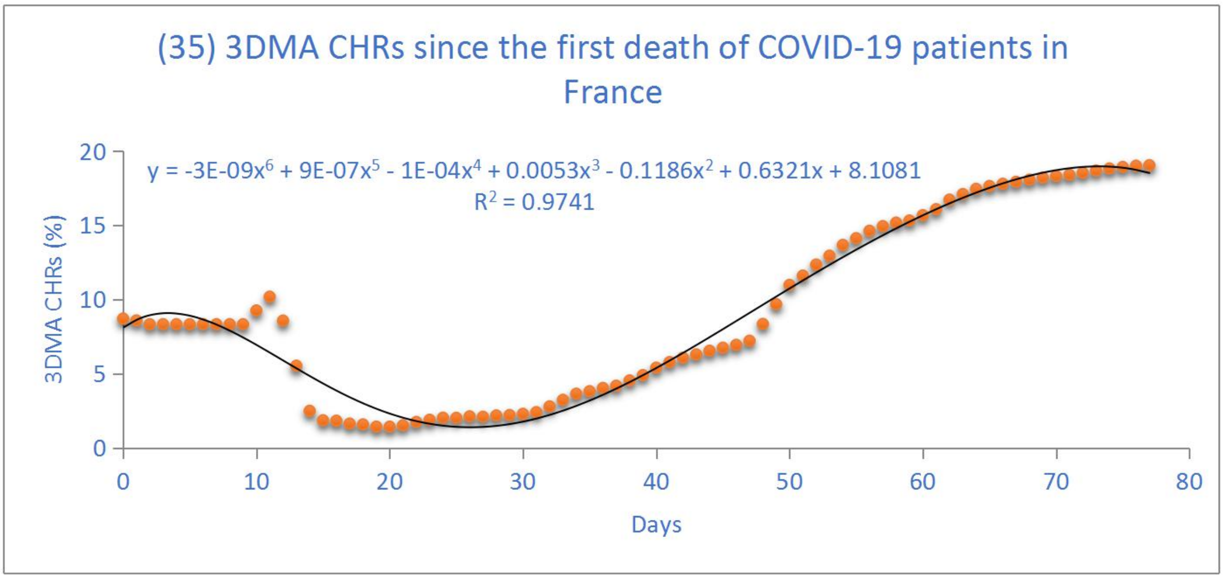
(35) 3DMA CHRs since the first death of COVID-19 patients in France
y = -3E-09x6 + 9E-07x5 - 1E-04x4 + 0.0053x3 - 0.1186x2 + 0.6321x + 8.1081
R2 = 0.9741
3DMA CHRs (%)
Days
0
5
10
15
20
0
10
20
30
40
50
60
70
80

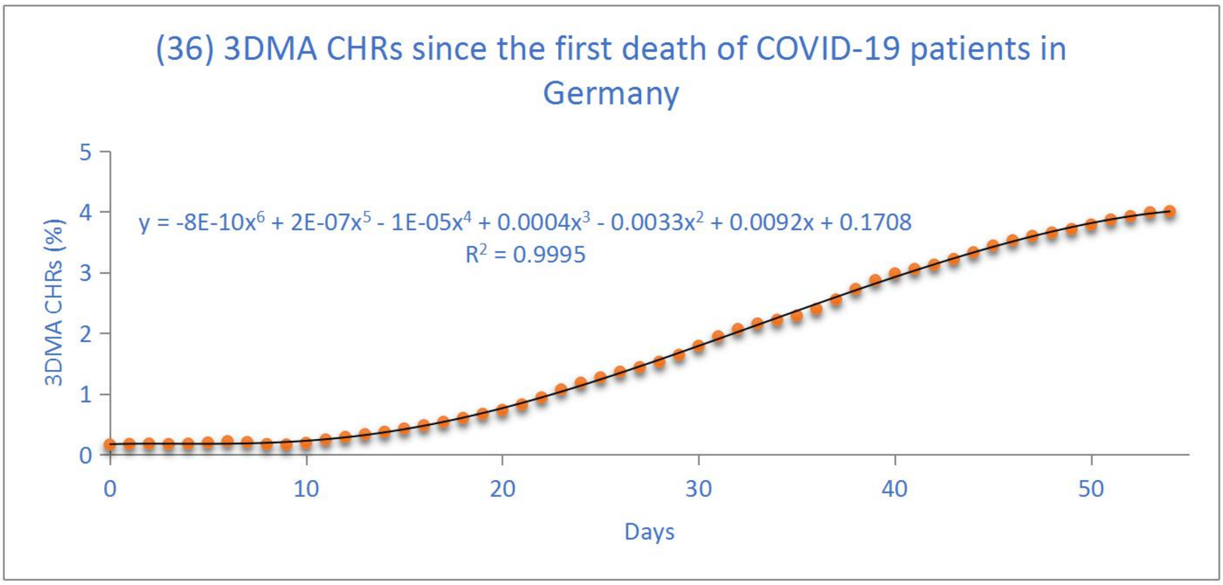
(36) 3DMA CHRs since the first death of COVID-19 patients in Germany
y = -8E-10x6 + 2E-07x5 - 1E-05x4 + 0.0004x3 - 0.0033x2 + 0.0092x + 0.1708
R2 = 0.9995
3DMA CHRs (%)
Days
0
1
2
3
4
5
0
10
20
30
40
50

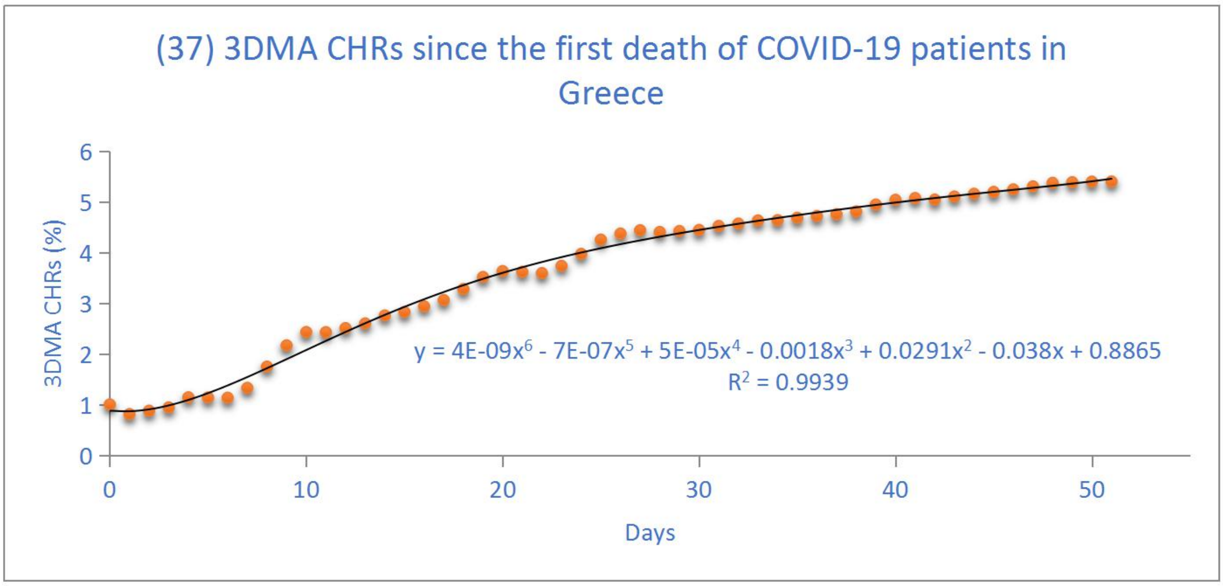
(37) 3DMA CHRs since the first death of COVID-19 patients in Greece
3DMA CHRs (%)
Days
y = 4E-09x6 - 7E-07x5 + 5E-05x4 - 0.0018x3 + 0.0291x2 - 0.038x + 0.8865
R2 = 0.9939
0
1
2
3
4
5
6
0
10
20
30
40
50

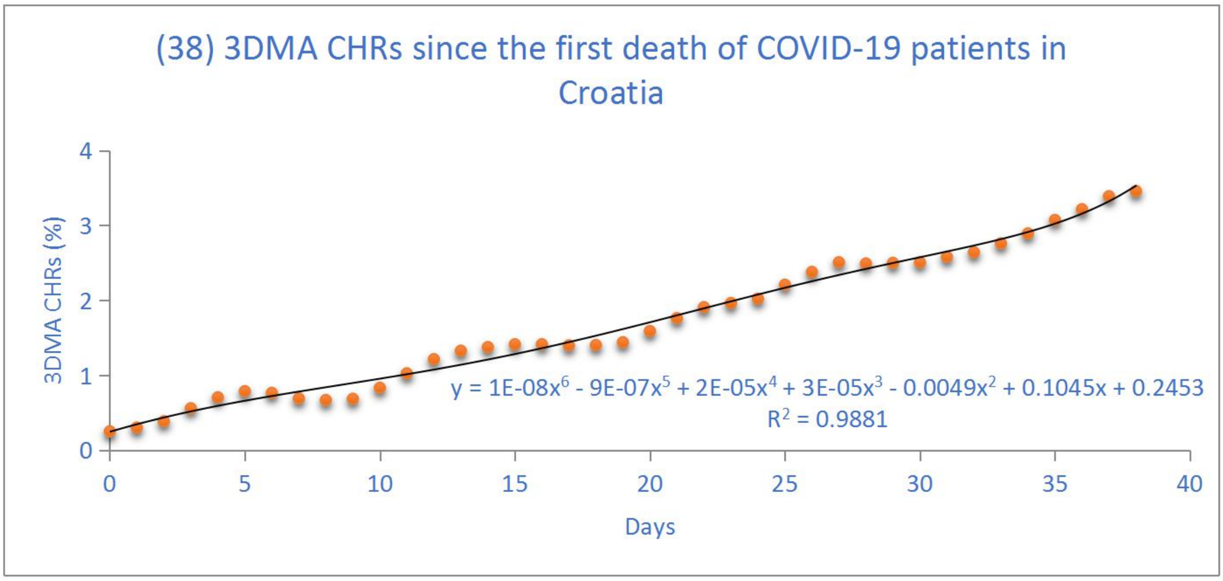
(38) 3DMA CHRs since the first death of COVID-19 patients in Croatia
3DMA CHRs (%)
Days
y = 1E-08x6 - 9E-07x5 + 2E-05x4 + 3E-05x3 - 0.0049x2 + 0.1045x + 0.2453
R2 = 0.9881
0
1
2
3
4
0
5
10
15
20
25
30
35
40

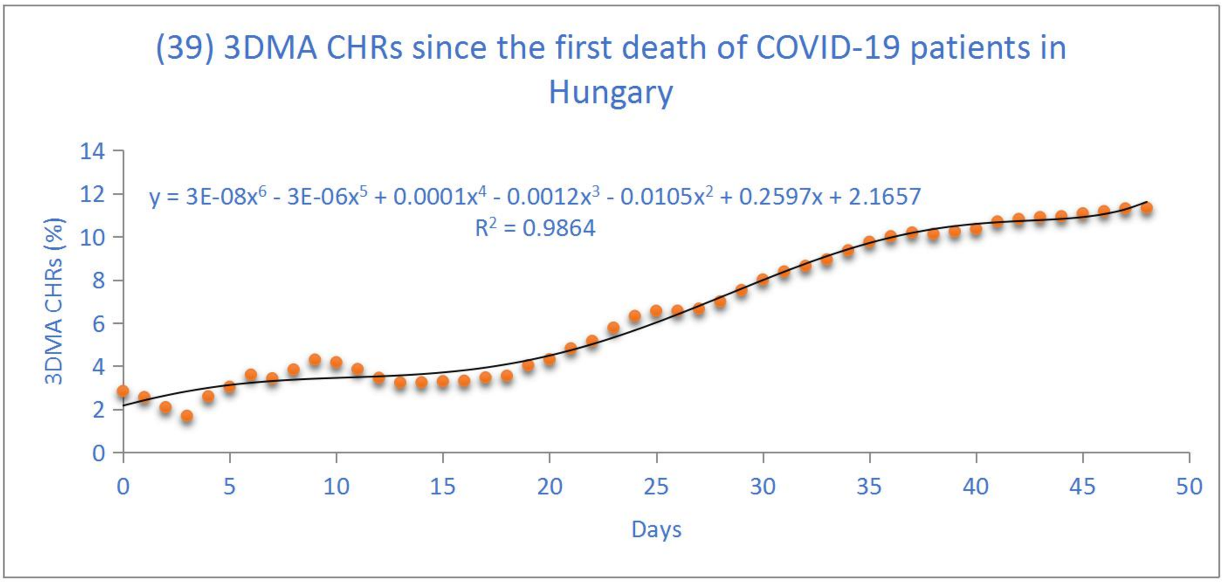
(39) 3DMA CHRs since the first death of COVID-19 patients in Hungary
3DMA CHRs (%)
Days
y = 3E-08x6 - 3E-06x5 + 0.0001x4 - 0.0012x3 - 0.0105x2 + 0.2597x + 2.1657
R2 = 0.9864
0
2
4
6
8
10
12
14
0
5
10
15
20
25
30
35
40
45
50

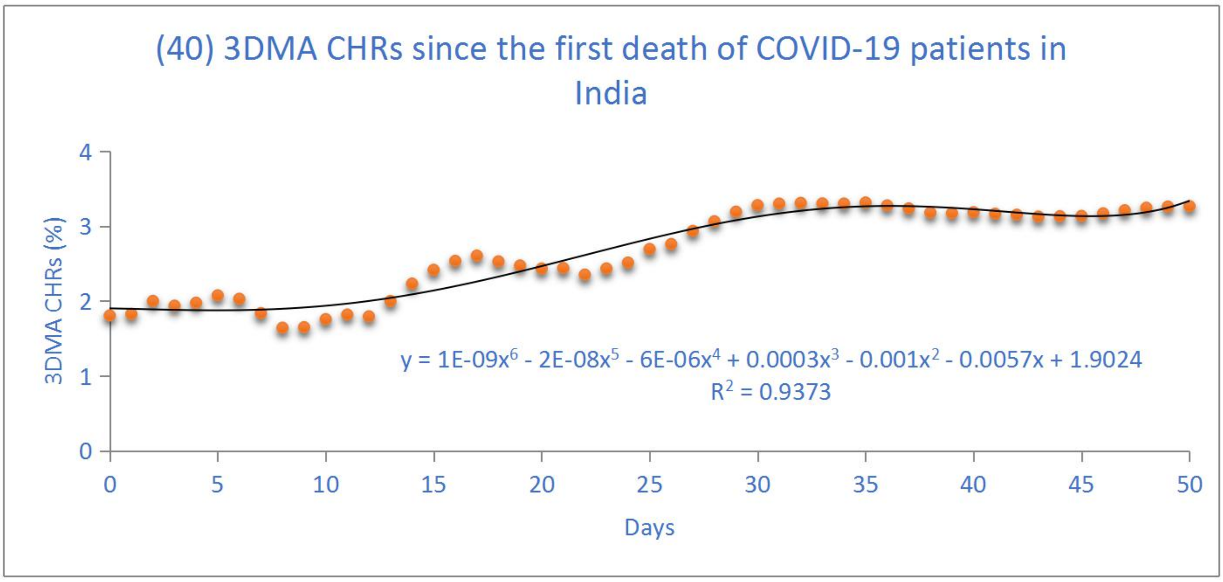
(40) 3DMA CHRs since the first death of COVID-19 patients in India
3DMA CHRs (%)
4
3
2
1
0
0
5
10
15
20
25
30
35
40
45
50
Days
y = 1E-09x6 - 2E-08x5 - 6E-06x4 + 0.0003x3 - 0.001x2 - 0.0057x + 1.9024
R2 = 0.9373

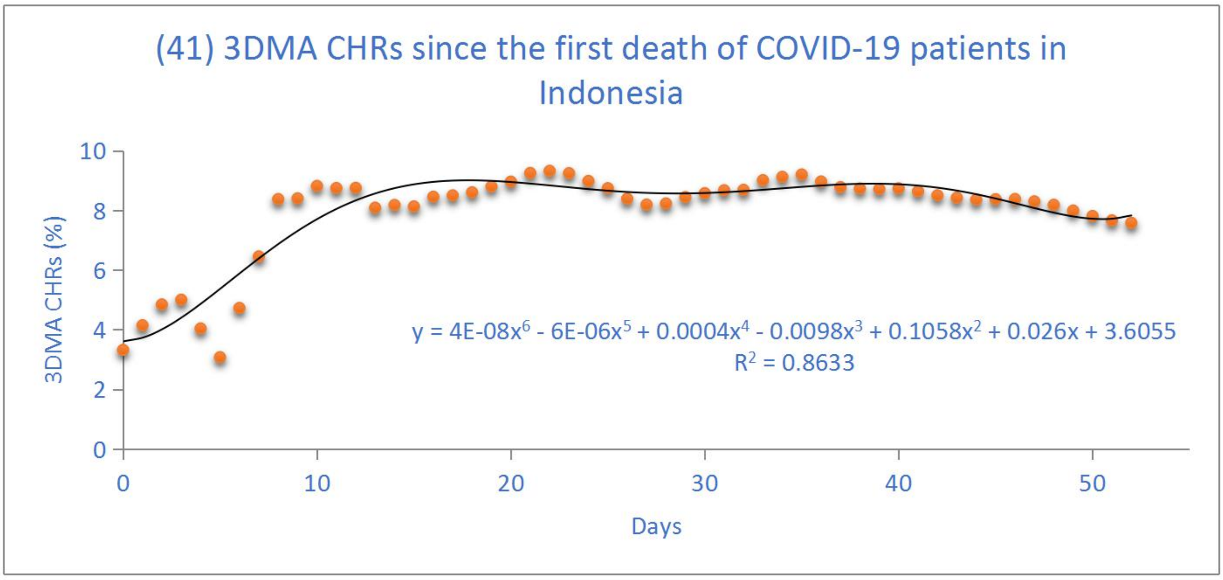
(41) 3DMA CHRs since the first death of COVID-19 patients in Indonesia
3DMA CHRs (%)
10
8
6
4
2
0
0
10
20
30
40
50
Days
y = 4E-08x6 - 6E-06x5 + 0.0004x4 - 0.0098x3 + 0.1058x2 + 0.026x + 3.6055
R2 = 0.8633

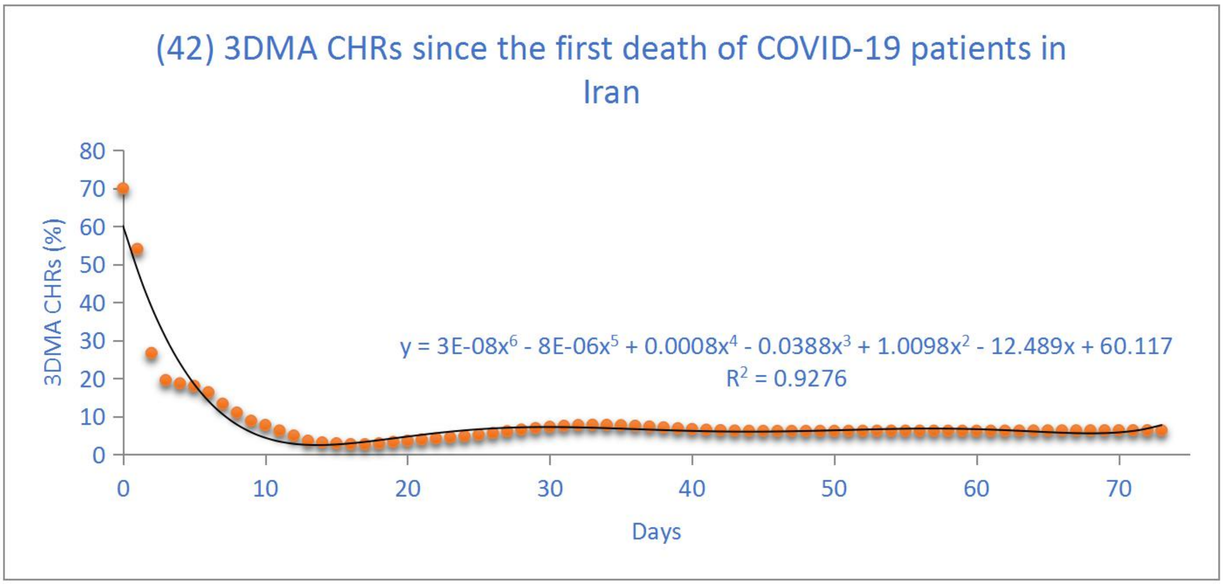
(42) 3DMA CHRs since the first death of COVID-19 patients in Iran
3DMA CHRs (%)
80
70
60
50
40
30
20
10
0
0
10
20
30
40
50
60
70
Days
y = 3E-08x6 - 8E-06x5 + 0.0008x4 - 0.0388x3 + 1.0098x2 - 12.489x + 60.117
R2 = 0.9276

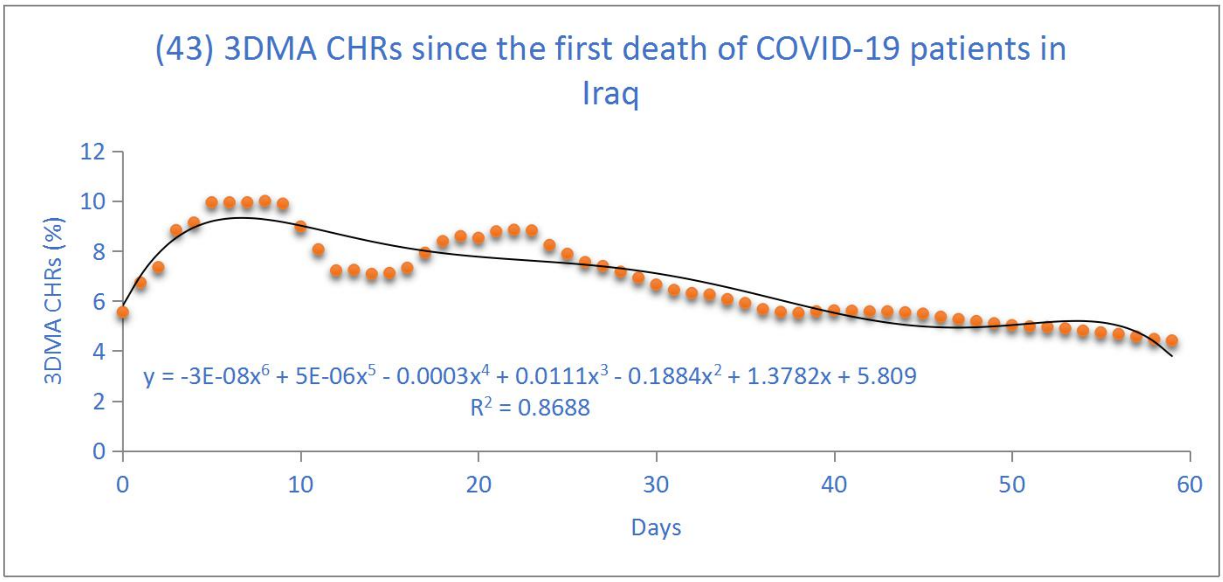
(43) 3DMA CHRs since the first death of COVID-19 patients in Iraq
y = -3E-08x6 + 5E-06x5 - 0.0003x4 + 0.0111x3 - 0.1884x2 + 1.3782x + 5.809
R2 = 0.8688
3DMA CHRs (%)
Days
12
10
8
6
4
2
0
0
10
20
30
40
50
60

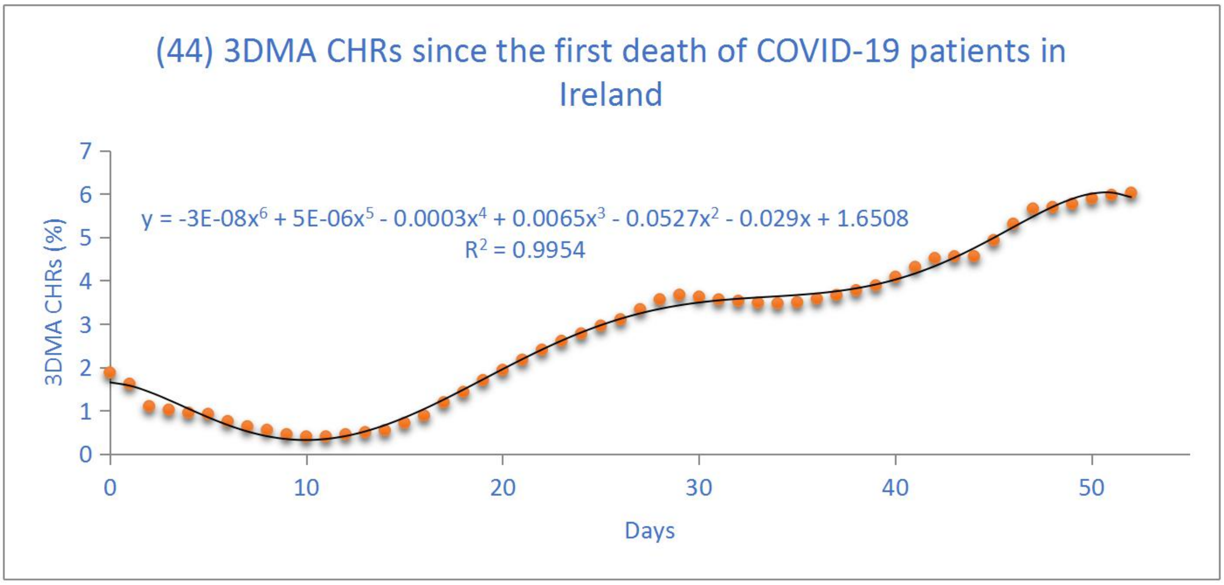
(44) 3DMA CHRs since the first death of COVID-19 patients in Ireland
y = -3E-08x6 + 5E-06x5 - 0.0003x4 + 0.0065x3 - 0.0527x2 - 0.029x + 1.6508
R2 = 0.9954
3DMA CHRs (%)
Days
7
6
5
4
3
2
1
0
0
10
20
30
40
50

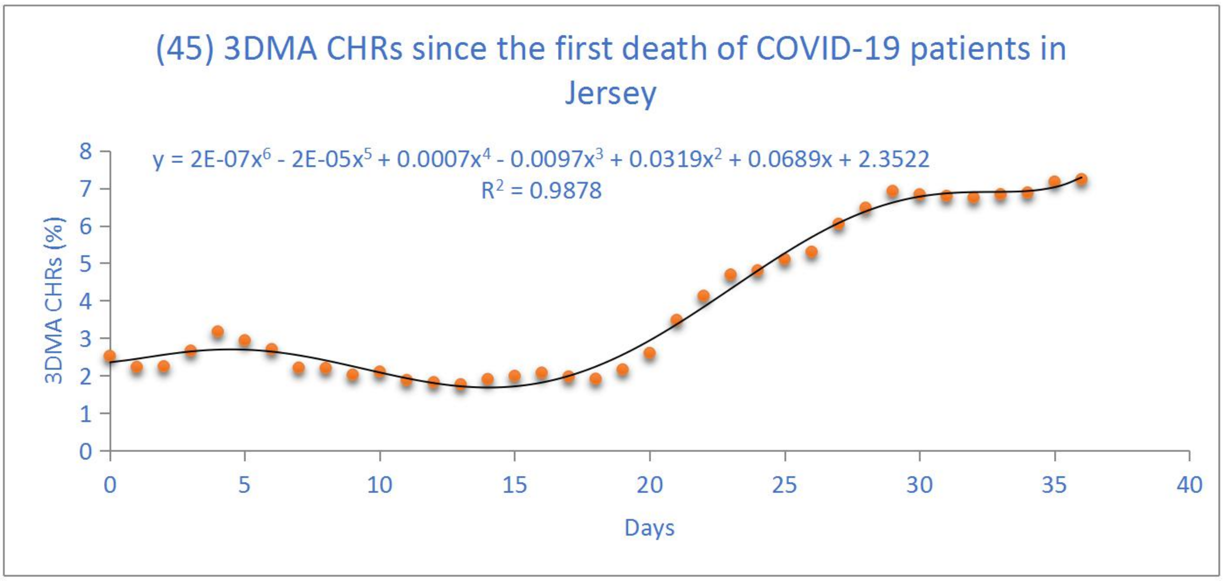
(45) 3DMA CHRs since the first death of COVID-19 patients in Jersey
y = 2E-07x6 - 2E-05x5 + 0.0007x4 - 0.0097x3 + 0.0319x2 + 0.0689x + 2.3522
R2 = 0.9878
3DMA CHRs (%)
Days
8
7
6
5
4
3
2
1
0
0
5
10
15
20
25
30
35
40

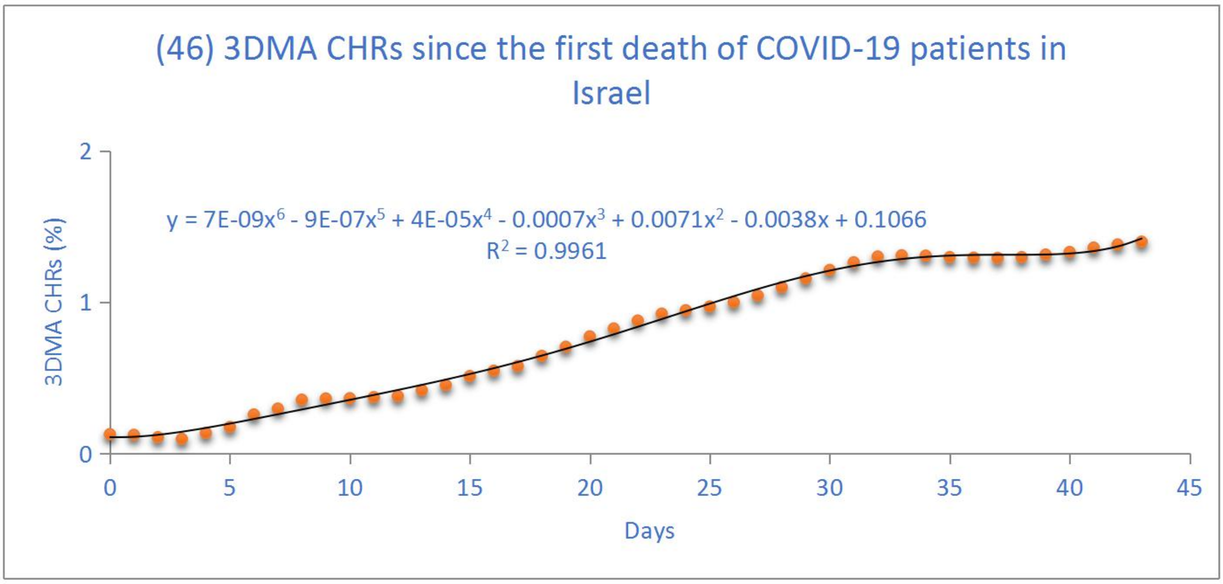
(46) 3DMA CHRs since the first death of COVID-19 patients in Israel
y = 7E-09x6 - 9E-07x5 + 4E-05x4 - 0.0007x3 + 0.0071x2 - 0.0038x + 0.1066
R2 = 0.9961
3DMA CHRs (%)
Days
0
1
2
0
5
10
15
20
25
30
35
40
45

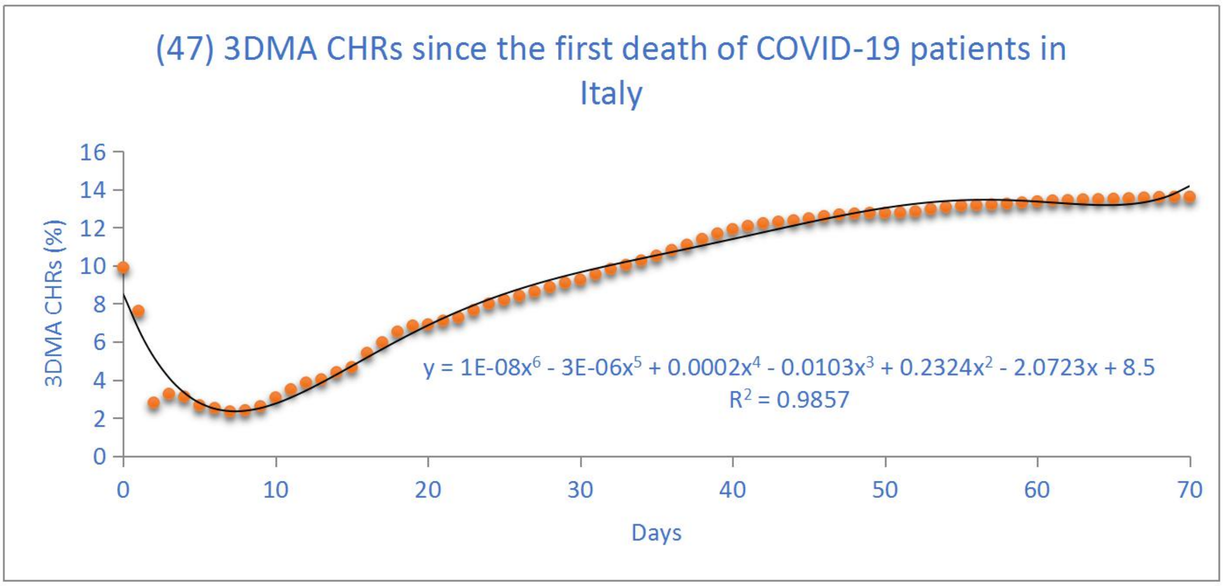
(47) 3DMA CHRs since the first death of COVID-19 patients in Italy
y = 1E-08x6 - 3E-06x5 + 0.0002x4 - 0.0103x3 + 0.2324x2 - 2.0723x + 8.5
R2 = 0.9857
3DMA CHRs (%)
Days
0
2
4
6
8
10
12
14
16
0
10
20
30
40
50
60
70

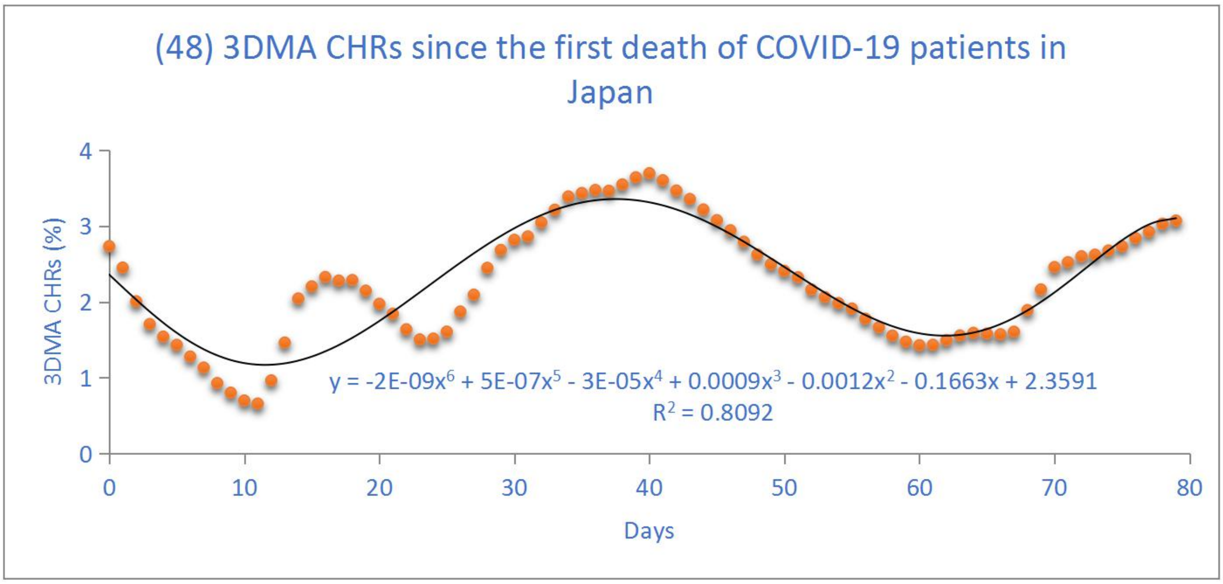
(48) 3DMA CHRs since the first death of COVID-19 patients in Japan
y = -2E-09x6 + 5E-07x5 - 3E-05x4 + 0.0009x3 - 0.0012x2 - 0.1663x + 2.3591
R2 = 0.8092
3DMA CHRs (%)
Days
0
1
2
3
4
0
10
20
30
40
50
60
70
80

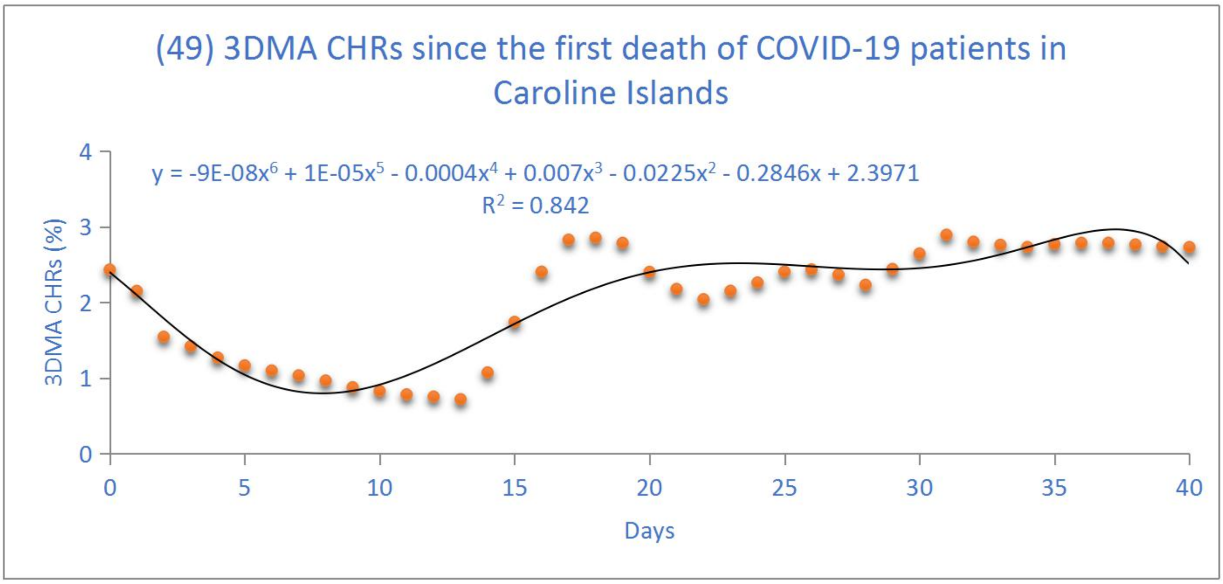
(49) 3DMA CHRs since the first death of COVID-19 patients in Caroline Islands
y = -9E-08x6 + 1E-05x5 - 0.0004x4 + 0.007x3 - 0.0225x2 - 0.2846x + 2.3971
R2 = 0.842
3DMA CHRs (%)
Days
0
1
2
3
4
5
10
15
20
25
30
35
40

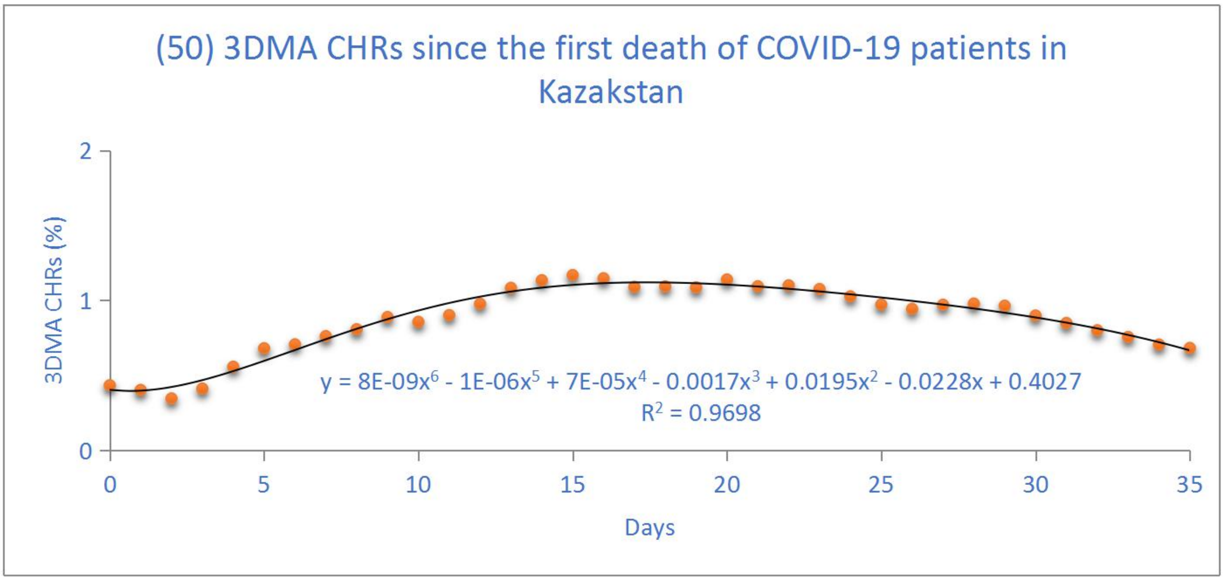
(50) 3DMA CHRs since the first death of COVID-19 patients in Kazakstan
y = 8E-09x6 - 1E-06x5 + 7E-05x4 - 0.0017x3 + 0.0195x2 - 0.0228x + 0.4027
R2 = 0.9698
3DMA CHRs (%)
Days
0
1
2
5
10
15
20
25
30
35

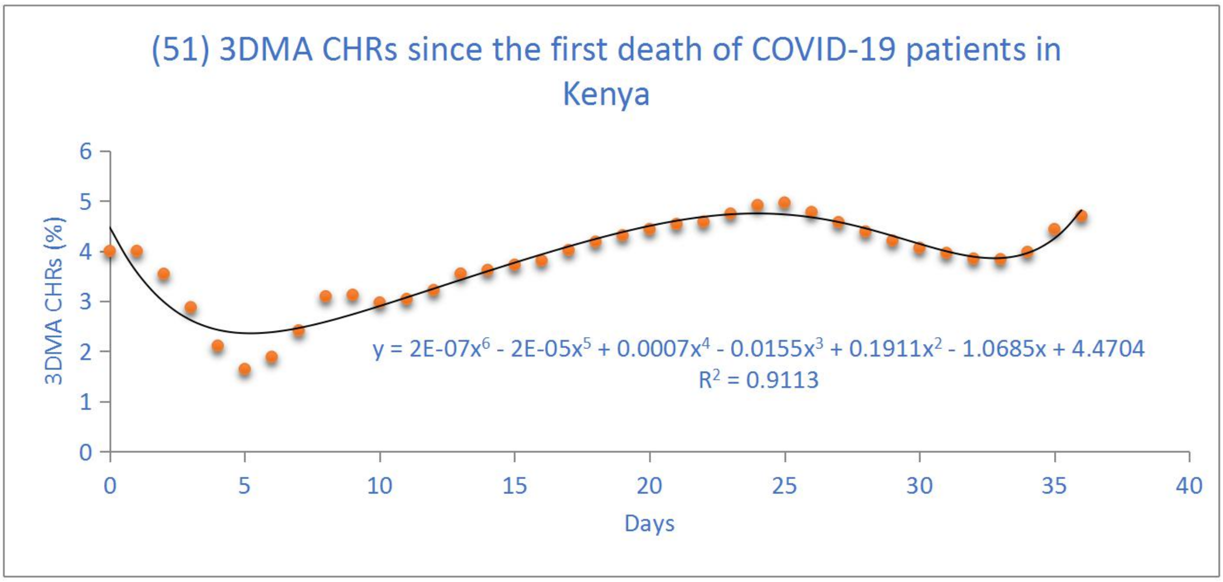
(51) 3DMA CHRs since the first death of COVID-19 patients in Kenya
y = 2E-07x6 - 2E-05x5 + 0.0007x4 - 0.0155x3 + 0.1911x2 - 1.0685x + 4.4704
R2 = 0.9113
3DMA CHRs (%)
Days
0
1
2
3
4
5
6
10
15
20
25
30
35
40

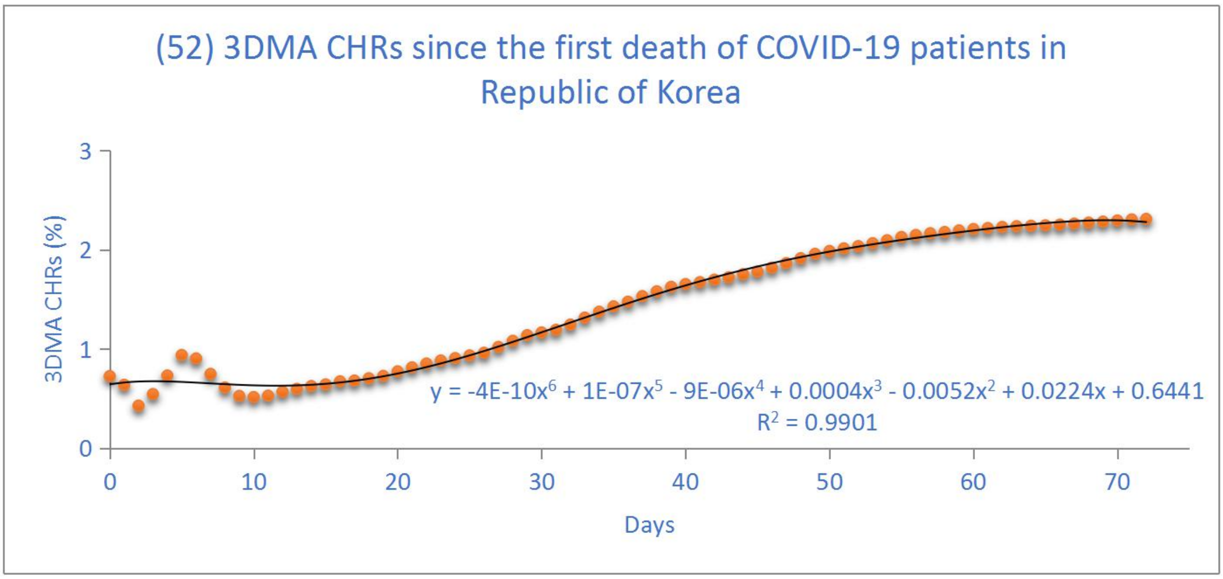
(52) 3DMA CHRs since the first death of COVID-19 patients in Republic of Korea
3DMA CHRs (%)
Days
y = -4E-10x6 + 1E-07x5 - 9E-06x4 + 0.0004x3 - 0.0052x2 + 0.0224x + 0.6441
R2 = 0.9901

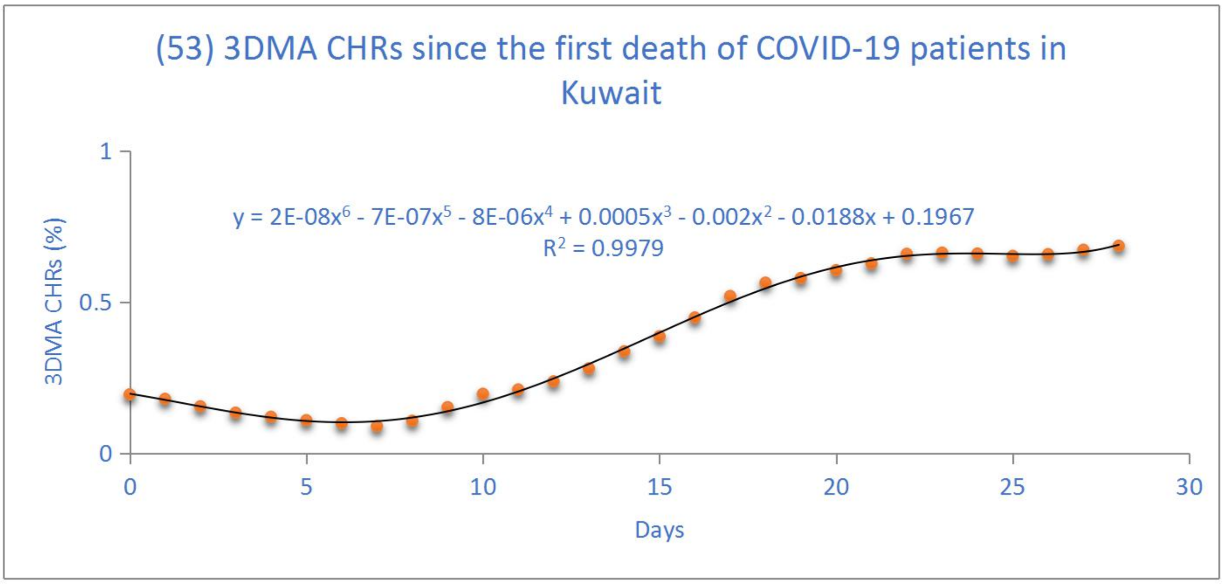
(53) 3DMA CHRs since the first death of COVID-19 patients in Kuwait
3DMA CHRs (%)
Days
y = 2E-08x6 - 7E-07x5 - 8E-06x4 + 0.0005x3 - 0.002x2 - 0.0188x + 0.1967
R2 = 0.9979

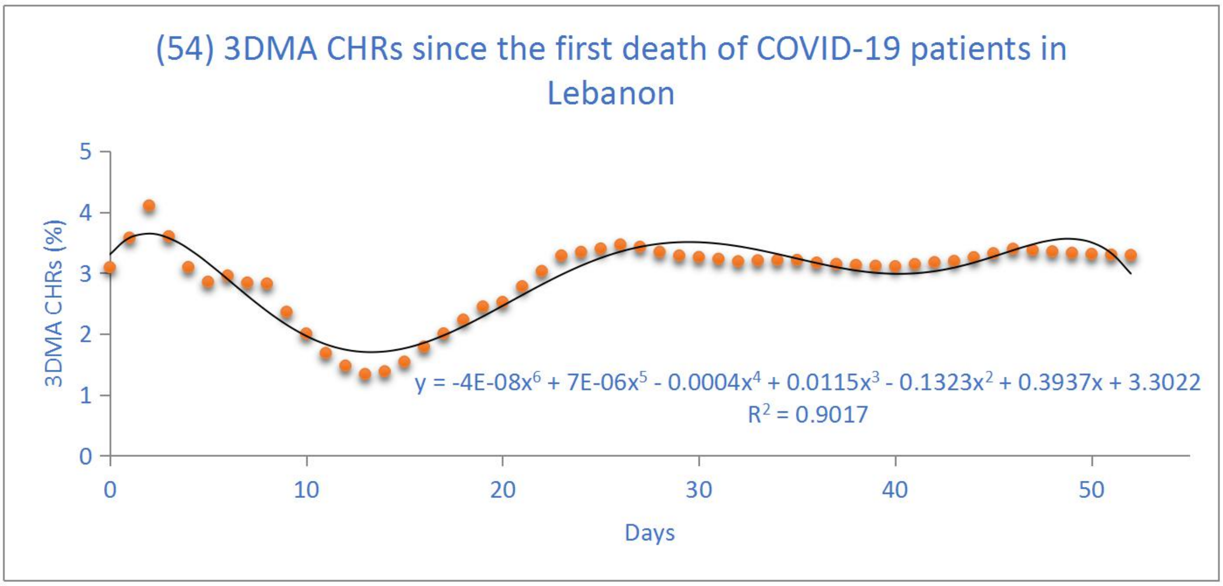
(54) 3DMA CHRs since the first death of COVID-19 patients in Lebanon
3DMA CHRs (%)
Days
y = -4E-08x6 + 7E-06x5 - 0.0004x4 + 0.0115x3 - 0.1323x2 + 0.3937x + 3.3022
R2 = 0.9017

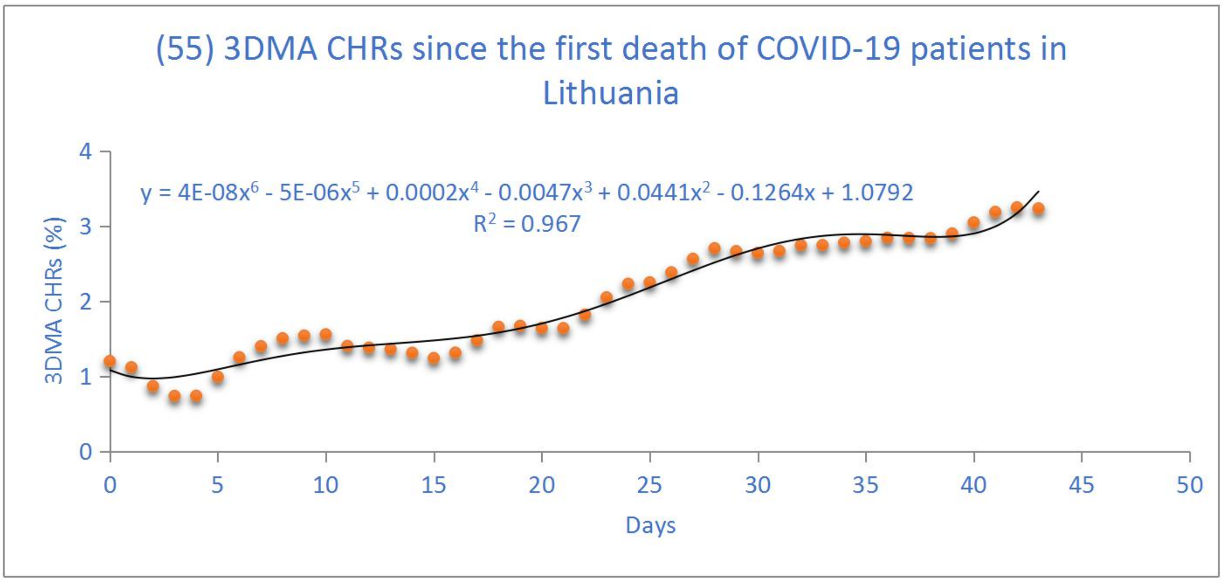
(55) 3DMA CHRs since the first death of COVID-19 patients in Lithuania
y = 4E-08x6 - 5E-06x5 + 0.0002x4 - 0.0047x3 + 0.0441x2 - 0.1264x + 1.0792
R2 = 0.967
3DMA CHRs (%)
Days

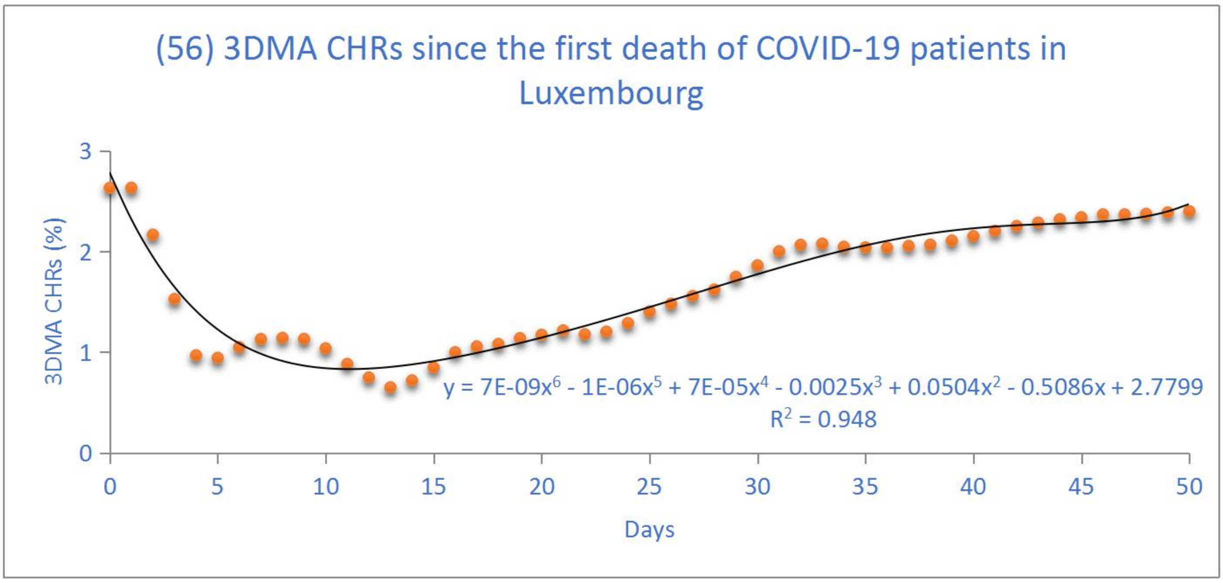
(56) 3DMA CHRs since the first death of COVID-19 patients in Luxembourg
y = 7E-09x6 - 1E-06x5 + 7E-05x4 - 0.0025x3 + 0.0504x2 - 0.5086x + 2.7799
R2 = 0.948
3DMA CHRs (%)
Days

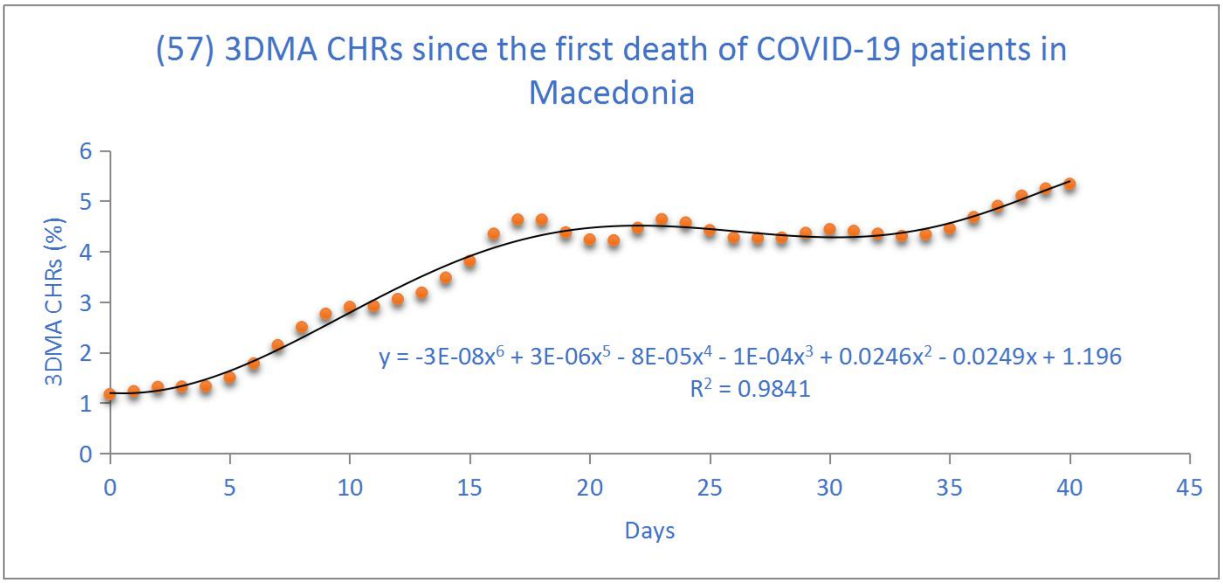
(57) 3DMA CHRs since the first death of COVID-19 patients in Macedonia
y = -3E-08x6 + 3E-06x5 - 8E-05x4 - 1E-04x3 + 0.0246x2 - 0.0249x + 1.196
R2 = 0.9841
3DMA CHRs (%)
Days

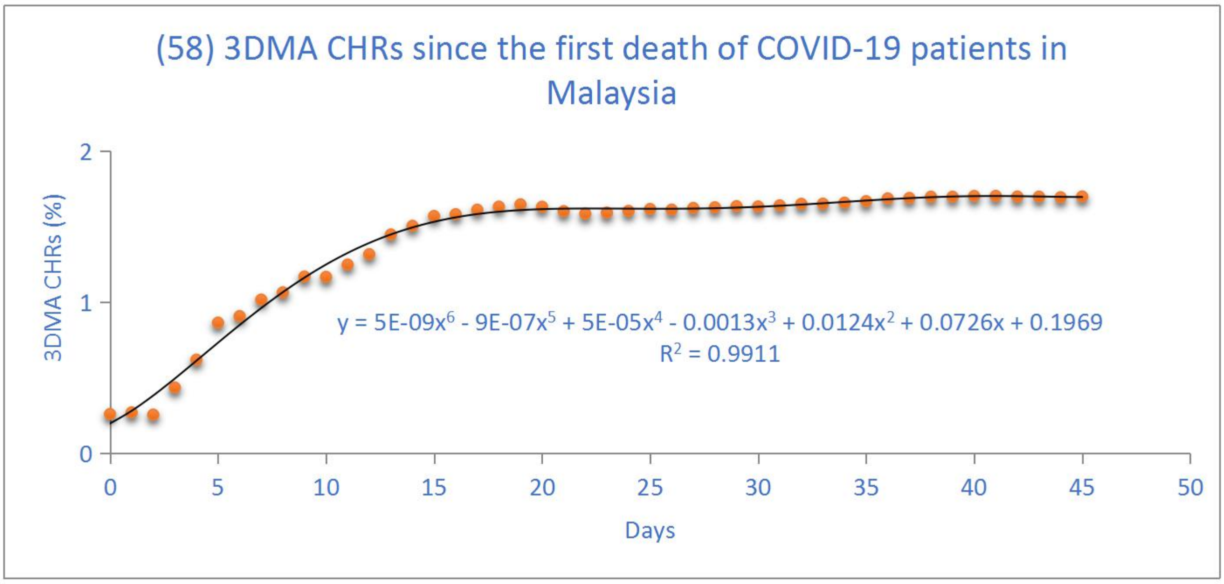

(58) 3DMA CHRs since the first death of COVID-19 patients in Malaysia
3DMA CHRs (%)
Days
y = 5E-09x6 - 9E-07x5 + 5E-05x4 - 0.0013x3 + 0.0124x2 + 0.0726x + 0.1969
R2 = 0.9911

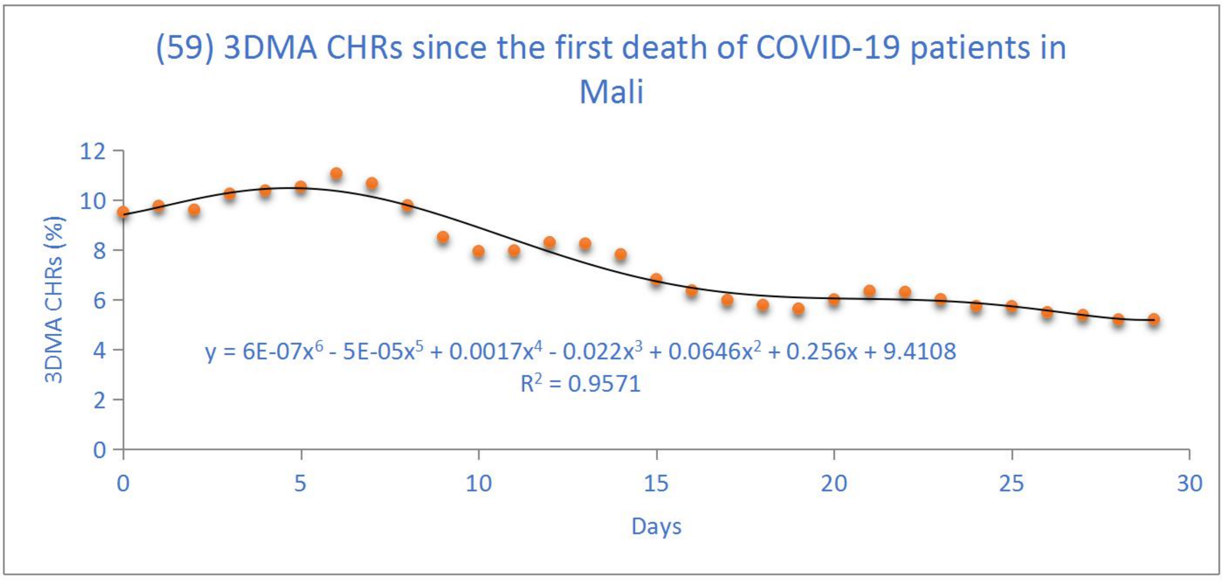

(59) 3DMA CHRs since the first death of COVID-19 patients in Mali
3DMA CHRs (%)
Days
y = 6E-07x6 - 5E-05x5 + 0.0017x4 - 0.022x3 + 0.0646x2 + 0.256x + 9.4108
R2 = 0.9571

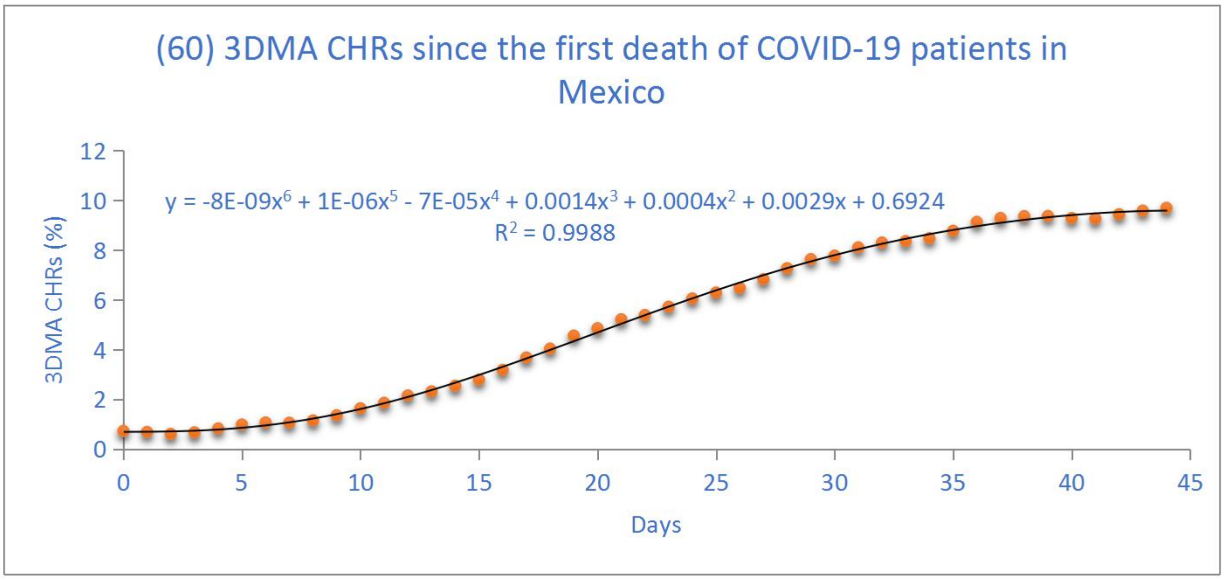

(60) 3DMA CHRs since the first death of COVID-19 patients in Mexico
3DMA CHRs (%)
Days
y = -8E-09x6 + 1E-06x5 - 7E-05x4 + 0.0014x3 + 0.0004x2 + 0.0029x + 0.6924
R2 = 0.9988

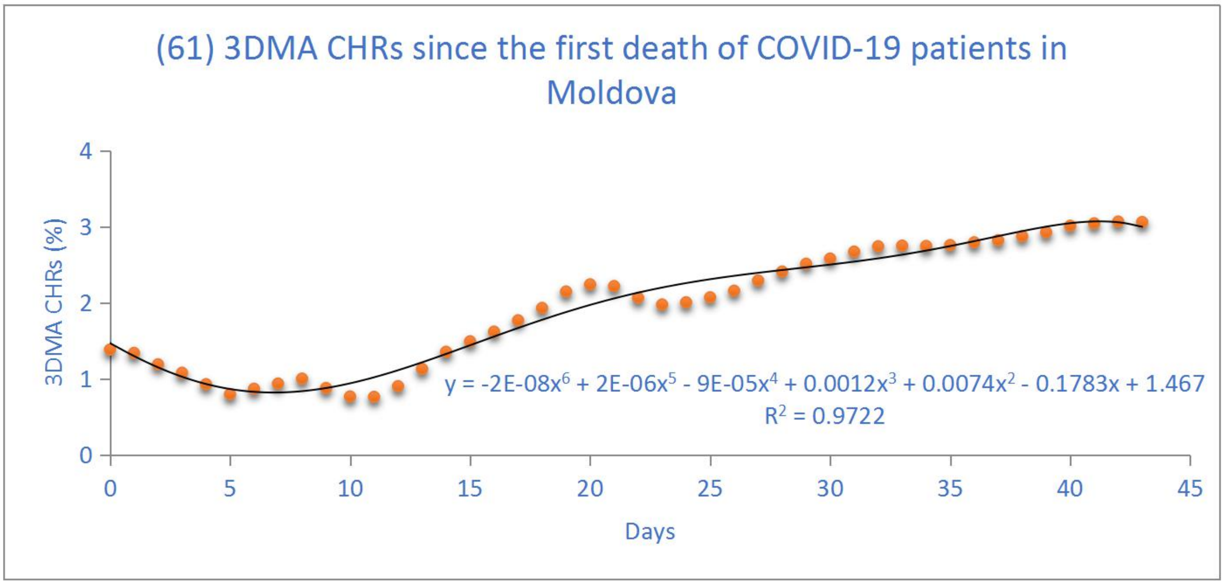
(61) 3DMA CHRs since the first death of COVID-19 patients in Moldova
3DMA CHRs (%)
Days
y = -2E-08x6 + 2E-06x5 - 9E-05x4 + 0.0012x3 + 0.0074x2 - 0.1783x + 1.467
R2 = 0.9722

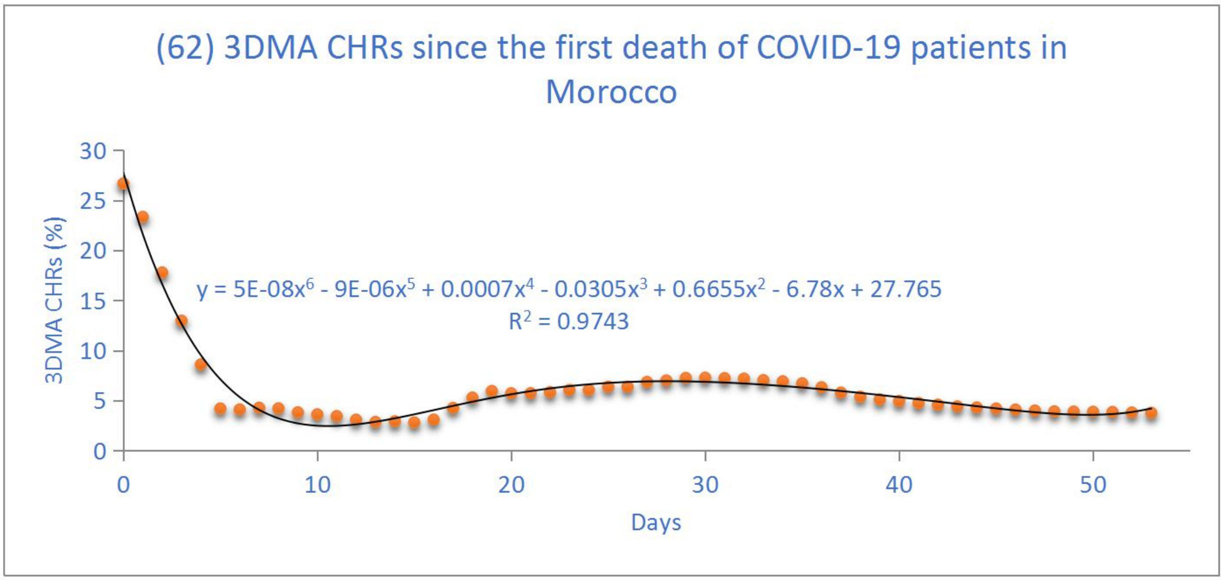
(62) 3DMA CHRs since the first death of COVID-19 patients in Morocco
3DMA CHRs (%)
Days
y = 5E-08x6 - 9E-06x5 + 0.0007x4 - 0.0305x3 + 0.6655x2 - 6.78x + 27.765
R2 = 0.9743

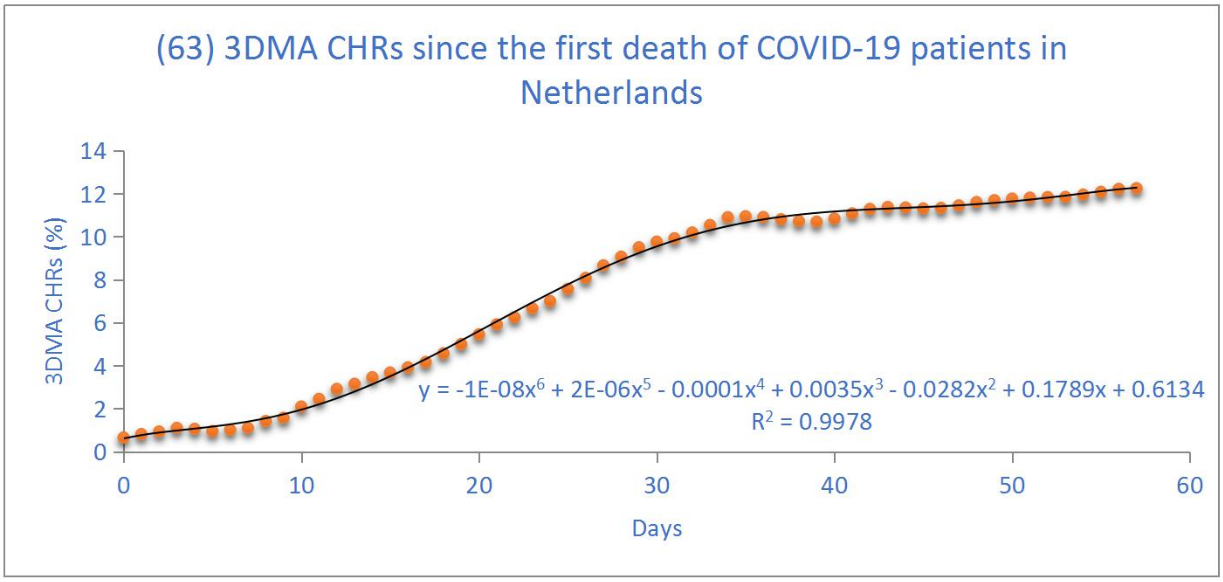
(63) 3DMA CHRs since the first death of COVID-19 patients in Netherlands
3DMA CHRs (%)
Days
y = -1E-08x6 + 2E-06x5 - 0.0001x4 + 0.0035x3 - 0.0282x2 + 0.1789x + 0.6134
R2 = 0.9978

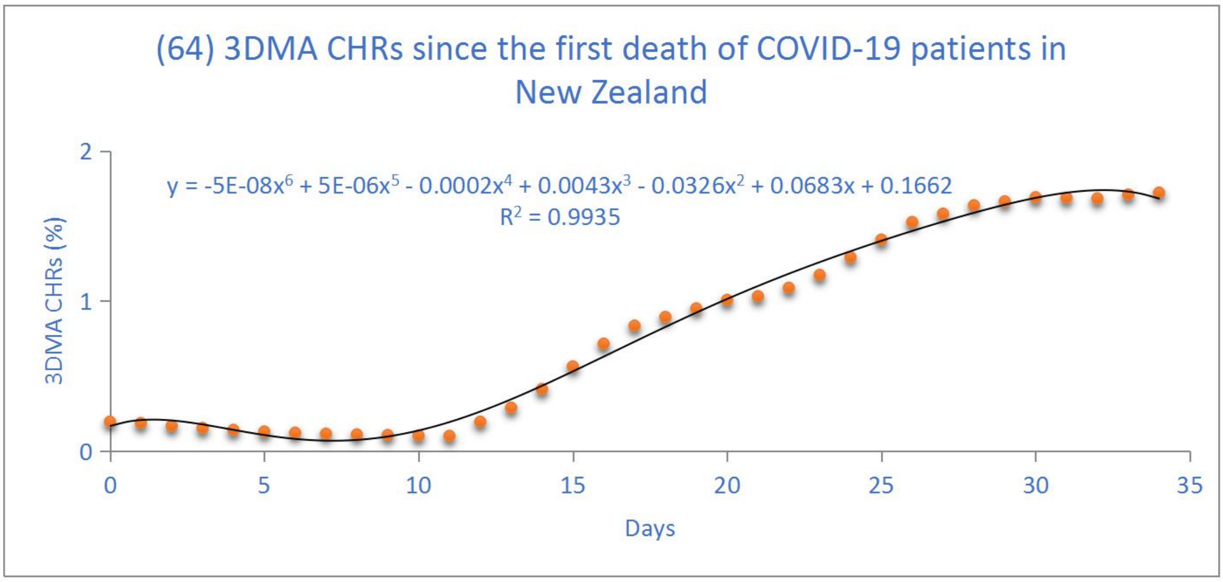

(64) 3DMA CHRs since the first death of COVID-19 patients in New Zealand
y = -5E-08x6 + 5E-06x5 - 0.0002x4 + 0.0043x3 - 0.0326x2 + 0.0683x + 0.1662
R2 = 0.9935
3DMA CHRs (%)
Days

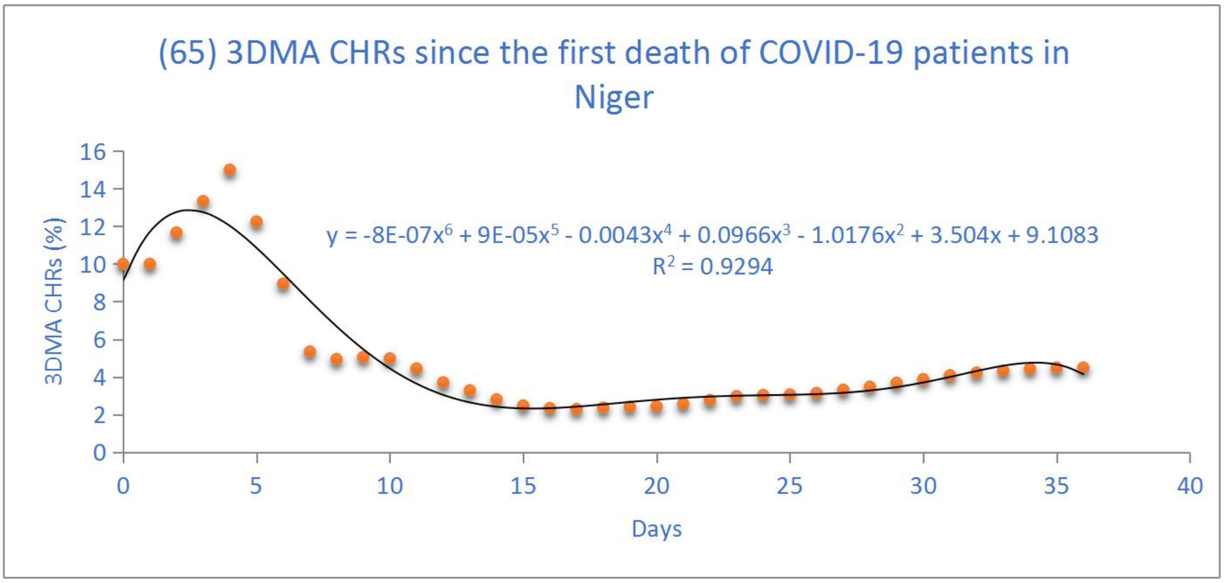

(65) 3DMA CHRs since the first death of COVID-19 patients in Niger
y = -8E-07x6 + 9E-05x5 - 0.0043x4 + 0.0966x3 - 1.0176x2 + 3.504x + 9.1083
R2 = 0.9294
3DMA CHRs (%)
Days

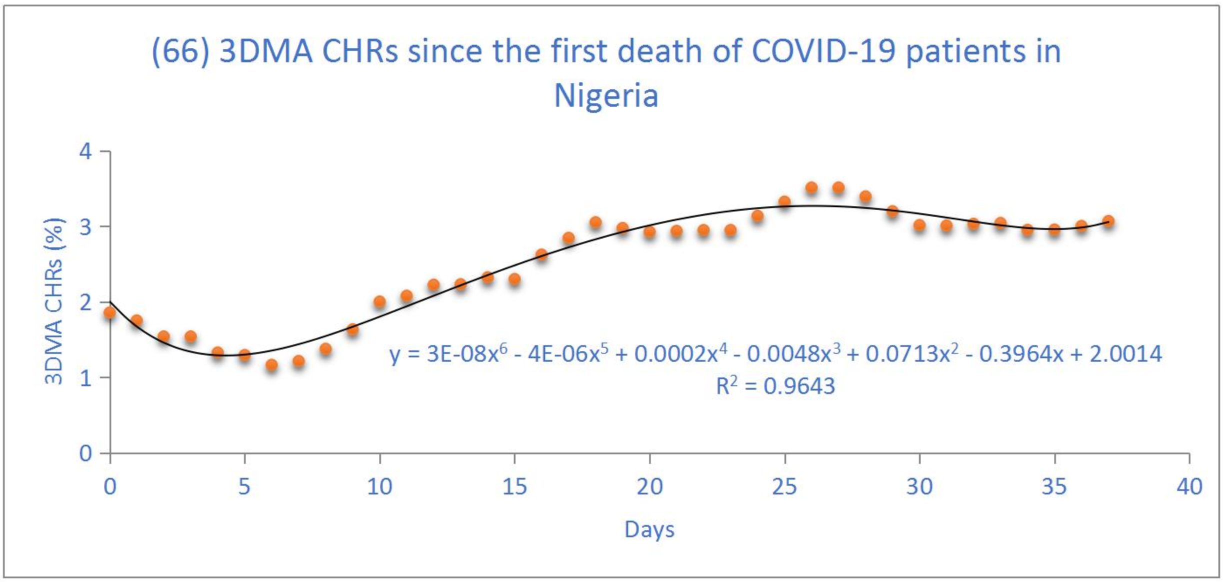

(66) 3DMA CHRs since the first death of COVID-19 patients in Nigeria
y = 3E-08x6 - 4E-06x5 + 0.0002x4 - 0.0048x3 + 0.0713x2 - 0.3964x + 2.0014
R2 = 0.9643
3DMA CHRs (%)
Days

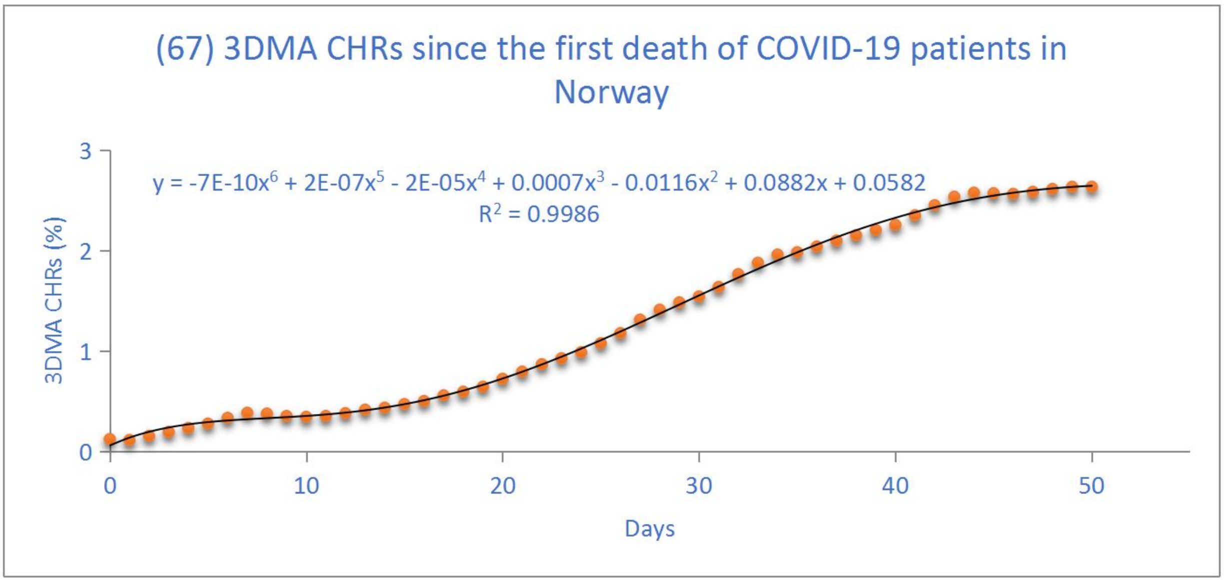
(67) 3DMA CHRs since the first death of COVID-19 patients in Norway
y = -7E-10x6 + 2E-07x5 - 2E-05x4 + 0.0007x3 - 0.0116x2 + 0.0882x + 0.0582
R2 = 0.9986
3DMA CHRs (%)
Days
0
1
2
3
0
10
20
30
40
50

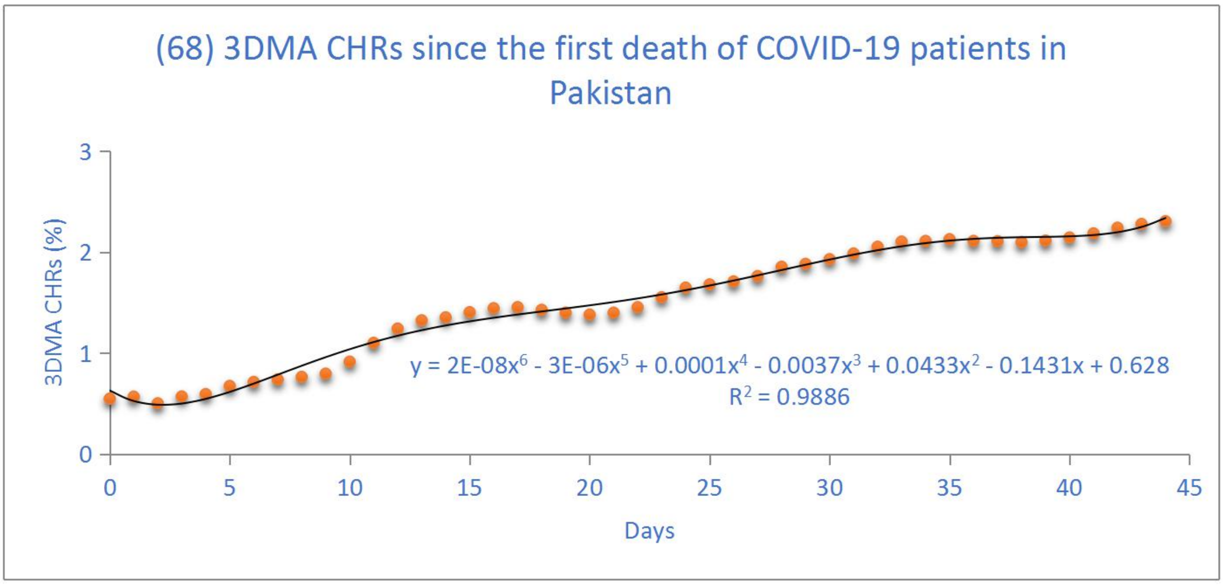
(68) 3DMA CHRs since the first death of COVID-19 patients in Pakistan
y = 2E-08x6 - 3E-06x5 + 0.0001x4 - 0.0037x3 + 0.0433x2 - 0.1431x + 0.628
R2 = 0.9886
3DMA CHRs (%)
Days
0
1
2
3
0
5
10
15
20
25
30
35
40
45

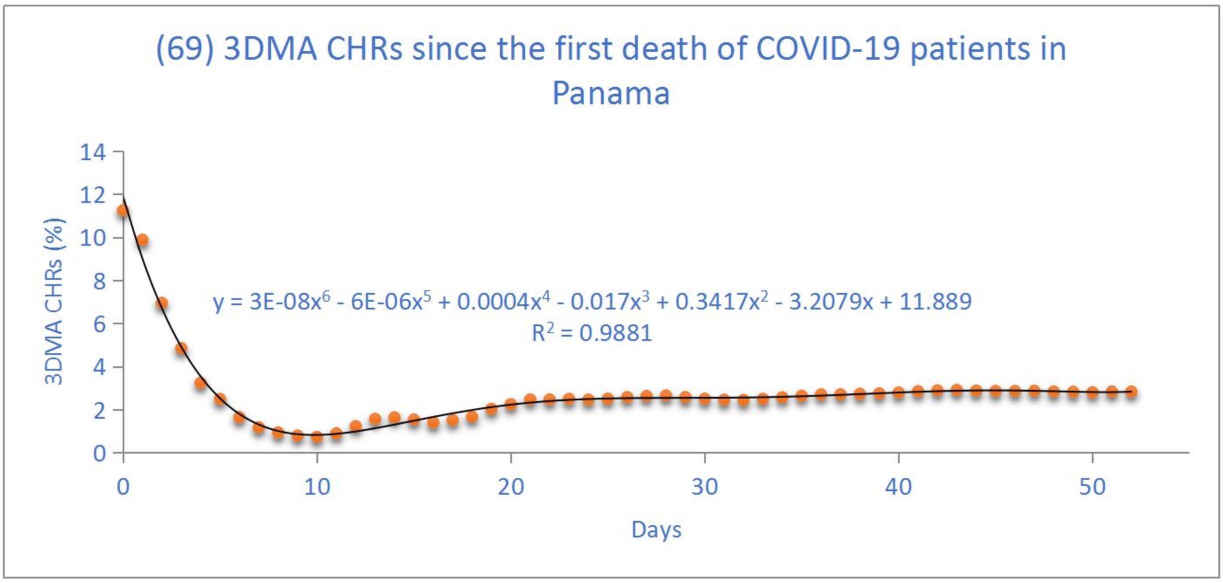
(69) 3DMA CHRs since the first death of COVID-19 patients in Panama
y = 3E-08x6 - 6E-06x5 + 0.0004x4 - 0.017x3 + 0.3417x2 - 3.2079x + 11.889
R2 = 0.9881
3DMA CHRs (%)
Days
0
2
4
6
8
10
12
14
0
10
20
30
40
50

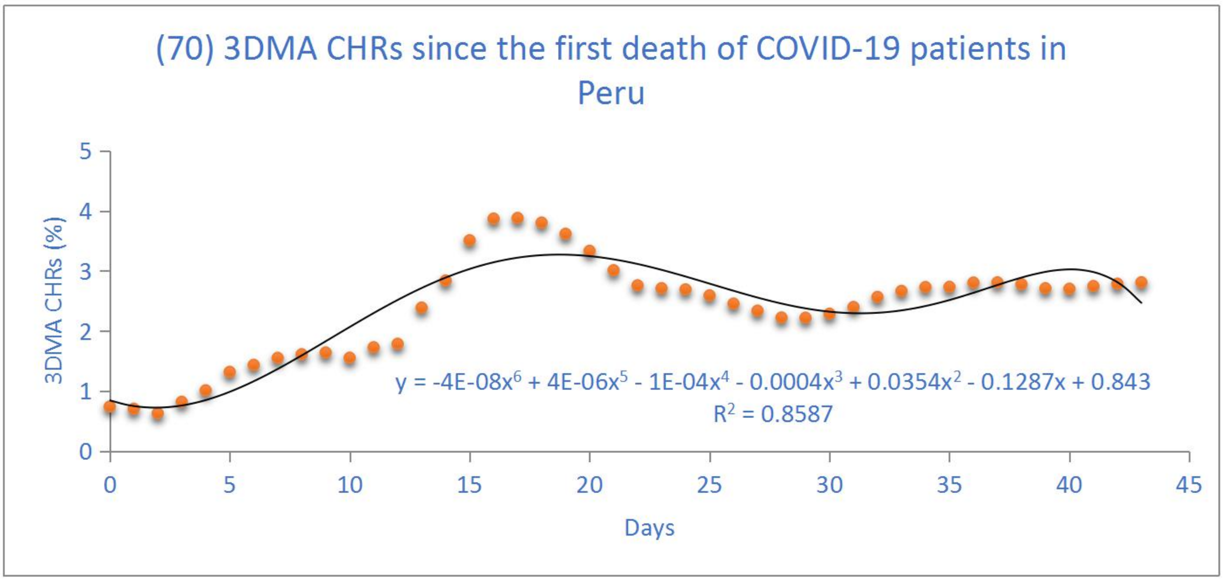
(70) 3DMA CHRs since the first death of COVID-19 patients in Peru
y = -4E-08x6 + 4E-06x5 - 1E-04x4 - 0.0004x3 + 0.0354x2 - 0.1287x + 0.843
R2 = 0.8587
3DMA CHRs (%)
Days
0
1
2
3
4
5
0
5
10
15
20
25
30
35
40
45

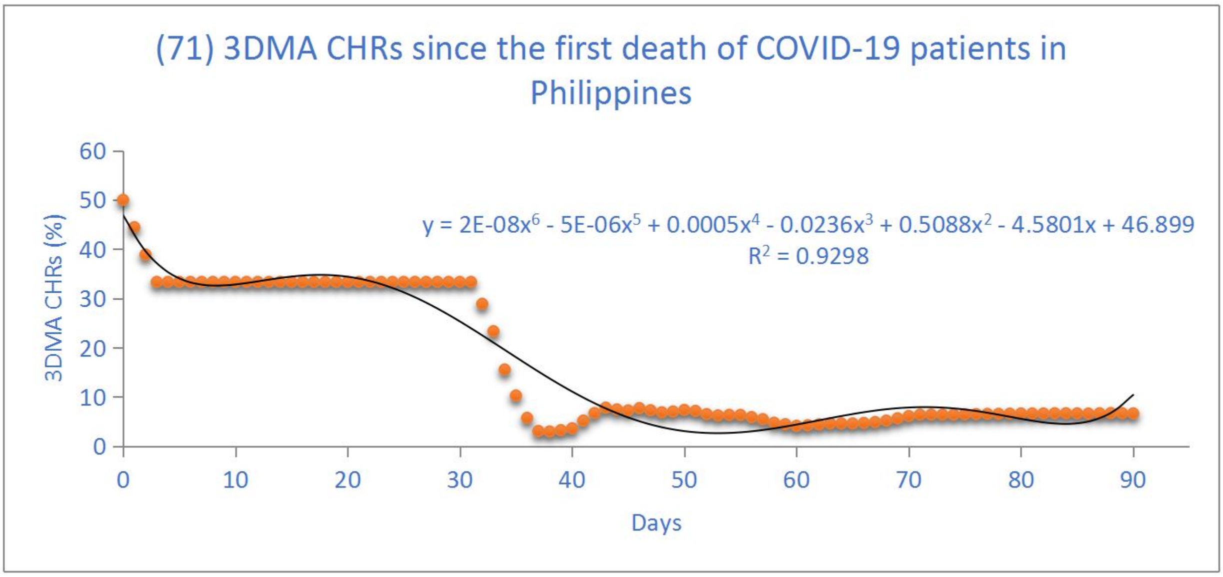
(71) 3DMA CHRs since the first death of COVID-19 patients in Philippines
y = 2E-08x6 - 5E-06x5 + 0.0005x4 - 0.0236x3 + 0.5088x2 - 4.5801x + 46.899
R2 = 0.9298
3DMA CHRs (%)
Days
0
10
20
30
40
50
60
0
10
20
30
40
50
60
70
80
90

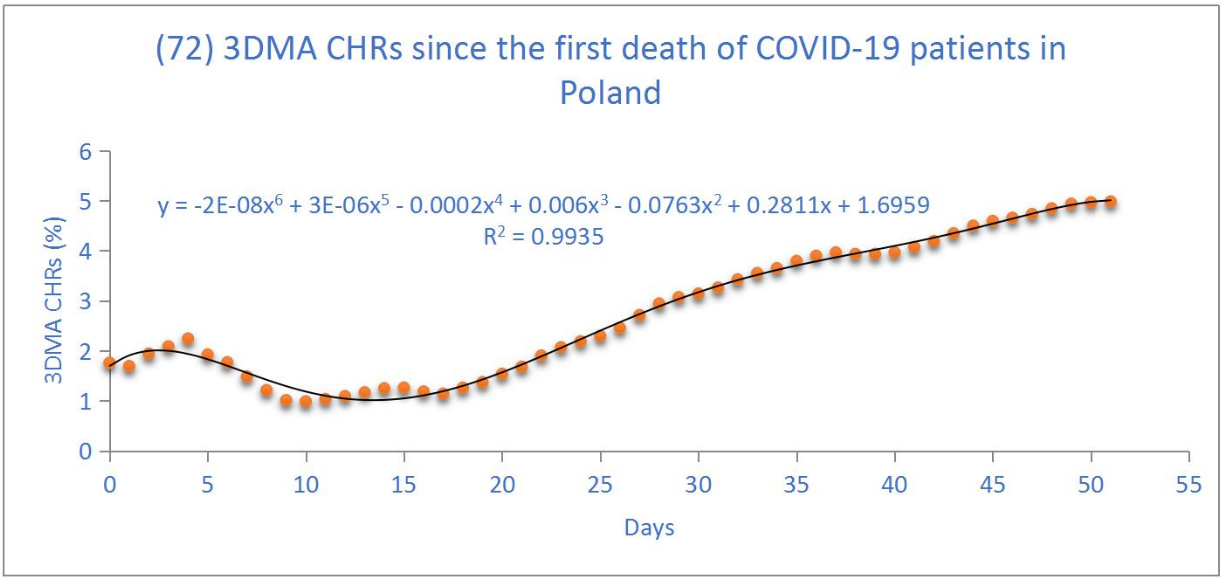
(72) 3DMA CHRs since the first death of COVID-19 patients in Poland
y = -2E-08x6 + 3E-06x5 - 0.0002x4 + 0.006x3 - 0.0763x2 + 0.2811x + 1.6959
R2 = 0.9935
3DMA CHRs (%)
Days
0
1
2
3
4
5
6
0
5
10
15
20
25
30
35
40
45
50
55

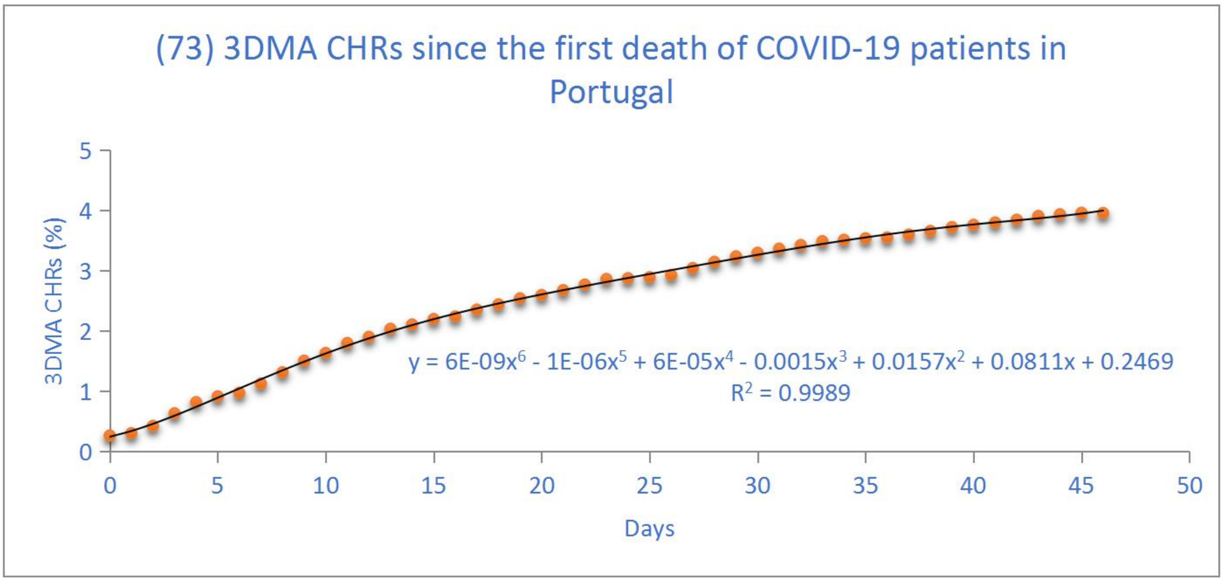
(73) 3DMA CHRs since the first death of COVID-19 patients in Portugal
3DMA CHRs (%)
Days
y = 6E-09x6 - 1E-06x5 + 6E-05x4 - 0.0015x3 + 0.0157x2 + 0.0811x + 0.2469
R2 = 0.9989

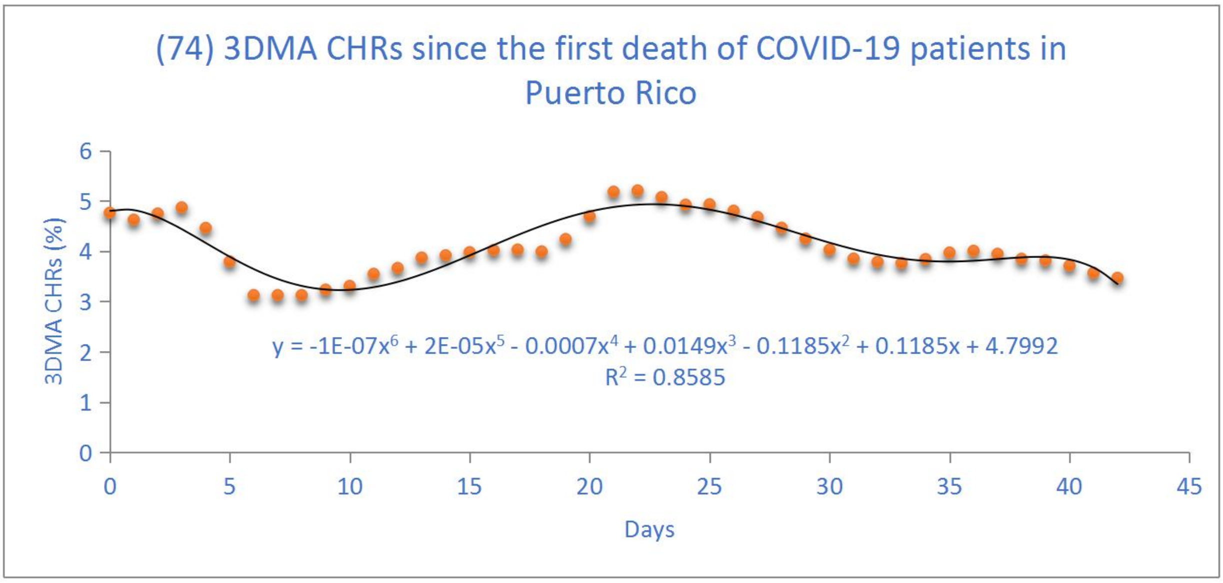
(74) 3DMA CHRs since the first death of COVID-19 patients in Puerto Rico
3DMA CHRs (%)
Days
y = -1E-07x6 + 2E-05x5 - 0.0007x4 + 0.0149x3 - 0.1185x2 + 0.1185x + 4.7992
R2 = 0.8585

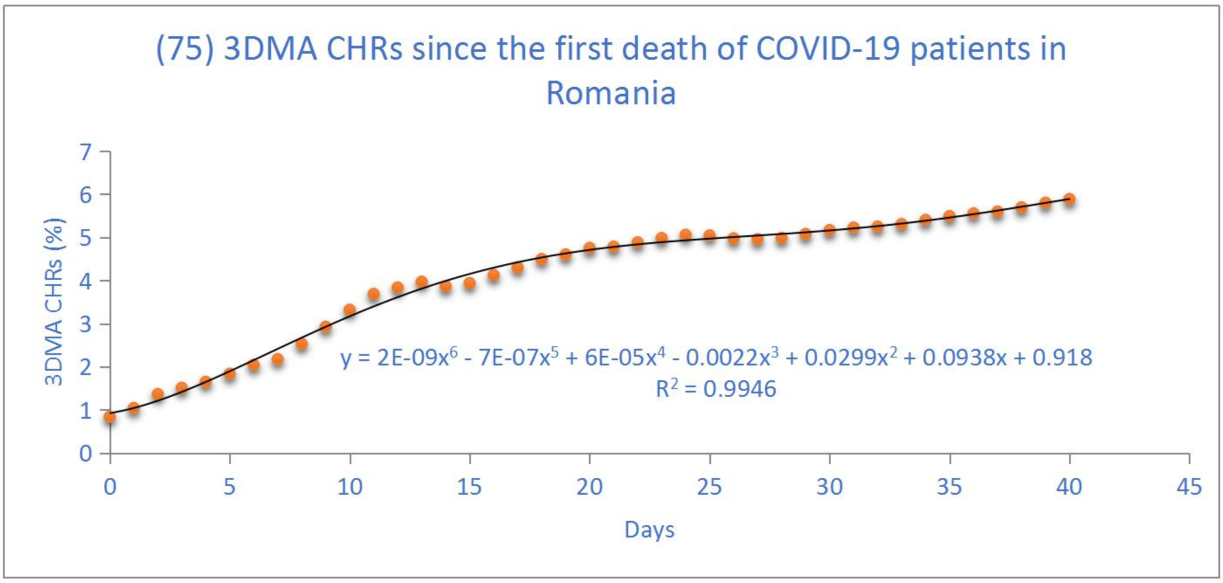
(75) 3DMA CHRs since the first death of COVID-19 patients in Romania
3DMA CHRs (%)
Days
y = 2E-09x6 - 7E-07x5 + 6E-05x4 - 0.0022x3 + 0.0299x2 + 0.0938x + 0.918
R2 = 0.9946

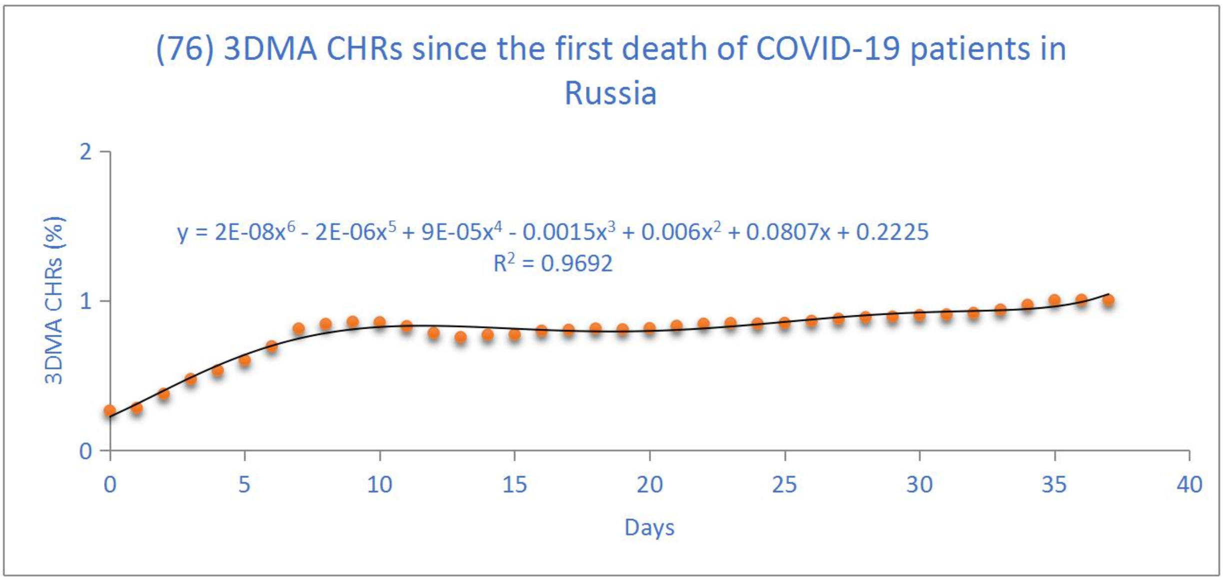
(76) 3DMA CHRs since the first death of COVID-19 patients in Russia
y = 2E-08x6 - 2E-06x5 + 9E-05x4 - 0.0015x3 + 0.006x2 + 0.0807x + 0.2225
R2 = 0.9692
3DMA CHRs (%)
Days

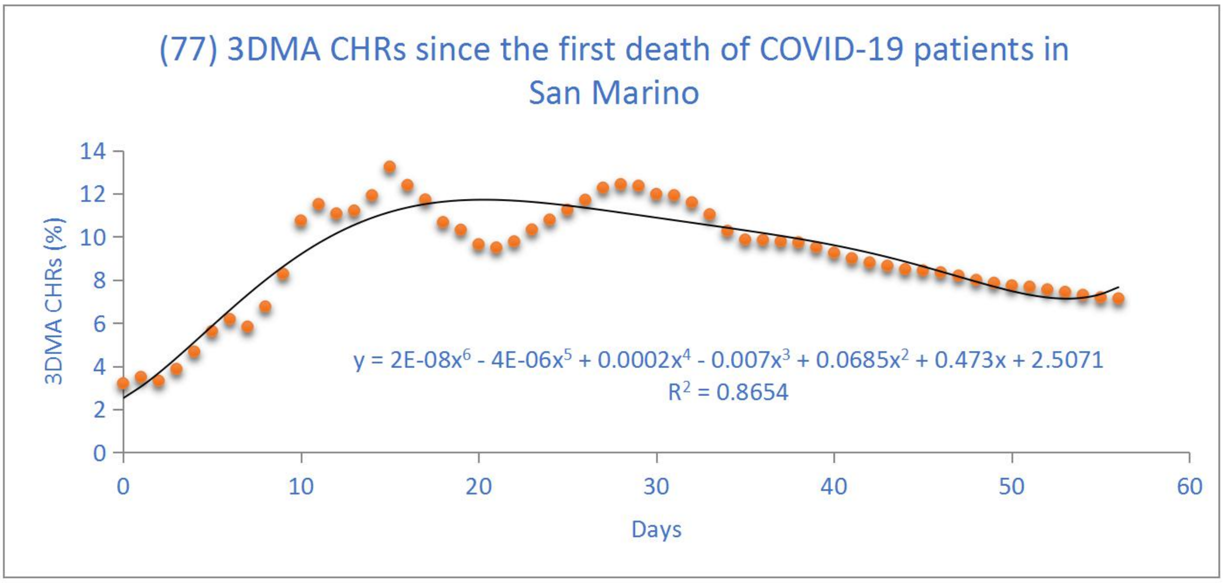
(77) 3DMA CHRs since the first death of COVID-19 patients in San Marino
y = 2E-08x6 - 4E-06x5 + 0.0002x4 - 0.007x3 + 0.0685x2 + 0.473x + 2.5071
R2 = 0.8654
3DMA CHRs (%)
Days

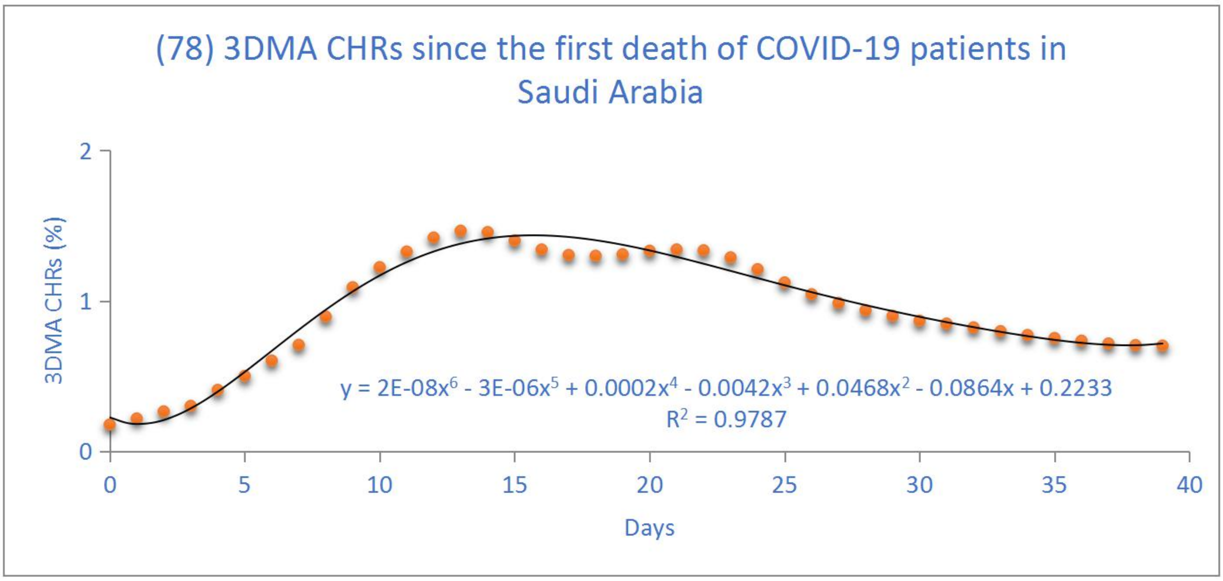
(78) 3DMA CHRs since the first death of COVID-19 patients in Saudi Arabia
y = 2E-08x6 - 3E-06x5 + 0.0002x4 - 0.0042x3 + 0.0468x2 - 0.0864x + 0.2233
R2 = 0.9787
3DMA CHRs (%)
Days

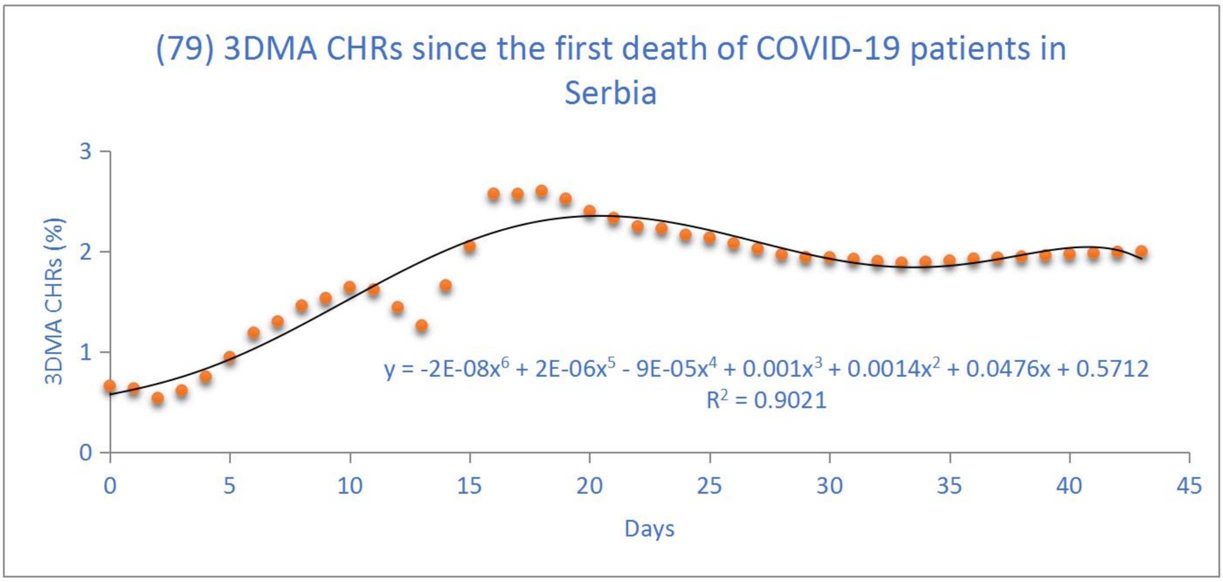
(79) 3DMA CHRs since the first death of COVID-19 patients in Serbia
3DMA CHRs (%)
y = -2E-08x6 + 2E-06x5 - 9E-05x4 + 0.001x3 + 0.0014x2 + 0.0476x + 0.5712
R2 = 0.9021
Days
0
1
2
3
5
10
15
20
25
30
35
40
45

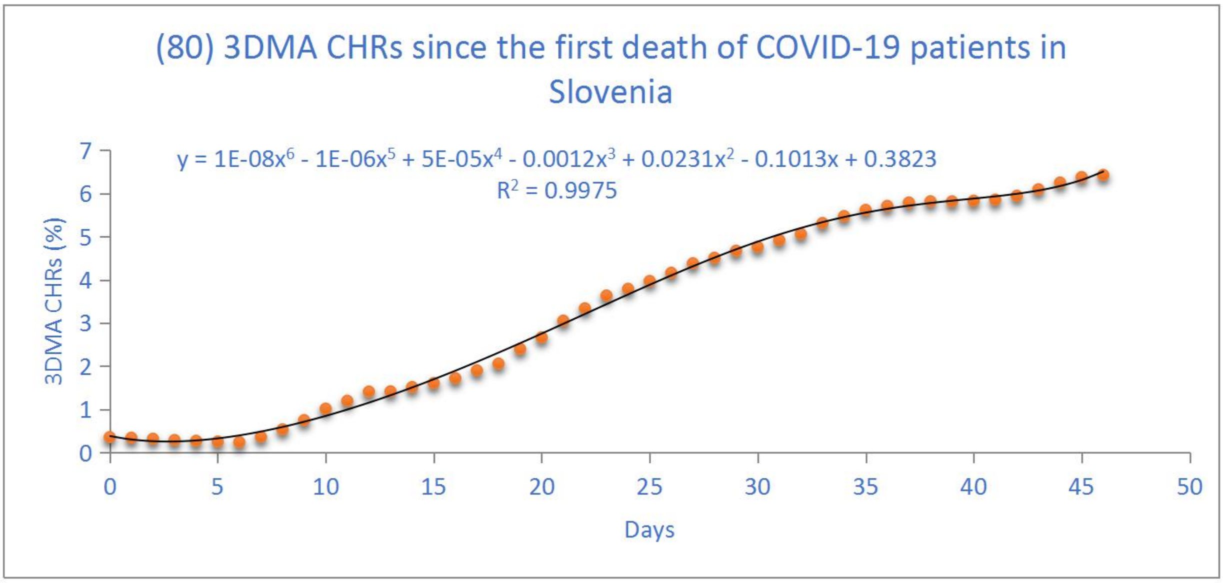
(80) 3DMA CHRs since the first death of COVID-19 patients in Slovenia
3DMA CHRs (%)
y = 1E-08x6 - 1E-06x5 + 5E-05x4 - 0.0012x3 + 0.0231x2 - 0.1013x + 0.3823
R2 = 0.9975
Days
0
1
2
3
4
5
6
7
10
15
20
25
30
35
40
45
50

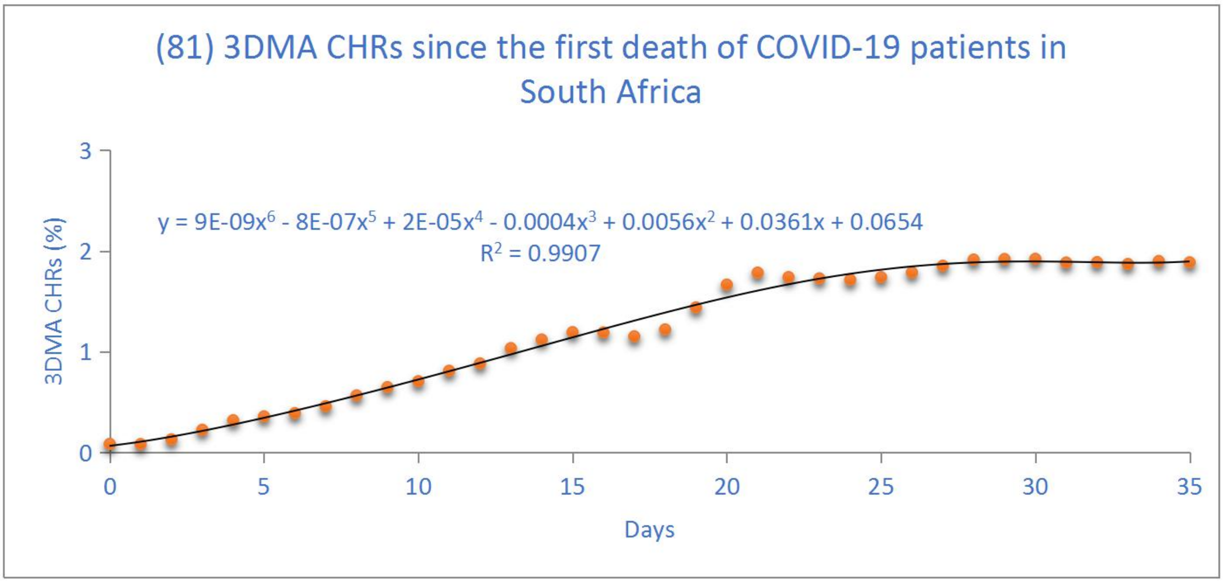
(81) 3DMA CHRs since the first death of COVID-19 patients in South Africa
3DMA CHRs (%)
y = 9E-09x6 - 8E-07x5 + 2E-05x4 - 0.0004x3 + 0.0056x2 + 0.0361x + 0.0654
R2 = 0.9907
Days
0
1
2
3
5
10
15
20
25
30
35

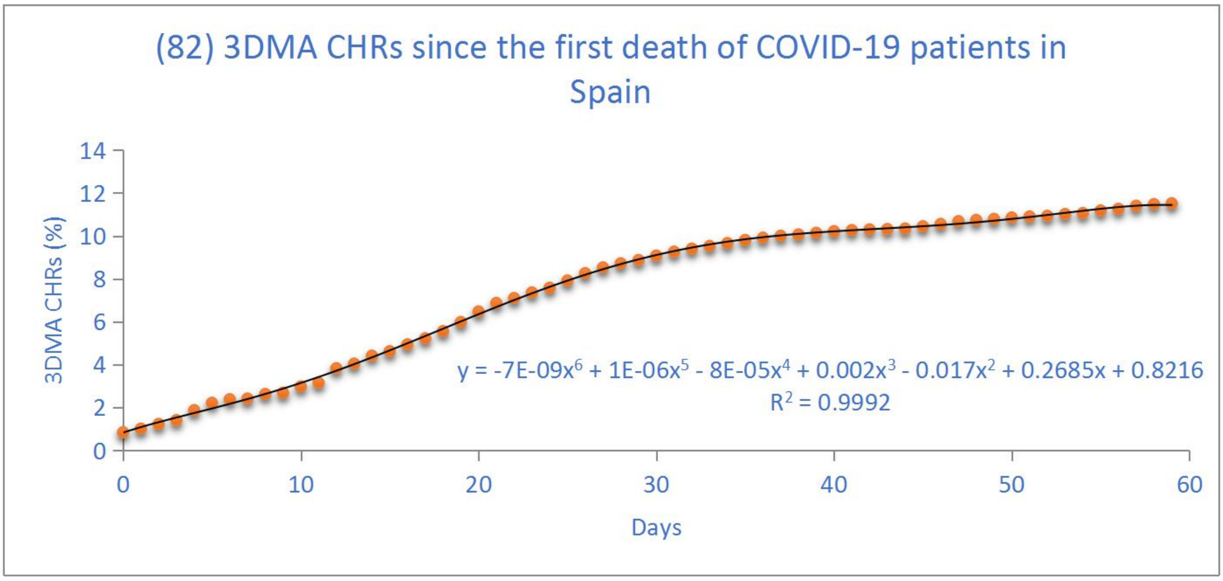
(82) 3DMA CHRs since the first death of COVID-19 patients in Spain
3DMA CHRs (%)
Days
y = -7E-09x6 + 1E-06x5 - 8E-05x4 + 0.002x3 - 0.017x2 + 0.2685x + 0.8216
R2 = 0.9992

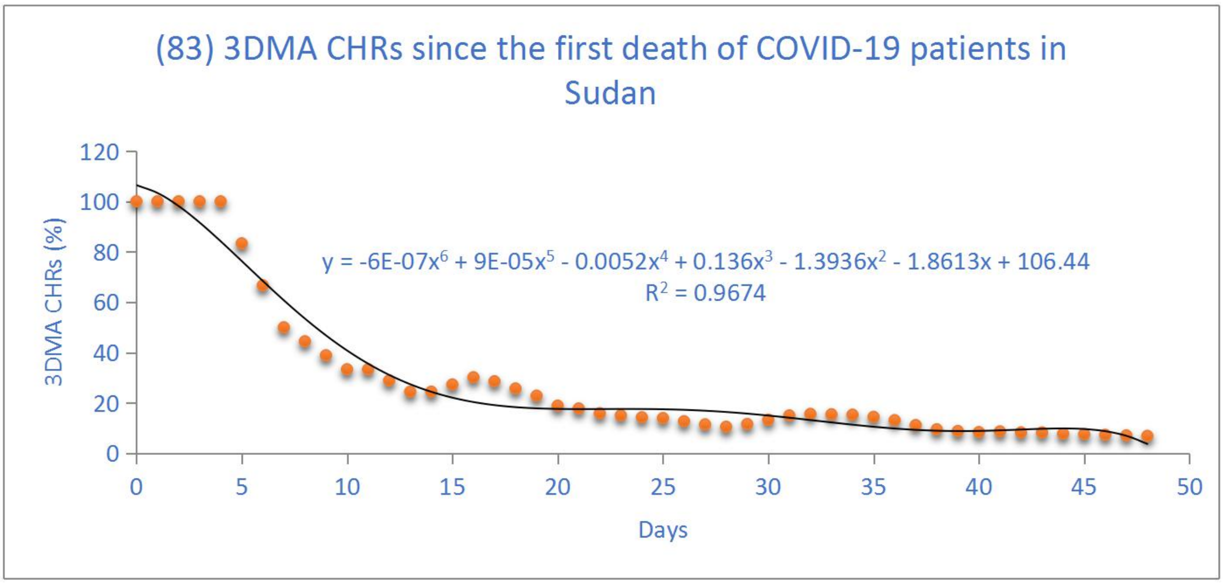
(83) 3DMA CHRs since the first death of COVID-19 patients in Sudan
3DMA CHRs (%)
Days
y = -6E-07x6 + 9E-05x5 - 0.0052x4 + 0.136x3 - 1.3936x2 - 1.8613x + 106.44
R2 = 0.9674

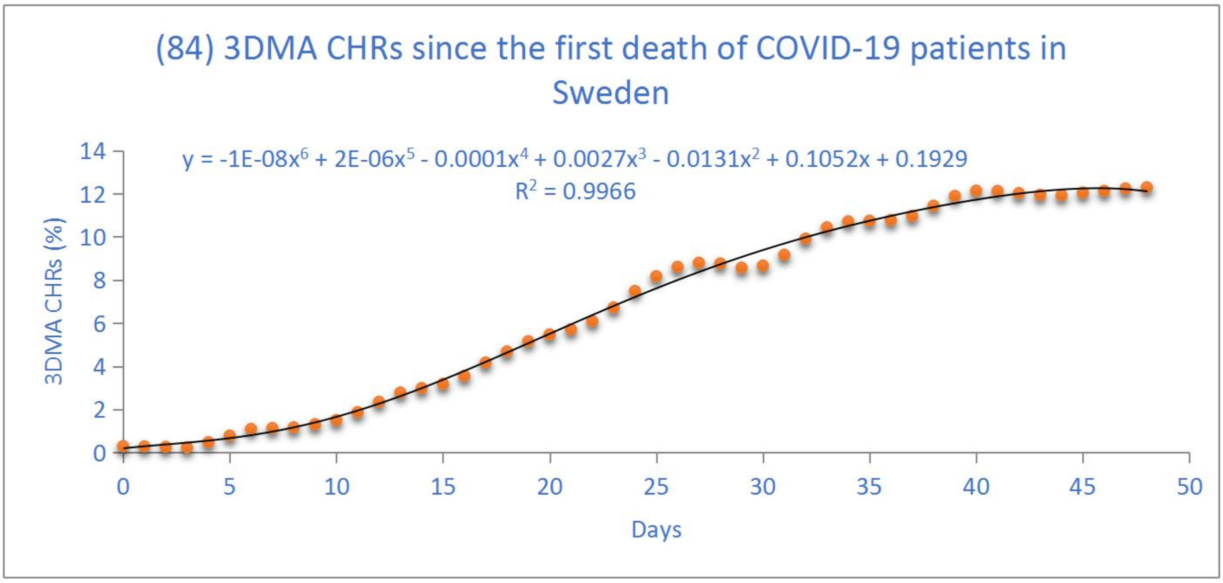
(84) 3DMA CHRs since the first death of COVID-19 patients in Sweden
3DMA CHRs (%)
Days
y = -1E-08x6 + 2E-06x5 - 0.0001x4 + 0.0027x3 - 0.0131x2 + 0.1052x + 0.1929
R2 = 0.9966

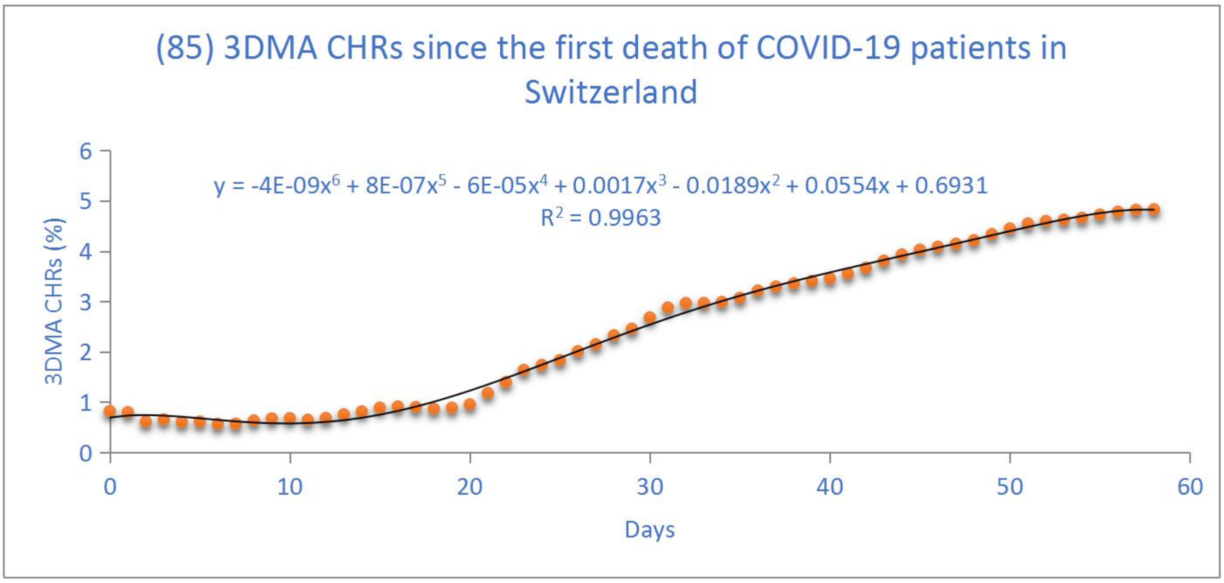
(85) 3DMA CHRs since the first death of COVID-19 patients in Switzerland
y = -4E-09x6 + 8E-07x5 - 6E-05x4 + 0.0017x3 - 0.0189x2 + 0.0554x + 0.6931
R2 = 0.9963
3DMA CHRs (%)
Days

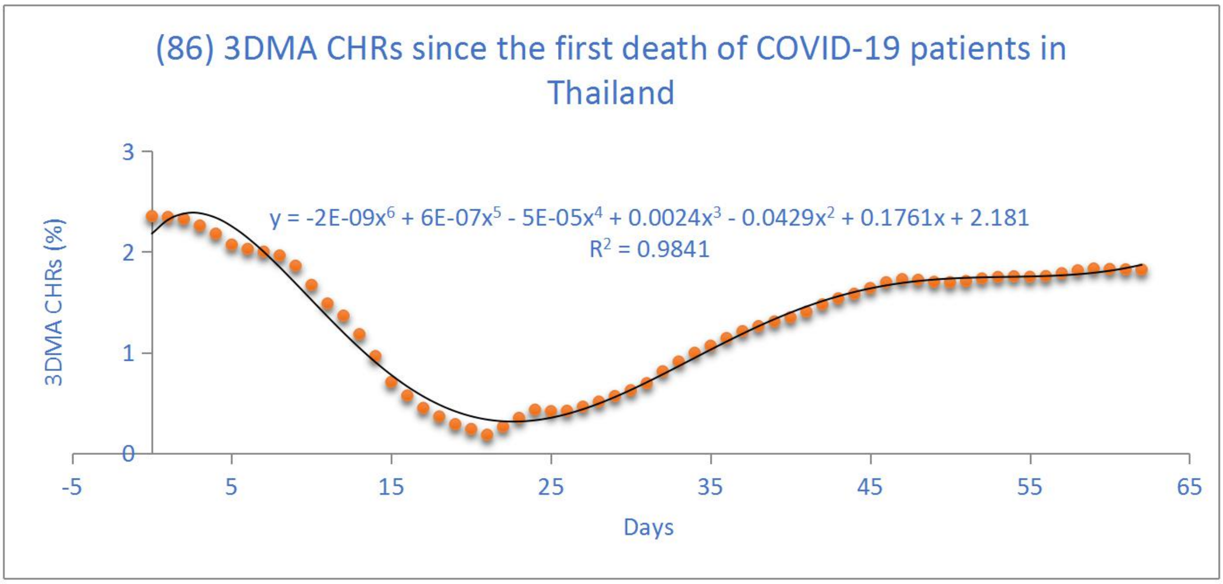
(86) 3DMA CHRs since the first death of COVID-19 patients in Thailand
y = -2E-09x6 + 6E-07x5 - 5E-05x4 + 0.0024x3 - 0.0429x2 + 0.1761x + 2.181
R2 = 0.9841
3DMA CHRs (%)
Days

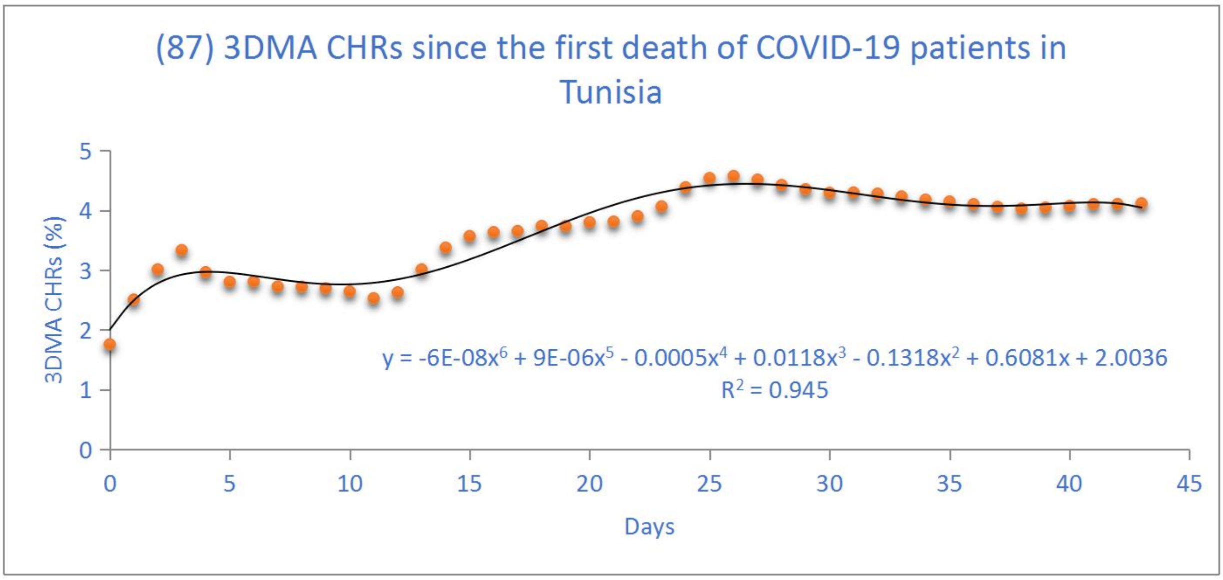
(87) 3DMA CHRs since the first death of COVID-19 patients in Tunisia
y = -6E-08x6 + 9E-06x5 - 0.0005x4 + 0.0118x3 - 0.1318x2 + 0.6081x + 2.0036
R2 = 0.945
3DMA CHRs (%)
Days

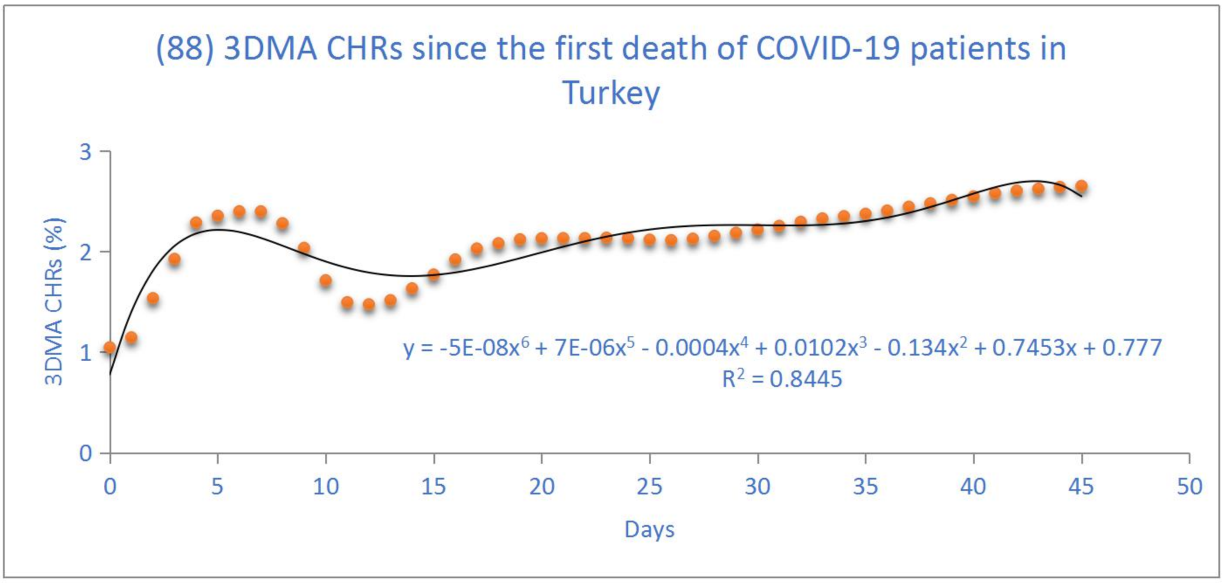

(88) 3DMA CHRs since the first death of COVID-19 patients in Turkey
3DMA CHRs (%)
Days
y = -5E-08x6 + 7E-06x5 - 0.0004x4 + 0.0102x3 - 0.134x2 + 0.7453x + 0.777
R2 = 0.8445

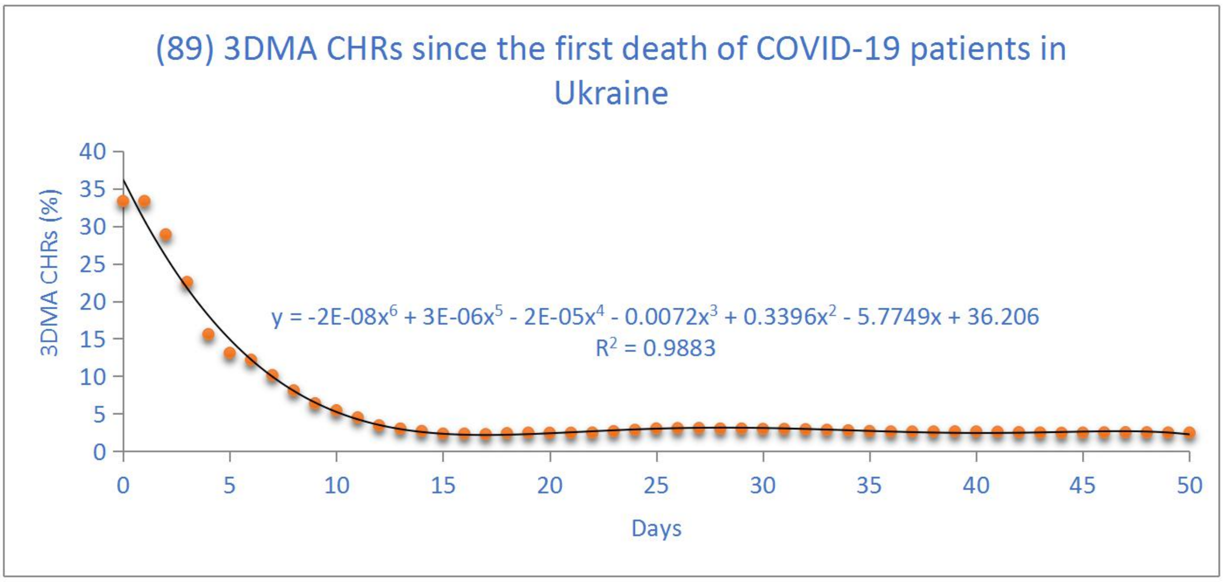

(89) 3DMA CHRs since the first death of COVID-19 patients in Ukraine
3DMA CHRs (%)
Days
y = -2E-08x6 + 3E-06x5 - 2E-05x4 - 0.0072x3 + 0.3396x2 - 5.7749x + 36.206
R2 = 0.9883

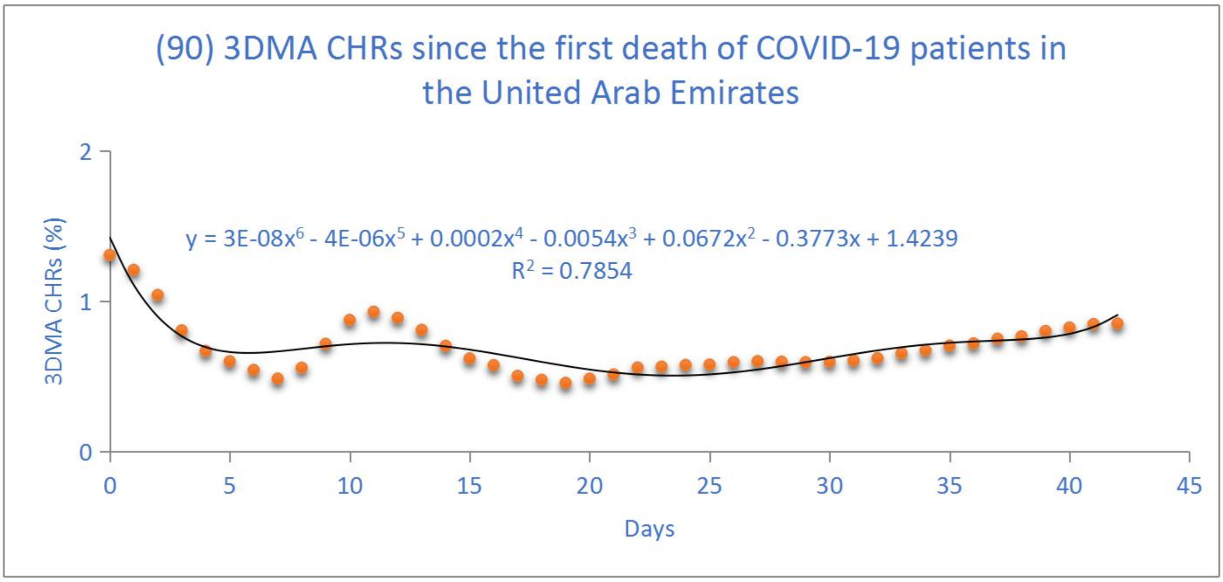

(90) 3DMA CHRs since the first death of COVID-19 patients in the United Arab Emirates
3DMA CHRs (%)
Days
y = 3E-08x6 - 4E-06x5 + 0.0002x4 - 0.0054x3 + 0.0672x2 - 0.3773x + 1.4239
R2 = 0.7854

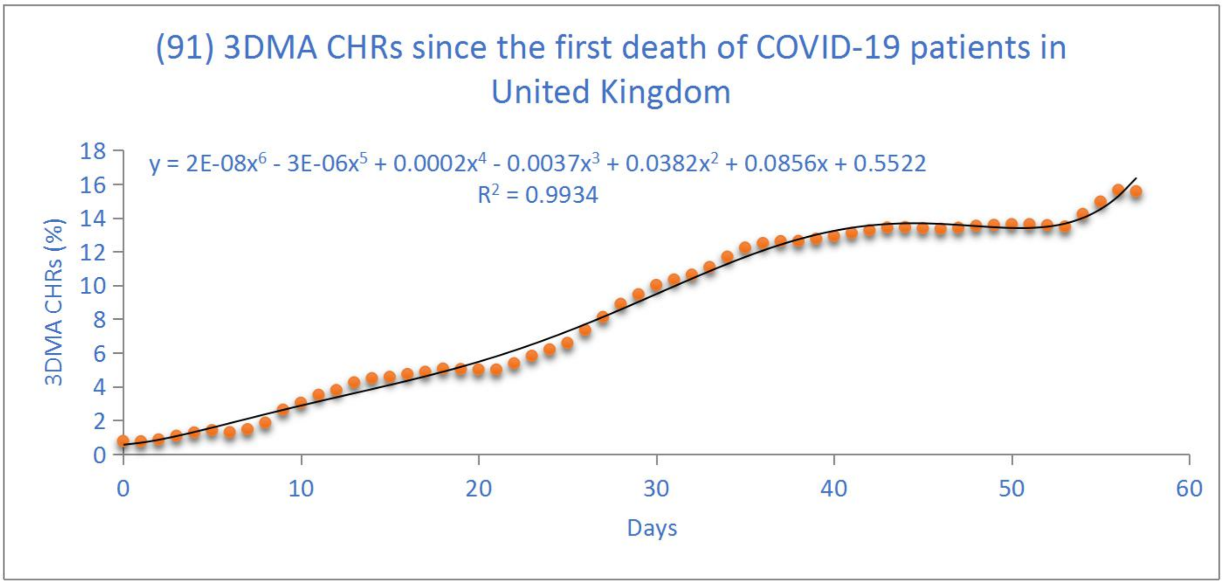
(91) 3DMA CHRs since the first death of COVID-19 patients in United Kingdom
y = 2E-08x6 - 3E-06x5 + 0.0002x4 - 0.0037x3 + 0.0382x2 + 0.0856x + 0.5522
R2 = 0.9934
3DMA CHRs (%)
Days
0
2
4
6
8
10
12
14
16
18
0
10
20
30
40
50
60

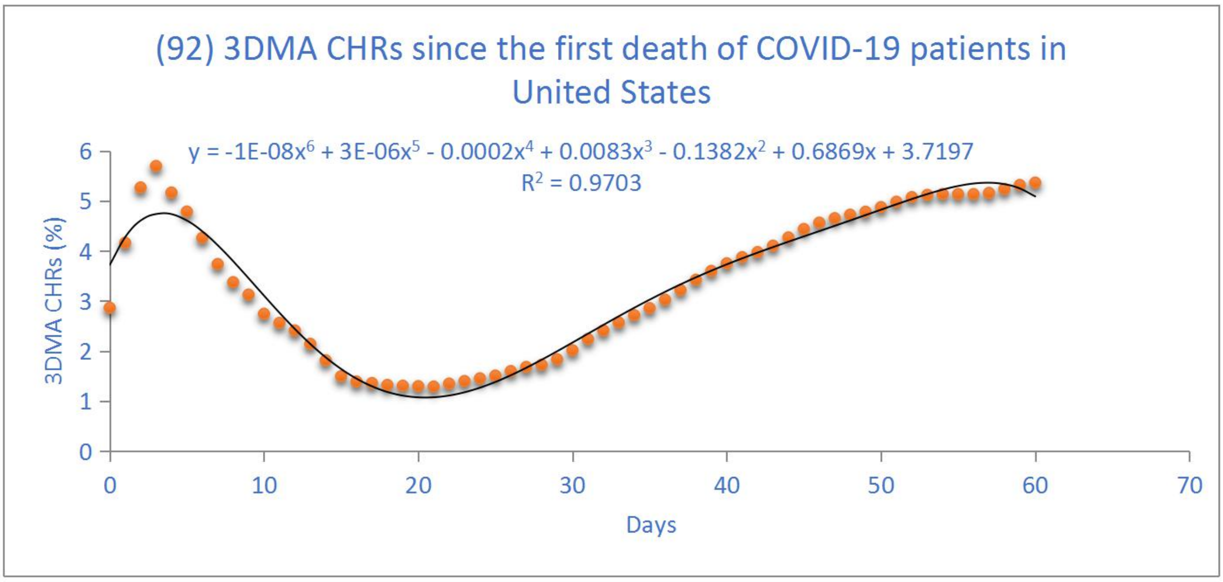
(92) 3DMA CHRs since the first death of COVID-19 patients in United States
y = -1E-08x6 + 3E-06x5 - 0.0002x4 + 0.0083x3 - 0.1382x2 + 0.6869x + 3.7197
R2 = 0.9703
3DMA CHRs (%)
Days
0
1
2
3
4
5
6
0
10
20
30
40
50
60
70
