# Supplementary material for: Predictive Model for National Minimal CFR during Spontaneous Initial Outbreak of Emerging Infectious Disease: Lessons from COVID-19 Pandemic in 214 Nations and Regions
Source: Int J Environ Res Public Health. 2022 Dec 29;20(1):594. doi: 10.3390/ijerph20010594 (PMC9819427; doi:10.3390/ijerph20010594)
Supplement: Supplementary file 1 [file ijerph-20-00594-s001.zip › Figure S3 3DMA CHRs in 17 nations.pdf]

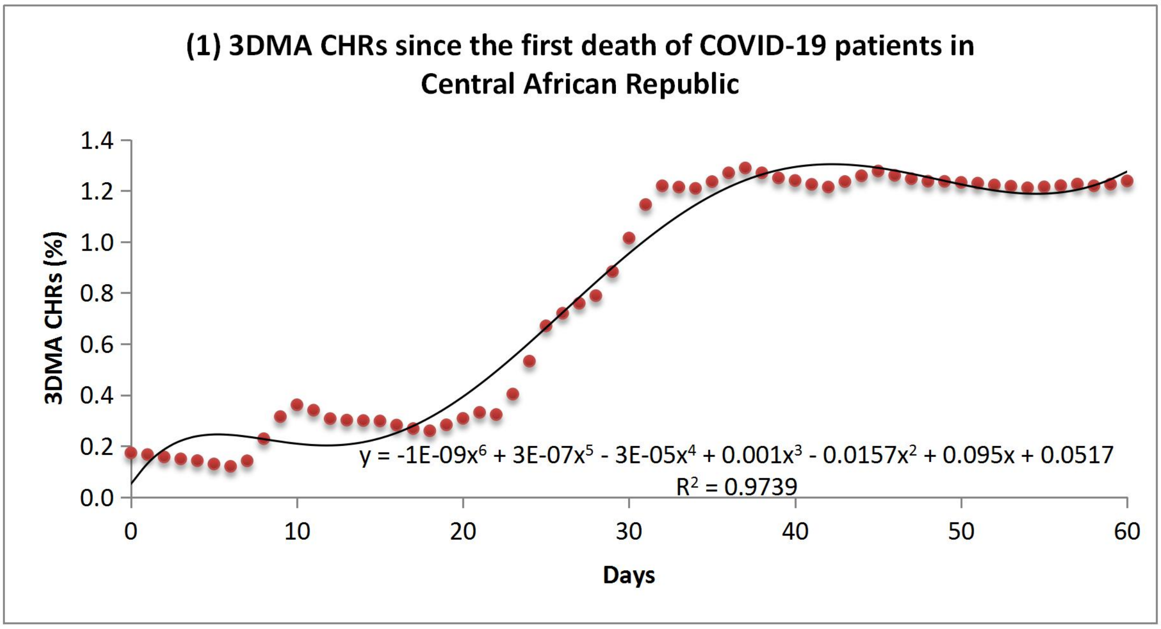
(1) 3DMA CHRs since the first death of COVID-19 patients in Central African Republic
3DMA CHRs (%)
1.4
1.2
1.0
0.8
0.6
0.4
0.2
0.0
y = -1E-09x⁶ + 3E-07x⁵ - 3E-05x⁴ + 0.001x³ - 0.0157x² + 0.095x + 0.0517
R² = 0.9739
0
10
20
30
40
50
60
Days

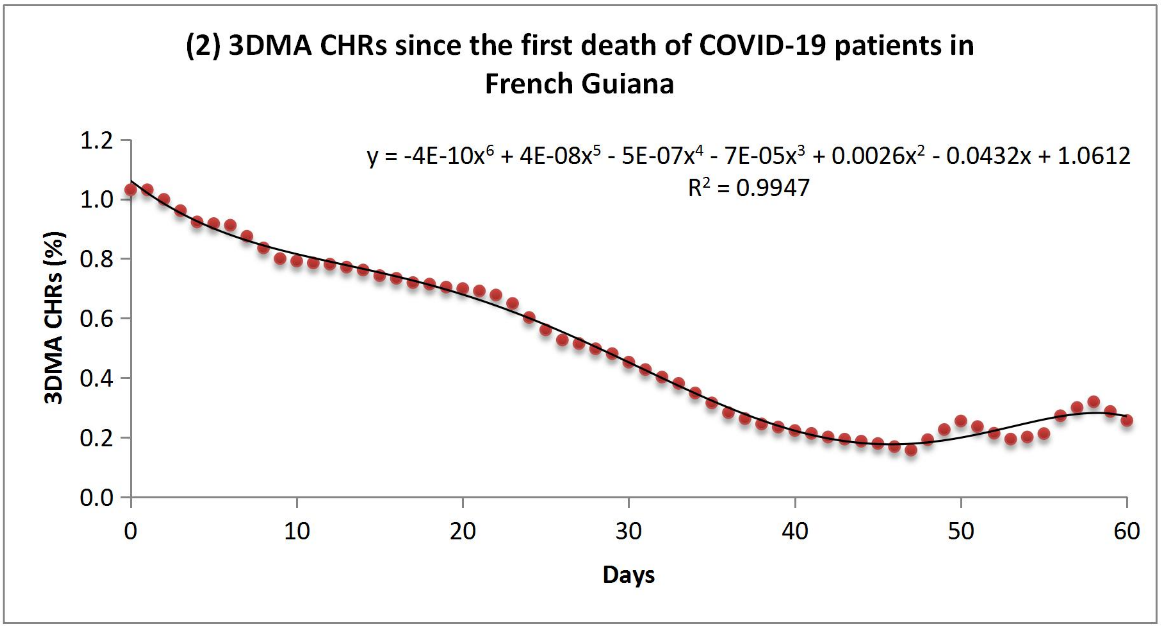
(2) 3DMA CHRs since the first death of COVID-19 patients in French Guiana
3DMA CHRs (%)
1.2
1.0
0.8
0.6
0.4
0.2
0.0
y = -4E-10x⁶ + 4E-08x⁵ - 5E-07x⁴ - 7E-05x³ + 0.0026x² - 0.0432x + 1.0612
R² = 0.9947
0
10
20
30
40
50
60
Days

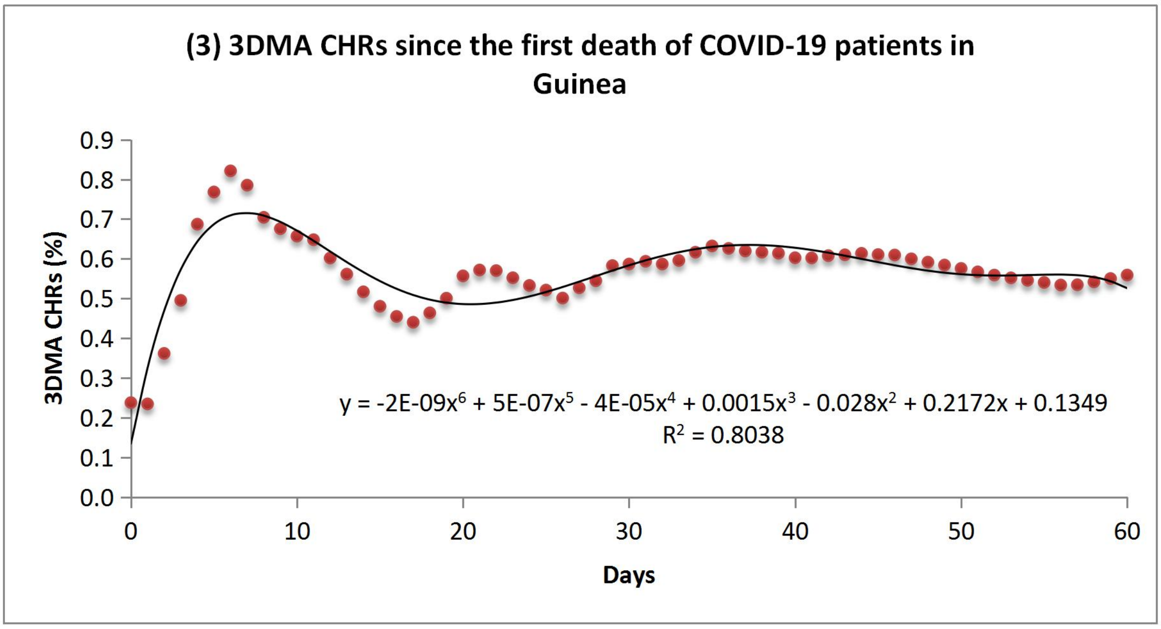
(3) 3DMA CHRs since the first death of COVID-19 patients in Guinea
0.9
0.8
0.7
0.6
0.5
0.4
0.3
0.2
0.1
0.0
3DMA CHRs (%)
y = -2E-09x6 + 5E-07x5 - 4E-05x4 + 0.0015x3 - 0.028x2 + 0.2172x + 0.1349
R2 = 0.8038
0
10
20
30
40
50
60
Days

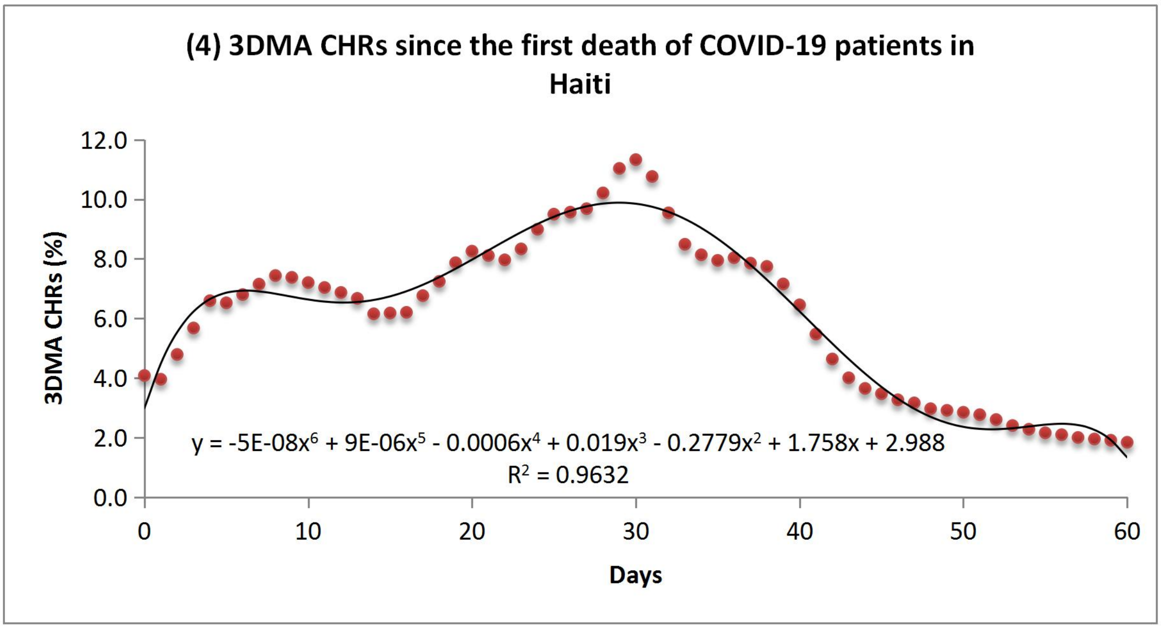
(4) 3DMA CHRs since the first death of COVID-19 patients in Haiti
12.0
10.0
8.0
6.0
4.0
2.0
0.0
3DMA CHRs (%)
y = -5E-08x6 + 9E-06x5 - 0.0006x4 + 0.019x3 - 0.2779x2 + 1.758x + 2.988
R2 = 0.9632
0
10
20
30
40
50
60
Days

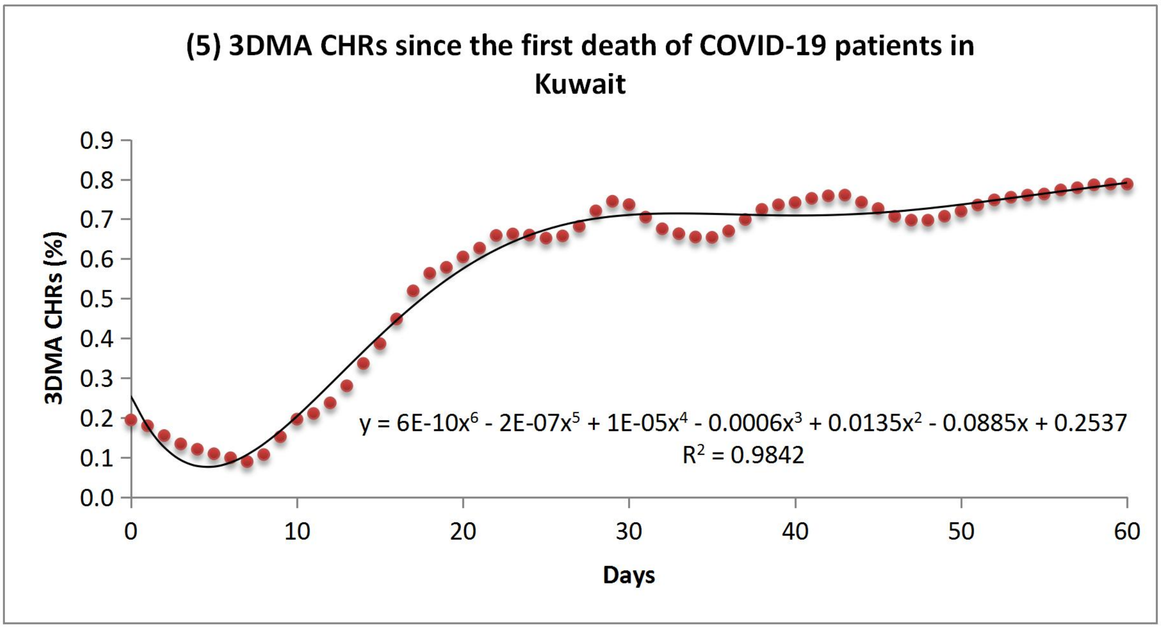
(5) 3DMA CHRs since the first death of COVID-19 patients in Kuwait
3DMA CHRs (%)
0.0
0.1
0.2
0.3
0.4
0.5
0.6
0.7
0.8
0.9
0
10
20
30
40
50
60
Days
y = 6E-10x6 - 2E-07x5 + 1E-05x4 - 0.0006x3 + 0.0135x2 - 0.0885x + 0.2537
R2 = 0.9842

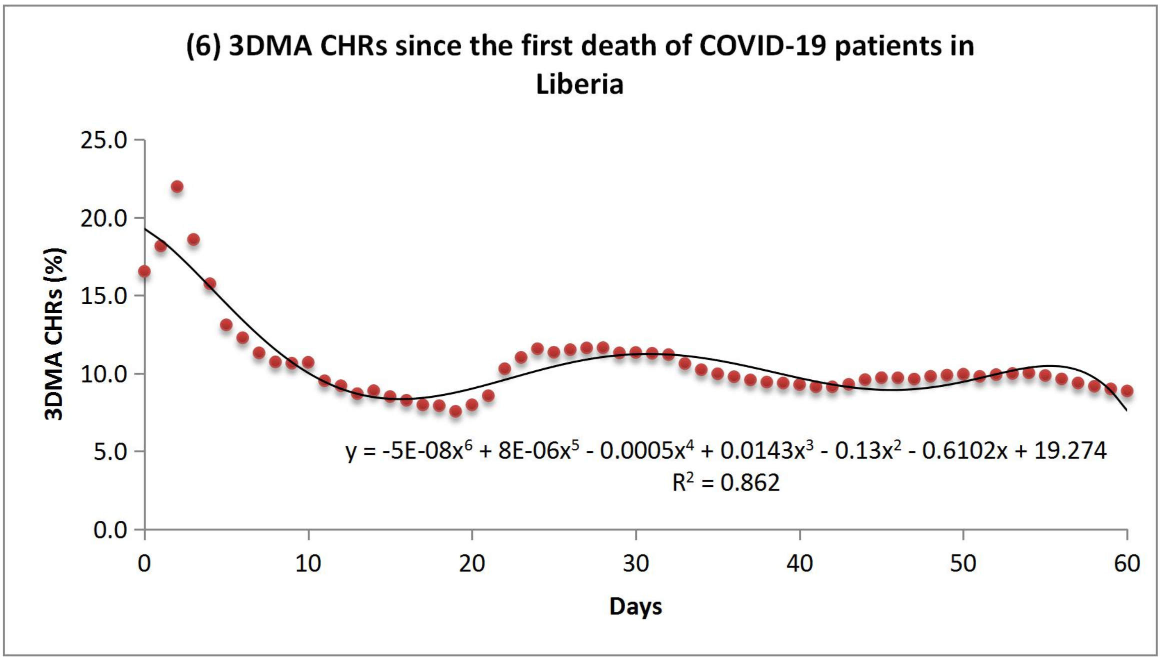
(6) 3DMA CHRs since the first death of COVID-19 patients in Liberia
3DMA CHRs (%)
0.0
5.0
10.0
15.0
20.0
25.0
0
10
20
30
40
50
60
Days
y = -5E-08x6 + 8E-06x5 - 0.0005x4 + 0.0143x3 - 0.13x2 - 0.6102x + 19.274
R2 = 0.862

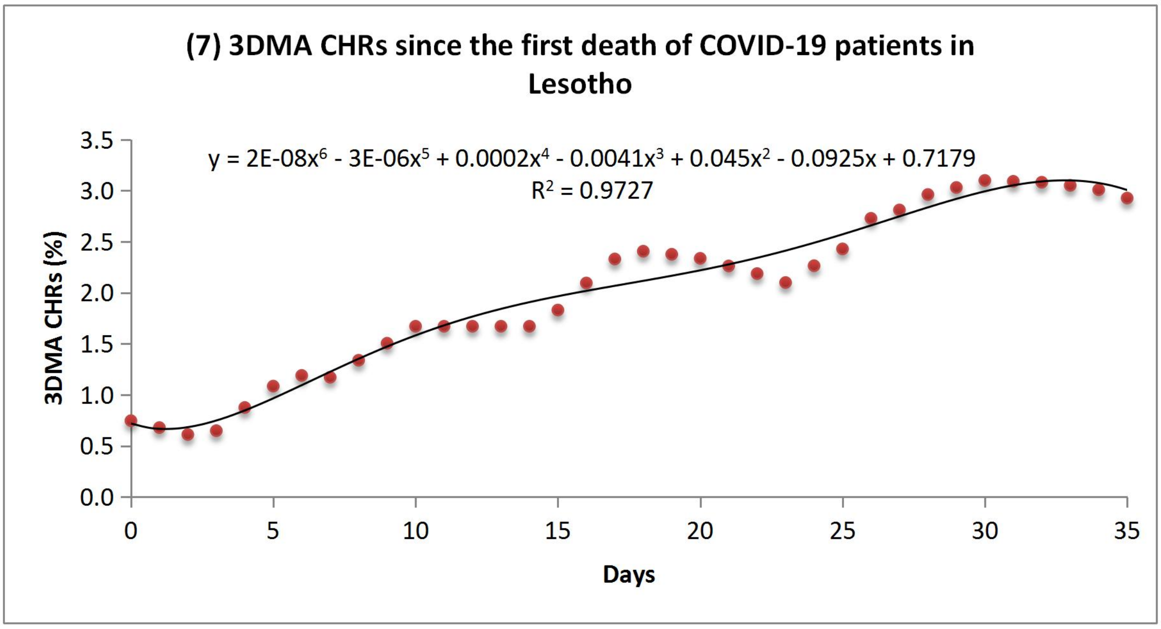
(7) 3DMA CHRs since the first death of COVID-19 patients in Lesotho
y = 2E-08x⁶ - 3E-06x⁵ + 0.0002x⁴ - 0.0041x³ + 0.045x² - 0.0925x + 0.7179
R² = 0.9727
3DMA CHRs (%)
3.5
3.0
2.5
2.0
1.5
1.0
0.5
0.0
0
5
10
15
20
25
30
35
Days

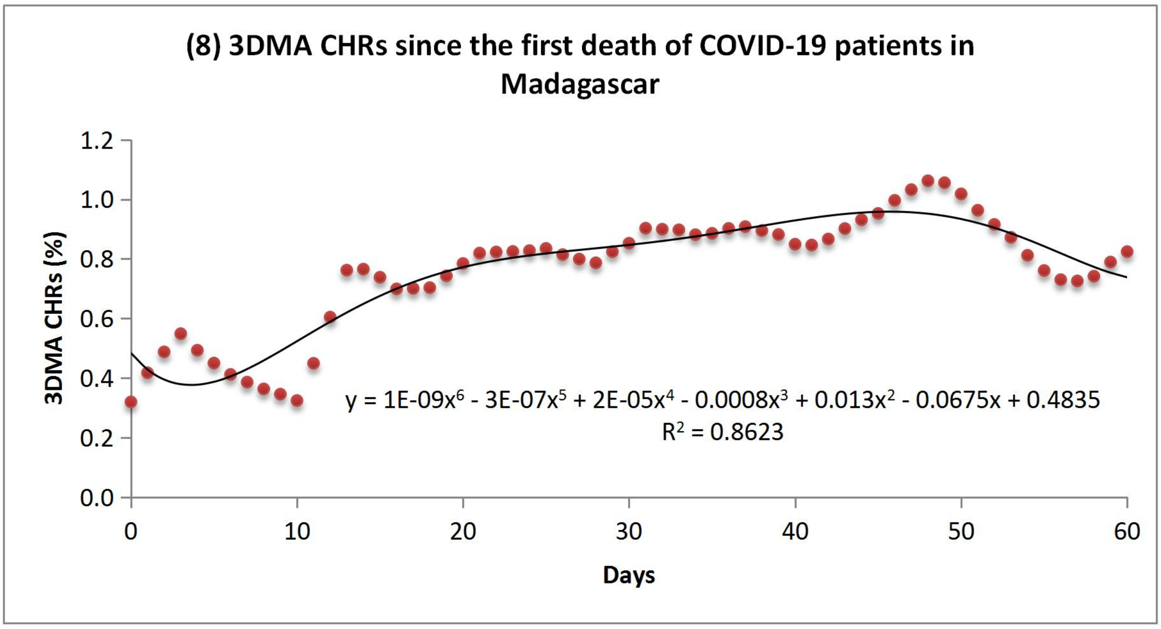
(8) 3DMA CHRs since the first death of COVID-19 patients in Madagascar
y = 1E-09x⁶ - 3E-07x⁵ + 2E-05x⁴ - 0.0008x³ + 0.013x² - 0.0675x + 0.4835
R² = 0.8623
3DMA CHRs (%)
1.2
1.0
0.8
0.6
0.4
0.2
0.0
0
10
20
30
40
50
60
Days

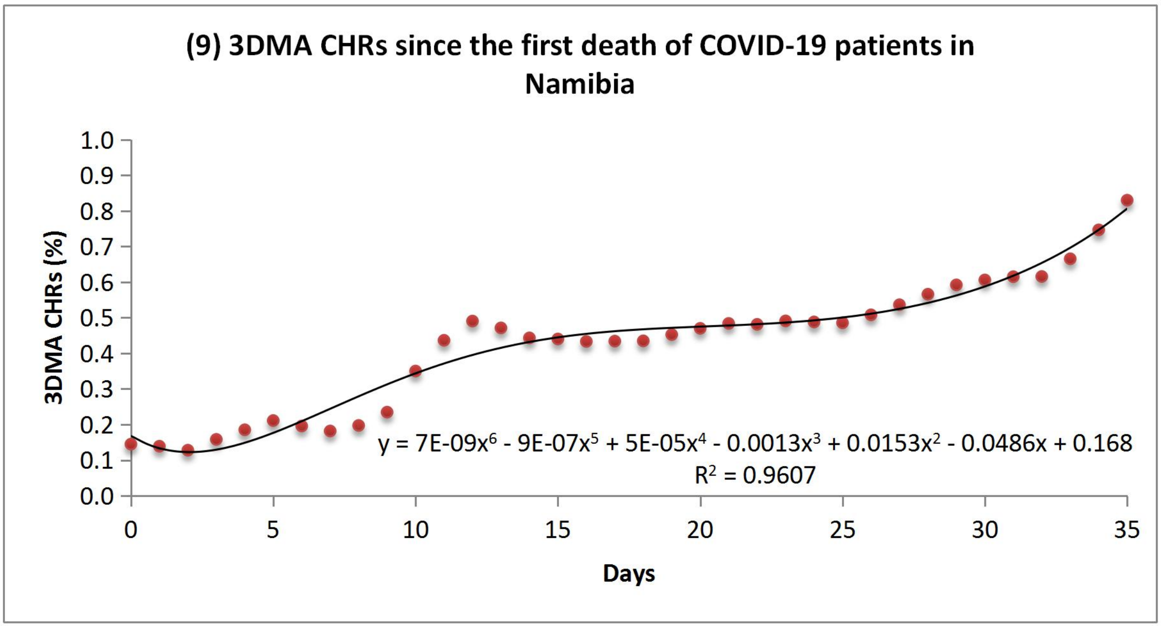
(9) 3DMA CHRs since the first death of COVID-19 patients in Namibia
3DMA CHRs (%)
Days
y = 7E-09x6 - 9E-07x5 + 5E-05x4 - 0.0013x3 + 0.0153x2 - 0.0486x + 0.168
R2 = 0.9607

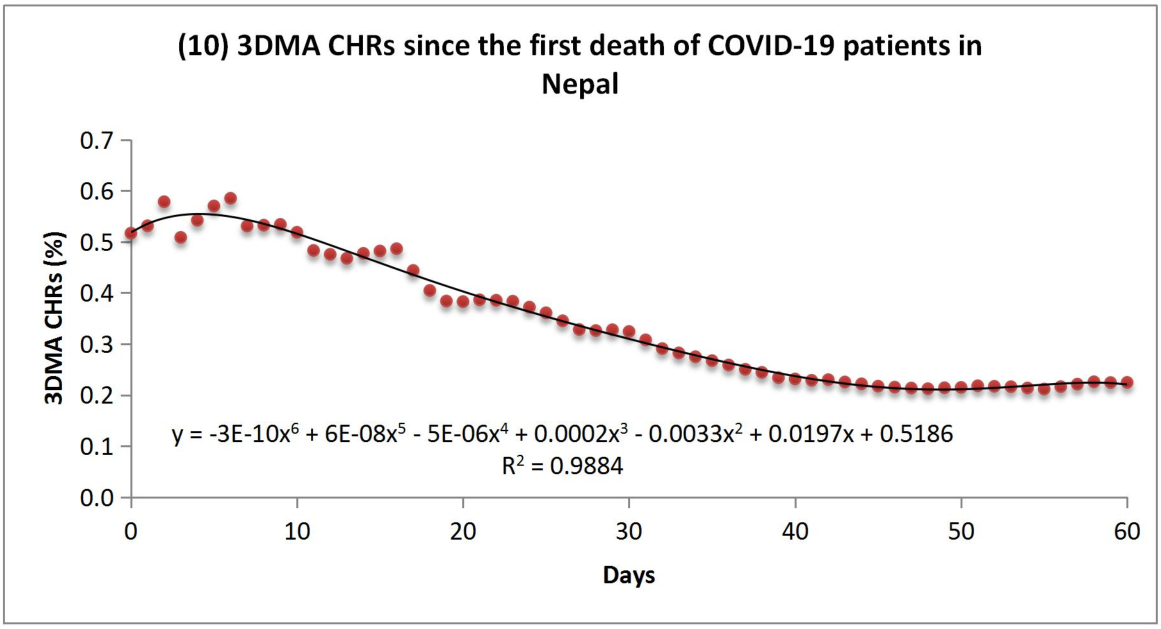
(10) 3DMA CHRs since the first death of COVID-19 patients in Nepal
3DMA CHRs (%)
Days
y = -3E-10x6 + 6E-08x5 - 5E-06x4 + 0.0002x3 - 0.0033x2 + 0.0197x + 0.5186
R2 = 0.9884

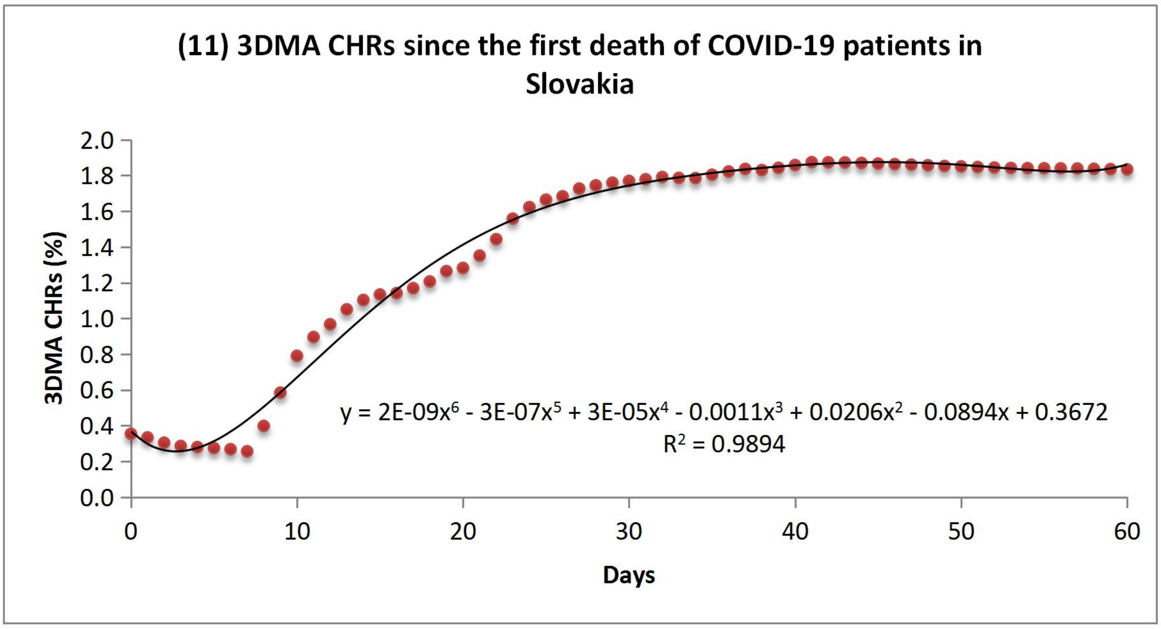

(11) 3DMA CHRs since the first death of COVID-19 patients in Slovakia
3DMA CHRs (%)
2.0
1.8
1.6
1.4
1.2
1.0
0.8
0.6
0.4
0.2
0.0
0
10
20
30
40
50
60
Days
y = 2E-09x6 - 3E-07x5 + 3E-05x4 - 0.0011x3 + 0.0206x2 - 0.0894x + 0.3672
R2 = 0.9894

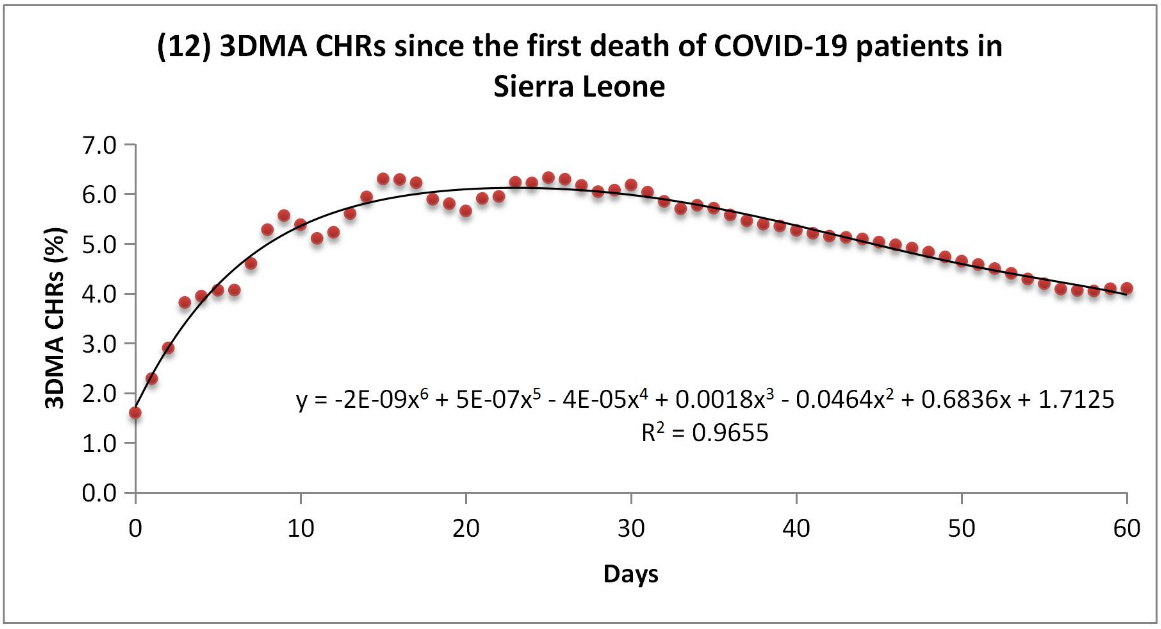

(12) 3DMA CHRs since the first death of COVID-19 patients in Sierra Leone
3DMA CHRs (%)
7.0
6.0
5.0
4.0
3.0
2.0
1.0
0.0
0
10
20
30
40
50
60
Days
y = -2E-09x6 + 5E-07x5 - 4E-05x4 + 0.0018x3 - 0.0464x2 + 0.6836x + 1.7125
R2 = 0.9655

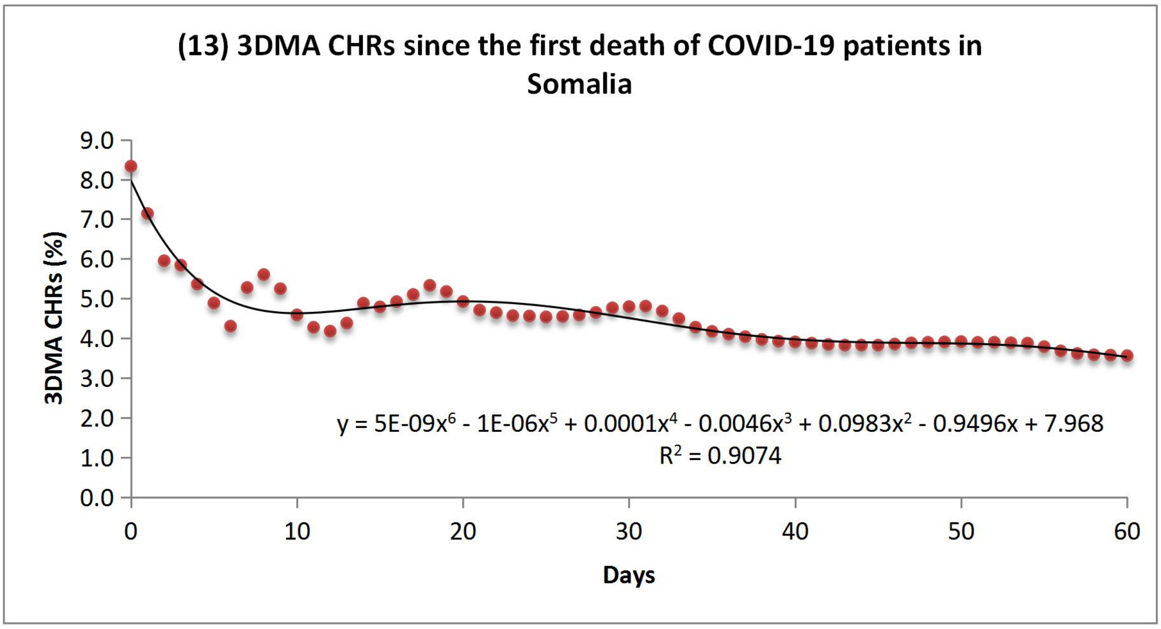
(13) 3DMA CHRs since the first death of COVID-19 patients in Somalia
3DMA CHRs (%)
9.0
8.0
7.0
6.0
5.0
4.0
3.0
2.0
1.0
0.0
0
10
20
30
40
50
60
Days
y = 5E-09x⁶ - 1E-06x⁵ + 0.0001x⁴ - 0.0046x³ + 0.0983x² - 0.9496x + 7.968
R² = 0.9074

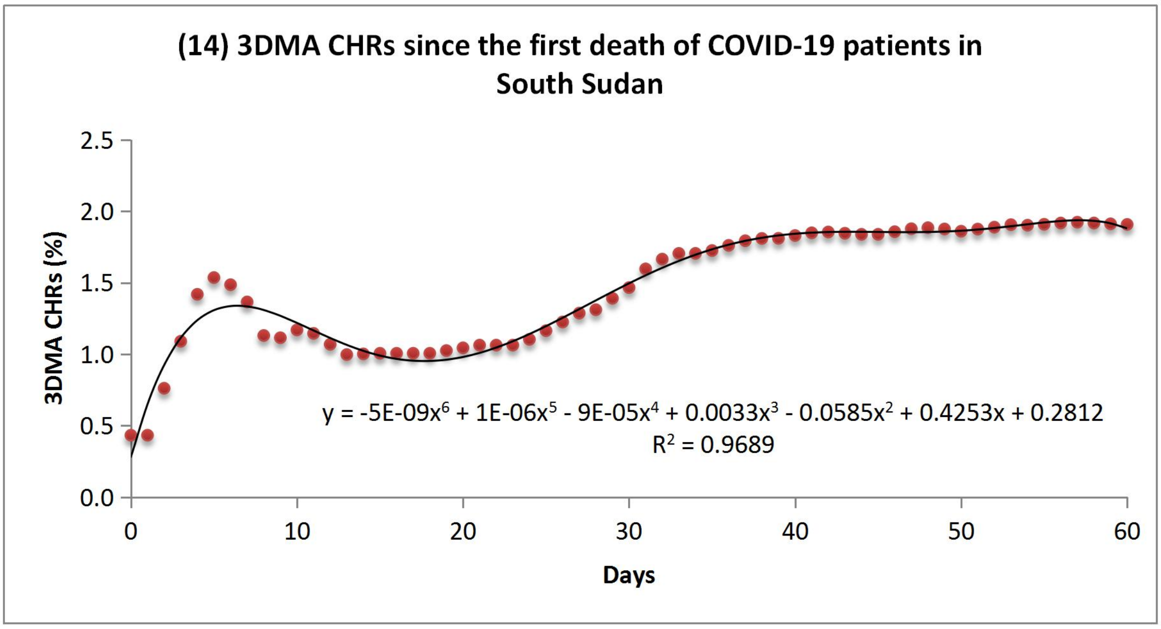
(14) 3DMA CHRs since the first death of COVID-19 patients in South Sudan
3DMA CHRs (%)
2.5
2.0
1.5
1.0
0.5
0.0
0
10
20
30
40
50
60
Days
y = -5E-09x⁶ + 1E-06x⁵ - 9E-05x⁴ + 0.0033x³ - 0.0585x² + 0.4253x + 0.2812
R² = 0.9689

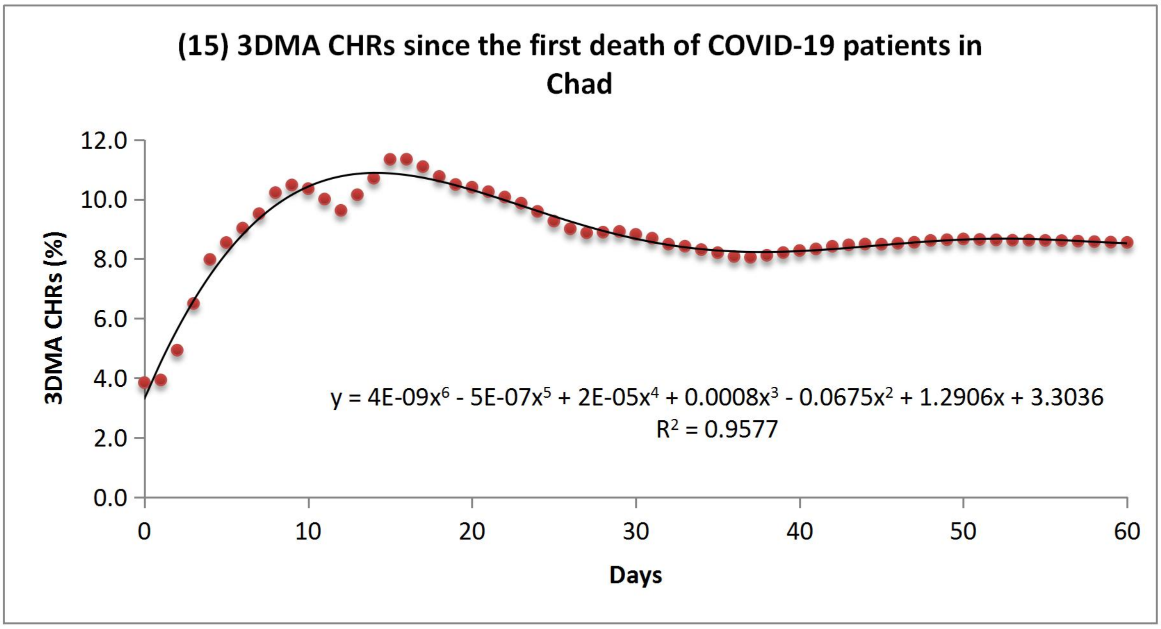
(15) 3DMA CHRs since the first death of COVID-19 patients in Chad
3DMA CHRs (%)
Days
y = 4E-09x6 - 5E-07x5 + 2E-05x4 + 0.0008x3 - 0.0675x2 + 1.2906x + 3.3036
R2 = 0.9577

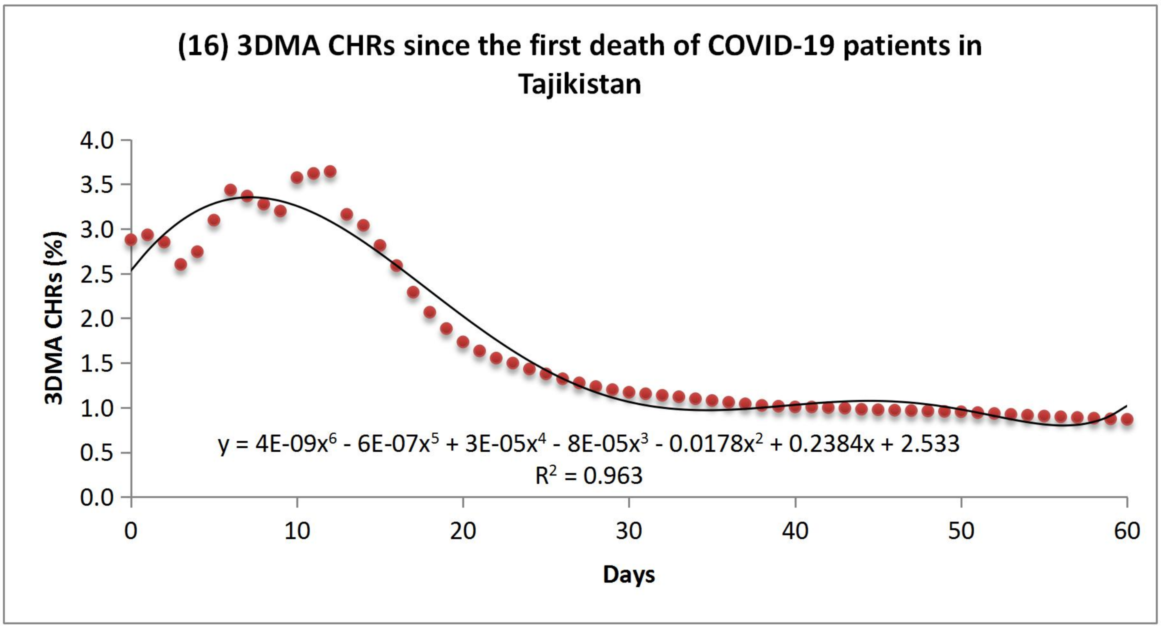
(16) 3DMA CHRs since the first death of COVID-19 patients in Tajikistan
3DMA CHRs (%)
Days
y = 4E-09x6 - 6E-07x5 + 3E-05x4 - 8E-05x3 - 0.0178x2 + 0.2384x + 2.533
R2 = 0.963

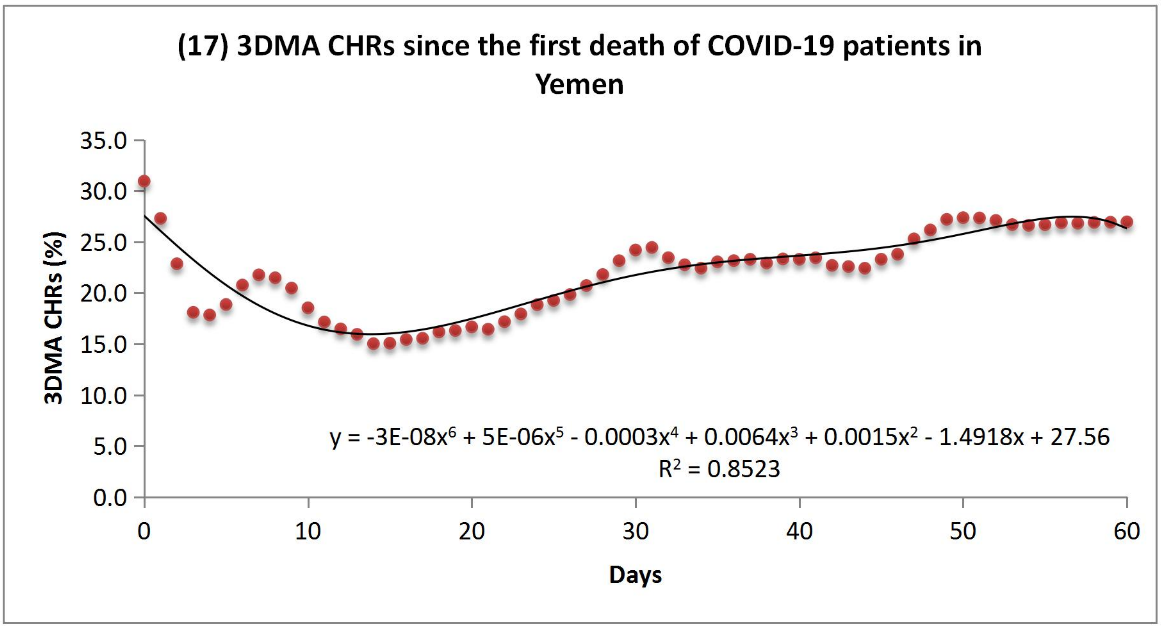
(17) 3DMA CHRs since the first death of COVID-19 patients in Yemen
35.0
30.0
25.0
20.0
15.0
10.0
5.0
0.0
3DMA CHRs (%)
y = -3E-08x6 + 5E-06x5 - 0.0003x4 + 0.0064x3 + 0.0015x2 - 1.4918x + 27.56
R2 = 0.8523
0
10
20
30
40
50
60
Days
