# Supplementary material for: Predictive Model for National Minimal CFR during Spontaneous Initial Outbreak of Emerging Infectious Disease: Lessons from COVID-19 Pandemic in 214 Nations and Regions
Source: Int J Environ Res Public Health. 2022 Dec 29;20(1):594. doi: 10.3390/ijerph20010594 (PMC9819427; doi:10.3390/ijerph20010594)
Supplement: Supplementary file 1 [file ijerph-20-00594-s001.zip › Table S1 Daily confirmed cases in 214 nations.pdf]

Table S1 Daily confirmed cases and daily deaths of COVID-19 in 214 nations from WHO

ID	Date and time	Country or region	Continent	Confirmed patients	Cumulative Confirmed patients	Daily dead patients	Daily cumulative dead patients	Lag days	Daily CFR	3DMA CFR
1	2020-2-24 8:00	AF	EMRO	1	1	0	0			
2	2020-2-25 8:00	AF	EMRO	0	1	0	0			
3	2020-2-26 8:00	AF	EMRO	0	1	0	0			
4	2020-2-27 8:00	AF	EMRO	0	1	0	0			
5	2020-2-28 8:00	AF	EMRO	0	1	0	0			
6	2020-2-29 8:00	AF	EMRO	0	1	0	0			
7	2020-3-1 8:00	AF	EMRO	0	1	0	0			
8	2020-3-2 8:00	AF	EMRO	0	1	0	0			
9	2020-3-3 8:00	AF	EMRO	0	1	0	0			
10	2020-3-4 8:00	AF	EMRO	0	1	0	0			
11	2020-3-5 8:00	AF	EMRO	0	1	0	0			
12	2020-3-6 8:00	AF	EMRO	0	1	0	0			
13	2020-3-7 8:00	AF	EMRO	3	4	0	0			
14	2020-3-8 8:00	AF	EMRO	0	4	0	0			
15	2020-3-9 8:00	AF	EMRO	0	4	0	0			
16	2020-3-10 8:00	AF	EMRO	0	4	0	0			
17	2020-3-11 8:00	AF	EMRO	3	7	0	0			
18	2020-3-12 8:00	AF	EMRO	0	7	0	0			
19	2020-3-13 8:00	AF	EMRO	0	7	0	0			
20	2020-3-14 8:00	AF	EMRO	3	10	0	0			
21	2020-3-15 8:00	AF	EMRO	6	16	0	0			
22	2020-3-16 8:00	AF	EMRO	5	21	0	0			
23	2020-3-17 8:00	AF	EMRO	1	22	0	0			
24	2020-3-18 8:00	AF	EMRO	0	22	0	0			
25	2020-3-19 8:00	AF	EMRO	0	22	0	0			
26	2020-3-20 8:00	AF	EMRO	2	24	0	0			
27	2020-3-21 8:00	AF	EMRO	0	24	0	0			
28	2020-3-22 8:00	AF	EMRO	10	34	0	0			
29	2020-3-23 8:00	AF	EMRO	6	40	1	1	0	2.500	2.440
30	2020-3-24 8:00	AF	EMRO	2	42	0	1	1	2.381	2.077
31	2020-3-25 8:00	AF	EMRO	32	74	0	1	2	1.351	2.077
32	2020-3-26 8:00	AF	EMRO	6	80	1	2	3	2.500	2.016
33	2020-3-27 8:00	AF	EMRO	11	91	0	2	4	2.198	2.509
34	2020-3-28 8:00	AF	EMRO	15	106	1	3	5	2.830	2.846
35	2020-3-29 8:00	AF	EMRO	8	114	1	4	6	3.509	3.283
36	2020-3-30 8:00	AF	EMRO	0	114	0	4	7	3.509	3.142

Table S1 Daily confirmed cases and daily deaths of COVID-19 in 214 nations from WHO

ID	Date and time	Country or region	Continent	Confirmed patients	Cumulative Confirmed patients	Daily dead patients	Daily cumulative dead patients	Lag days	Daily CFR	3DMA CFR
37	2020-3-31 8:00	AF	EMRO	52	166	0	4	8	2.410	2.667
38	2020-4-1 8:00	AF	EMRO	26	192	0	4	9	2.083	2.065
39	2020-4-2 8:00	AF	EMRO	43	235	0	4	10	1.702	1.881
40	2020-4-3 8:00	AF	EMRO	34	269	1	5	11	1.859	1.804
41	2020-4-4 8:00	AF	EMRO	1	270	0	5	12	1.852	2.017
42	2020-4-5 8:00	AF	EMRO	29	299	2	7	13	2.341	2.090
43	2020-4-6 8:00	AF	EMRO	38	337	0	7	14	2.077	2.472
44	2020-4-7 8:00	AF	EMRO	30	367	4	11	15	2.997	2.795
45	2020-4-8 8:00	AF	EMRO	56	423	3	14	16	3.310	3.135
46	2020-4-9 8:00	AF	EMRO	61	484	1	15	17	3.099	3.096
47	2020-4-10 8:00	AF	EMRO	37	521	0	15	18	2.879	2.952
48	2020-4-11 8:00	AF	EMRO	0	521	0	15	19	2.879	3.000
49	2020-4-12 8:00	AF	EMRO	34	555	3	18	20	3.243	3.093
50	2020-4-13 8:00	AF	EMRO	110	665	3	21	21	3.158	3.207
51	2020-4-14 8:00	AF	EMRO	49	714	2	23	22	3.221	3.189
52	2020-4-15 8:00	AF	EMRO	70	784	2	25	23	3.189	3.354
53	2020-4-16 8:00	AF	EMRO	10	794	4	29	24	3.652	3.464
54	2020-4-17 8:00	AF	EMRO	51	845	1	30	25	3.550	3.502
55	2020-4-18 8:00	AF	EMRO	63	908	0	30	26	3.304	3.389
56	2020-4-19 8:00	AF	EMRO	88	996	3	33	27	3.313	3.310
57	2020-4-20 8:00	AF	EMRO	0	996	0	33	28	3.313	3.308
58	2020-4-21 8:00	AF	EMRO	96	1092	3	36	29	3.297	3.337
59	2020-4-22 8:00	AF	EMRO	84	1176	4	40	30	3.401	3.366
60	2020-4-23 8:00	AF	EMRO	0	1176	0	40	31	3.401	3.355
61	2020-4-24 8:00	AF	EMRO	50	1226	0	40	32	3.263	3.292
62	2020-4-25 8:00	AF	EMRO	237	1463	7	47	33	3.213	3.275
63	2020-4-26 8:00	AF	EMRO	0	1463	2	49	34	3.349	3.303
64	2020-4-27 8:00	AF	EMRO	240	1703	8	57	35	3.347	3.327
65	2020-4-28 8:00	AF	EMRO	124	1827	3	60	36	3.284	3.305
66	2020-4-29 8:00	AF	EMRO	0	1827	0	60	37	3.284	3.172
67	2020-4-30 8:00	AF	EMRO	344	2171	4	64	38	2.948	3.060
68	2020-5-1 8:00	AF	EMRO	0	2171	0	64	39	2.948	2.937
69	2020-5-2 8:00	AF	EMRO	298	2469	8	72	40	2.916	2.932
70										
71	2020-3-9 8:00	AL	EURO	2	2	0	0			
72	2020-3-10 8:00	AL	EURO	4	6	0	0			

Table S1 Daily confirmed cases and daily deaths of COVID-19 in 214 nations from WHO

ID	Date and time	Country or region	Continent	Confirmed patients	Cumulative Confirmed patients	Daily dead patients	Daily cumulative dead patients	Lag days	Daily CFR	3DMA CFR
73	2020-3-11 8:00	AL	EURO	4	10	0	0			
74	2020-3-12 8:00	AL	EURO	13	23	0	0			
75	2020-3-13 8:00	AL	EURO	10	33	1	1	0	3.030	2.831
76	2020-3-14 8:00	AL	EURO	5	38	0	1	1	2.632	2.681
77	2020-3-15 8:00	AL	EURO	4	42	0	1	2	2.381	2.324
78	2020-3-16 8:00	AL	EURO	9	51	0	1	3	1.961	2.053
79	2020-3-17 8:00	AL	EURO	4	55	0	1	4	1.818	2.390
80	2020-3-18 8:00	AL	EURO	4	59	1	2	5	3.390	2.688
81	2020-3-19 8:00	AL	EURO	11	70	0	2	6	2.857	3.035
82	2020-3-20 8:00	AL	EURO	0	70	0	2	7	2.857	2.782
83	2020-3-21 8:00	AL	EURO	6	76	0	2	8	2.632	2.707
84	2020-3-22 8:00	AL	EURO	0	76	0	2	9	2.632	2.503
85	2020-3-23 8:00	AL	EURO	13	89	0	2	10	2.247	2.710
86	2020-3-24 8:00	AL	EURO	34	123	2	4	11	3.252	2.975
87	2020-3-25 8:00	AL	EURO	23	146	1	5	12	3.425	3.183
88	2020-3-26 8:00	AL	EURO	28	174	0	5	13	2.874	3.533
89	2020-3-27 8:00	AL	EURO	12	186	3	8	14	4.301	3.914
90	2020-3-28 8:00	AL	EURO	11	197	1	9	15	4.569	4.529
91	2020-3-29 8:00	AL	EURO	15	212	1	10	16	4.717	4.590
92	2020-3-30 8:00	AL	EURO	11	223	0	10	17	4.484	4.711
93	2020-3-31 8:00	AL	EURO	0	223	1	11	18	4.933	4.922
94	2020-4-1 8:00	AL	EURO	20	243	2	13	19	5.350	5.233
95	2020-4-2 8:00	AL	EURO	34	277	2	15	20	5.415	5.514
96	2020-4-3 8:00	AL	EURO	0	277	1	16	21	5.776	5.432
97	2020-4-4 8:00	AL	EURO	56	333	1	17	22	5.105	5.429
98	2020-4-5 8:00	AL	EURO	0	333	1	18	23	5.405	5.360
99	2020-4-6 8:00	AL	EURO	44	377	3	21	24	5.570	5.604
100	2020-4-7 8:00	AL	EURO	0	377	1	22	25	5.836	5.635
101	2020-4-8 8:00	AL	EURO	23	400	0	22	26	5.500	5.572
102	2020-4-9 8:00	AL	EURO	9	409	0	22	27	5.379	5.469
103	2020-4-10 8:00	AL	EURO	7	416	1	23	28	5.529	5.479
104	2020-4-11 8:00	AL	EURO	0	416	0	23	29	5.529	5.405
105	2020-4-12 8:00	AL	EURO	30	446	0	23	30	5.157	5.281
106	2020-4-13 8:00	AL	EURO	0	446	0	23	31	5.157	5.052
107	2020-4-14 8:00	AL	EURO	29	475	0	23	32	4.842	5.017
108	2020-4-15 8:00	AL	EURO	0	475	1	24	33	5.053	4.985

Table S1 Daily confirmed cases and daily deaths of COVID-19 in 214 nations from WHO

ID	Date and time	Country or region	Continent	Confirmed patients	Cumulative Confirmed patients	Daily dead patients	Daily cumulative dead patients	Lag days	Daily CFR	3DMA CFR
109	2020-4-16 8:00	AL	EURO	19	494	1	25	34	5.061	5.044
110	2020-4-17 8:00	AL	EURO	24	518	1	26	35	5.019	4.942
111	2020-4-18 8:00	AL	EURO	30	548	0	26	36	4.745	4.836
112	2020-4-19 8:00	AL	EURO	0	548	0	26	37	4.745	4.705
113	2020-4-20 8:00	AL	EURO	14	562	0	26	38	4.626	4.547
114	2020-4-21 8:00	AL	EURO	47	609	0	26	39	4.269	4.332
115	2020-4-22 8:00	AL	EURO	25	634	0	26	40	4.101	4.210
116	2020-4-23 8:00	AL	EURO	0	634	1	27	41	4.259	4.114
117	2020-4-24 8:00	AL	EURO	44	678	0	27	42	3.982	4.074
118	2020-4-25 8:00	AL	EURO	0	678	0	27	43	3.982	3.919
119	2020-4-26 8:00	AL	EURO	34	712	0	27	44	3.792	3.877
120	2020-4-27 8:00	AL	EURO	14	726	1	28	45	3.857	3.818
121	2020-4-28 8:00	AL	EURO	10	736	0	28	46	3.804	3.859
122	2020-4-29 8:00	AL	EURO	30	766	2	30	47	3.916	3.910
123	2020-4-30 8:00	AL	EURO	7	773	1	31	48	4.010	3.979
124	2020-5-1 8:00	AL	EURO	0	773	0	31	49	4.010	3.983
125	2020-5-2 8:00	AL	EURO	16	789	0	31	50	3.929	3.970
126										
127	2020-2-25 8:00	DZ	AFRO	1	1	0	0			
128	2020-2-26 8:00	DZ	AFRO	0	1	0	0			
129	2020-2-27 8:00	DZ	AFRO	0	1	0	0			
130	2020-2-28 8:00	DZ	AFRO	0	1	0	0			
131	2020-2-29 8:00	DZ	AFRO	0	1	0	0			
132	2020-3-1 8:00	DZ	AFRO	0	1	0	0			
133	2020-3-2 8:00	DZ	AFRO	2	3	0	0			
134	2020-3-3 8:00	DZ	AFRO	2	5	0	0			
135	2020-3-4 8:00	DZ	AFRO	7	12	0	0			
136	2020-3-5 8:00	DZ	AFRO	0	12	0	0			
137	2020-3-6 8:00	DZ	AFRO	5	17	0	0			
138	2020-3-7 8:00	DZ	AFRO	0	17	0	0			
139	2020-3-8 8:00	DZ	AFRO	0	17	0	0			
140	2020-3-9 8:00	DZ	AFRO	3	20	0	0			
141	2020-3-10 8:00	DZ	AFRO	0	20	0	0			
142	2020-3-11 8:00	DZ	AFRO	0	20	0	0			
143	2020-3-12 8:00	DZ	AFRO	5	25	1	1	0	4.000	4.000
144	2020-3-13 8:00	DZ	AFRO	0	25	0	1	1	4.000	4.468

Table S1 Daily confirmed cases and daily deaths of COVID-19 in 214 nations from WHO

ID	Date and time	Country or region	Continent	Confirmed patients	Cumulative Confirmed patients	Daily dead patients	Daily cumulative dead patients	Lag days	Daily CFR	3DMA CFR
145	2020-3-14 8:00	DZ	AFRO	12	37	1	2	2	5.405	5.176
146	2020-3-15 8:00	DZ	AFRO	12	49	1	3	3	6.122	6.564
147	2020-3-16 8:00	DZ	AFRO	0	49	1	4	4	8.163	6.984
148	2020-3-17 8:00	DZ	AFRO	11	60	0	4	5	6.667	7.721
149	2020-3-18 8:00	DZ	AFRO	12	72	2	6	6	8.333	7.846
150	2020-3-19 8:00	DZ	AFRO	10	82	1	7	7	8.537	9.169
151	2020-3-20 8:00	DZ	AFRO	12	94	3	10	8	10.638	11.711
152	2020-3-21 8:00	DZ	AFRO	0	94	5	15	9	15.957	12.462
153	2020-3-22 8:00	DZ	AFRO	45	139	0	15	10	10.791	11.736
154	2020-3-23 8:00	DZ	AFRO	62	201	2	17	11	8.458	8.869
155	2020-3-24 8:00	DZ	AFRO	30	231	0	17	12	7.359	7.419
156	2020-3-25 8:00	DZ	AFRO	33	264	0	17	13	6.439	6.746
157	2020-3-26 8:00	DZ	AFRO	0	264	0	17	14	6.439	6.588
158	2020-3-27 8:00	DZ	AFRO	41	305	4	21	15	6.885	6.712
159	2020-3-28 8:00	DZ	AFRO	62	367	4	25	16	6.812	6.685
160	2020-3-29 8:00	DZ	AFRO	42	409	1	26	17	6.357	6.519
161	2020-3-30 8:00	DZ	AFRO	45	454	3	29	18	6.388	6.270
162	2020-3-31 8:00	DZ	AFRO	57	511	2	31	19	6.067	6.149
163	2020-4-1 8:00	DZ	AFRO	73	584	4	35	20	5.993	6.302
164	2020-4-2 8:00	DZ	AFRO	263	847	23	58	21	6.848	7.086
165	2020-4-3 8:00	DZ	AFRO	139	986	25	83	22	8.418	8.077
166	2020-4-4 8:00	DZ	AFRO	185	1171	22	105	23	8.967	9.259
167	2020-4-5 8:00	DZ	AFRO	80	1251	25	130	24	10.392	9.917
168	2020-4-6 8:00	DZ	AFRO	0	1251	0	130	25	10.392	10.980
169	2020-4-7 8:00	DZ	AFRO	172	1423	43	173	26	12.157	11.921
170	2020-4-8 8:00	DZ	AFRO	45	1468	21	194	27	13.215	12.804
171	2020-4-9 8:00	DZ	AFRO	104	1572	11	205	28	13.041	13.454
172	2020-4-10 8:00	DZ	AFRO	94	1666	30	235	29	14.106	13.895
173	2020-4-11 8:00	DZ	AFRO	95	1761	21	256	30	14.537	14.570
174	2020-4-12 8:00	DZ	AFRO	64	1825	19	275	31	15.068	14.971
175	2020-4-13 8:00	DZ	AFRO	89	1914	18	293	32	15.308	15.387
176	2020-4-14 8:00	DZ	AFRO	69	1983	20	313	33	15.784	15.614
177	2020-4-15 8:00	DZ	AFRO	87	2070	13	326	34	15.749	15.696
178	2020-4-16 8:00	DZ	AFRO	90	2160	10	336	35	15.556	15.549
179	2020-4-17 8:00	DZ	AFRO	108	2268	12	348	36	15.344	15.318
180	2020-4-18 8:00	DZ	AFRO	150	2418	16	364	37	15.054	14.960

Table S1 Daily confirmed cases and daily deaths of COVID-19 in 214 nations from WHO

ID	Date and time	Country or region	Continent	Confirmed patients	Cumulative Confirmed patients	Daily dead patients	Daily cumulative dead patients	Lag days	Daily CFR	3DMA CFR
181	2020-4-19 8:00	DZ	AFRO	116	2534	3	367	38	14.483	14.600
182	2020-4-20 8:00	DZ	AFRO	95	2629	8	375	39	14.264	14.292
183	2020-4-21 8:00	DZ	AFRO	89	2718	9	384	40	14.128	14.112
184	2020-4-22 8:00	DZ	AFRO	93	2811	8	392	41	13.945	13.963
185	2020-4-23 8:00	DZ	AFRO	99	2910	10	402	42	13.814	13.765
186	2020-4-24 8:00	DZ	AFRO	97	3007	5	407	43	13.535	13.540
187	2020-4-25 8:00	DZ	AFRO	120	3127	8	415	44	13.272	13.225
188	2020-4-26 8:00	DZ	AFRO	129	3256	4	419	45	12.869	12.902
189	2020-4-27 8:00	DZ	AFRO	126	3382	6	425	46	12.567	12.573
190	2020-4-28 8:00	DZ	AFRO	135	3517	7	432	47	12.283	12.275
191	2020-4-29 8:00	DZ	AFRO	132	3649	5	437	48	11.976	11.933
192	2020-4-30 8:00	DZ	AFRO	199	3848	7	444	49	11.538	11.582
193	2020-5-1 8:00	DZ	AFRO	158	4006	6	450	50	11.233	11.226
194	2020-5-2 8:00	DZ	AFRO	148	4154	3	453	51	10.905	11.069
195										
196	2020-3-2 8:00	AD	EURO	1	1	0	0			
197	2020-3-3 8:00	AD	EURO	0	1	0	0			
198	2020-3-4 8:00	AD	EURO	0	1	0	0			
199	2020-3-5 8:00	AD	EURO	0	1	0	0			
200	2020-3-6 8:00	AD	EURO	0	1	0	0			
201	2020-3-7 8:00	AD	EURO	0	1	0	0			
202	2020-3-8 8:00	AD	EURO	0	1	0	0			
203	2020-3-9 8:00	AD	EURO	0	1	0	0			
204	2020-3-10 8:00	AD	EURO	0	1	0	0			
205	2020-3-11 8:00	AD	EURO	0	1	0	0			
206	2020-3-12 8:00	AD	EURO	0	1	0	0			
207	2020-3-13 8:00	AD	EURO	1	2	0	0			
208	2020-3-14 8:00	AD	EURO	0	2	0	0			
209	2020-3-15 8:00	AD	EURO	0	2	0	0			
210	2020-3-16 8:00	AD	EURO	12	14	0	0			
211	2020-3-17 8:00	AD	EURO	2	16	0	0			
212	2020-3-18 8:00	AD	EURO	23	39	0	0			
213	2020-3-19 8:00	AD	EURO	36	75	0	0			
214	2020-3-20 8:00	AD	EURO	0	75	0	0			
215	2020-3-21 8:00	AD	EURO	13	88	0	0			
216	2020-3-22 8:00	AD	EURO	0	88	0	0			

Table S1 Daily confirmed cases and daily deaths of COVID-19 in 214 nations from WHO

ID	Date and time	Country or region	Continent	Confirmed patients	Cumulative Confirmed patients	Daily dead patients	Daily cumulative dead patients	Lag days	Daily CFR	3DMA CFR
217	2020-3-23 8:00	AD	EURO	25	113	0	0			
218	2020-3-24 8:00	AD	EURO	51	164	1	1	0	0.610	0.571
219	2020-3-25 8:00	AD	EURO	24	188	0	1	1	0.532	0.850
220	2020-3-26 8:00	AD	EURO	25	213	2	3	2	1.408	1.080
221	2020-3-27 8:00	AD	EURO	18	231	0	3	3	1.299	1.248
222	2020-3-28 8:00	AD	EURO	58	289	0	3	4	1.038	1.194
223	2020-3-29 8:00	AD	EURO	32	321	1	4	5	1.246	1.348
224	2020-3-30 8:00	AD	EURO	20	341	2	6	6	1.760	1.723
225	2020-3-31 8:00	AD	EURO	29	370	2	8	7	2.162	2.371
226	2020-4-1 8:00	AD	EURO	6	376	4	12	8	3.191	2.879
227	2020-4-2 8:00	AD	EURO	20	396	1	13	9	3.283	3.324
228	2020-4-3 8:00	AD	EURO	33	429	2	15	10	3.497	3.466
229	2020-4-4 8:00	AD	EURO	13	442	1	16	11	3.620	3.588
230	2020-4-5 8:00	AD	EURO	24	466	1	17	12	3.648	3.506
231	2020-4-6 8:00	AD	EURO	57	523	0	17	13	3.250	3.596
232	2020-4-7 8:00	AD	EURO	17	540	4	21	14	3.889	3.711
233	2020-4-8 8:00	AD	EURO	11	551	1	22	15	3.993	4.041
234	2020-4-9 8:00	AD	EURO	15	566	2	24	16	4.240	4.216
235	2020-4-10 8:00	AD	EURO	23	589	2	26	17	4.414	4.324
236	2020-4-11 8:00	AD	EURO	13	602	0	26	18	4.319	4.412
237	2020-4-12 8:00	AD	EURO	20	622	2	28	19	4.502	4.453
238	2020-4-13 8:00	AD	EURO	17	639	1	29	20	4.538	4.498
239	2020-4-14 8:00	AD	EURO	12	651	0	29	21	4.455	4.566
240	2020-4-15 8:00	AD	EURO	8	659	2	31	22	4.704	4.687
241	2020-4-16 8:00	AD	EURO	14	673	2	33	23	4.903	4.836
242	2020-4-17 8:00	AD	EURO	21	694	1	34	24	4.899	4.927
243	2020-4-18 8:00	AD	EURO	9	703	1	35	25	4.979	4.950
244	2020-4-19 8:00	AD	EURO	1	704	0	35	26	4.972	4.990
245	2020-4-20 8:00	AD	EURO	13	717	1	36	27	5.021	5.002
246	2020-4-21 8:00	AD	EURO	1	718	0	36	28	5.014	5.051
247	2020-4-22 8:00	AD	EURO	5	723	1	37	29	5.118	5.081
248	2020-4-23 8:00	AD	EURO	1	724	0	37	30	5.110	5.154
249	2020-4-24 8:00	AD	EURO	2	726	1	38	31	5.234	5.267
250	2020-4-25 8:00	AD	EURO	7	733	2	40	32	5.457	5.370
251	2020-4-26 8:00	AD	EURO	5	738	0	40	33	5.420	5.427
252	2020-4-27 8:00	AD	EURO	2	740	0	40	34	5.405	5.408

Table S1 Daily confirmed cases and daily deaths of COVID-19 in 214 nations from WHO

ID	Date and time	Country or region	Continent	Confirmed patients	Cumulative Confirmed patients	Daily dead patients	Daily cumulative dead patients	Lag days	Daily CFR	3DMA CFR
253	2020-4-28 8:00	AD	EURO	1	741	0	40	35	5.398	5.443
254	2020-4-29 8:00	AD	EURO	1	742	1	41	36	5.526	5.478
255	2020-4-30 8:00	AD	EURO	2	744	0	41	37	5.511	5.555
256	2020-5-1 8:00	AD	EURO	2	746	1	42	38	5.630	5.635
257	2020-5-2 8:00	AD	EURO	0	746	1	43	39	5.764	5.697
258										
259	2020-3-21 8:00	AO	AFRO	2	2	0	0			
260	2020-3-22 8:00	AO	AFRO	0	2	0	0			
261	2020-3-23 8:00	AO	AFRO	0	2	0	0			
262	2020-3-24 8:00	AO	AFRO	0	2	0	0			
263	2020-3-25 8:00	AO	AFRO	0	2	0	0			
264	2020-3-26 8:00	AO	AFRO	0	2	0	0			
265	2020-3-27 8:00	AO	AFRO	0	2	0	0			
266	2020-3-28 8:00	AO	AFRO	0	2	0	0			
267	2020-3-29 8:00	AO	AFRO	0	2	0	0			
268	2020-3-30 8:00	AO	AFRO	0	2	0	0			
269	2020-3-31 8:00	AO	AFRO	5	7	1	1	0	14.286	21.429
270	2020-4-1 8:00	AO	AFRO	0	7	1	2	1	28.571	22.619
271	2020-4-2 8:00	AO	AFRO	1	8	0	2	2	25.000	26.190
272	2020-4-3 8:00	AO	AFRO	0	8	0	2	3	25.000	23.333
273	2020-4-4 8:00	AO	AFRO	2	10	0	2	4	20.000	21.667
274	2020-4-5 8:00	AO	AFRO	0	10	0	2	5	20.000	18.095
275	2020-4-6 8:00	AO	AFRO	4	14	0	2	6	14.286	15.595
276	2020-4-7 8:00	AO	AFRO	2	16	0	2	7	12.500	12.850
277	2020-4-8 8:00	AO	AFRO	1	17	0	2	8	11.765	11.597
278	2020-4-9 8:00	AO	AFRO	2	19	0	2	9	10.526	10.939
279	2020-4-10 8:00	AO	AFRO	0	19	0	2	10	10.526	10.526
280	2020-4-11 8:00	AO	AFRO	0	19	0	2	11	10.526	10.526
281	2020-4-12 8:00	AO	AFRO	0	19	0	2	12	10.526	10.526
282	2020-4-13 8:00	AO	AFRO	0	19	0	2	13	10.526	10.526
283	2020-4-14 8:00	AO	AFRO	0	19	0	2	14	10.526	10.526
284	2020-4-15 8:00	AO	AFRO	0	19	0	2	15	10.526	10.526
285	2020-4-16 8:00	AO	AFRO	0	19	0	2	16	10.526	10.526
286	2020-4-17 8:00	AO	AFRO	0	19	0	2	17	10.526	10.526
287	2020-4-18 8:00	AO	AFRO	0	19	0	2	18	10.526	10.526
288	2020-4-19 8:00	AO	AFRO	0	19	0	2	19	10.526	10.526

Table S1 Daily confirmed cases and daily deaths of COVID-19 in 214 nations from WHO

ID	Date and time	Country or region	Continent	Confirmed patients	Cumulative Confirmed patients	Daily dead patients	Daily cumulative dead patients	Lag days	Daily CFR	3DMA CFR
289	2020-4-20 8:00	AO	AFRO	0	19	0	2	20	10.526	9.795
290	2020-4-21 8:00	AO	AFRO	5	24	0	2	21	8.333	9.064
291	2020-4-22 8:00	AO	AFRO	0	24	0	2	22	8.333	8.333
292	2020-4-23 8:00	AO	AFRO	0	24	0	2	23	8.333	8.222
293	2020-4-24 8:00	AO	AFRO	1	25	0	2	24	8.000	8.111
294	2020-4-25 8:00	AO	AFRO	0	25	0	2	25	8.000	8.000
295	2020-4-26 8:00	AO	AFRO	0	25	0	2	26	8.000	7.897
296	2020-4-27 8:00	AO	AFRO	1	26	0	2	27	7.692	7.700
297	2020-4-28 8:00	AO	AFRO	1	27	0	2	28	7.407	7.502
298	2020-4-29 8:00	AO	AFRO	0	27	0	2	29	7.407	7.407
299	2020-4-30 8:00	AO	AFRO	0	27	0	2	30	7.407	7.407
300	2020-5-1 8:00	AO	AFRO	0	27	0	2	31	7.407	7.237
301	2020-5-2 8:00	AO	AFRO	2	29	0	2	32	6.897	7.152
302										
303	2020-3-26 8:00	AI	AMRO	2	2	0	0			
304	2020-3-27 8:00	AI	AMRO	0	2	0	0			
305	2020-3-28 8:00	AI	AMRO	0	2	0	0			
306	2020-3-29 8:00	AI	AMRO	0	2	0	0			
307	2020-3-30 8:00	AI	AMRO	0	2	0	0			
308	2020-3-31 8:00	AI	AMRO	0	2	0	0			
309	2020-4-1 8:00	AI	AMRO	0	2	0	0			
310	2020-4-2 8:00	AI	AMRO	0	2	0	0			
311	2020-4-3 8:00	AI	AMRO	0	2	0	0			
312	2020-4-4 8:00	AI	AMRO	1	3	0	0			
313	2020-4-5 8:00	AI	AMRO	0	3	0	0			
314	2020-4-6 8:00	AI	AMRO	0	3	0	0			
315	2020-4-7 8:00	AI	AMRO	0	3	0	0			
316	2020-4-8 8:00	AI	AMRO	0	3	0	0			
317	2020-4-9 8:00	AI	AMRO	0	3	0	0			
318	2020-4-10 8:00	AI	AMRO	0	3	0	0			
319	2020-4-11 8:00	AI	AMRO	0	3	0	0			
320	2020-4-12 8:00	AI	AMRO	0	3	0	0			
321	2020-4-13 8:00	AI	AMRO	0	3	0	0			
322	2020-4-14 8:00	AI	AMRO	0	3	0	0			
323	2020-4-15 8:00	AI	AMRO	0	3	0	0			
324	2020-4-16 8:00	AI	AMRO	0	3	0	0			

Table S1 Daily confirmed cases and daily deaths of COVID-19 in 214 nations from WHO

ID	Date and time	Country or region	Continent	Confirmed patients	Cumulative Confirmed patients	Daily dead patients	Daily cumulative dead patients	Lag days	Daily CFR	3DMA CFR
325	2020-4-17 8:00	AI	AMRO	0	3	0	0			
326	2020-4-18 8:00	AI	AMRO	0	3	0	0			
327	2020-4-19 8:00	AI	AMRO	0	3	0	0			
328	2020-4-20 8:00	AI	AMRO	0	3	0	0			
329	2020-4-21 8:00	AI	AMRO	0	3	0	0			
330	2020-4-22 8:00	AI	AMRO	0	3	0	0			
331	2020-4-23 8:00	AI	AMRO	0	3	0	0			
332	2020-4-24 8:00	AI	AMRO	0	3	0	0			
333	2020-4-25 8:00	AI	AMRO	0	3	0	0			
334	2020-4-26 8:00	AI	AMRO	0	3	0	0			
335	2020-4-27 8:00	AI	AMRO	0	3	0	0			
336	2020-4-28 8:00	AI	AMRO	0	3	0	0			
337	2020-4-29 8:00	AI	AMRO	0	3	0	0			
338	2020-4-30 8:00	AI	AMRO	0	3	0	0			
339	2020-5-1 8:00	AI	AMRO	0	3	0	0			
340	2020-5-2 8:00	AI	AMRO	0	3	0	0			
341										
342	2020-3-13 8:00	AG	AMRO	1	1	0	0			
343	2020-3-14 8:00	AG	AMRO	0	1	0	0			
344	2020-3-15 8:00	AG	AMRO	0	1	0	0			
345	2020-3-16 8:00	AG	AMRO	0	1	0	0			
346	2020-3-17 8:00	AG	AMRO	0	1	0	0			
347	2020-3-18 8:00	AG	AMRO	0	1	0	0			
348	2020-3-19 8:00	AG	AMRO	0	1	0	0			
349	2020-3-20 8:00	AG	AMRO	0	1	0	0			
350	2020-3-21 8:00	AG	AMRO	0	1	0	0			
351	2020-3-22 8:00	AG	AMRO	0	1	0	0			
352	2020-3-23 8:00	AG	AMRO	0	1	0	0			
353	2020-3-24 8:00	AG	AMRO	2	3	0	0			
354	2020-3-25 8:00	AG	AMRO	0	3	0	0			
355	2020-3-26 8:00	AG	AMRO	0	3	0	0			
356	2020-3-27 8:00	AG	AMRO	0	3	0	0			
357	2020-3-28 8:00	AG	AMRO	4	7	0	0			
358	2020-3-29 8:00	AG	AMRO	0	7	0	0			
359	2020-3-30 8:00	AG	AMRO	0	7	0	0			
360	2020-3-31 8:00	AG	AMRO	0	7	0	0			

Table S1 Daily confirmed cases and daily deaths of COVID-19 in 214 nations from WHO

ID	Date and time	Country or region	Continent	Confirmed patients	Cumulative Confirmed patients	Daily dead patients	Daily cumulative dead patients	Lag days	Daily CFR	3DMA CFR
361	2020-4-1 8:00	AG	AMRO	0	7	0	0			
362	2020-4-2 8:00	AG	AMRO	0	7	0	0			
363	2020-4-3 8:00	AG	AMRO	0	7	0	0			
364	2020-4-4 8:00	AG	AMRO	0	7	0	0			
365	2020-4-5 8:00	AG	AMRO	0	7	0	0			
366	2020-4-6 8:00	AG	AMRO	0	7	0	0			
367	2020-4-7 8:00	AG	AMRO	8	15	0	0			
368	2020-4-8 8:00	AG	AMRO	0	15	0	0			
369	2020-4-9 8:00	AG	AMRO	4	19	2	2	0	10.526	10.526
370	2020-4-10 8:00	AG	AMRO	0	19	0	2	1	10.526	10.526
371	2020-4-11 8:00	AG	AMRO	0	19	0	2	2	10.526	10.192
372	2020-4-12 8:00	AG	AMRO	2	21	0	2	3	9.524	9.858
373	2020-4-13 8:00	AG	AMRO	0	21	0	2	4	9.524	9.248
374	2020-4-14 8:00	AG	AMRO	2	23	0	2	5	8.696	8.972
375	2020-4-15 8:00	AG	AMRO	0	23	0	2	6	8.696	8.696
376	2020-4-16 8:00	AG	AMRO	0	23	0	2	7	8.696	8.696
377	2020-4-17 8:00	AG	AMRO	0	23	0	2	8	8.696	8.696
378	2020-4-18 8:00	AG	AMRO	0	23	0	2	9	8.696	8.696
379	2020-4-19 8:00	AG	AMRO	0	23	0	2	10	8.696	10.145
380	2020-4-20 8:00	AG	AMRO	0	23	1	3	11	13.043	11.594
381	2020-4-21 8:00	AG	AMRO	0	23	0	3	12	13.043	13.043
382	2020-4-22 8:00	AG	AMRO	0	23	0	3	13	13.043	12.862
383	2020-4-23 8:00	AG	AMRO	1	24	0	3	14	12.500	12.681
384	2020-4-24 8:00	AG	AMRO	0	24	0	3	15	12.500	12.500
385	2020-4-25 8:00	AG	AMRO	0	24	0	3	16	12.500	12.500
386	2020-4-26 8:00	AG	AMRO	0	24	0	3	17	12.500	12.500
387	2020-4-27 8:00	AG	AMRO	0	24	0	3	18	12.500	12.500
388	2020-4-28 8:00	AG	AMRO	0	24	0	3	19	12.500	12.500
389	2020-4-29 8:00	AG	AMRO	0	24	0	3	20	12.500	12.500
390	2020-4-30 8:00	AG	AMRO	0	24	0	3	21	12.500	12.500
391	2020-5-1 8:00	AG	AMRO	0	24	0	3	22	12.500	12.500
392	2020-5-2 8:00	AG	AMRO	0	24	0	3	23	12.500	12.500
393										
394	2020-3-3 8:00	AR	AMRO	1	1	0	0			
395	2020-3-4 8:00	AR	AMRO	0	1	0	0			
396	2020-3-5 8:00	AR	AMRO	0	1	0	0			

Table S1 Daily confirmed cases and daily deaths of COVID-19 in 214 nations from WHO

ID	Date and time	Country or region	Continent	Confirmed patients	Cumulative Confirmed patients	Daily dead patients	Daily cumulative dead patients	Lag days	Daily CFR	3DMA CFR
397	2020-3-6 8:00	AR	AMRO	1	2	0	0			
398	2020-3-7 8:00	AR	AMRO	7	9	1	1	0	11.111	9.722
399	2020-3-8 8:00	AR	AMRO	3	12	0	1	1	8.333	9.259
400	2020-3-9 8:00	AR	AMRO	0	12	0	1	2	8.333	8.333
401	2020-3-10 8:00	AR	AMRO	0	12	0	1	3	8.333	7.516
402	2020-3-11 8:00	AR	AMRO	5	17	0	1	4	5.882	6.493
403	2020-3-12 8:00	AR	AMRO	2	19	0	1	5	5.263	4.790
404	2020-3-13 8:00	AR	AMRO	12	31	0	1	6	3.226	4.311
405	2020-3-14 8:00	AR	AMRO	14	45	1	2	7	4.444	4.038
406	2020-3-15 8:00	AR	AMRO	0	45	0	2	8	4.444	4.153
407	2020-3-16 8:00	AR	AMRO	11	56	0	2	9	3.571	3.698
408	2020-3-17 8:00	AR	AMRO	9	65	0	2	10	3.077	2.903
409	2020-3-18 8:00	AR	AMRO	32	97	0	2	11	2.062	2.400
410	2020-3-19 8:00	AR	AMRO	0	97	0	2	12	2.062	2.156
411	2020-3-20 8:00	AR	AMRO	31	128	1	3	13	2.344	2.101
412	2020-3-21 8:00	AR	AMRO	30	158	0	3	14	1.899	2.047
413	2020-3-22 8:00	AR	AMRO	0	158	0	3	15	1.899	1.858
414	2020-3-23 8:00	AR	AMRO	67	225	1	4	16	1.778	1.668
415	2020-3-24 8:00	AR	AMRO	76	301	0	4	17	1.329	1.479
416	2020-3-25 8:00	AR	AMRO	0	301	0	4	18	1.329	1.417
417	2020-3-26 8:00	AR	AMRO	201	502	4	8	19	1.594	1.505
418	2020-3-27 8:00	AR	AMRO	0	502	0	8	20	1.594	1.742
419	2020-3-28 8:00	AR	AMRO	87	589	4	12	21	2.037	2.032
420	2020-3-29 8:00	AR	AMRO	101	690	5	17	22	2.464	2.350
421	2020-3-30 8:00	AR	AMRO	55	745	2	19	23	2.550	2.484
422	2020-3-31 8:00	AR	AMRO	75	820	1	20	24	2.439	2.491
423	2020-4-1 8:00	AR	AMRO	146	966	4	24	25	2.484	2.495
424	2020-4-2 8:00	AR	AMRO	88	1054	3	27	26	2.562	2.594
425	2020-4-3 8:00	AR	AMRO	79	1133	4	31	27	2.736	2.741
426	2020-4-4 8:00	AR	AMRO	132	1265	6	37	28	2.925	2.922
427	2020-4-5 8:00	AR	AMRO	88	1353	5	42	29	3.104	3.021
428	2020-4-6 8:00	AR	AMRO	98	1451	2	44	30	3.032	3.032
429	2020-4-7 8:00	AR	AMRO	103	1554	2	46	31	2.960	3.083
430	2020-4-8 8:00	AR	AMRO	74	1628	7	53	32	3.256	3.238
431	2020-4-9 8:00	AR	AMRO	87	1715	7	60	33	3.499	3.496
432	2020-4-10 8:00	AR	AMRO	80	1795	7	67	34	3.733	3.776

Table S1 Daily confirmed cases and daily deaths of COVID-19 in 214 nations from WHO

ID	Date and time	Country or region	Continent	Confirmed patients	Cumulative Confirmed patients	Daily dead patients	Daily cumulative dead patients	Lag days	Daily CFR	3DMA CFR
433	2020-4-11 8:00	AR	AMRO	134	1929	12	79	35	4.095	3.993
434	2020-4-12 8:00	AR	AMRO	46	1975	3	82	36	4.152	4.133
435	2020-4-13 8:00	AR	AMRO	0	1975	0	82	37	4.152	4.174
436	2020-4-14 8:00	AR	AMRO	277	2252	13	95	38	4.218	4.231
437	2020-4-15 8:00	AR	AMRO	84	2336	6	101	39	4.324	4.301
438	2020-4-16 8:00	AR	AMRO	141	2477	7	108	40	4.360	4.370
439	2020-4-17 8:00	AR	AMRO	121	2598	7	115	41	4.426	4.438
440	2020-4-18 8:00	AR	AMRO	96	2694	7	122	42	4.529	4.530
441	2020-4-19 8:00	AR	AMRO	90	2784	7	129	43	4.634	4.604
442	2020-4-20 8:00	AR	AMRO	55	2839	3	132	44	4.650	4.626
443	2020-4-21 8:00	AR	AMRO	121	2960	4	136	45	4.595	4.654
444	2020-4-22 8:00	AR	AMRO	113	3073	9	145	46	4.719	4.689
445	2020-4-23 8:00	AR	AMRO	124	3197	7	152	47	4.754	4.744
446	2020-4-24 8:00	AR	AMRO	143	3340	7	159	48	4.760	4.772
447	2020-4-25 8:00	AR	AMRO	139	3479	8	167	49	4.800	4.799
448	2020-4-26 8:00	AR	AMRO	222	3701	12	179	50	4.837	4.767
449	2020-4-27 8:00	AR	AMRO	137	3838	0	179	51	4.664	4.811
450	2020-4-28 8:00	AR	AMRO	54	3892	13	192	52	4.933	4.833
451	2020-4-29 8:00	AR	AMRO	127	4019	5	197	53	4.902	4.921
452	2020-4-30 8:00	AR	AMRO	182	4201	10	207	54	4.927	4.941
453	2020-5-1 8:00	AR	AMRO	103	4304	8	215	55	4.995	4.946
454	2020-5-2 8:00	AR	AMRO	172	4476	5	220	56	4.915	4.955
455										
456	2020-3-1 8:00	AM	EURO	1	1	0	0			
457	2020-3-2 8:00	AM	EURO	0	1	0	0			
458	2020-3-3 8:00	AM	EURO	0	1	0	0			
459	2020-3-4 8:00	AM	EURO	0	1	0	0			
460	2020-3-5 8:00	AM	EURO	0	1	0	0			
461	2020-3-6 8:00	AM	EURO	0	1	0	0			
462	2020-3-7 8:00	AM	EURO	0	1	0	0			
463	2020-3-8 8:00	AM	EURO	0	1	0	0			
464	2020-3-9 8:00	AM	EURO	0	1	0	0			
465	2020-3-10 8:00	AM	EURO	0	1	0	0			
466	2020-3-11 8:00	AM	EURO	0	1	0	0			
467	2020-3-12 8:00	AM	EURO	0	1	0	0			
468	2020-3-13 8:00	AM	EURO	7	8	0	0			

Table S1 Daily confirmed cases and daily deaths of COVID-19 in 214 nations from WHO

ID	Date and time	Country or region	Continent	Confirmed patients	Cumulative Confirmed patients	Daily dead patients	Daily cumulative dead patients	Lag days	Daily CFR	3DMA CFR
469	2020-3-14 8:00	AM	EURO	0	8	0	0			
470	2020-3-15 8:00	AM	EURO	18	26	0	0			
471	2020-3-16 8:00	AM	EURO	26	52	0	0			
472	2020-3-17 8:00	AM	EURO	0	52	0	0			
473	2020-3-18 8:00	AM	EURO	32	84	0	0			
474	2020-3-19 8:00	AM	EURO	38	122	0	0			
475	2020-3-20 8:00	AM	EURO	14	136	0	0			
476	2020-3-21 8:00	AM	EURO	24	160	0	0			
477	2020-3-22 8:00	AM	EURO	0	160	0	0			
478	2020-3-23 8:00	AM	EURO	30	190	0	0			
479	2020-3-24 8:00	AM	EURO	45	235	0	0			
480	2020-3-25 8:00	AM	EURO	30	265	0	0			
481	2020-3-26 8:00	AM	EURO	25	290	0	0			
482	2020-3-27 8:00	AM	EURO	39	329	1	1	0	0.304	0.286
483	2020-3-28 8:00	AM	EURO	43	372	0	1	1	0.269	0.427
484	2020-3-29 8:00	AM	EURO	52	424	2	3	2	0.708	0.533
485	2020-3-30 8:00	AM	EURO	58	482	0	3	3	0.622	0.651
486	2020-3-31 8:00	AM	EURO	0	482	0	3	4	0.622	0.603
487	2020-4-1 8:00	AM	EURO	50	532	0	3	5	0.564	0.571
488	2020-4-2 8:00	AM	EURO	39	571	0	3	6	0.525	0.571
489	2020-4-3 8:00	AM	EURO	69	640	1	4	7	0.625	0.700
490	2020-4-4 8:00	AM	EURO	96	736	3	7	8	0.951	0.838
491	2020-4-5 8:00	AM	EURO	10	746	0	7	9	0.938	0.943
492	2020-4-6 8:00	AM	EURO	0	746	0	7	10	0.938	0.946
493	2020-4-7 8:00	AM	EURO	87	833	1	8	11	0.960	0.937
494	2020-4-8 8:00	AM	EURO	44	877	0	8	12	0.912	0.986
495	2020-4-9 8:00	AM	EURO	44	921	2	10	13	1.086	1.057
496	2020-4-10 8:00	AM	EURO	16	937	1	11	14	1.174	1.145
497	2020-4-11 8:00	AM	EURO	0	937	0	11	15	1.174	1.210
498	2020-4-12 8:00	AM	EURO	76	1013	2	13	16	1.283	1.268
499	2020-4-13 8:00	AM	EURO	26	1039	1	14	17	1.347	1.326
500	2020-4-14 8:00	AM	EURO	0	1039	0	14	18	1.347	1.398
501	2020-4-15 8:00	AM	EURO	28	1067	2	16	19	1.500	1.478
502	2020-4-16 8:00	AM	EURO	68	1135	2	18	20	1.586	1.546
503	2020-4-17 8:00	AM	EURO	24	1159	0	18	21	1.553	1.574
504	2020-4-18 8:00	AM	EURO	42	1201	1	19	22	1.582	1.579

Table S1 Daily confirmed cases and daily deaths of COVID-19 in 214 nations from WHO

ID	Date and time	Country or region	Continent	Confirmed patients	Cumulative Confirmed patients	Daily dead patients	Daily cumulative dead patients	Lag days	Daily CFR	3DMA CFR
505	2020-4-19 8:00	AM	EURO	47	1248	1	20	23	1.603	1.578
506	2020-4-20 8:00	AM	EURO	43	1291	0	20	24	1.549	1.598
507	2020-4-21 8:00	AM	EURO	48	1339	2	22	25	1.643	1.645
508	2020-4-22 8:00	AM	EURO	38	1377	2	24	26	1.743	1.672
509	2020-4-23 8:00	AM	EURO	96	1473	0	24	27	1.629	1.649
510	2020-4-24 8:00	AM	EURO	50	1523	0	24	28	1.576	1.632
511	2020-4-25 8:00	AM	EURO	73	1596	3	27	29	1.692	1.624
512	2020-4-26 8:00	AM	EURO	150	1746	1	28	30	1.604	1.633
513	2020-4-27 8:00	AM	EURO	62	1808	1	29	31	1.604	1.605
514	2020-4-28 8:00	AM	EURO	59	1867	1	30	32	1.607	1.588
515	2020-4-29 8:00	AM	EURO	65	1932	0	30	33	1.553	1.570
516	2020-4-30 8:00	AM	EURO	134	2066	2	32	34	1.549	1.546
517	2020-5-1 8:00	AM	EURO	82	2148	1	33	35	1.536	1.512
518	2020-5-2 8:00	AM	EURO	125	2273	0	33	36	1.452	1.494
519										
520	2020-3-17 8:00	AW	AMRO	2	2	0	0			
521	2020-3-18 8:00	AW	AMRO	2	4	0	0			
522	2020-3-19 8:00	AW	AMRO	1	5	0	0			
523	2020-3-20 8:00	AW	AMRO	0	5	0	0			
524	2020-3-21 8:00	AW	AMRO	0	5	0	0			
525	2020-3-22 8:00	AW	AMRO	0	5	0	0			
526	2020-3-23 8:00	AW	AMRO	3	8	0	0			
527	2020-3-24 8:00	AW	AMRO	4	12	0	0			
528	2020-3-25 8:00	AW	AMRO	0	12	0	0			
529	2020-3-26 8:00	AW	AMRO	7	19	0	0			
530	2020-3-27 8:00	AW	AMRO	0	19	0	0			
531	2020-3-28 8:00	AW	AMRO	9	28	0	0			
532	2020-3-29 8:00	AW	AMRO	18	46	0	0			
533	2020-3-30 8:00	AW	AMRO	0	46	0	0			
534	2020-3-31 8:00	AW	AMRO	4	50	0	0			
535	2020-4-1 8:00	AW	AMRO	5	55	0	0			
536	2020-4-2 8:00	AW	AMRO	0	55	0	0			
537	2020-4-3 8:00	AW	AMRO	5	60	0	0			
538	2020-4-4 8:00	AW	AMRO	2	62	0	0			
539	2020-4-5 8:00	AW	AMRO	2	64	0	0			
540	2020-4-6 8:00	AW	AMRO	0	64	0	0			

Table S1 Daily confirmed cases and daily deaths of COVID-19 in 214 nations from WHO

ID	Date and time	Country or region	Continent	Confirmed patients	Cumulative Confirmed patients	Daily dead patients	Daily cumulative dead patients	Lag days	Daily CFR	3DMA CFR
541	2020-4-7 8:00	AW	AMRO	0	64	0	0			
542	2020-4-8 8:00	AW	AMRO	7	71	0	0			
543	2020-4-9 8:00	AW	AMRO	3	74	0	0			
544	2020-4-10 8:00	AW	AMRO	8	82	0	0			
545	2020-4-11 8:00	AW	AMRO	4	86	0	0			
546	2020-4-12 8:00	AW	AMRO	6	92	0	0			
547	2020-4-13 8:00	AW	AMRO	0	92	0	0			
548	2020-4-14 8:00	AW	AMRO	0	92	0	0			
549	2020-4-15 8:00	AW	AMRO	0	92	0	0			
550	2020-4-16 8:00	AW	AMRO	1	93	1	1	0	1.075	1.590
551	2020-4-17 8:00	AW	AMRO	2	95	1	2	1	2.105	1.755
552	2020-4-18 8:00	AW	AMRO	1	96	0	2	2	2.083	2.091
553	2020-4-19 8:00	AW	AMRO	0	96	0	2	3	2.083	2.076
554	2020-4-20 8:00	AW	AMRO	1	97	0	2	4	2.062	2.069
555	2020-4-21 8:00	AW	AMRO	0	97	0	2	5	2.062	2.062
556	2020-4-22 8:00	AW	AMRO	0	97	0	2	6	2.062	2.041
557	2020-4-23 8:00	AW	AMRO	3	100	0	2	7	2.000	2.021
558	2020-4-24 8:00	AW	AMRO	0	100	0	2	8	2.000	2.000
559	2020-4-25 8:00	AW	AMRO	0	100	0	2	9	2.000	2.000
560	2020-4-26 8:00	AW	AMRO	0	100	0	2	10	2.000	2.000
561	2020-4-27 8:00	AW	AMRO	0	100	0	2	11	2.000	2.000
562	2020-4-28 8:00	AW	AMRO	0	100	0	2	12	2.000	2.000
563	2020-4-29 8:00	AW	AMRO	0	100	0	2	13	2.000	2.000
564	2020-4-30 8:00	AW	AMRO	0	100	0	2	14	2.000	2.000
565	2020-5-1 8:00	AW	AMRO	0	100	0	2	15	2.000	2.000
566	2020-5-2 8:00	AW	AMRO	0	100	0	2	16	2.000	2.000
567										
568	2020-1-25 8:00	AU	WPRO	3	3	0	0			
569	2020-1-26 8:00	AU	WPRO	1	4	0	0			
570	2020-1-27 8:00	AU	WPRO	1	5	0	0			
571	2020-1-28 8:00	AU	WPRO	0	5	0	0			
572	2020-1-29 8:00	AU	WPRO	2	7	0	0			
573	2020-1-30 8:00	AU	WPRO	2	9	0	0			
574	2020-1-31 8:00	AU	WPRO	0	9	0	0			
575	2020-2-1 8:00	AU	WPRO	3	12	0	0			
576	2020-2-2 8:00	AU	WPRO	0	12	0	0			

Table S1 Daily confirmed cases and daily deaths of COVID-19 in 214 nations from WHO

ID	Date and time	Country or region	Continent	Confirmed patients	Cumulative Confirmed patients	Daily dead patients	Daily cumulative dead patients	Lag days	Daily CFR	3DMA CFR
577	2020-2-3 8:00	AU	WPRO	0	12	0	0			
578	2020-2-4 8:00	AU	WPRO	1	13	0	0			
579	2020-2-5 8:00	AU	WPRO	1	14	0	0			
580	2020-2-6 8:00	AU	WPRO	1	15	0	0			
581	2020-2-7 8:00	AU	WPRO	0	15	0	0			
582	2020-2-8 8:00	AU	WPRO	0	15	0	0			
583	2020-2-9 8:00	AU	WPRO	0	15	0	0			
584	2020-2-10 8:00	AU	WPRO	0	15	0	0			
585	2020-2-11 8:00	AU	WPRO	0	15	0	0			
586	2020-2-12 8:00	AU	WPRO	0	15	0	0			
587	2020-2-13 8:00	AU	WPRO	0	15	0	0			
588	2020-2-14 8:00	AU	WPRO	0	15	0	0			
589	2020-2-15 8:00	AU	WPRO	0	15	0	0			
590	2020-2-16 8:00	AU	WPRO	0	15	0	0			
591	2020-2-17 8:00	AU	WPRO	0	15	0	0			
592	2020-2-18 8:00	AU	WPRO	0	15	0	0			
593	2020-2-19 8:00	AU	WPRO	0	15	0	0			
594	2020-2-20 8:00	AU	WPRO	0	15	0	0			
595	2020-2-21 8:00	AU	WPRO	2	17	0	0			
596	2020-2-22 8:00	AU	WPRO	4	21	0	0			
597	2020-2-23 8:00	AU	WPRO	1	22	0	0			
598	2020-2-24 8:00	AU	WPRO	0	22	0	0			
599	2020-2-25 8:00	AU	WPRO	1	23	0	0			
600	2020-2-26 8:00	AU	WPRO	0	23	0	0			
601	2020-2-27 8:00	AU	WPRO	0	23	0	0			
602	2020-2-28 8:00	AU	WPRO	0	23	0	0			
603	2020-2-29 8:00	AU	WPRO	2	25	0	0			
604	2020-3-1 8:00	AU	WPRO	2	27	1	1	0	3.704	3.367
605	2020-3-2 8:00	AU	WPRO	6	33	0	1	1	3.030	3.255
606	2020-3-3 8:00	AU	WPRO	0	33	0	1	2	3.030	3.030
607	2020-3-4 8:00	AU	WPRO	0	33	0	1	3	3.030	3.190
608	2020-3-5 8:00	AU	WPRO	24	57	1	2	4	3.509	3.255
609	2020-3-6 8:00	AU	WPRO	5	62	0	2	5	3.226	3.197
610	2020-3-7 8:00	AU	WPRO	8	70	0	2	6	2.857	3.326
611	2020-3-8 8:00	AU	WPRO	7	77	1	3	7	3.896	3.338
612	2020-3-9 8:00	AU	WPRO	15	92	0	3	8	3.261	3.473

Table S1 Daily confirmed cases and daily deaths of COVID-19 in 214 nations from WHO

ID	Date and time	Country or region	Continent	Confirmed patients	Cumulative Confirmed patients	Daily dead patients	Daily cumulative dead patients	Lag days	Daily CFR	3DMA CFR
613	2020-3-10 8:00	AU	WPRO	0	92	0	3	9	3.261	2.994
614	2020-3-11 8:00	AU	WPRO	30	122	0	3	10	2.459	2.621
615	2020-3-12 8:00	AU	WPRO	18	140	0	3	11	2.143	2.063
616	2020-3-13 8:00	AU	WPRO	49	189	0	3	12	1.587	1.751
617	2020-3-14 8:00	AU	WPRO	8	197	0	3	13	1.523	1.596
618	2020-3-15 8:00	AU	WPRO	101	298	2	5	14	1.678	1.563
619	2020-3-16 8:00	AU	WPRO	38	336	0	5	15	1.488	1.458
620	2020-3-17 8:00	AU	WPRO	78	414	0	5	16	1.208	1.291
621	2020-3-18 8:00	AU	WPRO	96	510	1	6	17	1.176	1.077
622	2020-3-19 8:00	AU	WPRO	199	709	0	6	18	0.846	0.942
623	2020-3-20 8:00	AU	WPRO	164	873	1	7	19	0.802	0.765
624	2020-3-21 8:00	AU	WPRO	208	1081	0	7	20	0.648	0.696
625	2020-3-22 8:00	AU	WPRO	17	1098	0	7	21	0.638	0.565
626	2020-3-23 8:00	AU	WPRO	611	1709	0	7	22	0.410	0.474
627	2020-3-24 8:00	AU	WPRO	427	2136	1	8	23	0.375	0.371
628	2020-3-25 8:00	AU	WPRO	287	2423	0	8	24	0.330	0.366
629	2020-3-26 8:00	AU	WPRO	376	2799	3	11	25	0.393	0.386
630	2020-3-27 8:00	AU	WPRO	186	2985	2	13	26	0.436	0.405
631	2020-3-28 8:00	AU	WPRO	650	3635	1	14	27	0.385	0.408
632	2020-3-29 8:00	AU	WPRO	331	3966	2	16	28	0.403	0.404
633	2020-3-30 8:00	AU	WPRO	279	4245	2	18	29	0.424	0.415
634	2020-3-31 8:00	AU	WPRO	312	4557	1	19	30	0.417	0.417
635	2020-4-1 8:00	AU	WPRO	303	4860	1	20	31	0.412	0.417
636	2020-4-2 8:00	AU	WPRO	116	4976	1	21	32	0.422	0.440
637	2020-4-3 8:00	AU	WPRO	374	5350	5	26	33	0.486	0.474
638	2020-4-4 8:00	AU	WPRO	104	5454	2	28	34	0.513	0.534
639	2020-4-5 8:00	AU	WPRO	181	5635	6	34	35	0.603	0.597
640	2020-4-6 8:00	AU	WPRO	160	5795	5	39	36	0.673	0.665
641	2020-4-7 8:00	AU	WPRO	49	5844	3	42	37	0.719	0.716
642	2020-4-8 8:00	AU	WPRO	112	5956	3	45	38	0.756	0.767
643	2020-4-9 8:00	AU	WPRO	96	6052	5	50	39	0.826	0.809
644	2020-4-10 8:00	AU	WPRO	100	6152	2	52	40	0.845	0.854
645	2020-4-11 8:00	AU	WPRO	137	6289	4	56	41	0.890	0.890
646	2020-4-12 8:00	AU	WPRO	24	6313	3	59	42	0.935	0.930
647	2020-4-13 8:00	AU	WPRO	9	6322	2	61	43	0.965	0.953
648	2020-4-14 8:00	AU	WPRO	44	6366	0	61	44	0.958	0.958

Table S1 Daily confirmed cases and daily deaths of COVID-19 in 214 nations from WHO

ID	Date and time	Country or region	Continent	Confirmed patients	Cumulative Confirmed patients	Daily dead patients	Daily cumulative dead patients	Lag days	Daily CFR	3DMA CFR
649	2020-4-15 8:00	AU	WPRO	50	6416	0	61	45	0.951	0.961
650	2020-4-16 8:00	AU	WPRO	42	6458	2	63	46	0.976	0.967
651	2020-4-17 8:00	AU	WPRO	10	6468	0	63	47	0.974	0.992
652	2020-4-18 8:00	AU	WPRO	65	6533	4	67	48	1.026	1.020
653	2020-4-19 8:00	AU	WPRO	73	6606	3	70	49	1.060	1.048
654	2020-4-20 8:00	AU	WPRO	6	6612	0	70	50	1.059	1.063
655	2020-4-21 8:00	AU	WPRO	13	6625	1	71	51	1.072	1.081
656	2020-4-22 8:00	AU	WPRO	22	6647	3	74	52	1.113	1.104
657	2020-4-23 8:00	AU	WPRO	14	6661	1	75	53	1.126	1.126
658	2020-4-24 8:00	AU	WPRO	6	6667	1	76	54	1.140	1.149
659	2020-4-25 8:00	AU	WPRO	20	6687	3	79	55	1.181	1.177
660	2020-4-26 8:00	AU	WPRO	16	6703	2	81	56	1.208	1.209
661	2020-4-27 8:00	AU	WPRO	10	6713	2	83	57	1.236	1.231
662	2020-4-28 8:00	AU	WPRO	12	6725	1	84	58	1.249	1.264
663	2020-4-29 8:00	AU	WPRO	13	6738	4	88	59	1.306	1.296
664	2020-4-30 8:00	AU	WPRO	8	6746	2	90	60	1.334	1.334
665	2020-5-1 8:00	AU	WPRO	16	6762	2	92	61	1.361	1.356
666	2020-5-2 8:00	AU	WPRO	5	6767	1	93	62	1.374	1.367
667										
668	2020-2-25 8:00	AT	EURO	2	2	0	0			
669	2020-2-26 8:00	AT	EURO	0	2	0	0			
670	2020-2-27 8:00	AT	EURO	2	4	0	0			
671	2020-2-28 8:00	AT	EURO	1	5	0	0			
672	2020-2-29 8:00	AT	EURO	5	10	0	0			
673	2020-3-1 8:00	AT	EURO	0	10	0	0			
674	2020-3-2 8:00	AT	EURO	8	18	0	0			
675	2020-3-3 8:00	AT	EURO	6	24	0	0			
676	2020-3-4 8:00	AT	EURO	5	29	0	0			
677	2020-3-5 8:00	AT	EURO	8	37	0	0			
678	2020-3-6 8:00	AT	EURO	29	66	0	0			
679	2020-3-7 8:00	AT	EURO	13	79	0	0			
680	2020-3-8 8:00	AT	EURO	23	102	0	0			
681	2020-3-9 8:00	AT	EURO	10	112	0	0			
682	2020-3-10 8:00	AT	EURO	70	182	0	0			
683	2020-3-11 8:00	AT	EURO	0	182	0	0			
684	2020-3-12 8:00	AT	EURO	179	361	1	1	0	0.277	0.238

Table S1 Daily confirmed cases and daily deaths of COVID-19 in 214 nations from WHO

ID	Date and time	Country or region	Continent	Confirmed patients	Cumulative Confirmed patients	Daily dead patients	Daily cumulative dead patients	Lag days	Daily CFR	3DMA CFR
685	2020-3-13 8:00	AT	EURO	143	504	0	1	1	0.198	0.209
686	2020-3-14 8:00	AT	EURO	151	655	0	1	2	0.153	0.156
687	2020-3-15 8:00	AT	EURO	205	860	0	1	3	0.116	0.124
688	2020-3-16 8:00	AT	EURO	99	959	0	1	4	0.104	0.149
689	2020-3-17 8:00	AT	EURO	373	1332	2	3	5	0.225	0.191
690	2020-3-18 8:00	AT	EURO	314	1646	1	4	6	0.243	0.247
691	2020-3-19 8:00	AT	EURO	197	1843	1	5	7	0.271	0.247
692	2020-3-20 8:00	AT	EURO	806	2649	1	6	8	0.227	0.254
693	2020-3-21 8:00	AT	EURO	375	3024	2	8	9	0.265	0.252
694	2020-3-22 8:00	AT	EURO	0	3024	0	8	10	0.265	0.323
695	2020-3-23 8:00	AT	EURO	607	3631	8	16	11	0.441	0.421
696	2020-3-24 8:00	AT	EURO	855	4486	9	25	12	0.557	0.522
697	2020-3-25 8:00	AT	EURO	796	5282	5	30	13	0.568	0.568
698	2020-3-26 8:00	AT	EURO	606	5888	4	34	14	0.577	0.628
699	2020-3-27 8:00	AT	EURO	1141	7029	18	52	15	0.740	0.734
700	2020-3-28 8:00	AT	EURO	668	7697	16	68	16	0.883	0.814
701	2020-3-29 8:00	AT	EURO	594	8291	0	68	17	0.820	0.893
702	2020-3-30 8:00	AT	EURO	522	8813	18	86	18	0.976	0.973
703	2020-3-31 8:00	AT	EURO	805	9618	22	108	19	1.123	1.119
704	2020-4-1 8:00	AT	EURO	564	10182	20	128	20	1.257	1.248
705	2020-4-2 8:00	AT	EURO	529	10711	18	146	21	1.363	1.347
706	2020-4-3 8:00	AT	EURO	418	11129	12	158	22	1.420	1.413
707	2020-4-4 8:00	AT	EURO	396	11525	10	168	23	1.458	1.486
708	2020-4-5 8:00	AT	EURO	241	11766	18	186	24	1.581	1.580
709	2020-4-6 8:00	AT	EURO	217	11983	18	204	25	1.702	1.691
710	2020-4-7 8:00	AT	EURO	314	12297	16	220	26	1.789	1.805
711	2020-4-8 8:00	AT	EURO	343	12640	23	243	27	1.922	1.939
712	2020-4-9 8:00	AT	EURO	329	12969	30	273	28	2.105	2.085
713	2020-4-10 8:00	AT	EURO	279	13248	22	295	29	2.227	2.228
714	2020-4-11 8:00	AT	EURO	312	13560	24	319	30	2.353	2.340
715	2020-4-12 8:00	AT	EURO	247	13807	18	337	31	2.441	2.435
716	2020-4-13 8:00	AT	EURO	130	13937	13	350	32	2.511	2.524
717	2020-4-14 8:00	AT	EURO	106	14043	18	368	33	2.621	2.610
718	2020-4-15 8:00	AT	EURO	191	14234	16	384	34	2.698	2.684
719	2020-4-16 8:00	AT	EURO	136	14370	9	393	35	2.735	2.757
720	2020-4-17 8:00	AT	EURO	78	14448	17	410	36	2.838	2.841

Table S1 Daily confirmed cases and daily deaths of COVID-19 in 214 nations from WHO

ID	Date and time	Country or region	Continent	Confirmed patients	Cumulative Confirmed patients	Daily dead patients	Daily cumulative dead patients	Lag days	Daily CFR	3DMA CFR
721	2020-4-18 8:00	AT	EURO	155	14603	21	431	37	2.951	2.937
722	2020-4-19 8:00	AT	EURO	59	14662	12	443	38	3.021	3.015
723	2020-4-20 8:00	AT	EURO	48	14710	9	452	39	3.073	3.073
724	2020-4-21 8:00	AT	EURO	73	14783	10	462	40	3.125	3.106
725	2020-4-22 8:00	AT	EURO	50	14833	1	463	41	3.121	3.186
726	2020-4-23 8:00	AT	EURO	91	14924	31	494	42	3.310	3.274
727	2020-4-24 8:00	AT	EURO	61	14985	14	508	43	3.390	3.368
728	2020-4-25 8:00	AT	EURO	83	15068	5	513	44	3.405	3.445
729	2020-4-26 8:00	AT	EURO	66	15134	23	536	45	3.542	3.505
730	2020-4-27 8:00	AT	EURO	55	15189	6	542	46	3.568	3.570
731	2020-4-28 8:00	AT	EURO	67	15256	7	549	47	3.599	3.628
732	2020-4-29 8:00	AT	EURO	58	15314	20	569	48	3.716	3.696
733	2020-4-30 8:00	AT	EURO	50	15364	11	580	49	3.775	3.756
734	2020-5-1 8:00	AT	EURO	93	15457	4	584	50	3.778	3.782
735	2020-5-2 8:00	AT	EURO	74	15531	5	589	51	3.792	3.785
736										
737	2020-2-29 8:00	AZ	EURO	3	3	0	0			
738	2020-3-1 8:00	AZ	EURO	0	3	0	0			
739	2020-3-2 8:00	AZ	EURO	0	3	0	0			
740	2020-3-3 8:00	AZ	EURO	0	3	0	0			
741	2020-3-4 8:00	AZ	EURO	0	3	0	0			
742	2020-3-5 8:00	AZ	EURO	0	3	0	0			
743	2020-3-6 8:00	AZ	EURO	6	9	0	0			
744	2020-3-7 8:00	AZ	EURO	0	9	0	0			
745	2020-3-8 8:00	AZ	EURO	0	9	0	0			
746	2020-3-9 8:00	AZ	EURO	0	9	0	0			
747	2020-3-10 8:00	AZ	EURO	0	9	0	0			
748	2020-3-11 8:00	AZ	EURO	0	9	0	0			
749	2020-3-12 8:00	AZ	EURO	2	11	0	0			
750	2020-3-13 8:00	AZ	EURO	0	11	0	0			
751	2020-3-14 8:00	AZ	EURO	8	19	0	0			
752	2020-3-15 8:00	AZ	EURO	2	21	0	0			
753	2020-3-16 8:00	AZ	EURO	0	21	0	0			
754	2020-3-17 8:00	AZ	EURO	0	21	0	0			
755	2020-3-18 8:00	AZ	EURO	13	34	1	1	0	2.941	2.941
756	2020-3-19 8:00	AZ	EURO	0	34	0	1	1	2.941	2.718

Table S1 Daily confirmed cases and daily deaths of COVID-19 in 214 nations from WHO

ID	Date and time	Country or region	Continent	Confirmed patients	Cumulative Confirmed patients	Daily dead patients	Daily cumulative dead patients	Lag days	Daily CFR	3DMA CFR
757	2020-3-20 8:00	AZ	EURO	10	44	0	1	2	2.273	2.367
758	2020-3-21 8:00	AZ	EURO	9	53	0	1	3	1.887	2.015
759	2020-3-22 8:00	AZ	EURO	0	53	0	1	4	1.887	1.771
760	2020-3-23 8:00	AZ	EURO	12	65	0	1	5	1.538	1.605
761	2020-3-24 8:00	AZ	EURO	7	72	0	1	6	1.389	1.359
762	2020-3-25 8:00	AZ	EURO	15	87	0	1	7	1.149	1.563
763	2020-3-26 8:00	AZ	EURO	6	93	1	2	8	2.151	1.920
764	2020-3-27 8:00	AZ	EURO	29	122	1	3	9	2.459	2.217
765	2020-3-28 8:00	AZ	EURO	25	147	0	3	10	2.041	2.313
766	2020-3-29 8:00	AZ	EURO	17	164	1	4	11	2.439	2.195
767	2020-3-30 8:00	AZ	EURO	26	190	0	4	12	2.105	2.003
768	2020-3-31 8:00	AZ	EURO	83	273	0	4	13	1.465	1.749
769	2020-4-1 8:00	AZ	EURO	25	298	1	5	14	1.678	1.512
770	2020-4-2 8:00	AZ	EURO	61	359	0	5	15	1.393	1.440
771	2020-4-3 8:00	AZ	EURO	41	400	0	5	16	1.250	1.257
772	2020-4-4 8:00	AZ	EURO	43	443	0	5	17	1.129	1.118
773	2020-4-5 8:00	AZ	EURO	69	512	0	5	18	0.977	0.987
774	2020-4-6 8:00	AZ	EURO	72	584	0	5	19	0.856	0.975
775	2020-4-7 8:00	AZ	EURO	57	641	2	7	20	1.092	1.021
776	2020-4-8 8:00	AZ	EURO	76	717	1	8	21	1.116	1.060
777	2020-4-9 8:00	AZ	EURO	105	822	0	8	22	0.973	1.020
778	2020-4-10 8:00	AZ	EURO	104	926	1	9	23	0.972	0.985
779	2020-4-11 8:00	AZ	EURO	65	991	1	10	24	1.009	1.007
780	2020-4-12 8:00	AZ	EURO	67	1058	1	11	25	1.040	1.017
781	2020-4-13 8:00	AZ	EURO	40	1098	0	11	26	1.002	1.029
782	2020-4-14 8:00	AZ	EURO	50	1148	1	12	27	1.045	1.044
783	2020-4-15 8:00	AZ	EURO	49	1197	1	13	28	1.086	1.056
784	2020-4-16 8:00	AZ	EURO	56	1253	0	13	29	1.038	1.098
785	2020-4-17 8:00	AZ	EURO	30	1283	2	15	30	1.169	1.109
786	2020-4-18 8:00	AZ	EURO	57	1340	0	15	31	1.119	1.200
787	2020-4-19 8:00	AZ	EURO	33	1373	3	18	32	1.311	1.263
788	2020-4-20 8:00	AZ	EURO	25	1398	1	19	33	1.359	1.331
789	2020-4-21 8:00	AZ	EURO	38	1436	0	19	34	1.323	1.345
790	2020-4-22 8:00	AZ	EURO	44	1480	1	20	35	1.351	1.331
791	2020-4-23 8:00	AZ	EURO	38	1518	0	20	36	1.318	1.320
792	2020-4-24 8:00	AZ	EURO	30	1548	0	20	37	1.292	1.310

Table S1 Daily confirmed cases and daily deaths of COVID-19 in 214 nations from WHO

ID	Date and time	Country or region	Continent	Confirmed patients	Cumulative Confirmed patients	Daily dead patients	Daily cumulative dead patients	Lag days	Daily CFR	3DMA CFR
793	2020-4-25 8:00	AZ	EURO	44	1592	1	21	38	1.319	1.303
794	2020-4-26 8:00	AZ	EURO	25	1617	0	21	39	1.299	1.298
795	2020-4-27 8:00	AZ	EURO	28	1645	0	21	40	1.277	1.295
796	2020-4-28 8:00	AZ	EURO	33	1678	1	22	41	1.311	1.290
797	2020-4-29 8:00	AZ	EURO	39	1717	0	22	42	1.281	1.298
798	2020-4-30 8:00	AZ	EURO	49	1766	1	23	43	1.302	1.305
799	2020-5-1 8:00	AZ	EURO	38	1804	1	24	44	1.330	1.327
800	2020-5-2 8:00	AZ	EURO	50	1854	1	25	45	1.348	1.339
801										
802	2020-3-17 8:00	BS	AMRO	1	1	0	0			
803	2020-3-18 8:00	BS	AMRO	2	3	0	0			
804	2020-3-19 8:00	BS	AMRO	0	3	0	0			
805	2020-3-20 8:00	BS	AMRO	1	4	0	0			
806	2020-3-21 8:00	BS	AMRO	0	4	0	0			
807	2020-3-22 8:00	BS	AMRO	0	4	0	0			
808	2020-3-23 8:00	BS	AMRO	0	4	0	0			
809	2020-3-24 8:00	BS	AMRO	0	4	0	0			
810	2020-3-25 8:00	BS	AMRO	0	4	0	0			
811	2020-3-26 8:00	BS	AMRO	1	5	0	0			
812	2020-3-27 8:00	BS	AMRO	0	5	0	0			
813	2020-3-28 8:00	BS	AMRO	4	9	0	0			
814	2020-3-29 8:00	BS	AMRO	1	10	0	0			
815	2020-3-30 8:00	BS	AMRO	0	10	0	0			
816	2020-3-31 8:00	BS	AMRO	4	14	0	0			
817	2020-4-1 8:00	BS	AMRO	0	14	0	0			
818	2020-4-2 8:00	BS	AMRO	1	15	0	0			
819	2020-4-3 8:00	BS	AMRO	6	21	1	1	0	4.762	8.631
820	2020-4-4 8:00	BS	AMRO	3	24	2	3	1	12.500	9.921
821	2020-4-5 8:00	BS	AMRO	0	24	0	3	2	12.500	13.095
822	2020-4-6 8:00	BS	AMRO	4	28	1	4	3	14.286	14.676
823	2020-4-7 8:00	BS	AMRO	1	29	1	5	4	17.241	15.560
824	2020-4-8 8:00	BS	AMRO	4	33	0	5	5	15.152	16.353
825	2020-4-9 8:00	BS	AMRO	3	36	1	6	6	16.667	16.439
826	2020-4-10 8:00	BS	AMRO	4	40	1	7	7	17.500	17.893
827	2020-4-11 8:00	BS	AMRO	1	41	1	8	8	19.512	18.687
828	2020-4-12 8:00	BS	AMRO	1	42	0	8	9	19.048	19.202

Table S1 Daily confirmed cases and daily deaths of COVID-19 in 214 nations from WHO

ID	Date and time	Country or region	Continent	Confirmed patients	Cumulative Confirmed patients	Daily dead patients	Daily cumulative dead patients	Lag days	Daily CFR	3DMA CFR
829	2020-4-13 8:00	BS	AMRO	0	42	0	8	10	19.048	18.372
830	2020-4-14 8:00	BS	AMRO	5	47	0	8	11	17.021	17.465
831	2020-4-15 8:00	BS	AMRO	2	49	0	8	12	16.327	16.558
832	2020-4-16 8:00	BS	AMRO	0	49	0	8	13	16.327	15.916
833	2020-4-17 8:00	BS	AMRO	4	53	0	8	14	15.094	16.029
834	2020-4-18 8:00	BS	AMRO	1	54	1	9	15	16.667	16.143
835	2020-4-19 8:00	BS	AMRO	0	54	0	9	16	16.667	16.284
836	2020-4-20 8:00	BS	AMRO	4	58	0	9	17	15.517	15.728
837	2020-4-21 8:00	BS	AMRO	2	60	0	9	18	15.000	14.860
838	2020-4-22 8:00	BS	AMRO	4	64	0	9	19	14.063	14.303
839	2020-4-23 8:00	BS	AMRO	1	65	0	9	20	13.846	13.589
840	2020-4-24 8:00	BS	AMRO	5	70	0	9	21	12.857	13.994
841	2020-4-25 8:00	BS	AMRO	2	72	2	11	22	15.278	14.471
842	2020-4-26 8:00	BS	AMRO	0	72	0	11	23	15.278	14.886
843	2020-4-27 8:00	BS	AMRO	6	78	0	11	24	14.103	14.377
844	2020-4-28 8:00	BS	AMRO	2	80	0	11	25	13.750	13.868
845	2020-4-29 8:00	BS	AMRO	0	80	0	11	26	13.750	13.750
846	2020-4-30 8:00	BS	AMRO	0	80	0	11	27	13.750	13.750
847	2020-5-1 8:00	BS	AMRO	0	80	0	11	28	13.750	13.693
848	2020-5-2 8:00	BS	AMRO	1	81	0	11	29	13.580	13.665
849										
850	2020-2-24 8:00	BH	EMRO	2	2	0	0			
851	2020-2-25 8:00	BH	EMRO	15	17	0	0			
852	2020-2-26 8:00	BH	EMRO	16	33	0	0			
853	2020-2-27 8:00	BH	EMRO	0	33	0	0			
854	2020-2-28 8:00	BH	EMRO	5	38	0	0			
855	2020-2-29 8:00	BH	EMRO	2	40	0	0			
856	2020-3-1 8:00	BH	EMRO	0	40	0	0			
857	2020-3-2 8:00	BH	EMRO	9	49	0	0			
858	2020-3-3 8:00	BH	EMRO	0	49	0	0			
859	2020-3-4 8:00	BH	EMRO	0	49	0	0			
860	2020-3-5 8:00	BH	EMRO	0	49	0	0			
861	2020-3-6 8:00	BH	EMRO	0	49	0	0			
862	2020-3-7 8:00	BH	EMRO	7	56	0	0			
863	2020-3-8 8:00	BH	EMRO	23	79	0	0			
864	2020-3-9 8:00	BH	EMRO	16	95	0	0			

Table S1 Daily confirmed cases and daily deaths of COVID-19 in 214 nations from WHO

ID	Date and time	Country or region	Continent	Confirmed patients	Cumulative Confirmed patients	Daily dead patients	Daily cumulative dead patients	Lag days	Daily CFR	3DMA CFR
865	2020-3-10 8:00	BH	EMRO	15	110	0	0			
866	2020-3-11 8:00	BH	EMRO	79	189	0	0			
867	2020-3-12 8:00	BH	EMRO	6	195	0	0			
868	2020-3-13 8:00	BH	EMRO	0	195	0	0			
869	2020-3-14 8:00	BH	EMRO	16	211	0	0			
870	2020-3-15 8:00	BH	EMRO	3	214	0	0			
871	2020-3-16 8:00	BH	EMRO	7	221	1	1	0	0.452	0.437
872	2020-3-17 8:00	BH	EMRO	16	237	0	1	1	0.422	0.422
873	2020-3-18 8:00	BH	EMRO	19	256	0	1	2	0.391	0.395
874	2020-3-19 8:00	BH	EMRO	13	269	0	1	3	0.372	0.371
875	2020-3-20 8:00	BH	EMRO	16	285	0	1	4	0.351	0.350
876	2020-3-21 8:00	BH	EMRO	21	306	0	1	5	0.327	0.425
877	2020-3-22 8:00	BH	EMRO	28	334	1	2	6	0.599	0.505
878	2020-3-23 8:00	BH	EMRO	5	339	0	2	7	0.590	0.653
879	2020-3-24 8:00	BH	EMRO	51	390	1	3	8	0.769	0.692
880	2020-3-25 8:00	BH	EMRO	29	419	0	3	9	0.716	0.787
881	2020-3-26 8:00	BH	EMRO	38	457	1	4	10	0.875	0.817
882	2020-3-27 8:00	BH	EMRO	9	466	0	4	11	0.858	0.860
883	2020-3-28 8:00	BH	EMRO	7	473	0	4	12	0.846	0.835
884	2020-3-29 8:00	BH	EMRO	26	499	0	4	13	0.802	0.808
885	2020-3-30 8:00	BH	EMRO	16	515	0	4	14	0.777	0.761
886	2020-3-31 8:00	BH	EMRO	52	567	0	4	15	0.705	0.728
887	2020-4-1 8:00	BH	EMRO	2	569	0	4	16	0.703	0.679
888	2020-4-2 8:00	BH	EMRO	66	635	0	4	17	0.630	0.643
889	2020-4-3 8:00	BH	EMRO	37	672	0	4	18	0.595	0.602
890	2020-4-4 8:00	BH	EMRO	16	688	0	4	19	0.581	0.583
891	2020-4-5 8:00	BH	EMRO	10	698	0	4	20	0.573	0.569
892	2020-4-6 8:00	BH	EMRO	25	723	0	4	21	0.553	0.540
893	2020-4-7 8:00	BH	EMRO	88	811	0	4	22	0.493	0.552
894	2020-4-8 8:00	BH	EMRO	10	821	1	5	23	0.609	0.562
895	2020-4-9 8:00	BH	EMRO	34	855	0	5	24	0.585	0.617
896	2020-4-10 8:00	BH	EMRO	58	913	1	6	25	0.657	0.611
897	2020-4-11 8:00	BH	EMRO	103	1016	0	6	26	0.591	0.600
898	2020-4-12 8:00	BH	EMRO	71	1087	0	6	27	0.552	0.529
899	2020-4-13 8:00	BH	EMRO	261	1348	0	6	28	0.445	0.485
900	2020-4-14 8:00	BH	EMRO	180	1528	1	7	29	0.458	0.441

Table S1 Daily confirmed cases and daily deaths of COVID-19 in 214 nations from WHO

ID	Date and time	Country or region	Continent	Confirmed patients	Cumulative Confirmed patients	Daily dead patients	Daily cumulative dead patients	Lag days	Daily CFR	3DMA CFR
901	2020-4-15 8:00	BH	EMRO	143	1671	0	7	30	0.419	0.430
902	2020-4-16 8:00	BH	EMRO	27	1698	0	7	31	0.412	0.411
903	2020-4-17 8:00	BH	EMRO	42	1740	0	7	32	0.402	0.404
904	2020-4-18 8:00	BH	EMRO	27	1767	0	7	33	0.396	0.391
905	2020-4-19 8:00	BH	EMRO	106	1873	0	7	34	0.374	0.380
906	2020-4-20 8:00	BH	EMRO	22	1895	0	7	35	0.369	0.367
907	2020-4-21 8:00	BH	EMRO	57	1952	0	7	36	0.359	0.359
908	2020-4-22 8:00	BH	EMRO	55	2007	0	7	37	0.349	0.363
909	2020-4-23 8:00	BH	EMRO	91	2098	1	8	38	0.381	0.350
910	2020-4-24 8:00	BH	EMRO	408	2506	0	8	39	0.319	0.337
911	2020-4-25 8:00	BH	EMRO	82	2588	0	8	40	0.309	0.311
912	2020-4-26 8:00	BH	EMRO	45	2633	0	8	41	0.304	0.303
913	2020-4-27 8:00	BH	EMRO	75	2708	0	8	42	0.295	0.295
914	2020-4-28 8:00	BH	EMRO	102	2810	0	8	43	0.285	0.286
915	2020-4-29 8:00	BH	EMRO	59	2869	0	8	44	0.279	0.276
916	2020-4-30 8:00	BH	EMRO	168	3037	0	8	45	0.263	0.265
917	2020-5-1 8:00	BH	EMRO	132	3169	0	8	46	0.252	0.256
918	2020-5-2 8:00	BH	EMRO	1	3170	0	8	47	0.252	0.252
919										
920	2020-3-8 8:00	BD	SEARO	3	3	0	0			
921	2020-3-9 8:00	BD	SEARO	0	3	0	0			
922	2020-3-10 8:00	BD	SEARO	0	3	0	0			
923	2020-3-11 8:00	BD	SEARO	0	3	0	0			
924	2020-3-12 8:00	BD	SEARO	0	3	0	0			
925	2020-3-13 8:00	BD	SEARO	0	3	0	0			
926	2020-3-14 8:00	BD	SEARO	0	3	0	0			
927	2020-3-15 8:00	BD	SEARO	0	3	0	0			
928	2020-3-16 8:00	BD	SEARO	5	8	0	0			
929	2020-3-17 8:00	BD	SEARO	0	8	0	0			
930	2020-3-18 8:00	BD	SEARO	2	10	0	0			
931	2020-3-19 8:00	BD	SEARO	7	17	1	1	0	5.882	5.882
932	2020-3-20 8:00	BD	SEARO	0	17	0	1	1	5.882	6.699
933	2020-3-21 8:00	BD	SEARO	7	24	1	2	2	8.333	7.516
934	2020-3-22 8:00	BD	SEARO	0	24	0	2	3	8.333	8.025
935	2020-3-23 8:00	BD	SEARO	3	27	0	2	4	7.407	8.666
936	2020-3-24 8:00	BD	SEARO	12	39	2	4	5	10.256	9.307

Table S1 Daily confirmed cases and daily deaths of COVID-19 in 214 nations from WHO

ID	Date and time	Country or region	Continent	Confirmed patients	Cumulative Confirmed patients	Daily dead patients	Daily cumulative dead patients	Lag days	Daily CFR	3DMA CFR
937	2020-3-25 8:00	BD	SEARO	0	39	0	4	6	10.256	10.625
938	2020-3-26 8:00	BD	SEARO	5	44	1	5	7	11.364	10.679
939	2020-3-27 8:00	BD	SEARO	4	48	0	5	8	10.417	10.732
940	2020-3-28 8:00	BD	SEARO	0	48	0	5	9	10.417	10.417
941	2020-3-29 8:00	BD	SEARO	0	48	0	5	10	10.417	10.346
942	2020-3-30 8:00	BD	SEARO	1	49	0	5	11	10.204	10.275
943	2020-3-31 8:00	BD	SEARO	0	49	0	5	12	10.204	10.506
944	2020-4-1 8:00	BD	SEARO	5	54	1	6	13	11.111	10.676
945	2020-4-2 8:00	BD	SEARO	2	56	0	6	14	10.714	10.554
946	2020-4-3 8:00	BD	SEARO	5	61	0	6	15	9.836	10.660
947	2020-4-4 8:00	BD	SEARO	9	70	2	8	16	11.429	10.119
948	2020-4-5 8:00	BD	SEARO	18	88	0	8	17	9.091	10.092
949	2020-4-6 8:00	BD	SEARO	35	123	4	12	18	9.756	9.738
950	2020-4-7 8:00	BD	SEARO	41	164	5	17	19	10.366	9.765
951	2020-4-8 8:00	BD	SEARO	54	218	3	20	20	9.174	8.635
952	2020-4-9 8:00	BD	SEARO	112	330	1	21	21	6.364	7.302
953	2020-4-10 8:00	BD	SEARO	94	424	6	27	22	6.368	6.319
954	2020-4-11 8:00	BD	SEARO	58	482	3	30	23	6.224	6.022
955	2020-4-12 8:00	BD	SEARO	139	621	4	34	24	5.475	5.519
956	2020-4-13 8:00	BD	SEARO	182	803	5	39	25	4.857	4.959
957	2020-4-14 8:00	BD	SEARO	209	1012	7	46	26	4.545	4.488
958	2020-4-15 8:00	BD	SEARO	219	1231	4	50	27	4.062	4.141
959	2020-4-16 8:00	BD	SEARO	341	1572	10	60	28	3.817	3.986
960	2020-4-17 8:00	BD	SEARO	266	1838	15	75	29	4.081	3.938
961	2020-4-18 8:00	BD	SEARO	306	2144	9	84	30	3.918	3.901
962	2020-4-19 8:00	BD	SEARO	312	2456	7	91	31	3.705	3.683
963	2020-4-20 8:00	BD	SEARO	492	2948	10	101	32	3.426	3.461
964	2020-4-21 8:00	BD	SEARO	434	3382	9	110	33	3.253	3.287
965	2020-4-22 8:00	BD	SEARO	390	3772	10	120	34	3.181	3.156
966	2020-4-23 8:00	BD	SEARO	414	4186	7	127	35	3.034	3.003
967	2020-4-24 8:00	BD	SEARO	503	4689	4	131	36	2.794	2.876
968	2020-4-25 8:00	BD	SEARO	309	4998	9	140	37	2.801	2.757
969	2020-4-26 8:00	BD	SEARO	418	5416	5	145	38	2.677	2.683
970	2020-4-27 8:00	BD	SEARO	497	5913	7	152	39	2.571	2.549
971	2020-4-28 8:00	BD	SEARO	549	6462	3	155	40	2.399	2.421
972	2020-4-29 8:00	BD	SEARO	641	7103	8	163	41	2.295	2.295

Table S1 Daily confirmed cases and daily deaths of COVID-19 in 214 nations from WHO

ID	Date and time	Country or region	Continent	Confirmed patients	Cumulative Confirmed patients	Daily dead patients	Daily cumulative dead patients	Lag days	Daily CFR	3DMA CFR
973	2020-4-30 8:00	BD	SEARO	564	7667	5	168	42	2.191	2.183
974	2020-5-1 8:00	BD	SEARO	571	8238	2	170	43	2.064	2.106
975	2020-5-2 8:00	BD	SEARO	0	8238	0	170	44	2.064	2.064
976										
977	2020-3-18 8:00	BB	AMRO	2	2	0	0			
978	2020-3-19 8:00	BB	AMRO	0	2	0	0			
979	2020-3-20 8:00	BB	AMRO	3	5	0	0			
980	2020-3-21 8:00	BB	AMRO	0	5	0	0			
981	2020-3-22 8:00	BB	AMRO	0	5	0	0			
982	2020-3-23 8:00	BB	AMRO	0	5	0	0			
983	2020-3-24 8:00	BB	AMRO	13	18	0	0			
984	2020-3-25 8:00	BB	AMRO	0	18	0	0			
985	2020-3-26 8:00	BB	AMRO	0	18	0	0			
986	2020-3-27 8:00	BB	AMRO	0	18	0	0			
987	2020-3-28 8:00	BB	AMRO	6	24	0	0			
988	2020-3-29 8:00	BB	AMRO	0	24	0	0			
989	2020-3-30 8:00	BB	AMRO	2	26	0	0			
990	2020-3-31 8:00	BB	AMRO	7	33	0	0			
991	2020-4-1 8:00	BB	AMRO	0	33	0	0			
992	2020-4-2 8:00	BB	AMRO	0	33	0	0			
993	2020-4-3 8:00	BB	AMRO	12	45	0	0			
994	2020-4-4 8:00	BB	AMRO	0	45	0	0			
995	2020-4-5 8:00	BB	AMRO	6	51	0	0			
996	2020-4-6 8:00	BB	AMRO	0	51	0	0			
997	2020-4-7 8:00	BB	AMRO	5	56	1	1	0	1.786	2.560
998	2020-4-8 8:00	BB	AMRO	4	60	1	2	1	3.333	3.294
999	2020-4-9 8:00	BB	AMRO	3	63	1	3	2	4.762	4.286
1000	2020-4-10 8:00	BB	AMRO	0	63	0	3	3	4.762	5.195
1001	2020-4-11 8:00	BB	AMRO	3	66	1	4	4	6.061	5.598
1002	2020-4-12 8:00	BB	AMRO	1	67	0	4	5	5.970	5.971
1003	2020-4-13 8:00	BB	AMRO	1	68	0	4	6	5.882	5.829
1004	2020-4-14 8:00	BB	AMRO	3	71	0	4	7	5.634	5.691
1005	2020-4-15 8:00	BB	AMRO	1	72	0	4	8	5.556	6.013
1006	2020-4-16 8:00	BB	AMRO	1	73	1	5	9	6.849	6.357
1007	2020-4-17 8:00	BB	AMRO	2	75	0	5	10	6.667	6.728
1008	2020-4-18 8:00	BB	AMRO	0	75	0	5	11	6.667	6.667

Table S1 Daily confirmed cases and daily deaths of COVID-19 in 214 nations from WHO

ID	Date and time	Country or region	Continent	Confirmed patients	Cumulative Confirmed patients	Daily dead patients	Daily cumulative dead patients	Lag days	Daily CFR	3DMA CFR
1009	2020-4-19 8:00	BB	AMRO	0	75	0	5	12	6.667	6.667
1010	2020-4-20 8:00	BB	AMRO	0	75	0	5	13	6.667	6.667
1011	2020-4-21 8:00	BB	AMRO	0	75	0	5	14	6.667	6.667
1012	2020-4-22 8:00	BB	AMRO	0	75	0	5	15	6.667	6.667
1013	2020-4-23 8:00	BB	AMRO	0	75	0	5	16	6.667	7.076
1014	2020-4-24 8:00	BB	AMRO	1	76	1	6	17	7.895	7.485
1015	2020-4-25 8:00	BB	AMRO	0	76	0	6	18	7.895	7.861
1016	2020-4-26 8:00	BB	AMRO	1	77	0	6	19	7.792	7.761
1017	2020-4-27 8:00	BB	AMRO	2	79	0	6	20	7.595	7.661
1018	2020-4-28 8:00	BB	AMRO	0	79	0	6	21	7.595	7.563
1019	2020-4-29 8:00	BB	AMRO	1	80	0	6	22	7.500	7.532
1020	2020-4-30 8:00	BB	AMRO	0	80	0	6	23	7.500	7.917
1021	2020-5-1 8:00	BB	AMRO	0	80	1	7	24	8.750	8.297
1022	2020-5-2 8:00	BB	AMRO	1	81	0	7	25	8.642	8.696
1023										
1024	2020-2-28 8:00	BY	EURO	1	1	0	0			
1025	2020-2-29 8:00	BY	EURO	0	1	0	0			
1026	2020-3-1 8:00	BY	EURO	0	1	0	0			
1027	2020-3-2 8:00	BY	EURO	0	1	0	0			
1028	2020-3-3 8:00	BY	EURO	0	1	0	0			
1029	2020-3-4 8:00	BY	EURO	5	6	0	0			
1030	2020-3-5 8:00	BY	EURO	0	6	0	0			
1031	2020-3-6 8:00	BY	EURO	0	6	0	0			
1032	2020-3-7 8:00	BY	EURO	0	6	0	0			
1033	2020-3-8 8:00	BY	EURO	0	6	0	0			
1034	2020-3-9 8:00	BY	EURO	0	6	0	0			
1035	2020-3-10 8:00	BY	EURO	3	9	0	0			
1036	2020-3-11 8:00	BY	EURO	3	12	0	0			
1037	2020-3-12 8:00	BY	EURO	0	12	0	0			
1038	2020-3-13 8:00	BY	EURO	9	21	0	0			
1039	2020-3-14 8:00	BY	EURO	0	21	0	0			
1040	2020-3-15 8:00	BY	EURO	0	21	0	0			
1041	2020-3-16 8:00	BY	EURO	15	36	0	0			
1042	2020-3-17 8:00	BY	EURO	0	36	0	0			
1043	2020-3-18 8:00	BY	EURO	10	46	0	0			
1044	2020-3-19 8:00	BY	EURO	0	46	0	0			

Table S1 Daily confirmed cases and daily deaths of COVID-19 in 214 nations from WHO

ID	Date and time	Country or region	Continent	Confirmed patients	Cumulative Confirmed patients	Daily dead patients	Daily cumulative dead patients	Lag days	Daily CFR	3DMA CFR
1045	2020-3-20 8:00	BY	EURO	11	57	0	0			
1046	2020-3-21 8:00	BY	EURO	19	76	0	0			
1047	2020-3-22 8:00	BY	EURO	0	76	0	0			
1048	2020-3-23 8:00	BY	EURO	0	76	0	0			
1049	2020-3-24 8:00	BY	EURO	5	81	0	0			
1050	2020-3-25 8:00	BY	EURO	0	81	0	0			
1051	2020-3-26 8:00	BY	EURO	5	86	0	0			
1052	2020-3-27 8:00	BY	EURO	0	86	0	0			
1053	2020-3-28 8:00	BY	EURO	8	94	0	0			
1054	2020-3-29 8:00	BY	EURO	0	94	0	0			
1055	2020-3-30 8:00	BY	EURO	58	152	0	0			
1056	2020-3-31 8:00	BY	EURO	0	152	0	0			
1057	2020-4-1 8:00	BY	EURO	0	152	0	0			
1058	2020-4-2 8:00	BY	EURO	40	192	2	2	0	1.042	1.308
1059	2020-4-3 8:00	BY	EURO	62	254	2	4	1	1.575	1.397
1060	2020-4-4 8:00	BY	EURO	0	254	0	4	2	1.575	1.353
1061	2020-4-5 8:00	BY	EURO	186	440	0	4	3	0.909	1.302
1062	2020-4-6 8:00	BY	EURO	122	562	4	8	4	1.423	1.397
1063	2020-4-7 8:00	BY	EURO	138	700	5	13	5	1.857	1.597
1064	2020-4-8 8:00	BY	EURO	161	861	0	13	6	1.510	1.529
1065	2020-4-9 8:00	BY	EURO	205	1066	0	13	7	1.220	1.316
1066	2020-4-10 8:00	BY	EURO	0	1066	0	13	8	1.220	1.133
1067	2020-4-11 8:00	BY	EURO	915	1981	6	19	9	0.959	1.071
1068	2020-4-12 8:00	BY	EURO	245	2226	4	23	10	1.033	1.000
1069	2020-4-13 8:00	BY	EURO	352	2578	3	26	11	1.009	1.012
1070	2020-4-14 8:00	BY	EURO	341	2919	3	29	12	0.993	1.003
1071	2020-4-15 8:00	BY	EURO	362	3281	4	33	13	1.006	0.988
1072	2020-4-16 8:00	BY	EURO	447	3728	3	36	14	0.966	0.974
1073	2020-4-17 8:00	BY	EURO	476	4204	4	40	15	0.951	0.939
1074	2020-4-18 8:00	BY	EURO	575	4779	3	43	16	0.900	0.931
1075	2020-4-19 8:00	BY	EURO	0	4779	2	45	17	0.942	0.942
1076	2020-4-20 8:00	BY	EURO	0	4779	2	47	18	0.983	0.913
1077	2020-4-21 8:00	BY	EURO	1485	6264	4	51	19	0.814	0.872
1078	2020-4-22 8:00	BY	EURO	459	6723	4	55	20	0.818	0.819
1079	2020-4-23 8:00	BY	EURO	558	7281	5	60	21	0.824	0.797
1080	2020-4-24 8:00	BY	EURO	741	8022	0	60	22	0.748	0.763

Table S1 Daily confirmed cases and daily deaths of COVID-19 in 214 nations from WHO

ID	Date and time	Country or region	Continent	Confirmed patients	Cumulative Confirmed patients	Daily dead patients	Daily cumulative dead patients	Lag days	Daily CFR	3DMA CFR
1081	2020-4-25 8:00	BY	EURO	751	8773	3	63	23	0.718	0.722
1082	2020-4-26 8:00	BY	EURO	817	9590	4	67	24	0.699	0.702
1083	2020-4-27 8:00	BY	EURO	873	10463	5	72	25	0.688	0.678
1084	2020-4-28 8:00	BY	EURO	1745	12208	7	79	26	0.647	0.661
1085	2020-4-29 8:00	BY	EURO	0	12208	0	79	27	0.647	0.644
1086	2020-4-30 8:00	BY	EURO	973	13181	5	84	28	0.637	0.640
1087	2020-5-1 8:00	BY	EURO	846	14027	5	89	29	0.634	0.632
1088	2020-5-2 8:00	BY	EURO	890	14917	4	93	30	0.623	0.629
1089										
1090	2020-2-4 8:00	BE	EURO	1	1	0	0			
1091	2020-2-5 8:00	BE	EURO	0	1	0	0			
1092	2020-2-6 8:00	BE	EURO	0	1	0	0			
1093	2020-2-7 8:00	BE	EURO	0	1	0	0			
1094	2020-2-8 8:00	BE	EURO	0	1	0	0			
1095	2020-2-9 8:00	BE	EURO	0	1	0	0			
1096	2020-2-10 8:00	BE	EURO	0	1	0	0			
1097	2020-2-11 8:00	BE	EURO	0	1	0	0			
1098	2020-2-12 8:00	BE	EURO	0	1	0	0			
1099	2020-2-13 8:00	BE	EURO	0	1	0	0			
1100	2020-2-14 8:00	BE	EURO	0	1	0	0			
1101	2020-2-15 8:00	BE	EURO	0	1	0	0			
1102	2020-2-16 8:00	BE	EURO	0	1	0	0			
1103	2020-2-17 8:00	BE	EURO	0	1	0	0			
1104	2020-2-18 8:00	BE	EURO	0	1	0	0			
1105	2020-2-19 8:00	BE	EURO	0	1	0	0			
1106	2020-2-20 8:00	BE	EURO	0	1	0	0			
1107	2020-2-21 8:00	BE	EURO	0	1	0	0			
1108	2020-2-22 8:00	BE	EURO	0	1	0	0			
1109	2020-2-23 8:00	BE	EURO	0	1	0	0			
1110	2020-2-24 8:00	BE	EURO	0	1	0	0			
1111	2020-2-25 8:00	BE	EURO	0	1	0	0			
1112	2020-2-26 8:00	BE	EURO	0	1	0	0			
1113	2020-2-27 8:00	BE	EURO	0	1	0	0			
1114	2020-2-28 8:00	BE	EURO	0	1	0	0			
1115	2020-2-29 8:00	BE	EURO	0	1	0	0			
1116	2020-3-1 8:00	BE	EURO	0	1	0	0			

Table S1 Daily confirmed cases and daily deaths of COVID-19 in 214 nations from WHO

ID	Date and time	Country or region	Continent	Confirmed patients	Cumulative Confirmed patients	Daily dead patients	Daily cumulative dead patients	Lag days	Daily CFR	3DMA CFR
1117	2020-3-2 8:00	BE	EURO	7	8	0	0			
1118	2020-3-3 8:00	BE	EURO	0	8	0	0			
1119	2020-3-4 8:00	BE	EURO	15	23	0	0			
1120	2020-3-5 8:00	BE	EURO	27	50	0	0			
1121	2020-3-6 8:00	BE	EURO	59	109	0	0			
1122	2020-3-7 8:00	BE	EURO	60	169	0	0			
1123	2020-3-8 8:00	BE	EURO	31	200	0	0			
1124	2020-3-9 8:00	BE	EURO	0	200	0	0			
1125	2020-3-10 8:00	BE	EURO	67	267	0	0			
1126	2020-3-11 8:00	BE	EURO	0	267	0	0			
1127	2020-3-12 8:00	BE	EURO	47	314	0	0			
1128	2020-3-13 8:00	BE	EURO	285	599	0	0			
1129	2020-3-14 8:00	BE	EURO	90	689	0	0			
1130	2020-3-15 8:00	BE	EURO	197	886	0	0			
1131	2020-3-16 8:00	BE	EURO	199	1085	5	5	0	0.461	0.701
1132	2020-3-17 8:00	BE	EURO	401	1486	9	14	1	0.942	0.782
1133	2020-3-18 8:00	BE	EURO	0	1486	0	14	2	0.942	0.888
1134	2020-3-19 8:00	BE	EURO	309	1795	0	14	3	0.780	1.120
1135	2020-3-20 8:00	BE	EURO	462	2257	23	37	4	1.639	1.600
1136	2020-3-21 8:00	BE	EURO	558	2815	30	67	5	2.380	2.133
1137	2020-3-22 8:00	BE	EURO	0	2815	0	67	6	2.380	2.322
1138	2020-3-23 8:00	BE	EURO	586	3401	8	75	7	2.205	2.312
1139	2020-3-24 8:00	BE	EURO	342	3743	13	88	8	2.351	2.471
1140	2020-3-25 8:00	BE	EURO	526	4269	34	122	9	2.858	2.938
1141	2020-3-26 8:00	BE	EURO	668	4937	56	178	10	3.605	3.331
1142	2020-3-27 8:00	BE	EURO	1298	6235	42	220	11	3.528	3.700
1143	2020-3-28 8:00	BE	EURO	1049	7284	69	289	12	3.968	3.787
1144	2020-3-29 8:00	BE	EURO	1850	9134	64	353	13	3.865	3.937
1145	2020-3-30 8:00	BE	EURO	1702	10836	78	431	14	3.977	4.051
1146	2020-3-31 8:00	BE	EURO	1063	11899	82	513	15	4.311	4.602
1147	2020-4-1 8:00	BE	EURO	876	12775	192	705	16	5.519	5.253
1148	2020-4-2 8:00	BE	EURO	1189	13964	123	828	17	5.930	6.012
1149	2020-4-3 8:00	BE	EURO	1384	15348	183	1011	18	6.587	6.444
1150	2020-4-4 8:00	BE	EURO	1422	16770	132	1143	19	6.816	6.788
1151	2020-4-5 8:00	BE	EURO	1661	18431	140	1283	20	6.961	7.042
1152	2020-4-6 8:00	BE	EURO	1260	19691	164	1447	21	7.349	7.384

Table S1 Daily confirmed cases and daily deaths of COVID-19 in 214 nations from WHO

ID	Date and time	Country or region	Continent	Confirmed patients	Cumulative Confirmed patients	Daily dead patients	Daily cumulative dead patients	Lag days	Daily CFR	3DMA CFR
1153	2020-4-7 8:00	BE	EURO	1123	20814	185	1632	22	7.841	8.120
1154	2020-4-8 8:00	BE	EURO	1380	22194	403	2035	23	9.169	8.860
1155	2020-4-9 8:00	BE	EURO	1209	23403	205	2240	24	9.571	9.613
1156	2020-4-10 8:00	BE	EURO	1580	24983	283	2523	25	10.099	10.330
1157	2020-4-11 8:00	BE	EURO	1684	26667	496	3019	26	11.321	11.121
1158	2020-4-12 8:00	BE	EURO	1351	28018	327	3346	27	11.942	11.802
1159	2020-4-13 8:00	BE	EURO	1629	29647	254	3600	28	12.143	12.282
1160	2020-4-14 8:00	BE	EURO	942	30589	303	3903	29	12.759	12.754
1161	2020-4-15 8:00	BE	EURO	530	31119	254	4157	30	13.358	13.114
1162	2020-4-16 8:00	BE	EURO	2454	33573	283	4440	31	13.225	13.512
1163	2020-4-17 8:00	BE	EURO	1236	34809	417	4857	32	13.953	13.822
1164	2020-4-18 8:00	BE	EURO	1329	36138	306	5163	33	14.287	14.302
1165	2020-4-19 8:00	BE	EURO	1045	37183	290	5453	34	14.665	14.572
1166	2020-4-20 8:00	BE	EURO	1313	38496	230	5683	35	14.763	14.668
1167	2020-4-21 8:00	BE	EURO	1487	39983	145	5828	36	14.576	14.661
1168	2020-4-22 8:00	BE	EURO	973	40956	170	5998	37	14.645	14.723
1169	2020-4-23 8:00	BE	EURO	933	41889	264	6262	38	14.949	14.920
1170	2020-4-24 8:00	BE	EURO	908	42797	228	6490	39	15.165	15.064
1171	2020-4-25 8:00	BE	EURO	1496	44293	189	6679	40	15.079	15.168
1172	2020-4-26 8:00	BE	EURO	1032	45325	238	6917	41	15.261	15.239
1173	2020-4-27 8:00	BE	EURO	809	46134	177	7094	42	15.377	15.358
1174	2020-4-28 8:00	BE	EURO	553	46687	113	7207	43	15.437	15.434
1175	2020-4-29 8:00	BE	EURO	647	47334	124	7331	44	15.488	15.533
1176	2020-4-30 8:00	BE	EURO	525	47859	170	7501	45	15.673	15.604
1177	2020-5-1 8:00	BE	EURO	660	48519	93	7594	46	15.652	15.678
1178	2020-5-2 8:00	BE	EURO	513	49032	109	7703	47	15.710	15.681
1179										
1180	2020-3-24 8:00	BZ	AMRO	1	1	0	0			
1181	2020-3-25 8:00	BZ	AMRO	0	1	0	0			
1182	2020-3-26 8:00	BZ	AMRO	1	2	0	0			
1183	2020-3-27 8:00	BZ	AMRO	0	2	0	0			
1184	2020-3-28 8:00	BZ	AMRO	0	2	0	0			
1185	2020-3-29 8:00	BZ	AMRO	0	2	0	0			
1186	2020-3-30 8:00	BZ	AMRO	0	2	0	0			
1187	2020-3-31 8:00	BZ	AMRO	1	3	0	0			
1188	2020-4-1 8:00	BZ	AMRO	0	3	0	0			

Table S1 Daily confirmed cases and daily deaths of COVID-19 in 214 nations from WHO

ID	Date and time	Country or region	Continent	Confirmed patients	Cumulative Confirmed patients	Daily dead patients	Daily cumulative dead patients	Lag days	Daily CFR	3DMA CFR
1189	2020-4-2 8:00	BZ	AMRO	0	3	0	0			
1190	2020-4-3 8:00	BZ	AMRO	0	3	0	0			
1191	2020-4-4 8:00	BZ	AMRO	0	3	0	0			
1192	2020-4-5 8:00	BZ	AMRO	1	4	0	0			
1193	2020-4-6 8:00	BZ	AMRO	1	5	0	0			
1194	2020-4-7 8:00	BZ	AMRO	2	7	1	1	0	14.286	14.286
1195	2020-4-8 8:00	BZ	AMRO	0	7	0	1	1	14.286	13.690
1196	2020-4-9 8:00	BZ	AMRO	1	8	0	1	2	12.500	12.632
1197	2020-4-10 8:00	BZ	AMRO	1	9	0	1	3	11.111	11.204
1198	2020-4-11 8:00	BZ	AMRO	1	10	0	1	4	10.000	13.704
1199	2020-4-12 8:00	BZ	AMRO	0	10	1	2	5	20.000	15.128
1200	2020-4-13 8:00	BZ	AMRO	3	13	0	2	6	15.385	16.557
1201	2020-4-14 8:00	BZ	AMRO	1	14	0	2	7	14.286	13.594
1202	2020-4-15 8:00	BZ	AMRO	4	18	0	2	8	11.111	12.169
1203	2020-4-16 8:00	BZ	AMRO	0	18	0	2	9	11.111	11.111
1204	2020-4-17 8:00	BZ	AMRO	0	18	0	2	10	11.111	11.111
1205	2020-4-18 8:00	BZ	AMRO	0	18	0	2	11	11.111	11.111
1206	2020-4-19 8:00	BZ	AMRO	0	18	0	2	12	11.111	11.111
1207	2020-4-20 8:00	BZ	AMRO	0	18	0	2	13	11.111	11.111
1208	2020-4-21 8:00	BZ	AMRO	0	18	0	2	14	11.111	11.111
1209	2020-4-22 8:00	BZ	AMRO	0	18	0	2	15	11.111	11.111
1210	2020-4-23 8:00	BZ	AMRO	0	18	0	2	16	11.111	11.111
1211	2020-4-24 8:00	BZ	AMRO	0	18	0	2	17	11.111	11.111
1212	2020-4-25 8:00	BZ	AMRO	0	18	0	2	18	11.111	11.111
1213	2020-4-26 8:00	BZ	AMRO	0	18	0	2	19	11.111	11.111
1214	2020-4-27 8:00	BZ	AMRO	0	18	0	2	20	11.111	11.111
1215	2020-4-28 8:00	BZ	AMRO	0	18	0	2	21	11.111	11.111
1216	2020-4-29 8:00	BZ	AMRO	0	18	0	2	22	11.111	11.111
1217	2020-4-30 8:00	BZ	AMRO	0	18	0	2	23	11.111	11.111
1218	2020-5-1 8:00	BZ	AMRO	0	18	0	2	24	11.111	11.111
1219	2020-5-2 8:00	BZ	AMRO	0	18	0	2	25	11.111	11.111
1220										
1221	2020-3-17 8:00	BJ	AFRO	1	1	0	0			
1222	2020-3-18 8:00	BJ	AFRO	0	1	0	0			
1223	2020-3-19 8:00	BJ	AFRO	1	2	0	0			
1224	2020-3-20 8:00	BJ	AFRO	0	2	0	0			

Table S1 Daily confirmed cases and daily deaths of COVID-19 in 214 nations from WHO

ID	Date and time	Country or region	Continent	Confirmed patients	Cumulative Confirmed patients	Daily dead patients	Daily cumulative dead patients	Lag days	Daily CFR	3DMA CFR
1225	2020-3-21 8:00	BJ	AFRO	0	2	0	0			
1226	2020-3-22 8:00	BJ	AFRO	0	2	0	0			
1227	2020-3-23 8:00	BJ	AFRO	0	2	0	0			
1228	2020-3-24 8:00	BJ	AFRO	3	5	0	0			
1229	2020-3-25 8:00	BJ	AFRO	0	5	0	0			
1230	2020-3-26 8:00	BJ	AFRO	1	6	0	0			
1231	2020-3-27 8:00	BJ	AFRO	0	6	0	0			
1232	2020-3-28 8:00	BJ	AFRO	0	6	0	0			
1233	2020-3-29 8:00	BJ	AFRO	0	6	0	0			
1234	2020-3-30 8:00	BJ	AFRO	0	6	0	0			
1235	2020-3-31 8:00	BJ	AFRO	0	6	0	0			
1236	2020-4-1 8:00	BJ	AFRO	3	9	0	0			
1237	2020-4-2 8:00	BJ	AFRO	4	13	0	0			
1238	2020-4-3 8:00	BJ	AFRO	0	13	0	0			
1239	2020-4-4 8:00	BJ	AFRO	0	13	0	0			
1240	2020-4-5 8:00	BJ	AFRO	0	13	0	0			
1241	2020-4-6 8:00	BJ	AFRO	9	22	0	0			
1242	2020-4-7 8:00	BJ	AFRO	1	23	1	1	0	4.348	4.097
1243	2020-4-8 8:00	BJ	AFRO	3	26	0	1	1	3.846	4.013
1244	2020-4-9 8:00	BJ	AFRO	0	26	0	1	2	3.846	3.675
1245	2020-4-10 8:00	BJ	AFRO	4	30	0	1	3	3.333	3.504
1246	2020-4-11 8:00	BJ	AFRO	0	30	0	1	4	3.333	3.175
1247	2020-4-12 8:00	BJ	AFRO	5	35	0	1	5	2.857	3.016
1248	2020-4-13 8:00	BJ	AFRO	0	35	0	1	6	2.857	2.857
1249	2020-4-14 8:00	BJ	AFRO	0	35	0	1	7	2.857	2.857
1250	2020-4-15 8:00	BJ	AFRO	0	35	0	1	8	2.857	2.857
1251	2020-4-16 8:00	BJ	AFRO	0	35	0	1	9	2.857	2.806
1252	2020-4-17 8:00	BJ	AFRO	2	37	0	1	10	2.703	2.754
1253	2020-4-18 8:00	BJ	AFRO	0	37	0	1	11	2.703	2.703
1254	2020-4-19 8:00	BJ	AFRO	0	37	0	1	12	2.703	2.703
1255	2020-4-20 8:00	BJ	AFRO	0	37	0	1	13	2.703	2.419
1256	2020-4-21 8:00	BJ	AFRO	17	54	0	1	14	1.852	2.135
1257	2020-4-22 8:00	BJ	AFRO	0	54	0	1	15	1.852	1.852
1258	2020-4-23 8:00	BJ	AFRO	0	54	0	1	16	1.852	1.809
1259	2020-4-24 8:00	BJ	AFRO	4	58	0	1	17	1.724	1.767
1260	2020-4-25 8:00	BJ	AFRO	0	58	0	1	18	1.724	1.724

Table S1 Daily confirmed cases and daily deaths of COVID-19 in 214 nations from WHO

ID	Date and time	Country or region	Continent	Confirmed patients	Cumulative Confirmed patients	Daily dead patients	Daily cumulative dead patients	Lag days	Daily CFR	3DMA CFR
1261	2020-4-26 8:00	BJ	AFRO	0	58	0	1	19	1.724	1.670
1262	2020-4-27 8:00	BJ	AFRO	6	64	0	1	20	1.563	1.616
1263	2020-4-28 8:00	BJ	AFRO	0	64	0	1	21	1.563	1.563
1264	2020-4-29 8:00	BJ	AFRO	0	64	0	1	22	1.563	2.008
1265	2020-4-30 8:00	BJ	AFRO	5	69	1	2	23	2.899	2.281
1266	2020-5-1 8:00	BJ	AFRO	15	84	0	2	24	2.381	2.501
1267	2020-5-2 8:00	BJ	AFRO	6	90	0	2	25	2.222	2.302
1268										
1269	2020-3-18 8:00	BM	AMRO	2	2	0	0			
1270	2020-3-19 8:00	BM	AMRO	0	2	0	0			
1271	2020-3-20 8:00	BM	AMRO	0	2	0	0			
1272	2020-3-21 8:00	BM	AMRO	0	2	0	0			
1273	2020-3-22 8:00	BM	AMRO	0	2	0	0			
1274	2020-3-23 8:00	BM	AMRO	0	2	0	0			
1275	2020-3-24 8:00	BM	AMRO	4	6	0	0			
1276	2020-3-25 8:00	BM	AMRO	0	6	0	0			
1277	2020-3-26 8:00	BM	AMRO	1	7	0	0			
1278	2020-3-27 8:00	BM	AMRO	0	7	0	0			
1279	2020-3-28 8:00	BM	AMRO	8	15	0	0			
1280	2020-3-29 8:00	BM	AMRO	2	17	0	0			
1281	2020-3-30 8:00	BM	AMRO	5	22	0	0			
1282	2020-3-31 8:00	BM	AMRO	0	22	0	0			
1283	2020-4-1 8:00	BM	AMRO	5	27	0	0			
1284	2020-4-2 8:00	BM	AMRO	5	32	0	0			
1285	2020-4-3 8:00	BM	AMRO	0	32	0	0			
1286	2020-4-4 8:00	BM	AMRO	3	35	0	0			
1287	2020-4-5 8:00	BM	AMRO	0	35	0	0			
1288	2020-4-6 8:00	BM	AMRO	2	37	0	0			
1289	2020-4-7 8:00	BM	AMRO	0	37	0	0			
1290	2020-4-8 8:00	BM	AMRO	2	39	2	2	0	5.128	5.128
1291	2020-4-9 8:00	BM	AMRO	0	39	0	2	1	5.128	5.983
1292	2020-4-10 8:00	BM	AMRO	0	39	1	3	2	7.692	7.051
1293	2020-4-11 8:00	BM	AMRO	9	48	1	4	3	8.333	8.120
1294	2020-4-12 8:00	BM	AMRO	0	48	0	4	4	8.333	8.222
1295	2020-4-13 8:00	BM	AMRO	2	50	0	4	5	8.000	7.784
1296	2020-4-14 8:00	BM	AMRO	7	57	0	4	6	7.018	7.930

Table S1 Daily confirmed cases and daily deaths of COVID-19 in 214 nations from WHO

ID	Date and time	Country or region	Continent	Confirmed patients	Cumulative Confirmed patients	Daily dead patients	Daily cumulative dead patients	Lag days	Daily CFR	3DMA CFR
1297	2020-4-15 8:00	BM	AMRO	0	57	1	5	7	8.772	8.187
1298	2020-4-16 8:00	BM	AMRO	0	57	0	5	8	8.772	7.906
1299	2020-4-17 8:00	BM	AMRO	24	81	0	5	9	6.173	6.990
1300	2020-4-18 8:00	BM	AMRO	2	83	0	5	10	6.024	6.074
1301	2020-4-19 8:00	BM	AMRO	0	83	0	5	11	6.024	5.954
1302	2020-4-20 8:00	BM	AMRO	3	86	0	5	12	5.814	5.884
1303	2020-4-21 8:00	BM	AMRO	0	86	0	5	13	5.814	5.814
1304	2020-4-22 8:00	BM	AMRO	0	86	0	5	14	5.814	5.577
1305	2020-4-23 8:00	BM	AMRO	12	98	0	5	15	5.102	5.322
1306	2020-4-24 8:00	BM	AMRO	1	99	0	5	16	5.051	5.068
1307	2020-4-25 8:00	BM	AMRO	0	99	0	5	17	5.051	5.051
1308	2020-4-26 8:00	BM	AMRO	0	99	0	5	18	5.051	4.896
1309	2020-4-27 8:00	BM	AMRO	10	109	0	5	19	4.587	5.047
1310	2020-4-28 8:00	BM	AMRO	0	109	1	6	20	5.505	5.182
1311	2020-4-29 8:00	BM	AMRO	1	110	0	6	21	5.455	5.455
1312	2020-4-30 8:00	BM	AMRO	1	111	0	6	22	5.405	5.422
1313	2020-5-1 8:00	BM	AMRO	0	111	0	6	23	5.405	5.358
1314	2020-5-2 8:00	BM	AMRO	3	114	0	6	24	5.263	5.334
1315										
1316	2020-3-6 8:00	BT	SEARO	1	1	0	0			
1317	2020-3-7 8:00	BT	SEARO	0	1	0	0			
1318	2020-3-8 8:00	BT	SEARO	0	1	0	0			
1319	2020-3-9 8:00	BT	SEARO	0	1	0	0			
1320	2020-3-10 8:00	BT	SEARO	0	1	0	0			
1321	2020-3-11 8:00	BT	SEARO	0	1	0	0			
1322	2020-3-12 8:00	BT	SEARO	0	1	0	0			
1323	2020-3-13 8:00	BT	SEARO	0	1	0	0			
1324	2020-3-14 8:00	BT	SEARO	0	1	0	0			
1325	2020-3-15 8:00	BT	SEARO	0	1	0	0			
1326	2020-3-16 8:00	BT	SEARO	0	1	0	0			
1327	2020-3-17 8:00	BT	SEARO	0	1	0	0			
1328	2020-3-18 8:00	BT	SEARO	0	1	0	0			
1329	2020-3-19 8:00	BT	SEARO	1	2	0	0			
1330	2020-3-20 8:00	BT	SEARO	0	2	0	0			
1331	2020-3-21 8:00	BT	SEARO	0	2	0	0			
1332	2020-3-22 8:00	BT	SEARO	0	2	0	0			

Table S1 Daily confirmed cases and daily deaths of COVID-19 in 214 nations from WHO

ID	Date and time	Country or region	Continent	Confirmed patients	Cumulative Confirmed patients	Daily dead patients	Daily cumulative dead patients	Lag days	Daily CFR	3DMA CFR
1333	2020-3-23 8:00	BT	SEARO	0	2	0	0			
1334	2020-3-24 8:00	BT	SEARO	0	2	0	0			
1335	2020-3-25 8:00	BT	SEARO	0	2	0	0			
1336	2020-3-26 8:00	BT	SEARO	1	3	0	0			
1337	2020-3-27 8:00	BT	SEARO	0	3	0	0			
1338	2020-3-28 8:00	BT	SEARO	0	3	0	0			
1339	2020-3-29 8:00	BT	SEARO	0	3	0	0			
1340	2020-3-30 8:00	BT	SEARO	1	4	0	0			
1341	2020-3-31 8:00	BT	SEARO	0	4	0	0			
1342	2020-4-1 8:00	BT	SEARO	0	4	0	0			
1343	2020-4-2 8:00	BT	SEARO	1	5	0	0			
1344	2020-4-3 8:00	BT	SEARO	0	5	0	0			
1345	2020-4-4 8:00	BT	SEARO	0	5	0	0			
1346	2020-4-5 8:00	BT	SEARO	0	5	0	0			
1347	2020-4-6 8:00	BT	SEARO	0	5	0	0			
1348	2020-4-7 8:00	BT	SEARO	0	5	0	0			
1349	2020-4-8 8:00	BT	SEARO	0	5	0	0			
1350	2020-4-9 8:00	BT	SEARO	0	5	0	0			
1351	2020-4-10 8:00	BT	SEARO	0	5	0	0			
1352	2020-4-11 8:00	BT	SEARO	0	5	0	0			
1353	2020-4-12 8:00	BT	SEARO	0	5	0	0			
1354	2020-4-13 8:00	BT	SEARO	0	5	0	0			
1355	2020-4-14 8:00	BT	SEARO	0	5	0	0			
1356	2020-4-15 8:00	BT	SEARO	0	5	0	0			
1357	2020-4-16 8:00	BT	SEARO	0	5	0	0			
1358	2020-4-17 8:00	BT	SEARO	0	5	0	0			
1359	2020-4-18 8:00	BT	SEARO	0	5	0	0			
1360	2020-4-19 8:00	BT	SEARO	0	5	0	0			
1361	2020-4-20 8:00	BT	SEARO	0	5	0	0			
1362	2020-4-21 8:00	BT	SEARO	1	6	0	0			
1363	2020-4-22 8:00	BT	SEARO	0	6	0	0			
1364	2020-4-23 8:00	BT	SEARO	1	7	0	0			
1365	2020-4-24 8:00	BT	SEARO	0	7	0	0			
1366	2020-4-25 8:00	BT	SEARO	0	7	0	0			
1367	2020-4-26 8:00	BT	SEARO	0	7	0	0			
1368	2020-4-27 8:00	BT	SEARO	0	7	0	0			

Table S1 Daily confirmed cases and daily deaths of COVID-19 in 214 nations from WHO

ID	Date and time	Country or region	Continent	Confirmed patients	Cumulative Confirmed patients	Daily dead patients	Daily cumulative dead patients	Lag days	Daily CFR	3DMA CFR
1369	2020-4-28 8:00	BT	SEARO	0	7	0	0			
1370	2020-4-29 8:00	BT	SEARO	0	7	0	0			
1371	2020-4-30 8:00	BT	SEARO	0	7	0	0			
1372	2020-5-1 8:00	BT	SEARO	0	7	0	0			
1373	2020-5-2 8:00	BT	SEARO	0	7	0	0			
1374										
1375	2020-3-11 8:00	BO	AMRO	2	2	0	0			
1376	2020-3-12 8:00	BO	AMRO	0	2	0	0			
1377	2020-3-13 8:00	BO	AMRO	1	3	0	0			
1378	2020-3-14 8:00	BO	AMRO	0	3	0	0			
1379	2020-3-15 8:00	BO	AMRO	0	3	0	0			
1380	2020-3-16 8:00	BO	AMRO	8	11	0	0			
1381	2020-3-17 8:00	BO	AMRO	0	11	0	0			
1382	2020-3-18 8:00	BO	AMRO	1	12	0	0			
1383	2020-3-19 8:00	BO	AMRO	3	15	0	0			
1384	2020-3-20 8:00	BO	AMRO	1	16	0	0			
1385	2020-3-21 8:00	BO	AMRO	3	19	0	0			
1386	2020-3-22 8:00	BO	AMRO	0	19	0	0			
1387	2020-3-23 8:00	BO	AMRO	5	24	0	0			
1388	2020-3-24 8:00	BO	AMRO	4	28	0	0			
1389	2020-3-25 8:00	BO	AMRO	0	28	0	0			
1390	2020-3-26 8:00	BO	AMRO	11	39	0	0			
1391	2020-3-27 8:00	BO	AMRO	0	39	0	0			
1392	2020-3-28 8:00	BO	AMRO	22	61	0	0			
1393	2020-3-29 8:00	BO	AMRO	13	74	0	0			
1394	2020-3-30 8:00	BO	AMRO	0	74	0	0			
1395	2020-3-31 8:00	BO	AMRO	23	97	5	5	0	5.155	5.381
1396	2020-4-1 8:00	BO	AMRO	10	107	1	6	1	5.607	5.616
1397	2020-4-2 8:00	BO	AMRO	8	115	1	7	2	6.087	6.066
1398	2020-4-3 8:00	BO	AMRO	8	123	1	8	3	6.504	6.470
1399	2020-4-4 8:00	BO	AMRO	9	132	1	9	4	6.818	6.839
1400	2020-4-5 8:00	BO	AMRO	7	139	1	10	5	7.194	6.794
1401	2020-4-6 8:00	BO	AMRO	18	157	0	10	6	6.369	6.525
1402	2020-4-7 8:00	BO	AMRO	26	183	1	11	7	6.011	6.532
1403	2020-4-8 8:00	BO	AMRO	11	194	3	14	8	7.216	6.790
1404	2020-4-9 8:00	BO	AMRO	16	210	1	15	9	7.143	7.059

Table S1 Daily confirmed cases and daily deaths of COVID-19 in 214 nations from WHO

ID	Date and time	Country or region	Continent	Confirmed patients	Cumulative Confirmed patients	Daily dead patients	Daily cumulative dead patients	Lag days	Daily CFR	3DMA CFR
1405	2020-4-10 8:00	BO	AMRO	54	264	3	18	10	6.818	7.017
1406	2020-4-11 8:00	BO	AMRO	4	268	1	19	11	7.090	7.060
1407	2020-4-12 8:00	BO	AMRO	7	275	1	20	12	7.273	7.454
1408	2020-4-13 8:00	BO	AMRO	25	300	4	24	13	8.000	7.818
1409	2020-4-14 8:00	BO	AMRO	30	330	3	27	14	8.182	8.030
1410	2020-4-15 8:00	BO	AMRO	24	354	1	28	15	7.910	7.715
1411	2020-4-16 8:00	BO	AMRO	43	397	0	28	16	7.053	7.179
1412	2020-4-17 8:00	BO	AMRO	44	441	1	29	17	6.576	6.765
1413	2020-4-18 8:00	BO	AMRO	24	465	2	31	18	6.667	6.510
1414	2020-4-19 8:00	BO	AMRO	28	493	0	31	19	6.288	6.370
1415	2020-4-20 8:00	BO	AMRO	27	520	1	32	20	6.154	6.098
1416	2020-4-21 8:00	BO	AMRO	44	564	1	33	21	5.851	5.897
1417	2020-4-22 8:00	BO	AMRO	34	598	1	34	22	5.686	5.871
1418	2020-4-23 8:00	BO	AMRO	11	609	3	37	23	6.076	5.905
1419	2020-4-24 8:00	BO	AMRO	63	672	3	40	24	5.952	6.048
1420	2020-4-25 8:00	BO	AMRO	31	703	3	43	25	6.117	5.840
1421	2020-4-26 8:00	BO	AMRO	104	807	1	44	26	5.452	5.627
1422	2020-4-27 8:00	BO	AMRO	59	866	2	46	27	5.312	5.342
1423	2020-4-28 8:00	BO	AMRO	84	950	4	50	28	5.263	5.267
1424	2020-4-29 8:00	BO	AMRO	64	1014	3	53	29	5.227	5.238
1425	2020-4-30 8:00	BO	AMRO	39	1053	2	55	30	5.223	5.255
1426	2020-5-1 8:00	BO	AMRO	57	1110	4	59	31	5.315	5.284
1427	2020-5-2 8:00	BO	AMRO	57	1167	3	62	32	5.313	5.314
1428										
1429	2020-4-4 8:00	BQ	AMRO	2	2	0	0			
1430	2020-4-5 8:00	BQ	AMRO	0	2	0	0			
1431	2020-4-6 8:00	BQ	AMRO	0	2	0	0			
1432	2020-4-7 8:00	BQ	AMRO	0	2	0	0			
1433	2020-4-8 8:00	BQ	AMRO	0	2	0	0			
1434	2020-4-9 8:00	BQ	AMRO	0	2	0	0			
1435	2020-4-10 8:00	BQ	AMRO	0	2	0	0			
1436	2020-4-11 8:00	BQ	AMRO	0	2	0	0			
1437	2020-4-12 8:00	BQ	AMRO	0	2	0	0			
1438	2020-4-13 8:00	BQ	AMRO	1	3	0	0			
1439	2020-4-14 8:00	BQ	AMRO	0	3	0	0			
1440	2020-4-15 8:00	BQ	AMRO	1	4	0	0			

Table S1 Daily confirmed cases and daily deaths of COVID-19 in 214 nations from WHO

ID	Date and time	Country or region	Continent	Confirmed patients	Cumulative Confirmed patients	Daily dead patients	Daily cumulative dead patients	Lag days	Daily CFR	3DMA CFR
1441	2020-4-16 8:00	BQ	AMRO	0	4	0	0			
1442	2020-4-17 8:00	BQ	AMRO	0	4	0	0			
1443	2020-4-18 8:00	BQ	AMRO	1	5	0	0			
1444	2020-4-19 8:00	BQ	AMRO	0	5	0	0			
1445	2020-4-20 8:00	BQ	AMRO	0	5	0	0			
1446	2020-4-21 8:00	BQ	AMRO	0	5	0	0			
1447	2020-4-22 8:00	BQ	AMRO	0	5	0	0			
1448	2020-4-23 8:00	BQ	AMRO	0	5	0	0			
1449	2020-4-24 8:00	BQ	AMRO	0	5	0	0			
1450	2020-4-25 8:00	BQ	AMRO	0	5	0	0			
1451	2020-4-26 8:00	BQ	AMRO	0	5	0	0			
1452	2020-4-27 8:00	BQ	AMRO	0	5	0	0			
1453	2020-4-28 8:00	BQ	AMRO	1	6	0	0			
1454	2020-4-29 8:00	BQ	AMRO	0	6	0	0			
1455	2020-4-30 8:00	BQ	AMRO	0	6	0	0			
1456	2020-5-1 8:00	BQ	AMRO	0	6	0	0			
1457	2020-5-2 8:00	BQ	AMRO	0	6	0	0			
1458										
1459	2020-3-5 8:00	BA	EURO	2	2	0	0			
1460	2020-3-6 8:00	BA	EURO	0	2	0	0			
1461	2020-3-7 8:00	BA	EURO	0	2	0	0			
1462	2020-3-8 8:00	BA	EURO	0	2	0	0			
1463	2020-3-9 8:00	BA	EURO	0	2	0	0			
1464	2020-3-10 8:00	BA	EURO	0	2	0	0			
1465	2020-3-11 8:00	BA	EURO	2	4	0	0			
1466	2020-3-12 8:00	BA	EURO	0	4	0	0			
1467	2020-3-13 8:00	BA	EURO	7	11	0	0			
1468	2020-3-14 8:00	BA	EURO	0	11	0	0			
1469	2020-3-15 8:00	BA	EURO	8	19	0	0			
1470	2020-3-16 8:00	BA	EURO	0	19	0	0			
1471	2020-3-17 8:00	BA	EURO	0	19	0	0			
1472	2020-3-18 8:00	BA	EURO	17	36	0	0			
1473	2020-3-19 8:00	BA	EURO	8	44	0	0			
1474	2020-3-20 8:00	BA	EURO	0	44	0	0			
1475	2020-3-21 8:00	BA	EURO	48	92	1	1	0	1.087	1.087
1476	2020-3-22 8:00	BA	EURO	0	92	0	1	1	1.087	0.991

Table S1 Daily confirmed cases and daily deaths of COVID-19 in 214 nations from WHO

ID	Date and time	Country or region	Continent	Confirmed patients	Cumulative Confirmed patients	Daily dead patients	Daily cumulative dead patients	Lag days	Daily CFR	3DMA CFR
1477	2020-3-23 8:00	BA	EURO	33	125	0	1	2	0.800	0.883
1478	2020-3-24 8:00	BA	EURO	6	131	0	1	3	0.763	0.928
1479	2020-3-25 8:00	BA	EURO	33	164	1	2	4	1.220	1.239
1480	2020-3-26 8:00	BA	EURO	9	173	1	3	5	1.734	1.454
1481	2020-3-27 8:00	BA	EURO	40	213	0	3	6	1.408	1.620
1482	2020-3-28 8:00	BA	EURO	20	233	1	4	7	1.717	1.785
1483	2020-3-29 8:00	BA	EURO	36	269	2	6	8	2.230	1.931
1484	2020-3-30 8:00	BA	EURO	56	325	0	6	9	1.846	2.195
1485	2020-3-31 8:00	BA	EURO	34	359	3	9	10	2.507	2.420
1486	2020-4-1 8:00	BA	EURO	54	413	3	12	11	2.906	2.738
1487	2020-4-2 8:00	BA	EURO	51	464	1	13	12	2.802	2.926
1488	2020-4-3 8:00	BA	EURO	57	521	3	16	13	3.071	2.981
1489	2020-4-4 8:00	BA	EURO	65	586	2	18	14	3.072	3.155
1490	2020-4-5 8:00	BA	EURO	46	632	3	21	15	3.323	3.189
1491	2020-4-6 8:00	BA	EURO	30	662	0	21	16	3.172	3.508
1492	2020-4-7 8:00	BA	EURO	33	695	7	28	17	4.029	3.766
1493	2020-4-8 8:00	BA	EURO	86	781	4	32	18	4.097	4.138
1494	2020-4-9 8:00	BA	EURO	35	816	3	35	19	4.289	4.167
1495	2020-4-10 8:00	BA	EURO	59	875	1	36	20	4.114	4.170
1496	2020-4-11 8:00	BA	EURO	26	901	1	37	21	4.107	4.041
1497	2020-4-12 8:00	BA	EURO	47	948	0	37	22	3.903	3.928
1498	2020-4-13 8:00	BA	EURO	59	1007	1	38	23	3.774	3.784
1499	2020-4-14 8:00	BA	EURO	27	1034	0	38	24	3.675	3.711
1500	2020-4-15 8:00	BA	EURO	52	1086	2	40	25	3.683	3.677
1501	2020-4-16 8:00	BA	EURO	30	1116	1	41	26	3.674	3.650
1502	2020-4-17 8:00	BA	EURO	53	1169	1	42	27	3.593	3.634
1503	2020-4-18 8:00	BA	EURO	41	1210	2	44	28	3.636	3.619
1504	2020-4-19 8:00	BA	EURO	58	1268	2	46	29	3.628	3.614
1505	2020-4-20 8:00	BA	EURO	18	1286	0	46	30	3.577	3.658
1506	2020-4-21 8:00	BA	EURO	14	1300	3	49	31	3.769	3.693
1507	2020-4-22 8:00	BA	EURO	40	1340	1	50	32	3.731	3.768
1508	2020-4-23 8:00	BA	EURO	27	1367	2	52	33	3.804	3.762
1509	2020-4-24 8:00	BA	EURO	46	1413	1	53	34	3.751	3.779
1510	2020-4-25 8:00	BA	EURO	15	1428	1	54	35	3.782	3.768
1511	2020-4-26 8:00	BA	EURO	57	1485	2	56	36	3.771	3.793
1512	2020-4-27 8:00	BA	EURO	31	1516	2	58	37	3.826	3.811

Table S1 Daily confirmed cases and daily deaths of COVID-19 in 214 nations from WHO

ID	Date and time	Country or region	Continent	Confirmed patients	Cumulative Confirmed patients	Daily dead patients	Daily cumulative dead patients	Lag days	Daily CFR	3DMA CFR
1513	2020-4-28 8:00	BA	EURO	48	1564	2	60	38	3.836	3.855
1514	2020-4-29 8:00	BA	EURO	24	1588	2	62	39	3.904	3.843
1515	2020-4-30 8:00	BA	EURO	101	1689	2	64	40	3.789	3.855
1516	2020-5-1 8:00	BA	EURO	68	1757	4	68	41	3.870	3.843
1517	2020-5-2 8:00	BA	EURO	0	1757	0	68	42	3.870	3.870
1518										
1519	2020-4-1 8:00	BW	AFRO	3	3	0	0			
1520	2020-4-2 8:00	BW	AFRO	0	3	1	1	0	33.333	29.167
1521	2020-4-3 8:00	BW	AFRO	1	4	0	1	0	25.000	27.778
1522	2020-4-4 8:00	BW	AFRO	0	4	0	1	0	25.000	25.000
1523	2020-4-5 8:00	BW	AFRO	0	4	0	1	0	25.000	25.000
1524	2020-4-6 8:00	BW	AFRO	0	4	0	1	0	25.000	22.222
1525	2020-4-7 8:00	BW	AFRO	2	6	0	1	0	16.667	19.444
1526	2020-4-8 8:00	BW	AFRO	0	6	0	1	0	16.667	16.667
1527	2020-4-9 8:00	BW	AFRO	0	6	0	1	0	16.667	13.675
1528	2020-4-10 8:00	BW	AFRO	7	13	0	1	0	7.692	10.684
1529	2020-4-11 8:00	BW	AFRO	0	13	0	1	0	7.692	7.692
1530	2020-4-12 8:00	BW	AFRO	0	13	0	1	0	7.692	7.692
1531	2020-4-13 8:00	BW	AFRO	0	13	0	1	0	7.692	7.692
1532	2020-4-14 8:00	BW	AFRO	0	13	0	1	0	7.692	7.692
1533	2020-4-15 8:00	BW	AFRO	0	13	0	1	0	7.692	7.692
1534	2020-4-16 8:00	BW	AFRO	0	13	0	1	0	7.692	7.350
1535	2020-4-17 8:00	BW	AFRO	2	15	0	1	0	6.667	7.009
1536	2020-4-18 8:00	BW	AFRO	0	15	0	1	0	6.667	6.667
1537	2020-4-19 8:00	BW	AFRO	0	15	0	1	0	6.667	6.111
1538	2020-4-20 8:00	BW	AFRO	5	20	0	1	0	5.000	5.556
1539	2020-4-21 8:00	BW	AFRO	0	20	0	1	0	5.000	5.000
1540	2020-4-22 8:00	BW	AFRO	0	20	0	1	0	5.000	4.848
1541	2020-4-23 8:00	BW	AFRO	2	22	0	1	0	4.545	4.697
1542	2020-4-24 8:00	BW	AFRO	0	22	0	1	0	4.545	4.545
1543	2020-4-25 8:00	BW	AFRO	0	22	0	1	0	4.545	4.545
1544	2020-4-26 8:00	BW	AFRO	0	22	0	1	0	4.545	4.545
1545	2020-4-27 8:00	BW	AFRO	0	22	0	1	0	4.545	4.545
1546	2020-4-28 8:00	BW	AFRO	0	22	0	1	0	4.545	4.480
1547	2020-4-29 8:00	BW	AFRO	1	23	0	1	0	4.348	4.414
1548	2020-4-30 8:00	BW	AFRO	0	23	0	1	0	4.348	4.348

Table S1 Daily confirmed cases and daily deaths of COVID-19 in 214 nations from WHO

ID	Date and time	Country or region	Continent	Confirmed patients	Cumulative Confirmed patients	Daily dead patients	Daily cumulative dead patients	Lag days	Daily CFR	3DMA CFR
1549	2020-5-1 8:00	BW	AFRO	0	23	0	1	0	4.348	4.348
1550	2020-5-2 8:00	BW	AFRO	0	23	0	1	0	4.348	4.348
1551										
1552	2020-2-26 8:00	BR	AMRO	1	1	0	0			
1553	2020-2-27 8:00	BR	AMRO	0	1	0	0			
1554	2020-2-28 8:00	BR	AMRO	0	1	0	0			
1555	2020-2-29 8:00	BR	AMRO	0	1	0	0			
1556	2020-3-1 8:00	BR	AMRO	1	2	0	0			
1557	2020-3-2 8:00	BR	AMRO	0	2	0	0			
1558	2020-3-3 8:00	BR	AMRO	0	2	0	0			
1559	2020-3-4 8:00	BR	AMRO	1	3	0	0			
1560	2020-3-5 8:00	BR	AMRO	4	7	0	0			
1561	2020-3-6 8:00	BR	AMRO	1	8	0	0			
1562	2020-3-7 8:00	BR	AMRO	11	19	0	0			
1563	2020-3-8 8:00	BR	AMRO	0	19	0	0			
1564	2020-3-9 8:00	BR	AMRO	6	25	0	0			
1565	2020-3-10 8:00	BR	AMRO	0	25	0	0			
1566	2020-3-11 8:00	BR	AMRO	9	34	0	0			
1567	2020-3-12 8:00	BR	AMRO	18	52	0	0			
1568	2020-3-13 8:00	BR	AMRO	46	98	0	0			
1569	2020-3-14 8:00	BR	AMRO	23	121	0	0			
1570	2020-3-15 8:00	BR	AMRO	0	121	0	0			
1571	2020-3-16 8:00	BR	AMRO	79	200	0	0			
1572	2020-3-17 8:00	BR	AMRO	34	234	0	0			
1573	2020-3-18 8:00	BR	AMRO	57	291	1	1	0	0.344	0.639
1574	2020-3-19 8:00	BR	AMRO	137	428	3	4	1	0.935	0.641
1575	2020-3-20 8:00	BR	AMRO	193	621	0	4	2	0.644	0.932
1576	2020-3-21 8:00	BR	AMRO	283	904	7	11	3	1.217	1.026
1577	2020-3-22 8:00	BR	AMRO	0	904	0	11	4	1.217	1.217
1578	2020-3-23 8:00	BR	AMRO	0	904	0	11	5	1.217	1.508
1579	2020-3-24 8:00	BR	AMRO	1297	2201	35	46	6	2.090	1.799
1580	2020-3-25 8:00	BR	AMRO	0	2201	0	46	7	2.090	2.174
1581	2020-3-26 8:00	BR	AMRO	232	2433	11	57	8	2.343	2.259
1582	2020-3-27 8:00	BR	AMRO	0	2433	0	57	9	2.343	2.442
1583	2020-3-28 8:00	BR	AMRO	482	2915	20	77	10	2.642	2.559
1584	2020-3-29 8:00	BR	AMRO	502	3417	15	92	11	2.692	2.751

Table S1 Daily confirmed cases and daily deaths of COVID-19 in 214 nations from WHO

ID	Date and time	Country or region	Continent	Confirmed patients	Cumulative Confirmed patients	Daily dead patients	Daily cumulative dead patients	Lag days	Daily CFR	3DMA CFR
1585	2020-3-30 8:00	BR	AMRO	487	3904	22	114	12	2.920	2.936
1586	2020-3-31 8:00	BR	AMRO	352	4256	22	136	13	3.195	3.196
1587	2020-4-1 8:00	BR	AMRO	323	4579	23	159	14	3.472	3.395
1588	2020-4-2 8:00	BR	AMRO	1138	5717	42	201	15	3.516	3.505
1589	2020-4-3 8:00	BR	AMRO	1119	6836	40	241	16	3.525	3.607
1590	2020-4-4 8:00	BR	AMRO	1074	7910	58	299	17	3.780	3.757
1591	2020-4-5 8:00	BR	AMRO	1146	9056	60	359	18	3.964	3.982
1592	2020-4-6 8:00	BR	AMRO	1222	10278	73	432	19	4.203	4.178
1593	2020-4-7 8:00	BR	AMRO	852	11130	54	486	20	4.367	4.386
1594	2020-4-8 8:00	BR	AMRO	926	12056	67	553	21	4.587	4.605
1595	2020-4-9 8:00	BR	AMRO	1661	13717	114	667	22	4.863	4.824
1596	2020-4-10 8:00	BR	AMRO	2210	15927	133	800	23	5.023	5.052
1597	2020-4-11 8:00	BR	AMRO	1930	17857	141	941	24	5.270	5.223
1598	2020-4-12 8:00	BR	AMRO	1781	19638	115	1056	25	5.377	5.357
1599	2020-4-13 8:00	BR	AMRO	1089	20727	68	1124	26	5.423	5.439
1600	2020-4-14 8:00	BR	AMRO	1442	22169	99	1223	27	5.517	5.536
1601	2020-4-15 8:00	BR	AMRO	1261	23430	105	1328	28	5.668	5.750
1602	2020-4-16 8:00	BR	AMRO	1832	25262	204	1532	29	6.064	5.954
1603	2020-4-17 8:00	BR	AMRO	3058	28320	204	1736	30	6.130	6.173
1604	2020-4-18 8:00	BR	AMRO	2105	30425	188	1924	31	6.324	6.270
1605	2020-4-19 8:00	BR	AMRO	3257	33682	217	2141	32	6.357	6.364
1606	2020-4-20 8:00	BR	AMRO	2917	36599	206	2347	33	6.413	6.380
1607	2020-4-21 8:00	BR	AMRO	2055	38654	115	2462	34	6.369	6.376
1608	2020-4-22 8:00	BR	AMRO	1927	40581	113	2575	35	6.345	6.359
1609	2020-4-23 8:00	BR	AMRO	2498	43079	166	2741	36	6.363	6.353
1610	2020-4-24 8:00	BR	AMRO	2678	45757	165	2906	37	6.351	6.469
1611	2020-4-25 8:00	BR	AMRO	3735	49492	407	3313	38	6.694	6.657
1612	2020-4-26 8:00	BR	AMRO	3503	52995	357	3670	39	6.925	6.828
1613	2020-4-27 8:00	BR	AMRO	5514	58509	346	4016	40	6.864	6.861
1614	2020-4-28 8:00	BR	AMRO	3379	61888	189	4205	41	6.795	6.830
1615	2020-4-29 8:00	BR	AMRO	4613	66501	338	4543	42	6.831	6.868
1616	2020-4-30 8:00	BR	AMRO	5385	71886	474	5017	43	6.979	6.935
1617	2020-5-1 8:00	BR	AMRO	6276	78162	449	5466	44	6.993	6.961
1618	2020-5-2 8:00	BR	AMRO	7218	85380	435	5901	45	6.911	6.952
1619										
1620	2020-3-26 8:00	VG	AMRO	2	2	0	0			

Table S1 Daily confirmed cases and daily deaths of COVID-19 in 214 nations from WHO

ID	Date and time	Country or region	Continent	Confirmed patients	Cumulative Confirmed patients	Daily dead patients	Daily cumulative dead patients	Lag days	Daily CFR	3DMA CFR
1621	2020-3-27 8:00	VG	AMRO	0	2	0	0			
1622	2020-3-28 8:00	VG	AMRO	0	2	0	0			
1623	2020-3-29 8:00	VG	AMRO	0	2	0	0			
1624	2020-3-30 8:00	VG	AMRO	0	2	0	0			
1625	2020-3-31 8:00	VG	AMRO	0	2	0	0			
1626	2020-4-1 8:00	VG	AMRO	1	3	0	0			
1627	2020-4-2 8:00	VG	AMRO	0	3	0	0			
1628	2020-4-3 8:00	VG	AMRO	0	3	0	0			
1629	2020-4-4 8:00	VG	AMRO	0	3	0	0			
1630	2020-4-5 8:00	VG	AMRO	0	3	0	0			
1631	2020-4-6 8:00	VG	AMRO	0	3	0	0			
1632	2020-4-7 8:00	VG	AMRO	0	3	0	0			
1633	2020-4-8 8:00	VG	AMRO	0	3	0	0			
1634	2020-4-9 8:00	VG	AMRO	0	3	0	0			
1635	2020-4-10 8:00	VG	AMRO	0	3	0	0			
1636	2020-4-11 8:00	VG	AMRO	0	3	0	0			
1637	2020-4-12 8:00	VG	AMRO	0	3	0	0			
1638	2020-4-13 8:00	VG	AMRO	0	3	0	0			
1639	2020-4-14 8:00	VG	AMRO	0	3	0	0			
1640	2020-4-15 8:00	VG	AMRO	0	3	0	0			
1641	2020-4-16 8:00	VG	AMRO	0	3	0	0			
1642	2020-4-17 8:00	VG	AMRO	0	3	0	0			
1643	2020-4-18 8:00	VG	AMRO	0	3	0	0			
1644	2020-4-19 8:00	VG	AMRO	0	3	0	0			
1645	2020-4-20 8:00	VG	AMRO	1	4	1	1	0	25.000	25.000
1646	2020-4-21 8:00	VG	AMRO	0	4	0	1	1	25.000	25.000
1647	2020-4-22 8:00	VG	AMRO	0	4	0	1	2	25.000	25.000
1648	2020-4-23 8:00	VG	AMRO	0	4	0	1	3	25.000	23.333
1649	2020-4-24 8:00	VG	AMRO	1	5	0	1	4	20.000	21.667
1650	2020-4-25 8:00	VG	AMRO	0	5	0	1	5	20.000	18.889
1651	2020-4-26 8:00	VG	AMRO	1	6	0	1	6	16.667	17.778
1652	2020-4-27 8:00	VG	AMRO	0	6	0	1	7	16.667	16.667
1653	2020-4-28 8:00	VG	AMRO	0	6	0	1	8	16.667	16.667
1654	2020-4-29 8:00	VG	AMRO	0	6	0	1	9	16.667	16.667
1655	2020-4-30 8:00	VG	AMRO	0	6	0	1	10	16.667	16.667
1656	2020-5-1 8:00	VG	AMRO	0	6	0	1	11	16.667	16.667

Table S1 Daily confirmed cases and daily deaths of COVID-19 in 214 nations from WHO

ID	Date and time	Country or region	Continent	Confirmed patients	Cumulative Confirmed patients	Daily dead patients	Daily cumulative dead patients	Lag days	Daily CFR	3DMA CFR
1657	2020-5-2 8:00	VG	AMRO	0	6	0	1	12	16.667	16.667
1658										
1659	2020-3-10 8:00	BN	WPRO	1	1	0	0			
1660	2020-3-11 8:00	BN	WPRO	11	12	0	0			
1661	2020-3-12 8:00	BN	WPRO	0	12	0	0			
1662	2020-3-13 8:00	BN	WPRO	0	12	0	0			
1663	2020-3-14 8:00	BN	WPRO	13	25	0	0			
1664	2020-3-15 8:00	BN	WPRO	15	40	0	0			
1665	2020-3-16 8:00	BN	WPRO	10	50	0	0			
1666	2020-3-17 8:00	BN	WPRO	4	54	0	0			
1667	2020-3-18 8:00	BN	WPRO	2	56	0	0			
1668	2020-3-19 8:00	BN	WPRO	17	73	0	0			
1669	2020-3-20 8:00	BN	WPRO	5	78	0	0			
1670	2020-3-21 8:00	BN	WPRO	5	83	0	0			
1671	2020-3-22 8:00	BN	WPRO	0	83	0	0			
1672	2020-3-23 8:00	BN	WPRO	5	88	0	0			
1673	2020-3-24 8:00	BN	WPRO	3	91	0	0			
1674	2020-3-25 8:00	BN	WPRO	13	104	0	0			
1675	2020-3-26 8:00	BN	WPRO	5	109	0	0			
1676	2020-3-27 8:00	BN	WPRO	5	114	0	0			
1677	2020-3-28 8:00	BN	WPRO	1	115	1	1	0	0.870	0.851
1678	2020-3-29 8:00	BN	WPRO	5	120	0	1	1	0.833	0.832
1679	2020-3-30 8:00	BN	WPRO	6	126	0	1	2	0.794	0.805
1680	2020-3-31 8:00	BN	WPRO	1	127	0	1	3	0.787	0.785
1681	2020-4-1 8:00	BN	WPRO	2	129	0	1	4	0.775	0.775
1682	2020-4-2 8:00	BN	WPRO	2	131	0	1	5	0.763	0.763
1683	2020-4-3 8:00	BN	WPRO	2	133	0	1	6	0.752	0.754
1684	2020-4-4 8:00	BN	WPRO	1	134	0	1	7	0.746	0.746
1685	2020-4-5 8:00	BN	WPRO	1	135	0	1	8	0.741	0.743
1686	2020-4-6 8:00	BN	WPRO	0	135	0	1	9	0.741	0.741
1687	2020-4-7 8:00	BN	WPRO	0	135	0	1	10	0.741	0.741
1688	2020-4-8 8:00	BN	WPRO	0	135	0	1	11	0.741	0.741
1689	2020-4-9 8:00	BN	WPRO	0	135	0	1	12	0.741	0.741
1690	2020-4-10 8:00	BN	WPRO	0	135	0	1	13	0.741	0.739
1691	2020-4-11 8:00	BN	WPRO	1	136	0	1	14	0.735	0.737
1692	2020-4-12 8:00	BN	WPRO	0	136	0	1	15	0.735	0.735

Table S1 Daily confirmed cases and daily deaths of COVID-19 in 214 nations from WHO

ID	Date and time	Country or region	Continent	Confirmed patients	Cumulative Confirmed patients	Daily dead patients	Daily cumulative dead patients	Lag days	Daily CFR	3DMA CFR
1693	2020-4-13 8:00	BN	WPRO	0	136	0	1	16	0.735	0.735
1694	2020-4-14 8:00	BN	WPRO	0	136	0	1	17	0.735	0.735
1695	2020-4-15 8:00	BN	WPRO	0	136	0	1	18	0.735	0.735
1696	2020-4-16 8:00	BN	WPRO	0	136	0	1	19	0.735	0.735
1697	2020-4-17 8:00	BN	WPRO	0	136	0	1	20	0.735	0.735
1698	2020-4-18 8:00	BN	WPRO	0	136	0	1	21	0.735	0.734
1699	2020-4-19 8:00	BN	WPRO	1	137	0	1	22	0.730	0.730
1700	2020-4-20 8:00	BN	WPRO	1	138	0	1	23	0.725	0.726
1701	2020-4-21 8:00	BN	WPRO	0	138	0	1	24	0.725	0.725
1702	2020-4-22 8:00	BN	WPRO	0	138	0	1	25	0.725	0.725
1703	2020-4-23 8:00	BN	WPRO	0	138	0	1	26	0.725	0.725
1704	2020-4-24 8:00	BN	WPRO	0	138	0	1	27	0.725	0.725
1705	2020-4-25 8:00	BN	WPRO	0	138	0	1	28	0.725	0.725
1706	2020-4-26 8:00	BN	WPRO	0	138	0	1	29	0.725	0.725
1707	2020-4-27 8:00	BN	WPRO	0	138	0	1	30	0.725	0.725
1708	2020-4-28 8:00	BN	WPRO	0	138	0	1	31	0.725	0.725
1709	2020-4-29 8:00	BN	WPRO	0	138	0	1	32	0.725	0.725
1710	2020-4-30 8:00	BN	WPRO	0	138	0	1	33	0.725	0.725
1711	2020-5-1 8:00	BN	WPRO	0	138	0	1	34	0.725	0.725
1712	2020-5-2 8:00	BN	WPRO	0	138	0	1	35	0.725	0.725
1713										
1714	2020-3-8 8:00	BG	EURO	4	4	0	0			
1715	2020-3-9 8:00	BG	EURO	0	4	0	0			
1716	2020-3-10 8:00	BG	EURO	0	4	0	0			
1717	2020-3-11 8:00	BG	EURO	3	7	0	0			
1718	2020-3-12 8:00	BG	EURO	0	7	1	1	0	14.286	14.286
1719	2020-3-13 8:00	BG	EURO	0	7	0	1	1	14.286	14.286
1720	2020-3-14 8:00	BG	EURO	0	7	0	1	2	14.286	10.831
1721	2020-3-15 8:00	BG	EURO	44	51	1	2	3	3.922	7.064
1722	2020-3-16 8:00	BG	EURO	16	67	0	2	4	2.985	3.125
1723	2020-3-17 8:00	BG	EURO	14	81	0	2	5	2.469	2.543
1724	2020-3-18 8:00	BG	EURO	11	92	0	2	6	2.174	2.612
1725	2020-3-19 8:00	BG	EURO	2	94	1	3	7	3.191	2.576
1726	2020-3-20 8:00	BG	EURO	33	127	0	3	8	2.362	2.465
1727	2020-3-21 8:00	BG	EURO	36	163	0	3	9	1.840	2.014
1728	2020-3-22 8:00	BG	EURO	0	163	0	3	10	1.840	1.768

Table S1 Daily confirmed cases and daily deaths of COVID-19 in 214 nations from WHO

ID	Date and time	Country or region	Continent	Confirmed patients	Cumulative Confirmed patients	Daily dead patients	Daily cumulative dead patients	Lag days	Daily CFR	3DMA CFR
1729	2020-3-23 8:00	BG	EURO	22	185	0	3	11	1.622	1.652
1730	2020-3-24 8:00	BG	EURO	16	201	0	3	12	1.493	1.493
1731	2020-3-25 8:00	BG	EURO	19	220	0	3	13	1.364	1.365
1732	2020-3-26 8:00	BG	EURO	22	242	0	3	14	1.240	1.247
1733	2020-3-27 8:00	BG	EURO	22	264	0	3	15	1.136	1.133
1734	2020-3-28 8:00	BG	EURO	29	293	0	3	16	1.024	1.425
1735	2020-3-29 8:00	BG	EURO	38	331	4	7	17	2.115	1.817
1736	2020-3-30 8:00	BG	EURO	15	346	1	8	18	2.312	2.218
1737	2020-3-31 8:00	BG	EURO	13	359	0	8	19	2.228	2.182
1738	2020-4-1 8:00	BG	EURO	40	399	0	8	20	2.005	2.201
1739	2020-4-2 8:00	BG	EURO	23	422	2	10	21	2.370	2.188
1740	2020-4-3 8:00	BG	EURO	35	457	0	10	22	2.188	2.481
1741	2020-4-4 8:00	BG	EURO	28	485	4	14	23	2.887	2.818
1742	2020-4-5 8:00	BG	EURO	18	503	3	17	24	3.380	3.344
1743	2020-4-6 8:00	BG	EURO	28	531	3	20	25	3.766	3.718
1744	2020-4-7 8:00	BG	EURO	18	549	2	22	26	4.007	3.920
1745	2020-4-8 8:00	BG	EURO	28	577	1	23	27	3.986	4.014
1746	2020-4-9 8:00	BG	EURO	16	593	1	24	28	4.047	3.960
1747	2020-4-10 8:00	BG	EURO	31	624	0	24	29	3.846	3.943
1748	2020-4-11 8:00	BG	EURO	11	635	1	25	30	3.937	4.006
1749	2020-4-12 8:00	BG	EURO	26	661	3	28	31	4.236	4.156
1750	2020-4-13 8:00	BG	EURO	14	675	1	29	32	4.296	4.401
1751	2020-4-14 8:00	BG	EURO	10	685	3	32	33	4.672	4.626
1752	2020-4-15 8:00	BG	EURO	28	713	3	35	34	4.909	4.800
1753	2020-4-16 8:00	BG	EURO	34	747	1	36	35	4.819	4.826
1754	2020-4-17 8:00	BG	EURO	53	800	2	38	36	4.750	4.805
1755	2020-4-18 8:00	BG	EURO	46	846	3	41	37	4.846	4.755
1756	2020-4-19 8:00	BG	EURO	32	878	0	41	38	4.670	4.738
1757	2020-4-20 8:00	BG	EURO	37	915	2	43	39	4.699	4.666
1758	2020-4-21 8:00	BG	EURO	14	929	0	43	40	4.629	4.648
1759	2020-4-22 8:00	BG	EURO	46	975	2	45	41	4.615	4.676
1760	2020-4-23 8:00	BG	EURO	49	1024	4	49	42	4.785	4.714
1761	2020-4-24 8:00	BG	EURO	73	1097	3	52	43	4.740	4.690
1762	2020-4-25 8:00	BG	EURO	91	1188	2	54	44	4.545	4.565
1763	2020-4-26 8:00	BG	EURO	59	1247	1	55	45	4.411	4.421
1764	2020-4-27 8:00	BG	EURO	53	1300	1	56	46	4.308	4.325

Table S1 Daily confirmed cases and daily deaths of COVID-19 in 214 nations from WHO

ID	Date and time	Country or region	Continent	Confirmed patients	Cumulative Confirmed patients	Daily dead patients	Daily cumulative dead patients	Lag days	Daily CFR	3DMA CFR
1765	2020-4-28 8:00	BG	EURO	63	1363	2	58	47	4.255	4.236
1766	2020-4-29 8:00	BG	EURO	36	1399	0	58	48	4.146	4.275
1767	2020-4-30 8:00	BG	EURO	48	1447	6	64	49	4.423	4.317
1768	2020-5-1 8:00	BG	EURO	59	1506	2	66	50	4.382	4.384
1769	2020-5-2 8:00	BG	EURO	82	1588	3	69	51	4.345	4.364
1770										
1771	2020-3-11 8:00	BF	AFRO	2	2	0	0			
1772	2020-3-12 8:00	BF	AFRO	0	2	0	0			
1773	2020-3-13 8:00	BF	AFRO	0	2	0	0			
1774	2020-3-14 8:00	BF	AFRO	1	3	0	0			
1775	2020-3-15 8:00	BF	AFRO	0	3	0	0			
1776	2020-3-16 8:00	BF	AFRO	12	15	0	0			
1777	2020-3-17 8:00	BF	AFRO	5	20	0	0			
1778	2020-3-18 8:00	BF	AFRO	6	26	1	1	0	3.846	3.173
1779	2020-3-19 8:00	BF	AFRO	14	40	0	1	1	2.500	2.949
1780	2020-3-20 8:00	BF	AFRO	0	40	0	1	2	2.500	3.056
1781	2020-3-21 8:00	BF	AFRO	32	72	2	3	3	4.167	3.556
1782	2020-3-22 8:00	BF	AFRO	3	75	0	3	4	4.000	4.056
1783	2020-3-23 8:00	BF	AFRO	0	75	0	3	5	4.000	3.677
1784	2020-3-24 8:00	BF	AFRO	24	99	0	3	6	3.030	3.221
1785	2020-3-25 8:00	BF	AFRO	15	114	0	3	7	2.632	2.572
1786	2020-3-26 8:00	BF	AFRO	32	146	0	3	8	2.055	2.247
1787	2020-3-27 8:00	BF	AFRO	0	146	0	3	9	2.055	2.055
1788	2020-3-28 8:00	BF	AFRO	0	146	0	3	10	2.055	2.055
1789	2020-3-29 8:00	BF	AFRO	0	146	0	3	11	2.055	3.172
1790	2020-3-30 8:00	BF	AFRO	76	222	9	12	12	5.405	4.113
1791	2020-3-31 8:00	BF	AFRO	24	246	0	12	13	4.878	5.216
1792	2020-4-1 8:00	BF	AFRO	15	261	2	14	14	5.364	5.202
1793	2020-4-2 8:00	BF	AFRO	0	261	0	14	15	5.364	5.492
1794	2020-4-3 8:00	BF	AFRO	0	261	1	15	16	5.747	5.359
1795	2020-4-4 8:00	BF	AFRO	41	302	0	15	17	4.967	5.227
1796	2020-4-5 8:00	BF	AFRO	0	302	0	15	18	4.967	4.954
1797	2020-4-6 8:00	BF	AFRO	43	345	2	17	19	4.928	4.941
1798	2020-4-7 8:00	BF	AFRO	0	345	0	17	20	4.928	4.848
1799	2020-4-8 8:00	BF	AFRO	39	384	1	18	21	4.688	4.854
1800	2020-4-9 8:00	BF	AFRO	0	384	1	19	22	4.948	4.742

Table S1 Daily confirmed cases and daily deaths of COVID-19 in 214 nations from WHO

ID	Date and time	Country or region	Continent	Confirmed patients	Cumulative Confirmed patients	Daily dead patients	Daily cumulative dead patients	Lag days	Daily CFR	3DMA CFR
1801	2020-4-10 8:00	BF	AFRO	30	414	0	19	23	4.589	4.609
1802	2020-4-11 8:00	BF	AFRO	29	443	0	19	24	4.289	4.389
1803	2020-4-12 8:00	BF	AFRO	0	443	0	19	25	4.289	4.289
1804	2020-4-13 8:00	BF	AFRO	0	443	0	19	26	4.289	4.670
1805	2020-4-14 8:00	BF	AFRO	54	497	8	27	27	5.433	5.053
1806	2020-4-15 8:00	BF	AFRO	18	515	1	28	28	5.437	5.391
1807	2020-4-16 8:00	BF	AFRO	13	528	0	28	29	5.303	5.544
1808	2020-4-17 8:00	BF	AFRO	15	543	4	32	30	5.893	5.682
1809	2020-4-18 8:00	BF	AFRO	4	547	0	32	31	5.850	5.864
1810	2020-4-19 8:00	BF	AFRO	0	547	0	32	32	5.850	6.024
1811	2020-4-20 8:00	BF	AFRO	18	565	4	36	33	6.372	6.157
1812	2020-4-21 8:00	BF	AFRO	11	576	0	36	34	6.250	6.387
1813	2020-4-22 8:00	BF	AFRO	5	581	2	38	35	6.540	6.375
1814	2020-4-23 8:00	BF	AFRO	19	600	0	38	36	6.333	6.402
1815	2020-4-24 8:00	BF	AFRO	0	600	0	38	37	6.333	6.441
1816	2020-4-25 8:00	BF	AFRO	16	616	3	41	38	6.656	6.502
1817	2020-4-26 8:00	BF	AFRO	13	629	0	41	39	6.518	6.607
1818	2020-4-27 8:00	BF	AFRO	3	632	1	42	40	6.646	6.603
1819	2020-4-28 8:00	BF	AFRO	0	632	0	42	41	6.646	6.625
1820	2020-4-29 8:00	BF	AFRO	6	638	0	42	42	6.583	6.604
1821	2020-4-30 8:00	BF	AFRO	0	638	0	42	43	6.583	6.611
1822	2020-5-1 8:00	BF	AFRO	7	645	1	43	44	6.667	6.676
1823	2020-5-2 8:00	BF	AFRO	4	649	1	44	45	6.780	6.723
1824										
1825	2020-4-1 8:00	BI	AFRO	2	2	0	0			
1826	2020-4-2 8:00	BI	AFRO	0	2	0	0			
1827	2020-4-3 8:00	BI	AFRO	1	3	0	0			
1828	2020-4-4 8:00	BI	AFRO	0	3	0	0			
1829	2020-4-5 8:00	BI	AFRO	0	3	0	0			
1830	2020-4-6 8:00	BI	AFRO	0	3	0	0			
1831	2020-4-7 8:00	BI	AFRO	0	3	0	0			
1832	2020-4-8 8:00	BI	AFRO	0	3	0	0			
1833	2020-4-9 8:00	BI	AFRO	0	3	0	0			
1834	2020-4-10 8:00	BI	AFRO	0	3	0	0			
1835	2020-4-11 8:00	BI	AFRO	0	3	0	0			
1836	2020-4-12 8:00	BI	AFRO	0	3	0	0			

Table S1 Daily confirmed cases and daily deaths of COVID-19 in 214 nations from WHO

ID	Date and time	Country or region	Continent	Confirmed patients	Cumulative Confirmed patients	Daily dead patients	Daily cumulative dead patients	Lag days	Daily CFR	3DMA CFR
1837	2020-4-13 8:00	BI	AFRO	2	5	0	0			
1838	2020-4-14 8:00	BI	AFRO	0	5	0	0			
1839	2020-4-15 8:00	BI	AFRO	0	5	0	0			
1840	2020-4-16 8:00	BI	AFRO	0	5	0	0			
1841	2020-4-17 8:00	BI	AFRO	0	5	0	0			
1842	2020-4-18 8:00	BI	AFRO	0	5	0	0			
1843	2020-4-19 8:00	BI	AFRO	0	5	0	0			
1844	2020-4-20 8:00	BI	AFRO	1	6	1	1	0	16.667	16.667
1845	2020-4-21 8:00	BI	AFRO	0	6	0	1	1	16.667	14.141
1846	2020-4-22 8:00	BI	AFRO	5	11	0	1	2	9.091	11.616
1847	2020-4-23 8:00	BI	AFRO	0	11	0	1	3	9.091	9.091
1848	2020-4-24 8:00	BI	AFRO	0	11	0	1	4	9.091	8.838
1849	2020-4-25 8:00	BI	AFRO	1	12	0	1	5	8.333	8.586
1850	2020-4-26 8:00	BI	AFRO	0	12	0	1	6	8.333	7.778
1851	2020-4-27 8:00	BI	AFRO	3	15	0	1	7	6.667	7.222
1852	2020-4-28 8:00	BI	AFRO	0	15	0	1	8	6.667	6.667
1853	2020-4-29 8:00	BI	AFRO	0	15	0	1	9	6.667	6.667
1854	2020-4-30 8:00	BI	AFRO	0	15	0	1	10	6.667	6.667
1855	2020-5-1 8:00	BI	AFRO	0	15	0	1	11	6.667	6.667
1856	2020-5-2 8:00	BI	AFRO	0	15	0	1	12	6.667	6.667
1857										
1858	2020-3-20 8:00	CV	AFRO	1	1	0	0			
1859	2020-3-21 8:00	CV	AFRO	2	3	0	0			
1860	2020-3-22 8:00	CV	AFRO	0	3	0	0			
1861	2020-3-23 8:00	CV	AFRO	0	3	0	0			
1862	2020-3-24 8:00	CV	AFRO	0	3	0	0			
1863	2020-3-25 8:00	CV	AFRO	0	3	0	0			
1864	2020-3-26 8:00	CV	AFRO	0	3	0	0			
1865	2020-3-27 8:00	CV	AFRO	0	3	0	0			
1866	2020-3-28 8:00	CV	AFRO	2	5	1	1	0	20.000	20.000
1867	2020-3-29 8:00	CV	AFRO	0	5	0	1	1	20.000	20.000
1868	2020-3-30 8:00	CV	AFRO	0	5	0	1	2	20.000	20.000
1869	2020-3-31 8:00	CV	AFRO	0	5	0	1	3	20.000	20.000
1870	2020-4-1 8:00	CV	AFRO	0	5	0	1	4	20.000	20.000
1871	2020-4-2 8:00	CV	AFRO	0	5	0	1	5	20.000	20.000
1872	2020-4-3 8:00	CV	AFRO	0	5	0	1	6	20.000	20.000

Table S1 Daily confirmed cases and daily deaths of COVID-19 in 214 nations from WHO

ID	Date and time	Country or region	Continent	Confirmed patients	Cumulative Confirmed patients	Daily dead patients	Daily cumulative dead patients	Lag days	Daily CFR	3DMA CFR
1873	2020-4-4 8:00	CV	AFRO	0	5	0	1	7	20.000	20.000
1874	2020-4-5 8:00	CV	AFRO	0	5	0	1	8	20.000	20.000
1875	2020-4-6 8:00	CV	AFRO	0	5	0	1	9	20.000	18.095
1876	2020-4-7 8:00	CV	AFRO	2	7	0	1	10	14.286	16.190
1877	2020-4-8 8:00	CV	AFRO	0	7	0	1	11	14.286	14.286
1878	2020-4-9 8:00	CV	AFRO	0	7	0	1	12	14.286	14.286
1879	2020-4-10 8:00	CV	AFRO	0	7	0	1	13	14.286	14.286
1880	2020-4-11 8:00	CV	AFRO	0	7	0	1	14	14.286	14.286
1881	2020-4-12 8:00	CV	AFRO	0	7	0	1	15	14.286	14.286
1882	2020-4-13 8:00	CV	AFRO	0	7	0	1	16	14.286	12.857
1883	2020-4-14 8:00	CV	AFRO	3	10	0	1	17	10.000	11.429
1884	2020-4-15 8:00	CV	AFRO	0	10	0	1	18	10.000	10.000
1885	2020-4-16 8:00	CV	AFRO	0	10	0	1	19	10.000	7.273
1886	2020-4-17 8:00	CV	AFRO	45	55	0	1	20	1.818	4.545
1887	2020-4-18 8:00	CV	AFRO	0	55	0	1	21	1.818	1.818
1888	2020-4-19 8:00	CV	AFRO	0	55	0	1	22	1.818	1.818
1889	2020-4-20 8:00	CV	AFRO	0	55	0	1	23	1.818	1.818
1890	2020-4-21 8:00	CV	AFRO	0	55	0	1	24	1.818	1.710
1891	2020-4-22 8:00	CV	AFRO	12	67	0	1	25	1.493	1.601
1892	2020-4-23 8:00	CV	AFRO	0	67	0	1	26	1.493	1.493
1893	2020-4-24 8:00	CV	AFRO	0	67	0	1	27	1.493	1.374
1894	2020-4-25 8:00	CV	AFRO	21	88	0	1	28	1.136	1.247
1895	2020-4-26 8:00	CV	AFRO	2	90	0	1	29	1.111	1.064
1896	2020-4-27 8:00	CV	AFRO	16	106	0	1	30	0.943	0.999
1897	2020-4-28 8:00	CV	AFRO	0	106	0	1	31	0.943	0.924
1898	2020-4-29 8:00	CV	AFRO	7	113	0	1	32	0.885	0.904
1899	2020-4-30 8:00	CV	AFRO	0	113	0	1	33	0.885	0.865
1900	2020-5-1 8:00	CV	AFRO	8	121	0	1	34	0.826	0.846
1901	2020-5-2 8:00	CV	AFRO	0	121	0	1	35	0.826	0.826
1902										
1903	2020-1-27 8:00	KH	WPRO	1	1	0	0			
1904	2020-1-28 8:00	KH	WPRO	0	1	0	0			
1905	2020-1-29 8:00	KH	WPRO	0	1	0	0			
1906	2020-1-30 8:00	KH	WPRO	0	1	0	0			
1907	2020-1-31 8:00	KH	WPRO	0	1	0	0			
1908	2020-2-1 8:00	KH	WPRO	0	1	0	0			

Table S1 Daily confirmed cases and daily deaths of COVID-19 in 214 nations from WHO

ID	Date and time	Country or region	Continent	Confirmed patients	Cumulative Confirmed patients	Daily dead patients	Daily cumulative dead patients	Lag days	Daily CFR	3DMA CFR
1909	2020-2-2 8:00	KH	WPRO	0	1	0	0			
1910	2020-2-3 8:00	KH	WPRO	0	1	0	0			
1911	2020-2-4 8:00	KH	WPRO	0	1	0	0			
1912	2020-2-5 8:00	KH	WPRO	0	1	0	0			
1913	2020-2-6 8:00	KH	WPRO	0	1	0	0			
1914	2020-2-7 8:00	KH	WPRO	0	1	0	0			
1915	2020-2-8 8:00	KH	WPRO	0	1	0	0			
1916	2020-2-9 8:00	KH	WPRO	0	1	0	0			
1917	2020-2-10 8:00	KH	WPRO	0	1	0	0			
1918	2020-2-11 8:00	KH	WPRO	0	1	0	0			
1919	2020-2-12 8:00	KH	WPRO	0	1	0	0			
1920	2020-2-13 8:00	KH	WPRO	0	1	0	0			
1921	2020-2-14 8:00	KH	WPRO	0	1	0	0			
1922	2020-2-15 8:00	KH	WPRO	0	1	0	0			
1923	2020-2-16 8:00	KH	WPRO	0	1	0	0			
1924	2020-2-17 8:00	KH	WPRO	0	1	0	0			
1925	2020-2-18 8:00	KH	WPRO	0	1	0	0			
1926	2020-2-19 8:00	KH	WPRO	0	1	0	0			
1927	2020-2-20 8:00	KH	WPRO	0	1	0	0			
1928	2020-2-21 8:00	KH	WPRO	0	1	0	0			
1929	2020-2-22 8:00	KH	WPRO	0	1	0	0			
1930	2020-2-23 8:00	KH	WPRO	0	1	0	0			
1931	2020-2-24 8:00	KH	WPRO	0	1	0	0			
1932	2020-2-25 8:00	KH	WPRO	0	1	0	0			
1933	2020-2-26 8:00	KH	WPRO	0	1	0	0			
1934	2020-2-27 8:00	KH	WPRO	0	1	0	0			
1935	2020-2-28 8:00	KH	WPRO	0	1	0	0			
1936	2020-2-29 8:00	KH	WPRO	0	1	0	0			
1937	2020-3-1 8:00	KH	WPRO	0	1	0	0			
1938	2020-3-2 8:00	KH	WPRO	0	1	0	0			
1939	2020-3-3 8:00	KH	WPRO	0	1	0	0			
1940	2020-3-4 8:00	KH	WPRO	0	1	0	0			
1941	2020-3-5 8:00	KH	WPRO	0	1	0	0			
1942	2020-3-6 8:00	KH	WPRO	0	1	0	0			
1943	2020-3-7 8:00	KH	WPRO	1	2	0	0			
1944	2020-3-8 8:00	KH	WPRO	0	2	0	0			

Table S1 Daily confirmed cases and daily deaths of COVID-19 in 214 nations from WHO

ID	Date and time	Country or region	Continent	Confirmed patients	Cumulative Confirmed patients	Daily dead patients	Daily cumulative dead patients	Lag days	Daily CFR	3DMA CFR
1945	2020-3-9 8:00	KH	WPRO	0	2	0	0			
1946	2020-3-10 8:00	KH	WPRO	1	3	0	0			
1947	2020-3-11 8:00	KH	WPRO	0	3	0	0			
1948	2020-3-12 8:00	KH	WPRO	2	5	0	0			
1949	2020-3-13 8:00	KH	WPRO	0	5	0	0			
1950	2020-3-14 8:00	KH	WPRO	2	7	0	0			
1951	2020-3-15 8:00	KH	WPRO	5	12	0	0			
1952	2020-3-16 8:00	KH	WPRO	0	12	0	0			
1953	2020-3-17 8:00	KH	WPRO	12	24	0	0			
1954	2020-3-18 8:00	KH	WPRO	11	35	0	0			
1955	2020-3-19 8:00	KH	WPRO	12	47	0	0			
1956	2020-3-20 8:00	KH	WPRO	4	51	0	0			
1957	2020-3-21 8:00	KH	WPRO	2	53	0	0			
1958	2020-3-22 8:00	KH	WPRO	0	53	0	0			
1959	2020-3-23 8:00	KH	WPRO	33	86	0	0			
1960	2020-3-24 8:00	KH	WPRO	1	87	0	0			
1961	2020-3-25 8:00	KH	WPRO	4	91	0	0			
1962	2020-3-26 8:00	KH	WPRO	5	96	0	0			
1963	2020-3-27 8:00	KH	WPRO	2	98	0	0			
1964	2020-3-28 8:00	KH	WPRO	4	102	0	0			
1965	2020-3-29 8:00	KH	WPRO	0	102	0	0			
1966	2020-3-30 8:00	KH	WPRO	5	107	0	0			
1967	2020-3-31 8:00	KH	WPRO	0	107	0	0			
1968	2020-4-1 8:00	KH	WPRO	2	109	0	0			
1969	2020-4-2 8:00	KH	WPRO	0	109	0	0			
1970	2020-4-3 8:00	KH	WPRO	1	110	0	0			
1971	2020-4-4 8:00	KH	WPRO	4	114	0	0			
1972	2020-4-5 8:00	KH	WPRO	0	114	0	0			
1973	2020-4-6 8:00	KH	WPRO	0	114	0	0			
1974	2020-4-7 8:00	KH	WPRO	1	115	0	0			
1975	2020-4-8 8:00	KH	WPRO	2	117	0	0			
1976	2020-4-9 8:00	KH	WPRO	0	117	0	0			
1977	2020-4-10 8:00	KH	WPRO	1	118	0	0			
1978	2020-4-11 8:00	KH	WPRO	2	120	0	0			
1979	2020-4-12 8:00	KH	WPRO	2	122	0	0			
1980	2020-4-13 8:00	KH	WPRO	0	122	0	0			

Table S1 Daily confirmed cases and daily deaths of COVID-19 in 214 nations from WHO

ID	Date and time	Country or region	Continent	Confirmed patients	Cumulative Confirmed patients	Daily dead patients	Daily cumulative dead patients	Lag days	Daily CFR	3DMA CFR
1981	2020-4-14 8:00	KH	WPRO	0	122	0	0			
1982	2020-4-15 8:00	KH	WPRO	0	122	0	0			
1983	2020-4-16 8:00	KH	WPRO	0	122	0	0			
1984	2020-4-17 8:00	KH	WPRO	0	122	0	0			
1985	2020-4-18 8:00	KH	WPRO	0	122	0	0			
1986	2020-4-19 8:00	KH	WPRO	0	122	0	0			
1987	2020-4-20 8:00	KH	WPRO	0	122	0	0			
1988	2020-4-21 8:00	KH	WPRO	0	122	0	0			
1989	2020-4-22 8:00	KH	WPRO	0	122	0	0			
1990	2020-4-23 8:00	KH	WPRO	0	122	0	0			
1991	2020-4-24 8:00	KH	WPRO	0	122	0	0			
1992	2020-4-25 8:00	KH	WPRO	0	122	0	0			
1993	2020-4-26 8:00	KH	WPRO	0	122	0	0			
1994	2020-4-27 8:00	KH	WPRO	0	122	0	0			
1995	2020-4-28 8:00	KH	WPRO	0	122	0	0			
1996	2020-4-29 8:00	KH	WPRO	0	122	0	0			
1997	2020-4-30 8:00	KH	WPRO	0	122	0	0			
1998	2020-5-1 8:00	KH	WPRO	0	122	0	0			
1999	2020-5-2 8:00	KH	WPRO	0	122	0	0			
2000										
2001	2020-3-6 8:00	CM	AFRO	2	2	0	0			
2002	2020-3-7 8:00	CM	AFRO	0	2	0	0			
2003	2020-3-8 8:00	CM	AFRO	0	2	0	0			
2004	2020-3-9 8:00	CM	AFRO	0	2	0	0			
2005	2020-3-10 8:00	CM	AFRO	0	2	0	0			
2006	2020-3-11 8:00	CM	AFRO	0	2	0	0			
2007	2020-3-12 8:00	CM	AFRO	0	2	0	0			
2008	2020-3-13 8:00	CM	AFRO	0	2	0	0			
2009	2020-3-14 8:00	CM	AFRO	1	3	0	0			
2010	2020-3-15 8:00	CM	AFRO	0	3	0	0			
2011	2020-3-16 8:00	CM	AFRO	2	5	0	0			
2012	2020-3-17 8:00	CM	AFRO	0	5	0	0			
2013	2020-3-18 8:00	CM	AFRO	5	10	0	0			
2014	2020-3-19 8:00	CM	AFRO	5	15	0	0			
2015	2020-3-20 8:00	CM	AFRO	7	22	0	0			
2016	2020-3-21 8:00	CM	AFRO	5	27	0	0			

Table S1 Daily confirmed cases and daily deaths of COVID-19 in 214 nations from WHO

ID	Date and time	Country or region	Continent	Confirmed patients	Cumulative Confirmed patients	Daily dead patients	Daily cumulative dead patients	Lag days	Daily CFR	3DMA CFR
2017	2020-3-22 8:00	CM	AFRO	0	27	0	0			
2018	2020-3-23 8:00	CM	AFRO	13	40	0	0			
2019	2020-3-24 8:00	CM	AFRO	30	70	0	0			
2020	2020-3-25 8:00	CM	AFRO	0	70	1	1	0	1.429	1.381
2021	2020-3-26 8:00	CM	AFRO	5	75	0	1	1	1.333	1.365
2022	2020-3-27 8:00	CM	AFRO	0	75	0	1	2	1.333	1.333
2023	2020-3-28 8:00	CM	AFRO	0	75	0	1	3	1.333	1.621
2024	2020-3-29 8:00	CM	AFRO	16	91	1	2	4	2.198	1.767
2025	2020-3-30 8:00	CM	AFRO	22	113	0	2	5	1.770	2.761
2026	2020-3-31 8:00	CM	AFRO	26	139	4	6	6	4.317	3.468
2027	2020-4-1 8:00	CM	AFRO	0	139	0	6	7	4.317	4.317
2028	2020-4-2 8:00	CM	AFRO	0	139	0	6	8	4.317	3.826
2029	2020-4-3 8:00	CM	AFRO	107	246	1	7	9	2.846	2.911
2030	2020-4-4 8:00	CM	AFRO	263	509	1	8	10	1.572	2.013
2031	2020-4-5 8:00	CM	AFRO	46	555	1	9	11	1.622	1.605
2032	2020-4-6 8:00	CM	AFRO	0	555	0	9	12	1.622	1.622
2033	2020-4-7 8:00	CM	AFRO	0	555	0	9	13	1.622	1.622
2034	2020-4-8 8:00	CM	AFRO	0	555	0	9	14	1.622	1.538
2035	2020-4-9 8:00	CM	AFRO	175	730	1	10	15	1.370	1.454
2036	2020-4-10 8:00	CM	AFRO	0	730	0	10	16	1.370	1.328
2037	2020-4-11 8:00	CM	AFRO	73	803	0	10	17	1.245	1.287
2038	2020-4-12 8:00	CM	AFRO	0	803	0	10	18	1.245	1.245
2039	2020-4-13 8:00	CM	AFRO	0	803	0	10	19	1.245	1.245
2040	2020-4-14 8:00	CM	AFRO	0	803	0	10	20	1.245	1.415
2041	2020-4-15 8:00	CM	AFRO	52	855	5	15	21	1.754	1.663
2042	2020-4-16 8:00	CM	AFRO	0	855	2	17	22	1.988	1.910
2043	2020-4-17 8:00	CM	AFRO	0	855	0	17	23	1.988	2.015
2044	2020-4-18 8:00	CM	AFRO	161	1016	4	21	24	2.067	2.041
2045	2020-4-19 8:00	CM	AFRO	0	1016	0	21	25	2.067	2.067
2046	2020-4-20 8:00	CM	AFRO	0	1016	0	21	26	2.067	2.582
2047	2020-4-21 8:00	CM	AFRO	147	1163	21	42	27	3.611	3.125
2048	2020-4-22 8:00	CM	AFRO	0	1163	1	43	28	3.697	3.669
2049	2020-4-23 8:00	CM	AFRO	0	1163	0	43	29	3.697	3.631
2050	2020-4-24 8:00	CM	AFRO	238	1401	6	49	30	3.498	3.562
2051	2020-4-25 8:00	CM	AFRO	2	1403	0	49	31	3.493	3.494
2052	2020-4-26 8:00	CM	AFRO	115	1518	4	53	32	3.491	3.480

Table S1 Daily confirmed cases and daily deaths of COVID-19 in 214 nations from WHO

ID	Date and time	Country or region	Continent	Confirmed patients	Cumulative Confirmed patients	Daily dead patients	Daily cumulative dead patients	Lag days	Daily CFR	3DMA CFR
2053	2020-4-27 8:00	CM	AFRO	103	1621	3	56	33	3.455	3.467
2054	2020-4-28 8:00	CM	AFRO	0	1621	0	56	34	3.455	3.437
2055	2020-4-29 8:00	CM	AFRO	84	1705	2	58	35	3.402	3.374
2056	2020-4-30 8:00	CM	AFRO	101	1806	1	59	36	3.267	3.333
2057	2020-5-1 8:00	CM	AFRO	26	1832	2	61	37	3.330	3.182
2058	2020-5-2 8:00	CM	AFRO	237	2069	0	61	38	2.948	3.139
2059										
2060	2020-1-26 8:00	CA	AMRO	1	1	0	0			
2061	2020-1-27 8:00	CA	AMRO	1	2	0	0			
2062	2020-1-28 8:00	CA	AMRO	1	3	0	0			
2063	2020-1-29 8:00	CA	AMRO	0	3	0	0			
2064	2020-1-30 8:00	CA	AMRO	0	3	0	0			
2065	2020-1-31 8:00	CA	AMRO	1	4	0	0			
2066	2020-2-1 8:00	CA	AMRO	0	4	0	0			
2067	2020-2-2 8:00	CA	AMRO	0	4	0	0			
2068	2020-2-3 8:00	CA	AMRO	0	4	0	0			
2069	2020-2-4 8:00	CA	AMRO	0	4	0	0			
2070	2020-2-5 8:00	CA	AMRO	1	5	0	0			
2071	2020-2-6 8:00	CA	AMRO	0	5	0	0			
2072	2020-2-7 8:00	CA	AMRO	2	7	0	0			
2073	2020-2-8 8:00	CA	AMRO	0	7	0	0			
2074	2020-2-9 8:00	CA	AMRO	0	7	0	0			
2075	2020-2-10 8:00	CA	AMRO	0	7	0	0			
2076	2020-2-11 8:00	CA	AMRO	0	7	0	0			
2077	2020-2-12 8:00	CA	AMRO	0	7	0	0			
2078	2020-2-13 8:00	CA	AMRO	0	7	0	0			
2079	2020-2-14 8:00	CA	AMRO	0	7	0	0			
2080	2020-2-15 8:00	CA	AMRO	0	7	0	0			
2081	2020-2-16 8:00	CA	AMRO	0	7	0	0			
2082	2020-2-17 8:00	CA	AMRO	1	8	0	0			
2083	2020-2-18 8:00	CA	AMRO	0	8	0	0			
2084	2020-2-19 8:00	CA	AMRO	0	8	0	0			
2085	2020-2-20 8:00	CA	AMRO	0	8	0	0			
2086	2020-2-21 8:00	CA	AMRO	0	8	0	0			
2087	2020-2-22 8:00	CA	AMRO	1	9	0	0			
2088	2020-2-23 8:00	CA	AMRO	0	9	0	0			

Table S1 Daily confirmed cases and daily deaths of COVID-19 in 214 nations from WHO

ID	Date and time	Country or region	Continent	Confirmed patients	Cumulative Confirmed patients	Daily dead patients	Daily cumulative dead patients	Lag days	Daily CFR	3DMA CFR
2089	2020-2-24 8:00	CA	AMRO	1	10	0	0			
2090	2020-2-25 8:00	CA	AMRO	0	10	0	0			
2091	2020-2-26 8:00	CA	AMRO	1	11	0	0			
2092	2020-2-27 8:00	CA	AMRO	0	11	0	0			
2093	2020-2-28 8:00	CA	AMRO	3	14	0	0			
2094	2020-2-29 8:00	CA	AMRO	0	14	0	0			
2095	2020-3-1 8:00	CA	AMRO	5	19	0	0			
2096	2020-3-2 8:00	CA	AMRO	8	27	0	0			
2097	2020-3-3 8:00	CA	AMRO	3	30	0	0			
2098	2020-3-4 8:00	CA	AMRO	0	30	0	0			
2099	2020-3-5 8:00	CA	AMRO	15	45	0	0			
2100	2020-3-6 8:00	CA	AMRO	4	49	0	0			
2101	2020-3-7 8:00	CA	AMRO	8	57	0	0			
2102	2020-3-8 8:00	CA	AMRO	3	60	0	0			
2103	2020-3-9 8:00	CA	AMRO	12	72	0	0			
2104	2020-3-10 8:00	CA	AMRO	5	77	0	0			
2105	2020-3-11 8:00	CA	AMRO	16	93	1	1	0	1.075	1.075
2106	2020-3-12 8:00	CA	AMRO	0	93	0	1	1	1.075	0.936
2107	2020-3-13 8:00	CA	AMRO	59	152	0	1	2	0.658	0.767
2108	2020-3-14 8:00	CA	AMRO	24	176	0	1	3	0.568	0.518
2109	2020-3-15 8:00	CA	AMRO	128	304	0	1	4	0.329	0.409
2110	2020-3-16 8:00	CA	AMRO	0	304	0	1	5	0.329	0.298
2111	2020-3-17 8:00	CA	AMRO	120	424	0	1	6	0.236	0.716
2112	2020-3-18 8:00	CA	AMRO	145	569	8	9	7	1.582	1.013
2113	2020-3-19 8:00	CA	AMRO	167	736	0	9	8	1.223	1.289
2114	2020-3-20 8:00	CA	AMRO	110	846	0	9	9	1.064	1.176
2115	2020-3-21 8:00	CA	AMRO	202	1048	4	13	10	1.240	1.182
2116	2020-3-22 8:00	CA	AMRO	0	1048	0	13	11	1.240	1.285
2117	2020-3-23 8:00	CA	AMRO	336	1384	6	19	12	1.373	1.350
2118	2020-3-24 8:00	CA	AMRO	355	1739	6	25	13	1.438	1.416
2119	2020-3-25 8:00	CA	AMRO	0	1739	0	25	14	1.438	1.287
2120	2020-3-26 8:00	CA	AMRO	1816	3555	10	35	15	0.985	1.136
2121	2020-3-27 8:00	CA	AMRO	0	3555	0	35	16	0.985	0.980
2122	2020-3-28 8:00	CA	AMRO	463	4018	4	39	17	0.971	1.037
2123	2020-3-29 8:00	CA	AMRO	739	4757	16	55	18	1.156	1.069
2124	2020-3-30 8:00	CA	AMRO	898	5655	6	61	19	1.079	1.093

Table S1 Daily confirmed cases and daily deaths of COVID-19 in 214 nations from WHO

ID	Date and time	Country or region	Continent	Confirmed patients	Cumulative Confirmed patients	Daily dead patients	Daily cumulative dead patients	Lag days	Daily CFR	3DMA CFR
2125	2020-3-31 8:00	CA	AMRO	662	6317	5	66	20	1.045	1.093
2126	2020-4-1 8:00	CA	AMRO	1378	7695	23	89	21	1.157	1.122
2127	2020-4-2 8:00	CA	AMRO	1310	9005	16	105	22	1.166	1.193
2128	2020-4-3 8:00	CA	AMRO	1109	10114	22	127	23	1.256	1.239
2129	2020-4-4 8:00	CA	AMRO	1618	11732	25	152	24	1.296	1.402
2130	2020-4-5 8:00	CA	AMRO	1206	12938	62	214	25	1.654	1.537
2131	2020-4-6 8:00	CA	AMRO	966	13904	17	231	26	1.661	1.723
2132	2020-4-7 8:00	CA	AMRO	1902	15806	62	293	27	1.854	1.846
2133	2020-4-8 8:00	CA	AMRO	1243	17049	52	345	28	2.024	2.018
2134	2020-4-9 8:00	CA	AMRO	1384	18433	56	401	29	2.175	2.177
2135	2020-4-10 8:00	CA	AMRO	1326	19759	60	461	30	2.333	2.337
2136	2020-4-11 8:00	CA	AMRO	1467	21226	70	531	31	2.502	2.499
2137	2020-4-12 8:00	CA	AMRO	1318	22544	69	600	32	2.661	2.669
2138	2020-4-13 8:00	CA	AMRO	1158	23702	74	674	33	2.844	2.822
2139	2020-4-14 8:00	CA	AMRO	1084	24786	60	734	34	2.961	2.984
2140	2020-4-15 8:00	CA	AMRO	1360	26146	89	823	35	3.148	3.191
2141	2020-4-16 8:00	CA	AMRO	1394	27540	131	954	36	3.464	3.413
2142	2020-4-17 8:00	CA	AMRO	1344	28884	94	1048	37	3.628	3.723
2143	2020-4-18 8:00	CA	AMRO	1775	30659	202	1250	38	4.077	3.953
2144	2020-4-19 8:00	CA	AMRO	1741	32400	96	1346	39	4.154	4.224
2145	2020-4-20 8:00	CA	AMRO	1509	33909	160	1506	40	4.441	4.383
2146	2020-4-21 8:00	CA	AMRO	1474	35383	105	1611	41	4.553	4.539
2147	2020-4-22 8:00	CA	AMRO	1991	37374	117	1728	42	4.624	4.661
2148	2020-4-23 8:00	CA	AMRO	1549	38923	143	1871	43	4.807	4.800
2149	2020-4-24 8:00	CA	AMRO	1890	40813	157	2028	44	4.969	4.972
2150	2020-4-25 8:00	CA	AMRO	1926	42739	169	2197	45	5.141	5.136
2151	2020-4-26 8:00	CA	AMRO	1614	44353	153	2350	46	5.298	5.292
2152	2020-4-27 8:00	CA	AMRO	1425	45778	139	2489	47	5.437	5.422
2153	2020-4-28 8:00	CA	AMRO	1538	47316	128	2617	48	5.531	5.537
2154	2020-4-29 8:00	CA	AMRO	1698	49014	149	2766	49	5.643	5.647
2155	2020-4-30 8:00	CA	AMRO	1349	50363	138	2904	50	5.766	5.777
2156	2020-5-1 8:00	CA	AMRO	1693	52056	178	3082	51	5.921	5.898
2157	2020-5-2 8:00	CA	AMRO	1601	53657	141	3223	52	6.007	5.964
2158										
2159	2020-3-13 8:00	KY	AMRO	1	1	0	0			
2160	2020-3-14 8:00	KY	AMRO	0	1	0	0			

Table S1 Daily confirmed cases and daily deaths of COVID-19 in 214 nations from WHO

ID	Date and time	Country or region	Continent	Confirmed patients	Cumulative Confirmed patients	Daily dead patients	Daily cumulative dead patients	Lag days	Daily CFR	3DMA CFR
2161	2020-3-15 8:00	KY	AMRO	0	1	0	0			
2162	2020-3-16 8:00	KY	AMRO	0	1	1	1	0	100.000	100.000
2163	2020-3-17 8:00	KY	AMRO	0	1	0	1	1	100.000	100.000
2164	2020-3-18 8:00	KY	AMRO	0	1	0	1	2	100.000	77.778
2165	2020-3-19 8:00	KY	AMRO	2	3	0	1	3	33.333	55.556
2166	2020-3-20 8:00	KY	AMRO	0	3	0	1	4	33.333	33.333
2167	2020-3-21 8:00	KY	AMRO	0	3	0	1	5	33.333	33.333
2168	2020-3-22 8:00	KY	AMRO	0	3	0	1	6	33.333	33.333
2169	2020-3-23 8:00	KY	AMRO	0	3	0	1	7	33.333	28.889
2170	2020-3-24 8:00	KY	AMRO	2	5	0	1	8	20.000	24.444
2171	2020-3-25 8:00	KY	AMRO	0	5	0	1	9	20.000	17.500
2172	2020-3-26 8:00	KY	AMRO	3	8	0	1	10	12.500	15.000
2173	2020-3-27 8:00	KY	AMRO	0	8	0	1	11	12.500	12.500
2174	2020-3-28 8:00	KY	AMRO	0	8	0	1	12	12.500	12.500
2175	2020-3-29 8:00	KY	AMRO	0	8	0	1	13	12.500	12.500
2176	2020-3-30 8:00	KY	AMRO	0	8	0	1	14	12.500	11.111
2177	2020-3-31 8:00	KY	AMRO	4	12	0	1	15	8.333	9.722
2178	2020-4-1 8:00	KY	AMRO	0	12	0	1	16	8.333	7.937
2179	2020-4-2 8:00	KY	AMRO	2	14	0	1	17	7.143	6.674
2180	2020-4-3 8:00	KY	AMRO	8	22	0	1	18	4.545	5.087
2181	2020-4-4 8:00	KY	AMRO	6	28	0	1	19	3.571	3.896
2182	2020-4-5 8:00	KY	AMRO	0	28	0	1	20	3.571	3.333
2183	2020-4-6 8:00	KY	AMRO	7	35	0	1	21	2.857	2.998
2184	2020-4-7 8:00	KY	AMRO	4	39	0	1	22	2.564	2.662
2185	2020-4-8 8:00	KY	AMRO	0	39	0	1	23	2.564	2.450
2186	2020-4-9 8:00	KY	AMRO	6	45	0	1	24	2.222	2.336
2187	2020-4-10 8:00	KY	AMRO	0	45	0	1	25	2.222	2.222
2188	2020-4-11 8:00	KY	AMRO	0	45	0	1	26	2.222	2.222
2189	2020-4-12 8:00	KY	AMRO	0	45	0	1	27	2.222	2.110
2190	2020-4-13 8:00	KY	AMRO	8	53	0	1	28	1.887	1.999
2191	2020-4-14 8:00	KY	AMRO	0	53	0	1	29	1.887	1.875
2192	2020-4-15 8:00	KY	AMRO	1	54	0	1	30	1.852	1.863
2193	2020-4-16 8:00	KY	AMRO	0	54	0	1	31	1.852	1.790
2194	2020-4-17 8:00	KY	AMRO	6	60	0	1	32	1.667	1.719
2195	2020-4-18 8:00	KY	AMRO	1	61	0	1	33	1.639	1.648
2196	2020-4-19 8:00	KY	AMRO	0	61	0	1	34	1.639	1.639

Table S1 Daily confirmed cases and daily deaths of COVID-19 in 214 nations from WHO

ID	Date and time	Country or region	Continent	Confirmed patients	Cumulative Confirmed patients	Daily dead patients	Daily cumulative dead patients	Lag days	Daily CFR	3DMA CFR
2197	2020-4-20 8:00	KY	AMRO	0	61	0	1	35	1.639	1.639
2198	2020-4-21 8:00	KY	AMRO	0	61	0	1	36	1.639	1.598
2199	2020-4-22 8:00	KY	AMRO	5	66	0	1	37	1.515	1.557
2200	2020-4-23 8:00	KY	AMRO	0	66	0	1	38	1.515	1.515
2201	2020-4-24 8:00	KY	AMRO	0	66	0	1	39	1.515	1.515
2202	2020-4-25 8:00	KY	AMRO	0	66	0	1	40	1.515	1.486
2203	2020-4-26 8:00	KY	AMRO	4	70	0	1	41	1.429	1.457
2204	2020-4-27 8:00	KY	AMRO	0	70	0	1	42	1.429	1.429
2205	2020-4-28 8:00	KY	AMRO	0	70	0	1	43	1.429	1.429
2206	2020-4-29 8:00	KY	AMRO	0	70	0	1	44	1.429	1.409
2207	2020-4-30 8:00	KY	AMRO	3	73	0	1	45	1.370	1.389
2208	2020-5-1 8:00	KY	AMRO	0	73	0	1	46	1.370	1.370
2209	2020-5-2 8:00	KY	AMRO	0	73	0	1	47	1.370	1.370
2210										
2211	2020-3-14 8:00	CF	AFRO	1	1	0	0			
2212	2020-3-15 8:00	CF	AFRO	0	1	0	0			
2213	2020-3-16 8:00	CF	AFRO	0	1	0	0			
2214	2020-3-17 8:00	CF	AFRO	0	1	0	0			
2215	2020-3-18 8:00	CF	AFRO	0	1	0	0			
2216	2020-3-19 8:00	CF	AFRO	0	1	0	0			
2217	2020-3-20 8:00	CF	AFRO	0	1	0	0			
2218	2020-3-21 8:00	CF	AFRO	2	3	0	0			
2219	2020-3-22 8:00	CF	AFRO	0	3	0	0			
2220	2020-3-23 8:00	CF	AFRO	1	4	0	0			
2221	2020-3-24 8:00	CF	AFRO	0	4	0	0			
2222	2020-3-25 8:00	CF	AFRO	0	4	0	0			
2223	2020-3-26 8:00	CF	AFRO	1	5	0	0			
2224	2020-3-27 8:00	CF	AFRO	0	5	0	0			
2225	2020-3-28 8:00	CF	AFRO	1	6	0	0			
2226	2020-3-29 8:00	CF	AFRO	0	6	0	0			
2227	2020-3-30 8:00	CF	AFRO	0	6	0	0			
2228	2020-3-31 8:00	CF	AFRO	0	6	0	0			
2229	2020-4-1 8:00	CF	AFRO	0	6	0	0			
2230	2020-4-2 8:00	CF	AFRO	2	8	0	0			
2231	2020-4-3 8:00	CF	AFRO	0	8	0	0			
2232	2020-4-4 8:00	CF	AFRO	0	8	0	0			

Table S1 Daily confirmed cases and daily deaths of COVID-19 in 214 nations from WHO

ID	Date and time	Country or region	Continent	Confirmed patients	Cumulative Confirmed patients	Daily dead patients	Daily cumulative dead patients	Lag days	Daily CFR	3DMA CFR
2233	2020-4-5 8:00	CF	AFRO	1	9	0	0			
2234	2020-4-6 8:00	CF	AFRO	0	9	0	0			
2235	2020-4-7 8:00	CF	AFRO	0	9	0	0			
2236	2020-4-8 8:00	CF	AFRO	0	9	0	0			
2237	2020-4-9 8:00	CF	AFRO	1	10	0	0			
2238	2020-4-10 8:00	CF	AFRO	0	10	0	0			
2239	2020-4-11 8:00	CF	AFRO	1	11	0	0			
2240	2020-4-12 8:00	CF	AFRO	0	11	0	0			
2241	2020-4-13 8:00	CF	AFRO	0	11	0	0			
2242	2020-4-14 8:00	CF	AFRO	0	11	0	0			
2243	2020-4-15 8:00	CF	AFRO	0	11	0	0			
2244	2020-4-16 8:00	CF	AFRO	0	11	0	0			
2245	2020-4-17 8:00	CF	AFRO	1	12	0	0			
2246	2020-4-18 8:00	CF	AFRO	0	12	0	0			
2247	2020-4-19 8:00	CF	AFRO	0	12	0	0			
2248	2020-4-20 8:00	CF	AFRO	0	12	0	0			
2249	2020-4-21 8:00	CF	AFRO	0	12	0	0			
2250	2020-4-22 8:00	CF	AFRO	2	14	0	0			
2251	2020-4-23 8:00	CF	AFRO	0	14	0	0			
2252	2020-4-24 8:00	CF	AFRO	2	16	0	0			
2253	2020-4-25 8:00	CF	AFRO	3	19	0	0			
2254	2020-4-26 8:00	CF	AFRO	0	19	0	0			
2255	2020-4-27 8:00	CF	AFRO	0	19	0	0			
2256	2020-4-28 8:00	CF	AFRO	23	42	0	0			
2257	2020-4-29 8:00	CF	AFRO	8	50	0	0			
2258	2020-4-30 8:00	CF	AFRO	0	50	0	0			
2259	2020-5-1 8:00	CF	AFRO	14	64	0	0			
2260	2020-5-2 8:00	CF	AFRO	0	64	0	0			
2261										
2262	2020-3-19 8:00	TD	AFRO	1	1	0	0			
2263	2020-3-20 8:00	TD	AFRO	0	1	0	0			
2264	2020-3-21 8:00	TD	AFRO	0	1	0	0			
2265	2020-3-22 8:00	TD	AFRO	0	1	0	0			
2266	2020-3-23 8:00	TD	AFRO	0	1	0	0			
2267	2020-3-24 8:00	TD	AFRO	2	3	0	0			
2268	2020-3-25 8:00	TD	AFRO	0	3	0	0			

Table S1 Daily confirmed cases and daily deaths of COVID-19 in 214 nations from WHO

ID	Date and time	Country or region	Continent	Confirmed patients	Cumulative Confirmed patients	Daily dead patients	Daily cumulative dead patients	Lag days	Daily CFR	3DMA CFR
2269	2020-3-26 8:00	TD	AFRO	0	3	0	0			
2270	2020-3-27 8:00	TD	AFRO	2	5	0	0			
2271	2020-3-28 8:00	TD	AFRO	0	5	0	0			
2272	2020-3-29 8:00	TD	AFRO	0	5	0	0			
2273	2020-3-30 8:00	TD	AFRO	0	5	0	0			
2274	2020-3-31 8:00	TD	AFRO	0	5	0	0			
2275	2020-4-1 8:00	TD	AFRO	2	7	0	0			
2276	2020-4-2 8:00	TD	AFRO	0	7	0	0			
2277	2020-4-3 8:00	TD	AFRO	0	7	0	0			
2278	2020-4-4 8:00	TD	AFRO	0	7	0	0			
2279	2020-4-5 8:00	TD	AFRO	0	7	0	0			
2280	2020-4-6 8:00	TD	AFRO	2	9	0	0			
2281	2020-4-7 8:00	TD	AFRO	0	9	0	0			
2282	2020-4-8 8:00	TD	AFRO	1	10	0	0			
2283	2020-4-9 8:00	TD	AFRO	0	10	0	0			
2284	2020-4-10 8:00	TD	AFRO	1	11	0	0			
2285	2020-4-11 8:00	TD	AFRO	0	11	0	0			
2286	2020-4-12 8:00	TD	AFRO	0	11	0	0			
2287	2020-4-13 8:00	TD	AFRO	7	18	0	0			
2288	2020-4-14 8:00	TD	AFRO	5	23	0	0			
2289	2020-4-15 8:00	TD	AFRO	0	23	0	0			
2290	2020-4-16 8:00	TD	AFRO	4	27	0	0			
2291	2020-4-17 8:00	TD	AFRO	0	27	0	0			
2292	2020-4-18 8:00	TD	AFRO	6	33	0	0			
2293	2020-4-19 8:00	TD	AFRO	0	33	0	0			
2294	2020-4-20 8:00	TD	AFRO	0	33	0	0			
2295	2020-4-21 8:00	TD	AFRO	0	33	0	0			
2296	2020-4-22 8:00	TD	AFRO	1	34	0	0			
2297	2020-4-23 8:00	TD	AFRO	0	34	0	0			
2298	2020-4-24 8:00	TD	AFRO	0	34	0	0			
2299	2020-4-25 8:00	TD	AFRO	6	40	0	0			
2300	2020-4-26 8:00	TD	AFRO	6	46	0	0			
2301	2020-4-27 8:00	TD	AFRO	0	46	0	0			
2302	2020-4-28 8:00	TD	AFRO	0	46	0	0			
2303	2020-4-29 8:00	TD	AFRO	6	52	2	2	0	3.846	3.846
2304	2020-4-30 8:00	TD	AFRO	0	52	0	2	1	3.846	3.934

Table S1 Daily confirmed cases and daily deaths of COVID-19 in 214 nations from WHO

ID	Date and time	Country or region	Continent	Confirmed patients	Cumulative Confirmed patients	Daily dead patients	Daily cumulative dead patients	Lag days	Daily CFR	3DMA CFR
2305	2020-5-1 8:00	TD	AFRO	21	73	1	3	2	4.110	4.935
2306	2020-5-2 8:00	TD	AFRO	0	73	2	5	3	6.849	5.479
2307										
2308	2020-3-3 8:00	CL	AMRO	1	1	0	0			
2309	2020-3-4 8:00	CL	AMRO	0	1	0	0			
2310	2020-3-5 8:00	CL	AMRO	0	1	0	0			
2311	2020-3-6 8:00	CL	AMRO	4	5	0	0			
2312	2020-3-7 8:00	CL	AMRO	0	5	0	0			
2313	2020-3-8 8:00	CL	AMRO	5	10	0	0			
2314	2020-3-9 8:00	CL	AMRO	0	10	0	0			
2315	2020-3-10 8:00	CL	AMRO	3	13	0	0			
2316	2020-3-11 8:00	CL	AMRO	4	17	0	0			
2317	2020-3-12 8:00	CL	AMRO	6	23	0	0			
2318	2020-3-13 8:00	CL	AMRO	20	43	0	0			
2319	2020-3-14 8:00	CL	AMRO	18	61	0	0			
2320	2020-3-15 8:00	CL	AMRO	0	61	0	0			
2321	2020-3-16 8:00	CL	AMRO	14	75	0	0			
2322	2020-3-17 8:00	CL	AMRO	81	156	0	0			
2323	2020-3-18 8:00	CL	AMRO	82	238	0	0			
2324	2020-3-19 8:00	CL	AMRO	104	342	0	0			
2325	2020-3-20 8:00	CL	AMRO	92	434	0	0			
2326	2020-3-21 8:00	CL	AMRO	0	434	1	1	0	0.230	0.230
2327	2020-3-22 8:00	CL	AMRO	0	434	0	1	1	0.230	0.206
2328	2020-3-23 8:00	CL	AMRO	198	632	0	1	2	0.158	0.202
2329	2020-3-24 8:00	CL	AMRO	290	922	1	2	3	0.217	0.197
2330	2020-3-25 8:00	CL	AMRO	0	922	0	2	4	0.217	0.247
2331	2020-3-26 8:00	CL	AMRO	384	1306	2	4	5	0.306	0.276
2332	2020-3-27 8:00	CL	AMRO	0	1306	0	4	6	0.306	0.308
2333	2020-3-28 8:00	CL	AMRO	304	1610	1	5	7	0.311	0.310
2334	2020-3-29 8:00	CL	AMRO	299	1909	1	6	8	0.314	0.313
2335	2020-3-30 8:00	CL	AMRO	0	1909	0	6	9	0.314	0.318
2336	2020-3-31 8:00	CL	AMRO	540	2449	2	8	10	0.327	0.360
2337	2020-4-1 8:00	CL	AMRO	289	2738	4	12	11	0.438	0.431
2338	2020-4-2 8:00	CL	AMRO	293	3031	4	16	12	0.528	0.498
2339	2020-4-3 8:00	CL	AMRO	373	3404	2	18	13	0.529	0.548
2340	2020-4-4 8:00	CL	AMRO	333	3737	4	22	14	0.589	0.589

Table S1 Daily confirmed cases and daily deaths of COVID-19 in 214 nations from WHO

ID	Date and time	Country or region	Continent	Confirmed patients	Cumulative Confirmed patients	Daily dead patients	Daily cumulative dead patients	Lag days	Daily CFR	3DMA CFR
2341	2020-4-5 8:00	CL	AMRO	424	4161	5	27	15	0.649	0.666
2342	2020-4-6 8:00	CL	AMRO	310	4471	7	34	16	0.760	0.726
2343	2020-4-7 8:00	CL	AMRO	344	4815	3	37	17	0.768	0.790
2344	2020-4-8 8:00	CL	AMRO	301	5116	6	43	18	0.841	0.825
2345	2020-4-9 8:00	CL	AMRO	430	5546	5	48	19	0.865	0.887
2346	2020-4-10 8:00	CL	AMRO	426	5972	9	57	20	0.954	0.940
2347	2020-4-11 8:00	CL	AMRO	529	6501	8	65	21	1.000	1.003
2348	2020-4-12 8:00	CL	AMRO	426	6927	8	73	22	1.054	1.054
2349	2020-4-13 8:00	CL	AMRO	286	7213	7	80	23	1.109	1.084
2350	2020-4-14 8:00	CL	AMRO	312	7525	2	82	24	1.090	1.120
2351	2020-4-15 8:00	CL	AMRO	392	7917	10	92	25	1.162	1.129
2352	2020-4-16 8:00	CL	AMRO	356	8273	2	94	26	1.136	1.164
2353	2020-4-17 8:00	CL	AMRO	534	8807	11	105	27	1.192	1.194
2354	2020-4-18 8:00	CL	AMRO	445	9252	11	116	28	1.254	1.247
2355	2020-4-19 8:00	CL	AMRO	478	9730	10	126	29	1.295	1.272
2356	2020-4-20 8:00	CL	AMRO	777	10507	7	133	30	1.266	1.295
2357	2020-4-21 8:00	CL	AMRO	0	10507	6	139	31	1.323	1.315
2358	2020-4-22 8:00	CL	AMRO	325	10832	8	147	32	1.357	1.365
2359	2020-4-23 8:00	CL	AMRO	464	11296	13	160	33	1.416	1.399
2360	2020-4-24 8:00	CL	AMRO	516	11812	8	168	34	1.422	1.418
2361	2020-4-25 8:00	CL	AMRO	494	12306	6	174	35	1.414	1.415
2362	2020-4-26 8:00	CL	AMRO	552	12858	7	181	36	1.408	1.413
2363	2020-4-27 8:00	CL	AMRO	473	13331	8	189	37	1.418	1.420
2364	2020-4-28 8:00	CL	AMRO	482	13813	9	198	38	1.433	1.431
2365	2020-4-29 8:00	CL	AMRO	552	14365	9	207	39	1.441	1.442
2366	2020-4-30 8:00	CL	AMRO	520	14885	9	216	40	1.451	1.436
2367	2020-5-1 8:00	CL	AMRO	1138	16023	11	227	41	1.417	1.415
2368	2020-5-2 8:00	CL	AMRO	985	17008	7	234	42	1.376	1.744
2369										
2370	2020-1-11 8:00	CN	WPRO	41	41	1	1	0	2.439	2.085
2371	2020-1-12 8:00	CN	WPRO	0	41	0	1	1	2.439	2.439
2372	2020-1-13 8:00	CN	WPRO	0	41	0	1	2	2.439	2.439
2373	2020-1-14 8:00	CN	WPRO	0	41	0	1	3	2.439	2.439
2374	2020-1-15 8:00	CN	WPRO	0	41	0	1	4	2.439	2.439
2375	2020-1-16 8:00	CN	WPRO	0	41	0	1	5	2.439	3.107
2376	2020-1-17 8:00	CN	WPRO	4	45	1	2	6	4.444	3.370

Table S1 Daily confirmed cases and daily deaths of COVID-19 in 214 nations from WHO

ID	Date and time	Country or region	Continent	Confirmed patients	Cumulative Confirmed patients	Daily dead patients	Daily cumulative dead patients	Lag days	Daily CFR	3DMA CFR
2377	2020-1-18 8:00	CN	WPRO	17	62	0	2	7	3.226	3.383
2378	2020-1-19 8:00	CN	WPRO	59	121	1	3	8	2.479	2.575
2379	2020-1-20 8:00	CN	WPRO	77	198	1	4	9	2.020	2.187
2380	2020-1-21 8:00	CN	WPRO	93	291	2	6	10	2.062	2.038
2381	2020-1-22 8:00	CN	WPRO	152	443	3	9	11	2.032	2.352
2382	2020-1-23 8:00	CN	WPRO	131	574	8	17	12	2.962	2.662
2383	2020-1-24 8:00	CN	WPRO	261	835	8	25	13	2.994	3.039
2384	2020-1-25 8:00	CN	WPRO	462	1297	16	41	14	3.161	2.992
2385	2020-1-26 8:00	CN	WPRO	688	1985	15	56	15	2.821	2.960
2386	2020-1-27 8:00	CN	WPRO	776	2761	24	80	16	2.898	2.686
2387	2020-1-28 8:00	CN	WPRO	1772	4533	26	106	17	2.338	2.479
2388	2020-1-29 8:00	CN	WPRO	1462	5995	26	132	18	2.202	2.246
2389	2020-1-30 8:00	CN	WPRO	1741	7736	38	170	19	2.198	2.197
2390	2020-1-31 8:00	CN	WPRO	1984	9720	43	213	20	2.191	2.193
2391	2020-2-1 8:00	CN	WPRO	2101	11821	46	259	21	2.191	2.164
2392	2020-2-2 8:00	CN	WPRO	2590	14411	45	304	22	2.109	2.132
2393	2020-2-3 8:00	CN	WPRO	2827	17238	57	361	23	2.094	2.093
2394	2020-2-4 8:00	CN	WPRO	3233	20471	64	425	24	2.076	2.062
2395	2020-2-5 8:00	CN	WPRO	3892	24363	66	491	25	2.015	2.034
2396	2020-2-6 8:00	CN	WPRO	3697	28060	73	564	26	2.010	2.022
2397	2020-2-7 8:00	CN	WPRO	3151	31211	73	637	27	2.041	2.047
2398	2020-2-8 8:00	CN	WPRO	3387	34598	86	723	28	2.090	2.103
2399	2020-2-9 8:00	CN	WPRO	2653	37251	89	812	29	2.180	2.176
2400	2020-2-10 8:00	CN	WPRO	2985	40236	97	909	30	2.259	2.273
2401	2020-2-11 8:00	CN	WPRO	2472	42708	108	1017	31	2.381	2.377
2402	2020-2-12 8:00	CN	WPRO	2022	44730	97	1114	32	2.490	2.385
2403	2020-2-13 8:00	CN	WPRO	15152	59882	254	1368	33	2.284	2.312
2404	2020-2-14 8:00	CN	WPRO	4050	63932	13	1381	34	2.160	2.245
2405	2020-2-15 8:00	CN	WPRO	2644	66576	143	1524	35	2.289	2.293
2406	2020-2-16 8:00	CN	WPRO	2008	68584	142	1666	36	2.429	2.409
2407	2020-2-17 8:00	CN	WPRO	2051	70635	106	1772	37	2.509	2.505
2408	2020-2-18 8:00	CN	WPRO	1893	72528	98	1870	38	2.578	2.596
2409	2020-2-19 8:00	CN	WPRO	1751	74279	136	2006	39	2.701	2.706
2410	2020-2-20 8:00	CN	WPRO	396	74675	115	2121	40	2.840	2.835
2411	2020-2-21 8:00	CN	WPRO	892	75567	118	2239	41	2.963	2.959
2412	2020-2-22 8:00	CN	WPRO	825	76392	109	2348	42	3.074	3.070

Table S1 Daily confirmed cases and daily deaths of COVID-19 in 214 nations from WHO

ID	Date and time	Country or region	Continent	Confirmed patients	Cumulative Confirmed patients	Daily dead patients	Daily cumulative dead patients	Lag days	Daily CFR	3DMA CFR
2413	2020-2-23 8:00	CN	WPRO	649	77041	97	2445	43	3.174	3.202
2414	2020-2-24 8:00	CN	WPRO	221	77262	150	2595	44	3.359	3.320
2415	2020-2-25 8:00	CN	WPRO	517	77779	71	2666	45	3.428	3.421
2416	2020-2-26 8:00	CN	WPRO	411	78190	52	2718	46	3.476	3.466
2417	2020-2-27 8:00	CN	WPRO	440	78630	29	2747	47	3.494	3.501
2418	2020-2-28 8:00	CN	WPRO	329	78959	44	2791	48	3.535	3.534
2419	2020-2-29 8:00	CN	WPRO	430	79389	47	2838	49	3.575	3.567
2420	2020-3-1 8:00	CN	WPRO	579	79968	35	2873	50	3.593	3.601
2421	2020-3-2 8:00	CN	WPRO	206	80174	42	2915	51	3.636	3.632
2422	2020-3-3 8:00	CN	WPRO	128	80302	31	2946	52	3.669	3.672
2423	2020-3-4 8:00	CN	WPRO	120	80422	38	2984	53	3.710	3.707
2424	2020-3-5 8:00	CN	WPRO	143	80565	31	3015	54	3.742	3.742
2425	2020-3-6 8:00	CN	WPRO	146	80711	30	3045	55	3.773	3.773
2426	2020-3-7 8:00	CN	WPRO	102	80813	28	3073	56	3.803	3.803
2427	2020-3-8 8:00	CN	WPRO	46	80859	27	3100	57	3.834	3.832
2428	2020-3-9 8:00	CN	WPRO	45	80904	23	3123	58	3.860	3.858
2429	2020-3-10 8:00	CN	WPRO	20	80924	17	3140	59	3.880	3.882
2430	2020-3-11 8:00	CN	WPRO	31	80955	22	3162	60	3.906	3.901
2431	2020-3-12 8:00	CN	WPRO	26	80981	11	3173	61	3.918	3.917
2432	2020-3-13 8:00	CN	WPRO	10	80991	7	3180	62	3.926	3.929
2433	2020-3-14 8:00	CN	WPRO	30	81021	14	3194	63	3.942	3.941
2434	2020-3-15 8:00	CN	WPRO	27	81048	10	3204	64	3.953	3.955
2435	2020-3-16 8:00	CN	WPRO	29	81077	14	3218	65	3.969	3.968
2436	2020-3-17 8:00	CN	WPRO	39	81116	13	3231	66	3.983	3.982
2437	2020-3-18 8:00	CN	WPRO	58	81174	11	3242	67	3.994	3.993
2438	2020-3-19 8:00	CN	WPRO	126	81300	11	3253	68	4.001	4.000
2439	2020-3-20 8:00	CN	WPRO	116	81416	8	3261	69	4.005	4.005
2440	2020-3-21 8:00	CN	WPRO	82	81498	6	3267	70	4.009	4.008
2441	2020-3-22 8:00	CN	WPRO	1	81499	0	3267	71	4.009	4.011
2442	2020-3-23 8:00	CN	WPRO	104	81603	9	3276	72	4.015	4.013
2443	2020-3-24 8:00	CN	WPRO	164	81767	7	3283	73	4.015	4.015
2444	2020-3-25 8:00	CN	WPRO	102	81869	4	3287	74	4.015	4.016
2445	2020-3-26 8:00	CN	WPRO	92	81961	6	3293	75	4.018	4.017
2446	2020-3-27 8:00	CN	WPRO	132	82093	5	3298	76	4.017	4.017
2447	2020-3-28 8:00	CN	WPRO	120	82213	3	3301	77	4.015	4.016
2448	2020-3-29 8:00	CN	WPRO	128	82341	5	3306	78	4.015	4.015

Table S1 Daily confirmed cases and daily deaths of COVID-19 in 214 nations from WHO

ID	Date and time	Country or region	Continent	Confirmed patients	Cumulative Confirmed patients	Daily dead patients	Daily cumulative dead patients	Lag days	Daily CFR	3DMA CFR
2449	2020-3-30 8:00	CN	WPRO	114	82455	4	3310	79	4.014	4.015
2450	2020-3-31 8:00	CN	WPRO	90	82545	4	3314	80	4.015	4.016
2451	2020-4-1 8:00	CN	WPRO	93	82638	7	3321	81	4.019	4.018
2452	2020-4-2 8:00	CN	WPRO	86	82724	6	3327	82	4.022	4.021
2453	2020-4-3 8:00	CN	WPRO	78	82802	4	3331	83	4.023	4.023
2454	2020-4-4 8:00	CN	WPRO	73	82875	4	3335	84	4.024	4.024
2455	2020-4-5 8:00	CN	WPRO	55	82930	3	3338	85	4.025	4.024
2456	2020-4-6 8:00	CN	WPRO	75	83005	2	3340	86	4.024	4.023
2457	2020-4-7 8:00	CN	WPRO	66	83071	0	3340	87	4.021	4.021
2458	2020-4-8 8:00	CN	WPRO	90	83161	2	3342	88	4.019	4.019
2459	2020-4-9 8:00	CN	WPRO	88	83249	2	3344	89	4.017	4.017
2460	2020-4-10 8:00	CN	WPRO	56	83305	1	3345	90	4.015	4.016
2461	2020-4-11 8:00	CN	WPRO	78	83383	4	3349	91	4.016	4.014
2462	2020-4-12 8:00	CN	WPRO	102	83485	0	3349	92	4.011	4.012
2463	2020-4-13 8:00	CN	WPRO	112	83597	2	3351	93	4.009	4.008
2464	2020-4-14 8:00	CN	WPRO	99	83696	0	3351	94	4.004	4.005
2465	2020-4-15 8:00	CN	WPRO	51	83747	1	3352	95	4.003	4.002
2466	2020-4-16 8:00	CN	WPRO	50	83797	0	3352	96	4.000	4.506
2467	2020-4-17 8:00	CN	WPRO	352	84149	1290	4642	97	5.516	5.010
2468	2020-4-18 8:00	CN	WPRO	31	84180	0	4642	98	5.514	5.514
2469	2020-4-19 8:00	CN	WPRO	43	84223	0	4642	99	5.512	5.512
2470	2020-4-20 8:00	CN	WPRO	16	84239	0	4642	100	5.511	5.511
2471	2020-4-21 8:00	CN	WPRO	14	84253	0	4642	101	5.510	5.509
2472	2020-4-22 8:00	CN	WPRO	35	84288	0	4642	102	5.507	5.508
2473	2020-4-23 8:00	CN	WPRO	15	84303	0	4642	103	5.506	5.506
2474	2020-4-24 8:00	CN	WPRO	9	84312	0	4642	104	5.506	5.506
2475	2020-4-25 8:00	CN	WPRO	12	84324	0	4642	105	5.505	5.505
2476	2020-4-26 8:00	CN	WPRO	14	84338	0	4642	106	5.504	5.505
2477	2020-4-27 8:00	CN	WPRO	3	84341	1	4643	107	5.505	5.505
2478	2020-4-28 8:00	CN	WPRO	6	84347	0	4643	108	5.505	5.504
2479	2020-4-29 8:00	CN	WPRO	22	84369	0	4643	109	5.503	5.504
2480	2020-4-30 8:00	CN	WPRO	4	84373	0	4643	110	5.503	5.503
2481	2020-5-1 8:00	CN	WPRO	12	84385	0	4643	111	5.502	5.502
2482	2020-5-2 8:00	CN	WPRO	3	84388	0	4643	112	5.502	5.502
2483										
2484	2020-3-6 8:00	CO	AMRO	1	1	0	0			

Table S1 Daily confirmed cases and daily deaths of COVID-19 in 214 nations from WHO

ID	Date and time	Country or region	Continent	Confirmed patients	Cumulative Confirmed patients	Daily dead patients	Daily cumulative dead patients	Lag days	Daily CFR	3DMA CFR
2485	2020-3-7 8:00	CO	AMRO	0	1	0	0			
2486	2020-3-8 8:00	CO	AMRO	0	1	0	0			
2487	2020-3-9 8:00	CO	AMRO	0	1	0	0			
2488	2020-3-10 8:00	CO	AMRO	2	3	0	0			
2489	2020-3-11 8:00	CO	AMRO	0	3	0	0			
2490	2020-3-12 8:00	CO	AMRO	6	9	0	0			
2491	2020-3-13 8:00	CO	AMRO	7	16	0	0			
2492	2020-3-14 8:00	CO	AMRO	8	24	0	0			
2493	2020-3-15 8:00	CO	AMRO	0	24	0	0			
2494	2020-3-16 8:00	CO	AMRO	10	34	0	0			
2495	2020-3-17 8:00	CO	AMRO	11	45	0	0			
2496	2020-3-18 8:00	CO	AMRO	48	93	0	0			
2497	2020-3-19 8:00	CO	AMRO	15	108	0	0			
2498	2020-3-20 8:00	CO	AMRO	37	145	0	0			
2499	2020-3-21 8:00	CO	AMRO	51	196	0	0			
2500	2020-3-22 8:00	CO	AMRO	0	196	0	0			
2501	2020-3-23 8:00	CO	AMRO	0	196	2	2	0	1.020	1.000
2502	2020-3-24 8:00	CO	AMRO	110	306	1	3	1	0.980	0.994
2503	2020-3-25 8:00	CO	AMRO	0	306	0	3	2	0.980	0.937
2504	2020-3-26 8:00	CO	AMRO	164	470	1	4	3	0.851	0.894
2505	2020-3-27 8:00	CO	AMRO	0	470	0	4	4	0.851	0.975
2506	2020-3-28 8:00	CO	AMRO	21	491	2	6	5	1.222	1.062
2507	2020-3-29 8:00	CO	AMRO	48	539	0	6	6	1.113	1.107
2508	2020-3-30 8:00	CO	AMRO	69	608	0	6	7	0.987	1.175
2509	2020-3-31 8:00	CO	AMRO	94	702	4	10	8	1.425	1.389
2510	2020-4-1 8:00	CO	AMRO	96	798	4	14	9	1.754	1.648
2511	2020-4-2 8:00	CO	AMRO	108	906	2	16	10	1.766	1.706
2512	2020-4-3 8:00	CO	AMRO	159	1065	1	17	11	1.596	1.666
2513	2020-4-4 8:00	CO	AMRO	96	1161	2	19	12	1.637	1.735
2514	2020-4-5 8:00	CO	AMRO	106	1267	6	25	13	1.973	1.962
2515	2020-4-6 8:00	CO	AMRO	139	1406	7	32	14	2.276	2.202
2516	2020-4-7 8:00	CO	AMRO	79	1485	3	35	15	2.357	2.515
2517	2020-4-8 8:00	CO	AMRO	94	1579	11	46	16	2.913	2.693
2518	2020-4-9 8:00	CO	AMRO	201	1780	4	50	17	2.809	2.800
2519	2020-4-10 8:00	CO	AMRO	274	2054	5	55	18	2.678	2.864
2520	2020-4-11 8:00	CO	AMRO	169	2223	14	69	19	3.104	3.006

Table S1 Daily confirmed cases and daily deaths of COVID-19 in 214 nations from WHO

ID	Date and time	Country or region	Continent	Confirmed patients	Cumulative Confirmed patients	Daily dead patients	Daily cumulative dead patients	Lag days	Daily CFR	3DMA CFR
2521	2020-4-12 8:00	CO	AMRO	250	2473	11	80	20	3.235	3.343
2522	2020-4-13 8:00	CO	AMRO	236	2709	20	100	21	3.691	3.618
2523	2020-4-14 8:00	CO	AMRO	67	2776	9	109	22	3.927	3.848
2524	2020-4-15 8:00	CO	AMRO	76	2852	3	112	23	3.927	4.039
2525	2020-4-16 8:00	CO	AMRO	127	2979	15	127	24	4.263	4.136
2526	2020-4-17 8:00	CO	AMRO	126	3105	4	131	25	4.219	4.312
2527	2020-4-18 8:00	CO	AMRO	128	3233	13	144	26	4.454	4.374
2528	2020-4-19 8:00	CO	AMRO	206	3439	9	153	27	4.449	4.496
2529	2020-4-20 8:00	CO	AMRO	182	3621	13	166	28	4.584	4.585
2530	2020-4-21 8:00	CO	AMRO	171	3792	13	179	29	4.720	4.686
2531	2020-4-22 8:00	CO	AMRO	185	3977	10	189	30	4.752	4.732
2532	2020-4-23 8:00	CO	AMRO	172	4149	7	196	31	4.724	4.735
2533	2020-4-24 8:00	CO	AMRO	207	4356	10	206	32	4.729	4.722
2534	2020-4-25 8:00	CO	AMRO	205	4561	9	215	33	4.714	4.684
2535	2020-4-26 8:00	CO	AMRO	320	4881	10	225	34	4.610	4.618
2536	2020-4-27 8:00	CO	AMRO	261	5142	8	233	35	4.531	4.559
2537	2020-4-28 8:00	CO	AMRO	237	5379	11	244	36	4.536	4.529
2538	2020-4-29 8:00	CO	AMRO	218	5597	9	253	37	4.520	4.526
2539	2020-4-30 8:00	CO	AMRO	352	5949	16	269	38	4.522	4.506
2540	2020-5-1 8:00	CO	AMRO	262	6211	9	278	39	4.476	4.500
2541	2020-5-2 8:00	CO	AMRO	296	6507	15	293	40	4.503	2.993
2542										
2543	2020-5-1 8:00	KM	AFRO	1	1	0	0	0	0.000	1.501
2544	2020-5-2 8:00	KM	AFRO	0	1	0	0	0	0.000	0.000
2545										
2546	2020-3-15 8:00	CG	AFRO	1	1	0	0			
2547	2020-3-16 8:00	CG	AFRO	0	1	0	0			
2548	2020-3-17 8:00	CG	AFRO	0	1	0	0			
2549	2020-3-18 8:00	CG	AFRO	2	3	0	0			
2550	2020-3-19 8:00	CG	AFRO	0	3	0	0			
2551	2020-3-20 8:00	CG	AFRO	0	3	0	0			
2552	2020-3-21 8:00	CG	AFRO	1	4	0	0			
2553	2020-3-22 8:00	CG	AFRO	0	4	0	0			
2554	2020-3-23 8:00	CG	AFRO	0	4	0	0			
2555	2020-3-24 8:00	CG	AFRO	0	4	0	0			
2556	2020-3-25 8:00	CG	AFRO	0	4	0	0			

Table S1 Daily confirmed cases and daily deaths of COVID-19 in 214 nations from WHO

ID	Date and time	Country or region	Continent	Confirmed patients	Cumulative Confirmed patients	Daily dead patients	Daily cumulative dead patients	Lag days	Daily CFR	3DMA CFR
2557	2020-3-26 8:00	CG	AFRO	0	4	0	0			
2558	2020-3-27 8:00	CG	AFRO	0	4	0	0			
2559	2020-3-28 8:00	CG	AFRO	15	19	0	0			
2560	2020-3-29 8:00	CG	AFRO	0	19	0	0			
2561	2020-3-30 8:00	CG	AFRO	0	19	0	0			
2562	2020-3-31 8:00	CG	AFRO	0	19	0	0			
2563	2020-4-1 8:00	CG	AFRO	3	22	2	2	0	9.091	9.091
2564	2020-4-2 8:00	CG	AFRO	0	22	0	2	1	9.091	7.687
2565	2020-4-3 8:00	CG	AFRO	19	41	0	2	2	4.878	7.095
2566	2020-4-4 8:00	CG	AFRO	0	41	1	3	3	7.317	7.769
2567	2020-4-5 8:00	CG	AFRO	4	45	2	5	4	11.111	9.846
2568	2020-4-6 8:00	CG	AFRO	0	45	0	5	5	11.111	11.111
2569	2020-4-7 8:00	CG	AFRO	0	45	0	5	6	11.111	10.809
2570	2020-4-8 8:00	CG	AFRO	4	49	0	5	7	10.204	9.883
2571	2020-4-9 8:00	CG	AFRO	11	60	0	5	8	8.333	8.957
2572	2020-4-10 8:00	CG	AFRO	0	60	0	5	9	8.333	8.333
2573	2020-4-11 8:00	CG	AFRO	0	60	0	5	10	8.333	8.333
2574	2020-4-12 8:00	CG	AFRO	0	60	0	5	11	8.333	7.937
2575	2020-4-13 8:00	CG	AFRO	10	70	0	5	12	7.143	7.411
2576	2020-4-14 8:00	CG	AFRO	4	74	0	5	13	6.757	6.885
2577	2020-4-15 8:00	CG	AFRO	0	74	0	5	14	6.757	5.929
2578	2020-4-16 8:00	CG	AFRO	43	117	0	5	15	4.274	5.101
2579	2020-4-17 8:00	CG	AFRO	0	117	0	5	16	4.274	4.248
2580	2020-4-18 8:00	CG	AFRO	26	143	1	6	17	4.196	4.222
2581	2020-4-19 8:00	CG	AFRO	0	143	0	6	18	4.196	4.196
2582	2020-4-20 8:00	CG	AFRO	0	143	0	6	19	4.196	4.047
2583	2020-4-21 8:00	CG	AFRO	17	160	0	6	20	3.750	3.861
2584	2020-4-22 8:00	CG	AFRO	5	165	0	6	21	3.636	3.537
2585	2020-4-23 8:00	CG	AFRO	21	186	0	6	22	3.226	3.363
2586	2020-4-24 8:00	CG	AFRO	0	186	0	6	23	3.226	3.151
2587	2020-4-25 8:00	CG	AFRO	14	200	0	6	24	3.000	3.409
2588	2020-4-26 8:00	CG	AFRO	0	200	2	8	25	4.000	3.667
2589	2020-4-27 8:00	CG	AFRO	0	200	0	8	26	4.000	3.955
2590	2020-4-28 8:00	CG	AFRO	7	207	0	8	27	3.865	3.910
2591	2020-4-29 8:00	CG	AFRO	0	207	0	8	28	3.865	3.940
2592	2020-4-30 8:00	CG	AFRO	13	220	1	9	29	4.091	4.016

Table S1 Daily confirmed cases and daily deaths of COVID-19 in 214 nations from WHO

ID	Date and time	Country or region	Continent	Confirmed patients	Cumulative Confirmed patients	Daily dead patients	Daily cumulative dead patients	Lag days	Daily CFR	3DMA CFR
2593	2020-5-1 8:00	CG	AFRO	0	220	0	9	30	4.091	4.183
2594	2020-5-2 8:00	CG	AFRO	9	229	1	10	31	4.367	4.229
2595										
2596	2020-3-8 8:00	CR	AMRO	5	5	0	0			
2597	2020-3-9 8:00	CR	AMRO	4	9	0	0			
2598	2020-3-10 8:00	CR	AMRO	0	9	0	0			
2599	2020-3-11 8:00	CR	AMRO	4	13	0	0			
2600	2020-3-12 8:00	CR	AMRO	0	13	0	0			
2601	2020-3-13 8:00	CR	AMRO	10	23	0	0			
2602	2020-3-14 8:00	CR	AMRO	0	23	0	0			
2603	2020-3-15 8:00	CR	AMRO	0	23	0	0			
2604	2020-3-16 8:00	CR	AMRO	12	35	0	0			
2605	2020-3-17 8:00	CR	AMRO	6	41	0	0			
2606	2020-3-18 8:00	CR	AMRO	9	50	1	1	0	2.000	1.575
2607	2020-3-19 8:00	CR	AMRO	37	87	0	1	1	1.149	1.640
2608	2020-3-20 8:00	CR	AMRO	26	113	1	2	2	1.770	1.563
2609	2020-3-21 8:00	CR	AMRO	0	113	0	2	3	1.770	1.770
2610	2020-3-22 8:00	CR	AMRO	0	113	0	2	4	1.770	1.750
2611	2020-3-23 8:00	CR	AMRO	4	117	0	2	5	1.709	1.536
2612	2020-3-24 8:00	CR	AMRO	60	177	0	2	6	1.130	1.323
2613	2020-3-25 8:00	CR	AMRO	0	177	0	2	7	1.130	1.085
2614	2020-3-26 8:00	CR	AMRO	24	201	0	2	8	0.995	1.040
2615	2020-3-27 8:00	CR	AMRO	0	201	0	2	9	0.995	0.952
2616	2020-3-28 8:00	CR	AMRO	30	231	0	2	10	0.866	0.874
2617	2020-3-29 8:00	CR	AMRO	32	263	0	2	11	0.760	0.768
2618	2020-3-30 8:00	CR	AMRO	32	295	0	2	12	0.678	0.692
2619	2020-3-31 8:00	CR	AMRO	19	314	0	2	13	0.637	0.651
2620	2020-4-1 8:00	CR	AMRO	0	314	0	2	14	0.637	0.617
2621	2020-4-2 8:00	CR	AMRO	33	347	0	2	15	0.576	0.582
2622	2020-4-3 8:00	CR	AMRO	28	375	0	2	16	0.533	0.538
2623	2020-4-4 8:00	CR	AMRO	21	396	0	2	17	0.505	0.506
2624	2020-4-5 8:00	CR	AMRO	20	416	0	2	18	0.481	0.482
2625	2020-4-6 8:00	CR	AMRO	19	435	0	2	19	0.460	0.460
2626	2020-4-7 8:00	CR	AMRO	19	454	0	2	20	0.441	0.443
2627	2020-4-8 8:00	CR	AMRO	13	467	0	2	21	0.428	0.428
2628	2020-4-9 8:00	CR	AMRO	16	483	0	2	22	0.414	0.480

Table S1 Daily confirmed cases and daily deaths of COVID-19 in 214 nations from WHO

ID	Date and time	Country or region	Continent	Confirmed patients	Cumulative Confirmed patients	Daily dead patients	Daily cumulative dead patients	Lag days	Daily CFR	3DMA CFR
2629	2020-4-10 8:00	CR	AMRO	19	502	1	3	23	0.598	0.523
2630	2020-4-11 8:00	CR	AMRO	37	539	0	3	24	0.557	0.564
2631	2020-4-12 8:00	CR	AMRO	19	558	0	3	25	0.538	0.538
2632	2020-4-13 8:00	CR	AMRO	19	577	0	3	26	0.520	0.521
2633	2020-4-14 8:00	CR	AMRO	18	595	0	3	27	0.504	0.505
2634	2020-4-15 8:00	CR	AMRO	17	612	0	3	28	0.490	0.493
2635	2020-4-16 8:00	CR	AMRO	6	618	0	3	29	0.485	0.538
2636	2020-4-17 8:00	CR	AMRO	8	626	1	4	30	0.639	0.582
2637	2020-4-18 8:00	CR	AMRO	16	642	0	4	31	0.623	0.626
2638	2020-4-19 8:00	CR	AMRO	7	649	0	4	32	0.616	0.617
2639	2020-4-20 8:00	CR	AMRO	6	655	0	4	33	0.611	0.662
2640	2020-4-21 8:00	CR	AMRO	5	660	1	5	34	0.758	0.758
2641	2020-4-22 8:00	CR	AMRO	2	662	1	6	35	0.906	0.854
2642	2020-4-23 8:00	CR	AMRO	7	669	0	6	36	0.897	0.895
2643	2020-4-24 8:00	CR	AMRO	12	681	0	6	37	0.881	0.884
2644	2020-4-25 8:00	CR	AMRO	5	686	0	6	38	0.875	0.876
2645	2020-4-26 8:00	CR	AMRO	1	687	0	6	39	0.873	0.871
2646	2020-4-27 8:00	CR	AMRO	6	693	0	6	40	0.866	0.867
2647	2020-4-28 8:00	CR	AMRO	2	695	0	6	41	0.863	0.863
2648	2020-4-29 8:00	CR	AMRO	2	697	0	6	42	0.861	0.858
2649	2020-4-30 8:00	CR	AMRO	8	705	0	6	43	0.851	0.851
2650	2020-5-1 8:00	CR	AMRO	8	713	0	6	44	0.842	0.842
2651	2020-5-2 8:00	CR	AMRO	6	719	0	6	45	0.834	0.838
2652										
2653	2020-3-12 8:00	CI	AFRO	1	1	0	0			
2654	2020-3-13 8:00	CI	AFRO	0	1	0	0			
2655	2020-3-14 8:00	CI	AFRO	2	3	0	0			
2656	2020-3-15 8:00	CI	AFRO	0	3	0	0			
2657	2020-3-16 8:00	CI	AFRO	0	3	0	0			
2658	2020-3-17 8:00	CI	AFRO	3	6	0	0			
2659	2020-3-18 8:00	CI	AFRO	3	9	0	0			
2660	2020-3-19 8:00	CI	AFRO	0	9	0	0			
2661	2020-3-20 8:00	CI	AFRO	0	9	0	0			
2662	2020-3-21 8:00	CI	AFRO	0	9	0	0			
2663	2020-3-22 8:00	CI	AFRO	0	9	0	0			
2664	2020-3-23 8:00	CI	AFRO	16	25	0	0			

Table S1 Daily confirmed cases and daily deaths of COVID-19 in 214 nations from WHO

ID	Date and time	Country or region	Continent	Confirmed patients	Cumulative Confirmed patients	Daily dead patients	Daily cumulative dead patients	Lag days	Daily CFR	3DMA CFR
2665	2020-3-24 8:00	CI	AFRO	0	25	0	0			
2666	2020-3-25 8:00	CI	AFRO	47	72	0	0			
2667	2020-3-26 8:00	CI	AFRO	8	80	0	0			
2668	2020-3-27 8:00	CI	AFRO	0	80	0	0			
2669	2020-3-28 8:00	CI	AFRO	12	92	0	0			
2670	2020-3-29 8:00	CI	AFRO	48	140	0	0			
2671	2020-3-30 8:00	CI	AFRO	25	165	0	0			
2672	2020-3-31 8:00	CI	AFRO	4	169	0	0			
2673	2020-4-1 8:00	CI	AFRO	0	169	0	0			
2674	2020-4-2 8:00	CI	AFRO	21	190	1	1	0	0.526	0.526
2675	2020-4-3 8:00	CI	AFRO	0	190	0	1	1	0.526	0.504
2676	2020-4-4 8:00	CI	AFRO	28	218	0	1	2	0.459	0.600
2677	2020-4-5 8:00	CI	AFRO	27	245	1	2	3	0.816	0.680
2678	2020-4-6 8:00	CI	AFRO	16	261	0	2	4	0.766	0.837
2679	2020-4-7 8:00	CI	AFRO	62	323	1	3	5	0.929	0.852
2680	2020-4-8 8:00	CI	AFRO	26	349	0	3	6	0.860	0.857
2681	2020-4-9 8:00	CI	AFRO	35	384	0	3	7	0.781	0.807
2682	2020-4-10 8:00	CI	AFRO	0	384	0	3	8	0.781	0.729
2683	2020-4-11 8:00	CI	AFRO	96	480	0	3	9	0.625	0.719
2684	2020-4-12 8:00	CI	AFRO	53	533	1	4	10	0.750	0.709
2685	2020-4-13 8:00	CI	AFRO	0	533	0	4	11	0.750	0.820
2686	2020-4-14 8:00	CI	AFRO	93	626	2	6	12	0.958	0.883
2687	2020-4-15 8:00	CI	AFRO	12	638	0	6	13	0.940	0.939
2688	2020-4-16 8:00	CI	AFRO	16	654	0	6	14	0.917	0.910
2689	2020-4-17 8:00	CI	AFRO	34	688	0	6	15	0.872	0.866
2690	2020-4-18 8:00	CI	AFRO	54	742	0	6	16	0.809	0.830
2691	2020-4-19 8:00	CI	AFRO	0	742	0	6	17	0.809	0.893
2692	2020-4-20 8:00	CI	AFRO	105	847	3	9	18	1.063	1.003
2693	2020-4-21 8:00	CI	AFRO	32	879	1	10	19	1.138	1.206
2694	2020-4-22 8:00	CI	AFRO	37	916	3	13	20	1.419	1.342
2695	2020-4-23 8:00	CI	AFRO	36	952	1	14	21	1.471	1.428
2696	2020-4-24 8:00	CI	AFRO	52	1004	0	14	22	1.394	1.388
2697	2020-4-25 8:00	CI	AFRO	73	1077	0	14	23	1.300	1.318
2698	2020-4-26 8:00	CI	AFRO	34	1111	0	14	24	1.260	1.259
2699	2020-4-27 8:00	CI	AFRO	39	1150	0	14	25	1.217	1.227
2700	2020-4-28 8:00	CI	AFRO	14	1164	0	14	26	1.203	1.201

Table S1 Daily confirmed cases and daily deaths of COVID-19 in 214 nations from WHO

ID	Date and time	Country or region	Continent	Confirmed patients	Cumulative Confirmed patients	Daily dead patients	Daily cumulative dead patients	Lag days	Daily CFR	3DMA CFR
2701	2020-4-29 8:00	CI	AFRO	19	1183	0	14	27	1.183	1.172
2702	2020-4-30 8:00	CI	AFRO	55	1238	0	14	28	1.131	1.137
2703	2020-5-1 8:00	CI	AFRO	37	1275	0	14	29	1.098	1.109
2704	2020-5-2 8:00	CI	AFRO	0	1275	0	14	30	1.098	1.098
2705										
2706	2020-2-25 8:00	HR	EURO	1	1	0	0			
2707	2020-2-26 8:00	HR	EURO	2	3	0	0			
2708	2020-2-27 8:00	HR	EURO	0	3	0	0			
2709	2020-2-28 8:00	HR	EURO	2	5	0	0			
2710	2020-2-29 8:00	HR	EURO	2	7	0	0			
2711	2020-3-1 8:00	HR	EURO	0	7	0	0			
2712	2020-3-2 8:00	HR	EURO	1	8	0	0			
2713	2020-3-3 8:00	HR	EURO	1	9	0	0			
2714	2020-3-4 8:00	HR	EURO	0	9	0	0			
2715	2020-3-5 8:00	HR	EURO	1	10	0	0			
2716	2020-3-6 8:00	HR	EURO	1	11	0	0			
2717	2020-3-7 8:00	HR	EURO	0	11	0	0			
2718	2020-3-8 8:00	HR	EURO	0	11	0	0			
2719	2020-3-9 8:00	HR	EURO	1	12	0	0			
2720	2020-3-10 8:00	HR	EURO	0	12	0	0			
2721	2020-3-11 8:00	HR	EURO	8	20	0	0			
2722	2020-3-12 8:00	HR	EURO	0	20	0	0			
2723	2020-3-13 8:00	HR	EURO	11	31	0	0			
2724	2020-3-14 8:00	HR	EURO	0	31	0	0			
2725	2020-3-15 8:00	HR	EURO	6	37	0	0			
2726	2020-3-16 8:00	HR	EURO	19	56	0	0			
2727	2020-3-17 8:00	HR	EURO	9	65	0	0			
2728	2020-3-18 8:00	HR	EURO	16	81	0	0			
2729	2020-3-19 8:00	HR	EURO	0	81	0	0			
2730	2020-3-20 8:00	HR	EURO	45	126	0	0			
2731	2020-3-21 8:00	HR	EURO	80	206	0	0			
2732	2020-3-22 8:00	HR	EURO	0	206	0	0			
2733	2020-3-23 8:00	HR	EURO	29	235	0	0			
2734	2020-3-24 8:00	HR	EURO	71	306	0	0			
2735	2020-3-25 8:00	HR	EURO	76	382	1	1	0	0.262	0.251
2736	2020-3-26 8:00	HR	EURO	36	418	0	1	1	0.239	0.302

Table S1 Daily confirmed cases and daily deaths of COVID-19 in 214 nations from WHO

ID	Date and time	Country or region	Continent	Confirmed patients	Cumulative Confirmed patients	Daily dead patients	Daily cumulative dead patients	Lag days	Daily CFR	3DMA CFR
2737	2020-3-27 8:00	HR	EURO	77	495	1	2	2	0.404	0.385
2738	2020-3-28 8:00	HR	EURO	91	586	1	3	3	0.512	0.559
2739	2020-3-29 8:00	HR	EURO	71	657	2	5	4	0.761	0.705
2740	2020-3-30 8:00	HR	EURO	56	713	1	6	5	0.842	0.787
2741	2020-3-31 8:00	HR	EURO	77	790	0	6	6	0.759	0.764
2742	2020-4-1 8:00	HR	EURO	77	867	0	6	7	0.692	0.692
2743	2020-4-2 8:00	HR	EURO	96	963	0	6	8	0.623	0.669
2744	2020-4-3 8:00	HR	EURO	48	1011	1	7	9	0.692	0.686
2745	2020-4-4 8:00	HR	EURO	68	1079	1	8	10	0.741	0.833
2746	2020-4-5 8:00	HR	EURO	47	1126	4	12	11	1.066	1.025
2747	2020-4-6 8:00	HR	EURO	56	1182	3	15	12	1.269	1.215
2748	2020-4-7 8:00	HR	EURO	40	1222	1	16	13	1.309	1.327
2749	2020-4-8 8:00	HR	EURO	60	1282	2	18	14	1.404	1.376
2750	2020-4-9 8:00	HR	EURO	61	1343	1	19	15	1.415	1.413
2751	2020-4-10 8:00	HR	EURO	64	1407	1	20	16	1.421	1.414
2752	2020-4-11 8:00	HR	EURO	88	1495	1	21	17	1.405	1.398
2753	2020-4-12 8:00	HR	EURO	39	1534	0	21	18	1.369	1.404
2754	2020-4-13 8:00	HR	EURO	66	1600	2	23	19	1.438	1.441
2755	2020-4-14 8:00	HR	EURO	50	1650	2	25	20	1.515	1.591
2756	2020-4-15 8:00	HR	EURO	54	1704	6	31	21	1.819	1.762
2757	2020-4-16 8:00	HR	EURO	37	1741	3	34	22	1.953	1.909
2758	2020-4-17 8:00	HR	EURO	50	1791	1	35	23	1.954	1.964
2759	2020-4-18 8:00	HR	EURO	23	1814	1	36	24	1.985	2.023
2760	2020-4-19 8:00	HR	EURO	18	1832	3	39	25	2.129	2.208
2761	2020-4-20 8:00	HR	EURO	39	1871	8	47	26	2.512	2.380
2762	2020-4-21 8:00	HR	EURO	10	1881	0	47	27	2.499	2.509
2763	2020-4-22 8:00	HR	EURO	27	1908	1	48	28	2.516	2.492
2764	2020-4-23 8:00	HR	EURO	42	1950	0	48	29	2.462	2.500
2765	2020-4-24 8:00	HR	EURO	31	1981	2	50	30	2.524	2.508
2766	2020-4-25 8:00	HR	EURO	28	2009	1	51	31	2.539	2.580
2767	2020-4-26 8:00	HR	EURO	7	2016	3	54	32	2.679	2.642
2768	2020-4-27 8:00	HR	EURO	14	2030	1	55	33	2.709	2.761
2769	2020-4-28 8:00	HR	EURO	9	2039	4	59	34	2.894	2.894
2770	2020-4-29 8:00	HR	EURO	8	2047	4	63	35	3.078	3.074
2771	2020-4-30 8:00	HR	EURO	15	2062	4	67	36	3.249	3.217
2772	2020-5-1 8:00	HR	EURO	14	2076	2	69	37	3.324	3.390

Table S1 Daily confirmed cases and daily deaths of COVID-19 in 214 nations from WHO

ID	Date and time	Country or region	Continent	Confirmed patients	Cumulative Confirmed patients	Daily dead patients	Daily cumulative dead patients	Lag days	Daily CFR	3DMA CFR
2773	2020-5-2 8:00	HR	EURO	9	2085	6	75	38	3.597	3.460
2774										
2775	2020-3-13 8:00	CU	AMRO	4	4	0	0			
2776	2020-3-14 8:00	CU	AMRO	0	4	0	0			
2777	2020-3-15 8:00	CU	AMRO	0	4	0	0			
2778	2020-3-16 8:00	CU	AMRO	0	4	0	0			
2779	2020-3-17 8:00	CU	AMRO	1	5	0	0			
2780	2020-3-18 8:00	CU	AMRO	5	10	1	1	0	10.000	9.545
2781	2020-3-19 8:00	CU	AMRO	1	11	0	1	1	9.091	8.447
2782	2020-3-20 8:00	CU	AMRO	5	16	0	1	2	6.250	7.197
2783	2020-3-21 8:00	CU	AMRO	0	16	0	1	3	6.250	6.250
2784	2020-3-22 8:00	CU	AMRO	0	16	0	1	4	6.250	6.250
2785	2020-3-23 8:00	CU	AMRO	0	16	0	1	5	6.250	4.861
2786	2020-3-24 8:00	CU	AMRO	32	48	0	1	6	2.083	3.472
2787	2020-3-25 8:00	CU	AMRO	0	48	0	1	7	2.083	1.886
2788	2020-3-26 8:00	CU	AMRO	19	67	0	1	8	1.493	1.689
2789	2020-3-27 8:00	CU	AMRO	0	67	0	1	9	1.493	1.828
2790	2020-3-28 8:00	CU	AMRO	13	80	1	2	10	2.500	2.171
2791	2020-3-29 8:00	CU	AMRO	39	119	1	3	11	2.521	2.514
2792	2020-3-30 8:00	CU	AMRO	0	119	0	3	12	2.521	2.465
2793	2020-3-31 8:00	CU	AMRO	51	170	1	4	13	2.353	2.700
2794	2020-4-1 8:00	CU	AMRO	16	186	2	6	14	3.226	2.803
2795	2020-4-2 8:00	CU	AMRO	26	212	0	6	15	2.830	2.877
2796	2020-4-3 8:00	CU	AMRO	21	233	0	6	16	2.575	2.545
2797	2020-4-4 8:00	CU	AMRO	36	269	0	6	17	2.230	2.296
2798	2020-4-5 8:00	CU	AMRO	19	288	0	6	18	2.083	2.271
2799	2020-4-6 8:00	CU	AMRO	32	320	2	8	19	2.500	2.385
2800	2020-4-7 8:00	CU	AMRO	30	350	1	9	20	2.571	2.616
2801	2020-4-8 8:00	CU	AMRO	46	396	2	11	21	2.778	2.658
2802	2020-4-9 8:00	CU	AMRO	61	457	1	12	22	2.626	2.772
2803	2020-4-10 8:00	CU	AMRO	58	515	3	15	23	2.913	2.733
2804	2020-4-11 8:00	CU	AMRO	49	564	0	15	24	2.660	2.718
2805	2020-4-12 8:00	CU	AMRO	56	620	1	16	25	2.581	2.644
2806	2020-4-13 8:00	CU	AMRO	49	669	2	18	26	2.691	2.721
2807	2020-4-14 8:00	CU	AMRO	57	726	3	21	27	2.893	2.775
2808	2020-4-15 8:00	CU	AMRO	40	766	0	21	28	2.742	2.861

Table S1 Daily confirmed cases and daily deaths of COVID-19 in 214 nations from WHO

ID	Date and time	Country or region	Continent	Confirmed patients	Cumulative Confirmed patients	Daily dead patients	Daily cumulative dead patients	Lag days	Daily CFR	3DMA CFR
2809	2020-4-16 8:00	CU	AMRO	48	814	3	24	29	2.948	2.941
2810	2020-4-17 8:00	CU	AMRO	48	862	3	27	30	3.132	3.146
2811	2020-4-18 8:00	CU	AMRO	61	923	4	31	31	3.359	3.245
2812	2020-4-19 8:00	CU	AMRO	63	986	1	32	32	3.245	3.296
2813	2020-4-20 8:00	CU	AMRO	49	1035	2	34	33	3.285	3.281
2814	2020-4-21 8:00	CU	AMRO	52	1087	2	36	34	3.312	3.313
2815	2020-4-22 8:00	CU	AMRO	50	1137	2	38	35	3.342	3.339
2816	2020-4-23 8:00	CU	AMRO	52	1189	2	40	36	3.364	3.396
2817	2020-4-24 8:00	CU	AMRO	46	1235	3	43	37	3.482	3.553
2818	2020-4-25 8:00	CU	AMRO	50	1285	6	49	38	3.813	3.703
2819	2020-4-26 8:00	CU	AMRO	52	1337	2	51	39	3.815	3.857
2820	2020-4-27 8:00	CU	AMRO	32	1369	3	54	40	3.944	3.930
2821	2020-4-28 8:00	CU	AMRO	20	1389	2	56	41	4.032	4.004
2822	2020-4-29 8:00	CU	AMRO	48	1437	2	58	42	4.036	4.007
2823	2020-4-30 8:00	CU	AMRO	30	1467	0	58	43	3.954	4.018
2824	2020-5-1 8:00	CU	AMRO	34	1501	3	61	44	4.064	4.061
2825	2020-5-2 8:00	CU	AMRO	36	1537	3	64	45	4.164	4.114
2826										
2827	2020-3-13 8:00	CW	AMRO	1	1	0	0			
2828	2020-3-14 8:00	CW	AMRO	0	1	0	0			
2829	2020-3-15 8:00	CW	AMRO	1	2	0	0			
2830	2020-3-16 8:00	CW	AMRO	0	2	0	0			
2831	2020-3-17 8:00	CW	AMRO	1	3	0	0			
2832	2020-3-18 8:00	CW	AMRO	0	3	0	0			
2833	2020-3-19 8:00	CW	AMRO	0	3	0	0			
2834	2020-3-20 8:00	CW	AMRO	0	3	0	0			
2835	2020-3-21 8:00	CW	AMRO	0	3	1	1	0	33.333	33.333
2836	2020-3-22 8:00	CW	AMRO	0	3	0	1	1	33.333	33.333
2837	2020-3-23 8:00	CW	AMRO	0	3	0	1	2	33.333	27.778
2838	2020-3-24 8:00	CW	AMRO	3	6	0	1	3	16.667	22.222
2839	2020-3-25 8:00	CW	AMRO	0	6	0	1	4	16.667	15.873
2840	2020-3-26 8:00	CW	AMRO	1	7	0	1	5	14.286	15.079
2841	2020-3-27 8:00	CW	AMRO	0	7	0	1	6	14.286	14.286
2842	2020-3-28 8:00	CW	AMRO	0	7	0	1	7	14.286	14.286
2843	2020-3-29 8:00	CW	AMRO	0	7	0	1	8	14.286	14.286
2844	2020-3-30 8:00	CW	AMRO	0	7	0	1	9	14.286	12.554

Table S1 Daily confirmed cases and daily deaths of COVID-19 in 214 nations from WHO

ID	Date and time	Country or region	Continent	Confirmed patients	Cumulative Confirmed patients	Daily dead patients	Daily cumulative dead patients	Lag days	Daily CFR	3DMA CFR
2845	2020-3-31 8:00	CW	AMRO	4	11	0	1	10	9.091	10.823
2846	2020-4-1 8:00	CW	AMRO	0	11	0	1	11	9.091	9.091
2847	2020-4-2 8:00	CW	AMRO	0	11	0	1	12	9.091	9.091
2848	2020-4-3 8:00	CW	AMRO	0	11	0	1	13	9.091	9.091
2849	2020-4-4 8:00	CW	AMRO	0	11	0	1	14	9.091	9.091
2850	2020-4-5 8:00	CW	AMRO	0	11	0	1	15	9.091	9.091
2851	2020-4-6 8:00	CW	AMRO	0	11	0	1	16	9.091	8.625
2852	2020-4-7 8:00	CW	AMRO	2	13	0	1	17	7.692	8.159
2853	2020-4-8 8:00	CW	AMRO	0	13	0	1	18	7.692	7.509
2854	2020-4-9 8:00	CW	AMRO	1	14	0	1	19	7.143	7.326
2855	2020-4-10 8:00	CW	AMRO	0	14	0	1	20	7.143	7.143
2856	2020-4-11 8:00	CW	AMRO	0	14	0	1	21	7.143	7.143
2857	2020-4-12 8:00	CW	AMRO	0	14	0	1	22	7.143	7.143
2858	2020-4-13 8:00	CW	AMRO	0	14	0	1	23	7.143	7.143
2859	2020-4-14 8:00	CW	AMRO	0	14	0	1	24	7.143	7.143
2860	2020-4-15 8:00	CW	AMRO	0	14	0	1	25	7.143	7.143
2861	2020-4-16 8:00	CW	AMRO	0	14	0	1	26	7.143	7.143
2862	2020-4-17 8:00	CW	AMRO	0	14	0	1	27	7.143	7.143
2863	2020-4-18 8:00	CW	AMRO	0	14	0	1	28	7.143	7.143
2864	2020-4-19 8:00	CW	AMRO	0	14	0	1	29	7.143	7.143
2865	2020-4-20 8:00	CW	AMRO	0	14	0	1	30	7.143	7.143
2866	2020-4-21 8:00	CW	AMRO	0	14	0	1	31	7.143	7.143
2867	2020-4-22 8:00	CW	AMRO	0	14	0	1	32	7.143	7.143
2868	2020-4-23 8:00	CW	AMRO	0	14	0	1	33	7.143	7.143
2869	2020-4-24 8:00	CW	AMRO	0	14	0	1	34	7.143	7.143
2870	2020-4-25 8:00	CW	AMRO	0	14	0	1	35	7.143	7.143
2871	2020-4-26 8:00	CW	AMRO	0	14	0	1	36	7.143	7.143
2872	2020-4-27 8:00	CW	AMRO	0	14	0	1	37	7.143	6.845
2873	2020-4-28 8:00	CW	AMRO	2	16	0	1	38	6.250	6.548
2874	2020-4-29 8:00	CW	AMRO	0	16	0	1	39	6.250	6.250
2875	2020-4-30 8:00	CW	AMRO	0	16	0	1	40	6.250	6.250
2876	2020-5-1 8:00	CW	AMRO	0	16	0	1	41	6.250	6.250
2877	2020-5-2 8:00	CW	AMRO	0	16	0	1	42	6.250	6.250
2878										
2879	2020-3-9 8:00	CY	EURO	2	2	0	0			
2880	2020-3-10 8:00	CY	EURO	0	2	0	0			

Table S1 Daily confirmed cases and daily deaths of COVID-19 in 214 nations from WHO

ID	Date and time	Country or region	Continent	Confirmed patients	Cumulative Confirmed patients	Daily dead patients	Daily cumulative dead patients	Lag days	Daily CFR	3DMA CFR
2881	2020-3-11 8:00	CY	EURO	0	2	0	0			
2882	2020-3-12 8:00	CY	EURO	4	6	0	0			
2883	2020-3-13 8:00	CY	EURO	8	14	0	0			
2884	2020-3-14 8:00	CY	EURO	7	21	0	0			
2885	2020-3-15 8:00	CY	EURO	9	30	0	0			
2886	2020-3-16 8:00	CY	EURO	3	33	0	0			
2887	2020-3-17 8:00	CY	EURO	0	33	0	0			
2888	2020-3-18 8:00	CY	EURO	25	58	0	0			
2889	2020-3-19 8:00	CY	EURO	0	58	0	0			
2890	2020-3-20 8:00	CY	EURO	9	67	0	0			
2891	2020-3-21 8:00	CY	EURO	17	84	0	0			
2892	2020-3-22 8:00	CY	EURO	0	84	0	0			
2893	2020-3-23 8:00	CY	EURO	11	95	0	0			
2894	2020-3-24 8:00	CY	EURO	21	116	0	0			
2895	2020-3-25 8:00	CY	EURO	8	124	3	3	0	2.419	2.346
2896	2020-3-26 8:00	CY	EURO	8	132	0	3	1	2.273	2.249
2897	2020-3-27 8:00	CY	EURO	14	146	0	3	2	2.055	2.471
2898	2020-3-28 8:00	CY	EURO	16	162	2	5	3	3.086	2.645
2899	2020-3-29 8:00	CY	EURO	17	179	0	5	4	2.793	2.894
2900	2020-3-30 8:00	CY	EURO	35	214	1	6	5	2.804	2.880
2901	2020-3-31 8:00	CY	EURO	16	230	1	7	6	3.043	2.967
2902	2020-4-1 8:00	CY	EURO	32	262	1	8	7	3.053	2.970
2903	2020-4-2 8:00	CY	EURO	58	320	1	9	8	2.813	2.798
2904	2020-4-3 8:00	CY	EURO	36	356	0	9	9	2.528	2.706
2905	2020-4-4 8:00	CY	EURO	40	396	2	11	10	2.778	2.629
2906	2020-4-5 8:00	CY	EURO	30	426	0	11	11	2.582	2.833
2907	2020-4-6 8:00	CY	EURO	20	446	3	14	12	3.139	2.911
2908	2020-4-7 8:00	CY	EURO	19	465	0	14	13	3.011	2.995
2909	2020-4-8 8:00	CY	EURO	29	494	0	14	14	2.834	2.835
2910	2020-4-9 8:00	CY	EURO	32	526	0	14	15	2.662	2.718
2911	2020-4-10 8:00	CY	EURO	38	564	1	15	16	2.660	2.614
2912	2020-4-11 8:00	CY	EURO	31	595	0	15	17	2.521	2.539
2913	2020-4-12 8:00	CY	EURO	21	616	0	15	18	2.435	2.495
2914	2020-4-13 8:00	CY	EURO	17	633	1	16	19	2.528	2.510
2915	2020-4-14 8:00	CY	EURO	29	662	1	17	20	2.568	2.514
2916	2020-4-15 8:00	CY	EURO	33	695	0	17	21	2.446	2.464

Table S1 Daily confirmed cases and daily deaths of COVID-19 in 214 nations from WHO

ID	Date and time	Country or region	Continent	Confirmed patients	Cumulative Confirmed patients	Daily dead patients	Daily cumulative dead patients	Lag days	Daily CFR	3DMA CFR
2917	2020-4-16 8:00	CY	EURO	20	715	0	17	22	2.378	2.379
2918	2020-4-17 8:00	CY	EURO	20	735	0	17	23	2.313	2.334
2919	2020-4-18 8:00	CY	EURO	0	735	0	17	24	2.313	2.287
2920	2020-4-19 8:00	CY	EURO	26	761	0	17	25	2.234	2.254
2921	2020-4-20 8:00	CY	EURO	6	767	0	17	26	2.216	2.217
2922	2020-4-21 8:00	CY	EURO	5	772	0	17	27	2.202	2.196
2923	2020-4-22 8:00	CY	EURO	12	784	0	17	28	2.168	2.174
2924	2020-4-23 8:00	CY	EURO	6	790	0	17	29	2.152	2.153
2925	2020-4-24 8:00	CY	EURO	5	795	0	17	30	2.138	2.135
2926	2020-4-25 8:00	CY	EURO	9	804	0	17	31	2.114	2.117
2927	2020-4-26 8:00	CY	EURO	6	810	0	17	32	2.099	2.098
2928	2020-4-27 8:00	CY	EURO	7	817	0	17	33	2.081	2.204
2929	2020-4-28 8:00	CY	EURO	5	822	3	20	34	2.433	2.301
2930	2020-4-29 8:00	CY	EURO	15	837	0	20	35	2.389	2.398
2931	2020-4-30 8:00	CY	EURO	6	843	0	20	36	2.372	2.372
2932	2020-5-1 8:00	CY	EURO	7	850	0	20	37	2.353	2.353
2933	2020-5-2 8:00	CY	EURO	7	857	0	20	38	2.334	2.343
2934										
2935	2020-3-1 8:00	CZ	EURO	3	3	0	0			
2936	2020-3-2 8:00	CZ	EURO	0	3	0	0			
2937	2020-3-3 8:00	CZ	EURO	2	5	0	0			
2938	2020-3-4 8:00	CZ	EURO	0	5	0	0			
2939	2020-3-5 8:00	CZ	EURO	7	12	0	0			
2940	2020-3-6 8:00	CZ	EURO	0	12	0	0			
2941	2020-3-7 8:00	CZ	EURO	14	26	0	0			
2942	2020-3-8 8:00	CZ	EURO	6	32	0	0			
2943	2020-3-9 8:00	CZ	EURO	0	32	0	0			
2944	2020-3-10 8:00	CZ	EURO	29	61	0	0			
2945	2020-3-11 8:00	CZ	EURO	0	61	0	0			
2946	2020-3-12 8:00	CZ	EURO	55	116	0	0			
2947	2020-3-13 8:00	CZ	EURO	0	116	0	0			
2948	2020-3-14 8:00	CZ	EURO	34	150	0	0			
2949	2020-3-15 8:00	CZ	EURO	103	253	0	0			
2950	2020-3-16 8:00	CZ	EURO	45	298	0	0			
2951	2020-3-17 8:00	CZ	EURO	136	434	0	0			
2952	2020-3-18 8:00	CZ	EURO	88	522	0	0			

Table S1 Daily confirmed cases and daily deaths of COVID-19 in 214 nations from WHO

ID	Date and time	Country or region	Continent	Confirmed patients	Cumulative Confirmed patients	Daily dead patients	Daily cumulative dead patients	Lag days	Daily CFR	3DMA CFR
2953	2020-3-19 8:00	CZ	EURO	172	694	0	0			
2954	2020-3-20 8:00	CZ	EURO	210	904	0	0			
2955	2020-3-21 8:00	CZ	EURO	91	995	0	0			
2956	2020-3-22 8:00	CZ	EURO	0	995	0	0			
2957	2020-3-23 8:00	CZ	EURO	170	1165	1	1	0	0.086	0.083
2958	2020-3-24 8:00	CZ	EURO	71	1236	0	1	1	0.081	0.127
2959	2020-3-25 8:00	CZ	EURO	158	1394	2	3	2	0.215	0.220
2960	2020-3-26 8:00	CZ	EURO	260	1654	3	6	3	0.363	0.338
2961	2020-3-27 8:00	CZ	EURO	408	2062	3	9	4	0.436	0.398
2962	2020-3-28 8:00	CZ	EURO	217	2279	0	9	5	0.395	0.415
2963	2020-3-29 8:00	CZ	EURO	384	2663	2	11	6	0.413	0.458
2964	2020-3-30 8:00	CZ	EURO	166	2829	5	16	7	0.566	0.593
2965	2020-3-31 8:00	CZ	EURO	173	3002	8	24	8	0.799	0.767
2966	2020-4-1 8:00	CZ	EURO	306	3308	7	31	9	0.937	0.941
2967	2020-4-2 8:00	CZ	EURO	281	3589	8	39	10	1.087	1.055
2968	2020-4-3 8:00	CZ	EURO	269	3858	5	44	11	1.140	1.164
2969	2020-4-4 8:00	CZ	EURO	332	4190	9	53	12	1.265	1.242
2970	2020-4-5 8:00	CZ	EURO	282	4472	6	59	13	1.319	1.348
2971	2020-4-6 8:00	CZ	EURO	115	4587	8	67	14	1.461	1.466
2972	2020-4-7 8:00	CZ	EURO	235	4822	11	78	15	1.618	1.611
2973	2020-4-8 8:00	CZ	EURO	195	5017	10	88	16	1.754	1.745
2974	2020-4-9 8:00	CZ	EURO	295	5312	11	99	17	1.864	1.876
2975	2020-4-10 8:00	CZ	EURO	257	5569	13	112	18	2.011	1.984
2976	2020-4-11 8:00	CZ	EURO	163	5732	7	119	19	2.076	2.091
2977	2020-4-12 8:00	CZ	EURO	170	5902	10	129	20	2.186	2.188
2978	2020-4-13 8:00	CZ	EURO	89	5991	9	138	21	2.303	2.283
2979	2020-4-14 8:00	CZ	EURO	68	6059	5	143	22	2.360	2.428
2980	2020-4-15 8:00	CZ	EURO	82	6141	18	161	23	2.622	2.539
2981	2020-4-16 8:00	CZ	EURO	162	6303	5	166	24	2.634	2.627
2982	2020-4-17 8:00	CZ	EURO	130	6433	3	169	25	2.627	2.634
2983	2020-4-18 8:00	CZ	EURO	116	6549	4	173	26	2.642	2.663
2984	2020-4-19 8:00	CZ	EURO	105	6654	8	181	27	2.720	2.711
2985	2020-4-20 8:00	CZ	EURO	133	6787	7	188	28	2.770	2.775
2986	2020-4-21 8:00	CZ	EURO	127	6914	8	196	29	2.835	2.820
2987	2020-4-22 8:00	CZ	EURO	127	7041	5	201	30	2.855	2.877
2988	2020-4-23 8:00	CZ	EURO	95	7136	9	210	31	2.943	2.920

Table S1 Daily confirmed cases and daily deaths of COVID-19 in 214 nations from WHO

ID	Date and time	Country or region	Continent	Confirmed patients	Cumulative Confirmed patients	Daily dead patients	Daily cumulative dead patients	Lag days	Daily CFR	3DMA CFR
2989	2020-4-24 8:00	CZ	EURO	52	7188	3	213	32	2.963	2.954
2990	2020-4-25 8:00	CZ	EURO	85	7273	2	215	33	2.956	2.962
2991	2020-4-26 8:00	CZ	EURO	79	7352	3	218	34	2.965	2.969
2992	2020-4-27 8:00	CZ	EURO	52	7404	3	221	35	2.985	2.981
2993	2020-4-28 8:00	CZ	EURO	45	7449	2	223	36	2.994	3.001
2994	2020-4-29 8:00	CZ	EURO	55	7504	4	227	37	3.025	3.005
2995	2020-4-30 8:00	CZ	EURO	75	7579	0	227	38	2.995	3.031
2996	2020-5-1 8:00	CZ	EURO	103	7682	9	236	39	3.072	3.056
2997	2020-5-2 8:00	CZ	EURO	55	7737	4	240	40	3.102	3.087
2998										
2999	2020-3-11 8:00	CD	AFRO	1	1	0	0			
3000	2020-3-12 8:00	CD	AFRO	0	1	0	0			
3001	2020-3-13 8:00	CD	AFRO	0	1	0	0			
3002	2020-3-14 8:00	CD	AFRO	1	2	0	0			
3003	2020-3-15 8:00	CD	AFRO	0	2	0	0			
3004	2020-3-16 8:00	CD	AFRO	0	2	0	0			
3005	2020-3-17 8:00	CD	AFRO	1	3	0	0			
3006	2020-3-18 8:00	CD	AFRO	4	7	0	0			
3007	2020-3-19 8:00	CD	AFRO	7	14	0	0			
3008	2020-3-20 8:00	CD	AFRO	0	14	0	0			
3009	2020-3-21 8:00	CD	AFRO	9	23	1	1	0	4.348	4.348
3010	2020-3-22 8:00	CD	AFRO	0	23	0	1	1	4.348	5.121
3011	2020-3-23 8:00	CD	AFRO	7	30	1	2	2	6.667	5.523
3012	2020-3-24 8:00	CD	AFRO	6	36	0	2	3	5.556	5.556
3013	2020-3-25 8:00	CD	AFRO	9	45	0	2	4	4.444	5.294
3014	2020-3-26 8:00	CD	AFRO	6	51	1	3	5	5.882	5.911
3015	2020-3-27 8:00	CD	AFRO	3	54	1	4	6	7.407	7.303
3016	2020-3-28 8:00	CD	AFRO	4	58	1	5	7	8.621	8.284
3017	2020-3-29 8:00	CD	AFRO	10	68	1	6	8	8.824	9.107
3018	2020-3-30 8:00	CD	AFRO	13	81	2	8	9	9.877	8.954
3019	2020-3-31 8:00	CD	AFRO	17	98	0	8	10	8.163	8.766
3020	2020-4-1 8:00	CD	AFRO	11	109	1	9	11	8.257	8.454
3021	2020-4-2 8:00	CD	AFRO	14	123	2	11	12	8.943	8.967
3022	2020-4-3 8:00	CD	AFRO	11	134	2	13	13	9.701	9.818
3023	2020-4-4 8:00	CD	AFRO	14	148	3	16	14	10.811	10.441
3024	2020-4-5 8:00	CD	AFRO	0	148	0	16	15	10.811	10.934

Table S1 Daily confirmed cases and daily deaths of COVID-19 in 214 nations from WHO

ID	Date and time	Country or region	Continent	Confirmed patients	Cumulative Confirmed patients	Daily dead patients	Daily cumulative dead patients	Lag days	Daily CFR	3DMA CFR
3025	2020-4-6 8:00	CD	AFRO	13	161	2	18	16	11.180	11.057
3026	2020-4-7 8:00	CD	AFRO	0	161	0	18	17	11.180	11.096
3027	2020-4-8 8:00	CD	AFRO	22	183	2	20	18	10.929	10.590
3028	2020-4-9 8:00	CD	AFRO	24	207	0	20	19	9.662	9.964
3029	2020-4-10 8:00	CD	AFRO	8	215	0	20	20	9.302	9.422
3030	2020-4-11 8:00	CD	AFRO	0	215	0	20	21	9.302	9.191
3031	2020-4-12 8:00	CD	AFRO	8	223	0	20	22	8.969	8.927
3032	2020-4-13 8:00	CD	AFRO	12	235	0	20	23	8.511	8.593
3033	2020-4-14 8:00	CD	AFRO	6	241	0	20	24	8.299	8.359
3034	2020-4-15 8:00	CD	AFRO	13	254	1	21	25	8.268	8.269
3035	2020-4-16 8:00	CD	AFRO	13	267	1	22	26	8.240	8.174
3036	2020-4-17 8:00	CD	AFRO	20	287	1	23	27	8.014	8.089
3037	2020-4-18 8:00	CD	AFRO	0	287	0	23	28	8.014	7.891
3038	2020-4-19 8:00	CD	AFRO	40	327	2	25	29	7.645	7.730
3039	2020-4-20 8:00	CD	AFRO	5	332	0	25	30	7.530	7.439
3040	2020-4-21 8:00	CD	AFRO	18	350	0	25	31	7.143	7.212
3041	2020-4-22 8:00	CD	AFRO	9	359	0	25	32	6.964	7.023
3042	2020-4-23 8:00	CD	AFRO	0	359	0	25	33	6.964	6.758
3043	2020-4-24 8:00	CD	AFRO	35	394	0	25	34	6.345	6.680
3044	2020-4-25 8:00	CD	AFRO	22	416	3	28	35	6.731	6.470
3045	2020-4-26 8:00	CD	AFRO	26	442	0	28	36	6.335	6.389
3046	2020-4-27 8:00	CD	AFRO	17	459	0	28	37	6.100	6.268
3047	2020-4-28 8:00	CD	AFRO	12	471	2	30	38	6.369	6.193
3048	2020-4-29 8:00	CD	AFRO	20	491	0	30	39	6.110	6.226
3049	2020-4-30 8:00	CD	AFRO	9	500	1	31	40	6.200	5.910
3050	2020-5-1 8:00	CD	AFRO	72	572	0	31	41	5.420	5.639
3051	2020-5-2 8:00	CD	AFRO	32	604	1	32	42	5.298	5.359
3052										
3053	2020-2-27 8:00	DK	EURO	1	1	0	0			
3054	2020-2-28 8:00	DK	EURO	1	2	0	0			
3055	2020-2-29 8:00	DK	EURO	1	3	0	0			
3056	2020-3-1 8:00	DK	EURO	1	4	0	0			
3057	2020-3-2 8:00	DK	EURO	1	5	0	0			
3058	2020-3-3 8:00	DK	EURO	3	8	0	0			
3059	2020-3-4 8:00	DK	EURO	2	10	0	0			
3060	2020-3-5 8:00	DK	EURO	8	18	0	0			

Table S1 Daily confirmed cases and daily deaths of COVID-19 in 214 nations from WHO

ID	Date and time	Country or region	Continent	Confirmed patients	Cumulative Confirmed patients	Daily dead patients	Daily cumulative dead patients	Lag days	Daily CFR	3DMA CFR
3061	2020-3-6 8:00	DK	EURO	5	23	0	0			
3062	2020-3-7 8:00	DK	EURO	8	31	0	0			
3063	2020-3-8 8:00	DK	EURO	5	36	0	0			
3064	2020-3-9 8:00	DK	EURO	54	90	0	0			
3065	2020-3-10 8:00	DK	EURO	172	262	0	0			
3066	2020-3-11 8:00	DK	EURO	276	538	0	0			
3067	2020-3-12 8:00	DK	EURO	136	674	0	0			
3068	2020-3-13 8:00	DK	EURO	127	801	0	0			
3069	2020-3-14 8:00	DK	EURO	26	827	0	0			
3070	2020-3-15 8:00	DK	EURO	37	864	1	1	0	0.116	0.114
3071	2020-3-16 8:00	DK	EURO	34	898	0	1	1	0.111	0.212
3072	2020-3-17 8:00	DK	EURO	79	977	3	4	2	0.409	0.301
3073	2020-3-18 8:00	DK	EURO	67	1044	0	4	3	0.383	0.441
3074	2020-3-19 8:00	DK	EURO	88	1132	2	6	4	0.530	0.543
3075	2020-3-20 8:00	DK	EURO	123	1255	3	9	5	0.717	0.743
3076	2020-3-21 8:00	DK	EURO	71	1326	4	13	6	0.980	0.893
3077	2020-3-22 8:00	DK	EURO	0	1326	0	13	7	0.980	0.964
3078	2020-3-23 8:00	DK	EURO	69	1395	0	13	8	0.932	1.185
3079	2020-3-24 8:00	DK	EURO	65	1460	11	24	9	1.644	1.529
3080	2020-3-25 8:00	DK	EURO	131	1591	8	32	10	2.011	1.876
3081	2020-3-26 8:00	DK	EURO	133	1724	2	34	11	1.972	2.056
3082	2020-3-27 8:00	DK	EURO	153	1877	7	41	12	2.184	2.233
3083	2020-3-28 8:00	DK	EURO	169	2046	11	52	13	2.542	2.560
3084	2020-3-29 8:00	DK	EURO	155	2201	13	65	14	2.953	2.834
3085	2020-3-30 8:00	DK	EURO	194	2395	7	72	15	3.006	2.982
3086	2020-3-31 8:00	DK	EURO	182	2577	5	77	16	2.988	3.047
3087	2020-4-1 8:00	DK	EURO	283	2860	13	90	17	3.147	3.161
3088	2020-4-2 8:00	DK	EURO	247	3107	14	104	18	3.347	3.376
3089	2020-4-3 8:00	DK	EURO	279	3386	19	123	19	3.633	3.560
3090	2020-4-4 8:00	DK	EURO	371	3757	16	139	20	3.700	3.760
3091	2020-4-5 8:00	DK	EURO	320	4077	22	161	21	3.949	3.915
3092	2020-4-6 8:00	DK	EURO	292	4369	18	179	22	4.097	4.014
3093	2020-4-7 8:00	DK	EURO	312	4681	8	187	23	3.995	4.032
3094	2020-4-8 8:00	DK	EURO	390	5071	16	203	24	4.003	4.011
3095	2020-4-9 8:00	DK	EURO	331	5402	15	218	25	4.036	4.082
3096	2020-4-10 8:00	DK	EURO	233	5635	19	237	26	4.206	4.162

Table S1 Daily confirmed cases and daily deaths of COVID-19 in 214 nations from WHO

ID	Date and time	Country or region	Continent	Confirmed patients	Cumulative Confirmed patients	Daily dead patients	Daily cumulative dead patients	Lag days	Daily CFR	3DMA CFR
3097	2020-4-11 8:00	DK	EURO	184	5819	10	247	27	4.245	4.262
3098	2020-4-12 8:00	DK	EURO	177	5996	13	260	28	4.336	4.334
3099	2020-4-13 8:00	DK	EURO	178	6174	13	273	29	4.422	4.423
3100	2020-4-14 8:00	DK	EURO	144	6318	12	285	30	4.511	4.508
3101	2020-4-15 8:00	DK	EURO	193	6511	14	299	31	4.592	4.576
3102	2020-4-16 8:00	DK	EURO	170	6681	10	309	32	4.625	4.628
3103	2020-4-17 8:00	DK	EURO	198	6879	12	321	33	4.666	4.681
3104	2020-4-18 8:00	DK	EURO	194	7073	15	336	34	4.750	4.732
3105	2020-4-19 8:00	DK	EURO	169	7242	10	346	35	4.778	4.779
3106	2020-4-20 8:00	DK	EURO	142	7384	9	355	36	4.808	4.810
3107	2020-4-21 8:00	DK	EURO	131	7515	9	364	37	4.844	4.820
3108	2020-4-22 8:00	DK	EURO	180	7695	6	370	38	4.808	4.835
3109	2020-4-23 8:00	DK	EURO	217	7912	14	384	39	4.853	4.847
3110	2020-4-24 8:00	DK	EURO	161	8073	10	394	40	4.880	4.881
3111	2020-4-25 8:00	DK	EURO	137	8210	9	403	41	4.909	4.913
3112	2020-4-26 8:00	DK	EURO	235	8445	15	418	42	4.950	4.927
3113	2020-4-27 8:00	DK	EURO	130	8575	4	422	43	4.921	4.927
3114	2020-4-28 8:00	DK	EURO	123	8698	5	427	44	4.909	4.911
3115	2020-4-29 8:00	DK	EURO	153	8851	7	434	45	4.903	4.910
3116	2020-4-30 8:00	DK	EURO	157	9008	9	443	46	4.918	4.919
3117	2020-5-1 8:00	DK	EURO	150	9158	9	452	47	4.936	4.931
3118	2020-5-2 8:00	DK	EURO	153	9311	8	460	48	4.940	4.938
3119										
3120	2020-3-18 8:00	DJ	EMRO	1	1	0	0			
3121	2020-3-19 8:00	DJ	EMRO	0	1	0	0			
3122	2020-3-20 8:00	DJ	EMRO	0	1	0	0			
3123	2020-3-21 8:00	DJ	EMRO	0	1	0	0			
3124	2020-3-22 8:00	DJ	EMRO	0	1	0	0			
3125	2020-3-23 8:00	DJ	EMRO	2	3	0	0			
3126	2020-3-24 8:00	DJ	EMRO	0	3	0	0			
3127	2020-3-25 8:00	DJ	EMRO	9	12	0	0			
3128	2020-3-26 8:00	DJ	EMRO	0	12	0	0			
3129	2020-3-27 8:00	DJ	EMRO	1	13	0	0			
3130	2020-3-28 8:00	DJ	EMRO	2	15	0	0			
3131	2020-3-29 8:00	DJ	EMRO	4	19	0	0			
3132	2020-3-30 8:00	DJ	EMRO	7	26	0	0			

Table S1 Daily confirmed cases and daily deaths of COVID-19 in 214 nations from WHO

ID	Date and time	Country or region	Continent	Confirmed patients	Cumulative Confirmed patients	Daily dead patients	Daily cumulative dead patients	Lag days	Daily CFR	3DMA CFR
3133	2020-3-31 8:00	DJ	EMRO	5	31	0	0			
3134	2020-4-1 8:00	DJ	EMRO	3	34	0	0			
3135	2020-4-2 8:00	DJ	EMRO	7	41	0	0			
3136	2020-4-3 8:00	DJ	EMRO	9	50	0	0			
3137	2020-4-4 8:00	DJ	EMRO	1	51	0	0			
3138	2020-4-5 8:00	DJ	EMRO	8	59	0	0			
3139	2020-4-6 8:00	DJ	EMRO	31	90	0	0			
3140	2020-4-7 8:00	DJ	EMRO	31	121	0	0			
3141	2020-4-8 8:00	DJ	EMRO	14	135	0	0			
3142	2020-4-9 8:00	DJ	EMRO	5	140	1	1	0	0.714	0.690
3143	2020-4-10 8:00	DJ	EMRO	10	150	0	1	1	0.667	0.817
3144	2020-4-11 8:00	DJ	EMRO	37	187	1	2	2	1.070	0.889
3145	2020-4-12 8:00	DJ	EMRO	28	215	0	2	3	0.930	0.890
3146	2020-4-13 8:00	DJ	EMRO	83	298	0	2	4	0.671	0.717
3147	2020-4-14 8:00	DJ	EMRO	65	363	0	2	5	0.551	0.561
3148	2020-4-15 8:00	DJ	EMRO	72	435	0	2	6	0.460	0.450
3149	2020-4-16 8:00	DJ	EMRO	156	591	0	2	7	0.338	0.357
3150	2020-4-17 8:00	DJ	EMRO	141	732	0	2	8	0.273	0.295
3151	2020-4-18 8:00	DJ	EMRO	0	732	0	2	9	0.273	0.273
3152	2020-4-19 8:00	DJ	EMRO	0	732	0	2	10	0.273	0.261
3153	2020-4-20 8:00	DJ	EMRO	114	846	0	2	11	0.236	0.240
3154	2020-4-21 8:00	DJ	EMRO	99	945	0	2	12	0.212	0.218
3155	2020-4-22 8:00	DJ	EMRO	29	974	0	2	13	0.205	0.207
3156	2020-4-23 8:00	DJ	EMRO	12	986	0	2	14	0.203	0.203
3157	2020-4-24 8:00	DJ	EMRO	13	999	0	2	15	0.200	0.200
3158	2020-4-25 8:00	DJ	EMRO	9	1008	0	2	16	0.198	0.198
3159	2020-4-26 8:00	DJ	EMRO	15	1023	0	2	17	0.196	0.196
3160	2020-4-27 8:00	DJ	EMRO	12	1035	0	2	18	0.193	0.192
3161	2020-4-28 8:00	DJ	EMRO	37	1072	0	2	19	0.187	0.189
3162	2020-4-29 8:00	DJ	EMRO	5	1077	0	2	20	0.186	0.185
3163	2020-4-30 8:00	DJ	EMRO	12	1089	0	2	21	0.184	0.184
3164	2020-5-1 8:00	DJ	EMRO	8	1097	0	2	22	0.182	0.183
3165	2020-5-2 8:00	DJ	EMRO	0	1097	0	2	23	0.182	0.182
3166										
3167	2020-3-24 8:00	DM	AMRO	2	2	0	0			
3168	2020-3-25 8:00	DM	AMRO	0	2	0	0			

Table S1 Daily confirmed cases and daily deaths of COVID-19 in 214 nations from WHO

ID	Date and time	Country or region	Continent	Confirmed patients	Cumulative Confirmed patients	Daily dead patients	Daily cumulative dead patients	Lag days	Daily CFR	3DMA CFR
3169	2020-3-26 8:00	DM	AMRO	9	11	0	0			
3170	2020-3-27 8:00	DM	AMRO	0	11	0	0			
3171	2020-3-28 8:00	DM	AMRO	0	11	0	0			
3172	2020-3-29 8:00	DM	AMRO	0	11	0	0			
3173	2020-3-30 8:00	DM	AMRO	0	11	0	0			
3174	2020-3-31 8:00	DM	AMRO	0	11	0	0			
3175	2020-4-1 8:00	DM	AMRO	0	11	0	0			
3176	2020-4-2 8:00	DM	AMRO	0	11	0	0			
3177	2020-4-3 8:00	DM	AMRO	0	11	0	0			
3178	2020-4-4 8:00	DM	AMRO	0	11	0	0			
3179	2020-4-5 8:00	DM	AMRO	0	11	0	0			
3180	2020-4-6 8:00	DM	AMRO	0	11	0	0			
3181	2020-4-7 8:00	DM	AMRO	3	14	0	0			
3182	2020-4-8 8:00	DM	AMRO	1	15	0	0			
3183	2020-4-9 8:00	DM	AMRO	0	15	0	0			
3184	2020-4-10 8:00	DM	AMRO	0	15	0	0			
3185	2020-4-11 8:00	DM	AMRO	1	16	0	0			
3186	2020-4-12 8:00	DM	AMRO	0	16	0	0			
3187	2020-4-13 8:00	DM	AMRO	0	16	0	0			
3188	2020-4-14 8:00	DM	AMRO	0	16	0	0			
3189	2020-4-15 8:00	DM	AMRO	0	16	0	0			
3190	2020-4-16 8:00	DM	AMRO	0	16	0	0			
3191	2020-4-17 8:00	DM	AMRO	0	16	0	0			
3192	2020-4-18 8:00	DM	AMRO	0	16	0	0			
3193	2020-4-19 8:00	DM	AMRO	0	16	0	0			
3194	2020-4-20 8:00	DM	AMRO	0	16	0	0			
3195	2020-4-21 8:00	DM	AMRO	0	16	0	0			
3196	2020-4-22 8:00	DM	AMRO	0	16	0	0			
3197	2020-4-23 8:00	DM	AMRO	0	16	0	0			
3198	2020-4-24 8:00	DM	AMRO	0	16	0	0			
3199	2020-4-25 8:00	DM	AMRO	0	16	0	0			
3200	2020-4-26 8:00	DM	AMRO	0	16	0	0			
3201	2020-4-27 8:00	DM	AMRO	0	16	0	0			
3202	2020-4-28 8:00	DM	AMRO	0	16	0	0			
3203	2020-4-29 8:00	DM	AMRO	0	16	0	0			
3204	2020-4-30 8:00	DM	AMRO	0	16	0	0			

Table S1 Daily confirmed cases and daily deaths of COVID-19 in 214 nations from WHO

ID	Date and time	Country or region	Continent	Confirmed patients	Cumulative Confirmed patients	Daily dead patients	Daily cumulative dead patients	Lag days	Daily CFR	3DMA CFR
3205	2020-5-1 8:00	DM	AMRO	0	16	0	0			
3206	2020-5-2 8:00	DM	AMRO	0	16	0	0			
3207										
3208	2020-3-2 8:00	DO	AMRO	1	1	0	0			
3209	2020-3-3 8:00	DO	AMRO	0	1	0	0			
3210	2020-3-4 8:00	DO	AMRO	0	1	0	0			
3211	2020-3-5 8:00	DO	AMRO	0	1	0	0			
3212	2020-3-6 8:00	DO	AMRO	0	1	0	0			
3213	2020-3-7 8:00	DO	AMRO	0	1	0	0			
3214	2020-3-8 8:00	DO	AMRO	0	1	0	0			
3215	2020-3-9 8:00	DO	AMRO	0	1	0	0			
3216	2020-3-10 8:00	DO	AMRO	4	5	0	0			
3217	2020-3-11 8:00	DO	AMRO	0	5	0	0			
3218	2020-3-12 8:00	DO	AMRO	0	5	0	0			
3219	2020-3-13 8:00	DO	AMRO	0	5	0	0			
3220	2020-3-14 8:00	DO	AMRO	0	5	0	0			
3221	2020-3-15 8:00	DO	AMRO	0	5	0	0			
3222	2020-3-16 8:00	DO	AMRO	0	5	0	0			
3223	2020-3-17 8:00	DO	AMRO	16	21	1	1	0	4.762	4.762
3224	2020-3-18 8:00	DO	AMRO	0	21	0	1	1	4.762	5.135
3225	2020-3-19 8:00	DO	AMRO	13	34	1	2	2	5.882	4.474
3226	2020-3-20 8:00	DO	AMRO	38	72	0	2	3	2.778	3.813
3227	2020-3-21 8:00	DO	AMRO	0	72	0	2	4	2.778	2.778
3228	2020-3-22 8:00	DO	AMRO	0	72	0	2	5	2.778	2.778
3229	2020-3-23 8:00	DO	AMRO	0	72	0	2	6	2.778	2.493
3230	2020-3-24 8:00	DO	AMRO	240	312	4	6	7	1.923	2.208
3231	2020-3-25 8:00	DO	AMRO	0	312	0	6	8	1.923	1.965
3232	2020-3-26 8:00	DO	AMRO	176	488	4	10	9	2.049	2.007
3233	2020-3-27 8:00	DO	AMRO	0	488	0	10	10	2.049	2.514
3234	2020-3-28 8:00	DO	AMRO	93	581	10	20	11	3.442	2.978
3235	2020-3-29 8:00	DO	AMRO	0	581	0	20	12	3.442	3.442
3236	2020-3-30 8:00	DO	AMRO	0	581	0	20	13	3.442	3.849
3237	2020-3-31 8:00	DO	AMRO	320	901	22	42	14	4.661	4.234
3238	2020-4-1 8:00	DO	AMRO	208	1109	9	51	15	4.599	4.566
3239	2020-4-2 8:00	DO	AMRO	175	1284	6	57	16	4.439	4.462
3240	2020-4-3 8:00	DO	AMRO	96	1380	3	60	17	4.348	4.452

Table S1 Daily confirmed cases and daily deaths of COVID-19 in 214 nations from WHO

ID	Date and time	Country or region	Continent	Confirmed patients	Cumulative Confirmed patients	Daily dead patients	Daily cumulative dead patients	Lag days	Daily CFR	3DMA CFR
3241	2020-4-4 8:00	DO	AMRO	108	1488	8	68	18	4.570	4.496
3242	2020-4-5 8:00	DO	AMRO	0	1488	0	68	19	4.570	4.570
3243	2020-4-6 8:00	DO	AMRO	0	1488	0	68	20	4.570	4.615
3244	2020-4-7 8:00	DO	AMRO	340	1828	18	86	21	4.705	4.762
3245	2020-4-8 8:00	DO	AMRO	128	1956	12	98	22	5.010	4.908
3246	2020-4-9 8:00	DO	AMRO	0	1956	0	98	23	5.010	5.015
3247	2020-4-10 8:00	DO	AMRO	393	2349	20	118	24	5.023	5.019
3248	2020-4-11 8:00	DO	AMRO	0	2349	0	118	25	5.023	4.952
3249	2020-4-12 8:00	DO	AMRO	271	2620	8	126	26	4.809	5.221
3250	2020-4-13 8:00	DO	AMRO	347	2967	47	173	27	5.831	5.410
3251	2020-4-14 8:00	DO	AMRO	200	3167	4	177	28	5.589	5.663
3252	2020-4-15 8:00	DO	AMRO	119	3286	6	183	29	5.569	5.463
3253	2020-4-16 8:00	DO	AMRO	328	3614	6	189	30	5.230	5.339
3254	2020-4-17 8:00	DO	AMRO	141	3755	7	196	31	5.220	5.099
3255	2020-4-18 8:00	DO	AMRO	371	4126	4	200	32	4.847	5.024
3256	2020-4-19 8:00	DO	AMRO	209	4335	17	217	33	5.006	4.894
3257	2020-4-20 8:00	DO	AMRO	345	4680	9	226	34	4.829	4.856
3258	2020-4-21 8:00	DO	AMRO	284	4964	9	235	35	4.734	4.807
3259	2020-4-22 8:00	DO	AMRO	80	5044	10	245	36	4.857	4.832
3260	2020-4-23 8:00	DO	AMRO	256	5300	15	260	37	4.906	4.848
3261	2020-4-24 8:00	DO	AMRO	243	5543	5	265	38	4.781	4.777
3262	2020-4-25 8:00	DO	AMRO	206	5749	2	267	39	4.644	4.677
3263	2020-4-26 8:00	DO	AMRO	177	5926	6	273	40	4.607	4.594
3264	2020-4-27 8:00	DO	AMRO	209	6135	5	278	41	4.531	4.540
3265	2020-4-28 8:00	DO	AMRO	158	6293	4	282	42	4.481	4.490
3266	2020-4-29 8:00	DO	AMRO	123	6416	4	286	43	4.458	4.448
3267	2020-4-30 8:00	DO	AMRO	236	6652	7	293	44	4.405	4.393
3268	2020-5-1 8:00	DO	AMRO	320	6972	8	301	45	4.317	4.339
3269	2020-5-2 8:00	DO	AMRO	316	7288	12	313	46	4.295	4.306
3270										
3271	2020-2-29 8:00	EC	AMRO	1	1	0	0			
3272	2020-3-1 8:00	EC	AMRO	0	1	0	0			
3273	2020-3-2 8:00	EC	AMRO	5	6	0	0			
3274	2020-3-3 8:00	EC	AMRO	1	7	0	0			
3275	2020-3-4 8:00	EC	AMRO	0	7	0	0			
3276	2020-3-5 8:00	EC	AMRO	6	13	0	0			

Table S1 Daily confirmed cases and daily deaths of COVID-19 in 214 nations from WHO

ID	Date and time	Country or region	Continent	Confirmed patients	Cumulative Confirmed patients	Daily dead patients	Daily cumulative dead patients	Lag days	Daily CFR	3DMA CFR
3277	2020-3-6 8:00	EC	AMRO	0	13	0	0			
3278	2020-3-7 8:00	EC	AMRO	1	14	0	0			
3279	2020-3-8 8:00	EC	AMRO	0	14	0	0			
3280	2020-3-9 8:00	EC	AMRO	1	15	0	0			
3281	2020-3-10 8:00	EC	AMRO	0	15	0	0			
3282	2020-3-11 8:00	EC	AMRO	0	15	0	0			
3283	2020-3-12 8:00	EC	AMRO	2	17	0	0			
3284	2020-3-13 8:00	EC	AMRO	6	23	0	0			
3285	2020-3-14 8:00	EC	AMRO	0	23	0	0			
3286	2020-3-15 8:00	EC	AMRO	0	23	0	0			
3287	2020-3-16 8:00	EC	AMRO	14	37	2	2	0	5.405	4.427
3288	2020-3-17 8:00	EC	AMRO	21	58	0	2	1	3.448	3.381
3289	2020-3-18 8:00	EC	AMRO	97	155	0	2	2	1.290	2.082
3290	2020-3-19 8:00	EC	AMRO	44	199	1	3	3	1.508	1.387
3291	2020-3-20 8:00	EC	AMRO	168	367	2	5	4	1.362	1.418
3292	2020-3-21 8:00	EC	AMRO	139	506	2	7	5	1.383	1.376
3293	2020-3-22 8:00	EC	AMRO	0	506	0	7	6	1.383	1.361
3294	2020-3-23 8:00	EC	AMRO	26	532	0	7	7	1.316	1.154
3295	2020-3-24 8:00	EC	AMRO	517	1049	1	8	8	0.763	0.947
3296	2020-3-25 8:00	EC	AMRO	0	1049	0	8	9	0.763	0.729
3297	2020-3-26 8:00	EC	AMRO	162	1211	0	8	10	0.661	0.695
3298	2020-3-27 8:00	EC	AMRO	0	1211	0	8	11	0.661	0.608
3299	2020-3-28 8:00	EC	AMRO	384	1595	0	8	12	0.502	0.534
3300	2020-3-29 8:00	EC	AMRO	228	1823	0	8	13	0.439	0.459
3301	2020-3-30 8:00	EC	AMRO	12	1835	0	8	14	0.436	0.428
3302	2020-3-31 8:00	EC	AMRO	127	1962	0	8	15	0.408	0.400
3303	2020-4-1 8:00	EC	AMRO	278	2240	0	8	16	0.357	1.365
3304	2020-4-2 8:00	EC	AMRO	132	2372	71	79	17	3.331	2.494
3305	2020-4-3 8:00	EC	AMRO	791	3163	41	120	18	3.794	3.639
3306	2020-4-4 8:00	EC	AMRO	0	3163	0	120	19	3.794	4.184
3307	2020-4-5 8:00	EC	AMRO	302	3465	52	172	20	4.964	4.574
3308	2020-4-6 8:00	EC	AMRO	0	3465	0	172	21	4.964	5.008
3309	2020-4-7 8:00	EC	AMRO	282	3747	19	191	22	5.097	5.053
3310	2020-4-8 8:00	EC	AMRO	0	3747	0	191	23	5.097	5.211
3311	2020-4-9 8:00	EC	AMRO	703	4450	51	242	24	5.438	5.338
3312	2020-4-10 8:00	EC	AMRO	515	4965	30	272	25	5.478	5.021

Table S1 Daily confirmed cases and daily deaths of COVID-19 in 214 nations from WHO

ID	Date and time	Country or region	Continent	Confirmed patients	Cumulative Confirmed patients	Daily dead patients	Daily cumulative dead patients	Lag days	Daily CFR	3DMA CFR
3313	2020-4-11 8:00	EC	AMRO	2196	7161	25	297	26	4.147	4.655
3314	2020-4-12 8:00	EC	AMRO	96	7257	18	315	27	4.341	4.316
3315	2020-4-13 8:00	EC	AMRO	209	7466	18	333	28	4.460	4.505
3316	2020-4-14 8:00	EC	AMRO	63	7529	22	355	29	4.715	4.676
3317	2020-4-15 8:00	EC	AMRO	74	7603	14	369	30	4.853	4.835
3318	2020-4-16 8:00	EC	AMRO	255	7858	19	388	31	4.938	4.897
3319	2020-4-17 8:00	EC	AMRO	367	8225	15	403	32	4.900	4.940
3320	2020-4-18 8:00	EC	AMRO	225	8450	18	421	33	4.982	4.979
3321	2020-4-19 8:00	EC	AMRO	572	9022	35	456	34	5.054	5.014
3322	2020-4-20 8:00	EC	AMRO	446	9468	18	474	35	5.006	5.022
3323	2020-4-21 8:00	EC	AMRO	660	10128	33	507	36	5.006	5.004
3324	2020-4-22 8:00	EC	AMRO	270	10398	13	520	37	5.001	4.985
3325	2020-4-23 8:00	EC	AMRO	452	10850	17	537	38	4.949	4.986
3326	2020-4-24 8:00	EC	AMRO	333	11183	23	560	39	5.008	4.164
3327	2020-4-25 8:00	EC	AMRO	11536	22719	16	576	40	2.535	3.359
3328	2020-4-26 8:00	EC	AMRO	0	22719	0	576	41	2.535	2.535
3329	2020-4-27 8:00	EC	AMRO	0	22719	0	576	42	2.535	2.641
3330	2020-4-28 8:00	EC	AMRO	521	23240	87	663	43	2.853	2.993
3331	2020-4-29 8:00	EC	AMRO	1018	24258	208	871	44	3.591	3.341
3332	2020-4-30 8:00	EC	AMRO	417	24675	12	883	45	3.579	3.593
3333	2020-5-1 8:00	EC	AMRO	259	24934	17	900	46	3.610	3.741
3334	2020-5-2 8:00	EC	AMRO	1402	26336	163	1063	47	4.036	3.823
3335										
3336	2020-2-14 8:00	EG	EMRO	1	1	0	0			
3337	2020-2-15 8:00	EG	EMRO	0	1	0	0			
3338	2020-2-16 8:00	EG	EMRO	0	1	0	0			
3339	2020-2-17 8:00	EG	EMRO	0	1	0	0			
3340	2020-2-18 8:00	EG	EMRO	0	1	0	0			
3341	2020-2-19 8:00	EG	EMRO	0	1	0	0			
3342	2020-2-20 8:00	EG	EMRO	0	1	0	0			
3343	2020-2-21 8:00	EG	EMRO	0	1	0	0			
3344	2020-2-22 8:00	EG	EMRO	0	1	0	0			
3345	2020-2-23 8:00	EG	EMRO	0	1	0	0			
3346	2020-2-24 8:00	EG	EMRO	0	1	0	0			
3347	2020-2-25 8:00	EG	EMRO	0	1	0	0			
3348	2020-2-26 8:00	EG	EMRO	0	1	0	0			

Table S1 Daily confirmed cases and daily deaths of COVID-19 in 214 nations from WHO

ID	Date and time	Country or region	Continent	Confirmed patients	Cumulative Confirmed patients	Daily dead patients	Daily cumulative dead patients	Lag days	Daily CFR	3DMA CFR
3349	2020-2-27 8:00	EG	EMRO	0	1	0	0			
3350	2020-2-28 8:00	EG	EMRO	0	1	0	0			
3351	2020-2-29 8:00	EG	EMRO	0	1	0	0			
3352	2020-3-1 8:00	EG	EMRO	0	1	0	0			
3353	2020-3-2 8:00	EG	EMRO	1	2	0	0			
3354	2020-3-3 8:00	EG	EMRO	0	2	0	0			
3355	2020-3-4 8:00	EG	EMRO	0	2	0	0			
3356	2020-3-5 8:00	EG	EMRO	1	3	0	0			
3357	2020-3-6 8:00	EG	EMRO	0	3	0	0			
3358	2020-3-7 8:00	EG	EMRO	45	48	0	0			
3359	2020-3-8 8:00	EG	EMRO	1	49	1	1	0	2.041	1.868
3360	2020-3-9 8:00	EG	EMRO	10	59	0	1	1	1.695	1.810
3361	2020-3-10 8:00	EG	EMRO	0	59	0	1	2	1.695	1.627
3362	2020-3-11 8:00	EG	EMRO	8	67	0	1	3	1.493	1.560
3363	2020-3-12 8:00	EG	EMRO	0	67	0	1	4	1.493	1.493
3364	2020-3-13 8:00	EG	EMRO	0	67	0	1	5	1.493	1.712
3365	2020-3-14 8:00	EG	EMRO	26	93	1	2	6	2.151	1.820
3366	2020-3-15 8:00	EG	EMRO	17	110	0	2	7	1.818	1.852
3367	2020-3-16 8:00	EG	EMRO	16	126	0	2	8	1.587	1.938
3368	2020-3-17 8:00	EG	EMRO	40	166	2	4	9	2.410	2.353
3369	2020-3-18 8:00	EG	EMRO	30	196	2	6	10	3.061	2.776
3370	2020-3-19 8:00	EG	EMRO	14	210	0	6	11	2.857	2.884
3371	2020-3-20 8:00	EG	EMRO	46	256	1	7	12	2.734	2.800
3372	2020-3-21 8:00	EG	EMRO	29	285	1	8	13	2.807	2.981
3373	2020-3-22 8:00	EG	EMRO	9	294	2	10	14	3.401	3.497
3374	2020-3-23 8:00	EG	EMRO	33	327	4	14	15	4.281	4.291
3375	2020-3-24 8:00	EG	EMRO	39	366	5	19	16	5.191	4.741
3376	2020-3-25 8:00	EG	EMRO	76	442	2	21	17	4.751	4.849
3377	2020-3-26 8:00	EG	EMRO	14	456	0	21	18	4.605	4.735
3378	2020-3-27 8:00	EG	EMRO	39	495	3	24	19	4.848	5.017
3379	2020-3-28 8:00	EG	EMRO	41	536	6	30	20	5.597	5.565
3380	2020-3-29 8:00	EG	EMRO	40	576	6	36	21	6.250	6.138
3381	2020-3-30 8:00	EG	EMRO	33	609	4	40	22	6.568	6.356
3382	2020-3-31 8:00	EG	EMRO	47	656	1	41	23	6.250	6.432
3383	2020-4-1 8:00	EG	EMRO	54	710	5	46	24	6.479	6.468
3384	2020-4-2 8:00	EG	EMRO	69	779	6	52	25	6.675	6.620

Table S1 Daily confirmed cases and daily deaths of COVID-19 in 214 nations from WHO

ID	Date and time	Country or region	Continent	Confirmed patients	Cumulative Confirmed patients	Daily dead patients	Daily cumulative dead patients	Lag days	Daily CFR	3DMA CFR
3385	2020-4-3 8:00	EG	EMRO	86	865	6	58	26	6.705	6.694
3386	2020-4-4 8:00	EG	EMRO	120	985	8	66	27	6.701	6.680
3387	2020-4-5 8:00	EG	EMRO	85	1070	5	71	28	6.636	6.662
3388	2020-4-6 8:00	EG	EMRO	103	1173	7	78	29	6.650	6.572
3389	2020-4-7 8:00	EG	EMRO	149	1322	7	85	30	6.430	6.521
3390	2020-4-8 8:00	EG	EMRO	128	1450	9	94	31	6.483	6.505
3391	2020-4-9 8:00	EG	EMRO	110	1560	9	103	32	6.603	6.677
3392	2020-4-10 8:00	EG	EMRO	139	1699	15	118	33	6.945	7.024
3393	2020-4-11 8:00	EG	EMRO	95	1794	17	135	34	7.525	7.333
3394	2020-4-12 8:00	EG	EMRO	145	1939	11	146	35	7.530	7.585
3395	2020-4-13 8:00	EG	EMRO	126	2065	13	159	36	7.700	7.573
3396	2020-4-14 8:00	EG	EMRO	125	2190	5	164	37	7.489	7.588
3397	2020-4-15 8:00	EG	EMRO	160	2350	14	178	38	7.574	7.456
3398	2020-4-16 8:00	EG	EMRO	155	2505	5	183	39	7.305	7.404
3399	2020-4-17 8:00	EG	EMRO	168	2673	13	196	40	7.333	7.282
3400	2020-4-18 8:00	EG	EMRO	171	2844	9	205	41	7.208	7.310
3401	2020-4-19 8:00	EG	EMRO	188	3032	19	224	42	7.388	7.399
3402	2020-4-20 8:00	EG	EMRO	112	3144	15	239	43	7.602	7.497
3403	2020-4-21 8:00	EG	EMRO	189	3333	11	250	44	7.501	7.556
3404	2020-4-22 8:00	EG	EMRO	157	3490	14	264	45	7.564	7.536
3405	2020-4-23 8:00	EG	EMRO	169	3659	12	276	46	7.543	7.431
3406	2020-4-24 8:00	EG	EMRO	433	4092	18	294	47	7.185	7.304
3407	2020-4-25 8:00	EG	EMRO	0	4092	0	294	48	7.185	7.159
3408	2020-4-26 8:00	EG	EMRO	227	4319	13	307	49	7.108	7.113
3409	2020-4-27 8:00	EG	EMRO	463	4782	30	337	50	7.047	7.092
3410	2020-4-28 8:00	EG	EMRO	260	5042	22	359	51	7.120	7.127
3411	2020-4-29 8:00	EG	EMRO	226	5268	21	380	52	7.213	7.138
3412	2020-4-30 8:00	EG	EMRO	269	5537	12	392	53	7.080	7.060
3413	2020-5-1 8:00	EG	EMRO	358	5895	14	406	54	6.887	6.951
3414	2020-5-2 8:00	EG	EMRO	0	5895	0	406	55	6.887	6.887
3415										
3416	2020-3-19 8:00	SV	AMRO	1	1	0	0			
3417	2020-3-20 8:00	SV	AMRO	0	1	0	0			
3418	2020-3-21 8:00	SV	AMRO	0	1	0	0			
3419	2020-3-22 8:00	SV	AMRO	0	1	0	0			
3420	2020-3-23 8:00	SV	AMRO	0	1	0	0			

Table S1 Daily confirmed cases and daily deaths of COVID-19 in 214 nations from WHO

ID	Date and time	Country or region	Continent	Confirmed patients	Cumulative Confirmed patients	Daily dead patients	Daily cumulative dead patients	Lag days	Daily CFR	3DMA CFR
3421	2020-3-24 8:00	SV	AMRO	4	5	0	0			
3422	2020-3-25 8:00	SV	AMRO	0	5	0	0			
3423	2020-3-26 8:00	SV	AMRO	8	13	0	0			
3424	2020-3-27 8:00	SV	AMRO	0	13	0	0			
3425	2020-3-28 8:00	SV	AMRO	0	13	0	0			
3426	2020-3-29 8:00	SV	AMRO	6	19	0	0			
3427	2020-3-30 8:00	SV	AMRO	5	24	0	0			
3428	2020-3-31 8:00	SV	AMRO	6	30	0	0			
3429	2020-4-1 8:00	SV	AMRO	0	30	0	0			
3430	2020-4-2 8:00	SV	AMRO	2	32	1	1	0	3.125	4.002
3431	2020-4-3 8:00	SV	AMRO	9	41	1	2	1	4.878	4.117
3432	2020-4-4 8:00	SV	AMRO	5	46	0	2	2	4.348	4.861
3433	2020-4-5 8:00	SV	AMRO	10	56	1	3	3	5.357	4.848
3434	2020-4-6 8:00	SV	AMRO	6	62	0	3	4	4.839	4.848
3435	2020-4-7 8:00	SV	AMRO	7	69	0	3	5	4.348	4.772
3436	2020-4-8 8:00	SV	AMRO	9	78	1	4	6	5.128	4.951
3437	2020-4-9 8:00	SV	AMRO	15	93	1	5	7	5.376	5.120
3438	2020-4-10 8:00	SV	AMRO	10	103	0	5	8	4.854	5.120
3439	2020-4-11 8:00	SV	AMRO	14	117	1	6	9	5.128	5.022
3440	2020-4-12 8:00	SV	AMRO	1	118	0	6	10	5.085	5.004
3441	2020-4-13 8:00	SV	AMRO	7	125	0	6	11	4.800	4.755
3442	2020-4-14 8:00	SV	AMRO	12	137	0	6	12	4.380	4.402
3443	2020-4-15 8:00	SV	AMRO	12	149	0	6	13	4.027	4.060
3444	2020-4-16 8:00	SV	AMRO	10	159	0	6	14	3.774	3.820
3445	2020-4-17 8:00	SV	AMRO	5	164	0	6	15	3.659	3.796
3446	2020-4-18 8:00	SV	AMRO	13	177	1	7	16	3.955	3.766
3447	2020-4-19 8:00	SV	AMRO	13	190	0	7	17	3.684	3.707
3448	2020-4-20 8:00	SV	AMRO	11	201	0	7	18	3.483	3.459
3449	2020-4-21 8:00	SV	AMRO	17	218	0	7	19	3.211	3.268
3450	2020-4-22 8:00	SV	AMRO	7	225	0	7	20	3.111	3.092
3451	2020-4-23 8:00	SV	AMRO	12	237	0	7	21	2.954	3.088
3452	2020-4-24 8:00	SV	AMRO	13	250	1	8	22	3.200	3.073
3453	2020-4-25 8:00	SV	AMRO	11	261	0	8	23	3.065	3.062
3454	2020-4-26 8:00	SV	AMRO	13	274	0	8	24	2.920	2.890
3455	2020-4-27 8:00	SV	AMRO	24	298	0	8	25	2.685	2.694
3456	2020-4-28 8:00	SV	AMRO	25	323	0	8	26	2.477	2.493

Table S1 Daily confirmed cases and daily deaths of COVID-19 in 214 nations from WHO

ID	Date and time	Country or region	Continent	Confirmed patients	Cumulative Confirmed patients	Daily dead patients	Daily cumulative dead patients	Lag days	Daily CFR	3DMA CFR
3457	2020-4-29 8:00	SV	AMRO	22	345	0	8	27	2.319	2.394
3458	2020-4-30 8:00	SV	AMRO	32	377	1	9	28	2.387	2.328
3459	2020-5-1 8:00	SV	AMRO	18	395	0	9	29	2.278	2.341
3460	2020-5-2 8:00	SV	AMRO	29	424	1	10	30	2.358	2.318
3461										
3462	2020-3-14 8:00	GQ	AFRO	1	1	0	0			
3463	2020-3-15 8:00	GQ	AFRO	0	1	0	0			
3464	2020-3-16 8:00	GQ	AFRO	0	1	0	0			
3465	2020-3-17 8:00	GQ	AFRO	0	1	0	0			
3466	2020-3-18 8:00	GQ	AFRO	2	3	0	0			
3467	2020-3-19 8:00	GQ	AFRO	1	4	0	0			
3468	2020-3-20 8:00	GQ	AFRO	0	4	0	0			
3469	2020-3-21 8:00	GQ	AFRO	2	6	0	0			
3470	2020-3-22 8:00	GQ	AFRO	0	6	0	0			
3471	2020-3-23 8:00	GQ	AFRO	0	6	0	0			
3472	2020-3-24 8:00	GQ	AFRO	0	6	0	0			
3473	2020-3-25 8:00	GQ	AFRO	0	6	0	0			
3474	2020-3-26 8:00	GQ	AFRO	0	6	0	0			
3475	2020-3-27 8:00	GQ	AFRO	0	6	0	0			
3476	2020-3-28 8:00	GQ	AFRO	0	6	0	0			
3477	2020-3-29 8:00	GQ	AFRO	0	6	0	0			
3478	2020-3-30 8:00	GQ	AFRO	7	13	0	0			
3479	2020-3-31 8:00	GQ	AFRO	1	14	0	0			
3480	2020-4-1 8:00	GQ	AFRO	0	14	0	0			
3481	2020-4-2 8:00	GQ	AFRO	1	15	0	0			
3482	2020-4-3 8:00	GQ	AFRO	0	15	0	0			
3483	2020-4-4 8:00	GQ	AFRO	0	15	0	0			
3484	2020-4-5 8:00	GQ	AFRO	1	16	0	0			
3485	2020-4-6 8:00	GQ	AFRO	0	16	0	0			
3486	2020-4-7 8:00	GQ	AFRO	0	16	0	0			
3487	2020-4-8 8:00	GQ	AFRO	0	16	0	0			
3488	2020-4-9 8:00	GQ	AFRO	0	16	0	0			
3489	2020-4-10 8:00	GQ	AFRO	2	18	0	0			
3490	2020-4-11 8:00	GQ	AFRO	0	18	0	0			
3491	2020-4-12 8:00	GQ	AFRO	0	18	0	0			
3492	2020-4-13 8:00	GQ	AFRO	0	18	0	0			

Table S1 Daily confirmed cases and daily deaths of COVID-19 in 214 nations from WHO

ID	Date and time	Country or region	Continent	Confirmed patients	Cumulative Confirmed patients	Daily dead patients	Daily cumulative dead patients	Lag days	Daily CFR	3DMA CFR
3493	2020-4-14 8:00	GQ	AFRO	3	21	0	0			
3494	2020-4-15 8:00	GQ	AFRO	20	41	0	0			
3495	2020-4-16 8:00	GQ	AFRO	10	51	0	0			
3496	2020-4-17 8:00	GQ	AFRO	0	51	0	0			
3497	2020-4-18 8:00	GQ	AFRO	0	51	0	0			
3498	2020-4-19 8:00	GQ	AFRO	28	79	0	0			
3499	2020-4-20 8:00	GQ	AFRO	0	79	0	0			
3500	2020-4-21 8:00	GQ	AFRO	0	79	0	0			
3501	2020-4-22 8:00	GQ	AFRO	0	79	0	0			
3502	2020-4-23 8:00	GQ	AFRO	5	84	1	1	0	1.190	1.190
3503	2020-4-24 8:00	GQ	AFRO	0	84	0	1	1	1.190	0.951
3504	2020-4-25 8:00	GQ	AFRO	128	212	0	1	2	0.472	0.683
3505	2020-4-26 8:00	GQ	AFRO	46	258	0	1	3	0.388	0.416
3506	2020-4-27 8:00	GQ	AFRO	0	258	0	1	4	0.388	0.388
3507	2020-4-28 8:00	GQ	AFRO	0	258	0	1	5	0.388	0.364
3508	2020-4-29 8:00	GQ	AFRO	57	315	0	1	6	0.317	0.341
3509	2020-4-30 8:00	GQ	AFRO	0	315	0	1	7	0.317	0.423
3510	2020-5-1 8:00	GQ	AFRO	0	315	1	2	8	0.635	0.529
3511	2020-5-2 8:00	GQ	AFRO	0	315	0	2	9	0.635	0.635
3512										
3513	2020-3-21 8:00	ER	AFRO	1	1	0	0			
3514	2020-3-22 8:00	ER	AFRO	0	1	0	0			
3515	2020-3-23 8:00	ER	AFRO	0	1	0	0			
3516	2020-3-24 8:00	ER	AFRO	0	1	0	0			
3517	2020-3-25 8:00	ER	AFRO	0	1	0	0			
3518	2020-3-26 8:00	ER	AFRO	3	4	0	0			
3519	2020-3-27 8:00	ER	AFRO	2	6	0	0			
3520	2020-3-28 8:00	ER	AFRO	0	6	0	0			
3521	2020-3-29 8:00	ER	AFRO	0	6	0	0			
3522	2020-3-30 8:00	ER	AFRO	0	6	0	0			
3523	2020-3-31 8:00	ER	AFRO	0	6	0	0			
3524	2020-4-1 8:00	ER	AFRO	0	6	0	0			
3525	2020-4-2 8:00	ER	AFRO	9	15	0	0			
3526	2020-4-3 8:00	ER	AFRO	5	20	0	0			
3527	2020-4-4 8:00	ER	AFRO	0	20	0	0			
3528	2020-4-5 8:00	ER	AFRO	0	20	0	0			

Table S1 Daily confirmed cases and daily deaths of COVID-19 in 214 nations from WHO

ID	Date and time	Country or region	Continent	Confirmed patients	Cumulative Confirmed patients	Daily dead patients	Daily cumulative dead patients	Lag days	Daily CFR	3DMA CFR
3529	2020-4-6 8:00	ER	AFRO	9	29	0	0			
3530	2020-4-7 8:00	ER	AFRO	0	29	0	0			
3531	2020-4-8 8:00	ER	AFRO	2	31	0	0			
3532	2020-4-9 8:00	ER	AFRO	2	33	0	0			
3533	2020-4-10 8:00	ER	AFRO	0	33	0	0			
3534	2020-4-11 8:00	ER	AFRO	0	33	0	0			
3535	2020-4-12 8:00	ER	AFRO	0	33	0	0			
3536	2020-4-13 8:00	ER	AFRO	1	34	0	0			
3537	2020-4-14 8:00	ER	AFRO	0	34	0	0			
3538	2020-4-15 8:00	ER	AFRO	0	34	0	0			
3539	2020-4-16 8:00	ER	AFRO	1	35	0	0			
3540	2020-4-17 8:00	ER	AFRO	0	35	0	0			
3541	2020-4-18 8:00	ER	AFRO	0	35	0	0			
3542	2020-4-19 8:00	ER	AFRO	4	39	0	0			
3543	2020-4-20 8:00	ER	AFRO	0	39	0	0			
3544	2020-4-21 8:00	ER	AFRO	0	39	0	0			
3545	2020-4-22 8:00	ER	AFRO	0	39	0	0			
3546	2020-4-23 8:00	ER	AFRO	0	39	0	0			
3547	2020-4-24 8:00	ER	AFRO	0	39	0	0			
3548	2020-4-25 8:00	ER	AFRO	0	39	0	0			
3549	2020-4-26 8:00	ER	AFRO	0	39	0	0			
3550	2020-4-27 8:00	ER	AFRO	0	39	0	0			
3551	2020-4-28 8:00	ER	AFRO	0	39	0	0			
3552	2020-4-29 8:00	ER	AFRO	0	39	0	0			
3553	2020-4-30 8:00	ER	AFRO	0	39	0	0			
3554	2020-5-1 8:00	ER	AFRO	0	39	0	0			
3555	2020-5-2 8:00	ER	AFRO	0	39	0	0			
3556										
3557	2020-2-27 8:00	EE	EURO	1	1	0	0			
3558	2020-2-28 8:00	EE	EURO	0	1	0	0			
3559	2020-2-29 8:00	EE	EURO	0	1	0	0			
3560	2020-3-1 8:00	EE	EURO	0	1	0	0			
3561	2020-3-2 8:00	EE	EURO	0	1	0	0			
3562	2020-3-3 8:00	EE	EURO	1	2	0	0			
3563	2020-3-4 8:00	EE	EURO	0	2	0	0			
3564	2020-3-5 8:00	EE	EURO	1	3	0	0			

Table S1 Daily confirmed cases and daily deaths of COVID-19 in 214 nations from WHO

ID	Date and time	Country or region	Continent	Confirmed patients	Cumulative Confirmed patients	Daily dead patients	Daily cumulative dead patients	Lag days	Daily CFR	3DMA CFR
3565	2020-3-6 8:00	EE	EURO	7	10	0	0			
3566	2020-3-7 8:00	EE	EURO	0	10	0	0			
3567	2020-3-8 8:00	EE	EURO	0	10	0	0			
3568	2020-3-9 8:00	EE	EURO	0	10	0	0			
3569	2020-3-10 8:00	EE	EURO	0	10	0	0			
3570	2020-3-11 8:00	EE	EURO	3	13	0	0			
3571	2020-3-12 8:00	EE	EURO	0	13	0	0			
3572	2020-3-13 8:00	EE	EURO	66	79	0	0			
3573	2020-3-14 8:00	EE	EURO	0	79	0	0			
3574	2020-3-15 8:00	EE	EURO	92	171	0	0			
3575	2020-3-16 8:00	EE	EURO	34	205	0	0			
3576	2020-3-17 8:00	EE	EURO	20	225	0	0			
3577	2020-3-18 8:00	EE	EURO	33	258	0	0			
3578	2020-3-19 8:00	EE	EURO	9	267	0	0			
3579	2020-3-20 8:00	EE	EURO	16	283	0	0			
3580	2020-3-21 8:00	EE	EURO	23	306	0	0			
3581	2020-3-22 8:00	EE	EURO	0	306	0	0			
3582	2020-3-23 8:00	EE	EURO	20	326	0	0			
3583	2020-3-24 8:00	EE	EURO	26	352	0	0			
3584	2020-3-25 8:00	EE	EURO	17	369	0	0			
3585	2020-3-26 8:00	EE	EURO	35	404	1	1	0	0.248	0.217
3586	2020-3-27 8:00	EE	EURO	134	538	0	1	1	0.186	0.202
3587	2020-3-28 8:00	EE	EURO	37	575	0	1	2	0.174	0.172
3588	2020-3-29 8:00	EE	EURO	65	640	0	1	3	0.156	0.257
3589	2020-3-30 8:00	EE	EURO	39	679	2	3	4	0.442	0.339
3590	2020-3-31 8:00	EE	EURO	36	715	0	3	5	0.420	0.466
3591	2020-4-1 8:00	EE	EURO	30	745	1	4	6	0.537	0.533
3592	2020-4-2 8:00	EE	EURO	34	779	1	5	7	0.642	0.820
3593	2020-4-3 8:00	EE	EURO	79	858	6	11	8	1.282	1.058
3594	2020-4-4 8:00	EE	EURO	103	961	1	12	9	1.249	1.269
3595	2020-4-5 8:00	EE	EURO	57	1018	1	13	10	1.277	1.298
3596	2020-4-6 8:00	EE	EURO	79	1097	2	15	11	1.367	1.453
3597	2020-4-7 8:00	EE	EURO	11	1108	4	19	12	1.715	1.637
3598	2020-4-8 8:00	EE	EURO	41	1149	2	21	13	1.828	1.856
3599	2020-4-9 8:00	EE	EURO	36	1185	3	24	14	2.025	1.947
3600	2020-4-10 8:00	EE	EURO	22	1207	0	24	15	1.988	1.974

Table S1 Daily confirmed cases and daily deaths of COVID-19 in 214 nations from WHO

ID	Date and time	Country or region	Continent	Confirmed patients	Cumulative Confirmed patients	Daily dead patients	Daily cumulative dead patients	Lag days	Daily CFR	3DMA CFR
3601	2020-4-11 8:00	EE	EURO	51	1258	0	24	16	1.908	1.912
3602	2020-4-12 8:00	EE	EURO	46	1304	0	24	17	1.840	1.886
3603	2020-4-13 8:00	EE	EURO	5	1309	1	25	18	1.910	1.951
3604	2020-4-14 8:00	EE	EURO	23	1332	3	28	19	2.102	2.090
3605	2020-4-15 8:00	EE	EURO	41	1373	3	31	20	2.258	2.285
3606	2020-4-16 8:00	EE	EURO	29	1402	4	35	21	2.496	2.422
3607	2020-4-17 8:00	EE	EURO	32	1434	1	36	22	2.510	2.537
3608	2020-4-18 8:00	EE	EURO	25	1459	2	38	23	2.605	2.543
3609	2020-4-19 8:00	EE	EURO	53	1512	0	38	24	2.513	2.579
3610	2020-4-20 8:00	EE	EURO	16	1528	2	40	25	2.618	2.579
3611	2020-4-21 8:00	EE	EURO	7	1535	0	40	26	2.606	2.665
3612	2020-4-22 8:00	EE	EURO	17	1552	3	43	27	2.771	2.733
3613	2020-4-23 8:00	EE	EURO	7	1559	1	44	28	2.822	2.807
3614	2020-4-24 8:00	EE	EURO	33	1592	1	45	29	2.827	2.838
3615	2020-4-25 8:00	EE	EURO	13	1605	1	46	30	2.866	2.835
3616	2020-4-26 8:00	EE	EURO	30	1635	0	46	31	2.813	2.887
3617	2020-4-27 8:00	EE	EURO	8	1643	3	49	32	2.982	2.944
3618	2020-4-28 8:00	EE	EURO	4	1647	1	50	33	3.036	3.010
3619	2020-4-29 8:00	EE	EURO	13	1660	0	50	34	3.012	3.016
3620	2020-4-30 8:00	EE	EURO	6	1666	0	50	35	3.001	3.031
3621	2020-5-1 8:00	EE	EURO	23	1689	2	52	36	3.079	3.050
3622	2020-5-2 8:00	EE	EURO	5	1694	0	52	37	3.070	3.074
3623										
3624	2020-3-14 8:00	SZ	AFRO	1	1	0	0			
3625	2020-3-15 8:00	SZ	AFRO	0	1	0	0			
3626	2020-3-16 8:00	SZ	AFRO	0	1	0	0			
3627	2020-3-17 8:00	SZ	AFRO	0	1	0	0			
3628	2020-3-18 8:00	SZ	AFRO	0	1	0	0			
3629	2020-3-19 8:00	SZ	AFRO	0	1	0	0			
3630	2020-3-20 8:00	SZ	AFRO	0	1	0	0			
3631	2020-3-21 8:00	SZ	AFRO	0	1	0	0			
3632	2020-3-22 8:00	SZ	AFRO	0	1	0	0			
3633	2020-3-23 8:00	SZ	AFRO	3	4	0	0			
3634	2020-3-24 8:00	SZ	AFRO	0	4	0	0			
3635	2020-3-25 8:00	SZ	AFRO	0	4	0	0			
3636	2020-3-26 8:00	SZ	AFRO	0	4	0	0			

Table S1 Daily confirmed cases and daily deaths of COVID-19 in 214 nations from WHO

ID	Date and time	Country or region	Continent	Confirmed patients	Cumulative Confirmed patients	Daily dead patients	Daily cumulative dead patients	Lag days	Daily CFR	3DMA CFR
3637	2020-3-27 8:00	SZ	AFRO	2	6	0	0			
3638	2020-3-28 8:00	SZ	AFRO	3	9	0	0			
3639	2020-3-29 8:00	SZ	AFRO	0	9	0	0			
3640	2020-3-30 8:00	SZ	AFRO	0	9	0	0			
3641	2020-3-31 8:00	SZ	AFRO	0	9	0	0			
3642	2020-4-1 8:00	SZ	AFRO	0	9	0	0			
3643	2020-4-2 8:00	SZ	AFRO	0	9	0	0			
3644	2020-4-3 8:00	SZ	AFRO	0	9	0	0			
3645	2020-4-4 8:00	SZ	AFRO	0	9	0	0			
3646	2020-4-5 8:00	SZ	AFRO	0	9	0	0			
3647	2020-4-6 8:00	SZ	AFRO	0	9	0	0			
3648	2020-4-7 8:00	SZ	AFRO	0	9	0	0			
3649	2020-4-8 8:00	SZ	AFRO	1	10	0	0			
3650	2020-4-9 8:00	SZ	AFRO	2	12	0	0			
3651	2020-4-10 8:00	SZ	AFRO	0	12	0	0			
3652	2020-4-11 8:00	SZ	AFRO	0	12	0	0			
3653	2020-4-12 8:00	SZ	AFRO	0	12	0	0			
3654	2020-4-13 8:00	SZ	AFRO	2	14	0	0			
3655	2020-4-14 8:00	SZ	AFRO	0	14	0	0			
3656	2020-4-15 8:00	SZ	AFRO	1	15	0	0			
3657	2020-4-16 8:00	SZ	AFRO	1	16	0	0			
3658	2020-4-17 8:00	SZ	AFRO	0	16	1	1	0	6.250	5.757
3659	2020-4-18 8:00	SZ	AFRO	3	19	0	1	1	5.263	5.353
3660	2020-4-19 8:00	SZ	AFRO	3	22	0	1	2	4.545	4.785
3661	2020-4-20 8:00	SZ	AFRO	0	22	0	1	3	4.545	4.419
3662	2020-4-21 8:00	SZ	AFRO	2	24	0	1	4	4.167	4.293
3663	2020-4-22 8:00	SZ	AFRO	0	24	0	1	5	4.167	3.853
3664	2020-4-23 8:00	SZ	AFRO	7	31	0	1	6	3.226	3.539
3665	2020-4-24 8:00	SZ	AFRO	0	31	0	1	7	3.226	2.984
3666	2020-4-25 8:00	SZ	AFRO	9	40	0	1	8	2.500	2.504
3667	2020-4-26 8:00	SZ	AFRO	16	56	0	1	9	1.786	1.994
3668	2020-4-27 8:00	SZ	AFRO	3	59	0	1	10	1.695	1.673
3669	2020-4-28 8:00	SZ	AFRO	6	65	0	1	11	1.538	1.547
3670	2020-4-29 8:00	SZ	AFRO	6	71	0	1	12	1.408	1.349
3671	2020-4-30 8:00	SZ	AFRO	20	91	0	1	13	1.099	1.169
3672	2020-5-1 8:00	SZ	AFRO	9	100	0	1	14	1.000	1.014

Table S1 Daily confirmed cases and daily deaths of COVID-19 in 214 nations from WHO

ID	Date and time	Country or region	Continent	Confirmed patients	Cumulative Confirmed patients	Daily dead patients	Daily cumulative dead patients	Lag days	Daily CFR	3DMA CFR
3673	2020-5-2 8:00	SZ	AFRO	6	106	0	1	15	0.943	0.972
3674										
3675	2020-3-14 8:00	ET	AFRO	1	1	0	0			
3676	2020-3-15 8:00	ET	AFRO	0	1	0	0			
3677	2020-3-16 8:00	ET	AFRO	3	4	0	0			
3678	2020-3-17 8:00	ET	AFRO	1	5	0	0			
3679	2020-3-18 8:00	ET	AFRO	1	6	0	0			
3680	2020-3-19 8:00	ET	AFRO	3	9	0	0			
3681	2020-3-20 8:00	ET	AFRO	0	9	0	0			
3682	2020-3-21 8:00	ET	AFRO	0	9	0	0			
3683	2020-3-22 8:00	ET	AFRO	0	9	0	0			
3684	2020-3-23 8:00	ET	AFRO	2	11	0	0			
3685	2020-3-24 8:00	ET	AFRO	0	11	0	0			
3686	2020-3-25 8:00	ET	AFRO	1	12	0	0			
3687	2020-3-26 8:00	ET	AFRO	0	12	0	0			
3688	2020-3-27 8:00	ET	AFRO	0	12	0	0			
3689	2020-3-28 8:00	ET	AFRO	4	16	0	0			
3690	2020-3-29 8:00	ET	AFRO	0	16	0	0			
3691	2020-3-30 8:00	ET	AFRO	5	21	0	0			
3692	2020-3-31 8:00	ET	AFRO	2	23	0	0			
3693	2020-4-1 8:00	ET	AFRO	3	26	0	0			
3694	2020-4-2 8:00	ET	AFRO	0	26	0	0			
3695	2020-4-3 8:00	ET	AFRO	5	31	0	0			
3696	2020-4-4 8:00	ET	AFRO	4	35	0	0			
3697	2020-4-5 8:00	ET	AFRO	3	38	0	0			
3698	2020-4-6 8:00	ET	AFRO	5	43	1	1	0	2.326	2.299
3699	2020-4-7 8:00	ET	AFRO	1	44	0	1	1	2.273	2.815
3700	2020-4-8 8:00	ET	AFRO	8	52	1	2	2	3.846	3.252
3701	2020-4-9 8:00	ET	AFRO	3	55	0	2	3	3.636	3.685
3702	2020-4-10 8:00	ET	AFRO	1	56	0	2	4	3.571	3.428
3703	2020-4-11 8:00	ET	AFRO	9	65	0	2	5	3.077	3.665
3704	2020-4-12 8:00	ET	AFRO	4	69	1	3	6	4.348	3.883
3705	2020-4-13 8:00	ET	AFRO	2	71	0	3	7	4.225	4.209
3706	2020-4-14 8:00	ET	AFRO	3	74	0	3	8	4.054	3.979
3707	2020-4-15 8:00	ET	AFRO	8	82	0	3	9	3.659	3.747
3708	2020-4-16 8:00	ET	AFRO	3	85	0	3	10	3.529	3.483

Table S1 Daily confirmed cases and daily deaths of COVID-19 in 214 nations from WHO

ID	Date and time	Country or region	Continent	Confirmed patients	Cumulative Confirmed patients	Daily dead patients	Daily cumulative dead patients	Lag days	Daily CFR	3DMA CFR
3709	2020-4-17 8:00	ET	AFRO	7	92	0	3	11	3.261	3.305
3710	2020-4-18 8:00	ET	AFRO	4	96	0	3	12	3.125	3.081
3711	2020-4-19 8:00	ET	AFRO	9	105	0	3	13	2.857	2.920
3712	2020-4-20 8:00	ET	AFRO	3	108	0	3	14	2.778	2.779
3713	2020-4-21 8:00	ET	AFRO	3	111	0	3	15	2.703	2.704
3714	2020-4-22 8:00	ET	AFRO	3	114	0	3	16	2.632	2.640
3715	2020-4-23 8:00	ET	AFRO	2	116	0	3	17	2.586	2.601
3716	2020-4-24 8:00	ET	AFRO	0	116	0	3	18	2.586	2.579
3717	2020-4-25 8:00	ET	AFRO	1	117	0	3	19	2.564	2.536
3718	2020-4-26 8:00	ET	AFRO	5	122	0	3	20	2.459	2.487
3719	2020-4-27 8:00	ET	AFRO	1	123	0	3	21	2.439	2.439
3720	2020-4-28 8:00	ET	AFRO	1	124	0	3	22	2.419	2.413
3721	2020-4-29 8:00	ET	AFRO	2	126	0	3	23	2.381	2.369
3722	2020-4-30 8:00	ET	AFRO	4	130	0	3	24	2.308	2.326
3723	2020-5-1 8:00	ET	AFRO	1	131	0	3	25	2.290	2.284
3724	2020-5-2 8:00	ET	AFRO	2	133	0	3	26	2.256	2.273
3725										
3726	2020-4-5 8:00	FK	AMRO	1	1	0	0			
3727	2020-4-6 8:00	FK	AMRO	1	2	0	0			
3728	2020-4-7 8:00	FK	AMRO	0	2	0	0			
3729	2020-4-8 8:00	FK	AMRO	0	2	0	0			
3730	2020-4-9 8:00	FK	AMRO	3	5	0	0			
3731	2020-4-10 8:00	FK	AMRO	0	5	0	0			
3732	2020-4-11 8:00	FK	AMRO	0	5	0	0			
3733	2020-4-12 8:00	FK	AMRO	0	5	0	0			
3734	2020-4-13 8:00	FK	AMRO	0	5	0	0			
3735	2020-4-14 8:00	FK	AMRO	0	5	0	0			
3736	2020-4-15 8:00	FK	AMRO	6	11	0	0			
3737	2020-4-16 8:00	FK	AMRO	0	11	0	0			
3738	2020-4-17 8:00	FK	AMRO	0	11	0	0			
3739	2020-4-18 8:00	FK	AMRO	0	11	0	0			
3740	2020-4-19 8:00	FK	AMRO	0	11	0	0			
3741	2020-4-20 8:00	FK	AMRO	0	11	0	0			
3742	2020-4-21 8:00	FK	AMRO	0	11	0	0			
3743	2020-4-22 8:00	FK	AMRO	0	11	0	0			
3744	2020-4-23 8:00	FK	AMRO	1	12	0	0			

Table S1 Daily confirmed cases and daily deaths of COVID-19 in 214 nations from WHO

ID	Date and time	Country or region	Continent	Confirmed patients	Cumulative Confirmed patients	Daily dead patients	Daily cumulative dead patients	Lag days	Daily CFR	3DMA CFR
3745	2020-4-24 8:00	FK	AMRO	0	12	0	0			
3746	2020-4-25 8:00	FK	AMRO	0	12	0	0			
3747	2020-4-26 8:00	FK	AMRO	1	13	0	0			
3748	2020-4-27 8:00	FK	AMRO	0	13	0	0			
3749	2020-4-28 8:00	FK	AMRO	0	13	0	0			
3750	2020-4-29 8:00	FK	AMRO	0	13	0	0			
3751	2020-4-30 8:00	FK	AMRO	0	13	0	0			
3752	2020-5-1 8:00	FK	AMRO	0	13	0	0			
3753	2020-5-2 8:00	FK	AMRO	0	13	0	0			
3754										
3755	2020-3-5 8:00	FO	EURO	1	1	0	0			
3756	2020-3-6 8:00	FO	EURO	0	1	0	0			
3757	2020-3-7 8:00	FO	EURO	0	1	0	0			
3758	2020-3-8 8:00	FO	EURO	1	2	0	0			
3759	2020-3-9 8:00	FO	EURO	0	2	0	0			
3760	2020-3-10 8:00	FO	EURO	0	2	0	0			
3761	2020-3-11 8:00	FO	EURO	0	2	0	0			
3762	2020-3-12 8:00	FO	EURO	0	2	0	0			
3763	2020-3-13 8:00	FO	EURO	1	3	0	0			
3764	2020-3-14 8:00	FO	EURO	6	9	0	0			
3765	2020-3-15 8:00	FO	EURO	0	9	0	0			
3766	2020-3-16 8:00	FO	EURO	2	11	0	0			
3767	2020-3-17 8:00	FO	EURO	36	47	0	0			
3768	2020-3-18 8:00	FO	EURO	11	58	0	0			
3769	2020-3-19 8:00	FO	EURO	14	72	0	0			
3770	2020-3-20 8:00	FO	EURO	8	80	0	0			
3771	2020-3-21 8:00	FO	EURO	12	92	0	0			
3772	2020-3-22 8:00	FO	EURO	0	92	0	0			
3773	2020-3-23 8:00	FO	EURO	23	115	0	0			
3774	2020-3-24 8:00	FO	EURO	3	118	0	0			
3775	2020-3-25 8:00	FO	EURO	4	122	0	0			
3776	2020-3-26 8:00	FO	EURO	10	132	0	0			
3777	2020-3-27 8:00	FO	EURO	8	140	0	0			
3778	2020-3-28 8:00	FO	EURO	4	144	0	0			
3779	2020-3-29 8:00	FO	EURO	11	155	0	0			
3780	2020-3-30 8:00	FO	EURO	4	159	0	0			

Table S1 Daily confirmed cases and daily deaths of COVID-19 in 214 nations from WHO

ID	Date and time	Country or region	Continent	Confirmed patients	Cumulative Confirmed patients	Daily dead patients	Daily cumulative dead patients	Lag days	Daily CFR	3DMA CFR
3781	2020-3-31 8:00	FO	EURO	9	168	0	0			
3782	2020-4-1 8:00	FO	EURO	1	169	0	0			
3783	2020-4-2 8:00	FO	EURO	4	173	0	0			
3784	2020-4-3 8:00	FO	EURO	4	177	0	0			
3785	2020-4-4 8:00	FO	EURO	2	179	0	0			
3786	2020-4-5 8:00	FO	EURO	2	181	0	0			
3787	2020-4-6 8:00	FO	EURO	0	181	0	0			
3788	2020-4-7 8:00	FO	EURO	0	181	0	0			
3789	2020-4-8 8:00	FO	EURO	3	184	0	0			
3790	2020-4-9 8:00	FO	EURO	0	184	0	0			
3791	2020-4-10 8:00	FO	EURO	0	184	0	0			
3792	2020-4-11 8:00	FO	EURO	0	184	0	0			
3793	2020-4-12 8:00	FO	EURO	0	184	0	0			
3794	2020-4-13 8:00	FO	EURO	0	184	0	0			
3795	2020-4-14 8:00	FO	EURO	0	184	0	0			
3796	2020-4-15 8:00	FO	EURO	0	184	0	0			
3797	2020-4-16 8:00	FO	EURO	0	184	0	0			
3798	2020-4-17 8:00	FO	EURO	0	184	0	0			
3799	2020-4-18 8:00	FO	EURO	0	184	0	0			
3800	2020-4-19 8:00	FO	EURO	0	184	0	0			
3801	2020-4-20 8:00	FO	EURO	1	185	0	0			
3802	2020-4-21 8:00	FO	EURO	0	185	0	0			
3803	2020-4-22 8:00	FO	EURO	0	185	0	0			
3804	2020-4-23 8:00	FO	EURO	0	185	0	0			
3805	2020-4-24 8:00	FO	EURO	2	187	0	0			
3806	2020-4-25 8:00	FO	EURO	0	187	0	0			
3807	2020-4-26 8:00	FO	EURO	0	187	0	0			
3808	2020-4-27 8:00	FO	EURO	0	187	0	0			
3809	2020-4-28 8:00	FO	EURO	0	187	0	0			
3810	2020-4-29 8:00	FO	EURO	0	187	0	0			
3811	2020-4-30 8:00	FO	EURO	0	187	0	0			
3812	2020-5-1 8:00	FO	EURO	0	187	0	0			
3813	2020-5-2 8:00	FO	EURO	0	187	0	0			
3814										
3815	2020-3-19 8:00	FJ	WPRO	1	1	0	0			
3816	2020-3-20 8:00	FJ	WPRO	0	1	0	0			

Table S1 Daily confirmed cases and daily deaths of COVID-19 in 214 nations from WHO

ID	Date and time	Country or region	Continent	Confirmed patients	Cumulative Confirmed patients	Daily dead patients	Daily cumulative dead patients	Lag days	Daily CFR	3DMA CFR
3817	2020-3-21 8:00	FJ	WPRO	1	2	0	0			
3818	2020-3-22 8:00	FJ	WPRO	0	2	0	0			
3819	2020-3-23 8:00	FJ	WPRO	1	3	0	0			
3820	2020-3-24 8:00	FJ	WPRO	1	4	0	0			
3821	2020-3-25 8:00	FJ	WPRO	1	5	0	0			
3822	2020-3-26 8:00	FJ	WPRO	0	5	0	0			
3823	2020-3-27 8:00	FJ	WPRO	0	5	0	0			
3824	2020-3-28 8:00	FJ	WPRO	0	5	0	0			
3825	2020-3-29 8:00	FJ	WPRO	0	5	0	0			
3826	2020-3-30 8:00	FJ	WPRO	0	5	0	0			
3827	2020-3-31 8:00	FJ	WPRO	0	5	0	0			
3828	2020-4-1 8:00	FJ	WPRO	0	5	0	0			
3829	2020-4-2 8:00	FJ	WPRO	0	5	0	0			
3830	2020-4-3 8:00	FJ	WPRO	2	7	0	0			
3831	2020-4-4 8:00	FJ	WPRO	0	7	0	0			
3832	2020-4-5 8:00	FJ	WPRO	5	12	0	0			
3833	2020-4-6 8:00	FJ	WPRO	2	14	0	0			
3834	2020-4-7 8:00	FJ	WPRO	1	15	0	0			
3835	2020-4-8 8:00	FJ	WPRO	0	15	0	0			
3836	2020-4-9 8:00	FJ	WPRO	0	15	0	0			
3837	2020-4-10 8:00	FJ	WPRO	0	15	0	0			
3838	2020-4-11 8:00	FJ	WPRO	1	16	0	0			
3839	2020-4-12 8:00	FJ	WPRO	0	16	0	0			
3840	2020-4-13 8:00	FJ	WPRO	0	16	0	0			
3841	2020-4-14 8:00	FJ	WPRO	0	16	0	0			
3842	2020-4-15 8:00	FJ	WPRO	0	16	0	0			
3843	2020-4-16 8:00	FJ	WPRO	0	16	0	0			
3844	2020-4-17 8:00	FJ	WPRO	1	17	0	0			
3845	2020-4-18 8:00	FJ	WPRO	0	17	0	0			
3846	2020-4-19 8:00	FJ	WPRO	0	17	0	0			
3847	2020-4-20 8:00	FJ	WPRO	0	17	0	0			
3848	2020-4-21 8:00	FJ	WPRO	1	18	0	0			
3849	2020-4-22 8:00	FJ	WPRO	0	18	0	0			
3850	2020-4-23 8:00	FJ	WPRO	0	18	0	0			
3851	2020-4-24 8:00	FJ	WPRO	0	18	0	0			
3852	2020-4-25 8:00	FJ	WPRO	0	18	0	0			

Table S1 Daily confirmed cases and daily deaths of COVID-19 in 214 nations from WHO

ID	Date and time	Country or region	Continent	Confirmed patients	Cumulative Confirmed patients	Daily dead patients	Daily cumulative dead patients	Lag days	Daily CFR	3DMA CFR
3853	2020-4-26 8:00	FJ	WPRO	0	18	0	0			
3854	2020-4-27 8:00	FJ	WPRO	0	18	0	0			
3855	2020-4-28 8:00	FJ	WPRO	0	18	0	0			
3856	2020-4-29 8:00	FJ	WPRO	0	18	0	0			
3857	2020-4-30 8:00	FJ	WPRO	0	18	0	0			
3858	2020-5-1 8:00	FJ	WPRO	0	18	0	0			
3859	2020-5-2 8:00	FJ	WPRO	0	18	0	0			
3860										
3861	2020-1-29 8:00	FI	EURO	1	1	0	0			
3862	2020-1-30 8:00	FI	EURO	0	1	0	0			
3863	2020-1-31 8:00	FI	EURO	0	1	0	0			
3864	2020-2-1 8:00	FI	EURO	0	1	0	0			
3865	2020-2-2 8:00	FI	EURO	0	1	0	0			
3866	2020-2-3 8:00	FI	EURO	0	1	0	0			
3867	2020-2-4 8:00	FI	EURO	0	1	0	0			
3868	2020-2-5 8:00	FI	EURO	0	1	0	0			
3869	2020-2-6 8:00	FI	EURO	0	1	0	0			
3870	2020-2-7 8:00	FI	EURO	0	1	0	0			
3871	2020-2-8 8:00	FI	EURO	0	1	0	0			
3872	2020-2-9 8:00	FI	EURO	0	1	0	0			
3873	2020-2-10 8:00	FI	EURO	0	1	0	0			
3874	2020-2-11 8:00	FI	EURO	0	1	0	0			
3875	2020-2-12 8:00	FI	EURO	0	1	0	0			
3876	2020-2-13 8:00	FI	EURO	0	1	0	0			
3877	2020-2-14 8:00	FI	EURO	0	1	0	0			
3878	2020-2-15 8:00	FI	EURO	0	1	0	0			
3879	2020-2-16 8:00	FI	EURO	0	1	0	0			
3880	2020-2-17 8:00	FI	EURO	0	1	0	0			
3881	2020-2-18 8:00	FI	EURO	0	1	0	0			
3882	2020-2-19 8:00	FI	EURO	0	1	0	0			
3883	2020-2-20 8:00	FI	EURO	0	1	0	0			
3884	2020-2-21 8:00	FI	EURO	0	1	0	0			
3885	2020-2-22 8:00	FI	EURO	0	1	0	0			
3886	2020-2-23 8:00	FI	EURO	0	1	0	0			
3887	2020-2-24 8:00	FI	EURO	0	1	0	0			
3888	2020-2-25 8:00	FI	EURO	0	1	0	0			

Table S1 Daily confirmed cases and daily deaths of COVID-19 in 214 nations from WHO

ID	Date and time	Country or region	Continent	Confirmed patients	Cumulative Confirmed patients	Daily dead patients	Daily cumulative dead patients	Lag days	Daily CFR	3DMA CFR
3889	2020-2-26 8:00	FI	EURO	1	2	0	0			
3890	2020-2-27 8:00	FI	EURO	0	2	0	0			
3891	2020-2-28 8:00	FI	EURO	0	2	0	0			
3892	2020-2-29 8:00	FI	EURO	0	2	0	0			
3893	2020-3-1 8:00	FI	EURO	4	6	0	0			
3894	2020-3-2 8:00	FI	EURO	1	7	0	0			
3895	2020-3-3 8:00	FI	EURO	0	7	0	0			
3896	2020-3-4 8:00	FI	EURO	0	7	0	0			
3897	2020-3-5 8:00	FI	EURO	5	12	0	0			
3898	2020-3-6 8:00	FI	EURO	7	19	0	0			
3899	2020-3-7 8:00	FI	EURO	0	19	0	0			
3900	2020-3-8 8:00	FI	EURO	11	30	0	0			
3901	2020-3-9 8:00	FI	EURO	10	40	0	0			
3902	2020-3-10 8:00	FI	EURO	0	40	0	0			
3903	2020-3-11 8:00	FI	EURO	0	40	0	0			
3904	2020-3-12 8:00	FI	EURO	115	155	0	0			
3905	2020-3-13 8:00	FI	EURO	0	155	0	0			
3906	2020-3-14 8:00	FI	EURO	55	210	0	0			
3907	2020-3-15 8:00	FI	EURO	57	267	0	0			
3908	2020-3-16 8:00	FI	EURO	5	272	0	0			
3909	2020-3-17 8:00	FI	EURO	47	319	0	0			
3910	2020-3-18 8:00	FI	EURO	40	359	0	0			
3911	2020-3-19 8:00	FI	EURO	10	369	0	0			
3912	2020-3-20 8:00	FI	EURO	81	450	0	0			
3913	2020-3-21 8:00	FI	EURO	71	521	1	1	0	0.192	0.192
3914	2020-3-22 8:00	FI	EURO	0	521	0	1	1	0.192	0.181
3915	2020-3-23 8:00	FI	EURO	105	626	0	1	2	0.160	0.165
3916	2020-3-24 8:00	FI	EURO	74	700	0	1	3	0.143	0.143
3917	2020-3-25 8:00	FI	EURO	92	792	0	1	4	0.126	0.203
3918	2020-3-26 8:00	FI	EURO	88	880	2	3	5	0.341	0.295
3919	2020-3-27 8:00	FI	EURO	78	958	1	4	6	0.418	0.480
3920	2020-3-28 8:00	FI	EURO	67	1025	3	7	7	0.683	0.613
3921	2020-3-29 8:00	FI	EURO	193	1218	2	9	8	0.739	0.775
3922	2020-3-30 8:00	FI	EURO	0	1218	2	11	9	0.903	0.877
3923	2020-3-31 8:00	FI	EURO	95	1313	2	13	10	0.990	1.041
3924	2020-4-1 8:00	FI	EURO	71	1384	4	17	11	1.228	1.131

Table S1 Daily confirmed cases and daily deaths of COVID-19 in 214 nations from WHO

ID	Date and time	Country or region	Continent	Confirmed patients	Cumulative Confirmed patients	Daily dead patients	Daily cumulative dead patients	Lag days	Daily CFR	3DMA CFR
3925	2020-4-2 8:00	FI	EURO	62	1446	0	17	12	1.176	1.219
3926	2020-4-3 8:00	FI	EURO	72	1518	2	19	13	1.252	1.222
3927	2020-4-4 8:00	FI	EURO	97	1615	1	20	14	1.238	1.273
3928	2020-4-5 8:00	FI	EURO	267	1882	5	25	15	1.328	1.340
3929	2020-4-6 8:00	FI	EURO	45	1927	3	28	16	1.453	1.356
3930	2020-4-7 8:00	FI	EURO	249	2176	0	28	17	1.287	1.404
3931	2020-4-8 8:00	FI	EURO	132	2308	6	34	18	1.473	1.456
3932	2020-4-9 8:00	FI	EURO	179	2487	6	40	19	1.608	1.565
3933	2020-4-10 8:00	FI	EURO	118	2605	2	42	20	1.612	1.651
3934	2020-4-11 8:00	FI	EURO	164	2769	6	48	21	1.733	1.678
3935	2020-4-12 8:00	FI	EURO	136	2905	1	49	22	1.687	1.768
3936	2020-4-13 8:00	FI	EURO	69	2974	7	56	23	1.883	1.832
3937	2020-4-14 8:00	FI	EURO	90	3064	3	59	24	1.926	1.944
3938	2020-4-15 8:00	FI	EURO	97	3161	5	64	25	2.025	2.058
3939	2020-4-16 8:00	FI	EURO	76	3237	8	72	26	2.224	2.158
3940	2020-4-17 8:00	FI	EURO	132	3369	3	75	27	2.226	2.267
3941	2020-4-18 8:00	FI	EURO	120	3489	7	82	28	2.350	2.340
3942	2020-4-19 8:00	FI	EURO	192	3681	8	90	29	2.445	2.427
3943	2020-4-20 8:00	FI	EURO	102	3783	4	94	30	2.485	2.488
3944	2020-4-21 8:00	FI	EURO	85	3868	4	98	31	2.534	2.844
3945	2020-4-22 8:00	FI	EURO	146	4014	43	141	32	3.513	3.218
3946	2020-4-23 8:00	FI	EURO	115	4129	8	149	33	3.609	3.712
3947	2020-4-24 8:00	FI	EURO	155	4284	23	172	34	4.015	3.884
3948	2020-4-25 8:00	FI	EURO	111	4395	5	177	35	4.027	4.066
3949	2020-4-26 8:00	FI	EURO	80	4475	9	186	36	4.156	4.112
3950	2020-4-27 8:00	FI	EURO	101	4576	4	190	37	4.152	4.140
3951	2020-4-28 8:00	FI	EURO	119	4695	3	193	38	4.111	4.154
3952	2020-4-29 8:00	FI	EURO	45	4740	6	199	39	4.198	4.169
3953	2020-4-30 8:00	FI	EURO	166	4906	7	206	40	4.199	4.207
3954	2020-5-1 8:00	FI	EURO	89	4995	5	211	41	4.224	4.246
3955	2020-5-2 8:00	FI	EURO	56	5051	7	218	42	4.316	4.270
3956										
3957	2020-1-24 8:00	FR	EURO	3	3	0	0			
3958	2020-1-25 8:00	FR	EURO	0	3	0	0			
3959	2020-1-26 8:00	FR	EURO	0	3	0	0			
3960	2020-1-27 8:00	FR	EURO	0	3	0	0			

Table S1 Daily confirmed cases and daily deaths of COVID-19 in 214 nations from WHO

ID	Date and time	Country or region	Continent	Confirmed patients	Cumulative Confirmed patients	Daily dead patients	Daily cumulative dead patients	Lag days	Daily CFR	3DMA CFR
3961	2020-1-28 8:00	FR	EURO	1	4	0	0			
3962	2020-1-29 8:00	FR	EURO	0	4	0	0			
3963	2020-1-30 8:00	FR	EURO	2	6	0	0			
3964	2020-1-31 8:00	FR	EURO	0	6	0	0			
3965	2020-2-1 8:00	FR	EURO	0	6	0	0			
3966	2020-2-2 8:00	FR	EURO	0	6	0	0			
3967	2020-2-3 8:00	FR	EURO	0	6	0	0			
3968	2020-2-4 8:00	FR	EURO	0	6	0	0			
3969	2020-2-5 8:00	FR	EURO	0	6	0	0			
3970	2020-2-6 8:00	FR	EURO	0	6	0	0			
3971	2020-2-7 8:00	FR	EURO	0	6	0	0			
3972	2020-2-8 8:00	FR	EURO	5	11	0	0			
3973	2020-2-9 8:00	FR	EURO	0	11	0	0			
3974	2020-2-10 8:00	FR	EURO	0	11	0	0			
3975	2020-2-11 8:00	FR	EURO	0	11	0	0			
3976	2020-2-12 8:00	FR	EURO	0	11	0	0			
3977	2020-2-13 8:00	FR	EURO	0	11	0	0			
3978	2020-2-14 8:00	FR	EURO	0	11	0	0			
3979	2020-2-15 8:00	FR	EURO	0	11	1	1	0	9.091	8.712
3980	2020-2-16 8:00	FR	EURO	1	12	0	1	1	8.333	8.586
3981	2020-2-17 8:00	FR	EURO	0	12	0	1	2	8.333	8.333
3982	2020-2-18 8:00	FR	EURO	0	12	0	1	3	8.333	8.333
3983	2020-2-19 8:00	FR	EURO	0	12	0	1	4	8.333	8.333
3984	2020-2-20 8:00	FR	EURO	0	12	0	1	5	8.333	8.333
3985	2020-2-21 8:00	FR	EURO	0	12	0	1	6	8.333	8.333
3986	2020-2-22 8:00	FR	EURO	0	12	0	1	7	8.333	8.333
3987	2020-2-23 8:00	FR	EURO	0	12	0	1	8	8.333	8.333
3988	2020-2-24 8:00	FR	EURO	0	12	0	1	9	8.333	8.333
3989	2020-2-25 8:00	FR	EURO	0	12	0	1	10	8.333	9.259
3990	2020-2-26 8:00	FR	EURO	6	18	1	2	11	11.111	10.185
3991	2020-2-27 8:00	FR	EURO	0	18	0	2	12	11.111	8.577
3992	2020-2-28 8:00	FR	EURO	39	57	0	2	13	3.509	5.540
3993	2020-2-29 8:00	FR	EURO	43	100	0	2	14	2.000	2.503
3994	2020-3-1 8:00	FR	EURO	0	100	0	2	15	2.000	1.865
3995	2020-3-2 8:00	FR	EURO	88	188	1	3	16	1.596	1.837
3996	2020-3-3 8:00	FR	EURO	21	209	1	4	17	1.914	1.643

Table S1 Daily confirmed cases and daily deaths of COVID-19 in 214 nations from WHO

ID	Date and time	Country or region	Continent	Confirmed patients	Cumulative Confirmed patients	Daily dead patients	Daily cumulative dead patients	Lag days	Daily CFR	3DMA CFR
3997	2020-3-4 8:00	FR	EURO	73	282	0	4	18	1.418	1.587
3998	2020-3-5 8:00	FR	EURO	138	420	2	6	19	1.429	1.438
3999	2020-3-6 8:00	FR	EURO	193	613	3	9	20	1.468	1.438
4000	2020-3-7 8:00	FR	EURO	93	706	1	10	21	1.416	1.529
4001	2020-3-8 8:00	FR	EURO	410	1116	9	19	22	1.703	1.753
4002	2020-3-9 8:00	FR	EURO	286	1402	11	30	23	2.140	1.901
4003	2020-3-10 8:00	FR	EURO	372	1774	3	33	24	1.860	2.038
4004	2020-3-11 8:00	FR	EURO	495	2269	15	48	25	2.115	2.027
4005	2020-3-12 8:00	FR	EURO	12	2281	0	48	26	2.104	2.130
4006	2020-3-13 8:00	FR	EURO	1359	3640	31	79	27	2.170	2.104
4007	2020-3-14 8:00	FR	EURO	829	4469	12	91	28	2.036	2.189
4008	2020-3-15 8:00	FR	EURO	911	5380	36	127	29	2.361	2.216
4009	2020-3-16 8:00	FR	EURO	1193	6573	21	148	30	2.252	2.300
4010	2020-3-17 8:00	FR	EURO	1079	7652	27	175	31	2.287	2.412
4011	2020-3-18 8:00	FR	EURO	1391	9043	69	244	32	2.698	2.802
4012	2020-3-19 8:00	FR	EURO	1834	10877	128	372	33	3.420	3.242
4013	2020-3-20 8:00	FR	EURO	1598	12475	78	450	34	3.607	3.653
4014	2020-3-21 8:00	FR	EURO	1821	14296	112	562	35	3.931	3.823
4015	2020-3-22 8:00	FR	EURO	0	14296	0	562	36	3.931	4.041
4016	2020-3-23 8:00	FR	EURO	1525	15821	112	674	37	4.260	4.192
4017	2020-3-24 8:00	FR	EURO	3794	19615	186	860	38	4.384	4.546
4018	2020-3-25 8:00	FR	EURO	2410	22025	240	1100	39	4.994	4.907
4019	2020-3-26 8:00	FR	EURO	2895	24920	231	1331	40	5.341	5.408
4020	2020-3-27 8:00	FR	EURO	3866	28786	364	1695	41	5.888	5.784
4021	2020-3-28 8:00	FR	EURO	3756	32542	297	1992	42	6.121	6.077
4022	2020-3-29 8:00	FR	EURO	4603	37145	319	2311	43	6.222	6.302
4023	2020-3-30 8:00	FR	EURO	2497	39642	291	2602	44	6.564	6.549
4024	2020-3-31 8:00	FR	EURO	4335	43977	415	3017	45	6.860	6.750
4025	2020-4-1 8:00	FR	EURO	7500	51477	497	3514	46	6.826	6.943
4026	2020-4-2 8:00	FR	EURO	4784	56261	505	4019	47	7.143	7.223
4027	2020-4-3 8:00	FR	EURO	2066	58327	471	4490	48	7.698	8.354
4028	2020-4-4 8:00	FR	EURO	5209	63536	2003	6493	49	10.219	9.685
4029	2020-4-5 8:00	FR	EURO	4221	67757	1053	7546	50	11.137	10.980
4030	2020-4-6 8:00	FR	EURO	1850	69607	518	8064	51	11.585	11.609
4031	2020-4-7 8:00	FR	EURO	3881	73488	832	8896	52	12.105	12.348
4032	2020-4-8 8:00	FR	EURO	3738	77226	1417	10313	53	13.354	12.948

Table S1 Daily confirmed cases and daily deaths of COVID-19 in 214 nations from WHO

ID	Date and time	Country or region	Continent	Confirmed patients	Cumulative Confirmed patients	Daily dead patients	Daily cumulative dead patients	Lag days	Daily CFR	3DMA CFR
4033	2020-4-9 8:00	FR	EURO	3869	81095	540	10853	54	13.383	13.674
4034	2020-4-10 8:00	FR	EURO	4256	85351	1339	12192	55	14.285	14.121
4035	2020-4-11 8:00	FR	EURO	4332	89683	987	13179	56	14.695	14.622
4036	2020-4-12 8:00	FR	EURO	3104	92787	635	13814	57	14.888	14.938
4037	2020-4-13 8:00	FR	EURO	1595	94382	560	14374	58	15.230	15.173
4038	2020-4-14 8:00	FR	EURO	2668	97050	572	14946	59	15.400	15.317
4039	2020-4-15 8:00	FR	EURO	5483	102533	762	15708	60	15.320	15.675
4040	2020-4-16 8:00	FR	EURO	2622	105155	1438	17146	61	16.305	16.078
4041	2020-4-17 8:00	FR	EURO	2623	107778	753	17899	62	16.607	16.721
4042	2020-4-18 8:00	FR	EURO	385	108163	760	18659	63	17.251	17.095
4043	2020-4-19 8:00	FR	EURO	2558	110721	635	19294	64	17.426	17.447
4044	2020-4-20 8:00	FR	EURO	742	111463	395	19689	65	17.664	17.638
4045	2020-4-21 8:00	FR	EURO	2050	113513	544	20233	66	17.824	17.788
4046	2020-4-22 8:00	FR	EURO	2638	116151	530	20763	67	17.876	17.921
4047	2020-4-23 8:00	FR	EURO	1810	117961	544	21307	68	18.063	18.063
4048	2020-4-24 8:00	FR	EURO	1622	119583	516	21823	69	18.249	18.206
4049	2020-4-25 8:00	FR	EURO	1755	121338	389	22212	70	18.306	18.311
4050	2020-4-26 8:00	FR	EURO	1537	122875	368	22580	71	18.376	18.398
4051	2020-4-27 8:00	FR	EURO	404	123279	241	22821	72	18.512	18.527
4052	2020-4-28 8:00	FR	EURO	1160	124439	440	23261	73	18.693	18.679
4053	2020-4-29 8:00	FR	EURO	1025	125464	366	23627	74	18.832	18.818
4054	2020-4-30 8:00	FR	EURO	1602	127066	427	24054	75	18.930	18.920
4055	2020-5-1 8:00	FR	EURO	1055	128121	288	24342	76	18.999	19.003
4056	2020-5-2 8:00	FR	EURO	601	128722	218	24560	77	19.080	19.040
4057										
4058	2020-3-7 8:00	GF	AMRO	5	5	0	0			
4059	2020-3-8 8:00	GF	AMRO	0	5	0	0			
4060	2020-3-9 8:00	GF	AMRO	0	5	0	0			
4061	2020-3-10 8:00	GF	AMRO	0	5	0	0			
4062	2020-3-11 8:00	GF	AMRO	0	5	0	0			
4063	2020-3-12 8:00	GF	AMRO	0	5	0	0			
4064	2020-3-13 8:00	GF	AMRO	1	6	0	0			
4065	2020-3-14 8:00	GF	AMRO	1	7	0	0			
4066	2020-3-15 8:00	GF	AMRO	0	7	0	0			
4067	2020-3-16 8:00	GF	AMRO	0	7	0	0			
4068	2020-3-17 8:00	GF	AMRO	0	7	0	0			

Table S1 Daily confirmed cases and daily deaths of COVID-19 in 214 nations from WHO

ID	Date and time	Country or region	Continent	Confirmed patients	Cumulative Confirmed patients	Daily dead patients	Daily cumulative dead patients	Lag days	Daily CFR	3DMA CFR
4069	2020-3-18 8:00	GF	AMRO	4	11	0	0			
4070	2020-3-19 8:00	GF	AMRO	4	15	0	0			
4071	2020-3-20 8:00	GF	AMRO	0	15	0	0			
4072	2020-3-21 8:00	GF	AMRO	0	15	0	0			
4073	2020-3-22 8:00	GF	AMRO	0	15	0	0			
4074	2020-3-23 8:00	GF	AMRO	3	18	0	0			
4075	2020-3-24 8:00	GF	AMRO	5	23	0	0			
4076	2020-3-25 8:00	GF	AMRO	0	23	0	0			
4077	2020-3-26 8:00	GF	AMRO	5	28	0	0			
4078	2020-3-27 8:00	GF	AMRO	0	28	0	0			
4079	2020-3-28 8:00	GF	AMRO	3	31	0	0			
4080	2020-3-29 8:00	GF	AMRO	0	31	0	0			
4081	2020-3-30 8:00	GF	AMRO	0	31	0	0			
4082	2020-3-31 8:00	GF	AMRO	12	43	0	0			
4083	2020-4-1 8:00	GF	AMRO	3	46	0	0			
4084	2020-4-2 8:00	GF	AMRO	5	51	0	0			
4085	2020-4-3 8:00	GF	AMRO	4	55	0	0			
4086	2020-4-4 8:00	GF	AMRO	2	57	0	0			
4087	2020-4-5 8:00	GF	AMRO	5	62	0	0			
4088	2020-4-6 8:00	GF	AMRO	4	66	0	0			
4089	2020-4-7 8:00	GF	AMRO	2	68	0	0			
4090	2020-4-8 8:00	GF	AMRO	4	72	0	0			
4091	2020-4-9 8:00	GF	AMRO	5	77	0	0			
4092	2020-4-10 8:00	GF	AMRO	6	83	0	0			
4093	2020-4-11 8:00	GF	AMRO	1	84	0	0			
4094	2020-4-12 8:00	GF	AMRO	2	86	0	0			
4095	2020-4-13 8:00	GF	AMRO	0	86	0	0			
4096	2020-4-14 8:00	GF	AMRO	2	88	0	0			
4097	2020-4-15 8:00	GF	AMRO	0	88	0	0			
4098	2020-4-16 8:00	GF	AMRO	7	95	0	0			
4099	2020-4-17 8:00	GF	AMRO	1	96	0	0			
4100	2020-4-18 8:00	GF	AMRO	0	96	0	0			
4101	2020-4-19 8:00	GF	AMRO	0	96	0	0			
4102	2020-4-20 8:00	GF	AMRO	0	96	0	0			
4103	2020-4-21 8:00	GF	AMRO	1	97	1	1	0	1.031	1.031
4104	2020-4-22 8:00	GF	AMRO	0	97	0	1	1	1.031	1.031

Table S1 Daily confirmed cases and daily deaths of COVID-19 in 214 nations from WHO

ID	Date and time	Country or region	Continent	Confirmed patients	Cumulative Confirmed patients	Daily dead patients	Daily cumulative dead patients	Lag days	Daily CFR	3DMA CFR
4105	2020-4-23 8:00	GF	AMRO	0	97	0	1	2	1.031	0.999
4106	2020-4-24 8:00	GF	AMRO	10	107	0	1	3	0.935	0.961
4107	2020-4-25 8:00	GF	AMRO	2	109	0	1	4	0.917	0.923
4108	2020-4-26 8:00	GF	AMRO	0	109	0	1	5	0.917	0.917
4109	2020-4-27 8:00	GF	AMRO	0	109	0	1	6	0.917	0.912
4110	2020-4-28 8:00	GF	AMRO	2	111	0	1	7	0.901	0.875
4111	2020-4-29 8:00	GF	AMRO	13	124	0	1	8	0.806	0.836
4112	2020-4-30 8:00	GF	AMRO	1	125	0	1	9	0.800	0.800
4113	2020-5-1 8:00	GF	AMRO	1	126	0	1	10	0.794	0.792
4114	2020-5-2 8:00	GF	AMRO	2	128	0	1	0	0.781	0.787
4115										
4116	2020-3-12 8:00	PF	WPRO	1	1	0	0			
4117	2020-3-13 8:00	PF	WPRO	0	1	0	0			
4118	2020-3-14 8:00	PF	WPRO	0	1	0	0			
4119	2020-3-15 8:00	PF	WPRO	2	3	0	0			
4120	2020-3-16 8:00	PF	WPRO	0	3	0	0			
4121	2020-3-17 8:00	PF	WPRO	0	3	0	0			
4122	2020-3-18 8:00	PF	WPRO	0	3	0	0			
4123	2020-3-19 8:00	PF	WPRO	8	11	0	0			
4124	2020-3-20 8:00	PF	WPRO	0	11	0	0			
4125	2020-3-21 8:00	PF	WPRO	4	15	0	0			
4126	2020-3-22 8:00	PF	WPRO	0	15	0	0			
4127	2020-3-23 8:00	PF	WPRO	3	18	0	0			
4128	2020-3-24 8:00	PF	WPRO	5	23	0	0			
4129	2020-3-25 8:00	PF	WPRO	2	25	0	0			
4130	2020-3-26 8:00	PF	WPRO	0	25	0	0			
4131	2020-3-27 8:00	PF	WPRO	5	30	0	0			
4132	2020-3-28 8:00	PF	WPRO	0	30	0	0			
4133	2020-3-29 8:00	PF	WPRO	4	34	0	0			
4134	2020-3-30 8:00	PF	WPRO	1	35	0	0			
4135	2020-3-31 8:00	PF	WPRO	1	36	0	0			
4136	2020-4-1 8:00	PF	WPRO	1	37	0	0			
4137	2020-4-2 8:00	PF	WPRO	0	37	0	0			
4138	2020-4-3 8:00	PF	WPRO	0	37	0	0			
4139	2020-4-4 8:00	PF	WPRO	2	39	0	0			
4140	2020-4-5 8:00	PF	WPRO	1	40	0	0			

Table S1 Daily confirmed cases and daily deaths of COVID-19 in 214 nations from WHO

ID	Date and time	Country or region	Continent	Confirmed patients	Cumulative Confirmed patients	Daily dead patients	Daily cumulative dead patients	Lag days	Daily CFR	3DMA CFR
4141	2020-4-6 8:00	PF	WPRO	1	41	0	0			
4142	2020-4-7 8:00	PF	WPRO	1	42	0	0			
4143	2020-4-8 8:00	PF	WPRO	5	47	0	0			
4144	2020-4-9 8:00	PF	WPRO	4	51	0	0			
4145	2020-4-10 8:00	PF	WPRO	0	51	0	0			
4146	2020-4-11 8:00	PF	WPRO	0	51	0	0			
4147	2020-4-12 8:00	PF	WPRO	0	51	0	0			
4148	2020-4-13 8:00	PF	WPRO	2	53	0	0			
4149	2020-4-14 8:00	PF	WPRO	2	55	0	0			
4150	2020-4-15 8:00	PF	WPRO	0	55	0	0			
4151	2020-4-16 8:00	PF	WPRO	0	55	0	0			
4152	2020-4-17 8:00	PF	WPRO	0	55	0	0			
4153	2020-4-18 8:00	PF	WPRO	0	55	0	0			
4154	2020-4-19 8:00	PF	WPRO	0	55	0	0			
4155	2020-4-20 8:00	PF	WPRO	0	55	0	0			
4156	2020-4-21 8:00	PF	WPRO	1	56	0	0			
4157	2020-4-22 8:00	PF	WPRO	1	57	0	0			
4158	2020-4-23 8:00	PF	WPRO	0	57	0	0			
4159	2020-4-24 8:00	PF	WPRO	0	57	0	0			
4160	2020-4-25 8:00	PF	WPRO	0	57	0	0			
4161	2020-4-26 8:00	PF	WPRO	0	57	0	0			
4162	2020-4-27 8:00	PF	WPRO	0	57	0	0			
4163	2020-4-28 8:00	PF	WPRO	1	58	0	0			
4164	2020-4-29 8:00	PF	WPRO	0	58	0	0			
4165	2020-4-30 8:00	PF	WPRO	0	58	0	0			
4166	2020-5-1 8:00	PF	WPRO	0	58	0	0			
4167	2020-5-2 8:00	PF	WPRO	0	58	0	0			
4168										
4169	2020-3-14 8:00	GA	AFRO	1	1	0	0			
4170	2020-3-15 8:00	GA	AFRO	0	1	0	0			
4171	2020-3-16 8:00	GA	AFRO	0	1	0	0			
4172	2020-3-17 8:00	GA	AFRO	0	1	0	0			
4173	2020-3-18 8:00	GA	AFRO	2	3	0	0			
4174	2020-3-19 8:00	GA	AFRO	0	3	0	0			
4175	2020-3-20 8:00	GA	AFRO	0	3	1	1	0	33.333	33.333
4176	2020-3-21 8:00	GA	AFRO	0	3	0	1	1	33.333	27.778

Table S1 Daily confirmed cases and daily deaths of COVID-19 in 214 nations from WHO

ID	Date and time	Country or region	Continent	Confirmed patients	Cumulative Confirmed patients	Daily dead patients	Daily cumulative dead patients	Lag days	Daily CFR	3DMA CFR
4177	2020-3-22 8:00	GA	AFRO	3	6	0	1	2	16.667	22.222
4178	2020-3-23 8:00	GA	AFRO	0	6	0	1	3	16.667	16.667
4179	2020-3-24 8:00	GA	AFRO	0	6	0	1	4	16.667	16.667
4180	2020-3-25 8:00	GA	AFRO	0	6	0	1	5	16.667	16.667
4181	2020-3-26 8:00	GA	AFRO	0	6	0	1	6	16.667	16.667
4182	2020-3-27 8:00	GA	AFRO	0	6	0	1	7	16.667	15.873
4183	2020-3-28 8:00	GA	AFRO	1	7	0	1	8	14.286	15.079
4184	2020-3-29 8:00	GA	AFRO	0	7	0	1	9	14.286	14.286
4185	2020-3-30 8:00	GA	AFRO	0	7	0	1	10	14.286	14.286
4186	2020-3-31 8:00	GA	AFRO	0	7	0	1	11	14.286	14.286
4187	2020-4-1 8:00	GA	AFRO	0	7	0	1	12	14.286	14.286
4188	2020-4-2 8:00	GA	AFRO	0	7	0	1	13	14.286	11.376
4189	2020-4-3 8:00	GA	AFRO	11	18	0	1	14	5.556	8.201
4190	2020-4-4 8:00	GA	AFRO	3	21	0	1	15	4.762	5.026
4191	2020-4-5 8:00	GA	AFRO	0	21	0	1	16	4.762	4.762
4192	2020-4-6 8:00	GA	AFRO	0	21	0	1	17	4.762	4.762
4193	2020-4-7 8:00	GA	AFRO	0	21	0	1	18	4.762	4.563
4194	2020-4-8 8:00	GA	AFRO	3	24	0	1	19	4.167	4.365
4195	2020-4-9 8:00	GA	AFRO	0	24	0	1	20	4.167	3.535
4196	2020-4-10 8:00	GA	AFRO	20	44	0	1	21	2.273	2.904
4197	2020-4-11 8:00	GA	AFRO	0	44	0	1	22	2.273	2.195
4198	2020-4-12 8:00	GA	AFRO	5	49	0	1	23	2.041	2.023
4199	2020-4-13 8:00	GA	AFRO	8	57	0	1	24	1.754	1.850
4200	2020-4-14 8:00	GA	AFRO	0	57	0	1	25	1.754	1.586
4201	2020-4-15 8:00	GA	AFRO	23	80	0	1	26	1.250	1.385
4202	2020-4-16 8:00	GA	AFRO	7	87	0	1	27	1.149	1.151
4203	2020-4-17 8:00	GA	AFRO	8	95	0	1	28	1.053	1.085
4204	2020-4-18 8:00	GA	AFRO	0	95	0	1	29	1.053	1.010
4205	2020-4-19 8:00	GA	AFRO	13	108	0	1	30	0.926	0.965
4206	2020-4-20 8:00	GA	AFRO	1	109	0	1	31	0.917	0.892
4207	2020-4-21 8:00	GA	AFRO	11	120	0	1	32	0.833	0.797
4208	2020-4-22 8:00	GA	AFRO	36	156	0	1	33	0.641	0.692
4209	2020-4-23 8:00	GA	AFRO	10	166	0	1	34	0.602	0.816
4210	2020-4-24 8:00	GA	AFRO	0	166	1	2	35	1.205	1.184
4211	2020-4-25 8:00	GA	AFRO	6	172	1	3	36	1.744	1.551
4212	2020-4-26 8:00	GA	AFRO	4	176	0	3	37	1.705	1.718

Table S1 Daily confirmed cases and daily deaths of COVID-19 in 214 nations from WHO

ID	Date and time	Country or region	Continent	Confirmed patients	Cumulative Confirmed patients	Daily dead patients	Daily cumulative dead patients	Lag days	Daily CFR	3DMA CFR
4213	2020-4-27 8:00	GA	AFRO	0	176	0	3	38	1.705	1.705
4214	2020-4-28 8:00	GA	AFRO	0	176	0	3	39	1.705	1.557
4215	2020-4-29 8:00	GA	AFRO	62	238	0	3	40	1.261	1.351
4216	2020-4-30 8:00	GA	AFRO	38	276	0	3	41	1.087	1.145
4217	2020-5-1 8:00	GA	AFRO	0	276	0	3	42	1.087	1.158
4218	2020-5-2 8:00	GA	AFRO	32	308	1	4	43	1.299	1.193
4219										
4220	2020-3-18 8:00	GM	AFRO	1	1	0	0			
4221	2020-3-19 8:00	GM	AFRO	0	1	0	0			
4222	2020-3-20 8:00	GM	AFRO	0	1	0	0			
4223	2020-3-21 8:00	GM	AFRO	0	1	0	0			
4224	2020-3-22 8:00	GM	AFRO	0	1	0	0			
4225	2020-3-23 8:00	GM	AFRO	0	1	0	0			
4226	2020-3-24 8:00	GM	AFRO	0	1	0	0			
4227	2020-3-25 8:00	GM	AFRO	1	2	0	0			
4228	2020-3-26 8:00	GM	AFRO	0	2	0	0			
4229	2020-3-27 8:00	GM	AFRO	0	2	0	0			
4230	2020-3-28 8:00	GM	AFRO	0	2	1	1	0	50.000	41.667
4231	2020-3-29 8:00	GM	AFRO	1	3	0	1	1	33.333	38.889
4232	2020-3-30 8:00	GM	AFRO	0	3	0	1	2	33.333	33.333
4233	2020-3-31 8:00	GM	AFRO	0	3	0	1	3	33.333	33.333
4234	2020-4-1 8:00	GM	AFRO	0	3	0	1	4	33.333	33.333
4235	2020-4-2 8:00	GM	AFRO	0	3	0	1	5	33.333	30.556
4236	2020-4-3 8:00	GM	AFRO	1	4	0	1	6	25.000	27.778
4237	2020-4-4 8:00	GM	AFRO	0	4	0	1	7	25.000	25.000
4238	2020-4-5 8:00	GM	AFRO	0	4	0	1	8	25.000	25.000
4239	2020-4-6 8:00	GM	AFRO	0	4	0	1	9	25.000	25.000
4240	2020-4-7 8:00	GM	AFRO	0	4	0	1	10	25.000	25.000
4241	2020-4-8 8:00	GM	AFRO	0	4	0	1	11	25.000	25.000
4242	2020-4-9 8:00	GM	AFRO	0	4	0	1	12	25.000	25.000
4243	2020-4-10 8:00	GM	AFRO	0	4	0	1	13	25.000	25.000
4244	2020-4-11 8:00	GM	AFRO	0	4	0	1	14	25.000	20.370
4245	2020-4-12 8:00	GM	AFRO	5	9	0	1	15	11.111	15.741
4246	2020-4-13 8:00	GM	AFRO	0	9	0	1	16	11.111	11.111
4247	2020-4-14 8:00	GM	AFRO	0	9	0	1	17	11.111	11.111
4248	2020-4-15 8:00	GM	AFRO	0	9	0	1	18	11.111	11.111

Table S1 Daily confirmed cases and daily deaths of COVID-19 in 214 nations from WHO

ID	Date and time	Country or region	Continent	Confirmed patients	Cumulative Confirmed patients	Daily dead patients	Daily cumulative dead patients	Lag days	Daily CFR	3DMA CFR
4249	2020-4-16 8:00	GM	AFRO	0	9	0	1	19	11.111	11.111
4250	2020-4-17 8:00	GM	AFRO	0	9	0	1	20	11.111	11.111
4251	2020-4-18 8:00	GM	AFRO	0	9	0	1	21	11.111	11.111
4252	2020-4-19 8:00	GM	AFRO	0	9	0	1	22	11.111	11.111
4253	2020-4-20 8:00	GM	AFRO	0	9	0	1	23	11.111	10.741
4254	2020-4-21 8:00	GM	AFRO	1	10	0	1	24	10.000	10.370
4255	2020-4-22 8:00	GM	AFRO	0	10	0	1	25	10.000	10.000
4256	2020-4-23 8:00	GM	AFRO	0	10	0	1	26	10.000	10.000
4257	2020-4-24 8:00	GM	AFRO	0	10	0	1	27	10.000	10.000
4258	2020-4-25 8:00	GM	AFRO	0	10	0	1	28	10.000	10.000
4259	2020-4-26 8:00	GM	AFRO	0	10	0	1	29	10.000	10.000
4260	2020-4-27 8:00	GM	AFRO	0	10	0	1	30	10.000	10.000
4261	2020-4-28 8:00	GM	AFRO	0	10	0	1	31	10.000	10.000
4262	2020-4-29 8:00	GM	AFRO	0	10	0	1	32	10.000	9.697
4263	2020-4-30 8:00	GM	AFRO	1	11	0	1	33	9.091	9.141
4264	2020-5-1 8:00	GM	AFRO	1	12	0	1	34	8.333	8.586
4265	2020-5-2 8:00	GM	AFRO	0	12	0	1	35	8.333	8.333
4266										
4267	2020-2-26 8:00	GE	EURO	1	1	0	0			
4268	2020-2-27 8:00	GE	EURO	0	1	0	0			
4269	2020-2-28 8:00	GE	EURO	1	2	0	0			
4270	2020-2-29 8:00	GE	EURO	1	3	0	0			
4271	2020-3-1 8:00	GE	EURO	0	3	0	0			
4272	2020-3-2 8:00	GE	EURO	0	3	0	0			
4273	2020-3-3 8:00	GE	EURO	0	3	0	0			
4274	2020-3-4 8:00	GE	EURO	0	3	0	0			
4275	2020-3-5 8:00	GE	EURO	6	9	0	0			
4276	2020-3-6 8:00	GE	EURO	0	9	0	0			
4277	2020-3-7 8:00	GE	EURO	3	12	0	0			
4278	2020-3-8 8:00	GE	EURO	1	13	0	0			
4279	2020-3-9 8:00	GE	EURO	2	15	0	0			
4280	2020-3-10 8:00	GE	EURO	8	23	0	0			
4281	2020-3-11 8:00	GE	EURO	0	23	0	0			
4282	2020-3-12 8:00	GE	EURO	2	25	0	0			
4283	2020-3-13 8:00	GE	EURO	0	25	0	0			
4284	2020-3-14 8:00	GE	EURO	5	30	0	0			

Table S1 Daily confirmed cases and daily deaths of COVID-19 in 214 nations from WHO

ID	Date and time	Country or region	Continent	Confirmed patients	Cumulative Confirmed patients	Daily dead patients	Daily cumulative dead patients	Lag days	Daily CFR	3DMA CFR
4285	2020-3-15 8:00	GE	EURO	3	33	0	0			
4286	2020-3-16 8:00	GE	EURO	0	33	0	0			
4287	2020-3-17 8:00	GE	EURO	1	34	0	0			
4288	2020-3-18 8:00	GE	EURO	0	34	0	0			
4289	2020-3-19 8:00	GE	EURO	4	38	0	0			
4290	2020-3-20 8:00	GE	EURO	5	43	0	0			
4291	2020-3-21 8:00	GE	EURO	6	49	0	0			
4292	2020-3-22 8:00	GE	EURO	0	49	0	0			
4293	2020-3-23 8:00	GE	EURO	5	54	0	0			
4294	2020-3-24 8:00	GE	EURO	13	67	0	0			
4295	2020-3-25 8:00	GE	EURO	6	73	0	0			
4296	2020-3-26 8:00	GE	EURO	4	77	0	0			
4297	2020-3-27 8:00	GE	EURO	4	81	0	0			
4298	2020-3-28 8:00	GE	EURO	4	85	0	0			
4299	2020-3-29 8:00	GE	EURO	5	90	0	0			
4300	2020-3-30 8:00	GE	EURO	8	98	0	0			
4301	2020-3-31 8:00	GE	EURO	5	103	0	0			
4302	2020-4-1 8:00	GE	EURO	12	115	0	0			
4303	2020-4-2 8:00	GE	EURO	6	121	0	0			
4304	2020-4-3 8:00	GE	EURO	27	148	0	0			
4305	2020-4-4 8:00	GE	EURO	9	157	0	0			
4306	2020-4-5 8:00	GE	EURO	13	170	1	1	0	0.588	0.826
4307	2020-4-6 8:00	GE	EURO	18	188	1	2	1	1.064	0.893
4308	2020-4-7 8:00	GE	EURO	7	195	0	2	2	1.026	1.177
4309	2020-4-8 8:00	GE	EURO	13	208	1	3	3	1.442	1.290
4310	2020-4-9 8:00	GE	EURO	6	214	0	3	4	1.402	1.383
4311	2020-4-10 8:00	GE	EURO	16	230	0	3	5	1.304	1.331
4312	2020-4-11 8:00	GE	EURO	3	233	0	3	6	1.288	1.261
4313	2020-4-12 8:00	GE	EURO	19	252	0	3	7	1.190	1.202
4314	2020-4-13 8:00	GE	EURO	14	266	0	3	8	1.128	1.111
4315	2020-4-14 8:00	GE	EURO	30	296	0	3	9	1.014	1.041
4316	2020-4-15 8:00	GE	EURO	10	306	0	3	10	0.980	0.962
4317	2020-4-16 8:00	GE	EURO	30	336	0	3	11	0.893	0.895
4318	2020-4-17 8:00	GE	EURO	34	370	0	3	12	0.811	0.828
4319	2020-4-18 8:00	GE	EURO	15	385	0	3	13	0.779	0.868
4320	2020-4-19 8:00	GE	EURO	9	394	1	4	14	1.015	0.932

Table S1 Daily confirmed cases and daily deaths of COVID-19 in 214 nations from WHO

ID	Date and time	Country or region	Continent	Confirmed patients	Cumulative Confirmed patients	Daily dead patients	Daily cumulative dead patients	Lag days	Daily CFR	3DMA CFR
4321	2020-4-20 8:00	GE	EURO	5	399	0	4	15	1.003	0.999
4322	2020-4-21 8:00	GE	EURO	9	408	0	4	16	0.980	1.066
4323	2020-4-22 8:00	GE	EURO	3	411	1	5	17	1.217	1.129
4324	2020-4-23 8:00	GE	EURO	9	420	0	5	18	1.190	1.189
4325	2020-4-24 8:00	GE	EURO	11	431	0	5	19	1.160	1.149
4326	2020-4-25 8:00	GE	EURO	25	456	0	5	20	1.096	1.096
4327	2020-4-26 8:00	GE	EURO	29	485	0	5	21	1.031	1.112
4328	2020-4-27 8:00	GE	EURO	11	496	1	6	22	1.210	1.138
4329	2020-4-28 8:00	GE	EURO	15	511	0	6	23	1.174	1.181
4330	2020-4-29 8:00	GE	EURO	6	517	0	6	24	1.161	1.149
4331	2020-4-30 8:00	GE	EURO	22	539	0	6	25	1.113	1.111
4332	2020-5-1 8:00	GE	EURO	27	566	0	6	26	1.060	1.183
4333	2020-5-2 8:00	GE	EURO	16	582	2	8	27	1.375	1.217
4334										
4335	2020-1-28 8:00	DE	EURO	4	4	0	0			
4336	2020-1-29 8:00	DE	EURO	0	4	0	0			
4337	2020-1-30 8:00	DE	EURO	0	4	0	0			
4338	2020-1-31 8:00	DE	EURO	3	7	0	0			
4339	2020-2-1 8:00	DE	EURO	1	8	0	0			
4340	2020-2-2 8:00	DE	EURO	2	10	0	0			
4341	2020-2-3 8:00	DE	EURO	0	10	0	0			
4342	2020-2-4 8:00	DE	EURO	2	12	0	0			
4343	2020-2-5 8:00	DE	EURO	0	12	0	0			
4344	2020-2-6 8:00	DE	EURO	1	13	0	0			
4345	2020-2-7 8:00	DE	EURO	1	14	0	0			
4346	2020-2-8 8:00	DE	EURO	0	14	0	0			
4347	2020-2-9 8:00	DE	EURO	0	14	0	0			
4348	2020-2-10 8:00	DE	EURO	0	14	0	0			
4349	2020-2-11 8:00	DE	EURO	2	16	0	0			
4350	2020-2-12 8:00	DE	EURO	0	16	0	0			
4351	2020-2-13 8:00	DE	EURO	0	16	0	0			
4352	2020-2-14 8:00	DE	EURO	0	16	0	0			
4353	2020-2-15 8:00	DE	EURO	0	16	0	0			
4354	2020-2-16 8:00	DE	EURO	0	16	0	0			
4355	2020-2-17 8:00	DE	EURO	0	16	0	0			
4356	2020-2-18 8:00	DE	EURO	0	16	0	0			

Table S1 Daily confirmed cases and daily deaths of COVID-19 in 214 nations from WHO

ID	Date and time	Country or region	Continent	Confirmed patients	Cumulative Confirmed patients	Daily dead patients	Daily cumulative dead patients	Lag days	Daily CFR	3DMA CFR
4357	2020-2-19 8:00	DE	EURO	0	16	0	0			
4358	2020-2-20 8:00	DE	EURO	0	16	0	0			
4359	2020-2-21 8:00	DE	EURO	0	16	0	0			
4360	2020-2-22 8:00	DE	EURO	0	16	0	0			
4361	2020-2-23 8:00	DE	EURO	0	16	0	0			
4362	2020-2-24 8:00	DE	EURO	0	16	0	0			
4363	2020-2-25 8:00	DE	EURO	2	18	0	0			
4364	2020-2-26 8:00	DE	EURO	3	21	0	0			
4365	2020-2-27 8:00	DE	EURO	5	26	0	0			
4366	2020-2-28 8:00	DE	EURO	31	57	0	0			
4367	2020-2-29 8:00	DE	EURO	0	57	0	0			
4368	2020-3-1 8:00	DE	EURO	72	129	0	0			
4369	2020-3-2 8:00	DE	EURO	29	158	0	0			
4370	2020-3-3 8:00	DE	EURO	38	196	0	0			
4371	2020-3-4 8:00	DE	EURO	66	262	0	0			
4372	2020-3-5 8:00	DE	EURO	138	400	0	0			
4373	2020-3-6 8:00	DE	EURO	239	639	0	0			
4374	2020-3-7 8:00	DE	EURO	156	795	0	0			
4375	2020-3-8 8:00	DE	EURO	107	902	0	0			
4376	2020-3-9 8:00	DE	EURO	237	1139	2	2	0	0.176	0.165
4377	2020-3-10 8:00	DE	EURO	157	1296	0	2	1	0.154	0.174
4378	2020-3-11 8:00	DE	EURO	271	1567	1	3	2	0.191	0.179
4379	2020-3-12 8:00	DE	EURO	0	1567	0	3	3	0.191	0.172
4380	2020-3-13 8:00	DE	EURO	693	2260	0	3	4	0.133	0.178
4381	2020-3-14 8:00	DE	EURO	1535	3795	5	8	5	0.211	0.197
4382	2020-3-15 8:00	DE	EURO	1043	4838	4	12	6	0.248	0.219
4383	2020-3-16 8:00	DE	EURO	1174	6012	0	12	7	0.200	0.205
4384	2020-3-17 8:00	DE	EURO	1144	7156	0	12	8	0.168	0.171
4385	2020-3-18 8:00	DE	EURO	1042	8198	0	12	9	0.146	0.165
4386	2020-3-19 8:00	DE	EURO	2801	10999	8	20	10	0.182	0.191
4387	2020-3-20 8:00	DE	EURO	7324	18323	25	45	11	0.246	0.247
4388	2020-3-21 8:00	DE	EURO	3140	21463	22	67	12	0.312	0.290
4389	2020-3-22 8:00	DE	EURO	0	21463	0	67	13	0.312	0.335
4390	2020-3-23 8:00	DE	EURO	3311	24774	27	94	14	0.379	0.374
4391	2020-3-24 8:00	DE	EURO	4438	29212	32	126	15	0.431	0.428
4392	2020-3-25 8:00	DE	EURO	2342	31554	23	149	16	0.472	0.482

Table S1 Daily confirmed cases and daily deaths of COVID-19 in 214 nations from WHO

ID	Date and time	Country or region	Continent	Confirmed patients	Cumulative Confirmed patients	Daily dead patients	Daily cumulative dead patients	Lag days	Daily CFR	3DMA CFR
4393	2020-3-26 8:00	DE	EURO	4954	36508	49	198	17	0.542	0.538
4394	2020-3-27 8:00	DE	EURO	5780	42288	55	253	18	0.598	0.603
4395	2020-3-28 8:00	DE	EURO	6294	48582	72	325	19	0.669	0.669
4396	2020-3-29 8:00	DE	EURO	3965	52547	64	389	20	0.740	0.734
4397	2020-3-30 8:00	DE	EURO	4751	57298	66	455	21	0.794	0.825
4398	2020-3-31 8:00	DE	EURO	4615	61913	128	583	22	0.942	0.941
4399	2020-4-1 8:00	DE	EURO	5453	67366	149	732	23	1.087	1.071
4400	2020-4-2 8:00	DE	EURO	6156	73522	140	872	24	1.186	1.183
4401	2020-4-3 8:00	DE	EURO	6174	79696	145	1017	25	1.276	1.271
4402	2020-4-4 8:00	DE	EURO	6082	85778	141	1158	26	1.350	1.363
4403	2020-4-5 8:00	DE	EURO	5936	91714	184	1342	27	1.463	1.439
4404	2020-4-6 8:00	DE	EURO	3677	95391	92	1434	28	1.503	1.529
4405	2020-4-7 8:00	DE	EURO	3834	99225	173	1607	29	1.620	1.642
4406	2020-4-8 8:00	DE	EURO	4003	103228	254	1861	30	1.803	1.790
4407	2020-4-9 8:00	DE	EURO	4974	108202	246	2107	31	1.947	1.947
4408	2020-4-10 8:00	DE	EURO	5323	113525	266	2373	32	2.090	2.067
4409	2020-4-11 8:00	DE	EURO	4133	117658	171	2544	33	2.162	2.157
4410	2020-4-12 8:00	DE	EURO	2821	120479	129	2673	34	2.219	2.219
4411	2020-4-13 8:00	DE	EURO	2537	123016	126	2799	35	2.275	2.289
4412	2020-4-14 8:00	DE	EURO	2082	125098	170	2969	36	2.373	2.400
4413	2020-4-15 8:00	DE	EURO	2486	127584	285	3254	37	2.550	2.553
4414	2020-4-16 8:00	DE	EURO	2866	130450	315	3569	38	2.736	2.726
4415	2020-4-17 8:00	DE	EURO	3380	133830	299	3868	39	2.890	2.872
4416	2020-4-18 8:00	DE	EURO	3609	137439	242	4110	40	2.990	2.983
4417	2020-4-19 8:00	DE	EURO	2458	139897	184	4294	41	3.069	3.056
4418	2020-4-20 8:00	DE	EURO	1775	141672	110	4404	42	3.109	3.128
4419	2020-4-21 8:00	DE	EURO	1785	143457	194	4598	43	3.205	3.221
4420	2020-4-22 8:00	DE	EURO	2237	145694	281	4879	44	3.349	3.332
4421	2020-4-23 8:00	DE	EURO	2352	148046	215	5094	45	3.441	3.443
4422	2020-4-24 8:00	DE	EURO	2337	150383	227	5321	46	3.538	3.529
4423	2020-4-25 8:00	DE	EURO	2055	152438	179	5500	47	3.608	3.602
4424	2020-4-26 8:00	DE	EURO	1737	154175	140	5640	48	3.658	3.657
4425	2020-4-27 8:00	DE	EURO	1018	155193	110	5750	49	3.705	3.715
4426	2020-4-28 8:00	DE	EURO	1144	156337	163	5913	50	3.782	3.789
4427	2020-4-29 8:00	DE	EURO	1304	157641	202	6115	51	3.879	3.871
4428	2020-4-30 8:00	DE	EURO	1478	159119	173	6288	52	3.952	3.928

Table S1 Daily confirmed cases and daily deaths of COVID-19 in 214 nations from WHO

ID	Date and time	Country or region	Continent	Confirmed patients	Cumulative Confirmed patients	Daily dead patients	Daily cumulative dead patients	Lag days	Daily CFR	3DMA CFR
4429	2020-5-1 8:00	DE	EURO	0	159119	0	6288	53	3.952	3.990
4430	2020-5-2 8:00	DE	EURO	2584	161703	287	6575	54	4.066	4.009
4431										
4432	2020-3-14 8:00	GH	AFRO	1	1	0	0			
4433	2020-3-15 8:00	GH	AFRO	1	2	0	0			
4434	2020-3-16 8:00	GH	AFRO	0	2	0	0			
4435	2020-3-17 8:00	GH	AFRO	4	6	0	0			
4436	2020-3-18 8:00	GH	AFRO	3	9	0	0			
4437	2020-3-19 8:00	GH	AFRO	2	11	0	0			
4438	2020-3-20 8:00	GH	AFRO	5	16	0	0			
4439	2020-3-21 8:00	GH	AFRO	3	19	0	0			
4440	2020-3-22 8:00	GH	AFRO	0	19	0	0			
4441	2020-3-23 8:00	GH	AFRO	5	24	0	0			
4442	2020-3-24 8:00	GH	AFRO	3	27	2	2	0	7.407	5.590
4443	2020-3-25 8:00	GH	AFRO	26	53	0	2	1	3.774	4.232
4444	2020-3-26 8:00	GH	AFRO	79	132	0	2	2	1.515	2.498
4445	2020-3-27 8:00	GH	AFRO	4	136	1	3	3	2.206	2.214
4446	2020-3-28 8:00	GH	AFRO	1	137	1	4	4	2.920	2.682
4447	2020-3-29 8:00	GH	AFRO	0	137	0	4	5	2.920	2.824
4448	2020-3-30 8:00	GH	AFRO	15	152	0	4	6	2.632	2.728
4449	2020-3-31 8:00	GH	AFRO	0	152	0	4	7	2.632	2.583
4450	2020-4-1 8:00	GH	AFRO	9	161	0	4	8	2.484	2.389
4451	2020-4-2 8:00	GH	AFRO	34	195	0	4	9	2.051	2.166
4452	2020-4-3 8:00	GH	AFRO	9	204	0	4	10	1.961	1.988
4453	2020-4-4 8:00	GH	AFRO	1	205	0	4	11	1.951	1.954
4454	2020-4-5 8:00	GH	AFRO	0	205	0	4	12	1.951	2.080
4455	2020-4-6 8:00	GH	AFRO	9	214	1	5	13	2.336	2.208
4456	2020-4-7 8:00	GH	AFRO	0	214	0	5	14	2.336	2.138
4457	2020-4-8 8:00	GH	AFRO	73	287	0	5	15	1.742	1.999
4458	2020-4-9 8:00	GH	AFRO	26	313	1	6	16	1.917	1.859
4459	2020-4-10 8:00	GH	AFRO	0	313	0	6	17	1.917	1.807
4460	2020-4-11 8:00	GH	AFRO	65	378	0	6	18	1.587	1.822
4461	2020-4-12 8:00	GH	AFRO	30	408	2	8	19	1.961	1.654
4462	2020-4-13 8:00	GH	AFRO	158	566	0	8	20	1.413	1.596
4463	2020-4-14 8:00	GH	AFRO	0	566	0	8	21	1.413	1.362
4464	2020-4-15 8:00	GH	AFRO	70	636	0	8	22	1.258	1.310

Table S1 Daily confirmed cases and daily deaths of COVID-19 in 214 nations from WHO

ID	Date and time	Country or region	Continent	Confirmed patients	Cumulative Confirmed patients	Daily dead patients	Daily cumulative dead patients	Lag days	Daily CFR	3DMA CFR
4465	2020-4-16 8:00	GH	AFRO	0	636	0	8	23	1.258	1.255
4466	2020-4-17 8:00	GH	AFRO	5	641	0	8	24	1.248	1.251
4467	2020-4-18 8:00	GH	AFRO	0	641	0	8	25	1.248	1.192
4468	2020-4-19 8:00	GH	AFRO	193	834	1	9	26	1.079	1.064
4469	2020-4-20 8:00	GH	AFRO	208	1042	0	9	27	0.864	0.936
4470	2020-4-21 8:00	GH	AFRO	0	1042	0	9	28	0.864	0.864
4471	2020-4-22 8:00	GH	AFRO	0	1042	0	9	29	0.864	0.836
4472	2020-4-23 8:00	GH	AFRO	112	1154	0	9	30	0.780	0.808
4473	2020-4-24 8:00	GH	AFRO	0	1154	0	9	31	0.780	0.781
4474	2020-4-25 8:00	GH	AFRO	125	1279	1	10	32	0.782	0.781
4475	2020-4-26 8:00	GH	AFRO	0	1279	0	10	33	0.782	0.758
4476	2020-4-27 8:00	GH	AFRO	271	1550	1	11	34	0.710	0.734
4477	2020-4-28 8:00	GH	AFRO	0	1550	0	11	35	0.710	0.792
4478	2020-4-29 8:00	GH	AFRO	121	1671	5	16	36	0.958	0.875
4479	2020-4-30 8:00	GH	AFRO	0	1671	0	16	37	0.958	0.912
4480	2020-5-1 8:00	GH	AFRO	403	2074	1	17	38	0.820	0.866
4481	2020-5-2 8:00	GH	AFRO	0	2074	0	17	39	0.820	0.820
4482										
4483	2020-3-3 8:00	GI	EURO	1	1	0	0			
4484	2020-3-4 8:00	GI	EURO	0	1	0	0			
4485	2020-3-5 8:00	GI	EURO	0	1	0	0			
4486	2020-3-6 8:00	GI	EURO	0	1	0	0			
4487	2020-3-7 8:00	GI	EURO	0	1	0	0			
4488	2020-3-8 8:00	GI	EURO	0	1	0	0			
4489	2020-3-9 8:00	GI	EURO	0	1	0	0			
4490	2020-3-10 8:00	GI	EURO	0	1	0	0			
4491	2020-3-11 8:00	GI	EURO	0	1	0	0			
4492	2020-3-12 8:00	GI	EURO	0	1	0	0			
4493	2020-3-13 8:00	GI	EURO	0	1	0	0			
4494	2020-3-14 8:00	GI	EURO	0	1	0	0			
4495	2020-3-15 8:00	GI	EURO	0	1	0	0			
4496	2020-3-16 8:00	GI	EURO	2	3	0	0			
4497	2020-3-17 8:00	GI	EURO	0	3	0	0			
4498	2020-3-18 8:00	GI	EURO	5	8	0	0			
4499	2020-3-19 8:00	GI	EURO	2	10	0	0			
4500	2020-3-20 8:00	GI	EURO	0	10	0	0			

Table S1 Daily confirmed cases and daily deaths of COVID-19 in 214 nations from WHO

ID	Date and time	Country or region	Continent	Confirmed patients	Cumulative Confirmed patients	Daily dead patients	Daily cumulative dead patients	Lag days	Daily CFR	3DMA CFR
4501	2020-3-21 8:00	GI	EURO	0	10	0	0			
4502	2020-3-22 8:00	GI	EURO	0	10	0	0			
4503	2020-3-23 8:00	GI	EURO	5	15	0	0			
4504	2020-3-24 8:00	GI	EURO	0	15	0	0			
4505	2020-3-25 8:00	GI	EURO	0	15	0	0			
4506	2020-3-26 8:00	GI	EURO	11	26	0	0			
4507	2020-3-27 8:00	GI	EURO	9	35	0	0			
4508	2020-3-28 8:00	GI	EURO	20	55	0	0			
4509	2020-3-29 8:00	GI	EURO	1	56	0	0			
4510	2020-3-30 8:00	GI	EURO	9	65	0	0			
4511	2020-3-31 8:00	GI	EURO	4	69	0	0			
4512	2020-4-1 8:00	GI	EURO	0	69	0	0			
4513	2020-4-2 8:00	GI	EURO	0	69	0	0			
4514	2020-4-3 8:00	GI	EURO	12	81	0	0			
4515	2020-4-4 8:00	GI	EURO	14	95	0	0			
4516	2020-4-5 8:00	GI	EURO	3	98	0	0			
4517	2020-4-6 8:00	GI	EURO	5	103	0	0			
4518	2020-4-7 8:00	GI	EURO	0	103	0	0			
4519	2020-4-8 8:00	GI	EURO	10	113	0	0			
4520	2020-4-9 8:00	GI	EURO	0	113	0	0			
4521	2020-4-10 8:00	GI	EURO	0	113	0	0			
4522	2020-4-11 8:00	GI	EURO	0	113	0	0			
4523	2020-4-12 8:00	GI	EURO	0	113	0	0			
4524	2020-4-13 8:00	GI	EURO	16	129	0	0			
4525	2020-4-14 8:00	GI	EURO	0	129	0	0			
4526	2020-4-15 8:00	GI	EURO	0	129	0	0			
4527	2020-4-16 8:00	GI	EURO	0	129	0	0			
4528	2020-4-17 8:00	GI	EURO	2	131	0	0			
4529	2020-4-18 8:00	GI	EURO	2	133	0	0			
4530	2020-4-19 8:00	GI	EURO	0	133	0	0			
4531	2020-4-20 8:00	GI	EURO	0	133	0	0			
4532	2020-4-21 8:00	GI	EURO	0	133	0	0			
4533	2020-4-22 8:00	GI	EURO	0	133	0	0			
4534	2020-4-23 8:00	GI	EURO	0	133	0	0			
4535	2020-4-24 8:00	GI	EURO	0	133	0	0			
4536	2020-4-25 8:00	GI	EURO	0	133	0	0			

Table S1 Daily confirmed cases and daily deaths of COVID-19 in 214 nations from WHO

ID	Date and time	Country or region	Continent	Confirmed patients	Cumulative Confirmed patients	Daily dead patients	Daily cumulative dead patients	Lag days	Daily CFR	3DMA CFR
4537	2020-4-26 8:00	GI	EURO	3	136	0	0			
4538	2020-4-27 8:00	GI	EURO	5	141	0	0			
4539	2020-4-28 8:00	GI	EURO	0	141	0	0			
4540	2020-4-29 8:00	GI	EURO	0	141	0	0			
4541	2020-4-30 8:00	GI	EURO	0	141	0	0			
4542	2020-5-1 8:00	GI	EURO	0	141	0	0			
4543	2020-5-2 8:00	GI	EURO	3	144	0	0			
4544										
4545	2020-2-26 8:00	GR	EURO	1	1	0	0			
4546	2020-2-27 8:00	GR	EURO	2	3	0	0			
4547	2020-2-28 8:00	GR	EURO	0	3	0	0			
4548	2020-2-29 8:00	GR	EURO	0	3	0	0			
4549	2020-3-1 8:00	GR	EURO	4	7	0	0			
4550	2020-3-2 8:00	GR	EURO	0	7	0	0			
4551	2020-3-3 8:00	GR	EURO	0	7	0	0			
4552	2020-3-4 8:00	GR	EURO	2	9	0	0			
4553	2020-3-5 8:00	GR	EURO	23	32	0	0			
4554	2020-3-6 8:00	GR	EURO	0	32	0	0			
4555	2020-3-7 8:00	GR	EURO	34	66	0	0			
4556	2020-3-8 8:00	GR	EURO	7	73	0	0			
4557	2020-3-9 8:00	GR	EURO	0	73	0	0			
4558	2020-3-10 8:00	GR	EURO	17	90	0	0			
4559	2020-3-11 8:00	GR	EURO	0	90	0	0			
4560	2020-3-12 8:00	GR	EURO	9	99	1	1	0	1.010	1.010
4561	2020-3-13 8:00	GR	EURO	0	99	0	1	1	1.010	0.820
4562	2020-3-14 8:00	GR	EURO	129	228	0	1	2	0.439	0.886
4563	2020-3-15 8:00	GR	EURO	103	331	3	4	3	1.208	0.952
4564	2020-3-16 8:00	GR	EURO	0	331	0	4	4	1.208	1.150
4565	2020-3-17 8:00	GR	EURO	56	387	0	4	5	1.034	1.146
4566	2020-3-18 8:00	GR	EURO	31	418	1	5	6	1.196	1.142
4567	2020-3-19 8:00	GR	EURO	0	418	0	5	7	1.196	1.336
4568	2020-3-20 8:00	GR	EURO	77	495	3	8	8	1.616	1.755
4569	2020-3-21 8:00	GR	EURO	35	530	5	13	9	2.453	2.174
4570	2020-3-22 8:00	GR	EURO	0	530	0	13	10	2.453	2.437
4571	2020-3-23 8:00	GR	EURO	94	624	2	15	11	2.404	2.434
4572	2020-3-24 8:00	GR	EURO	71	695	2	17	12	2.446	2.514

Table S1 Daily confirmed cases and daily deaths of COVID-19 in 214 nations from WHO

ID	Date and time	Country or region	Continent	Confirmed patients	Cumulative Confirmed patients	Daily dead patients	Daily cumulative dead patients	Lag days	Daily CFR	3DMA CFR
4573	2020-3-25 8:00	GR	EURO	48	743	3	20	13	2.692	2.606
4574	2020-3-26 8:00	GR	EURO	78	821	2	22	14	2.680	2.762
4575	2020-3-27 8:00	GR	EURO	71	892	4	26	15	2.915	2.831
4576	2020-3-28 8:00	GR	EURO	74	966	2	28	16	2.899	2.943
4577	2020-3-29 8:00	GR	EURO	95	1061	4	32	17	3.016	3.067
4578	2020-3-30 8:00	GR	EURO	95	1156	6	38	18	3.287	3.284
4579	2020-3-31 8:00	GR	EURO	56	1212	5	43	19	3.548	3.521
4580	2020-4-1 8:00	GR	EURO	102	1314	6	49	20	3.729	3.638
4581	2020-4-2 8:00	GR	EURO	61	1375	1	50	21	3.636	3.622
4582	2020-4-3 8:00	GR	EURO	139	1514	3	53	22	3.501	3.598
4583	2020-4-4 8:00	GR	EURO	99	1613	6	59	23	3.658	3.741
4584	2020-4-5 8:00	GR	EURO	60	1673	9	68	24	4.065	3.977
4585	2020-4-6 8:00	GR	EURO	62	1735	5	73	25	4.207	4.258
4586	2020-4-7 8:00	GR	EURO	20	1755	6	79	26	4.501	4.377
4587	2020-4-8 8:00	GR	EURO	77	1832	2	81	27	4.421	4.443
4588	2020-4-9 8:00	GR	EURO	52	1884	2	83	28	4.406	4.409
4589	2020-4-10 8:00	GR	EURO	71	1955	3	86	29	4.399	4.427
4590	2020-4-11 8:00	GR	EURO	56	2011	4	90	30	4.475	4.448
4591	2020-4-12 8:00	GR	EURO	70	2081	3	93	31	4.469	4.527
4592	2020-4-13 8:00	GR	EURO	33	2114	5	98	32	4.636	4.573
4593	2020-4-14 8:00	GR	EURO	31	2145	1	99	33	4.615	4.635
4594	2020-4-15 8:00	GR	EURO	25	2170	2	101	34	4.654	4.641
4595	2020-4-16 8:00	GR	EURO	22	2192	1	102	35	4.653	4.688
4596	2020-4-17 8:00	GR	EURO	15	2207	3	105	36	4.758	4.723
4597	2020-4-18 8:00	GR	EURO	0	2207	0	105	37	4.758	4.758
4598	2020-4-19 8:00	GR	EURO	0	2207	0	105	38	4.758	4.812
4599	2020-4-20 8:00	GR	EURO	28	2235	5	110	39	4.922	4.949
4600	2020-4-21 8:00	GR	EURO	10	2245	6	116	40	5.167	5.043
4601	2020-4-22 8:00	GR	EURO	156	2401	5	121	41	5.040	5.077
4602	2020-4-23 8:00	GR	EURO	7	2408	0	121	42	5.025	5.047
4603	2020-4-24 8:00	GR	EURO	55	2463	4	125	43	5.075	5.107
4604	2020-4-25 8:00	GR	EURO	27	2490	5	130	44	5.221	5.161
4605	2020-4-26 8:00	GR	EURO	16	2506	0	130	45	5.188	5.199
4606	2020-4-27 8:00	GR	EURO	0	2506	0	130	46	5.188	5.247
4607	2020-4-28 8:00	GR	EURO	28	2534	6	136	47	5.367	5.307
4608	2020-4-29 8:00	GR	EURO	0	2534	0	136	48	5.367	5.377

Table S1 Daily confirmed cases and daily deaths of COVID-19 in 214 nations from WHO

ID	Date and time	Country or region	Continent	Confirmed patients	Cumulative Confirmed patients	Daily dead patients	Daily cumulative dead patients	Lag days	Daily CFR	3DMA CFR
4609	2020-4-30 8:00	GR	EURO	42	2576	3	139	49	5.396	5.389
4610	2020-5-1 8:00	GR	EURO	15	2591	1	140	50	5.403	5.401
4611	2020-5-2 8:00	GR	EURO	0	2591	0	140	51	5.403	5.403
4612										
4613	2020-3-18 8:00	GL	EURO	2	2	0	0			
4614	2020-3-19 8:00	GL	EURO	0	2	0	0			
4615	2020-3-20 8:00	GL	EURO	0	2	0	0			
4616	2020-3-21 8:00	GL	EURO	0	2	0	0			
4617	2020-3-22 8:00	GL	EURO	0	2	0	0			
4618	2020-3-23 8:00	GL	EURO	0	2	0	0			
4619	2020-3-24 8:00	GL	EURO	2	4	0	0			
4620	2020-3-25 8:00	GL	EURO	0	4	0	0			
4621	2020-3-26 8:00	GL	EURO	1	5	0	0			
4622	2020-3-27 8:00	GL	EURO	1	6	0	0			
4623	2020-3-28 8:00	GL	EURO	3	9	0	0			
4624	2020-3-29 8:00	GL	EURO	1	10	0	0			
4625	2020-3-30 8:00	GL	EURO	0	10	0	0			
4626	2020-3-31 8:00	GL	EURO	0	10	0	0			
4627	2020-4-1 8:00	GL	EURO	0	10	0	0			
4628	2020-4-2 8:00	GL	EURO	0	10	0	0			
4629	2020-4-3 8:00	GL	EURO	0	10	0	0			
4630	2020-4-4 8:00	GL	EURO	0	10	0	0			
4631	2020-4-5 8:00	GL	EURO	0	10	0	0			
4632	2020-4-6 8:00	GL	EURO	1	11	0	0			
4633	2020-4-7 8:00	GL	EURO	0	11	0	0			
4634	2020-4-8 8:00	GL	EURO	0	11	0	0			
4635	2020-4-9 8:00	GL	EURO	0	11	0	0			
4636	2020-4-10 8:00	GL	EURO	0	11	0	0			
4637	2020-4-11 8:00	GL	EURO	0	11	0	0			
4638	2020-4-12 8:00	GL	EURO	0	11	0	0			
4639	2020-4-13 8:00	GL	EURO	0	11	0	0			
4640	2020-4-14 8:00	GL	EURO	0	11	0	0			
4641	2020-4-15 8:00	GL	EURO	0	11	0	0			
4642	2020-4-16 8:00	GL	EURO	0	11	0	0			
4643	2020-4-17 8:00	GL	EURO	0	11	0	0			
4644	2020-4-18 8:00	GL	EURO	0	11	0	0			

Table S1 Daily confirmed cases and daily deaths of COVID-19 in 214 nations from WHO

ID	Date and time	Country or region	Continent	Confirmed patients	Cumulative Confirmed patients	Daily dead patients	Daily cumulative dead patients	Lag days	Daily CFR	3DMA CFR
4645	2020-4-19 8:00	GL	EURO	0	11	0	0			
4646	2020-4-20 8:00	GL	EURO	0	11	0	0			
4647	2020-4-21 8:00	GL	EURO	0	11	0	0			
4648	2020-4-22 8:00	GL	EURO	0	11	0	0			
4649	2020-4-23 8:00	GL	EURO	0	11	0	0			
4650	2020-4-24 8:00	GL	EURO	0	11	0	0			
4651	2020-4-25 8:00	GL	EURO	0	11	0	0			
4652	2020-4-26 8:00	GL	EURO	0	11	0	0			
4653	2020-4-27 8:00	GL	EURO	0	11	0	0			
4654	2020-4-28 8:00	GL	EURO	0	11	0	0			
4655	2020-4-29 8:00	GL	EURO	0	11	0	0			
4656	2020-4-30 8:00	GL	EURO	0	11	0	0			
4657	2020-5-1 8:00	GL	EURO	0	11	0	0			
4658	2020-5-2 8:00	GL	EURO	0	11	0	0			
4659										
4660	2020-3-23 8:00	GD	AMRO	1	1	0	0			
4661	2020-3-24 8:00	GD	AMRO	0	1	0	0			
4662	2020-3-25 8:00	GD	AMRO	0	1	0	0			
4663	2020-3-26 8:00	GD	AMRO	6	7	0	0			
4664	2020-3-27 8:00	GD	AMRO	0	7	0	0			
4665	2020-3-28 8:00	GD	AMRO	0	7	0	0			
4666	2020-3-29 8:00	GD	AMRO	0	7	0	0			
4667	2020-3-30 8:00	GD	AMRO	0	7	0	0			
4668	2020-3-31 8:00	GD	AMRO	2	9	0	0			
4669	2020-4-1 8:00	GD	AMRO	0	9	0	0			
4670	2020-4-2 8:00	GD	AMRO	0	9	0	0			
4671	2020-4-3 8:00	GD	AMRO	1	10	0	0			
4672	2020-4-4 8:00	GD	AMRO	0	10	0	0			
4673	2020-4-5 8:00	GD	AMRO	2	12	0	0			
4674	2020-4-6 8:00	GD	AMRO	0	12	0	0			
4675	2020-4-7 8:00	GD	AMRO	0	12	0	0			
4676	2020-4-8 8:00	GD	AMRO	0	12	0	0			
4677	2020-4-9 8:00	GD	AMRO	0	12	0	0			
4678	2020-4-10 8:00	GD	AMRO	0	12	0	0			
4679	2020-4-11 8:00	GD	AMRO	0	12	0	0			
4680	2020-4-12 8:00	GD	AMRO	1	13	0	0			

Table S1 Daily confirmed cases and daily deaths of COVID-19 in 214 nations from WHO

ID	Date and time	Country or region	Continent	Confirmed patients	Cumulative Confirmed patients	Daily dead patients	Daily cumulative dead patients	Lag days	Daily CFR	3DMA CFR
4681	2020-4-13 8:00	GD	AMRO	0	13	0	0			
4682	2020-4-14 8:00	GD	AMRO	0	13	0	0			
4683	2020-4-15 8:00	GD	AMRO	0	13	0	0			
4684	2020-4-16 8:00	GD	AMRO	0	13	0	0			
4685	2020-4-17 8:00	GD	AMRO	0	13	0	0			
4686	2020-4-18 8:00	GD	AMRO	0	13	0	0			
4687	2020-4-19 8:00	GD	AMRO	0	13	0	0			
4688	2020-4-20 8:00	GD	AMRO	0	13	0	0			
4689	2020-4-21 8:00	GD	AMRO	0	13	0	0			
4690	2020-4-22 8:00	GD	AMRO	1	14	0	0			
4691	2020-4-23 8:00	GD	AMRO	0	14	0	0			
4692	2020-4-24 8:00	GD	AMRO	1	15	0	0			
4693	2020-4-25 8:00	GD	AMRO	0	15	0	0			
4694	2020-4-26 8:00	GD	AMRO	0	15	0	0			
4695	2020-4-27 8:00	GD	AMRO	3	18	0	0			
4696	2020-4-28 8:00	GD	AMRO	0	18	0	0			
4697	2020-4-29 8:00	GD	AMRO	0	18	0	0			
4698	2020-4-30 8:00	GD	AMRO	1	19	0	0			
4699	2020-5-1 8:00	GD	AMRO	1	20	0	0			
4700	2020-5-2 8:00	GD	AMRO	0	20	0	0			
4701										
4702	2020-3-14 8:00	GP	AMRO	3	3	0	0			
4703	2020-3-15 8:00	GP	AMRO	3	6	0	0			
4704	2020-3-16 8:00	GP	AMRO	0	6	0	0			
4705	2020-3-17 8:00	GP	AMRO	12	18	0	0			
4706	2020-3-18 8:00	GP	AMRO	15	33	0	0			
4707	2020-3-19 8:00	GP	AMRO	12	45	0	0			
4708	2020-3-20 8:00	GP	AMRO	6	51	0	0			
4709	2020-3-21 8:00	GP	AMRO	0	51	0	0			
4710	2020-3-22 8:00	GP	AMRO	0	51	0	0			
4711	2020-3-23 8:00	GP	AMRO	5	56	0	0			
4712	2020-3-24 8:00	GP	AMRO	17	73	0	0			
4713	2020-3-25 8:00	GP	AMRO	0	73	0	0			
4714	2020-3-26 8:00	GP	AMRO	3	76	0	0			
4715	2020-3-27 8:00	GP	AMRO	0	76	0	0			
4716	2020-3-28 8:00	GP	AMRO	20	96	2	2	0	2.083	2.083

Table S1 Daily confirmed cases and daily deaths of COVID-19 in 214 nations from WHO

ID	Date and time	Country or region	Continent	Confirmed patients	Cumulative Confirmed patients	Daily dead patients	Daily cumulative dead patients	Lag days	Daily CFR	3DMA CFR
4717	2020-3-29 8:00	GP	AMRO	0	96	0	2	1	2.083	2.083
4718	2020-3-30 8:00	GP	AMRO	0	96	0	2	2	2.083	2.647
4719	2020-3-31 8:00	GP	AMRO	10	106	2	4	3	3.774	3.414
4720	2020-4-1 8:00	GP	AMRO	8	114	1	5	4	4.386	4.320
4721	2020-4-2 8:00	GP	AMRO	11	125	1	6	5	4.800	4.624
4722	2020-4-3 8:00	GP	AMRO	3	128	0	6	6	4.688	4.957
4723	2020-4-4 8:00	GP	AMRO	2	130	1	7	7	5.385	5.152
4724	2020-4-5 8:00	GP	AMRO	0	130	0	7	8	5.385	5.331
4725	2020-4-6 8:00	GP	AMRO	4	134	0	7	9	5.224	5.265
4726	2020-4-7 8:00	GP	AMRO	1	135	0	7	10	5.185	5.148
4727	2020-4-8 8:00	GP	AMRO	4	139	0	7	11	5.036	5.326
4728	2020-4-9 8:00	GP	AMRO	0	139	1	8	12	5.755	5.488
4729	2020-4-10 8:00	GP	AMRO	2	141	0	8	13	5.674	5.675
4730	2020-4-11 8:00	GP	AMRO	2	143	0	8	14	5.594	5.621
4731	2020-4-12 8:00	GP	AMRO	0	143	0	8	15	5.594	5.594
4732	2020-4-13 8:00	GP	AMRO	0	143	0	8	16	5.594	5.594
4733	2020-4-14 8:00	GP	AMRO	0	143	0	8	17	5.594	5.569
4734	2020-4-15 8:00	GP	AMRO	2	145	0	8	18	5.517	5.543
4735	2020-4-16 8:00	GP	AMRO	0	145	0	8	19	5.517	5.517
4736	2020-4-17 8:00	GP	AMRO	0	145	0	8	20	5.517	5.517
4737	2020-4-18 8:00	GP	AMRO	0	145	0	8	21	5.517	5.977
4738	2020-4-19 8:00	GP	AMRO	0	145	2	10	22	6.897	6.390
4739	2020-4-20 8:00	GP	AMRO	3	148	0	10	23	6.757	6.803
4740	2020-4-21 8:00	GP	AMRO	0	148	0	10	24	6.757	6.757
4741	2020-4-22 8:00	GP	AMRO	0	148	0	10	25	6.757	6.757
4742	2020-4-23 8:00	GP	AMRO	0	148	0	10	26	6.757	6.757
4743	2020-4-24 8:00	GP	AMRO	0	148	0	10	27	6.757	6.742
4744	2020-4-25 8:00	GP	AMRO	1	149	0	10	28	6.711	6.727
4745	2020-4-26 8:00	GP	AMRO	0	149	0	10	29	6.711	6.711
4746	2020-4-27 8:00	GP	AMRO	0	149	0	10	30	6.711	6.711
4747	2020-4-28 8:00	GP	AMRO	0	149	0	10	31	6.711	6.935
4748	2020-4-29 8:00	GP	AMRO	0	149	1	11	32	7.383	7.126
4749	2020-4-30 8:00	GP	AMRO	2	151	0	11	33	7.285	7.521
4750	2020-5-1 8:00	GP	AMRO	1	152	1	12	34	7.895	7.691
4751	2020-5-2 8:00	GP	AMRO	0	152	0	12	35	7.895	7.895
4752										

Table S1 Daily confirmed cases and daily deaths of COVID-19 in 214 nations from WHO

ID	Date and time	Country or region	Continent	Confirmed patients	Cumulative Confirmed patients	Daily dead patients	Daily cumulative dead patients	Lag days	Daily CFR	3DMA CFR
4753	2020-3-17 8:00	GU	WPRO	3	3	0	0			
4754	2020-3-18 8:00	GU	WPRO	2	5	0	0			
4755	2020-3-19 8:00	GU	WPRO	7	12	0	0			
4756	2020-3-20 8:00	GU	WPRO	2	14	0	0			
4757	2020-3-21 8:00	GU	WPRO	1	15	0	0			
4758	2020-3-22 8:00	GU	WPRO	0	15	0	0			
4759	2020-3-23 8:00	GU	WPRO	12	27	1	1	0	3.704	3.576
4760	2020-3-24 8:00	GU	WPRO	2	29	0	1	1	3.448	3.426
4761	2020-3-25 8:00	GU	WPRO	3	32	0	1	2	3.125	3.092
4762	2020-3-26 8:00	GU	WPRO	5	37	0	1	3	2.703	2.683
4763	2020-3-27 8:00	GU	WPRO	8	45	0	1	4	2.222	2.295
4764	2020-3-28 8:00	GU	WPRO	6	51	0	1	5	1.961	1.990
4765	2020-3-29 8:00	GU	WPRO	5	56	0	1	6	1.786	1.844
4766	2020-3-30 8:00	GU	WPRO	0	56	0	1	7	1.786	1.765
4767	2020-3-31 8:00	GU	WPRO	2	58	0	1	8	1.724	2.136
4768	2020-4-1 8:00	GU	WPRO	11	69	1	2	9	2.899	2.840
4769	2020-4-2 8:00	GU	WPRO	8	77	1	3	10	3.896	3.484
4770	2020-4-3 8:00	GU	WPRO	5	82	0	3	11	3.659	3.709
4771	2020-4-4 8:00	GU	WPRO	2	84	0	3	12	3.571	3.844
4772	2020-4-5 8:00	GU	WPRO	9	93	1	4	13	4.301	3.815
4773	2020-4-6 8:00	GU	WPRO	19	112	0	4	14	3.571	3.804
4774	2020-4-7 8:00	GU	WPRO	1	113	0	4	15	3.540	3.446
4775	2020-4-8 8:00	GU	WPRO	11	124	0	4	16	3.226	3.322
4776	2020-4-9 8:00	GU	WPRO	1	125	0	4	17	3.200	3.184
4777	2020-4-10 8:00	GU	WPRO	3	128	0	4	18	3.125	3.134
4778	2020-4-11 8:00	GU	WPRO	2	130	0	4	19	3.077	3.320
4779	2020-4-12 8:00	GU	WPRO	3	133	1	5	20	3.759	3.532
4780	2020-4-13 8:00	GU	WPRO	0	133	0	5	21	3.759	3.759
4781	2020-4-14 8:00	GU	WPRO	0	133	0	5	22	3.759	3.759
4782	2020-4-15 8:00	GU	WPRO	0	133	0	5	23	3.759	3.759
4783	2020-4-16 8:00	GU	WPRO	0	133	0	5	24	3.759	3.759
4784	2020-4-17 8:00	GU	WPRO	0	133	0	5	25	3.759	3.759
4785	2020-4-18 8:00	GU	WPRO	0	133	0	5	26	3.759	3.759
4786	2020-4-19 8:00	GU	WPRO	0	133	0	5	27	3.759	3.759
4787	2020-4-20 8:00	GU	WPRO	0	133	0	5	28	3.759	3.759
4788	2020-4-21 8:00	GU	WPRO	0	133	0	5	29	3.759	3.759

Table S1 Daily confirmed cases and daily deaths of COVID-19 in 214 nations from WHO

ID	Date and time	Country or region	Continent	Confirmed patients	Cumulative Confirmed patients	Daily dead patients	Daily cumulative dead patients	Lag days	Daily CFR	3DMA CFR
4789	2020-4-22 8:00	GU	WPRO	0	133	0	5	30	3.759	3.741
4790	2020-4-23 8:00	GU	WPRO	2	135	0	5	31	3.704	3.713
4791	2020-4-24 8:00	GU	WPRO	1	136	0	5	32	3.676	3.686
4792	2020-4-25 8:00	GU	WPRO	0	136	0	5	33	3.676	3.668
4793	2020-4-26 8:00	GU	WPRO	1	137	0	5	34	3.650	3.650
4794	2020-4-27 8:00	GU	WPRO	1	138	0	5	35	3.623	3.615
4795	2020-4-28 8:00	GU	WPRO	2	140	0	5	36	3.571	3.580
4796	2020-4-29 8:00	GU	WPRO	1	141	0	5	37	3.546	3.555
4797	2020-4-30 8:00	GU	WPRO	0	141	0	5	38	3.546	3.538
4798	2020-5-1 8:00	GU	WPRO	1	142	0	5	39	3.521	3.529
4799	2020-5-2 8:00	GU	WPRO	0	142	0	5	40	3.521	35.681
4800										
4801	2020-3-15 8:00	GT	AMRO	1	1	1	1	0	100.000	67.840
4802	2020-3-16 8:00	GT	AMRO	0	1	0	1	1	100.000	72.222
4803	2020-3-17 8:00	GT	AMRO	5	6	0	1	2	16.667	44.444
4804	2020-3-18 8:00	GT	AMRO	0	6	0	1	3	16.667	14.815
4805	2020-3-19 8:00	GT	AMRO	3	9	0	1	4	11.111	12.037
4806	2020-3-20 8:00	GT	AMRO	3	12	0	1	5	8.333	9.259
4807	2020-3-21 8:00	GT	AMRO	0	12	0	1	6	8.333	8.333
4808	2020-3-22 8:00	GT	AMRO	0	12	0	1	7	8.333	7.407
4809	2020-3-23 8:00	GT	AMRO	6	18	0	1	8	5.556	6.217
4810	2020-3-24 8:00	GT	AMRO	3	21	0	1	9	4.762	5.026
4811	2020-3-25 8:00	GT	AMRO	0	21	0	1	10	4.762	4.563
4812	2020-3-26 8:00	GT	AMRO	3	24	0	1	11	4.167	4.365
4813	2020-3-27 8:00	GT	AMRO	0	24	0	1	12	4.167	4.111
4814	2020-3-28 8:00	GT	AMRO	1	25	0	1	13	4.000	3.764
4815	2020-3-29 8:00	GT	AMRO	7	32	0	1	14	3.125	3.355
4816	2020-3-30 8:00	GT	AMRO	2	34	0	1	15	2.941	2.948
4817	2020-3-31 8:00	GT	AMRO	2	36	0	1	16	2.778	2.832
4818	2020-4-1 8:00	GT	AMRO	0	36	0	1	17	2.778	2.707
4819	2020-4-2 8:00	GT	AMRO	3	39	0	1	18	2.564	2.490
4820	2020-4-3 8:00	GT	AMRO	8	47	0	1	19	2.128	2.231
4821	2020-4-4 8:00	GT	AMRO	3	50	0	1	20	2.000	2.043
4822	2020-4-5 8:00	GT	AMRO	0	50	0	1	21	2.000	2.426
4823	2020-4-6 8:00	GT	AMRO	11	61	1	2	22	3.279	3.188
4824	2020-4-7 8:00	GT	AMRO	9	70	1	3	23	4.286	3.820

Table S1 Daily confirmed cases and daily deaths of COVID-19 in 214 nations from WHO

ID	Date and time	Country or region	Continent	Confirmed patients	Cumulative Confirmed patients	Daily dead patients	Daily cumulative dead patients	Lag days	Daily CFR	3DMA CFR
4825	2020-4-8 8:00	GT	AMRO	7	77	0	3	24	3.896	3.877
4826	2020-4-9 8:00	GT	AMRO	10	87	0	3	25	3.448	3.501
4827	2020-4-10 8:00	GT	AMRO	8	95	0	3	26	3.158	2.996
4828	2020-4-11 8:00	GT	AMRO	31	126	0	3	27	2.381	2.576
4829	2020-4-12 8:00	GT	AMRO	11	137	0	3	28	2.190	2.177
4830	2020-4-13 8:00	GT	AMRO	16	153	0	3	29	1.961	2.452
4831	2020-4-14 8:00	GT	AMRO	3	156	2	5	30	3.205	2.720
4832	2020-4-15 8:00	GT	AMRO	11	167	0	5	31	2.994	2.992
4833	2020-4-16 8:00	GT	AMRO	13	180	0	5	32	2.778	2.774
4834	2020-4-17 8:00	GT	AMRO	16	196	0	5	33	2.551	2.867
4835	2020-4-18 8:00	GT	AMRO	18	214	2	7	34	3.271	2.934
4836	2020-4-19 8:00	GT	AMRO	21	235	0	7	35	2.979	2.991
4837	2020-4-20 8:00	GT	AMRO	22	257	0	7	36	2.724	2.708
4838	2020-4-21 8:00	GT	AMRO	32	289	0	7	37	2.422	2.622
4839	2020-4-22 8:00	GT	AMRO	5	294	1	8	38	2.721	2.558
4840	2020-4-23 8:00	GT	AMRO	22	316	0	8	39	2.532	2.726
4841	2020-4-24 8:00	GT	AMRO	26	342	2	10	40	2.924	2.773
4842	2020-4-25 8:00	GT	AMRO	42	384	1	11	41	2.865	2.937
4843	2020-4-26 8:00	GT	AMRO	46	430	2	13	42	3.023	3.020
4844	2020-4-27 8:00	GT	AMRO	43	473	2	15	43	3.171	3.065
4845	2020-4-28 8:00	GT	AMRO	27	500	0	15	44	3.000	3.000
4846	2020-4-29 8:00	GT	AMRO	30	530	0	15	45	2.830	2.901
4847	2020-4-30 8:00	GT	AMRO	27	557	1	16	46	2.873	2.813
4848	2020-5-1 8:00	GT	AMRO	28	585	0	16	47	2.735	2.760
4849	2020-5-2 8:00	GT	AMRO	14	599	0	16	48	2.671	2.703
4850										
4851	2020-3-9 8:00	GG	EURO	1	1	0	0			
4852	2020-3-10 8:00	GG	EURO	0	1	0	0			
4853	2020-3-11 8:00	GG	EURO	0	1	0	0			
4854	2020-3-12 8:00	GG	EURO	0	1	0	0			
4855	2020-3-13 8:00	GG	EURO	0	1	0	0			
4856	2020-3-14 8:00	GG	EURO	0	1	0	0			
4857	2020-3-15 8:00	GG	EURO	0	1	0	0			
4858	2020-3-16 8:00	GG	EURO	0	1	0	0			
4859	2020-3-17 8:00	GG	EURO	0	1	0	0			
4860	2020-3-18 8:00	GG	EURO	0	1	0	0			

Table S1 Daily confirmed cases and daily deaths of COVID-19 in 214 nations from WHO

ID	Date and time	Country or region	Continent	Confirmed patients	Cumulative Confirmed patients	Daily dead patients	Daily cumulative dead patients	Lag days	Daily CFR	3DMA CFR
4861	2020-3-19 8:00	GG	EURO	0	1	0	0			
4862	2020-3-20 8:00	GG	EURO	0	1	0	0			
4863	2020-3-21 8:00	GG	EURO	0	1	0	0			
4864	2020-3-22 8:00	GG	EURO	0	1	0	0			
4865	2020-3-23 8:00	GG	EURO	16	17	0	0			
4866	2020-3-24 8:00	GG	EURO	3	20	0	0			
4867	2020-3-25 8:00	GG	EURO	3	23	0	0			
4868	2020-3-26 8:00	GG	EURO	7	30	0	0			
4869	2020-3-27 8:00	GG	EURO	4	34	0	0			
4870	2020-3-28 8:00	GG	EURO	2	36	0	0			
4871	2020-3-29 8:00	GG	EURO	3	39	0	0			
4872	2020-3-30 8:00	GG	EURO	0	39	0	0			
4873	2020-3-31 8:00	GG	EURO	6	45	0	0			
4874	2020-4-1 8:00	GG	EURO	15	60	1	1	0	1.667	1.474
4875	2020-4-2 8:00	GG	EURO	18	78	0	1	1	1.282	1.349
4876	2020-4-3 8:00	GG	EURO	13	91	0	1	2	1.099	1.378
4877	2020-4-4 8:00	GG	EURO	23	114	1	2	3	1.754	1.441
4878	2020-4-5 8:00	GG	EURO	22	136	0	2	4	1.471	1.724
4879	2020-4-6 8:00	GG	EURO	18	154	1	3	5	1.948	1.789
4880	2020-4-7 8:00	GG	EURO	0	154	0	3	6	1.948	2.102
4881	2020-4-8 8:00	GG	EURO	12	166	1	4	7	2.410	2.256
4882	2020-4-9 8:00	GG	EURO	0	166	0	4	8	2.410	2.527
4883	2020-4-10 8:00	GG	EURO	15	181	1	5	9	2.762	2.597
4884	2020-4-11 8:00	GG	EURO	10	191	0	5	10	2.618	2.793
4885	2020-4-12 8:00	GG	EURO	9	200	1	6	11	3.000	2.830
4886	2020-4-13 8:00	GG	EURO	9	209	0	6	12	2.871	2.874
4887	2020-4-14 8:00	GG	EURO	9	218	0	6	13	2.752	2.788
4888	2020-4-15 8:00	GG	EURO	1	219	0	6	14	2.740	2.877
4889	2020-4-16 8:00	GG	EURO	4	223	1	7	15	3.139	3.129
4890	2020-4-17 8:00	GG	EURO	5	228	1	8	16	3.509	3.498
4891	2020-4-18 8:00	GG	EURO	6	234	1	9	17	3.846	3.723
4892	2020-4-19 8:00	GG	EURO	2	236	0	9	18	3.814	3.808
4893	2020-4-20 8:00	GG	EURO	3	239	0	9	19	3.766	3.782
4894	2020-4-21 8:00	GG	EURO	0	239	0	9	20	3.766	3.905
4895	2020-4-22 8:00	GG	EURO	0	239	1	10	21	4.184	4.033
4896	2020-4-23 8:00	GG	EURO	2	241	0	10	22	4.149	4.150

Table S1 Daily confirmed cases and daily deaths of COVID-19 in 214 nations from WHO

ID	Date and time	Country or region	Continent	Confirmed patients	Cumulative Confirmed patients	Daily dead patients	Daily cumulative dead patients	Lag days	Daily CFR	3DMA CFR
4897	2020-4-24 8:00	GG	EURO	2	243	0	10	23	4.115	4.115
4898	2020-4-25 8:00	GG	EURO	2	245	0	10	24	4.082	4.229
4899	2020-4-26 8:00	GG	EURO	0	245	1	11	25	4.490	4.490
4900	2020-4-27 8:00	GG	EURO	0	245	1	12	26	4.898	4.884
4901	2020-4-28 8:00	GG	EURO	2	247	1	13	27	5.263	5.141
4902	2020-4-29 8:00	GG	EURO	0	247	0	13	28	5.263	5.263
4903	2020-4-30 8:00	GG	EURO	0	247	0	13	29	5.263	5.235
4904	2020-5-1 8:00	GG	EURO	4	251	0	13	30	5.179	5.207
4905	2020-5-2 8:00	GG	EURO	0	251	0	13	31	5.179	5.179
4906										
4907	2020-3-14 8:00	GN	AFRO	1	1	0	0			
4908	2020-3-15 8:00	GN	AFRO	0	1	0	0			
4909	2020-3-16 8:00	GN	AFRO	0	1	0	0			
4910	2020-3-17 8:00	GN	AFRO	0	1	0	0			
4911	2020-3-18 8:00	GN	AFRO	0	1	0	0			
4912	2020-3-19 8:00	GN	AFRO	1	2	0	0			
4913	2020-3-20 8:00	GN	AFRO	0	2	0	0			
4914	2020-3-21 8:00	GN	AFRO	0	2	0	0			
4915	2020-3-22 8:00	GN	AFRO	0	2	0	0			
4916	2020-3-23 8:00	GN	AFRO	0	2	0	0			
4917	2020-3-24 8:00	GN	AFRO	2	4	0	0			
4918	2020-3-25 8:00	GN	AFRO	0	4	0	0			
4919	2020-3-26 8:00	GN	AFRO	0	4	0	0			
4920	2020-3-27 8:00	GN	AFRO	1	5	0	0			
4921	2020-3-28 8:00	GN	AFRO	3	8	0	0			
4922	2020-3-29 8:00	GN	AFRO	0	8	0	0			
4923	2020-3-30 8:00	GN	AFRO	8	16	0	0			
4924	2020-3-31 8:00	GN	AFRO	0	16	0	0			
4925	2020-4-1 8:00	GN	AFRO	0	16	0	0			
4926	2020-4-2 8:00	GN	AFRO	14	30	0	0			
4927	2020-4-3 8:00	GN	AFRO	22	52	0	0			
4928	2020-4-4 8:00	GN	AFRO	0	52	0	0			
4929	2020-4-5 8:00	GN	AFRO	59	111	0	0			
4930	2020-4-6 8:00	GN	AFRO	0	111	0	0			
4931	2020-4-7 8:00	GN	AFRO	0	111	0	0			
4932	2020-4-8 8:00	GN	AFRO	33	144	0	0			

Table S1 Daily confirmed cases and daily deaths of COVID-19 in 214 nations from WHO

ID	Date and time	Country or region	Continent	Confirmed patients	Cumulative Confirmed patients	Daily dead patients	Daily cumulative dead patients	Lag days	Daily CFR	3DMA CFR
4933	2020-4-9 8:00	GN	AFRO	20	164	0	0			
4934	2020-4-10 8:00	GN	AFRO	30	194	0	0			
4935	2020-4-11 8:00	GN	AFRO	0	194	0	0			
4936	2020-4-12 8:00	GN	AFRO	56	250	0	0			
4937	2020-4-13 8:00	GN	AFRO	0	250	0	0			
4938	2020-4-14 8:00	GN	AFRO	69	319	0	0			
4939	2020-4-15 8:00	GN	AFRO	44	363	0	0			
4940	2020-4-16 8:00	GN	AFRO	41	404	1	1	0	0.248	0.238
4941	2020-4-17 8:00	GN	AFRO	34	438	0	1	1	0.228	0.235
4942	2020-4-18 8:00	GN	AFRO	0	438	0	1	2	0.228	0.362
4943	2020-4-19 8:00	GN	AFRO	39	477	2	3	3	0.629	0.495
4944	2020-4-20 8:00	GN	AFRO	0	477	0	3	4	0.629	0.687
4945	2020-4-21 8:00	GN	AFRO	145	622	2	5	5	0.804	0.768
4946	2020-4-22 8:00	GN	AFRO	66	688	1	6	6	0.872	0.821
4947	2020-4-23 8:00	GN	AFRO	73	761	0	6	7	0.788	0.786
4948	2020-4-24 8:00	GN	AFRO	101	862	0	6	8	0.696	0.704
4949	2020-4-25 8:00	GN	AFRO	92	954	0	6	9	0.629	0.676
4950	2020-4-26 8:00	GN	AFRO	42	996	1	7	10	0.703	0.657
4951	2020-4-27 8:00	GN	AFRO	98	1094	0	7	11	0.640	0.648
4952	2020-4-28 8:00	GN	AFRO	69	1163	0	7	12	0.602	0.602
4953	2020-4-29 8:00	GN	AFRO	77	1240	0	7	13	0.565	0.562
4954	2020-4-30 8:00	GN	AFRO	111	1351	0	7	14	0.518	0.517
4955	2020-5-1 8:00	GN	AFRO	144	1495	0	7	15	0.468	0.481
4956	2020-5-2 8:00	GN	AFRO	42	1537	0	7	16	0.455	0.462
4957										
4958	2020-3-26 8:00	GW	AFRO	2	2	0	0			
4959	2020-3-27 8:00	GW	AFRO	0	2	0	0			
4960	2020-3-28 8:00	GW	AFRO	0	2	0	0			
4961	2020-3-29 8:00	GW	AFRO	0	2	0	0			
4962	2020-3-30 8:00	GW	AFRO	0	2	0	0			
4963	2020-3-31 8:00	GW	AFRO	6	8	0	0			
4964	2020-4-1 8:00	GW	AFRO	1	9	0	0			
4965	2020-4-2 8:00	GW	AFRO	0	9	0	0			
4966	2020-4-3 8:00	GW	AFRO	0	9	0	0			
4967	2020-4-4 8:00	GW	AFRO	6	15	0	0			
4968	2020-4-5 8:00	GW	AFRO	3	18	0	0			

Table S1 Daily confirmed cases and daily deaths of COVID-19 in 214 nations from WHO

ID	Date and time	Country or region	Continent	Confirmed patients	Cumulative Confirmed patients	Daily dead patients	Daily cumulative dead patients	Lag days	Daily CFR	3DMA CFR
4969	2020-4-6 8:00	GW	AFRO	0	18	0	0			
4970	2020-4-7 8:00	GW	AFRO	15	33	0	0			
4971	2020-4-8 8:00	GW	AFRO	0	33	0	0			
4972	2020-4-9 8:00	GW	AFRO	0	33	0	0			
4973	2020-4-10 8:00	GW	AFRO	2	35	0	0			
4974	2020-4-11 8:00	GW	AFRO	0	35	0	0			
4975	2020-4-12 8:00	GW	AFRO	3	38	0	0			
4976	2020-4-13 8:00	GW	AFRO	1	39	0	0			
4977	2020-4-14 8:00	GW	AFRO	0	39	0	0			
4978	2020-4-15 8:00	GW	AFRO	1	40	0	0			
4979	2020-4-16 8:00	GW	AFRO	3	43	0	0			
4980	2020-4-17 8:00	GW	AFRO	3	46	0	0			
4981	2020-4-18 8:00	GW	AFRO	4	50	0	0			
4982	2020-4-19 8:00	GW	AFRO	0	50	0	0			
4983	2020-4-20 8:00	GW	AFRO	0	50	0	0			
4984	2020-4-21 8:00	GW	AFRO	0	50	0	0			
4985	2020-4-22 8:00	GW	AFRO	0	50	0	0			
4986	2020-4-23 8:00	GW	AFRO	0	50	0	0			
4987	2020-4-24 8:00	GW	AFRO	2	52	0	0			
4988	2020-4-25 8:00	GW	AFRO	0	52	0	0			
4989	2020-4-26 8:00	GW	AFRO	0	52	0	0			
4990	2020-4-27 8:00	GW	AFRO	1	53	1	1	0	1.887	1.628
4991	2020-4-28 8:00	GW	AFRO	20	73	0	1	1	1.370	1.542
4992	2020-4-29 8:00	GW	AFRO	0	73	0	1	2	1.370	1.346
4993	2020-4-30 8:00	GW	AFRO	4	77	0	1	3	1.299	1.055
4994	2020-5-1 8:00	GW	AFRO	124	201	0	1	4	0.498	0.728
4995	2020-5-2 8:00	GW	AFRO	56	257	0	1	5	0.389	33.629
4996										
4997	2020-3-13 8:00	GY	AMRO	1	1	1	1	0	100.000	66.796
4998	2020-3-14 8:00	GY	AMRO	0	1	0	1	1	100.000	75.000
4999	2020-3-15 8:00	GY	AMRO	3	4	0	1	2	25.000	50.000
5000	2020-3-16 8:00	GY	AMRO	0	4	0	1	3	25.000	25.000
5001	2020-3-17 8:00	GY	AMRO	0	4	0	1	4	25.000	23.333
5002	2020-3-18 8:00	GY	AMRO	1	5	0	1	5	20.000	21.667
5003	2020-3-19 8:00	GY	AMRO	0	5	0	1	6	20.000	20.000
5004	2020-3-20 8:00	GY	AMRO	0	5	0	1	7	20.000	20.000

Table S1 Daily confirmed cases and daily deaths of COVID-19 in 214 nations from WHO

ID	Date and time	Country or region	Continent	Confirmed patients	Cumulative Confirmed patients	Daily dead patients	Daily cumulative dead patients	Lag days	Daily CFR	3DMA CFR
5005	2020-3-21 8:00	GY	AMRO	0	5	0	1	8	20.000	20.000
5006	2020-3-22 8:00	GY	AMRO	0	5	0	1	9	20.000	20.000
5007	2020-3-23 8:00	GY	AMRO	0	5	0	1	10	20.000	20.000
5008	2020-3-24 8:00	GY	AMRO	0	5	0	1	11	20.000	20.000
5009	2020-3-25 8:00	GY	AMRO	0	5	0	1	12	20.000	20.000
5010	2020-3-26 8:00	GY	AMRO	0	5	0	1	13	20.000	20.000
5011	2020-3-27 8:00	GY	AMRO	0	5	0	1	14	20.000	20.000
5012	2020-3-28 8:00	GY	AMRO	0	5	0	1	15	20.000	20.000
5013	2020-3-29 8:00	GY	AMRO	0	5	0	1	16	20.000	20.000
5014	2020-3-30 8:00	GY	AMRO	0	5	0	1	17	20.000	17.500
5015	2020-3-31 8:00	GY	AMRO	3	8	0	1	18	12.500	16.389
5016	2020-4-1 8:00	GY	AMRO	4	12	1	2	19	16.667	13.231
5017	2020-4-2 8:00	GY	AMRO	7	19	0	2	20	10.526	16.082
5018	2020-4-3 8:00	GY	AMRO	0	19	2	4	21	21.053	17.544
5019	2020-4-4 8:00	GY	AMRO	0	19	0	4	22	21.053	19.832
5020	2020-4-5 8:00	GY	AMRO	4	23	0	4	23	17.391	18.370
5021	2020-4-6 8:00	GY	AMRO	1	24	0	4	24	16.667	15.950
5022	2020-4-7 8:00	GY	AMRO	5	29	0	4	25	13.793	15.530
5023	2020-4-8 8:00	GY	AMRO	2	31	1	5	26	16.129	15.025
5024	2020-4-9 8:00	GY	AMRO	2	33	0	5	27	15.152	15.832
5025	2020-4-10 8:00	GY	AMRO	4	37	1	6	28	16.216	15.861
5026	2020-4-11 8:00	GY	AMRO	0	37	0	6	29	16.216	16.216
5027	2020-4-12 8:00	GY	AMRO	0	37	0	6	30	16.216	16.216
5028	2020-4-13 8:00	GY	AMRO	0	37	0	6	31	16.216	15.066
5029	2020-4-14 8:00	GY	AMRO	10	47	0	6	32	12.766	13.916
5030	2020-4-15 8:00	GY	AMRO	0	47	0	6	33	12.766	12.677
5031	2020-4-16 8:00	GY	AMRO	1	48	0	6	34	12.500	12.058
5032	2020-4-17 8:00	GY	AMRO	7	55	0	6	35	10.909	11.312
5033	2020-4-18 8:00	GY	AMRO	2	57	0	6	36	10.526	10.320
5034	2020-4-19 8:00	GY	AMRO	6	63	0	6	37	9.524	10.387
5035	2020-4-20 8:00	GY	AMRO	0	63	1	7	38	11.111	10.468
5036	2020-4-21 8:00	GY	AMRO	2	65	0	7	39	10.769	10.829
5037	2020-4-22 8:00	GY	AMRO	1	66	0	7	40	10.606	10.608
5038	2020-4-23 8:00	GY	AMRO	1	67	0	7	41	10.448	10.501
5039	2020-4-24 8:00	GY	AMRO	0	67	0	7	42	10.448	10.299
5040	2020-4-25 8:00	GY	AMRO	3	70	0	7	43	10.000	10.012

Table S1 Daily confirmed cases and daily deaths of COVID-19 in 214 nations from WHO

ID	Date and time	Country or region	Continent	Confirmed patients	Cumulative Confirmed patients	Daily dead patients	Daily cumulative dead patients	Lag days	Daily CFR	3DMA CFR
5041	2020-4-26 8:00	GY	AMRO	3	73	0	7	44	9.589	10.133
5042	2020-4-27 8:00	GY	AMRO	1	74	1	8	45	10.811	10.404
5043	2020-4-28 8:00	GY	AMRO	0	74	0	8	46	10.811	10.811
5044	2020-4-29 8:00	GY	AMRO	0	74	0	8	47	10.811	10.763
5045	2020-4-30 8:00	GY	AMRO	1	75	0	8	48	10.667	10.578
5046	2020-5-1 8:00	GY	AMRO	3	78	0	8	49	10.256	10.633
5047	2020-5-2 8:00	GY	AMRO	4	82	1	9	50	10.976	10.616
5048										
5049	2020-3-20 8:00	HT	AMRO	2	2	0	0			
5050	2020-3-21 8:00	HT	AMRO	0	2	0	0			
5051	2020-3-22 8:00	HT	AMRO	0	2	0	0			
5052	2020-3-23 8:00	HT	AMRO	0	2	0	0			
5053	2020-3-24 8:00	HT	AMRO	5	7	0	0			
5054	2020-3-25 8:00	HT	AMRO	0	7	0	0			
5055	2020-3-26 8:00	HT	AMRO	1	8	0	0			
5056	2020-3-27 8:00	HT	AMRO	0	8	0	0			
5057	2020-3-28 8:00	HT	AMRO	0	8	0	0			
5058	2020-3-29 8:00	HT	AMRO	0	8	0	0			
5059	2020-3-30 8:00	HT	AMRO	7	15	0	0			
5060	2020-3-31 8:00	HT	AMRO	0	15	0	0			
5061	2020-4-1 8:00	HT	AMRO	0	15	0	0			
5062	2020-4-2 8:00	HT	AMRO	1	16	0	0			
5063	2020-4-3 8:00	HT	AMRO	0	16	0	0			
5064	2020-4-4 8:00	HT	AMRO	2	18	0	0			
5065	2020-4-5 8:00	HT	AMRO	0	18	0	0			
5066	2020-4-6 8:00	HT	AMRO	3	21	0	0			
5067	2020-4-7 8:00	HT	AMRO	3	24	1	1	0	4.167	4.083
5068	2020-4-8 8:00	HT	AMRO	1	25	0	1	1	4.000	3.957
5069	2020-4-9 8:00	HT	AMRO	2	27	0	1	2	3.704	4.790
5070	2020-4-10 8:00	HT	AMRO	3	30	1	2	3	6.667	5.679
5071	2020-4-11 8:00	HT	AMRO	0	30	0	2	4	6.667	6.595
5072	2020-4-12 8:00	HT	AMRO	1	31	0	2	5	6.452	6.523
5073	2020-4-13 8:00	HT	AMRO	0	31	0	2	6	6.452	6.801
5074	2020-4-14 8:00	HT	AMRO	9	40	1	3	7	7.500	7.151
5075	2020-4-15 8:00	HT	AMRO	0	40	0	3	8	7.500	7.439
5076	2020-4-16 8:00	HT	AMRO	1	41	0	3	9	7.317	7.378

Table S1 Daily confirmed cases and daily deaths of COVID-19 in 214 nations from WHO

ID	Date and time	Country or region	Continent	Confirmed patients	Cumulative Confirmed patients	Daily dead patients	Daily cumulative dead patients	Lag days	Daily CFR	3DMA CFR
5077	2020-4-17 8:00	HT	AMRO	0	41	0	3	10	7.317	7.204
5078	2020-4-18 8:00	HT	AMRO	2	43	0	3	11	6.977	7.037
5079	2020-4-19 8:00	HT	AMRO	1	44	0	3	12	6.818	6.871
5080	2020-4-20 8:00	HT	AMRO	0	44	0	3	13	6.818	6.673
5081	2020-4-21 8:00	HT	AMRO	3	47	0	3	14	6.383	6.155
5082	2020-4-22 8:00	HT	AMRO	10	57	0	3	15	5.263	6.181
5083	2020-4-23 8:00	HT	AMRO	1	58	1	4	16	6.897	6.204
5084	2020-4-24 8:00	HT	AMRO	4	62	0	4	17	6.452	6.764
5085	2020-4-25 8:00	HT	AMRO	10	72	1	5	18	6.944	7.243
5086	2020-4-26 8:00	HT	AMRO	0	72	1	6	19	8.333	7.870
5087	2020-4-27 8:00	HT	AMRO	0	72	0	6	20	8.333	8.258
5088	2020-4-28 8:00	HT	AMRO	2	74	0	6	21	8.108	8.112
5089	2020-4-29 8:00	HT	AMRO	2	76	0	6	22	7.895	7.966
5090	2020-4-30 8:00	HT	AMRO	0	76	0	6	23	7.895	8.333
5091	2020-5-1 8:00	HT	AMRO	0	76	1	7	24	9.211	8.994
5092	2020-5-2 8:00	HT	AMRO	5	81	1	8	25	9.877	9.544
5093										
5094	2020-3-6 8:00	VA	EURO	1	1	0	0			
5095	2020-3-7 8:00	VA	EURO	0	1	0	0			
5096	2020-3-8 8:00	VA	EURO	0	1	0	0			
5097	2020-3-9 8:00	VA	EURO	0	1	0	0			
5098	2020-3-10 8:00	VA	EURO	0	1	0	0			
5099	2020-3-11 8:00	VA	EURO	0	1	0	0			
5100	2020-3-12 8:00	VA	EURO	0	1	0	0			
5101	2020-3-13 8:00	VA	EURO	0	1	0	0			
5102	2020-3-14 8:00	VA	EURO	0	1	0	0			
5103	2020-3-15 8:00	VA	EURO	0	1	0	0			
5104	2020-3-16 8:00	VA	EURO	0	1	0	0			
5105	2020-3-17 8:00	VA	EURO	0	1	0	0			
5106	2020-3-18 8:00	VA	EURO	0	1	0	0			
5107	2020-3-19 8:00	VA	EURO	0	1	0	0			
5108	2020-3-20 8:00	VA	EURO	0	1	0	0			
5109	2020-3-21 8:00	VA	EURO	0	1	0	0			
5110	2020-3-22 8:00	VA	EURO	0	1	0	0			
5111	2020-3-23 8:00	VA	EURO	0	1	0	0			
5112	2020-3-24 8:00	VA	EURO	0	1	0	0			

Table S1 Daily confirmed cases and daily deaths of COVID-19 in 214 nations from WHO

ID	Date and time	Country or region	Continent	Confirmed patients	Cumulative Confirmed patients	Daily dead patients	Daily cumulative dead patients	Lag days	Daily CFR	3DMA CFR
5113	2020-3-25 8:00	VA	EURO	0	1	0	0			
5114	2020-3-26 8:00	VA	EURO	3	4	0	0			
5115	2020-3-27 8:00	VA	EURO	0	4	0	0			
5116	2020-3-28 8:00	VA	EURO	0	4	0	0			
5117	2020-3-29 8:00	VA	EURO	0	4	0	0			
5118	2020-3-30 8:00	VA	EURO	2	6	0	0			
5119	2020-3-31 8:00	VA	EURO	0	6	0	0			
5120	2020-4-1 8:00	VA	EURO	0	6	0	0			
5121	2020-4-2 8:00	VA	EURO	0	6	0	0			
5122	2020-4-3 8:00	VA	EURO	1	7	0	0			
5123	2020-4-4 8:00	VA	EURO	0	7	0	0			
5124	2020-4-5 8:00	VA	EURO	0	7	0	0			
5125	2020-4-6 8:00	VA	EURO	0	7	0	0			
5126	2020-4-7 8:00	VA	EURO	0	7	0	0			
5127	2020-4-8 8:00	VA	EURO	0	7	0	0			
5128	2020-4-9 8:00	VA	EURO	1	8	0	0			
5129	2020-4-10 8:00	VA	EURO	0	8	0	0			
5130	2020-4-11 8:00	VA	EURO	0	8	0	0			
5131	2020-4-12 8:00	VA	EURO	0	8	0	0			
5132	2020-4-13 8:00	VA	EURO	0	8	0	0			
5133	2020-4-14 8:00	VA	EURO	0	8	0	0			
5134	2020-4-15 8:00	VA	EURO	0	8	0	0			
5135	2020-4-16 8:00	VA	EURO	0	8	0	0			
5136	2020-4-17 8:00	VA	EURO	0	8	0	0			
5137	2020-4-18 8:00	VA	EURO	0	8	0	0			
5138	2020-4-19 8:00	VA	EURO	0	8	0	0			
5139	2020-4-20 8:00	VA	EURO	0	8	0	0			
5140	2020-4-21 8:00	VA	EURO	1	9	0	0			
5141	2020-4-22 8:00	VA	EURO	0	9	0	0			
5142	2020-4-23 8:00	VA	EURO	0	9	0	0			
5143	2020-4-24 8:00	VA	EURO	0	9	0	0			
5144	2020-4-25 8:00	VA	EURO	0	9	0	0			
5145	2020-4-26 8:00	VA	EURO	0	9	0	0			
5146	2020-4-27 8:00	VA	EURO	0	9	0	0			
5147	2020-4-28 8:00	VA	EURO	0	9	0	0			
5148	2020-4-29 8:00	VA	EURO	1	10	0	0			

Table S1 Daily confirmed cases and daily deaths of COVID-19 in 214 nations from WHO

ID	Date and time	Country or region	Continent	Confirmed patients	Cumulative Confirmed patients	Daily dead patients	Daily cumulative dead patients	Lag days	Daily CFR	3DMA CFR
5149	2020-4-30 8:00	VA	EURO	0	10	0	0			
5150	2020-5-1 8:00	VA	EURO	1	11	0	0			
5151	2020-5-2 8:00	VA	EURO	0	11	0	0			
5152										
5153	2020-3-12 8:00	HN	AMRO	2	2	0	0			
5154	2020-3-13 8:00	HN	AMRO	0	2	0	0			
5155	2020-3-14 8:00	HN	AMRO	0	2	0	0			
5156	2020-3-15 8:00	HN	AMRO	0	2	0	0			
5157	2020-3-16 8:00	HN	AMRO	0	2	0	0			
5158	2020-3-17 8:00	HN	AMRO	6	8	0	0			
5159	2020-3-18 8:00	HN	AMRO	1	9	0	0			
5160	2020-3-19 8:00	HN	AMRO	3	12	0	0			
5161	2020-3-20 8:00	HN	AMRO	12	24	0	0			
5162	2020-3-21 8:00	HN	AMRO	0	24	0	0			
5163	2020-3-22 8:00	HN	AMRO	0	24	0	0			
5164	2020-3-23 8:00	HN	AMRO	2	26	0	0			
5165	2020-3-24 8:00	HN	AMRO	4	30	0	0			
5166	2020-3-25 8:00	HN	AMRO	0	30	0	0			
5167	2020-3-26 8:00	HN	AMRO	22	52	1	1	0	1.923	1.923
5168	2020-3-27 8:00	HN	AMRO	0	52	0	1	1	1.923	1.780
5169	2020-3-28 8:00	HN	AMRO	15	67	0	1	2	1.493	1.636
5170	2020-3-29 8:00	HN	AMRO	0	67	0	1	3	1.493	1.601
5171	2020-3-30 8:00	HN	AMRO	43	110	1	2	4	1.818	1.583
5172	2020-3-31 8:00	HN	AMRO	29	139	0	2	5	1.439	1.565
5173	2020-4-1 8:00	HN	AMRO	0	139	0	2	6	1.439	2.897
5174	2020-4-2 8:00	HN	AMRO	33	172	8	10	7	5.814	4.548
5175	2020-4-3 8:00	HN	AMRO	47	219	4	14	8	6.393	6.321
5176	2020-4-4 8:00	HN	AMRO	3	222	1	15	9	6.757	6.277
5177	2020-4-5 8:00	HN	AMRO	42	264	0	15	10	5.682	6.883
5178	2020-4-6 8:00	HN	AMRO	4	268	7	22	11	8.209	7.091
5179	2020-4-7 8:00	HN	AMRO	30	298	0	22	12	7.383	7.602
5180	2020-4-8 8:00	HN	AMRO	7	305	0	22	13	7.213	7.216
5181	2020-4-9 8:00	HN	AMRO	7	312	0	22	14	7.051	6.990
5182	2020-4-10 8:00	HN	AMRO	31	343	1	23	15	6.706	6.593
5183	2020-4-11 8:00	HN	AMRO	39	382	0	23	16	6.021	6.283
5184	2020-4-12 8:00	HN	AMRO	10	392	1	24	17	6.122	6.168

Table S1 Daily confirmed cases and daily deaths of COVID-19 in 214 nations from WHO

ID	Date and time	Country or region	Continent	Confirmed patients	Cumulative Confirmed patients	Daily dead patients	Daily cumulative dead patients	Lag days	Daily CFR	3DMA CFR
5185	2020-4-13 8:00	HN	AMRO	1	393	1	25	18	6.361	6.260
5186	2020-4-14 8:00	HN	AMRO	4	397	0	25	19	6.297	6.349
5187	2020-4-15 8:00	HN	AMRO	10	407	1	26	20	6.388	6.695
5188	2020-4-16 8:00	HN	AMRO	12	419	5	31	21	7.399	7.334
5189	2020-4-17 8:00	HN	AMRO	7	426	4	35	22	8.216	8.297
5190	2020-4-18 8:00	HN	AMRO	16	442	6	41	23	9.276	9.186
5191	2020-4-19 8:00	HN	AMRO	15	457	5	46	24	10.066	9.696
5192	2020-4-20 8:00	HN	AMRO	15	472	0	46	25	9.746	9.818
5193	2020-4-21 8:00	HN	AMRO	5	477	0	46	26	9.644	9.567
5194	2020-4-22 8:00	HN	AMRO	17	494	0	46	27	9.312	9.325
5195	2020-4-23 8:00	HN	AMRO	16	510	0	46	28	9.020	9.129
5196	2020-4-24 8:00	HN	AMRO	9	519	1	47	29	9.056	8.813
5197	2020-4-25 8:00	HN	AMRO	43	562	0	47	30	8.363	8.908
5198	2020-4-26 8:00	HN	AMRO	29	591	8	55	31	9.306	9.026
5199	2020-4-27 8:00	HN	AMRO	36	627	4	59	32	9.410	9.315
5200	2020-4-28 8:00	HN	AMRO	34	661	2	61	33	9.228	9.252
5201	2020-4-29 8:00	HN	AMRO	41	702	3	64	34	9.117	9.096
5202	2020-4-30 8:00	HN	AMRO	36	738	2	66	35	8.943	9.090
5203	2020-5-1 8:00	HN	AMRO	33	771	5	71	36	9.209	9.160
5204	2020-5-2 8:00	HN	AMRO	33	804	4	75	37	9.328	9.269
5205										
5206	2020-3-4 8:00	HU	EURO	2	2	0	0			
5207	2020-3-5 8:00	HU	EURO	0	2	0	0			
5208	2020-3-6 8:00	HU	EURO	2	4	0	0			
5209	2020-3-7 8:00	HU	EURO	3	7	0	0			
5210	2020-3-8 8:00	HU	EURO	1	8	0	0			
5211	2020-3-9 8:00	HU	EURO	1	9	0	0			
5212	2020-3-10 8:00	HU	EURO	3	12	0	0			
5213	2020-3-11 8:00	HU	EURO	1	13	0	0			
5214	2020-3-12 8:00	HU	EURO	0	13	0	0			
5215	2020-3-13 8:00	HU	EURO	6	19	0	0			
5216	2020-3-14 8:00	HU	EURO	6	25	0	0			
5217	2020-3-15 8:00	HU	EURO	7	32	1	1	0	3.125	2.845
5218	2020-3-16 8:00	HU	EURO	7	39	0	1	1	2.564	2.563
5219	2020-3-17 8:00	HU	EURO	11	50	0	1	2	2.000	2.096
5220	2020-3-18 8:00	HU	EURO	8	58	0	1	3	1.724	1.698

Table S1 Daily confirmed cases and daily deaths of COVID-19 in 214 nations from WHO

ID	Date and time	Country or region	Continent	Confirmed patients	Cumulative Confirmed patients	Daily dead patients	Daily cumulative dead patients	Lag days	Daily CFR	3DMA CFR
5221	2020-3-19 8:00	HU	EURO	15	73	0	1	4	1.370	2.600
5222	2020-3-20 8:00	HU	EURO	12	85	3	4	5	4.706	3.043
5223	2020-3-21 8:00	HU	EURO	46	131	0	4	6	3.053	3.604
5224	2020-3-22 8:00	HU	EURO	0	131	0	4	7	3.053	3.433
5225	2020-3-23 8:00	HU	EURO	36	167	3	7	8	4.192	3.841
5226	2020-3-24 8:00	HU	EURO	20	187	1	8	9	4.278	4.298
5227	2020-3-25 8:00	HU	EURO	39	226	2	10	10	4.425	4.178
5228	2020-3-26 8:00	HU	EURO	35	261	0	10	11	3.831	3.863
5229	2020-3-27 8:00	HU	EURO	39	300	0	10	12	3.333	3.457
5230	2020-3-28 8:00	HU	EURO	43	343	1	11	13	3.207	3.242
5231	2020-3-29 8:00	HU	EURO	65	408	2	13	14	3.186	3.250
5232	2020-3-30 8:00	HU	EURO	39	447	2	15	15	3.356	3.299
5233	2020-3-31 8:00	HU	EURO	0	447	0	15	16	3.356	3.321
5234	2020-4-1 8:00	HU	EURO	45	492	1	16	17	3.252	3.472
5235	2020-4-2 8:00	HU	EURO	33	525	4	20	18	3.810	3.550
5236	2020-4-3 8:00	HU	EURO	60	585	1	21	19	3.590	4.040
5237	2020-4-4 8:00	HU	EURO	93	678	11	32	20	4.720	4.316
5238	2020-4-5 8:00	HU	EURO	55	733	2	34	21	4.638	4.822
5239	2020-4-6 8:00	HU	EURO	11	744	4	38	22	5.108	5.166
5240	2020-4-7 8:00	HU	EURO	73	817	9	47	23	5.753	5.780
5241	2020-4-8 8:00	HU	EURO	78	895	11	58	24	6.480	6.323
5242	2020-4-9 8:00	HU	EURO	85	980	8	66	25	6.735	6.562
5243	2020-4-10 8:00	HU	EURO	210	1190	11	77	26	6.471	6.565
5244	2020-4-11 8:00	HU	EURO	120	1310	8	85	27	6.489	6.660
5245	2020-4-12 8:00	HU	EURO	100	1410	14	99	28	7.021	6.995
5246	2020-4-13 8:00	HU	EURO	48	1458	10	109	29	7.476	7.522
5247	2020-4-14 8:00	HU	EURO	54	1512	13	122	30	8.069	8.010
5248	2020-4-15 8:00	HU	EURO	67	1579	12	134	31	8.486	8.384
5249	2020-4-16 8:00	HU	EURO	73	1652	8	142	32	8.596	8.644
5250	2020-4-17 8:00	HU	EURO	111	1763	14	156	33	8.849	8.941
5251	2020-4-18 8:00	HU	EURO	71	1834	16	172	34	9.378	9.364
5252	2020-4-19 8:00	HU	EURO	82	1916	17	189	35	9.864	9.758
5253	2020-4-20 8:00	HU	EURO	68	1984	10	199	36	10.030	10.016
5254	2020-4-21 8:00	HU	EURO	114	2098	14	213	37	10.153	10.187
5255	2020-4-22 8:00	HU	EURO	70	2168	12	225	38	10.378	10.127
5256	2020-4-23 8:00	HU	EURO	116	2284	0	225	39	9.851	10.240

Table S1 Daily confirmed cases and daily deaths of COVID-19 in 214 nations from WHO

ID	Date and time	Country or region	Continent	Confirmed patients	Cumulative Confirmed patients	Daily dead patients	Daily cumulative dead patients	Lag days	Daily CFR	3DMA CFR
5257	2020-4-24 8:00	HU	EURO	99	2383	25	250	40	10.491	10.356
5258	2020-4-25 8:00	HU	EURO	60	2443	12	262	41	10.725	10.698
5259	2020-4-26 8:00	HU	EURO	57	2500	10	272	42	10.880	10.815
5260	2020-4-27 8:00	HU	EURO	83	2583	8	280	43	10.840	10.902
5261	2020-4-28 8:00	HU	EURO	66	2649	11	291	44	10.985	10.942
5262	2020-4-29 8:00	HU	EURO	78	2727	9	300	45	11.001	11.077
5263	2020-4-30 8:00	HU	EURO	48	2775	12	312	46	11.243	11.175
5264	2020-5-1 8:00	HU	EURO	88	2863	11	323	47	11.282	11.304
5265	2020-5-2 8:00	HU	EURO	79	2942	12	335	48	11.387	11.334
5266										
5267	2020-3-1 8:00	IS	EURO	2	2	0	0			
5268	2020-3-2 8:00	IS	EURO	7	9	0	0			
5269	2020-3-3 8:00	IS	EURO	7	16	0	0			
5270	2020-3-4 8:00	IS	EURO	10	26	0	0			
5271	2020-3-5 8:00	IS	EURO	0	26	0	0			
5272	2020-3-6 8:00	IS	EURO	2	28	0	0			
5273	2020-3-7 8:00	IS	EURO	17	45	0	0			
5274	2020-3-8 8:00	IS	EURO	10	55	0	0			
5275	2020-3-9 8:00	IS	EURO	0	55	0	0			
5276	2020-3-10 8:00	IS	EURO	6	61	0	0			
5277	2020-3-11 8:00	IS	EURO	0	61	0	0			
5278	2020-3-12 8:00	IS	EURO	0	61	0	0			
5279	2020-3-13 8:00	IS	EURO	56	117	0	0			
5280	2020-3-14 8:00	IS	EURO	21	138	0	0			
5281	2020-3-15 8:00	IS	EURO	0	138	0	0			
5282	2020-3-16 8:00	IS	EURO	42	180	0	0			
5283	2020-3-17 8:00	IS	EURO	45	225	0	0			
5284	2020-3-18 8:00	IS	EURO	25	250	0	0			
5285	2020-3-19 8:00	IS	EURO	80	330	0	0			
5286	2020-3-20 8:00	IS	EURO	79	409	1	1	0	0.244	0.228
5287	2020-3-21 8:00	IS	EURO	64	473	0	1	1	0.211	0.222
5288	2020-3-22 8:00	IS	EURO	0	473	0	1	2	0.211	0.200
5289	2020-3-23 8:00	IS	EURO	95	568	0	1	3	0.176	0.243
5290	2020-3-24 8:00	IS	EURO	20	588	1	2	4	0.340	0.275
5291	2020-3-25 8:00	IS	EURO	60	648	0	2	5	0.309	0.307
5292	2020-3-26 8:00	IS	EURO	89	737	0	2	6	0.271	0.276

Table S1 Daily confirmed cases and daily deaths of COVID-19 in 214 nations from WHO

ID	Date and time	Country or region	Continent	Confirmed patients	Cumulative Confirmed patients	Daily dead patients	Daily cumulative dead patients	Lag days	Daily CFR	3DMA CFR
5293	2020-3-27 8:00	IS	EURO	65	802	0	2	7	0.249	0.248
5294	2020-3-28 8:00	IS	EURO	88	890	0	2	8	0.225	0.227
5295	2020-3-29 8:00	IS	EURO	73	963	0	2	9	0.208	0.209
5296	2020-3-30 8:00	IS	EURO	57	1020	0	2	10	0.196	0.196
5297	2020-3-31 8:00	IS	EURO	66	1086	0	2	11	0.184	0.185
5298	2020-4-1 8:00	IS	EURO	49	1135	0	2	12	0.176	0.175
5299	2020-4-2 8:00	IS	EURO	85	1220	0	2	13	0.164	0.214
5300	2020-4-3 8:00	IS	EURO	99	1319	2	4	14	0.303	0.253
5301	2020-4-4 8:00	IS	EURO	45	1364	0	4	15	0.293	0.293
5302	2020-4-5 8:00	IS	EURO	53	1417	0	4	16	0.282	0.282
5303	2020-4-6 8:00	IS	EURO	69	1486	0	4	17	0.269	0.312
5304	2020-4-7 8:00	IS	EURO	76	1562	2	6	18	0.384	0.344
5305	2020-4-8 8:00	IS	EURO	24	1586	0	6	19	0.378	0.378
5306	2020-4-9 8:00	IS	EURO	30	1616	0	6	20	0.371	0.371
5307	2020-4-10 8:00	IS	EURO	32	1648	0	6	21	0.364	0.384
5308	2020-4-11 8:00	IS	EURO	27	1675	1	7	22	0.418	0.419
5309	2020-4-12 8:00	IS	EURO	14	1689	1	8	23	0.474	0.454
5310	2020-4-13 8:00	IS	EURO	12	1701	0	8	24	0.470	0.471
5311	2020-4-14 8:00	IS	EURO	10	1711	0	8	25	0.468	0.468
5312	2020-4-15 8:00	IS	EURO	9	1720	0	8	26	0.465	0.465
5313	2020-4-16 8:00	IS	EURO	7	1727	0	8	27	0.463	0.463
5314	2020-4-17 8:00	IS	EURO	12	1739	0	8	28	0.460	0.460
5315	2020-4-18 8:00	IS	EURO	15	1754	0	8	29	0.456	0.476
5316	2020-4-19 8:00	IS	EURO	6	1760	1	9	30	0.511	0.492
5317	2020-4-20 8:00	IS	EURO	11	1771	0	9	31	0.508	0.528
5318	2020-4-21 8:00	IS	EURO	2	1773	1	10	32	0.564	0.545
5319	2020-4-22 8:00	IS	EURO	5	1778	0	10	33	0.562	0.562
5320	2020-4-23 8:00	IS	EURO	7	1785	0	10	34	0.560	0.561
5321	2020-4-24 8:00	IS	EURO	4	1789	0	10	35	0.559	0.559
5322	2020-4-25 8:00	IS	EURO	0	1789	0	10	36	0.559	0.559
5323	2020-4-26 8:00	IS	EURO	1	1790	0	10	37	0.559	0.559
5324	2020-4-27 8:00	IS	EURO	2	1792	0	10	38	0.558	0.558
5325	2020-4-28 8:00	IS	EURO	0	1792	0	10	39	0.558	0.558
5326	2020-4-29 8:00	IS	EURO	3	1795	0	10	40	0.557	0.557
5327	2020-4-30 8:00	IS	EURO	2	1797	0	10	41	0.556	0.557
5328	2020-5-1 8:00	IS	EURO	0	1797	0	10	42	0.556	0.556

Table S1 Daily confirmed cases and daily deaths of COVID-19 in 214 nations from WHO

ID	Date and time	Country or region	Continent	Confirmed patients	Cumulative Confirmed patients	Daily dead patients	Daily cumulative dead patients	Lag days	Daily CFR	3DMA CFR
5329	2020-5-2 8:00	IS	EURO	1	1798	0	10	43	0.556	0.556
5330										
5331	2020-1-30 8:00	IN	SEARO	1	1	0	0			
5332	2020-1-31 8:00	IN	SEARO	0	1	0	0			
5333	2020-2-1 8:00	IN	SEARO	0	1	0	0			
5334	2020-2-2 8:00	IN	SEARO	1	2	0	0			
5335	2020-2-3 8:00	IN	SEARO	1	3	0	0			
5336	2020-2-4 8:00	IN	SEARO	0	3	0	0			
5337	2020-2-5 8:00	IN	SEARO	0	3	0	0			
5338	2020-2-6 8:00	IN	SEARO	0	3	0	0			
5339	2020-2-7 8:00	IN	SEARO	0	3	0	0			
5340	2020-2-8 8:00	IN	SEARO	0	3	0	0			
5341	2020-2-9 8:00	IN	SEARO	0	3	0	0			
5342	2020-2-10 8:00	IN	SEARO	0	3	0	0			
5343	2020-2-11 8:00	IN	SEARO	0	3	0	0			
5344	2020-2-12 8:00	IN	SEARO	0	3	0	0			
5345	2020-2-13 8:00	IN	SEARO	0	3	0	0			
5346	2020-2-14 8:00	IN	SEARO	0	3	0	0			
5347	2020-2-15 8:00	IN	SEARO	0	3	0	0			
5348	2020-2-16 8:00	IN	SEARO	0	3	0	0			
5349	2020-2-17 8:00	IN	SEARO	0	3	0	0			
5350	2020-2-18 8:00	IN	SEARO	0	3	0	0			
5351	2020-2-19 8:00	IN	SEARO	0	3	0	0			
5352	2020-2-20 8:00	IN	SEARO	0	3	0	0			
5353	2020-2-21 8:00	IN	SEARO	0	3	0	0			
5354	2020-2-22 8:00	IN	SEARO	0	3	0	0			
5355	2020-2-23 8:00	IN	SEARO	0	3	0	0			
5356	2020-2-24 8:00	IN	SEARO	0	3	0	0			
5357	2020-2-25 8:00	IN	SEARO	0	3	0	0			
5358	2020-2-26 8:00	IN	SEARO	0	3	0	0			
5359	2020-2-27 8:00	IN	SEARO	0	3	0	0			
5360	2020-2-28 8:00	IN	SEARO	0	3	0	0			
5361	2020-2-29 8:00	IN	SEARO	0	3	0	0			
5362	2020-3-1 8:00	IN	SEARO	0	3	0	0			
5363	2020-3-2 8:00	IN	SEARO	2	5	0	0			
5364	2020-3-3 8:00	IN	SEARO	0	5	0	0			

Table S1 Daily confirmed cases and daily deaths of COVID-19 in 214 nations from WHO

ID	Date and time	Country or region	Continent	Confirmed patients	Cumulative Confirmed patients	Daily dead patients	Daily cumulative dead patients	Lag days	Daily CFR	3DMA CFR
5365	2020-3-4 8:00	IN	SEARO	22	27	0	0			
5366	2020-3-5 8:00	IN	SEARO	2	29	0	0			
5367	2020-3-6 8:00	IN	SEARO	2	31	0	0			
5368	2020-3-7 8:00	IN	SEARO	3	34	0	0			
5369	2020-3-8 8:00	IN	SEARO	5	39	0	0			
5370	2020-3-9 8:00	IN	SEARO	5	44	0	0			
5371	2020-3-10 8:00	IN	SEARO	6	50	0	0			
5372	2020-3-11 8:00	IN	SEARO	10	60	0	0			
5373	2020-3-12 8:00	IN	SEARO	14	74	0	0			
5374	2020-3-13 8:00	IN	SEARO	7	81	1	1	0	1.235	1.808
5375	2020-3-14 8:00	IN	SEARO	3	84	1	2	1	2.381	1.828
5376	2020-3-15 8:00	IN	SEARO	23	107	0	2	2	1.869	2.001
5377	2020-3-16 8:00	IN	SEARO	7	114	0	2	3	1.754	1.938
5378	2020-3-17 8:00	IN	SEARO	23	137	1	3	4	2.190	1.977
5379	2020-3-18 8:00	IN	SEARO	14	151	0	3	5	1.987	2.076
5380	2020-3-19 8:00	IN	SEARO	44	195	1	4	6	2.051	2.030
5381	2020-3-20 8:00	IN	SEARO	0	195	0	4	7	2.051	1.839
5382	2020-3-21 8:00	IN	SEARO	88	283	0	4	8	1.413	1.644
5383	2020-3-22 8:00	IN	SEARO	58	341	1	5	9	1.466	1.651
5384	2020-3-23 8:00	IN	SEARO	93	434	4	9	10	2.074	1.758
5385	2020-3-24 8:00	IN	SEARO	85	519	0	9	11	1.734	1.819
5386	2020-3-25 8:00	IN	SEARO	87	606	1	10	12	1.650	1.796
5387	2020-3-26 8:00	IN	SEARO	43	649	3	13	13	2.003	2.000
5388	2020-3-27 8:00	IN	SEARO	75	724	4	17	14	2.348	2.233
5389	2020-3-28 8:00	IN	SEARO	0	724	0	17	15	2.348	2.417
5390	2020-3-29 8:00	IN	SEARO	255	979	8	25	16	2.554	2.536
5391	2020-3-30 8:00	IN	SEARO	92	1071	4	29	17	2.708	2.606
5392	2020-3-31 8:00	IN	SEARO	180	1251	3	32	18	2.558	2.529
5393	2020-4-1 8:00	IN	SEARO	385	1636	6	38	19	2.323	2.475
5394	2020-4-2 8:00	IN	SEARO	329	1965	12	50	20	2.545	2.434
5395	2020-4-3 8:00	IN	SEARO	336	2301	6	56	21	2.434	2.440
5396	2020-4-4 8:00	IN	SEARO	601	2902	12	68	22	2.343	2.353
5397	2020-4-5 8:00	IN	SEARO	472	3374	9	77	23	2.282	2.435
5398	2020-4-6 8:00	IN	SEARO	693	4067	32	109	24	2.680	2.514
5399	2020-4-7 8:00	IN	SEARO	354	4421	5	114	25	2.579	2.695
5400	2020-4-8 8:00	IN	SEARO	853	5274	35	149	26	2.825	2.762

Table S1 Daily confirmed cases and daily deaths of COVID-19 in 214 nations from WHO

ID	Date and time	Country or region	Continent	Confirmed patients	Cumulative Confirmed patients	Daily dead patients	Daily cumulative dead patients	Lag days	Daily CFR	3DMA CFR
5401	2020-4-9 8:00	IN	SEARO	591	5865	20	169	27	2.882	2.937
5402	2020-4-10 8:00	IN	SEARO	547	6412	30	199	28	3.104	3.065
5403	2020-4-11 8:00	IN	SEARO	1035	7447	40	239	29	3.209	3.193
5404	2020-4-12 8:00	IN	SEARO	909	8356	34	273	30	3.267	3.281
5405	2020-4-13 8:00	IN	SEARO	796	9152	35	308	31	3.365	3.301
5406	2020-4-14 8:00	IN	SEARO	1211	10363	31	339	32	3.271	3.311
5407	2020-4-15 8:00	IN	SEARO	1076	11439	38	377	33	3.296	3.304
5408	2020-4-16 8:00	IN	SEARO	941	12380	37	414	34	3.344	3.301
5409	2020-4-17 8:00	IN	SEARO	1007	13387	23	437	35	3.264	3.316
5410	2020-4-18 8:00	IN	SEARO	991	14378	43	480	36	3.338	3.277
5411	2020-4-19 8:00	IN	SEARO	1334	15712	27	507	37	3.227	3.237
5412	2020-4-20 8:00	IN	SEARO	1553	17265	36	543	38	3.145	3.181
5413	2020-4-21 8:00	IN	SEARO	1336	18601	47	590	39	3.172	3.173
5414	2020-4-22 8:00	IN	SEARO	1383	19984	50	640	40	3.203	3.186
5415	2020-4-23 8:00	IN	SEARO	1409	21393	41	681	41	3.183	3.166
5416	2020-4-24 8:00	IN	SEARO	1684	23077	37	718	42	3.111	3.152
5417	2020-4-25 8:00	IN	SEARO	1429	24506	57	775	43	3.162	3.128
5418	2020-4-26 8:00	IN	SEARO	1990	26496	49	824	44	3.110	3.133
5419	2020-4-27 8:00	IN	SEARO	1396	27892	48	872	45	3.126	3.136
5420	2020-4-28 8:00	IN	SEARO	1543	29435	62	934	46	3.173	3.171
5421	2020-4-29 8:00	IN	SEARO	1897	31332	73	1007	47	3.214	3.212
5422	2020-4-30 8:00	IN	SEARO	1718	33050	67	1074	48	3.250	3.246
5423	2020-5-1 8:00	IN	SEARO	1993	35043	73	1147	49	3.273	3.262
5424	2020-5-2 8:00	IN	SEARO	2293	37336	71	1218	50	3.262	3.268
5425										
5426	2020-3-2 8:00	ID	SEARO	2	2	0	0			
5427	2020-3-3 8:00	ID	SEARO	0	2	0	0			
5428	2020-3-4 8:00	ID	SEARO	0	2	0	0			
5429	2020-3-5 8:00	ID	SEARO	0	2	0	0			
5430	2020-3-6 8:00	ID	SEARO	2	4	0	0			
5431	2020-3-7 8:00	ID	SEARO	0	4	0	0			
5432	2020-3-8 8:00	ID	SEARO	2	6	0	0			
5433	2020-3-9 8:00	ID	SEARO	0	6	0	0			
5434	2020-3-10 8:00	ID	SEARO	13	19	0	0			
5435	2020-3-11 8:00	ID	SEARO	8	27	1	1	0	3.704	3.322
5436	2020-3-12 8:00	ID	SEARO	7	34	0	1	1	2.941	4.147

Table S1 Daily confirmed cases and daily deaths of COVID-19 in 214 nations from WHO

ID	Date and time	Country or region	Continent	Confirmed patients	Cumulative Confirmed patients	Daily dead patients	Daily cumulative dead patients	Lag days	Daily CFR	3DMA CFR
5437	2020-3-13 8:00	ID	SEARO	35	69	3	4	2	5.797	4.845
5438	2020-3-14 8:00	ID	SEARO	0	69	0	4	3	5.797	5.004
5439	2020-3-15 8:00	ID	SEARO	48	117	0	4	4	3.419	4.041
5440	2020-3-16 8:00	ID	SEARO	55	172	1	5	5	2.907	3.078
5441	2020-3-17 8:00	ID	SEARO	0	172	0	5	6	2.907	4.728
5442	2020-3-18 8:00	ID	SEARO	55	227	14	19	7	8.370	6.456
5443	2020-3-19 8:00	ID	SEARO	82	309	6	25	8	8.091	8.378
5444	2020-3-20 8:00	ID	SEARO	60	369	7	32	9	8.672	8.402
5445	2020-3-21 8:00	ID	SEARO	81	450	6	38	10	8.444	8.818
5446	2020-3-22 8:00	ID	SEARO	64	514	10	48	11	9.339	8.749
5447	2020-3-23 8:00	ID	SEARO	65	579	1	49	12	8.463	8.755
5448	2020-3-24 8:00	ID	SEARO	0	579	0	49	13	8.463	8.089
5449	2020-3-25 8:00	ID	SEARO	211	790	9	58	14	7.342	8.180
5450	2020-3-26 8:00	ID	SEARO	103	893	20	78	15	8.735	8.131
5451	2020-3-27 8:00	ID	SEARO	153	1046	9	87	16	8.317	8.456
5452	2020-3-28 8:00	ID	SEARO	0	1046	0	87	17	8.317	8.502
5453	2020-3-29 8:00	ID	SEARO	239	1285	27	114	18	8.872	8.606
5454	2020-3-30 8:00	ID	SEARO	129	1414	8	122	19	8.628	8.800
5455	2020-3-31 8:00	ID	SEARO	114	1528	14	136	20	8.901	8.963
5456	2020-4-1 8:00	ID	SEARO	149	1677	21	157	21	9.362	9.253
5457	2020-4-2 8:00	ID	SEARO	113	1790	13	170	22	9.497	9.324
5458	2020-4-3 8:00	ID	SEARO	196	1986	11	181	23	9.114	9.247
5459	2020-4-4 8:00	ID	SEARO	106	2092	10	191	24	9.130	8.985
5460	2020-4-5 8:00	ID	SEARO	181	2273	7	198	25	8.711	8.744
5461	2020-4-6 8:00	ID	SEARO	218	2491	11	209	26	8.390	8.391
5462	2020-4-7 8:00	ID	SEARO	247	2738	12	221	27	8.072	8.194
5463	2020-4-8 8:00	ID	SEARO	218	2956	19	240	28	8.119	8.231
5464	2020-4-9 8:00	ID	SEARO	337	3293	40	280	29	8.503	8.445
5465	2020-4-10 8:00	ID	SEARO	219	3512	26	306	30	8.713	8.576
5466	2020-4-11 8:00	ID	SEARO	330	3842	21	327	31	8.511	8.673
5467	2020-4-12 8:00	ID	SEARO	399	4241	46	373	32	8.795	8.687
5468	2020-4-13 8:00	ID	SEARO	316	4557	26	399	33	8.756	9.012
5469	2020-4-14 8:00	ID	SEARO	282	4839	60	459	34	9.485	9.124
5470	2020-4-15 8:00	ID	SEARO	297	5136	10	469	35	9.132	9.203
5471	2020-4-16 8:00	ID	SEARO	380	5516	27	496	36	8.992	8.968
5472	2020-4-17 8:00	ID	SEARO	407	5923	24	520	37	8.779	8.778

Table S1 Daily confirmed cases and daily deaths of COVID-19 in 214 nations from WHO

ID	Date and time	Country or region	Continent	Confirmed patients	Cumulative Confirmed patients	Daily dead patients	Daily cumulative dead patients	Lag days	Daily CFR	3DMA CFR
5473	2020-4-18 8:00	ID	SEARO	325	6248	15	535	38	8.563	8.731
5474	2020-4-19 8:00	ID	SEARO	327	6575	47	582	39	8.852	8.714
5475	2020-4-20 8:00	ID	SEARO	185	6760	8	590	40	8.728	8.738
5476	2020-4-21 8:00	ID	SEARO	375	7135	26	616	41	8.633	8.641
5477	2020-4-22 8:00	ID	SEARO	283	7418	19	635	42	8.560	8.505
5478	2020-4-23 8:00	ID	SEARO	357	7775	12	647	43	8.322	8.424
5479	2020-4-24 8:00	ID	SEARO	436	8211	42	689	44	8.391	8.359
5480	2020-4-25 8:00	ID	SEARO	396	8607	31	720	45	8.365	8.374
5481	2020-4-26 8:00	ID	SEARO	275	8882	23	743	46	8.365	8.380
5482	2020-4-27 8:00	ID	SEARO	214	9096	22	765	47	8.410	8.301
5483	2020-4-28 8:00	ID	SEARO	415	9511	8	773	48	8.127	8.187
5484	2020-4-29 8:00	ID	SEARO	260	9771	11	784	49	8.024	7.993
5485	2020-4-30 8:00	ID	SEARO	347	10118	8	792	50	7.828	7.811
5486	2020-5-1 8:00	ID	SEARO	433	10551	8	800	51	7.582	7.664
5487	2020-5-2 8:00	ID	SEARO	0	10551	0	800	52	7.582	7.582
5488										
5489	2020-2-5 8:00	DP		10	10	0	0			
5490	2020-2-6 8:00	DP		10	20	0	0			
5491	2020-2-7 8:00	DP		41	61	0	0			
5492	2020-2-8 8:00	DP		3	64	0	0			
5493	2020-2-9 8:00	DP		6	70	0	0			
5494	2020-2-10 8:00	DP		65	135	0	0			
5495	2020-2-11 8:00	DP		0	135	0	0			
5496	2020-2-12 8:00	DP		39	174	0	0			
5497	2020-2-13 8:00	DP		44	218	0	0			
5498	2020-2-14 8:00	DP		0	218	0	0			
5499	2020-2-15 8:00	DP		67	285	0	0			
5500	2020-2-16 8:00	DP		70	355	0	0			
5501	2020-2-17 8:00	DP		99	454	0	0			
5502	2020-2-18 8:00	DP		88	542	0	0			
5503	2020-2-19 8:00	DP		79	621	0	0			
5504	2020-2-20 8:00	DP		13	634	2	2	0	0.315	0.315
5505	2020-2-21 8:00	DP		0	634	0	2	1	0.315	0.315
5506	2020-2-22 8:00	DP		0	634	0	2	2	0.315	0.355
5507	2020-2-23 8:00	DP		57	691	1	3	3	0.434	0.395
5508	2020-2-24 8:00	DP		0	691	0	3	4	0.434	0.482

Table S1 Daily confirmed cases and daily deaths of COVID-19 in 214 nations from WHO

ID	Date and time	Country or region	Continent	Confirmed patients	Cumulative Confirmed patients	Daily dead patients	Daily cumulative dead patients	Lag days	Daily CFR	3DMA CFR
5509	2020-2-25 8:00	DP		0	691	1	4	5	0.579	0.530
5510	2020-2-26 8:00	DP		4	695	0	4	6	0.576	0.577
5511	2020-2-27 8:00	DP		0	695	0	4	7	0.576	0.671
5512	2020-2-28 8:00	DP		0	695	2	6	8	0.863	0.767
5513	2020-2-29 8:00	DP		0	695	0	6	9	0.863	0.863
5514	2020-3-1 8:00	DP		1	696	0	6	10	0.862	0.862
5515	2020-3-2 8:00	DP		0	696	0	6	11	0.862	0.862
5516	2020-3-3 8:00	DP		0	696	0	6	12	0.862	0.862
5517	2020-3-4 8:00	DP		0	696	0	6	13	0.862	0.862
5518	2020-3-5 8:00	DP		0	696	0	6	14	0.862	0.862
5519	2020-3-6 8:00	DP		0	696	0	6	15	0.862	0.862
5520	2020-3-7 8:00	DP		0	696	0	6	16	0.862	0.910
5521	2020-3-8 8:00	DP		0	696	1	7	17	1.006	0.958
5522	2020-3-9 8:00	DP		0	696	0	7	18	1.006	1.006
5523	2020-3-10 8:00	DP		0	696	0	7	19	1.006	1.006
5524	2020-3-11 8:00	DP		0	696	0	7	20	1.006	1.006
5525	2020-3-12 8:00	DP		0	696	0	7	21	1.006	1.005
5526	2020-3-13 8:00	DP		1	697	0	7	22	1.004	1.005
5527	2020-3-14 8:00	DP		0	697	0	7	23	1.004	0.997
5528	2020-3-15 8:00	DP		15	712	0	7	24	0.983	0.990
5529	2020-3-16 8:00	DP		0	712	0	7	25	0.983	0.983
5530	2020-3-17 8:00	DP		0	712	0	7	26	0.983	0.983
5531	2020-3-18 8:00	DP		0	712	0	7	27	0.983	0.983
5532	2020-3-19 8:00	DP		0	712	0	7	28	0.983	1.030
5533	2020-3-20 8:00	DP		0	712	1	8	29	1.124	1.077
5534	2020-3-21 8:00	DP		0	712	0	8	30	1.124	1.124
5535	2020-3-22 8:00	DP		0	712	0	8	31	1.124	1.124
5536	2020-3-23 8:00	DP		0	712	0	8	32	1.124	1.217
5537	2020-3-24 8:00	DP		0	712	2	10	33	1.404	1.311
5538	2020-3-25 8:00	DP		0	712	0	10	34	1.404	1.404
5539	2020-3-26 8:00	DP		0	712	0	10	35	1.404	1.404
5540	2020-3-27 8:00	DP		0	712	0	10	36	1.404	1.404
5541	2020-3-28 8:00	DP		0	712	0	10	37	1.404	1.404
5542	2020-3-29 8:00	DP		0	712	0	10	38	1.404	1.404
5543	2020-3-30 8:00	DP		0	712	0	10	39	1.404	1.451
5544	2020-3-31 8:00	DP		0	712	1	11	40	1.545	1.498

Table S1 Daily confirmed cases and daily deaths of COVID-19 in 214 nations from WHO

ID	Date and time	Country or region	Continent	Confirmed patients	Cumulative Confirmed patients	Daily dead patients	Daily cumulative dead patients	Lag days	Daily CFR	3DMA CFR
5545	2020-4-1 8:00	DP		0	712	0	11	41	1.545	1.545
5546	2020-4-2 8:00	DP		0	712	0	11	42	1.545	1.545
5547	2020-4-3 8:00	DP		0	712	0	11	43	1.545	1.545
5548	2020-4-4 8:00	DP		0	712	0	11	44	1.545	1.545
5549	2020-4-5 8:00	DP		0	712	0	11	45	1.545	1.545
5550	2020-4-6 8:00	DP		0	712	0	11	46	1.545	1.545
5551	2020-4-7 8:00	DP		0	712	0	11	47	1.545	1.545
5552	2020-4-8 8:00	DP		0	712	0	11	48	1.545	1.545
5553	2020-4-9 8:00	DP		0	712	0	11	49	1.545	1.545
5554	2020-4-10 8:00	DP		0	712	0	11	50	1.545	1.545
5555	2020-4-11 8:00	DP		0	712	0	11	51	1.545	1.545
5556	2020-4-12 8:00	DP		0	712	0	11	52	1.545	1.592
5557	2020-4-13 8:00	DP		0	712	1	12	53	1.685	1.639
5558	2020-4-14 8:00	DP		0	712	0	12	54	1.685	1.685
5559	2020-4-15 8:00	DP		0	712	0	12	55	1.685	1.685
5560	2020-4-16 8:00	DP		0	712	0	12	56	1.685	1.732
5561	2020-4-17 8:00	DP		0	712	1	13	57	1.826	1.779
5562	2020-4-18 8:00	DP		0	712	0	13	58	1.826	1.826
5563	2020-4-19 8:00	DP		0	712	0	13	59	1.826	1.826
5564	2020-4-20 8:00	DP		0	712	0	13	60	1.826	1.826
5565	2020-4-21 8:00	DP		0	712	0	13	61	1.826	1.826
5566	2020-4-22 8:00	DP		0	712	0	13	62	1.826	1.826
5567	2020-4-23 8:00	DP		0	712	0	13	63	1.826	1.826
5568	2020-4-24 8:00	DP		0	712	0	13	64	1.826	1.826
5569	2020-4-25 8:00	DP		0	712	0	13	65	1.826	1.826
5570	2020-4-26 8:00	DP		0	712	0	13	66	1.826	1.826
5571	2020-4-27 8:00	DP		0	712	0	13	67	1.826	1.826
5572	2020-4-28 8:00	DP		0	712	0	13	68	1.826	1.826
5573	2020-4-29 8:00	DP		0	712	0	13	69	1.826	1.826
5574	2020-4-30 8:00	DP		0	712	0	13	70	1.826	1.826
5575	2020-5-1 8:00	DP		0	712	0	13	71	1.826	1.826
5576	2020-5-2 8:00	DP		0	712	0	13	72	1.826	34.551
5577										
5578	2020-2-19 8:00	IR	EMRO	2	2	2	2	0	100.000	47.275
5579	2020-2-20 8:00	IR	EMRO	3	5	0	2	1	40.000	54.074
5580	2020-2-21 8:00	IR	EMRO	13	18	2	4	2	22.222	26.693

Table S1 Daily confirmed cases and daily deaths of COVID-19 in 214 nations from WHO

ID	Date and time	Country or region	Continent	Confirmed patients	Cumulative Confirmed patients	Daily dead patients	Daily cumulative dead patients	Lag days	Daily CFR	3DMA CFR
5581	2020-2-22 8:00	IR	EMRO	10	28	1	5	3	17.857	19.561
5582	2020-2-23 8:00	IR	EMRO	15	43	3	8	4	18.605	18.711
5583	2020-2-24 8:00	IR	EMRO	18	61	4	12	5	19.672	18.022
5584	2020-2-25 8:00	IR	EMRO	34	95	3	15	6	15.789	16.377
5585	2020-2-26 8:00	IR	EMRO	44	139	4	19	7	13.669	13.357
5586	2020-2-27 8:00	IR	EMRO	106	245	7	26	8	10.612	11.015
5587	2020-2-28 8:00	IR	EMRO	143	388	8	34	9	8.763	8.875
5588	2020-2-29 8:00	IR	EMRO	205	593	9	43	10	7.251	7.755
5589	2020-3-1 8:00	IR	EMRO	0	593	0	43	11	7.251	6.300
5590	2020-3-2 8:00	IR	EMRO	908	1501	23	66	12	4.397	4.982
5591	2020-3-3 8:00	IR	EMRO	835	2336	11	77	13	3.296	3.614
5592	2020-3-4 8:00	IR	EMRO	586	2922	15	92	14	3.149	3.164
5593	2020-3-5 8:00	IR	EMRO	591	3513	15	107	15	3.046	2.936
5594	2020-3-6 8:00	IR	EMRO	1234	4747	17	124	16	2.612	2.716
5595	2020-3-7 8:00	IR	EMRO	1076	5823	21	145	17	2.490	2.686
5596	2020-3-8 8:00	IR	EMRO	743	6566	49	194	18	2.955	2.918
5597	2020-3-9 8:00	IR	EMRO	595	7161	43	237	19	3.310	3.294
5598	2020-3-10 8:00	IR	EMRO	881	8042	54	291	20	3.619	3.620
5599	2020-3-11 8:00	IR	EMRO	958	9000	63	354	21	3.933	3.937
5600	2020-3-12 8:00	IR	EMRO	1075	10075	75	429	22	4.258	4.238
5601	2020-3-13 8:00	IR	EMRO	1289	11364	85	514	23	4.523	4.519
5602	2020-3-14 8:00	IR	EMRO	1365	12729	94	608	24	4.776	4.826
5603	2020-3-15 8:00	IR	EMRO	1254	13983	116	724	25	5.178	5.215
5604	2020-3-16 8:00	IR	EMRO	1008	14991	129	853	26	5.690	5.659
5605	2020-3-17 8:00	IR	EMRO	1178	16169	135	988	27	6.110	6.113
5606	2020-3-18 8:00	IR	EMRO	1192	17361	147	1135	28	6.538	6.541
5607	2020-3-19 8:00	IR	EMRO	1046	18407	149	1284	29	6.976	6.936
5608	2020-3-20 8:00	IR	EMRO	1237	19644	149	1433	30	7.295	7.273
5609	2020-3-21 8:00	IR	EMRO	966	20610	123	1556	31	7.550	7.544
5610	2020-3-22 8:00	IR	EMRO	1028	21638	129	1685	32	7.787	7.733
5611	2020-3-23 8:00	IR	EMRO	1411	23049	127	1812	33	7.862	7.815
5612	2020-3-24 8:00	IR	EMRO	1762	24811	122	1934	34	7.795	7.781
5613	2020-3-25 8:00	IR	EMRO	2206	27017	143	2077	35	7.688	7.693
5614	2020-3-26 8:00	IR	EMRO	2389	29406	157	2234	36	7.597	7.547
5615	2020-3-27 8:00	IR	EMRO	2926	32332	144	2378	37	7.355	7.354
5616	2020-3-28 8:00	IR	EMRO	3076	35408	139	2517	38	7.109	7.118

Table S1 Daily confirmed cases and daily deaths of COVID-19 in 214 nations from WHO

ID	Date and time	Country or region	Continent	Confirmed patients	Cumulative Confirmed patients	Daily dead patients	Daily cumulative dead patients	Lag days	Daily CFR	3DMA CFR
5617	2020-3-29 8:00	IR	EMRO	2901	38309	123	2640	39	6.891	6.881
5618	2020-3-30 8:00	IR	EMRO	3186	41495	117	2757	40	6.644	6.677
5619	2020-3-31 8:00	IR	EMRO	3111	44606	141	2898	41	6.497	6.507
5620	2020-4-1 8:00	IR	EMRO	2987	47593	138	3036	42	6.379	6.379
5621	2020-4-2 8:00	IR	EMRO	2875	50468	124	3160	43	6.261	6.278
5622	2020-4-3 8:00	IR	EMRO	2715	53183	134	3294	44	6.194	6.216
5623	2020-4-4 8:00	IR	EMRO	2560	55743	158	3452	45	6.193	6.191
5624	2020-4-5 8:00	IR	EMRO	2483	58226	151	3603	46	6.188	6.187
5625	2020-4-6 8:00	IR	EMRO	2274	60500	136	3739	47	6.180	6.185
5626	2020-4-7 8:00	IR	EMRO	2089	62589	133	3872	48	6.186	6.183
5627	2020-4-8 8:00	IR	EMRO	1997	64586	121	3993	49	6.182	6.192
5628	2020-4-9 8:00	IR	EMRO	1634	66220	117	4110	50	6.207	6.198
5629	2020-4-10 8:00	IR	EMRO	1972	68192	122	4232	51	6.206	6.211
5630	2020-4-11 8:00	IR	EMRO	1837	70029	125	4357	52	6.222	6.223
5631	2020-4-12 8:00	IR	EMRO	1657	71686	117	4474	53	6.241	6.239
5632	2020-4-13 8:00	IR	EMRO	1617	73303	111	4585	54	6.255	6.250
5633	2020-4-14 8:00	IR	EMRO	1574	74877	98	4683	55	6.254	6.254
5634	2020-4-15 8:00	IR	EMRO	1512	76389	94	4777	56	6.254	6.250
5635	2020-4-16 8:00	IR	EMRO	1606	77995	92	4869	57	6.243	6.244
5636	2020-4-17 8:00	IR	EMRO	1499	79494	89	4958	58	6.237	6.234
5637	2020-4-18 8:00	IR	EMRO	1374	80868	73	5031	59	6.221	6.228
5638	2020-4-19 8:00	IR	EMRO	1343	82211	87	5118	60	6.225	6.228
5639	2020-4-20 8:00	IR	EMRO	1294	83505	91	5209	61	6.238	6.237
5640	2020-4-21 8:00	IR	EMRO	1297	84802	88	5297	62	6.246	6.251
5641	2020-4-22 8:00	IR	EMRO	1194	85996	94	5391	63	6.269	6.271
5642	2020-4-23 8:00	IR	EMRO	1030	87026	90	5481	64	6.298	6.296
5643	2020-4-24 8:00	IR	EMRO	1168	88194	93	5574	65	6.320	6.314
5644	2020-4-25 8:00	IR	EMRO	1134	89328	76	5650	66	6.325	6.319
5645	2020-4-26 8:00	IR	EMRO	1153	90481	60	5710	67	6.311	6.328
5646	2020-4-27 8:00	IR	EMRO	991	91472	96	5806	68	6.347	6.335
5647	2020-4-28 8:00	IR	EMRO	1112	92584	71	5877	69	6.348	6.352
5648	2020-4-29 8:00	IR	EMRO	1073	93657	80	5957	70	6.360	6.359
5649	2020-4-30 8:00	IR	EMRO	983	94640	71	6028	71	6.369	6.366
5650	2020-5-1 8:00	IR	EMRO	1006	95646	63	6091	72	6.368	6.369
5651	2020-5-2 8:00	IR	EMRO	0	95646	0	6091	73	6.368	6.368
5652										

Table S1 Daily confirmed cases and daily deaths of COVID-19 in 214 nations from WHO

ID	Date and time	Country or region	Continent	Confirmed patients	Cumulative Confirmed patients	Daily dead patients	Daily cumulative dead patients	Lag days	Daily CFR	3DMA CFR
5653	2020-2-24 8:00	IQ	EMRO	1	1	0	0			
5654	2020-2-25 8:00	IQ	EMRO	4	5	0	0			
5655	2020-2-26 8:00	IQ	EMRO	0	5	0	0			
5656	2020-2-27 8:00	IQ	EMRO	1	6	0	0			
5657	2020-2-28 8:00	IQ	EMRO	2	8	0	0			
5658	2020-2-29 8:00	IQ	EMRO	5	13	0	0			
5659	2020-3-1 8:00	IQ	EMRO	6	19	0	0			
5660	2020-3-2 8:00	IQ	EMRO	7	26	0	0			
5661	2020-3-3 8:00	IQ	EMRO	5	31	0	0			
5662	2020-3-4 8:00	IQ	EMRO	5	36	2	2	0	5.556	5.556
5663	2020-3-5 8:00	IQ	EMRO	0	36	0	2	1	5.556	6.734
5664	2020-3-6 8:00	IQ	EMRO	8	44	2	4	2	9.091	7.351
5665	2020-3-7 8:00	IQ	EMRO	10	54	0	4	3	7.407	8.833
5666	2020-3-8 8:00	IQ	EMRO	6	60	2	6	4	10.000	9.136
5667	2020-3-9 8:00	IQ	EMRO	0	60	0	6	5	10.000	9.945
5668	2020-3-10 8:00	IQ	EMRO	1	61	0	6	6	9.836	9.945
5669	2020-3-11 8:00	IQ	EMRO	9	70	1	7	7	10.000	9.945
5670	2020-3-12 8:00	IQ	EMRO	0	70	0	7	8	10.000	10.000
5671	2020-3-13 8:00	IQ	EMRO	0	70	0	7	9	10.000	9.892
5672	2020-3-14 8:00	IQ	EMRO	23	93	2	9	10	9.677	8.978
5673	2020-3-15 8:00	IQ	EMRO	31	124	0	9	11	7.258	8.065
5674	2020-3-16 8:00	IQ	EMRO	0	124	0	9	12	7.258	7.220
5675	2020-3-17 8:00	IQ	EMRO	30	154	2	11	13	7.143	7.239
5676	2020-3-18 8:00	IQ	EMRO	10	164	1	12	14	7.317	7.080
5677	2020-3-19 8:00	IQ	EMRO	13	177	0	12	15	6.780	7.117
5678	2020-3-20 8:00	IQ	EMRO	16	193	2	14	16	7.254	7.326
5679	2020-3-21 8:00	IQ	EMRO	21	214	3	17	17	7.944	7.927
5680	2020-3-22 8:00	IQ	EMRO	19	233	3	20	18	8.584	8.391
5681	2020-3-23 8:00	IQ	EMRO	33	266	3	23	19	8.647	8.592
5682	2020-3-24 8:00	IQ	EMRO	50	316	4	27	20	8.544	8.524
5683	2020-3-25 8:00	IQ	EMRO	30	346	2	29	21	8.382	8.783
5684	2020-3-26 8:00	IQ	EMRO	36	382	7	36	22	9.424	8.846
5685	2020-3-27 8:00	IQ	EMRO	76	458	4	40	23	8.734	8.819
5686	2020-3-28 8:00	IQ	EMRO	48	506	2	42	24	8.300	8.237
5687	2020-3-29 8:00	IQ	EMRO	41	547	0	42	25	7.678	7.886
5688	2020-3-30 8:00	IQ	EMRO	0	547	0	42	26	7.678	7.553

Table S1 Daily confirmed cases and daily deaths of COVID-19 in 214 nations from WHO

ID	Date and time	Country or region	Continent	Confirmed patients	Cumulative Confirmed patients	Daily dead patients	Daily cumulative dead patients	Lag days	Daily CFR	3DMA CFR
5689	2020-3-31 8:00	IQ	EMRO	83	630	4	46	27	7.302	7.395
5690	2020-4-1 8:00	IQ	EMRO	64	694	4	50	28	7.205	7.167
5691	2020-4-2 8:00	IQ	EMRO	78	772	4	54	29	6.995	6.928
5692	2020-4-3 8:00	IQ	EMRO	48	820	0	54	30	6.585	6.653
5693	2020-4-4 8:00	IQ	EMRO	58	878	2	56	31	6.378	6.437
5694	2020-4-5 8:00	IQ	EMRO	83	961	5	61	32	6.348	6.311
5695	2020-4-6 8:00	IQ	EMRO	70	1031	3	64	33	6.208	6.254
5696	2020-4-7 8:00	IQ	EMRO	0	1031	0	64	34	6.208	6.069
5697	2020-4-8 8:00	IQ	EMRO	91	1122	1	65	35	5.793	5.914
5698	2020-4-9 8:00	IQ	EMRO	80	1202	4	69	36	5.740	5.669
5699	2020-4-10 8:00	IQ	EMRO	77	1279	1	70	37	5.473	5.559
5700	2020-4-11 8:00	IQ	EMRO	39	1318	2	72	38	5.463	5.519
5701	2020-4-12 8:00	IQ	EMRO	34	1352	4	76	39	5.621	5.582
5702	2020-4-13 8:00	IQ	EMRO	26	1378	2	78	40	5.660	5.618
5703	2020-4-14 8:00	IQ	EMRO	22	1400	0	78	41	5.571	5.605
5704	2020-4-15 8:00	IQ	EMRO	15	1415	1	79	42	5.583	5.578
5705	2020-4-16 8:00	IQ	EMRO	19	1434	1	80	43	5.579	5.580
5706	2020-4-17 8:00	IQ	EMRO	0	1434	0	80	44	5.579	5.541
5707	2020-4-18 8:00	IQ	EMRO	48	1482	1	81	45	5.466	5.488
5708	2020-4-19 8:00	IQ	EMRO	31	1513	1	82	46	5.420	5.365
5709	2020-4-20 8:00	IQ	EMRO	61	1574	0	82	47	5.210	5.270
5710	2020-4-21 8:00	IQ	EMRO	28	1602	1	83	48	5.181	5.191
5711	2020-4-22 8:00	IQ	EMRO	0	1602	0	83	49	5.181	5.104
5712	2020-4-23 8:00	IQ	EMRO	75	1677	0	83	50	4.949	5.027
5713	2020-4-24 8:00	IQ	EMRO	0	1677	0	83	51	4.949	4.978
5714	2020-4-25 8:00	IQ	EMRO	31	1708	3	86	52	5.035	4.954
5715	2020-4-26 8:00	IQ	EMRO	55	1763	0	86	53	4.878	4.898
5716	2020-4-27 8:00	IQ	EMRO	57	1820	1	87	54	4.780	4.808
5717	2020-4-28 8:00	IQ	EMRO	27	1847	1	88	55	4.764	4.738
5718	2020-4-29 8:00	IQ	EMRO	81	1928	2	90	56	4.668	4.675
5719	2020-4-30 8:00	IQ	EMRO	75	2003	2	92	57	4.593	4.574
5720	2020-5-1 8:00	IQ	EMRO	82	2085	1	93	58	4.460	4.473
5721	2020-5-2 8:00	IQ	EMRO	68	2153	1	94	59	4.366	4.413
5722										
5723	2020-3-1 8:00	IE	EURO	1	1	0	0			
5724	2020-3-2 8:00	IE	EURO	0	1	0	0			

Table S1 Daily confirmed cases and daily deaths of COVID-19 in 214 nations from WHO

ID	Date and time	Country or region	Continent	Confirmed patients	Cumulative Confirmed patients	Daily dead patients	Daily cumulative dead patients	Lag days	Daily CFR	3DMA CFR
5725	2020-3-3 8:00	IE	EURO	1	2	0	0			
5726	2020-3-4 8:00	IE	EURO	0	2	0	0			
5727	2020-3-5 8:00	IE	EURO	12	14	0	0			
5728	2020-3-6 8:00	IE	EURO	4	18	0	0			
5729	2020-3-7 8:00	IE	EURO	1	19	0	0			
5730	2020-3-8 8:00	IE	EURO	2	21	0	0			
5731	2020-3-9 8:00	IE	EURO	0	21	0	0			
5732	2020-3-10 8:00	IE	EURO	13	34	0	0			
5733	2020-3-11 8:00	IE	EURO	9	43	1	1	0	2.326	1.877
5734	2020-3-12 8:00	IE	EURO	27	70	0	1	1	1.429	1.618
5735	2020-3-13 8:00	IE	EURO	21	91	0	1	2	1.099	1.101
5736	2020-3-14 8:00	IE	EURO	38	129	0	1	3	0.775	1.019
5737	2020-3-15 8:00	IE	EURO	40	169	1	2	4	1.183	0.952
5738	2020-3-16 8:00	IE	EURO	54	223	0	2	5	0.897	0.922
5739	2020-3-17 8:00	IE	EURO	69	292	0	2	6	0.685	0.756
5740	2020-3-18 8:00	IE	EURO	0	292	0	2	7	0.685	0.636
5741	2020-3-19 8:00	IE	EURO	265	557	1	3	8	0.539	0.554
5742	2020-3-20 8:00	IE	EURO	126	683	0	3	9	0.439	0.453
5743	2020-3-21 8:00	IE	EURO	102	785	0	3	10	0.382	0.401
5744	2020-3-22 8:00	IE	EURO	0	785	0	3	11	0.382	0.402
5745	2020-3-23 8:00	IE	EURO	121	906	1	4	12	0.442	0.452
5746	2020-3-24 8:00	IE	EURO	219	1125	2	6	13	0.533	0.501
5747	2020-3-25 8:00	IE	EURO	204	1329	1	7	14	0.527	0.545
5748	2020-3-26 8:00	IE	EURO	235	1564	2	9	15	0.575	0.716
5749	2020-3-27 8:00	IE	EURO	255	1819	10	19	16	1.045	0.886
5750	2020-3-28 8:00	IE	EURO	302	2121	3	22	17	1.037	1.191
5751	2020-3-29 8:00	IE	EURO	294	2415	14	36	18	1.491	1.429
5752	2020-3-30 8:00	IE	EURO	200	2615	10	46	19	1.759	1.702
5753	2020-3-31 8:00	IE	EURO	295	2910	8	54	20	1.856	1.936
5754	2020-4-1 8:00	IE	EURO	325	3235	17	71	21	2.195	2.172
5755	2020-4-2 8:00	IE	EURO	212	3447	14	85	22	2.466	2.402
5756	2020-4-3 8:00	IE	EURO	402	3849	13	98	23	2.546	2.607
5757	2020-4-4 8:00	IE	EURO	424	4273	22	120	24	2.808	2.777
5758	2020-4-5 8:00	IE	EURO	331	4604	17	137	25	2.976	2.958
5759	2020-4-6 8:00	IE	EURO	507	5111	21	158	26	3.091	3.104
5760	2020-4-7 8:00	IE	EURO	253	5364	16	174	27	3.244	3.338

Table S1 Daily confirmed cases and daily deaths of COVID-19 in 214 nations from WHO

ID	Date and time	Country or region	Continent	Confirmed patients	Cumulative Confirmed patients	Daily dead patients	Daily cumulative dead patients	Lag days	Daily CFR	3DMA CFR
5761	2020-4-8 8:00	IE	EURO	345	5709	36	210	28	3.678	3.566
5762	2020-4-9 8:00	IE	EURO	515	6224	25	235	29	3.776	3.671
5763	2020-4-10 8:00	IE	EURO	1169	7393	28	263	30	3.557	3.627
5764	2020-4-11 8:00	IE	EURO	696	8089	24	287	31	3.548	3.563
5765	2020-4-12 8:00	IE	EURO	839	8928	33	320	32	3.584	3.531
5766	2020-4-13 8:00	IE	EURO	727	9655	14	334	33	3.459	3.491
5767	2020-4-14 8:00	IE	EURO	992	10647	31	365	34	3.428	3.475
5768	2020-4-15 8:00	IE	EURO	832	11479	41	406	35	3.537	3.501
5769	2020-4-16 8:00	IE	EURO	1068	12547	38	444	36	3.539	3.579
5770	2020-4-17 8:00	IE	EURO	724	13271	42	486	37	3.662	3.664
5771	2020-4-18 8:00	IE	EURO	709	13980	44	530	38	3.791	3.774
5772	2020-4-19 8:00	IE	EURO	778	14758	41	571	39	3.869	3.887
5773	2020-4-20 8:00	IE	EURO	493	15251	39	610	40	4.000	4.086
5774	2020-4-21 8:00	IE	EURO	401	15652	77	687	41	4.389	4.313
5775	2020-4-22 8:00	IE	EURO	388	16040	43	730	42	4.551	4.518
5776	2020-4-23 8:00	IE	EURO	631	16671	39	769	43	4.613	4.558
5777	2020-4-24 8:00	IE	EURO	936	17607	25	794	44	4.510	4.560
5778	2020-4-25 8:00	IE	EURO	577	18184	35	829	45	4.559	4.932
5779	2020-4-26 8:00	IE	EURO	377	18561	234	1063	46	5.727	5.310
5780	2020-4-27 8:00	IE	EURO	701	19262	24	1087	47	5.643	5.660
5781	2020-4-28 8:00	IE	EURO	386	19648	15	1102	48	5.609	5.694
5782	2020-4-29 8:00	IE	EURO	229	19877	57	1159	49	5.831	5.772
5783	2020-4-30 8:00	IE	EURO	376	20253	31	1190	50	5.876	5.895
5784	2020-5-1 8:00	IE	EURO	359	20612	42	1232	51	5.977	5.975
5785	2020-5-2 8:00	IE	EURO	221	20833	33	1265	52	6.072	6.025
5786										
5787	2020-3-20 8:00	IM	EURO	1	1	0	0			
5788	2020-3-21 8:00	IM	EURO	1	2	0	0			
5789	2020-3-22 8:00	IM	EURO	0	2	0	0			
5790	2020-3-23 8:00	IM	EURO	0	2	0	0			
5791	2020-3-24 8:00	IM	EURO	11	13	0	0			
5792	2020-3-25 8:00	IM	EURO	10	23	0	0			
5793	2020-3-26 8:00	IM	EURO	0	23	0	0			
5794	2020-3-27 8:00	IM	EURO	3	26	0	0			
5795	2020-3-28 8:00	IM	EURO	3	29	0	0			
5796	2020-3-29 8:00	IM	EURO	3	32	0	0			

Table S1 Daily confirmed cases and daily deaths of COVID-19 in 214 nations from WHO

ID	Date and time	Country or region	Continent	Confirmed patients	Cumulative Confirmed patients	Daily dead patients	Daily cumulative dead patients	Lag days	Daily CFR	3DMA CFR
5797	2020-3-30 8:00	IM	EURO	10	42	0	0			
5798	2020-3-31 8:00	IM	EURO	0	42	0	0			
5799	2020-4-1 8:00	IM	EURO	10	52	0	0			
5800	2020-4-2 8:00	IM	EURO	13	65	0	0			
5801	2020-4-3 8:00	IM	EURO	6	71	1	1	0	1.408	1.143
5802	2020-4-4 8:00	IM	EURO	43	114	0	1	1	0.877	1.026
5803	2020-4-5 8:00	IM	EURO	12	126	0	1	2	0.794	0.819
5804	2020-4-6 8:00	IM	EURO	1	127	0	1	3	0.787	0.789
5805	2020-4-7 8:00	IM	EURO	0	127	0	1	4	0.787	0.747
5806	2020-4-8 8:00	IM	EURO	23	150	0	1	5	0.667	0.707
5807	2020-4-9 8:00	IM	EURO	0	150	0	1	6	0.667	0.646
5808	2020-4-10 8:00	IM	EURO	15	165	0	1	7	0.606	0.600
5809	2020-4-11 8:00	IM	EURO	25	190	0	1	8	0.526	0.541
5810	2020-4-12 8:00	IM	EURO	14	204	0	1	9	0.490	0.486
5811	2020-4-13 8:00	IM	EURO	22	226	0	1	10	0.442	0.603
5812	2020-4-14 8:00	IM	EURO	2	228	1	2	11	0.877	0.715
5813	2020-4-15 8:00	IM	EURO	14	242	0	2	12	0.826	0.830
5814	2020-4-16 8:00	IM	EURO	12	254	0	2	13	0.787	1.055
5815	2020-4-17 8:00	IM	EURO	4	258	2	4	14	1.550	1.241
5816	2020-4-18 8:00	IM	EURO	31	289	0	4	15	1.384	1.436
5817	2020-4-19 8:00	IM	EURO	2	291	0	4	16	1.375	1.368
5818	2020-4-20 8:00	IM	EURO	6	297	0	4	17	1.347	1.356
5819	2020-4-21 8:00	IM	EURO	0	297	0	4	18	1.347	1.984
5820	2020-4-22 8:00	IM	EURO	10	307	6	10	19	3.257	2.620
5821	2020-4-23 8:00	IM	EURO	0	307	0	10	20	3.257	3.909
5822	2020-4-24 8:00	IM	EURO	0	307	6	16	21	5.212	4.663
5823	2020-4-25 8:00	IM	EURO	1	308	1	17	22	5.519	5.525
5824	2020-4-26 8:00	IM	EURO	0	308	1	18	23	5.844	5.736
5825	2020-4-27 8:00	IM	EURO	0	308	0	18	24	5.844	5.844
5826	2020-4-28 8:00	IM	EURO	0	308	0	18	25	5.844	6.061
5827	2020-4-29 8:00	IM	EURO	0	308	2	20	26	6.494	6.378
5828	2020-4-30 8:00	IM	EURO	1	309	1	21	27	6.796	6.788
5829	2020-5-1 8:00	IM	EURO	2	311	1	22	28	7.074	6.981
5830	2020-5-2 8:00	IM	EURO	0	311	0	22	29	7.074	7.074
5831										
5832	2020-2-21 8:00	IL	EURO	1	1	0	0			

Table S1 Daily confirmed cases and daily deaths of COVID-19 in 214 nations from WHO

ID	Date and time	Country or region	Continent	Confirmed patients	Cumulative Confirmed patients	Daily dead patients	Daily cumulative dead patients	Lag days	Daily CFR	3DMA CFR
5833	2020-2-22 8:00	IL	EURO	0	1	0	0			
5834	2020-2-23 8:00	IL	EURO	0	1	0	0			
5835	2020-2-24 8:00	IL	EURO	1	2	0	0			
5836	2020-2-25 8:00	IL	EURO	0	2	0	0			
5837	2020-2-26 8:00	IL	EURO	0	2	0	0			
5838	2020-2-27 8:00	IL	EURO	1	3	0	0			
5839	2020-2-28 8:00	IL	EURO	2	5	0	0			
5840	2020-2-29 8:00	IL	EURO	2	7	0	0			
5841	2020-3-1 8:00	IL	EURO	0	7	0	0			
5842	2020-3-2 8:00	IL	EURO	3	10	0	0			
5843	2020-3-3 8:00	IL	EURO	2	12	0	0			
5844	2020-3-4 8:00	IL	EURO	3	15	0	0			
5845	2020-3-5 8:00	IL	EURO	0	15	0	0			
5846	2020-3-6 8:00	IL	EURO	4	19	0	0			
5847	2020-3-7 8:00	IL	EURO	2	21	0	0			
5848	2020-3-8 8:00	IL	EURO	18	39	0	0			
5849	2020-3-9 8:00	IL	EURO	0	39	0	0			
5850	2020-3-10 8:00	IL	EURO	19	58	0	0			
5851	2020-3-11 8:00	IL	EURO	17	75	0	0			
5852	2020-3-12 8:00	IL	EURO	24	99	0	0			
5853	2020-3-13 8:00	IL	EURO	27	126	0	0			
5854	2020-3-14 8:00	IL	EURO	52	178	0	0			
5855	2020-3-15 8:00	IL	EURO	22	200	0	0			
5856	2020-3-16 8:00	IL	EURO	50	250	0	0			
5857	2020-3-17 8:00	IL	EURO	54	304	0	0			
5858	2020-3-18 8:00	IL	EURO	123	427	0	0			
5859	2020-3-19 8:00	IL	EURO	102	529	0	0			
5860	2020-3-20 8:00	IL	EURO	183	712	1	1	0	0.140	0.127
5861	2020-3-21 8:00	IL	EURO	171	883	0	1	1	0.113	0.122
5862	2020-3-22 8:00	IL	EURO	0	883	0	1	2	0.113	0.107
5863	2020-3-23 8:00	IL	EURO	188	1071	0	1	3	0.093	0.096
5864	2020-3-24 8:00	IL	EURO	167	1238	0	1	4	0.081	0.135
5865	2020-3-25 8:00	IL	EURO	932	2170	4	5	5	0.230	0.174
5866	2020-3-26 8:00	IL	EURO	199	2369	0	5	6	0.211	0.257
5867	2020-3-27 8:00	IL	EURO	666	3035	5	10	7	0.329	0.296
5868	2020-3-28 8:00	IL	EURO	425	3460	2	12	8	0.347	0.355

Table S1 Daily confirmed cases and daily deaths of COVID-19 in 214 nations from WHO

ID	Date and time	Country or region	Continent	Confirmed patients	Cumulative Confirmed patients	Daily dead patients	Daily cumulative dead patients	Lag days	Daily CFR	3DMA CFR
5869	2020-3-29 8:00	IL	EURO	405	3865	3	15	9	0.388	0.363
5870	2020-3-30 8:00	IL	EURO	382	4247	0	15	10	0.353	0.364
5871	2020-3-31 8:00	IL	EURO	584	4831	2	17	11	0.352	0.372
5872	2020-4-1 8:00	IL	EURO	298	5129	4	21	12	0.409	0.379
5873	2020-4-2 8:00	IL	EURO	462	5591	0	21	13	0.376	0.417
5874	2020-4-3 8:00	IL	EURO	620	6211	8	29	14	0.467	0.452
5875	2020-4-4 8:00	IL	EURO	819	7030	7	36	15	0.512	0.511
5876	2020-4-5 8:00	IL	EURO	559	7589	6	42	16	0.553	0.546
5877	2020-4-6 8:00	IL	EURO	429	8018	4	46	17	0.574	0.577
5878	2020-4-7 8:00	IL	EURO	593	8611	6	52	18	0.604	0.644
5879	2020-4-8 8:00	IL	EURO	793	9404	19	71	19	0.755	0.705
5880	2020-4-9 8:00	IL	EURO	0	9404	0	71	20	0.755	0.773
5881	2020-4-10 8:00	IL	EURO	351	9755	8	79	21	0.810	0.825
5882	2020-4-11 8:00	IL	EURO	340	10095	13	92	22	0.911	0.878
5883	2020-4-12 8:00	IL	EURO	430	10525	4	96	23	0.912	0.923
5884	2020-4-13 8:00	IL	EURO	353	10878	7	103	24	0.947	0.946
5885	2020-4-14 8:00	IL	EURO	357	11235	7	110	25	0.979	0.971
5886	2020-4-15 8:00	IL	EURO	633	11868	7	117	26	0.986	0.999
5887	2020-4-16 8:00	IL	EURO	332	12200	9	126	27	1.033	1.044
5888	2020-4-17 8:00	IL	EURO	391	12591	14	140	28	1.112	1.099
5889	2020-4-18 8:00	IL	EURO	264	12855	8	148	29	1.151	1.156
5890	2020-4-19 8:00	IL	EURO	252	13107	10	158	30	1.205	1.212
5891	2020-4-20 8:00	IL	EURO	255	13362	13	171	31	1.280	1.263
5892	2020-4-21 8:00	IL	EURO	521	13883	10	181	32	1.304	1.301
5893	2020-4-22 8:00	IL	EURO	59	13942	3	184	33	1.320	1.309
5894	2020-4-23 8:00	IL	EURO	556	14498	5	189	34	1.304	1.307
5895	2020-4-24 8:00	IL	EURO	305	14803	3	192	35	1.297	1.297
5896	2020-4-25 8:00	IL	EURO	225	15028	2	194	36	1.291	1.293
5897	2020-4-26 8:00	IL	EURO	370	15398	5	199	37	1.292	1.292
5898	2020-4-27 8:00	IL	EURO	0	15398	0	199	38	1.292	1.297
5899	2020-4-28 8:00	IL	EURO	68	15466	3	202	39	1.306	1.314
5900	2020-4-29 8:00	IL	EURO	316	15782	10	212	40	1.343	1.331
5901	2020-4-30 8:00	IL	EURO	0	15782	0	212	41	1.343	1.360
5902	2020-5-1 8:00	IL	EURO	222	16004	11	223	42	1.393	1.381
5903	2020-5-2 8:00	IL	EURO	148	16152	4	227	43	1.405	1.399
5904										

Table S1 Daily confirmed cases and daily deaths of COVID-19 in 214 nations from WHO

ID	Date and time	Country or region	Continent	Confirmed patients	Cumulative Confirmed patients	Daily dead patients	Daily cumulative dead patients	Lag days	Daily CFR	3DMA CFR
5905	2020-1-29 8:00	IT	EURO	2	2	0	0			
5906	2020-1-30 8:00	IT	EURO	0	2	0	0			
5907	2020-1-31 8:00	IT	EURO	0	2	0	0			
5908	2020-2-1 8:00	IT	EURO	0	2	0	0			
5909	2020-2-2 8:00	IT	EURO	0	2	0	0			
5910	2020-2-3 8:00	IT	EURO	0	2	0	0			
5911	2020-2-4 8:00	IT	EURO	0	2	0	0			
5912	2020-2-5 8:00	IT	EURO	0	2	0	0			
5913	2020-2-6 8:00	IT	EURO	0	2	0	0			
5914	2020-2-7 8:00	IT	EURO	1	3	0	0			
5915	2020-2-8 8:00	IT	EURO	0	3	0	0			
5916	2020-2-9 8:00	IT	EURO	0	3	0	0			
5917	2020-2-10 8:00	IT	EURO	0	3	0	0			
5918	2020-2-11 8:00	IT	EURO	0	3	0	0			
5919	2020-2-12 8:00	IT	EURO	0	3	0	0			
5920	2020-2-13 8:00	IT	EURO	0	3	0	0			
5921	2020-2-14 8:00	IT	EURO	0	3	0	0			
5922	2020-2-15 8:00	IT	EURO	0	3	0	0			
5923	2020-2-16 8:00	IT	EURO	0	3	0	0			
5924	2020-2-17 8:00	IT	EURO	0	3	0	0			
5925	2020-2-18 8:00	IT	EURO	0	3	0	0			
5926	2020-2-19 8:00	IT	EURO	0	3	0	0			
5927	2020-2-20 8:00	IT	EURO	0	3	0	0			
5928	2020-2-21 8:00	IT	EURO	1	4	0	0			
5929	2020-2-22 8:00	IT	EURO	7	11	2	2	0	18.182	9.897
5930	2020-2-23 8:00	IT	EURO	113	124	0	2	1	1.613	7.617
5931	2020-2-24 8:00	IT	EURO	105	229	5	7	2	3.057	2.799
5932	2020-2-25 8:00	IT	EURO	93	322	5	12	3	3.727	3.261
5933	2020-2-26 8:00	IT	EURO	78	400	0	12	4	3.000	3.114
5934	2020-2-27 8:00	IT	EURO	250	650	5	17	5	2.615	2.660
5935	2020-2-28 8:00	IT	EURO	238	888	4	21	6	2.365	2.517
5936	2020-2-29 8:00	IT	EURO	240	1128	8	29	7	2.571	2.336
5937	2020-3-1 8:00	IT	EURO	561	1689	6	35	8	2.072	2.399
5938	2020-3-2 8:00	IT	EURO	347	2036	17	52	9	2.554	2.608
5939	2020-3-3 8:00	IT	EURO	466	2502	28	80	10	3.197	3.072
5940	2020-3-4 8:00	IT	EURO	587	3089	27	107	11	3.464	3.499

Table S1 Daily confirmed cases and daily deaths of COVID-19 in 214 nations from WHO

ID	Date and time	Country or region	Continent	Confirmed patients	Cumulative Confirmed patients	Daily dead patients	Daily cumulative dead patients	Lag days	Daily CFR	3DMA CFR
5941	2020-3-5 8:00	IT	EURO	769	3858	41	148	12	3.836	3.850
5942	2020-3-6 8:00	IT	EURO	778	4636	49	197	13	4.249	4.021
5943	2020-3-7 8:00	IT	EURO	1247	5883	37	234	14	3.978	4.397
5944	2020-3-8 8:00	IT	EURO	1492	7375	132	366	15	4.963	4.663
5945	2020-3-9 8:00	IT	EURO	1797	9172	97	463	16	5.048	5.409
5946	2020-3-10 8:00	IT	EURO	977	10149	168	631	17	6.217	5.967
5947	2020-3-11 8:00	IT	EURO	2313	12462	196	827	18	6.636	6.525
5948	2020-3-12 8:00	IT	EURO	2651	15113	189	1016	19	6.723	6.846
5949	2020-3-13 8:00	IT	EURO	2547	17660	252	1268	20	7.180	6.905
5950	2020-3-14 8:00	IT	EURO	3497	21157	173	1441	21	6.811	7.100
5951	2020-3-15 8:00	IT	EURO	3590	24747	368	1809	22	7.310	7.278
5952	2020-3-16 8:00	IT	EURO	3233	27980	349	2158	23	7.713	7.658
5953	2020-3-17 8:00	IT	EURO	3526	31506	347	2505	24	7.951	8.001
5954	2020-3-18 8:00	IT	EURO	4207	35713	473	2978	25	8.339	8.197
5955	2020-3-19 8:00	IT	EURO	5322	41035	429	3407	26	8.303	8.405
5956	2020-3-20 8:00	IT	EURO	5986	47021	625	4032	27	8.575	8.629
5957	2020-3-21 8:00	IT	EURO	6557	53578	795	4827	28	9.009	8.864
5958	2020-3-22 8:00	IT	EURO	0	53578	0	4827	29	9.009	9.093
5959	2020-3-23 8:00	IT	EURO	5560	59138	649	5476	30	9.260	9.258
5960	2020-3-24 8:00	IT	EURO	4789	63927	601	6077	31	9.506	9.542
5961	2020-3-25 8:00	IT	EURO	5249	69176	743	6820	32	9.859	9.818
5962	2020-3-26 8:00	IT	EURO	5210	74386	685	7505	33	10.089	10.029
5963	2020-3-27 8:00	IT	EURO	6153	80539	660	8165	34	10.138	10.263
5964	2020-3-28 8:00	IT	EURO	5959	86498	971	9136	35	10.562	10.513
5965	2020-3-29 8:00	IT	EURO	5974	92472	887	10023	36	10.839	10.812
5966	2020-3-30 8:00	IT	EURO	5217	97689	758	10781	37	11.036	11.089
5967	2020-3-31 8:00	IT	EURO	4050	101739	810	11591	38	11.393	11.393
5968	2020-4-1 8:00	IT	EURO	4053	105792	839	12430	39	11.749	11.680
5969	2020-4-2 8:00	IT	EURO	4782	110574	727	13157	40	11.899	11.908
5970	2020-4-3 8:00	IT	EURO	4668	115242	760	13917	41	12.076	12.076
5971	2020-4-4 8:00	IT	EURO	4585	119827	764	14681	42	12.252	12.218
5972	2020-4-5 8:00	IT	EURO	4805	124632	681	15362	43	12.326	12.300
5973	2020-4-6 8:00	IT	EURO	4316	128948	527	15889	44	12.322	12.372
5974	2020-4-7 8:00	IT	EURO	3599	132547	636	16525	45	12.467	12.474
5975	2020-4-8 8:00	IT	EURO	3039	135586	604	17129	46	12.633	12.591
5976	2020-4-9 8:00	IT	EURO	3836	139422	540	17669	47	12.673	12.678

Table S1 Daily confirmed cases and daily deaths of COVID-19 in 214 nations from WHO

ID	Date and time	Country or region	Continent	Confirmed patients	Cumulative Confirmed patients	Daily dead patients	Daily cumulative dead patients	Lag days	Daily CFR	3DMA CFR
5977	2020-4-10 8:00	IT	EURO	4204	143626	612	18281	48	12.728	12.725
5978	2020-4-11 8:00	IT	EURO	3951	147577	570	18851	49	12.774	12.763
5979	2020-4-12 8:00	IT	EURO	4694	152271	619	19470	50	12.786	12.763
5980	2020-4-13 8:00	IT	EURO	4092	156363	431	19901	51	12.727	12.781
5981	2020-4-14 8:00	IT	EURO	3153	159516	564	20465	52	12.829	12.841
5982	2020-4-15 8:00	IT	EURO	2972	162488	604	21069	53	12.966	12.968
5983	2020-4-16 8:00	IT	EURO	2667	165155	578	21647	54	13.107	13.066
5984	2020-4-17 8:00	IT	EURO	3786	168941	525	22172	55	13.124	13.141
5985	2020-4-18 8:00	IT	EURO	3493	172434	575	22747	56	13.192	13.173
5986	2020-4-19 8:00	IT	EURO	3491	175925	480	23227	57	13.203	13.205
5987	2020-4-20 8:00	IT	EURO	3047	178972	433	23660	58	13.220	13.243
5988	2020-4-21 8:00	IT	EURO	2256	181228	454	24114	59	13.306	13.308
5989	2020-4-22 8:00	IT	EURO	2729	183957	534	24648	60	13.399	13.365
5990	2020-4-23 8:00	IT	EURO	3370	187327	437	25085	61	13.391	13.413
5991	2020-4-24 8:00	IT	EURO	2646	189973	464	25549	62	13.449	13.432
5992	2020-4-25 8:00	IT	EURO	3021	192994	420	25969	63	13.456	13.470
5993	2020-4-26 8:00	IT	EURO	2357	195351	415	26384	64	13.506	13.480
5994	2020-4-27 8:00	IT	EURO	2324	197675	260	26644	65	13.479	13.504
5995	2020-4-28 8:00	IT	EURO	1739	199414	333	26977	66	13.528	13.528
5996	2020-4-29 8:00	IT	EURO	2091	201505	382	27359	67	13.577	13.567
5997	2020-4-30 8:00	IT	EURO	2086	203591	323	27682	68	13.597	13.595
5998	2020-5-1 8:00	IT	EURO	1872	205463	285	27967	69	13.612	13.607
5999	2020-5-2 8:00	IT	EURO	1965	207428	269	28236	70	13.612	13.612
6000										
6001	2020-3-10 8:00	JM	AMRO	1	1	0	0			
6002	2020-3-11 8:00	JM	AMRO	0	1	0	0			
6003	2020-3-12 8:00	JM	AMRO	0	1	0	0			
6004	2020-3-13 8:00	JM	AMRO	1	2	0	0			
6005	2020-3-14 8:00	JM	AMRO	6	8	0	0			
6006	2020-3-15 8:00	JM	AMRO	0	8	0	0			
6007	2020-3-16 8:00	JM	AMRO	2	10	0	0			
6008	2020-3-17 8:00	JM	AMRO	2	12	0	0			
6009	2020-3-18 8:00	JM	AMRO	1	13	0	0			
6010	2020-3-19 8:00	JM	AMRO	2	15	1	1	0	6.667	6.458
6011	2020-3-20 8:00	JM	AMRO	1	16	0	1	1	6.250	6.389
6012	2020-3-21 8:00	JM	AMRO	0	16	0	1	2	6.250	6.250

Table S1 Daily confirmed cases and daily deaths of COVID-19 in 214 nations from WHO

ID	Date and time	Country or region	Continent	Confirmed patients	Cumulative Confirmed patients	Daily dead patients	Daily cumulative dead patients	Lag days	Daily CFR	3DMA CFR
6013	2020-3-22 8:00	JM	AMRO	0	16	0	1	3	6.250	5.921
6014	2020-3-23 8:00	JM	AMRO	3	19	0	1	4	5.263	5.425
6015	2020-3-24 8:00	JM	AMRO	2	21	0	1	5	4.762	4.929
6016	2020-3-25 8:00	JM	AMRO	0	21	0	1	6	4.762	4.457
6017	2020-3-26 8:00	JM	AMRO	5	26	0	1	7	3.846	4.151
6018	2020-3-27 8:00	JM	AMRO	0	26	0	1	8	3.846	3.846
6019	2020-3-28 8:00	JM	AMRO	0	26	0	1	9	3.846	3.675
6020	2020-3-29 8:00	JM	AMRO	4	30	0	1	10	3.333	3.435
6021	2020-3-30 8:00	JM	AMRO	2	32	0	1	11	3.125	3.079
6022	2020-3-31 8:00	JM	AMRO	4	36	0	1	12	2.778	2.894
6023	2020-4-1 8:00	JM	AMRO	0	36	0	1	13	2.778	2.729
6024	2020-4-2 8:00	JM	AMRO	2	38	0	1	14	2.632	4.076
6025	2020-4-3 8:00	JM	AMRO	6	44	2	3	15	6.818	5.278
6026	2020-4-4 8:00	JM	AMRO	3	47	0	3	16	6.383	6.287
6027	2020-4-5 8:00	JM	AMRO	6	53	0	3	17	5.660	5.833
6028	2020-4-6 8:00	JM	AMRO	2	55	0	3	18	5.455	5.429
6029	2020-4-7 8:00	JM	AMRO	3	58	0	3	19	5.172	5.237
6030	2020-4-8 8:00	JM	AMRO	1	59	0	3	20	5.085	5.006
6031	2020-4-9 8:00	JM	AMRO	4	63	0	3	21	4.762	5.399
6032	2020-4-10 8:00	JM	AMRO	0	63	1	4	22	6.349	5.820
6033	2020-4-11 8:00	JM	AMRO	0	63	0	4	23	6.349	6.284
6034	2020-4-12 8:00	JM	AMRO	2	65	0	4	24	6.154	6.100
6035	2020-4-13 8:00	JM	AMRO	4	69	0	4	25	5.797	5.836
6036	2020-4-14 8:00	JM	AMRO	3	72	0	4	26	5.556	5.611
6037	2020-4-15 8:00	JM	AMRO	1	73	0	4	27	5.479	5.266
6038	2020-4-16 8:00	JM	AMRO	32	105	1	5	28	4.762	4.747
6039	2020-4-17 8:00	JM	AMRO	20	125	0	5	29	4.000	4.086
6040	2020-4-18 8:00	JM	AMRO	18	143	0	5	30	3.497	3.521
6041	2020-4-19 8:00	JM	AMRO	20	163	0	5	31	3.067	3.151
6042	2020-4-20 8:00	JM	AMRO	10	173	0	5	32	2.890	2.836
6043	2020-4-21 8:00	JM	AMRO	23	196	0	5	33	2.551	2.711
6044	2020-4-22 8:00	JM	AMRO	27	223	1	6	34	2.691	2.606
6045	2020-4-23 8:00	JM	AMRO	10	233	0	6	35	2.575	2.578
6046	2020-4-24 8:00	JM	AMRO	10	243	0	6	36	2.469	2.589
6047	2020-4-25 8:00	JM	AMRO	14	257	1	7	37	2.724	2.541
6048	2020-4-26 8:00	JM	AMRO	31	288	0	7	38	2.431	2.483

Table S1 Daily confirmed cases and daily deaths of COVID-19 in 214 nations from WHO

ID	Date and time	Country or region	Continent	Confirmed patients	Cumulative Confirmed patients	Daily dead patients	Daily cumulative dead patients	Lag days	Daily CFR	3DMA CFR
6049	2020-4-27 8:00	JM	AMRO	17	305	0	7	39	2.295	2.340
6050	2020-4-28 8:00	JM	AMRO	0	305	0	7	40	2.295	2.171
6051	2020-4-29 8:00	JM	AMRO	59	364	0	7	41	1.923	2.018
6052	2020-4-30 8:00	JM	AMRO	17	381	0	7	42	1.837	1.843
6053	2020-5-1 8:00	JM	AMRO	15	396	0	7	43	1.768	1.834
6054	2020-5-2 8:00	JM	AMRO	26	422	1	8	44	1.896	1.832
6055										
6056	2020-1-14 8:00	JP	WPRO	1	1	0	0			
6057	2020-1-15 8:00	JP	WPRO	0	1	0	0			
6058	2020-1-16 8:00	JP	WPRO	0	1	0	0			
6059	2020-1-17 8:00	JP	WPRO	0	1	0	0			
6060	2020-1-18 8:00	JP	WPRO	0	1	0	0			
6061	2020-1-19 8:00	JP	WPRO	0	1	0	0			
6062	2020-1-20 8:00	JP	WPRO	0	1	0	0			
6063	2020-1-21 8:00	JP	WPRO	0	1	0	0			
6064	2020-1-22 8:00	JP	WPRO	0	1	0	0			
6065	2020-1-23 8:00	JP	WPRO	0	1	0	0			
6066	2020-1-24 8:00	JP	WPRO	1	2	0	0			
6067	2020-1-25 8:00	JP	WPRO	1	3	0	0			
6068	2020-1-26 8:00	JP	WPRO	1	4	0	0			
6069	2020-1-27 8:00	JP	WPRO	0	4	0	0			
6070	2020-1-28 8:00	JP	WPRO	3	7	0	0			
6071	2020-1-29 8:00	JP	WPRO	4	11	0	0			
6072	2020-1-30 8:00	JP	WPRO	3	14	0	0			
6073	2020-1-31 8:00	JP	WPRO	3	17	0	0			
6074	2020-2-1 8:00	JP	WPRO	3	20	0	0			
6075	2020-2-2 8:00	JP	WPRO	0	20	0	0			
6076	2020-2-3 8:00	JP	WPRO	0	20	0	0			
6077	2020-2-4 8:00	JP	WPRO	3	23	0	0			
6078	2020-2-5 8:00	JP	WPRO	2	25	0	0			
6079	2020-2-6 8:00	JP	WPRO	0	25	0	0			
6080	2020-2-7 8:00	JP	WPRO	0	25	0	0			
6081	2020-2-8 8:00	JP	WPRO	0	25	0	0			
6082	2020-2-9 8:00	JP	WPRO	1	26	0	0			
6083	2020-2-10 8:00	JP	WPRO	0	26	0	0			
6084	2020-2-11 8:00	JP	WPRO	2	28	0	0			

Table S1 Daily confirmed cases and daily deaths of COVID-19 in 214 nations from WHO

ID	Date and time	Country or region	Continent	Confirmed patients	Cumulative Confirmed patients	Daily dead patients	Daily cumulative dead patients	Lag days	Daily CFR	3DMA CFR
6085	2020-2-12 8:00	JP	WPRO	1	29	0	0			
6086	2020-2-13 8:00	JP	WPRO	4	33	1	1	0	3.030	2.735
6087	2020-2-14 8:00	JP	WPRO	8	41	0	1	1	2.439	2.452
6088	2020-2-15 8:00	JP	WPRO	12	53	0	1	2	1.887	2.007
6089	2020-2-16 8:00	JP	WPRO	6	59	0	1	3	1.695	1.707
6090	2020-2-17 8:00	JP	WPRO	6	65	0	1	4	1.538	1.541
6091	2020-2-18 8:00	JP	WPRO	7	72	0	1	5	1.389	1.432
6092	2020-2-19 8:00	JP	WPRO	1	73	0	1	6	1.370	1.278
6093	2020-2-20 8:00	JP	WPRO	20	93	0	1	7	1.075	1.133
6094	2020-2-21 8:00	JP	WPRO	12	105	0	1	8	0.952	0.928
6095	2020-2-22 8:00	JP	WPRO	27	132	0	1	9	0.758	0.801
6096	2020-2-23 8:00	JP	WPRO	12	144	0	1	10	0.694	0.698
6097	2020-2-24 8:00	JP	WPRO	12	156	0	1	11	0.641	0.657
6098	2020-2-25 8:00	JP	WPRO	1	157	0	1	12	0.637	0.964
6099	2020-2-26 8:00	JP	WPRO	29	186	2	3	13	1.613	1.463
6100	2020-2-27 8:00	JP	WPRO	1	187	1	4	14	2.139	2.044
6101	2020-2-28 8:00	JP	WPRO	23	210	1	5	15	2.381	2.204
6102	2020-2-29 8:00	JP	WPRO	29	239	0	5	16	2.092	2.328
6103	2020-3-1 8:00	JP	WPRO	0	239	1	6	17	2.510	2.280
6104	2020-3-2 8:00	JP	WPRO	29	268	0	6	18	2.239	2.287
6105	2020-3-3 8:00	JP	WPRO	16	284	0	6	19	2.113	2.147
6106	2020-3-4 8:00	JP	WPRO	3	287	0	6	20	2.091	1.974
6107	2020-3-5 8:00	JP	WPRO	62	349	0	6	21	1.719	1.843
6108	2020-3-6 8:00	JP	WPRO	0	349	0	6	22	1.719	1.638
6109	2020-3-7 8:00	JP	WPRO	58	407	0	6	23	1.474	1.504
6110	2020-3-8 8:00	JP	WPRO	48	455	0	6	24	1.319	1.515
6111	2020-3-9 8:00	JP	WPRO	59	514	3	9	25	1.751	1.607
6112	2020-3-10 8:00	JP	WPRO	0	514	0	9	26	1.751	1.872
6113	2020-3-11 8:00	JP	WPRO	54	568	3	12	27	2.113	2.094
6114	2020-3-12 8:00	JP	WPRO	52	620	3	15	28	2.419	2.449
6115	2020-3-13 8:00	JP	WPRO	55	675	4	19	29	2.815	2.685
6116	2020-3-14 8:00	JP	WPRO	105	780	3	22	30	2.821	2.819
6117	2020-3-15 8:00	JP	WPRO	0	780	0	22	31	2.821	2.863
6118	2020-3-16 8:00	JP	WPRO	34	814	2	24	32	2.948	3.049
6119	2020-3-17 8:00	JP	WPRO	15	829	4	28	33	3.378	3.216
6120	2020-3-18 8:00	JP	WPRO	44	873	1	29	34	3.322	3.391

Table S1 Daily confirmed cases and daily deaths of COVID-19 in 214 nations from WHO

ID	Date and time	Country or region	Continent	Confirmed patients	Cumulative Confirmed patients	Daily dead patients	Daily cumulative dead patients	Lag days	Daily CFR	3DMA CFR
6121	2020-3-19 8:00	JP	WPRO	77	950	4	33	35	3.474	3.437
6122	2020-3-20 8:00	JP	WPRO	46	996	2	35	36	3.514	3.476
6123	2020-3-21 8:00	JP	WPRO	50	1046	1	36	37	3.442	3.466
6124	2020-3-22 8:00	JP	WPRO	0	1046	0	36	38	3.442	3.549
6125	2020-3-23 8:00	JP	WPRO	43	1089	5	41	39	3.765	3.643
6126	2020-3-24 8:00	JP	WPRO	39	1128	1	42	40	3.723	3.698
6127	2020-3-25 8:00	JP	WPRO	65	1193	1	43	41	3.604	3.604
6128	2020-3-26 8:00	JP	WPRO	98	1291	2	45	42	3.486	3.469
6129	2020-3-27 8:00	JP	WPRO	96	1387	1	46	43	3.317	3.357
6130	2020-3-28 8:00	JP	WPRO	112	1499	3	49	44	3.269	3.219
6131	2020-3-29 8:00	JP	WPRO	194	1693	3	52	45	3.071	3.078
6132	2020-3-30 8:00	JP	WPRO	173	1866	2	54	46	2.894	2.944
6133	2020-3-31 8:00	JP	WPRO	87	1953	2	56	47	2.867	2.793
6134	2020-4-1 8:00	JP	WPRO	225	2178	1	57	48	2.617	2.625
6135	2020-4-2 8:00	JP	WPRO	206	2384	0	57	49	2.391	2.497
6136	2020-4-3 8:00	JP	WPRO	233	2617	8	65	50	2.484	2.409
6137	2020-4-4 8:00	JP	WPRO	318	2935	4	69	51	2.351	2.325
6138	2020-4-5 8:00	JP	WPRO	336	3271	1	70	52	2.140	2.163
6139	2020-4-6 8:00	JP	WPRO	383	3654	3	73	53	1.998	2.062
6140	2020-4-7 8:00	JP	WPRO	252	3906	7	80	54	2.048	1.983
6141	2020-4-8 8:00	JP	WPRO	351	4257	1	81	55	1.903	1.911
6142	2020-4-9 8:00	JP	WPRO	511	4768	4	85	56	1.783	1.777
6143	2020-4-10 8:00	JP	WPRO	579	5347	3	88	57	1.646	1.665
6144	2020-4-11 8:00	JP	WPRO	658	6005	6	94	58	1.565	1.554
6145	2020-4-12 8:00	JP	WPRO	743	6748	4	98	59	1.452	1.475
6146	2020-4-13 8:00	JP	WPRO	507	7255	4	102	60	1.406	1.428
6147	2020-4-14 8:00	JP	WPRO	390	7645	7	109	61	1.426	1.434
6148	2020-4-15 8:00	JP	WPRO	455	8100	10	119	62	1.469	1.493
6149	2020-4-16 8:00	JP	WPRO	482	8582	17	136	63	1.585	1.556
6150	2020-4-17 8:00	JP	WPRO	585	9167	12	148	64	1.614	1.590
6151	2020-4-18 8:00	JP	WPRO	628	9795	6	154	65	1.572	1.580
6152	2020-4-19 8:00	JP	WPRO	566	10361	7	161	66	1.554	1.572
6153	2020-4-20 8:00	JP	WPRO	390	10751	10	171	67	1.591	1.606
6154	2020-4-21 8:00	JP	WPRO	368	11119	15	186	68	1.673	1.891
6155	2020-4-22 8:00	JP	WPRO	377	11496	91	277	69	2.410	2.163
6156	2020-4-23 8:00	JP	WPRO	423	11919	10	287	70	2.408	2.459

Table S1 Daily confirmed cases and daily deaths of COVID-19 in 214 nations from WHO

ID	Date and time	Country or region	Continent	Confirmed patients	Cumulative Confirmed patients	Daily dead patients	Daily cumulative dead patients	Lag days	Daily CFR	3DMA CFR
6157	2020-4-24 8:00	JP	WPRO	469	12388	30	317	71	2.559	2.523
6158	2020-4-25 8:00	JP	WPRO	441	12829	17	334	72	2.603	2.601
6159	2020-4-26 8:00	JP	WPRO	353	13182	14	348	73	2.640	2.622
6160	2020-4-27 8:00	JP	WPRO	203	13385	3	351	74	2.622	2.677
6161	2020-4-28 8:00	JP	WPRO	191	13576	25	376	75	2.770	2.733
6162	2020-4-29 8:00	JP	WPRO	276	13852	13	389	76	2.808	2.841
6163	2020-4-30 8:00	JP	WPRO	236	14088	26	415	77	2.946	2.926
6164	2020-5-1 8:00	JP	WPRO	193	14281	17	432	78	3.025	3.031
6165	2020-5-2 8:00	JP	WPRO	264	14545	22	454	79	3.121	3.073
6166										
6167	2020-3-12 8:00	JE	EURO	2	2	0	0			
6168	2020-3-13 8:00	JE	EURO	0	2	0	0			
6169	2020-3-14 8:00	JE	EURO	0	2	0	0			
6170	2020-3-15 8:00	JE	EURO	0	2	0	0			
6171	2020-3-16 8:00	JE	EURO	0	2	0	0			
6172	2020-3-17 8:00	JE	EURO	3	5	0	0			
6173	2020-3-18 8:00	JE	EURO	0	5	0	0			
6174	2020-3-19 8:00	JE	EURO	0	5	0	0			
6175	2020-3-20 8:00	JE	EURO	7	12	0	0			
6176	2020-3-21 8:00	JE	EURO	0	12	0	0			
6177	2020-3-22 8:00	JE	EURO	0	12	0	0			
6178	2020-3-23 8:00	JE	EURO	3	15	0	0			
6179	2020-3-24 8:00	JE	EURO	1	16	0	0			
6180	2020-3-25 8:00	JE	EURO	0	16	0	0			
6181	2020-3-26 8:00	JE	EURO	2	18	0	0			
6182	2020-3-27 8:00	JE	EURO	14	32	1	1	0	3.125	2.524
6183	2020-3-28 8:00	JE	EURO	20	52	0	1	1	1.923	2.229
6184	2020-3-29 8:00	JE	EURO	9	61	0	1	2	1.639	2.246
6185	2020-3-30 8:00	JE	EURO	2	63	1	2	3	3.175	2.663
6186	2020-3-31 8:00	JE	EURO	0	63	0	2	4	3.175	3.175
6187	2020-4-1 8:00	JE	EURO	0	63	0	2	5	3.175	2.939
6188	2020-4-2 8:00	JE	EURO	18	81	0	2	6	2.469	2.704
6189	2020-4-3 8:00	JE	EURO	0	81	0	2	7	2.469	2.211
6190	2020-4-4 8:00	JE	EURO	37	118	0	2	8	1.695	2.201
6191	2020-4-5 8:00	JE	EURO	5	123	1	3	9	2.439	2.023
6192	2020-4-6 8:00	JE	EURO	32	155	0	3	10	1.935	2.103

Table S1 Daily confirmed cases and daily deaths of COVID-19 in 214 nations from WHO

ID	Date and time	Country or region	Continent	Confirmed patients	Cumulative Confirmed patients	Daily dead patients	Daily cumulative dead patients	Lag days	Daily CFR	3DMA CFR
6193	2020-4-7 8:00	JE	EURO	0	155	0	3	11	1.935	1.879
6194	2020-4-8 8:00	JE	EURO	15	170	0	3	12	1.765	1.822
6195	2020-4-9 8:00	JE	EURO	0	170	0	3	13	1.765	1.765
6196	2020-4-10 8:00	JE	EURO	0	170	0	3	14	1.765	1.905
6197	2020-4-11 8:00	JE	EURO	13	183	1	4	15	2.186	1.990
6198	2020-4-12 8:00	JE	EURO	15	198	0	4	16	2.020	2.075
6199	2020-4-13 8:00	JE	EURO	0	198	0	4	17	2.020	1.973
6200	2020-4-14 8:00	JE	EURO	15	213	0	4	18	1.878	1.914
6201	2020-4-15 8:00	JE	EURO	4	217	0	4	19	1.843	2.162
6202	2020-4-16 8:00	JE	EURO	0	217	2	6	20	2.765	2.602
6203	2020-4-17 8:00	JE	EURO	2	219	1	7	21	3.196	3.482
6204	2020-4-18 8:00	JE	EURO	4	223	3	10	22	4.484	4.127
6205	2020-4-19 8:00	JE	EURO	11	234	1	11	23	4.701	4.694
6206	2020-4-20 8:00	JE	EURO	11	245	1	12	24	4.898	4.806
6207	2020-4-21 8:00	JE	EURO	4	249	0	12	25	4.819	5.106
6208	2020-4-22 8:00	JE	EURO	1	250	2	14	26	5.600	5.303
6209	2020-4-23 8:00	JE	EURO	5	255	0	14	27	5.490	6.050
6210	2020-4-24 8:00	JE	EURO	0	255	4	18	28	7.059	6.478
6211	2020-4-25 8:00	JE	EURO	21	276	1	19	29	6.884	6.926
6212	2020-4-26 8:00	JE	EURO	2	278	0	19	30	6.835	6.835
6213	2020-4-27 8:00	JE	EURO	2	280	0	19	31	6.786	6.794
6214	2020-4-28 8:00	JE	EURO	1	281	0	19	32	6.762	6.754
6215	2020-4-29 8:00	JE	EURO	2	283	0	19	33	6.714	6.839
6216	2020-4-30 8:00	JE	EURO	1	284	1	20	34	7.042	6.884
6217	2020-5-1 8:00	JE	EURO	6	290	0	20	35	6.897	7.175
6218	2020-5-2 8:00	JE	EURO	0	290	2	22	36	7.586	7.241
6219										
6220	2020-3-2 8:00	JO	EMRO	1	1	0	0			
6221	2020-3-3 8:00	JO	EMRO	0	1	0	0			
6222	2020-3-4 8:00	JO	EMRO	0	1	0	0			
6223	2020-3-5 8:00	JO	EMRO	0	1	0	0			
6224	2020-3-6 8:00	JO	EMRO	0	1	0	0			
6225	2020-3-7 8:00	JO	EMRO	0	1	0	0			
6226	2020-3-8 8:00	JO	EMRO	0	1	0	0			
6227	2020-3-9 8:00	JO	EMRO	0	1	0	0			
6228	2020-3-10 8:00	JO	EMRO	0	1	0	0			

Table S1 Daily confirmed cases and daily deaths of COVID-19 in 214 nations from WHO

ID	Date and time	Country or region	Continent	Confirmed patients	Cumulative Confirmed patients	Daily dead patients	Daily cumulative dead patients	Lag days	Daily CFR	3DMA CFR
6229	2020-3-11 8:00	JO	EMRO	0	1	0	0			
6230	2020-3-12 8:00	JO	EMRO	0	1	0	0			
6231	2020-3-13 8:00	JO	EMRO	0	1	0	0			
6232	2020-3-14 8:00	JO	EMRO	0	1	0	0			
6233	2020-3-15 8:00	JO	EMRO	5	6	0	0			
6234	2020-3-16 8:00	JO	EMRO	0	6	0	0			
6235	2020-3-17 8:00	JO	EMRO	29	35	0	0			
6236	2020-3-18 8:00	JO	EMRO	17	52	0	0			
6237	2020-3-19 8:00	JO	EMRO	4	56	0	0			
6238	2020-3-20 8:00	JO	EMRO	13	69	0	0			
6239	2020-3-21 8:00	JO	EMRO	15	84	0	0			
6240	2020-3-22 8:00	JO	EMRO	15	99	0	0			
6241	2020-3-23 8:00	JO	EMRO	13	112	0	0			
6242	2020-3-24 8:00	JO	EMRO	15	127	0	0			
6243	2020-3-25 8:00	JO	EMRO	26	153	0	0			
6244	2020-3-26 8:00	JO	EMRO	19	172	0	0			
6245	2020-3-27 8:00	JO	EMRO	40	212	0	0			
6246	2020-3-28 8:00	JO	EMRO	23	235	1	1	0	0.426	0.416
6247	2020-3-29 8:00	JO	EMRO	11	246	0	1	1	0.407	0.792
6248	2020-3-30 8:00	JO	EMRO	13	259	3	4	2	1.544	1.272
6249	2020-3-31 8:00	JO	EMRO	9	268	1	5	3	1.866	1.745
6250	2020-4-1 8:00	JO	EMRO	6	274	0	5	4	1.825	1.830
6251	2020-4-2 8:00	JO	EMRO	4	278	0	5	5	1.799	1.765
6252	2020-4-3 8:00	JO	EMRO	21	299	0	5	6	1.672	1.695
6253	2020-4-4 8:00	JO	EMRO	11	310	0	5	7	1.613	1.611
6254	2020-4-5 8:00	JO	EMRO	13	323	0	5	8	1.548	1.537
6255	2020-4-6 8:00	JO	EMRO	22	345	0	5	9	1.449	1.572
6256	2020-4-7 8:00	JO	EMRO	4	349	1	6	10	1.719	1.623
6257	2020-4-8 8:00	JO	EMRO	4	353	0	6	11	1.700	1.698
6258	2020-4-9 8:00	JO	EMRO	5	358	0	6	12	1.676	1.752
6259	2020-4-10 8:00	JO	EMRO	14	372	1	7	13	1.882	1.813
6260	2020-4-11 8:00	JO	EMRO	0	372	0	7	14	1.882	1.867
6261	2020-4-12 8:00	JO	EMRO	9	381	0	7	15	1.837	1.839
6262	2020-4-13 8:00	JO	EMRO	8	389	0	7	16	1.799	1.809
6263	2020-4-14 8:00	JO	EMRO	2	391	0	7	17	1.790	1.784
6264	2020-4-15 8:00	JO	EMRO	6	397	0	7	18	1.763	1.766

Table S1 Daily confirmed cases and daily deaths of COVID-19 in 214 nations from WHO

ID	Date and time	Country or region	Continent	Confirmed patients	Cumulative Confirmed patients	Daily dead patients	Daily cumulative dead patients	Lag days	Daily CFR	3DMA CFR
6265	2020-4-16 8:00	JO	EMRO	4	401	0	7	19	1.746	1.750
6266	2020-4-17 8:00	JO	EMRO	1	402	0	7	20	1.741	1.736
6267	2020-4-18 8:00	JO	EMRO	5	407	0	7	21	1.720	1.719
6268	2020-4-19 8:00	JO	EMRO	6	413	0	7	22	1.695	1.698
6269	2020-4-20 8:00	JO	EMRO	4	417	0	7	23	1.679	1.674
6270	2020-4-21 8:00	JO	EMRO	8	425	0	7	24	1.647	1.654
6271	2020-4-22 8:00	JO	EMRO	3	428	0	7	25	1.636	1.631
6272	2020-4-23 8:00	JO	EMRO	7	435	0	7	26	1.609	1.616
6273	2020-4-24 8:00	JO	EMRO	2	437	0	7	27	1.602	1.596
6274	2020-4-25 8:00	JO	EMRO	7	444	0	7	28	1.577	1.581
6275	2020-4-26 8:00	JO	EMRO	3	447	0	7	29	1.566	1.567
6276	2020-4-27 8:00	JO	EMRO	2	449	0	7	30	1.559	1.636
6277	2020-4-28 8:00	JO	EMRO	0	449	1	8	31	1.782	1.707
6278	2020-4-29 8:00	JO	EMRO	0	449	0	8	32	1.782	1.776
6279	2020-4-30 8:00	JO	EMRO	4	453	0	8	33	1.766	1.771
6280	2020-5-1 8:00	JO	EMRO	0	453	0	8	34	1.766	1.758
6281	2020-5-2 8:00	JO	EMRO	6	459	0	8	35	1.743	1.754
6282										
6283	2020-3-14 8:00	KZ	EURO	6	6	0	0			
6284	2020-3-15 8:00	KZ	EURO	0	6	0	0			
6285	2020-3-16 8:00	KZ	EURO	0	6	0	0			
6286	2020-3-17 8:00	KZ	EURO	27	33	0	0			
6287	2020-3-18 8:00	KZ	EURO	3	36	0	0			
6288	2020-3-19 8:00	KZ	EURO	10	46	0	0			
6289	2020-3-20 8:00	KZ	EURO	7	53	0	0			
6290	2020-3-21 8:00	KZ	EURO	3	56	0	0			
6291	2020-3-22 8:00	KZ	EURO	0	56	0	0			
6292	2020-3-23 8:00	KZ	EURO	4	60	0	0			
6293	2020-3-24 8:00	KZ	EURO	3	63	0	0			
6294	2020-3-25 8:00	KZ	EURO	16	79	0	0			
6295	2020-3-26 8:00	KZ	EURO	18	97	0	0			
6296	2020-3-27 8:00	KZ	EURO	28	125	0	0			
6297	2020-3-28 8:00	KZ	EURO	79	204	1	1	0	0.490	0.434
6298	2020-3-29 8:00	KZ	EURO	61	265	0	1	1	0.377	0.403
6299	2020-3-30 8:00	KZ	EURO	29	294	0	1	2	0.340	0.346
6300	2020-3-31 8:00	KZ	EURO	18	312	0	1	3	0.321	0.412

Table S1 Daily confirmed cases and daily deaths of COVID-19 in 214 nations from WHO

ID	Date and time	Country or region	Continent	Confirmed patients	Cumulative Confirmed patients	Daily dead patients	Daily cumulative dead patients	Lag days	Daily CFR	3DMA CFR
6301	2020-4-1 8:00	KZ	EURO	36	348	1	2	4	0.575	0.557
6302	2020-4-2 8:00	KZ	EURO	38	386	1	3	5	0.777	0.681
6303	2020-4-3 8:00	KZ	EURO	49	435	0	3	6	0.690	0.706
6304	2020-4-4 8:00	KZ	EURO	25	460	0	3	7	0.652	0.761
6305	2020-4-5 8:00	KZ	EURO	71	531	2	5	8	0.942	0.807
6306	2020-4-6 8:00	KZ	EURO	73	604	0	5	9	0.828	0.888
6307	2020-4-7 8:00	KZ	EURO	66	670	1	6	10	0.896	0.857
6308	2020-4-8 8:00	KZ	EURO	39	709	0	6	11	0.846	0.902
6309	2020-4-9 8:00	KZ	EURO	18	727	1	7	12	0.963	0.977
6310	2020-4-10 8:00	KZ	EURO	75	802	2	9	13	1.122	1.083
6311	2020-4-11 8:00	KZ	EURO	57	859	1	10	14	1.164	1.134
6312	2020-4-12 8:00	KZ	EURO	38	897	0	10	15	1.115	1.168
6313	2020-4-13 8:00	KZ	EURO	82	979	2	12	16	1.226	1.147
6314	2020-4-14 8:00	KZ	EURO	112	1091	0	12	17	1.100	1.089
6315	2020-4-15 8:00	KZ	EURO	184	1275	0	12	18	0.941	1.092
6316	2020-4-16 8:00	KZ	EURO	20	1295	4	16	19	1.236	1.086
6317	2020-4-17 8:00	KZ	EURO	185	1480	0	16	20	1.081	1.139
6318	2020-4-18 8:00	KZ	EURO	66	1546	1	17	21	1.100	1.093
6319	2020-4-19 8:00	KZ	EURO	0	1546	0	17	22	1.100	1.100
6320	2020-4-20 8:00	KZ	EURO	0	1546	0	17	23	1.100	1.075
6321	2020-4-21 8:00	KZ	EURO	306	1852	2	19	24	1.026	1.026
6322	2020-4-22 8:00	KZ	EURO	143	1995	0	19	25	0.952	0.972
6323	2020-4-23 8:00	KZ	EURO	30	2025	0	19	26	0.938	0.943
6324	2020-4-24 8:00	KZ	EURO	0	2025	0	19	27	0.938	0.970
6325	2020-4-25 8:00	KZ	EURO	391	2416	6	25	28	1.035	0.978
6326	2020-4-26 8:00	KZ	EURO	185	2601	0	25	29	0.961	0.964
6327	2020-4-27 8:00	KZ	EURO	190	2791	0	25	30	0.896	0.898
6328	2020-4-28 8:00	KZ	EURO	191	2982	0	25	31	0.838	0.849
6329	2020-4-29 8:00	KZ	EURO	96	3078	0	25	32	0.812	0.800
6330	2020-4-30 8:00	KZ	EURO	255	3333	0	25	33	0.750	0.755
6331	2020-5-1 8:00	KZ	EURO	218	3551	0	25	34	0.704	0.705
6332	2020-5-2 8:00	KZ	EURO	234	3785	0	25	35	0.661	0.682
6333										
6334	2020-3-14 8:00	KE	AFRO	1	1	0	0			
6335	2020-3-15 8:00	KE	AFRO	0	1	0	0			
6336	2020-3-16 8:00	KE	AFRO	2	3	0	0			

Table S1 Daily confirmed cases and daily deaths of COVID-19 in 214 nations from WHO

ID	Date and time	Country or region	Continent	Confirmed patients	Cumulative Confirmed patients	Daily dead patients	Daily cumulative dead patients	Lag days	Daily CFR	3DMA CFR
6337	2020-3-17 8:00	KE	AFRO	0	3	0	0			
6338	2020-3-18 8:00	KE	AFRO	4	7	0	0			
6339	2020-3-19 8:00	KE	AFRO	0	7	0	0			
6340	2020-3-20 8:00	KE	AFRO	0	7	0	0			
6341	2020-3-21 8:00	KE	AFRO	0	7	0	0			
6342	2020-3-22 8:00	KE	AFRO	0	7	0	0			
6343	2020-3-23 8:00	KE	AFRO	8	15	0	0			
6344	2020-3-24 8:00	KE	AFRO	1	16	0	0			
6345	2020-3-25 8:00	KE	AFRO	9	25	0	0			
6346	2020-3-26 8:00	KE	AFRO	0	25	0	0			
6347	2020-3-27 8:00	KE	AFRO	0	25	1	1	0	4.000	4.000
6348	2020-3-28 8:00	KE	AFRO	0	25	0	1	1	4.000	4.000
6349	2020-3-29 8:00	KE	AFRO	0	25	0	1	2	4.000	3.544
6350	2020-3-30 8:00	KE	AFRO	13	38	0	1	3	2.632	2.877
6351	2020-3-31 8:00	KE	AFRO	12	50	0	1	4	2.000	2.109
6352	2020-4-1 8:00	KE	AFRO	9	59	0	1	5	1.695	1.643
6353	2020-4-2 8:00	KE	AFRO	22	81	0	1	6	1.235	1.886
6354	2020-4-3 8:00	KE	AFRO	29	110	2	3	7	2.727	2.414
6355	2020-4-4 8:00	KE	AFRO	12	122	1	4	8	3.279	3.095
6356	2020-4-5 8:00	KE	AFRO	0	122	0	4	9	3.279	3.125
6357	2020-4-6 8:00	KE	AFRO	20	142	0	4	10	2.817	2.971
6358	2020-4-7 8:00	KE	AFRO	0	142	0	4	11	2.817	3.041
6359	2020-4-8 8:00	KE	AFRO	30	172	2	6	12	3.488	3.219
6360	2020-4-9 8:00	KE	AFRO	7	179	0	6	13	3.352	3.548
6361	2020-4-10 8:00	KE	AFRO	5	184	1	7	14	3.804	3.620
6362	2020-4-11 8:00	KE	AFRO	5	189	0	7	15	3.704	3.724
6363	2020-4-12 8:00	KE	AFRO	2	191	0	7	16	3.665	3.810
6364	2020-4-13 8:00	KE	AFRO	6	197	1	8	17	4.061	4.018
6365	2020-4-14 8:00	KE	AFRO	11	208	1	9	18	4.327	4.185
6366	2020-4-15 8:00	KE	AFRO	8	216	0	9	19	4.167	4.313
6367	2020-4-16 8:00	KE	AFRO	9	225	1	10	20	4.444	4.437
6368	2020-4-17 8:00	KE	AFRO	9	234	1	11	21	4.701	4.539
6369	2020-4-18 8:00	KE	AFRO	12	246	0	11	22	4.472	4.584
6370	2020-4-19 8:00	KE	AFRO	16	262	1	12	23	4.580	4.746
6371	2020-4-20 8:00	KE	AFRO	8	270	2	14	24	5.185	4.916
6372	2020-4-21 8:00	KE	AFRO	11	281	0	14	25	4.982	4.966

Table S1 Daily confirmed cases and daily deaths of COVID-19 in 214 nations from WHO

ID	Date and time	Country or region	Continent	Confirmed patients	Cumulative Confirmed patients	Daily dead patients	Daily cumulative dead patients	Lag days	Daily CFR	3DMA CFR
6373	2020-4-22 8:00	KE	AFRO	15	296	0	14	26	4.730	4.777
6374	2020-4-23 8:00	KE	AFRO	7	303	0	14	27	4.620	4.575
6375	2020-4-24 8:00	KE	AFRO	17	320	0	14	28	4.375	4.387
6376	2020-4-25 8:00	KE	AFRO	16	336	0	14	29	4.167	4.208
6377	2020-4-26 8:00	KE	AFRO	7	343	0	14	30	4.082	4.064
6378	2020-4-27 8:00	KE	AFRO	12	355	0	14	31	3.944	3.961
6379	2020-4-28 8:00	KE	AFRO	8	363	0	14	32	3.857	3.848
6380	2020-4-29 8:00	KE	AFRO	11	374	0	14	33	3.743	3.835
6381	2020-4-30 8:00	KE	AFRO	10	384	1	15	34	3.906	3.981
6382	2020-5-1 8:00	KE	AFRO	12	396	2	17	35	4.293	4.436
6383	2020-5-2 8:00	KE	AFRO	15	411	4	21	36	5.109	4.701
6384										
6385	2020-3-13 8:00	XK	EURO	2	2	0	0			
6386	2020-3-14 8:00	XK	EURO	3	5	0	0			
6387	2020-3-15 8:00	XK	EURO	0	5	0	0			
6388	2020-3-16 8:00	XK	EURO	0	5	0	0			
6389	2020-3-17 8:00	XK	EURO	8	13	0	0			
6390	2020-3-18 8:00	XK	EURO	0	13	0	0			
6391	2020-3-19 8:00	XK	EURO	8	21	0	0			
6392	2020-3-20 8:00	XK	EURO	3	24	0	0			
6393	2020-3-21 8:00	XK	EURO	0	24	0	0			
6394	2020-3-22 8:00	XK	EURO	0	24	0	0			
6395	2020-3-23 8:00	XK	EURO	7	31	1	1	0	3.226	2.433
6396	2020-3-24 8:00	XK	EURO	30	61	0	1	1	1.639	2.151
6397	2020-3-25 8:00	XK	EURO	2	63	0	1	2	1.587	1.545
6398	2020-3-26 8:00	XK	EURO	8	71	0	1	3	1.408	1.421
6399	2020-3-27 8:00	XK	EURO	8	79	0	1	4	1.266	1.270
6400	2020-3-28 8:00	XK	EURO	9	88	0	1	5	1.136	1.167
6401	2020-3-29 8:00	XK	EURO	3	91	0	1	6	1.099	1.100
6402	2020-3-30 8:00	XK	EURO	3	94	0	1	7	1.064	1.035
6403	2020-3-31 8:00	XK	EURO	12	106	0	1	8	0.943	0.967
6404	2020-4-1 8:00	XK	EURO	6	112	0	1	9	0.893	0.879
6405	2020-4-2 8:00	XK	EURO	13	125	0	1	10	0.800	0.829
6406	2020-4-3 8:00	XK	EURO	1	126	0	1	11	0.794	0.784
6407	2020-4-4 8:00	XK	EURO	6	132	0	1	12	0.758	0.755
6408	2020-4-5 8:00	XK	EURO	8	140	0	1	13	0.714	0.721

Table S1 Daily confirmed cases and daily deaths of COVID-19 in 214 nations from WHO

ID	Date and time	Country or region	Continent	Confirmed patients	Cumulative Confirmed patients	Daily dead patients	Daily cumulative dead patients	Lag days	Daily CFR	3DMA CFR
6409	2020-4-6 8:00	XK	EURO	5	145	0	1	14	0.690	1.074
6410	2020-4-7 8:00	XK	EURO	20	165	2	3	15	1.818	1.742
6411	2020-4-8 8:00	XK	EURO	19	184	2	5	16	2.717	2.405
6412	2020-4-9 8:00	XK	EURO	40	224	1	6	17	2.679	2.827
6413	2020-4-10 8:00	XK	EURO	3	227	1	7	18	3.084	2.854
6414	2020-4-11 8:00	XK	EURO	23	250	0	7	19	2.800	2.786
6415	2020-4-12 8:00	XK	EURO	33	283	0	7	20	2.473	2.402
6416	2020-4-13 8:00	XK	EURO	79	362	0	7	21	1.934	2.176
6417	2020-4-14 8:00	XK	EURO	15	377	1	8	22	2.122	2.041
6418	2020-4-15 8:00	XK	EURO	10	387	0	8	23	2.067	2.152
6419	2020-4-16 8:00	XK	EURO	10	397	1	9	24	2.267	2.261
6420	2020-4-17 8:00	XK	EURO	52	449	2	11	25	2.450	2.406
6421	2020-4-18 8:00	XK	EURO	31	480	1	12	26	2.500	2.434
6422	2020-4-19 8:00	XK	EURO	30	510	0	12	27	2.353	2.365
6423	2020-4-20 8:00	XK	EURO	25	535	0	12	28	2.243	2.231
6424	2020-4-21 8:00	XK	EURO	37	572	0	12	29	2.098	2.440
6425	2020-4-22 8:00	XK	EURO	32	604	6	18	30	2.980	2.645
6426	2020-4-23 8:00	XK	EURO	26	630	0	18	31	2.857	2.892
6427	2020-4-24 8:00	XK	EURO	39	669	1	19	32	2.840	2.800
6428	2020-4-25 8:00	XK	EURO	34	703	0	19	33	2.703	2.760
6429	2020-4-26 8:00	XK	EURO	28	731	1	20	34	2.736	2.730
6430	2020-4-27 8:00	XK	EURO	32	763	1	21	35	2.752	2.770
6431	2020-4-28 8:00	XK	EURO	17	780	1	22	36	2.821	2.786
6432	2020-4-29 8:00	XK	EURO	10	790	0	22	37	2.785	2.786
6433	2020-4-30 8:00	XK	EURO	9	799	0	22	38	2.753	2.764
6434	2020-5-1 8:00	XK	EURO	0	799	0	22	39	2.753	2.738
6435	2020-5-2 8:00	XK	EURO	14	813	0	22	40	2.706	2.730
6436										
6437	2020-2-24 8:00	KW	EMRO	5	5	0	0			
6438	2020-2-25 8:00	KW	EMRO	4	9	0	0			
6439	2020-2-26 8:00	KW	EMRO	17	26	0	0			
6440	2020-2-27 8:00	KW	EMRO	17	43	0	0			
6441	2020-2-28 8:00	KW	EMRO	2	45	0	0			
6442	2020-2-29 8:00	KW	EMRO	0	45	0	0			
6443	2020-3-1 8:00	KW	EMRO	0	45	0	0			
6444	2020-3-2 8:00	KW	EMRO	11	56	0	0			

Table S1 Daily confirmed cases and daily deaths of COVID-19 in 214 nations from WHO

ID	Date and time	Country or region	Continent	Confirmed patients	Cumulative Confirmed patients	Daily dead patients	Daily cumulative dead patients	Lag days	Daily CFR	3DMA CFR
6445	2020-3-3 8:00	KW	EMRO	0	56	0	0			
6446	2020-3-4 8:00	KW	EMRO	0	56	0	0			
6447	2020-3-5 8:00	KW	EMRO	2	58	0	0			
6448	2020-3-6 8:00	KW	EMRO	0	58	0	0			
6449	2020-3-7 8:00	KW	EMRO	3	61	0	0			
6450	2020-3-8 8:00	KW	EMRO	3	64	0	0			
6451	2020-3-9 8:00	KW	EMRO	0	64	0	0			
6452	2020-3-10 8:00	KW	EMRO	5	69	0	0			
6453	2020-3-11 8:00	KW	EMRO	3	72	0	0			
6454	2020-3-12 8:00	KW	EMRO	8	80	0	0			
6455	2020-3-13 8:00	KW	EMRO	20	100	0	0			
6456	2020-3-14 8:00	KW	EMRO	4	104	0	0			
6457	2020-3-15 8:00	KW	EMRO	8	112	0	0			
6458	2020-3-16 8:00	KW	EMRO	11	123	0	0			
6459	2020-3-17 8:00	KW	EMRO	7	130	0	0			
6460	2020-3-18 8:00	KW	EMRO	12	142	0	0			
6461	2020-3-19 8:00	KW	EMRO	6	148	0	0			
6462	2020-3-20 8:00	KW	EMRO	11	159	0	0			
6463	2020-3-21 8:00	KW	EMRO	17	176	0	0			
6464	2020-3-22 8:00	KW	EMRO	12	188	0	0			
6465	2020-3-23 8:00	KW	EMRO	1	189	0	0			
6466	2020-3-24 8:00	KW	EMRO	2	191	0	0			
6467	2020-3-25 8:00	KW	EMRO	4	195	0	0			
6468	2020-3-26 8:00	KW	EMRO	13	208	0	0			
6469	2020-3-27 8:00	KW	EMRO	17	225	0	0			
6470	2020-3-28 8:00	KW	EMRO	10	235	0	0			
6471	2020-3-29 8:00	KW	EMRO	20	255	0	0			
6472	2020-3-30 8:00	KW	EMRO	11	266	0	0			
6473	2020-3-31 8:00	KW	EMRO	23	289	0	0			
6474	2020-4-1 8:00	KW	EMRO	28	317	0	0			
6475	2020-4-2 8:00	KW	EMRO	25	342	0	0			
6476	2020-4-3 8:00	KW	EMRO	75	417	0	0			
6477	2020-4-4 8:00	KW	EMRO	62	479	1	1	0	0.209	0.194
6478	2020-4-5 8:00	KW	EMRO	77	556	0	1	1	0.180	0.180
6479	2020-4-6 8:00	KW	EMRO	109	665	0	1	2	0.150	0.155
6480	2020-4-7 8:00	KW	EMRO	78	743	0	1	3	0.135	0.134

Table S1 Daily confirmed cases and daily deaths of COVID-19 in 214 nations from WHO

ID	Date and time	Country or region	Continent	Confirmed patients	Cumulative Confirmed patients	Daily dead patients	Daily cumulative dead patients	Lag days	Daily CFR	3DMA CFR
6481	2020-4-8 8:00	KW	EMRO	112	855	0	1	4	0.117	0.120
6482	2020-4-9 8:00	KW	EMRO	55	910	0	1	5	0.110	0.109
6483	2020-4-10 8:00	KW	EMRO	83	993	0	1	6	0.101	0.099
6484	2020-4-11 8:00	KW	EMRO	161	1154	0	1	7	0.087	0.089
6485	2020-4-12 8:00	KW	EMRO	80	1234	0	1	8	0.081	0.107
6486	2020-4-13 8:00	KW	EMRO	66	1300	1	2	9	0.154	0.152
6487	2020-4-14 8:00	KW	EMRO	55	1355	1	3	10	0.221	0.196
6488	2020-4-15 8:00	KW	EMRO	50	1405	0	3	11	0.214	0.211
6489	2020-4-16 8:00	KW	EMRO	119	1524	0	3	12	0.197	0.237
6490	2020-4-17 8:00	KW	EMRO	134	1658	2	5	13	0.302	0.280
6491	2020-4-18 8:00	KW	EMRO	93	1751	1	6	14	0.343	0.337
6492	2020-4-19 8:00	KW	EMRO	164	1915	1	7	15	0.366	0.386
6493	2020-4-20 8:00	KW	EMRO	80	1995	2	9	16	0.451	0.449
6494	2020-4-21 8:00	KW	EMRO	85	2080	2	11	17	0.529	0.519
6495	2020-4-22 8:00	KW	EMRO	168	2248	2	13	18	0.578	0.564
6496	2020-4-23 8:00	KW	EMRO	151	2399	1	14	19	0.584	0.579
6497	2020-4-24 8:00	KW	EMRO	215	2614	1	15	20	0.574	0.605
6498	2020-4-25 8:00	KW	EMRO	278	2892	4	19	21	0.657	0.627
6499	2020-4-26 8:00	KW	EMRO	183	3075	1	20	22	0.650	0.659
6500	2020-4-27 8:00	KW	EMRO	213	3288	2	22	23	0.669	0.663
6501	2020-4-28 8:00	KW	EMRO	152	3440	1	23	24	0.669	0.660
6502	2020-4-29 8:00	KW	EMRO	300	3740	1	24	25	0.642	0.652
6503	2020-4-30 8:00	KW	EMRO	284	4024	2	26	26	0.646	0.658
6504	2020-5-1 8:00	KW	EMRO	353	4377	4	30	27	0.685	0.672
6505	2020-5-2 8:00	KW	EMRO	0	4377	0	30	28	0.685	0.685
6506										
6507	2020-3-18 8:00	KG	EURO	3	3	0	0			
6508	2020-3-19 8:00	KG	EURO	0	3	0	0			
6509	2020-3-20 8:00	KG	EURO	3	6	0	0			
6510	2020-3-21 8:00	KG	EURO	8	14	0	0			
6511	2020-3-22 8:00	KG	EURO	0	14	0	0			
6512	2020-3-23 8:00	KG	EURO	0	14	0	0			
6513	2020-3-24 8:00	KG	EURO	2	16	0	0			
6514	2020-3-25 8:00	KG	EURO	26	42	0	0			
6515	2020-3-26 8:00	KG	EURO	2	44	0	0			
6516	2020-3-27 8:00	KG	EURO	14	58	0	0			

Table S1 Daily confirmed cases and daily deaths of COVID-19 in 214 nations from WHO

ID	Date and time	Country or region	Continent	Confirmed patients	Cumulative Confirmed patients	Daily dead patients	Daily cumulative dead patients	Lag days	Daily CFR	3DMA CFR
6517	2020-3-28 8:00	KG	EURO	0	58	0	0			
6518	2020-3-29 8:00	KG	EURO	26	84	0	0			
6519	2020-3-30 8:00	KG	EURO	0	84	0	0			
6520	2020-3-31 8:00	KG	EURO	23	107	0	0			
6521	2020-4-1 8:00	KG	EURO	4	111	0	0			
6522	2020-4-2 8:00	KG	EURO	4	115	0	0			
6523	2020-4-3 8:00	KG	EURO	15	130	1	1	0	0.769	0.732
6524	2020-4-4 8:00	KG	EURO	14	144	0	1	1	0.694	0.715
6525	2020-4-5 8:00	KG	EURO	3	147	0	1	2	0.680	1.076
6526	2020-4-6 8:00	KG	EURO	69	216	3	4	3	1.852	1.429
6527	2020-4-7 8:00	KG	EURO	12	228	0	4	4	1.754	1.696
6528	2020-4-8 8:00	KG	EURO	42	270	0	4	5	1.481	1.555
6529	2020-4-9 8:00	KG	EURO	10	280	0	4	6	1.429	1.529
6530	2020-4-10 8:00	KG	EURO	18	298	1	5	7	1.678	1.527
6531	2020-4-11 8:00	KG	EURO	41	339	0	5	8	1.475	1.493
6532	2020-4-12 8:00	KG	EURO	38	377	0	5	9	1.326	1.332
6533	2020-4-13 8:00	KG	EURO	42	419	0	5	10	1.193	1.227
6534	2020-4-14 8:00	KG	EURO	11	430	0	5	11	1.163	1.157
6535	2020-4-15 8:00	KG	EURO	19	449	0	5	12	1.114	1.116
6536	2020-4-16 8:00	KG	EURO	17	466	0	5	13	1.073	1.070
6537	2020-4-17 8:00	KG	EURO	23	489	0	5	14	1.022	1.028
6538	2020-4-18 8:00	KG	EURO	17	506	0	5	15	0.988	0.971
6539	2020-4-19 8:00	KG	EURO	48	554	0	5	16	0.903	1.041
6540	2020-4-20 8:00	KG	EURO	14	568	2	7	17	1.232	1.107
6541	2020-4-21 8:00	KG	EURO	22	590	0	7	18	1.186	1.188
6542	2020-4-22 8:00	KG	EURO	22	612	0	7	19	1.144	1.199
6543	2020-4-23 8:00	KG	EURO	19	631	1	8	20	1.268	1.210
6544	2020-4-24 8:00	KG	EURO	25	656	0	8	21	1.220	1.230
6545	2020-4-25 8:00	KG	EURO	9	665	0	8	22	1.203	1.199
6546	2020-4-26 8:00	KG	EURO	17	682	0	8	23	1.173	1.176
6547	2020-4-27 8:00	KG	EURO	13	695	0	8	24	1.151	1.151
6548	2020-4-28 8:00	KG	EURO	13	708	0	8	25	1.130	1.126
6549	2020-4-29 8:00	KG	EURO	21	729	0	8	26	1.097	1.100
6550	2020-4-30 8:00	KG	EURO	17	746	0	8	27	1.072	1.076
6551	2020-5-1 8:00	KG	EURO	10	756	0	8	28	1.058	1.057
6552	2020-5-2 8:00	KG	EURO	13	769	0	8	29	1.040	1.049

Table S1 Daily confirmed cases and daily deaths of COVID-19 in 214 nations from WHO

ID	Date and time	Country or region	Continent	Confirmed patients	Cumulative Confirmed patients	Daily dead patients	Daily cumulative dead patients	Lag days	Daily CFR	3DMA CFR
6553										
6554	2020-3-24 8:00	LA	WPRO	2	2	0	0			
6555	2020-3-25 8:00	LA	WPRO	0	2	0	0			
6556	2020-3-26 8:00	LA	WPRO	1	3	0	0			
6557	2020-3-27 8:00	LA	WPRO	3	6	0	0			
6558	2020-3-28 8:00	LA	WPRO	0	6	0	0			
6559	2020-3-29 8:00	LA	WPRO	0	6	0	0			
6560	2020-3-30 8:00	LA	WPRO	2	8	0	0			
6561	2020-3-31 8:00	LA	WPRO	1	9	0	0			
6562	2020-4-1 8:00	LA	WPRO	0	9	0	0			
6563	2020-4-2 8:00	LA	WPRO	1	10	0	0			
6564	2020-4-3 8:00	LA	WPRO	0	10	0	0			
6565	2020-4-4 8:00	LA	WPRO	0	10	0	0			
6566	2020-4-5 8:00	LA	WPRO	0	10	0	0			
6567	2020-4-6 8:00	LA	WPRO	1	11	0	0			
6568	2020-4-7 8:00	LA	WPRO	1	12	0	0			
6569	2020-4-8 8:00	LA	WPRO	2	14	0	0			
6570	2020-4-9 8:00	LA	WPRO	1	15	0	0			
6571	2020-4-10 8:00	LA	WPRO	1	16	0	0			
6572	2020-4-11 8:00	LA	WPRO	0	16	0	0			
6573	2020-4-12 8:00	LA	WPRO	2	18	0	0			
6574	2020-4-13 8:00	LA	WPRO	1	19	0	0			
6575	2020-4-14 8:00	LA	WPRO	0	19	0	0			
6576	2020-4-15 8:00	LA	WPRO	0	19	0	0			
6577	2020-4-16 8:00	LA	WPRO	0	19	0	0			
6578	2020-4-17 8:00	LA	WPRO	0	19	0	0			
6579	2020-4-18 8:00	LA	WPRO	0	19	0	0			
6580	2020-4-19 8:00	LA	WPRO	0	19	0	0			
6581	2020-4-20 8:00	LA	WPRO	0	19	0	0			
6582	2020-4-21 8:00	LA	WPRO	0	19	0	0			
6583	2020-4-22 8:00	LA	WPRO	0	19	0	0			
6584	2020-4-23 8:00	LA	WPRO	0	19	0	0			
6585	2020-4-24 8:00	LA	WPRO	0	19	0	0			
6586	2020-4-25 8:00	LA	WPRO	0	19	0	0			
6587	2020-4-26 8:00	LA	WPRO	0	19	0	0			
6588	2020-4-27 8:00	LA	WPRO	0	19	0	0			

Table S1 Daily confirmed cases and daily deaths of COVID-19 in 214 nations from WHO

ID	Date and time	Country or region	Continent	Confirmed patients	Cumulative Confirmed patients	Daily dead patients	Daily cumulative dead patients	Lag days	Daily CFR	3DMA CFR
6589	2020-4-28 8:00	LA	WPRO	0	19	0	0			
6590	2020-4-29 8:00	LA	WPRO	0	19	0	0			
6591	2020-4-30 8:00	LA	WPRO	0	19	0	0			
6592	2020-5-1 8:00	LA	WPRO	0	19	0	0			
6593	2020-5-2 8:00	LA	WPRO	0	19	0	0			
6594										
6595	2020-3-2 8:00	LV	EURO	1	1	0	0			
6596	2020-3-3 8:00	LV	EURO	0	1	0	0			
6597	2020-3-4 8:00	LV	EURO	0	1	0	0			
6598	2020-3-5 8:00	LV	EURO	0	1	0	0			
6599	2020-3-6 8:00	LV	EURO	0	1	0	0			
6600	2020-3-7 8:00	LV	EURO	0	1	0	0			
6601	2020-3-8 8:00	LV	EURO	2	3	0	0			
6602	2020-3-9 8:00	LV	EURO	3	6	0	0			
6603	2020-3-10 8:00	LV	EURO	2	8	0	0			
6604	2020-3-11 8:00	LV	EURO	2	10	0	0			
6605	2020-3-12 8:00	LV	EURO	6	16	0	0			
6606	2020-3-13 8:00	LV	EURO	3	19	0	0			
6607	2020-3-14 8:00	LV	EURO	0	19	0	0			
6608	2020-3-15 8:00	LV	EURO	12	31	0	0			
6609	2020-3-16 8:00	LV	EURO	5	36	0	0			
6610	2020-3-17 8:00	LV	EURO	24	60	0	0			
6611	2020-3-18 8:00	LV	EURO	11	71	0	0			
6612	2020-3-19 8:00	LV	EURO	15	86	0	0			
6613	2020-3-20 8:00	LV	EURO	25	111	0	0			
6614	2020-3-21 8:00	LV	EURO	13	124	0	0			
6615	2020-3-22 8:00	LV	EURO	0	124	0	0			
6616	2020-3-23 8:00	LV	EURO	15	139	0	0			
6617	2020-3-24 8:00	LV	EURO	41	180	0	0			
6618	2020-3-25 8:00	LV	EURO	17	197	0	0			
6619	2020-3-26 8:00	LV	EURO	24	221	0	0			
6620	2020-3-27 8:00	LV	EURO	23	244	0	0			
6621	2020-3-28 8:00	LV	EURO	36	280	0	0			
6622	2020-3-29 8:00	LV	EURO	25	305	0	0			
6623	2020-3-30 8:00	LV	EURO	71	376	0	0			
6624	2020-3-31 8:00	LV	EURO	0	376	0	0			

Table S1 Daily confirmed cases and daily deaths of COVID-19 in 214 nations from WHO

ID	Date and time	Country or region	Continent	Confirmed patients	Cumulative Confirmed patients	Daily dead patients	Daily cumulative dead patients	Lag days	Daily CFR	3DMA CFR
6625	2020-4-1 8:00	LV	EURO	22	398	0	0			
6626	2020-4-2 8:00	LV	EURO	48	446	0	0			
6627	2020-4-3 8:00	LV	EURO	12	458	0	0			
6628	2020-4-4 8:00	LV	EURO	35	493	1	1	0	0.203	0.200
6629	2020-4-5 8:00	LV	EURO	16	509	0	1	1	0.196	0.196
6630	2020-4-6 8:00	LV	EURO	24	533	0	1	2	0.188	0.190
6631	2020-4-7 8:00	LV	EURO	9	542	0	1	3	0.185	0.246
6632	2020-4-8 8:00	LV	EURO	6	548	1	2	4	0.365	0.299
6633	2020-4-9 8:00	LV	EURO	29	577	0	2	5	0.347	0.350
6634	2020-4-10 8:00	LV	EURO	12	589	0	2	6	0.340	0.338
6635	2020-4-11 8:00	LV	EURO	23	612	0	2	7	0.327	0.381
6636	2020-4-12 8:00	LV	EURO	18	630	1	3	8	0.476	0.524
6637	2020-4-13 8:00	LV	EURO	21	651	2	5	9	0.768	0.669
6638	2020-4-14 8:00	LV	EURO	4	655	0	5	10	0.763	0.764
6639	2020-4-15 8:00	LV	EURO	2	657	0	5	11	0.761	0.758
6640	2020-4-16 8:00	LV	EURO	9	666	0	5	12	0.751	0.751
6641	2020-4-17 8:00	LV	EURO	9	675	0	5	13	0.741	0.742
6642	2020-4-18 8:00	LV	EURO	7	682	0	5	14	0.733	0.725
6643	2020-4-19 8:00	LV	EURO	30	712	0	5	15	0.702	0.708
6644	2020-4-20 8:00	LV	EURO	15	727	0	5	16	0.688	0.689
6645	2020-4-21 8:00	LV	EURO	12	739	0	5	17	0.677	0.856
6646	2020-4-22 8:00	LV	EURO	9	748	4	9	18	1.203	1.108
6647	2020-4-23 8:00	LV	EURO	13	761	2	11	19	1.445	1.354
6648	2020-4-24 8:00	LV	EURO	17	778	0	11	20	1.414	1.463
6649	2020-4-25 8:00	LV	EURO	6	784	1	12	21	1.531	1.479
6650	2020-4-26 8:00	LV	EURO	20	804	0	12	22	1.493	1.500
6651	2020-4-27 8:00	LV	EURO	8	812	0	12	23	1.478	1.520
6652	2020-4-28 8:00	LV	EURO	6	818	1	13	24	1.589	1.541
6653	2020-4-29 8:00	LV	EURO	18	836	0	13	25	1.555	1.637
6654	2020-4-30 8:00	LV	EURO	13	849	2	15	26	1.767	1.690
6655	2020-5-1 8:00	LV	EURO	9	858	0	15	27	1.748	1.785
6656	2020-5-2 8:00	LV	EURO	12	870	1	16	28	1.839	1.794
6657										
6658	2020-2-21 8:00	LB	EMRO	1	1	0	0			
6659	2020-2-22 8:00	LB	EMRO	0	1	0	0			
6660	2020-2-23 8:00	LB	EMRO	0	1	0	0			

Table S1 Daily confirmed cases and daily deaths of COVID-19 in 214 nations from WHO

ID	Date and time	Country or region	Continent	Confirmed patients	Cumulative Confirmed patients	Daily dead patients	Daily cumulative dead patients	Lag days	Daily CFR	3DMA CFR
6661	2020-2-24 8:00	LB	EMRO	0	1	0	0			
6662	2020-2-25 8:00	LB	EMRO	0	1	0	0			
6663	2020-2-26 8:00	LB	EMRO	1	2	0	0			
6664	2020-2-27 8:00	LB	EMRO	1	3	0	0			
6665	2020-2-28 8:00	LB	EMRO	1	4	0	0			
6666	2020-2-29 8:00	LB	EMRO	3	7	0	0			
6667	2020-3-1 8:00	LB	EMRO	3	10	0	0			
6668	2020-3-2 8:00	LB	EMRO	3	13	0	0			
6669	2020-3-3 8:00	LB	EMRO	0	13	0	0			
6670	2020-3-4 8:00	LB	EMRO	0	13	0	0			
6671	2020-3-5 8:00	LB	EMRO	3	16	0	0			
6672	2020-3-6 8:00	LB	EMRO	6	22	0	0			
6673	2020-3-7 8:00	LB	EMRO	0	22	0	0			
6674	2020-3-8 8:00	LB	EMRO	10	32	0	0			
6675	2020-3-9 8:00	LB	EMRO	0	32	0	0			
6676	2020-3-10 8:00	LB	EMRO	0	32	0	0			
6677	2020-3-11 8:00	LB	EMRO	29	61	1	1	0	1.639	3.092
6678	2020-3-12 8:00	LB	EMRO	5	66	2	3	1	4.545	3.577
6679	2020-3-13 8:00	LB	EMRO	0	66	0	3	2	4.545	4.106
6680	2020-3-14 8:00	LB	EMRO	27	93	0	3	3	3.226	3.601
6681	2020-3-15 8:00	LB	EMRO	6	99	0	3	4	3.030	3.095
6682	2020-3-16 8:00	LB	EMRO	0	99	0	3	5	3.030	2.854
6683	2020-3-17 8:00	LB	EMRO	21	120	0	3	6	2.500	2.955
6684	2020-3-18 8:00	LB	EMRO	0	120	1	4	7	3.333	2.839
6685	2020-3-19 8:00	LB	EMRO	29	149	0	4	8	2.685	2.824
6686	2020-3-20 8:00	LB	EMRO	14	163	0	4	9	2.454	2.360
6687	2020-3-21 8:00	LB	EMRO	43	206	0	4	10	1.942	2.003
6688	2020-3-22 8:00	LB	EMRO	42	248	0	4	11	1.613	1.684
6689	2020-3-23 8:00	LB	EMRO	19	267	0	4	12	1.498	1.476
6690	2020-3-24 8:00	LB	EMRO	37	304	0	4	13	1.316	1.338
6691	2020-3-25 8:00	LB	EMRO	29	333	0	4	14	1.201	1.382
6692	2020-3-26 8:00	LB	EMRO	35	368	2	6	15	1.630	1.541
6693	2020-3-27 8:00	LB	EMRO	23	391	1	7	16	1.790	1.787
6694	2020-3-28 8:00	LB	EMRO	21	412	1	8	17	1.942	2.005
6695	2020-3-29 8:00	LB	EMRO	26	438	2	10	18	2.283	2.230
6696	2020-3-30 8:00	LB	EMRO	8	446	1	11	19	2.466	2.447

Table S1 Daily confirmed cases and daily deaths of COVID-19 in 214 nations from WHO

ID	Date and time	Country or region	Continent	Confirmed patients	Cumulative Confirmed patients	Daily dead patients	Daily cumulative dead patients	Lag days	Daily CFR	3DMA CFR
6697	2020-3-31 8:00	LB	EMRO	17	463	1	12	20	2.592	2.521
6698	2020-4-1 8:00	LB	EMRO	16	479	0	12	21	2.505	2.779
6699	2020-4-2 8:00	LB	EMRO	15	494	4	16	22	3.239	3.030
6700	2020-4-3 8:00	LB	EMRO	14	508	1	17	23	3.346	3.285
6701	2020-4-4 8:00	LB	EMRO	12	520	0	17	24	3.269	3.344
6702	2020-4-5 8:00	LB	EMRO	7	527	1	18	25	3.416	3.399
6703	2020-4-6 8:00	LB	EMRO	14	541	1	19	26	3.512	3.465
6704	2020-4-7 8:00	LB	EMRO	7	548	0	19	27	3.467	3.428
6705	2020-4-8 8:00	LB	EMRO	27	575	0	19	28	3.304	3.345
6706	2020-4-9 8:00	LB	EMRO	7	582	0	19	29	3.265	3.284
6707	2020-4-10 8:00	LB	EMRO	27	609	1	20	30	3.284	3.260
6708	2020-4-11 8:00	LB	EMRO	10	619	0	20	31	3.231	3.230
6709	2020-4-12 8:00	LB	EMRO	11	630	0	20	32	3.175	3.190
6710	2020-4-13 8:00	LB	EMRO	2	632	0	20	33	3.165	3.205
6711	2020-4-14 8:00	LB	EMRO	9	641	1	21	34	3.276	3.211
6712	2020-4-15 8:00	LB	EMRO	17	658	0	21	35	3.191	3.212
6713	2020-4-16 8:00	LB	EMRO	5	663	0	21	36	3.167	3.168
6714	2020-4-17 8:00	LB	EMRO	5	668	0	21	37	3.144	3.145
6715	2020-4-18 8:00	LB	EMRO	4	672	0	21	38	3.125	3.130
6716	2020-4-19 8:00	LB	EMRO	1	673	0	21	39	3.120	3.116
6717	2020-4-20 8:00	LB	EMRO	4	677	0	21	40	3.102	3.108
6718	2020-4-21 8:00	LB	EMRO	0	677	0	21	41	3.102	3.143
6719	2020-4-22 8:00	LB	EMRO	5	682	1	22	42	3.226	3.175
6720	2020-4-23 8:00	LB	EMRO	6	688	0	22	43	3.198	3.195
6721	2020-4-24 8:00	LB	EMRO	8	696	0	22	44	3.161	3.256
6722	2020-4-25 8:00	LB	EMRO	8	704	2	24	45	3.409	3.322
6723	2020-4-26 8:00	LB	EMRO	3	707	0	24	46	3.395	3.395
6724	2020-4-27 8:00	LB	EMRO	3	710	0	24	47	3.380	3.374
6725	2020-4-28 8:00	LB	EMRO	7	717	0	24	48	3.347	3.352
6726	2020-4-29 8:00	LB	EMRO	4	721	0	24	49	3.329	3.329
6727	2020-4-30 8:00	LB	EMRO	4	725	0	24	50	3.310	3.310
6728	2020-5-1 8:00	LB	EMRO	4	729	0	24	51	3.292	3.298
6729	2020-5-2 8:00	LB	EMRO	0	729	0	24	52	3.292	3.292
6730										
6731	2020-3-17 8:00	LR	AFRO	1	1	0	0			
6732	2020-3-18 8:00	LR	AFRO	1	2	0	0			

Table S1 Daily confirmed cases and daily deaths of COVID-19 in 214 nations from WHO

ID	Date and time	Country or region	Continent	Confirmed patients	Cumulative Confirmed patients	Daily dead patients	Daily cumulative dead patients	Lag days	Daily CFR	3DMA CFR
6733	2020-3-19 8:00	LR	AFRO	0	2	0	0			
6734	2020-3-20 8:00	LR	AFRO	0	2	0	0			
6735	2020-3-21 8:00	LR	AFRO	1	3	0	0			
6736	2020-3-22 8:00	LR	AFRO	0	3	0	0			
6737	2020-3-23 8:00	LR	AFRO	0	3	0	0			
6738	2020-3-24 8:00	LR	AFRO	0	3	0	0			
6739	2020-3-25 8:00	LR	AFRO	0	3	0	0			
6740	2020-3-26 8:00	LR	AFRO	0	3	0	0			
6741	2020-3-27 8:00	LR	AFRO	0	3	0	0			
6742	2020-3-28 8:00	LR	AFRO	0	3	0	0			
6743	2020-3-29 8:00	LR	AFRO	0	3	0	0			
6744	2020-3-30 8:00	LR	AFRO	0	3	0	0			
6745	2020-3-31 8:00	LR	AFRO	0	3	0	0			
6746	2020-4-1 8:00	LR	AFRO	3	6	0	0			
6747	2020-4-2 8:00	LR	AFRO	0	6	0	0			
6748	2020-4-3 8:00	LR	AFRO	0	6	0	0			
6749	2020-4-4 8:00	LR	AFRO	1	7	0	0			
6750	2020-4-5 8:00	LR	AFRO	3	10	1	1	0	10.000	16.538
6751	2020-4-6 8:00	LR	AFRO	3	13	2	3	1	23.077	18.168
6752	2020-4-7 8:00	LR	AFRO	1	14	0	3	2	21.429	21.978
6753	2020-4-8 8:00	LR	AFRO	0	14	0	3	3	21.429	18.587
6754	2020-4-9 8:00	LR	AFRO	17	31	1	4	4	12.903	15.745
6755	2020-4-10 8:00	LR	AFRO	0	31	0	4	5	12.903	13.107
6756	2020-4-11 8:00	LR	AFRO	6	37	1	5	6	13.514	12.278
6757	2020-4-12 8:00	LR	AFRO	11	48	0	5	7	10.417	11.310
6758	2020-4-13 8:00	LR	AFRO	2	50	0	5	8	10.000	10.727
6759	2020-4-14 8:00	LR	AFRO	1	51	1	6	9	11.765	10.645
6760	2020-4-15 8:00	LR	AFRO	8	59	0	6	10	10.169	10.701
6761	2020-4-16 8:00	LR	AFRO	0	59	0	6	11	10.169	9.519
6762	2020-4-17 8:00	LR	AFRO	14	73	0	6	12	8.219	9.200
6763	2020-4-18 8:00	LR	AFRO	3	76	1	7	13	9.211	8.691
6764	2020-4-19 8:00	LR	AFRO	5	81	0	7	14	8.642	8.881
6765	2020-4-20 8:00	LR	AFRO	10	91	1	8	15	8.791	8.505
6766	2020-4-21 8:00	LR	AFRO	8	99	0	8	16	8.081	8.264
6767	2020-4-22 8:00	LR	AFRO	2	101	0	8	17	7.921	7.974
6768	2020-4-23 8:00	LR	AFRO	0	101	0	8	18	7.921	7.921

Table S1 Daily confirmed cases and daily deaths of COVID-19 in 214 nations from WHO

ID	Date and time	Country or region	Continent	Confirmed patients	Cumulative Confirmed patients	Daily dead patients	Daily cumulative dead patients	Lag days	Daily CFR	3DMA CFR
6769	2020-4-24 8:00	LR	AFRO	0	101	0	8	19	7.921	7.560
6770	2020-4-25 8:00	LR	AFRO	16	117	0	8	20	6.838	7.975
6771	2020-4-26 8:00	LR	AFRO	3	120	3	11	21	9.167	8.561
6772	2020-4-27 8:00	LR	AFRO	4	124	1	12	22	9.677	10.291
6773	2020-4-28 8:00	LR	AFRO	9	133	4	16	23	12.030	11.018
6774	2020-4-29 8:00	LR	AFRO	8	141	0	16	24	11.348	11.575
6775	2020-4-30 8:00	LR	AFRO	0	141	0	16	25	11.348	11.348
6776	2020-5-1 8:00	LR	AFRO	0	141	0	16	26	11.348	11.512
6777	2020-5-2 8:00	LR	AFRO	11	152	2	18	27	11.842	11.595
6778										
6779	2020-3-25 8:00	LY	EMRO	1	1	0	0			
6780	2020-3-26 8:00	LY	EMRO	0	1	0	0			
6781	2020-3-27 8:00	LY	EMRO	0	1	0	0			
6782	2020-3-28 8:00	LY	EMRO	0	1	0	0			
6783	2020-3-29 8:00	LY	EMRO	2	3	0	0			
6784	2020-3-30 8:00	LY	EMRO	5	8	0	0			
6785	2020-3-31 8:00	LY	EMRO	0	8	0	0			
6786	2020-4-1 8:00	LY	EMRO	2	10	0	0			
6787	2020-4-2 8:00	LY	EMRO	0	10	0	0			
6788	2020-4-3 8:00	LY	EMRO	1	11	1	1	0	9.091	7.487
6789	2020-4-4 8:00	LY	EMRO	6	17	0	1	1	5.882	6.843
6790	2020-4-5 8:00	LY	EMRO	1	18	0	1	2	5.556	5.664
6791	2020-4-6 8:00	LY	EMRO	0	18	0	1	3	5.556	5.458
6792	2020-4-7 8:00	LY	EMRO	1	19	0	1	4	5.263	5.194
6793	2020-4-8 8:00	LY	EMRO	2	21	0	1	5	4.762	4.929
6794	2020-4-9 8:00	LY	EMRO	0	21	0	1	6	4.762	4.563
6795	2020-4-10 8:00	LY	EMRO	3	24	0	1	7	4.167	4.365
6796	2020-4-11 8:00	LY	EMRO	0	24	0	1	8	4.167	4.111
6797	2020-4-12 8:00	LY	EMRO	1	25	0	1	9	4.000	4.056
6798	2020-4-13 8:00	LY	EMRO	0	25	0	1	10	4.000	3.949
6799	2020-4-14 8:00	LY	EMRO	1	26	0	1	11	3.846	3.568
6800	2020-4-15 8:00	LY	EMRO	9	35	0	1	12	2.857	2.929
6801	2020-4-16 8:00	LY	EMRO	13	48	0	1	13	2.083	2.327
6802	2020-4-17 8:00	LY	EMRO	1	49	0	1	14	2.041	2.055
6803	2020-4-18 8:00	LY	EMRO	0	49	0	1	15	2.041	2.041
6804	2020-4-19 8:00	LY	EMRO	0	49	0	1	16	2.041	2.014

Table S1 Daily confirmed cases and daily deaths of COVID-19 in 214 nations from WHO

ID	Date and time	Country or region	Continent	Confirmed patients	Cumulative Confirmed patients	Daily dead patients	Daily cumulative dead patients	Lag days	Daily CFR	3DMA CFR
6805	2020-4-20 8:00	LY	EMRO	2	51	0	1	17	1.961	1.987
6806	2020-4-21 8:00	LY	EMRO	0	51	0	1	18	1.961	1.872
6807	2020-4-22 8:00	LY	EMRO	8	59	0	1	19	1.695	1.774
6808	2020-4-23 8:00	LY	EMRO	1	60	0	1	20	1.667	2.232
6809	2020-4-24 8:00	LY	EMRO	0	60	1	2	21	3.333	2.760
6810	2020-4-25 8:00	LY	EMRO	1	61	0	2	22	3.279	3.297
6811	2020-4-26 8:00	LY	EMRO	0	61	0	2	23	3.279	3.279
6812	2020-4-27 8:00	LY	EMRO	0	61	0	2	24	3.279	3.279
6813	2020-4-28 8:00	LY	EMRO	0	61	0	2	25	3.279	3.279
6814	2020-4-29 8:00	LY	EMRO	0	61	0	2	26	3.279	3.279
6815	2020-4-30 8:00	LY	EMRO	0	61	0	2	27	3.279	3.773
6816	2020-5-1 8:00	LY	EMRO	2	63	1	3	28	4.762	4.267
6817	2020-5-2 8:00	LY	EMRO	0	63	0	3	29	4.762	4.762
6818										
6819	2020-3-5 8:00	LI	EURO	1	1	0	0			
6820	2020-3-6 8:00	LI	EURO	0	1	0	0			
6821	2020-3-7 8:00	LI	EURO	0	1	0	0			
6822	2020-3-8 8:00	LI	EURO	0	1	0	0			
6823	2020-3-9 8:00	LI	EURO	0	1	0	0			
6824	2020-3-10 8:00	LI	EURO	0	1	0	0			
6825	2020-3-11 8:00	LI	EURO	0	1	0	0			
6826	2020-3-12 8:00	LI	EURO	3	4	0	0			
6827	2020-3-13 8:00	LI	EURO	0	4	0	0			
6828	2020-3-14 8:00	LI	EURO	0	4	0	0			
6829	2020-3-15 8:00	LI	EURO	0	4	0	0			
6830	2020-3-16 8:00	LI	EURO	3	7	0	0			
6831	2020-3-17 8:00	LI	EURO	0	7	0	0			
6832	2020-3-18 8:00	LI	EURO	18	25	0	0			
6833	2020-3-19 8:00	LI	EURO	0	25	0	0			
6834	2020-3-20 8:00	LI	EURO	9	34	0	0			
6835	2020-3-21 8:00	LI	EURO	2	36	0	0			
6836	2020-3-22 8:00	LI	EURO	0	36	0	0			
6837	2020-3-23 8:00	LI	EURO	10	46	0	0			
6838	2020-3-24 8:00	LI	EURO	0	46	0	0			
6839	2020-3-25 8:00	LI	EURO	1	47	0	0			
6840	2020-3-26 8:00	LI	EURO	4	51	0	0			

Table S1 Daily confirmed cases and daily deaths of COVID-19 in 214 nations from WHO

ID	Date and time	Country or region	Continent	Confirmed patients	Cumulative Confirmed patients	Daily dead patients	Daily cumulative dead patients	Lag days	Daily CFR	3DMA CFR
6841	2020-3-27 8:00	LI	EURO	5	56	0	0			
6842	2020-3-28 8:00	LI	EURO	4	60	0	0			
6843	2020-3-29 8:00	LI	EURO	1	61	0	0			
6844	2020-3-30 8:00	LI	EURO	1	62	0	0			
6845	2020-3-31 8:00	LI	EURO	2	64	0	0			
6846	2020-4-1 8:00	LI	EURO	4	68	0	0			
6847	2020-4-2 8:00	LI	EURO	4	72	0	0			
6848	2020-4-3 8:00	LI	EURO	3	75	0	0			
6849	2020-4-4 8:00	LI	EURO	1	76	0	0			
6850	2020-4-5 8:00	LI	EURO	1	77	1	1	0	1.299	1.290
6851	2020-4-6 8:00	LI	EURO	1	78	0	1	1	1.282	1.288
6852	2020-4-7 8:00	LI	EURO	0	78	0	1	2	1.282	1.282
6853	2020-4-8 8:00	LI	EURO	0	78	0	1	3	1.282	1.277
6854	2020-4-9 8:00	LI	EURO	1	79	0	1	4	1.266	1.271
6855	2020-4-10 8:00	LI	EURO	0	79	0	1	5	1.266	1.261
6856	2020-4-11 8:00	LI	EURO	1	80	0	1	6	1.250	1.255
6857	2020-4-12 8:00	LI	EURO	0	80	0	1	7	1.250	1.250
6858	2020-4-13 8:00	LI	EURO	0	80	0	1	8	1.250	1.250
6859	2020-4-14 8:00	LI	EURO	0	80	0	1	9	1.250	1.245
6860	2020-4-15 8:00	LI	EURO	1	81	0	1	10	1.235	1.240
6861	2020-4-16 8:00	LI	EURO	0	81	0	1	11	1.235	1.235
6862	2020-4-17 8:00	LI	EURO	0	81	0	1	12	1.235	1.235
6863	2020-4-18 8:00	LI	EURO	0	81	0	1	13	1.235	1.230
6864	2020-4-19 8:00	LI	EURO	1	82	0	1	14	1.220	1.225
6865	2020-4-20 8:00	LI	EURO	0	82	0	1	15	1.220	1.220
6866	2020-4-21 8:00	LI	EURO	0	82	0	1	16	1.220	1.220
6867	2020-4-22 8:00	LI	EURO	0	82	0	1	17	1.220	1.220
6868	2020-4-23 8:00	LI	EURO	0	82	0	1	18	1.220	1.220
6869	2020-4-24 8:00	LI	EURO	0	82	0	1	19	1.220	1.220
6870	2020-4-25 8:00	LI	EURO	0	82	0	1	20	1.220	1.215
6871	2020-4-26 8:00	LI	EURO	1	83	0	1	21	1.205	1.210
6872	2020-4-27 8:00	LI	EURO	0	83	0	1	22	1.205	1.205
6873	2020-4-28 8:00	LI	EURO	0	83	0	1	23	1.205	1.205
6874	2020-4-29 8:00	LI	EURO	0	83	0	1	24	1.205	1.205
6875	2020-4-30 8:00	LI	EURO	0	83	0	1	25	1.205	1.205
6876	2020-5-1 8:00	LI	EURO	0	83	0	1	26	1.205	1.205

Table S1 Daily confirmed cases and daily deaths of COVID-19 in 214 nations from WHO

ID	Date and time	Country or region	Continent	Confirmed patients	Cumulative Confirmed patients	Daily dead patients	Daily cumulative dead patients	Lag days	Daily CFR	3DMA CFR
6877	2020-5-2 8:00	LI	EURO	0	83	0	1	27	1.205	1.205
6878										
6879	2020-2-28 8:00	LT	EURO	1	1	0	0			
6880	2020-2-29 8:00	LT	EURO	0	1	0	0			
6881	2020-3-1 8:00	LT	EURO	0	1	0	0			
6882	2020-3-2 8:00	LT	EURO	0	1	0	0			
6883	2020-3-3 8:00	LT	EURO	0	1	0	0			
6884	2020-3-4 8:00	LT	EURO	0	1	0	0			
6885	2020-3-5 8:00	LT	EURO	0	1	0	0			
6886	2020-3-6 8:00	LT	EURO	0	1	0	0			
6887	2020-3-7 8:00	LT	EURO	0	1	0	0			
6888	2020-3-8 8:00	LT	EURO	0	1	0	0			
6889	2020-3-9 8:00	LT	EURO	0	1	0	0			
6890	2020-3-10 8:00	LT	EURO	0	1	0	0			
6891	2020-3-11 8:00	LT	EURO	2	3	0	0			
6892	2020-3-12 8:00	LT	EURO	0	3	0	0			
6893	2020-3-13 8:00	LT	EURO	3	6	0	0			
6894	2020-3-14 8:00	LT	EURO	2	8	0	0			
6895	2020-3-15 8:00	LT	EURO	1	9	0	0			
6896	2020-3-16 8:00	LT	EURO	8	17	0	0			
6897	2020-3-17 8:00	LT	EURO	8	25	0	0			
6898	2020-3-18 8:00	LT	EURO	1	26	0	0			
6899	2020-3-19 8:00	LT	EURO	10	36	0	0			
6900	2020-3-20 8:00	LT	EURO	33	69	1	1	0	1.449	1.201
6901	2020-3-21 8:00	LT	EURO	36	105	0	1	1	0.952	1.118
6902	2020-3-22 8:00	LT	EURO	0	105	0	1	2	0.952	0.868
6903	2020-3-23 8:00	LT	EURO	38	143	0	1	3	0.699	0.737
6904	2020-3-24 8:00	LT	EURO	36	179	0	1	4	0.559	0.738
6905	2020-3-25 8:00	LT	EURO	30	209	1	2	5	0.957	0.992
6906	2020-3-26 8:00	LT	EURO	65	274	2	4	6	1.460	1.252
6907	2020-3-27 8:00	LT	EURO	25	299	0	4	7	1.338	1.398
6908	2020-3-28 8:00	LT	EURO	59	358	1	5	8	1.397	1.504
6909	2020-3-29 8:00	LT	EURO	36	394	2	7	9	1.777	1.540
6910	2020-3-30 8:00	LT	EURO	90	484	0	7	10	1.446	1.556
6911	2020-3-31 8:00	LT	EURO	0	484	0	7	11	1.446	1.402
6912	2020-4-1 8:00	LT	EURO	49	533	0	7	12	1.313	1.379

Table S1 Daily confirmed cases and daily deaths of COVID-19 in 214 nations from WHO

ID	Date and time	Country or region	Continent	Confirmed patients	Cumulative Confirmed patients	Daily dead patients	Daily cumulative dead patients	Lag days	Daily CFR	3DMA CFR
6913	2020-4-2 8:00	LT	EURO	48	581	1	8	13	1.377	1.359
6914	2020-4-3 8:00	LT	EURO	68	649	1	9	14	1.387	1.310
6915	2020-4-4 8:00	LT	EURO	122	771	0	9	15	1.167	1.240
6916	2020-4-5 8:00	LT	EURO	0	771	0	9	16	1.167	1.313
6917	2020-4-6 8:00	LT	EURO	40	811	4	13	17	1.603	1.477
6918	2020-4-7 8:00	LT	EURO	32	843	1	14	18	1.661	1.656
6919	2020-4-8 8:00	LT	EURO	37	880	1	15	19	1.705	1.670
6920	2020-4-9 8:00	LT	EURO	32	912	0	15	20	1.645	1.640
6921	2020-4-10 8:00	LT	EURO	43	955	0	15	21	1.571	1.639
6922	2020-4-11 8:00	LT	EURO	44	999	2	17	22	1.702	1.819
6923	2020-4-12 8:00	LT	EURO	54	1053	6	23	23	2.184	2.049
6924	2020-4-13 8:00	LT	EURO	9	1062	1	24	24	2.260	2.229
6925	2020-4-14 8:00	LT	EURO	8	1070	0	24	25	2.243	2.249
6926	2020-4-15 8:00	LT	EURO	0	1070	0	24	26	2.243	2.381
6927	2020-4-16 8:00	LT	EURO	21	1091	5	29	27	2.658	2.562
6928	2020-4-17 8:00	LT	EURO	58	1149	3	32	28	2.785	2.702
6929	2020-4-18 8:00	LT	EURO	90	1239	1	33	29	2.663	2.664
6930	2020-4-19 8:00	LT	EURO	59	1298	0	33	30	2.542	2.640
6931	2020-4-20 8:00	LT	EURO	28	1326	3	36	31	2.715	2.666
6932	2020-4-21 8:00	LT	EURO	24	1350	1	37	32	2.741	2.743
6933	2020-4-22 8:00	LT	EURO	20	1370	1	38	33	2.774	2.744
6934	2020-4-23 8:00	LT	EURO	28	1398	0	38	34	2.718	2.776
6935	2020-4-24 8:00	LT	EURO	12	1410	2	40	35	2.837	2.797
6936	2020-4-25 8:00	LT	EURO	0	1410	0	40	36	2.837	2.842
6937	2020-4-26 8:00	LT	EURO	28	1438	1	41	37	2.851	2.839
6938	2020-4-27 8:00	LT	EURO	11	1449	0	41	38	2.830	2.837
6939	2020-4-28 8:00	LT	EURO	0	1449	0	41	39	2.830	2.899
6940	2020-4-29 8:00	LT	EURO	0	1449	3	44	40	3.037	3.046
6941	2020-4-30 8:00	LT	EURO	-74	1375	1	45	41	3.273	3.186
6942	2020-5-1 8:00	LT	EURO	10	1385	0	45	42	3.249	3.246
6943	2020-5-2 8:00	LT	EURO	14	1399	0	45	43	3.217	3.233
6944										
6945	2020-3-1 8:00	LU	EURO	1	1	0	0			
6946	2020-3-2 8:00	LU	EURO	0	1	0	0			
6947	2020-3-3 8:00	LU	EURO	0	1	0	0			
6948	2020-3-4 8:00	LU	EURO	0	1	0	0			

Table S1 Daily confirmed cases and daily deaths of COVID-19 in 214 nations from WHO

ID	Date and time	Country or region	Continent	Confirmed patients	Cumulative Confirmed patients	Daily dead patients	Daily cumulative dead patients	Lag days	Daily CFR	3DMA CFR
6949	2020-3-5 8:00	LU	EURO	0	1	0	0			
6950	2020-3-6 8:00	LU	EURO	1	2	0	0			
6951	2020-3-7 8:00	LU	EURO	0	2	0	0			
6952	2020-3-8 8:00	LU	EURO	0	2	0	0			
6953	2020-3-9 8:00	LU	EURO	3	5	0	0			
6954	2020-3-10 8:00	LU	EURO	0	5	0	0			
6955	2020-3-11 8:00	LU	EURO	0	5	0	0			
6956	2020-3-12 8:00	LU	EURO	12	17	0	0			
6957	2020-3-13 8:00	LU	EURO	21	38	1	1	0	2.632	2.632
6958	2020-3-14 8:00	LU	EURO	0	38	0	1	1	2.632	2.632
6959	2020-3-15 8:00	LU	EURO	0	38	0	1	2	2.632	2.166
6960	2020-3-16 8:00	LU	EURO	43	81	0	1	3	1.235	1.527
6961	2020-3-17 8:00	LU	EURO	59	140	0	1	4	0.714	0.967
6962	2020-3-18 8:00	LU	EURO	70	210	1	2	5	0.952	0.942
6963	2020-3-19 8:00	LU	EURO	135	345	2	4	6	1.159	1.048
6964	2020-3-20 8:00	LU	EURO	139	484	1	5	7	1.033	1.129
6965	2020-3-21 8:00	LU	EURO	186	670	3	8	8	1.194	1.140
6966	2020-3-22 8:00	LU	EURO	0	670	0	8	9	1.194	1.130
6967	2020-3-23 8:00	LU	EURO	128	798	0	8	10	1.003	1.037
6968	2020-3-24 8:00	LU	EURO	77	875	0	8	11	0.914	0.882
6969	2020-3-25 8:00	LU	EURO	224	1099	0	8	12	0.728	0.747
6970	2020-3-26 8:00	LU	EURO	234	1333	0	8	13	0.600	0.649
6971	2020-3-27 8:00	LU	EURO	120	1453	1	9	14	0.619	0.718
6972	2020-3-28 8:00	LU	EURO	152	1605	6	15	15	0.935	0.846
6973	2020-3-29 8:00	LU	EURO	226	1831	3	18	16	0.983	0.998
6974	2020-3-30 8:00	LU	EURO	119	1950	3	21	17	1.077	1.056
6975	2020-3-31 8:00	LU	EURO	38	1988	1	22	18	1.107	1.080
6976	2020-4-1 8:00	LU	EURO	190	2178	1	23	19	1.056	1.138
6977	2020-4-2 8:00	LU	EURO	141	2319	6	29	20	1.251	1.171
6978	2020-4-3 8:00	LU	EURO	168	2487	1	30	21	1.206	1.215
6979	2020-4-4 8:00	LU	EURO	125	2612	1	31	22	1.187	1.176
6980	2020-4-5 8:00	LU	EURO	117	2729	0	31	23	1.136	1.202
6981	2020-4-6 8:00	LU	EURO	75	2804	5	36	24	1.284	1.287
6982	2020-4-7 8:00	LU	EURO	39	2843	5	41	25	1.442	1.403
6983	2020-4-8 8:00	LU	EURO	127	2970	3	44	26	1.481	1.480
6984	2020-4-9 8:00	LU	EURO	64	3034	2	46	27	1.516	1.556

Table S1 Daily confirmed cases and daily deaths of COVID-19 in 214 nations from WHO

ID	Date and time	Country or region	Continent	Confirmed patients	Cumulative Confirmed patients	Daily dead patients	Daily cumulative dead patients	Lag days	Daily CFR	3DMA CFR
6985	2020-4-10 8:00	LU	EURO	81	3115	6	52	28	1.669	1.620
6986	2020-4-11 8:00	LU	EURO	108	3223	2	54	29	1.675	1.747
6987	2020-4-12 8:00	LU	EURO	47	3270	8	62	30	1.896	1.861
6988	2020-4-13 8:00	LU	EURO	11	3281	4	66	31	2.012	2.001
6989	2020-4-14 8:00	LU	EURO	11	3292	3	69	32	2.096	2.065
6990	2020-4-15 8:00	LU	EURO	15	3307	0	69	33	2.086	2.076
6991	2020-4-16 8:00	LU	EURO	66	3373	0	69	34	2.046	2.045
6992	2020-4-17 8:00	LU	EURO	71	3444	0	69	35	2.003	2.039
6993	2020-4-18 8:00	LU	EURO	36	3480	3	72	36	2.069	2.036
6994	2020-4-19 8:00	LU	EURO	57	3537	0	72	37	2.036	2.054
6995	2020-4-20 8:00	LU	EURO	13	3550	1	73	38	2.056	2.067
6996	2020-4-21 8:00	LU	EURO	8	3558	2	75	39	2.108	2.107
6997	2020-4-22 8:00	LU	EURO	60	3618	3	78	40	2.156	2.151
6998	2020-4-23 8:00	LU	EURO	36	3654	2	80	41	2.189	2.203
6999	2020-4-24 8:00	LU	EURO	11	3665	3	83	42	2.265	2.251
7000	2020-4-25 8:00	LU	EURO	30	3695	2	85	43	2.300	2.285
7001	2020-4-26 8:00	LU	EURO	16	3711	0	85	44	2.290	2.318
7002	2020-4-27 8:00	LU	EURO	12	3723	3	88	45	2.364	2.338
7003	2020-4-28 8:00	LU	EURO	6	3729	0	88	46	2.360	2.368
7004	2020-4-29 8:00	LU	EURO	12	3741	1	89	47	2.379	2.367
7005	2020-4-30 8:00	LU	EURO	28	3769	0	89	48	2.361	2.373
7006	2020-5-1 8:00	LU	EURO	15	3784	1	90	49	2.378	2.387
7007	2020-5-2 8:00	LU	EURO	18	3802	2	92	50	2.420	2.399
7008										
7009	2020-3-21 8:00	MG	AFRO	3	3	0	0			
7010	2020-3-22 8:00	MG	AFRO	0	3	0	0			
7011	2020-3-23 8:00	MG	AFRO	9	12	0	0			
7012	2020-3-24 8:00	MG	AFRO	1	13	0	0			
7013	2020-3-25 8:00	MG	AFRO	6	19	0	0			
7014	2020-3-26 8:00	MG	AFRO	0	19	0	0			
7015	2020-3-27 8:00	MG	AFRO	5	24	0	0			
7016	2020-3-28 8:00	MG	AFRO	2	26	0	0			
7017	2020-3-29 8:00	MG	AFRO	11	37	0	0			
7018	2020-3-30 8:00	MG	AFRO	7	44	0	0			
7019	2020-3-31 8:00	MG	AFRO	2	46	0	0			
7020	2020-4-1 8:00	MG	AFRO	7	53	0	0			

Table S1 Daily confirmed cases and daily deaths of COVID-19 in 214 nations from WHO

ID	Date and time	Country or region	Continent	Confirmed patients	Cumulative Confirmed patients	Daily dead patients	Daily cumulative dead patients	Lag days	Daily CFR	3DMA CFR
7021	2020-4-2 8:00	MG	AFRO	6	59	0	0			
7022	2020-4-3 8:00	MG	AFRO	6	65	0	0			
7023	2020-4-4 8:00	MG	AFRO	5	70	0	0			
7024	2020-4-5 8:00	MG	AFRO	0	70	0	0			
7025	2020-4-6 8:00	MG	AFRO	7	77	0	0			
7026	2020-4-7 8:00	MG	AFRO	8	85	0	0			
7027	2020-4-8 8:00	MG	AFRO	7	92	0	0			
7028	2020-4-9 8:00	MG	AFRO	1	93	0	0			
7029	2020-4-10 8:00	MG	AFRO	2	95	0	0			
7030	2020-4-11 8:00	MG	AFRO	0	95	0	0			
7031	2020-4-12 8:00	MG	AFRO	9	104	0	0			
7032	2020-4-13 8:00	MG	AFRO	2	106	0	0			
7033	2020-4-14 8:00	MG	AFRO	0	106	0	0			
7034	2020-4-15 8:00	MG	AFRO	4	110	0	0			
7035	2020-4-16 8:00	MG	AFRO	0	110	0	0			
7036	2020-4-17 8:00	MG	AFRO	7	117	0	0			
7037	2020-4-18 8:00	MG	AFRO	0	117	0	0			
7038	2020-4-19 8:00	MG	AFRO	3	120	0	0			
7039	2020-4-20 8:00	MG	AFRO	1	121	0	0			
7040	2020-4-21 8:00	MG	AFRO	0	121	0	0			
7041	2020-4-22 8:00	MG	AFRO	0	121	0	0			
7042	2020-4-23 8:00	MG	AFRO	0	121	0	0			
7043	2020-4-24 8:00	MG	AFRO	0	121	0	0			
7044	2020-4-25 8:00	MG	AFRO	1	122	0	0			
7045	2020-4-26 8:00	MG	AFRO	2	124	0	0			
7046	2020-4-27 8:00	MG	AFRO	4	128	0	0			
7047	2020-4-28 8:00	MG	AFRO	0	128	0	0			
7048	2020-4-29 8:00	MG	AFRO	0	128	0	0			
7049	2020-4-30 8:00	MG	AFRO	0	128	0	0			
7050	2020-5-1 8:00	MG	AFRO	4	132	0	0			
7051	2020-5-2 8:00	MG	AFRO	3	135	0	0			
7052										
7053	2020-4-3 8:00	MW	AFRO	3	3	0	0			
7054	2020-4-4 8:00	MW	AFRO	0	3	0	0			
7055	2020-4-5 8:00	MW	AFRO	0	3	0	0			
7056	2020-4-6 8:00	MW	AFRO	1	4	0	0			

Table S1 Daily confirmed cases and daily deaths of COVID-19 in 214 nations from WHO

ID	Date and time	Country or region	Continent	Confirmed patients	Cumulative Confirmed patients	Daily dead patients	Daily cumulative dead patients	Lag days	Daily CFR	3DMA CFR
7057	2020-4-7 8:00	MW	AFRO	0	4	0	0			
7058	2020-4-8 8:00	MW	AFRO	4	8	1	1	0	12.500	12.500
7059	2020-4-9 8:00	MW	AFRO	0	8	0	1	1	12.500	12.500
7060	2020-4-10 8:00	MW	AFRO	0	8	0	1	2	12.500	12.037
7061	2020-4-11 8:00	MW	AFRO	1	9	0	1	3	11.111	13.426
7062	2020-4-12 8:00	MW	AFRO	3	12	1	2	4	16.667	14.387
7063	2020-4-13 8:00	MW	AFRO	1	13	0	2	5	15.385	14.850
7064	2020-4-14 8:00	MW	AFRO	3	16	0	2	6	12.500	13.462
7065	2020-4-15 8:00	MW	AFRO	0	16	0	2	7	12.500	12.500
7066	2020-4-16 8:00	MW	AFRO	0	16	0	2	8	12.500	12.500
7067	2020-4-17 8:00	MW	AFRO	0	16	0	2	9	12.500	12.255
7068	2020-4-18 8:00	MW	AFRO	1	17	0	2	10	11.765	12.010
7069	2020-4-19 8:00	MW	AFRO	0	17	0	2	11	11.765	11.765
7070	2020-4-20 8:00	MW	AFRO	0	17	0	2	12	11.765	11.765
7071	2020-4-21 8:00	MW	AFRO	0	17	0	2	13	11.765	11.547
7072	2020-4-22 8:00	MW	AFRO	1	18	0	2	14	11.111	11.973
7073	2020-4-23 8:00	MW	AFRO	5	23	1	3	15	13.043	11.082
7074	2020-4-24 8:00	MW	AFRO	10	33	0	3	16	9.091	10.408
7075	2020-4-25 8:00	MW	AFRO	0	33	0	3	17	9.091	9.091
7076	2020-4-26 8:00	MW	AFRO	0	33	0	3	18	9.091	9.002
7077	2020-4-27 8:00	MW	AFRO	1	34	0	3	19	8.824	8.749
7078	2020-4-28 8:00	MW	AFRO	2	36	0	3	20	8.333	8.497
7079	2020-4-29 8:00	MW	AFRO	0	36	0	3	21	8.333	8.333
7080	2020-4-30 8:00	MW	AFRO	0	36	0	3	22	8.333	8.258
7081	2020-5-1 8:00	MW	AFRO	1	37	0	3	23	8.108	8.183
7082	2020-5-2 8:00	MW	AFRO	0	37	0	3	24	8.108	8.108
7083										
7084	2020-1-25 8:00	MY	WPRO	3	3	0	0			
7085	2020-1-26 8:00	MY	WPRO	1	4	0	0			
7086	2020-1-27 8:00	MY	WPRO	0	4	0	0			
7087	2020-1-28 8:00	MY	WPRO	0	4	0	0			
7088	2020-1-29 8:00	MY	WPRO	3	7	0	0			
7089	2020-1-30 8:00	MY	WPRO	1	8	0	0			
7090	2020-1-31 8:00	MY	WPRO	0	8	0	0			
7091	2020-2-1 8:00	MY	WPRO	0	8	0	0			
7092	2020-2-2 8:00	MY	WPRO	0	8	0	0			

Table S1 Daily confirmed cases and daily deaths of COVID-19 in 214 nations from WHO

ID	Date and time	Country or region	Continent	Confirmed patients	Cumulative Confirmed patients	Daily dead patients	Daily cumulative dead patients	Lag days	Daily CFR	3DMA CFR
7093	2020-2-3 8:00	MY	WPRO	0	8	0	0			
7094	2020-2-4 8:00	MY	WPRO	2	10	0	0			
7095	2020-2-5 8:00	MY	WPRO	2	12	0	0			
7096	2020-2-6 8:00	MY	WPRO	2	14	0	0			
7097	2020-2-7 8:00	MY	WPRO	1	15	0	0			
7098	2020-2-8 8:00	MY	WPRO	1	16	0	0			
7099	2020-2-9 8:00	MY	WPRO	1	17	0	0			
7100	2020-2-10 8:00	MY	WPRO	1	18	0	0			
7101	2020-2-11 8:00	MY	WPRO	0	18	0	0			
7102	2020-2-12 8:00	MY	WPRO	0	18	0	0			
7103	2020-2-13 8:00	MY	WPRO	1	19	0	0			
7104	2020-2-14 8:00	MY	WPRO	0	19	0	0			
7105	2020-2-15 8:00	MY	WPRO	3	22	0	0			
7106	2020-2-16 8:00	MY	WPRO	0	22	0	0			
7107	2020-2-17 8:00	MY	WPRO	0	22	0	0			
7108	2020-2-18 8:00	MY	WPRO	0	22	0	0			
7109	2020-2-19 8:00	MY	WPRO	0	22	0	0			
7110	2020-2-20 8:00	MY	WPRO	0	22	0	0			
7111	2020-2-21 8:00	MY	WPRO	0	22	0	0			
7112	2020-2-22 8:00	MY	WPRO	0	22	0	0			
7113	2020-2-23 8:00	MY	WPRO	0	22	0	0			
7114	2020-2-24 8:00	MY	WPRO	0	22	0	0			
7115	2020-2-25 8:00	MY	WPRO	0	22	0	0			
7116	2020-2-26 8:00	MY	WPRO	0	22	0	0			
7117	2020-2-27 8:00	MY	WPRO	2	24	0	0			
7118	2020-2-28 8:00	MY	WPRO	0	24	0	0			
7119	2020-2-29 8:00	MY	WPRO	0	24	0	0			
7120	2020-3-1 8:00	MY	WPRO	0	24	0	0			
7121	2020-3-2 8:00	MY	WPRO	5	29	0	0			
7122	2020-3-3 8:00	MY	WPRO	7	36	0	0			
7123	2020-3-4 8:00	MY	WPRO	0	36	0	0			
7124	2020-3-5 8:00	MY	WPRO	19	55	0	0			
7125	2020-3-6 8:00	MY	WPRO	28	83	0	0			
7126	2020-3-7 8:00	MY	WPRO	10	93	0	0			
7127	2020-3-8 8:00	MY	WPRO	0	93	0	0			
7128	2020-3-9 8:00	MY	WPRO	24	117	0	0			

Table S1 Daily confirmed cases and daily deaths of COVID-19 in 214 nations from WHO

ID	Date and time	Country or region	Continent	Confirmed patients	Cumulative Confirmed patients	Daily dead patients	Daily cumulative dead patients	Lag days	Daily CFR	3DMA CFR
7129	2020-3-10 8:00	MY	WPRO	0	117	0	0			
7130	2020-3-11 8:00	MY	WPRO	12	129	0	0			
7131	2020-3-12 8:00	MY	WPRO	0	129	0	0			
7132	2020-3-13 8:00	MY	WPRO	0	129	0	0			
7133	2020-3-14 8:00	MY	WPRO	68	197	0	0			
7134	2020-3-15 8:00	MY	WPRO	41	238	0	0			
7135	2020-3-16 8:00	MY	WPRO	315	553	0	0			
7136	2020-3-17 8:00	MY	WPRO	0	553	0	0			
7137	2020-3-18 8:00	MY	WPRO	120	673	2	2	0	0.297	0.260
7138	2020-3-19 8:00	MY	WPRO	227	900	0	2	1	0.222	0.270
7139	2020-3-20 8:00	MY	WPRO	130	1030	1	3	2	0.291	0.256
7140	2020-3-21 8:00	MY	WPRO	153	1183	0	3	3	0.254	0.435
7141	2020-3-22 8:00	MY	WPRO	0	1183	6	9	4	0.761	0.619
7142	2020-3-23 8:00	MY	WPRO	123	1306	2	11	5	0.842	0.864
7143	2020-3-24 8:00	MY	WPRO	212	1518	4	15	6	0.988	0.907
7144	2020-3-25 8:00	MY	WPRO	278	1796	1	16	7	0.891	1.016
7145	2020-3-26 8:00	MY	WPRO	0	1796	5	21	8	1.169	1.064
7146	2020-3-27 8:00	MY	WPRO	235	2031	2	23	9	1.132	1.168
7147	2020-3-28 8:00	MY	WPRO	130	2161	3	26	10	1.203	1.166
7148	2020-3-29 8:00	MY	WPRO	159	2320	1	27	11	1.164	1.248
7149	2020-3-30 8:00	MY	WPRO	150	2470	7	34	12	1.377	1.316
7150	2020-3-31 8:00	MY	WPRO	156	2626	3	37	13	1.409	1.447
7151	2020-4-1 8:00	MY	WPRO	140	2766	6	43	14	1.555	1.504
7152	2020-4-2 8:00	MY	WPRO	142	2908	2	45	15	1.547	1.569
7153	2020-4-3 8:00	MY	WPRO	208	3116	5	50	16	1.605	1.581
7154	2020-4-4 8:00	MY	WPRO	217	3333	3	53	17	1.590	1.610
7155	2020-4-5 8:00	MY	WPRO	150	3483	4	57	18	1.637	1.631
7156	2020-4-6 8:00	MY	WPRO	179	3662	4	61	19	1.666	1.646
7157	2020-4-7 8:00	MY	WPRO	131	3793	1	62	20	1.635	1.630
7158	2020-4-8 8:00	MY	WPRO	170	3963	1	63	21	1.590	1.601
7159	2020-4-9 8:00	MY	WPRO	156	4119	2	65	22	1.578	1.584
7160	2020-4-10 8:00	MY	WPRO	109	4228	2	67	23	1.585	1.591
7161	2020-4-11 8:00	MY	WPRO	118	4346	3	70	24	1.611	1.602
7162	2020-4-12 8:00	MY	WPRO	184	4530	3	73	25	1.611	1.615
7163	2020-4-13 8:00	MY	WPRO	153	4683	3	76	26	1.623	1.611
7164	2020-4-14 8:00	MY	WPRO	134	4817	1	77	27	1.599	1.622

Table S1 Daily confirmed cases and daily deaths of COVID-19 in 214 nations from WHO

ID	Date and time	Country or region	Continent	Confirmed patients	Cumulative Confirmed patients	Daily dead patients	Daily cumulative dead patients	Lag days	Daily CFR	3DMA CFR
7165	2020-4-15 8:00	MY	WPRO	170	4987	5	82	28	1.644	1.626
7166	2020-4-16 8:00	MY	WPRO	85	5072	1	83	29	1.636	1.634
7167	2020-4-17 8:00	MY	WPRO	110	5182	1	84	30	1.621	1.632
7168	2020-4-18 8:00	MY	WPRO	69	5251	2	86	31	1.638	1.639
7169	2020-4-19 8:00	MY	WPRO	54	5305	2	88	32	1.659	1.649
7170	2020-4-20 8:00	MY	WPRO	84	5389	1	89	33	1.652	1.650
7171	2020-4-21 8:00	MY	WPRO	35	5424	0	89	34	1.641	1.657
7172	2020-4-22 8:00	MY	WPRO	58	5482	3	92	35	1.678	1.667
7173	2020-4-23 8:00	MY	WPRO	50	5532	1	93	36	1.681	1.685
7174	2020-4-24 8:00	MY	WPRO	71	5603	2	95	37	1.696	1.688
7175	2020-4-25 8:00	MY	WPRO	88	5691	1	96	38	1.687	1.696
7176	2020-4-26 8:00	MY	WPRO	51	5742	2	98	39	1.707	1.696
7177	2020-4-27 8:00	MY	WPRO	38	5780	0	98	40	1.696	1.701
7178	2020-4-28 8:00	MY	WPRO	40	5820	1	99	41	1.701	1.702
7179	2020-4-29 8:00	MY	WPRO	31	5851	1	100	42	1.709	1.697
7180	2020-4-30 8:00	MY	WPRO	94	5945	0	100	43	1.682	1.697
7181	2020-5-1 8:00	MY	WPRO	57	6002	2	102	44	1.699	1.693
7182	2020-5-2 8:00	MY	WPRO	69	6071	1	103	45	1.697	1.698
7183										
7184	2020-3-7 8:00	MV	SEARO	2	2	0	0			
7185	2020-3-8 8:00	MV	SEARO	2	4	0	0			
7186	2020-3-9 8:00	MV	SEARO	0	4	0	0			
7187	2020-3-10 8:00	MV	SEARO	2	6	0	0			
7188	2020-3-11 8:00	MV	SEARO	2	8	0	0			
7189	2020-3-12 8:00	MV	SEARO	0	8	0	0			
7190	2020-3-13 8:00	MV	SEARO	0	8	0	0			
7191	2020-3-14 8:00	MV	SEARO	1	9	0	0			
7192	2020-3-15 8:00	MV	SEARO	4	13	0	0			
7193	2020-3-16 8:00	MV	SEARO	0	13	0	0			
7194	2020-3-17 8:00	MV	SEARO	0	13	0	0			
7195	2020-3-18 8:00	MV	SEARO	0	13	0	0			
7196	2020-3-19 8:00	MV	SEARO	0	13	0	0			
7197	2020-3-20 8:00	MV	SEARO	0	13	0	0			
7198	2020-3-21 8:00	MV	SEARO	0	13	0	0			
7199	2020-3-22 8:00	MV	SEARO	0	13	0	0			
7200	2020-3-23 8:00	MV	SEARO	0	13	0	0			

Table S1 Daily confirmed cases and daily deaths of COVID-19 in 214 nations from WHO

ID	Date and time	Country or region	Continent	Confirmed patients	Cumulative Confirmed patients	Daily dead patients	Daily cumulative dead patients	Lag days	Daily CFR	3DMA CFR
7201	2020-3-24 8:00	MV	SEARO	0	13	0	0			
7202	2020-3-25 8:00	MV	SEARO	0	13	0	0			
7203	2020-3-26 8:00	MV	SEARO	0	13	0	0			
7204	2020-3-27 8:00	MV	SEARO	0	13	0	0			
7205	2020-3-28 8:00	MV	SEARO	0	13	0	0			
7206	2020-3-29 8:00	MV	SEARO	3	16	0	0			
7207	2020-3-30 8:00	MV	SEARO	1	17	0	0			
7208	2020-3-31 8:00	MV	SEARO	0	17	0	0			
7209	2020-4-1 8:00	MV	SEARO	1	18	0	0			
7210	2020-4-2 8:00	MV	SEARO	1	19	0	0			
7211	2020-4-3 8:00	MV	SEARO	0	19	0	0			
7212	2020-4-4 8:00	MV	SEARO	0	19	0	0			
7213	2020-4-5 8:00	MV	SEARO	0	19	0	0			
7214	2020-4-6 8:00	MV	SEARO	0	19	0	0			
7215	2020-4-7 8:00	MV	SEARO	0	19	0	0			
7216	2020-4-8 8:00	MV	SEARO	0	19	0	0			
7217	2020-4-9 8:00	MV	SEARO	0	19	0	0			
7218	2020-4-10 8:00	MV	SEARO	0	19	0	0			
7219	2020-4-11 8:00	MV	SEARO	0	19	0	0			
7220	2020-4-12 8:00	MV	SEARO	1	20	0	0			
7221	2020-4-13 8:00	MV	SEARO	0	20	0	0			
7222	2020-4-14 8:00	MV	SEARO	0	20	0	0			
7223	2020-4-15 8:00	MV	SEARO	1	21	0	0			
7224	2020-4-16 8:00	MV	SEARO	2	23	0	0			
7225	2020-4-17 8:00	MV	SEARO	5	28	0	0			
7226	2020-4-18 8:00	MV	SEARO	6	34	0	0			
7227	2020-4-19 8:00	MV	SEARO	17	51	0	0			
7228	2020-4-20 8:00	MV	SEARO	9	60	0	0			
7229	2020-4-21 8:00	MV	SEARO	23	83	0	0			
7230	2020-4-22 8:00	MV	SEARO	2	85	0	0			
7231	2020-4-23 8:00	MV	SEARO	9	94	0	0			
7232	2020-4-24 8:00	MV	SEARO	22	116	0	0			
7233	2020-4-25 8:00	MV	SEARO	21	137	0	0			
7234	2020-4-26 8:00	MV	SEARO	51	188	0	0			
7235	2020-4-27 8:00	MV	SEARO	26	214	0	0			
7236	2020-4-28 8:00	MV	SEARO	31	245	0	0			

Table S1 Daily confirmed cases and daily deaths of COVID-19 in 214 nations from WHO

ID	Date and time	Country or region	Continent	Confirmed patients	Cumulative Confirmed patients	Daily dead patients	Daily cumulative dead patients	Lag days	Daily CFR	3DMA CFR
7237	2020-4-29 8:00	MV	SEARO	11	256	0	0			
7238	2020-4-30 8:00	MV	SEARO	140	396	0	0			
7239	2020-5-1 8:00	MV	SEARO	72	468	1	1	0	0.214	0.214
7240	2020-5-2 8:00	MV	SEARO	0	468	0	1	1	0.214	0.214
7241										
7242	2020-3-26 8:00	ML	AFRO	2	2	0	0			
7243	2020-3-27 8:00	ML	AFRO	0	2	0	0			
7244	2020-3-28 8:00	ML	AFRO	7	9	0	0			
7245	2020-3-29 8:00	ML	AFRO	9	18	0	0			
7246	2020-3-30 8:00	ML	AFRO	0	18	0	0			
7247	2020-3-31 8:00	ML	AFRO	0	18	0	0			
7248	2020-4-1 8:00	ML	AFRO	0	18	0	0			
7249	2020-4-2 8:00	ML	AFRO	10	28	0	0			
7250	2020-4-3 8:00	ML	AFRO	0	28	3	3	0	10.714	9.524
7251	2020-4-4 8:00	ML	AFRO	8	36	0	3	1	8.333	9.768
7252	2020-4-5 8:00	ML	AFRO	3	39	1	4	2	10.256	9.615
7253	2020-4-6 8:00	ML	AFRO	0	39	0	4	3	10.256	10.256
7254	2020-4-7 8:00	ML	AFRO	0	39	0	4	4	10.256	10.384
7255	2020-4-8 8:00	ML	AFRO	8	47	1	5	5	10.638	10.536
7256	2020-4-9 8:00	ML	AFRO	9	56	1	6	6	10.714	11.072
7257	2020-4-10 8:00	ML	AFRO	3	59	1	7	7	11.864	10.679
7258	2020-4-11 8:00	ML	AFRO	15	74	0	7	8	9.459	9.790
7259	2020-4-12 8:00	ML	AFRO	13	87	0	7	9	8.046	8.517
7260	2020-4-13 8:00	ML	AFRO	0	87	0	7	10	8.046	7.950
7261	2020-4-14 8:00	ML	AFRO	29	116	2	9	11	7.759	7.978
7262	2020-4-15 8:00	ML	AFRO	7	123	1	10	12	8.130	8.305
7263	2020-4-16 8:00	ML	AFRO	21	144	3	13	13	9.028	8.253
7264	2020-4-17 8:00	ML	AFRO	27	171	0	13	14	7.602	7.824
7265	2020-4-18 8:00	ML	AFRO	19	190	0	13	15	6.842	6.821
7266	2020-4-19 8:00	ML	AFRO	26	216	0	13	16	6.019	6.370
7267	2020-4-20 8:00	ML	AFRO	8	224	1	14	17	6.250	5.987
7268	2020-4-21 8:00	ML	AFRO	22	246	0	14	18	5.691	5.789
7269	2020-4-22 8:00	ML	AFRO	12	258	0	14	19	5.426	5.640
7270	2020-4-23 8:00	ML	AFRO	35	293	3	17	20	5.802	6.008
7271	2020-4-24 8:00	ML	AFRO	16	309	4	21	21	6.796	6.353
7272	2020-4-25 8:00	ML	AFRO	16	325	0	21	22	6.462	6.311

Table S1 Daily confirmed cases and daily deaths of COVID-19 in 214 nations from WHO

ID	Date and time	Country or region	Continent	Confirmed patients	Cumulative Confirmed patients	Daily dead patients	Daily cumulative dead patients	Lag days	Daily CFR	3DMA CFR
7273	2020-4-26 8:00	ML	AFRO	45	370	0	21	23	5.676	6.017
7274	2020-4-27 8:00	ML	AFRO	19	389	2	23	24	5.913	5.742
7275	2020-4-28 8:00	ML	AFRO	19	408	0	23	25	5.637	5.737
7276	2020-4-29 8:00	ML	AFRO	16	424	1	24	26	5.660	5.495
7277	2020-4-30 8:00	ML	AFRO	58	482	1	25	27	5.187	5.384
7278	2020-5-1 8:00	ML	AFRO	8	490	1	26	28	5.306	5.204
7279	2020-5-2 8:00	ML	AFRO	18	508	0	26	29	5.118	5.212
7280										
7281	2020-3-7 8:00	MT	EURO	3	3	0	0			
7282	2020-3-8 8:00	MT	EURO	0	3	0	0			
7283	2020-3-9 8:00	MT	EURO	0	3	0	0			
7284	2020-3-10 8:00	MT	EURO	1	4	0	0			
7285	2020-3-11 8:00	MT	EURO	2	6	0	0			
7286	2020-3-12 8:00	MT	EURO	3	9	0	0			
7287	2020-3-13 8:00	MT	EURO	3	12	0	0			
7288	2020-3-14 8:00	MT	EURO	0	12	0	0			
7289	2020-3-15 8:00	MT	EURO	9	21	0	0			
7290	2020-3-16 8:00	MT	EURO	9	30	0	0			
7291	2020-3-17 8:00	MT	EURO	8	38	0	0			
7292	2020-3-18 8:00	MT	EURO	10	48	0	0			
7293	2020-3-19 8:00	MT	EURO	5	53	0	0			
7294	2020-3-20 8:00	MT	EURO	11	64	0	0			
7295	2020-3-21 8:00	MT	EURO	9	73	0	0			
7296	2020-3-22 8:00	MT	EURO	0	73	0	0			
7297	2020-3-23 8:00	MT	EURO	17	90	0	0			
7298	2020-3-24 8:00	MT	EURO	17	107	0	0			
7299	2020-3-25 8:00	MT	EURO	13	120	0	0			
7300	2020-3-26 8:00	MT	EURO	9	129	0	0			
7301	2020-3-27 8:00	MT	EURO	5	134	0	0			
7302	2020-3-28 8:00	MT	EURO	5	139	0	0			
7303	2020-3-29 8:00	MT	EURO	0	139	0	0			
7304	2020-3-30 8:00	MT	EURO	12	151	0	0			
7305	2020-3-31 8:00	MT	EURO	5	156	0	0			
7306	2020-4-1 8:00	MT	EURO	11	167	0	0			
7307	2020-4-2 8:00	MT	EURO	21	188	0	0			
7308	2020-4-3 8:00	MT	EURO	7	195	0	0			

Table S1 Daily confirmed cases and daily deaths of COVID-19 in 214 nations from WHO

ID	Date and time	Country or region	Continent	Confirmed patients	Cumulative Confirmed patients	Daily dead patients	Daily cumulative dead patients	Lag days	Daily CFR	3DMA CFR
7309	2020-4-4 8:00	MT	EURO	7	202	0	0			
7310	2020-4-5 8:00	MT	EURO	11	213	0	0			
7311	2020-4-6 8:00	MT	EURO	21	234	0	0			
7312	2020-4-7 8:00	MT	EURO	7	241	0	0			
7313	2020-4-8 8:00	MT	EURO	52	293	0	0			
7314	2020-4-9 8:00	MT	EURO	6	299	1	1	0	0.334	0.464
7315	2020-4-10 8:00	MT	EURO	38	337	1	2	1	0.593	0.500
7316	2020-4-11 8:00	MT	EURO	13	350	0	2	2	0.571	0.659
7317	2020-4-12 8:00	MT	EURO	20	370	1	3	3	0.811	0.725
7318	2020-4-13 8:00	MT	EURO	8	378	0	3	4	0.794	0.795
7319	2020-4-14 8:00	MT	EURO	6	384	0	3	5	0.781	0.779
7320	2020-4-15 8:00	MT	EURO	9	393	0	3	6	0.763	0.765
7321	2020-4-16 8:00	MT	EURO	6	399	0	3	7	0.752	0.748
7322	2020-4-17 8:00	MT	EURO	13	412	0	3	8	0.728	0.730
7323	2020-4-18 8:00	MT	EURO	10	422	0	3	9	0.711	0.714
7324	2020-4-19 8:00	MT	EURO	4	426	0	3	10	0.704	0.706
7325	2020-4-20 8:00	MT	EURO	1	427	0	3	11	0.703	0.701
7326	2020-4-21 8:00	MT	EURO	4	431	0	3	12	0.696	0.692
7327	2020-4-22 8:00	MT	EURO	12	443	0	3	13	0.677	0.683
7328	2020-4-23 8:00	MT	EURO	1	444	0	3	14	0.676	0.676
7329	2020-4-24 8:00	MT	EURO	1	445	0	3	15	0.674	0.674
7330	2020-4-25 8:00	MT	EURO	2	447	0	3	16	0.671	0.746
7331	2020-4-26 8:00	MT	EURO	1	448	1	4	17	0.893	0.819
7332	2020-4-27 8:00	MT	EURO	0	448	0	4	18	0.893	0.892
7333	2020-4-28 8:00	MT	EURO	2	450	0	4	19	0.889	0.890
7334	2020-4-29 8:00	MT	EURO	0	450	0	4	20	0.889	0.881
7335	2020-4-30 8:00	MT	EURO	13	463	0	4	21	0.864	0.871
7336	2020-5-1 8:00	MT	EURO	2	465	0	4	22	0.860	0.860
7337	2020-5-2 8:00	MT	EURO	2	467	0	4	23	0.857	0.858
7338										
7339	2020-3-7 8:00	MQ	AMRO	2	2	0	0			
7340	2020-3-8 8:00	MQ	AMRO	0	2	0	0			
7341	2020-3-9 8:00	MQ	AMRO	0	2	0	0			
7342	2020-3-10 8:00	MQ	AMRO	0	2	0	0			
7343	2020-3-11 8:00	MQ	AMRO	1	3	0	0			
7344	2020-3-12 8:00	MQ	AMRO	0	3	0	0			

Table S1 Daily confirmed cases and daily deaths of COVID-19 in 214 nations from WHO

ID	Date and time	Country or region	Continent	Confirmed patients	Cumulative Confirmed patients	Daily dead patients	Daily cumulative dead patients	Lag days	Daily CFR	3DMA CFR
7345	2020-3-13 8:00	MQ	AMRO	1	4	0	0			
7346	2020-3-14 8:00	MQ	AMRO	6	10	0	0			
7347	2020-3-15 8:00	MQ	AMRO	5	15	0	0			
7348	2020-3-16 8:00	MQ	AMRO	1	16	0	0			
7349	2020-3-17 8:00	MQ	AMRO	0	16	0	0			
7350	2020-3-18 8:00	MQ	AMRO	7	23	0	0			
7351	2020-3-19 8:00	MQ	AMRO	9	32	0	0			
7352	2020-3-20 8:00	MQ	AMRO	0	32	0	0			
7353	2020-3-21 8:00	MQ	AMRO	0	32	0	0			
7354	2020-3-22 8:00	MQ	AMRO	0	32	0	0			
7355	2020-3-23 8:00	MQ	AMRO	5	37	0	0			
7356	2020-3-24 8:00	MQ	AMRO	20	57	0	0			
7357	2020-3-25 8:00	MQ	AMRO	0	57	0	0			
7358	2020-3-26 8:00	MQ	AMRO	9	66	1	1	0	1.515	1.515
7359	2020-3-27 8:00	MQ	AMRO	0	66	0	1	1	1.515	1.369
7360	2020-3-28 8:00	MQ	AMRO	27	93	0	1	2	1.075	1.222
7361	2020-3-29 8:00	MQ	AMRO	0	93	0	1	3	1.075	1.352
7362	2020-3-30 8:00	MQ	AMRO	12	105	1	2	4	1.905	1.594
7363	2020-3-31 8:00	MQ	AMRO	6	111	0	2	5	1.802	1.796
7364	2020-4-1 8:00	MQ	AMRO	8	119	0	2	6	1.681	1.942
7365	2020-4-2 8:00	MQ	AMRO	9	128	1	3	7	2.344	2.105
7366	2020-4-3 8:00	MQ	AMRO	3	131	0	3	8	2.290	2.269
7367	2020-4-4 8:00	MQ	AMRO	7	138	0	3	9	2.174	2.178
7368	2020-4-5 8:00	MQ	AMRO	7	145	0	3	10	2.069	2.104
7369	2020-4-6 8:00	MQ	AMRO	0	145	0	3	11	2.069	2.274
7370	2020-4-7 8:00	MQ	AMRO	4	149	1	4	12	2.685	2.468
7371	2020-4-8 8:00	MQ	AMRO	2	151	0	4	13	2.649	2.655
7372	2020-4-9 8:00	MQ	AMRO	1	152	0	4	14	2.632	3.059
7373	2020-4-10 8:00	MQ	AMRO	2	154	2	6	15	3.896	3.475
7374	2020-4-11 8:00	MQ	AMRO	0	154	0	6	16	3.896	3.888
7375	2020-4-12 8:00	MQ	AMRO	1	155	0	6	17	3.871	3.871
7376	2020-4-13 8:00	MQ	AMRO	1	156	0	6	18	3.846	4.271
7377	2020-4-14 8:00	MQ	AMRO	1	157	2	8	19	5.096	4.679
7378	2020-4-15 8:00	MQ	AMRO	0	157	0	8	20	5.096	5.085
7379	2020-4-16 8:00	MQ	AMRO	1	158	0	8	21	5.063	5.063
7380	2020-4-17 8:00	MQ	AMRO	1	159	0	8	22	5.031	5.042

Table S1 Daily confirmed cases and daily deaths of COVID-19 in 214 nations from WHO

ID	Date and time	Country or region	Continent	Confirmed patients	Cumulative Confirmed patients	Daily dead patients	Daily cumulative dead patients	Lag days	Daily CFR	3DMA CFR
7381	2020-4-18 8:00	MQ	AMRO	0	159	0	8	23	5.031	5.808
7382	2020-4-19 8:00	MQ	AMRO	4	163	4	12	24	7.362	6.585
7383	2020-4-20 8:00	MQ	AMRO	0	163	0	12	25	7.362	7.771
7384	2020-4-21 8:00	MQ	AMRO	0	163	2	14	26	8.589	8.180
7385	2020-4-22 8:00	MQ	AMRO	0	163	0	14	27	8.589	8.571
7386	2020-4-23 8:00	MQ	AMRO	1	164	0	14	28	8.537	8.454
7387	2020-4-24 8:00	MQ	AMRO	6	170	0	14	29	8.235	8.336
7388	2020-4-25 8:00	MQ	AMRO	0	170	0	14	30	8.235	8.235
7389	2020-4-26 8:00	MQ	AMRO	0	170	0	14	31	8.235	8.157
7390	2020-4-27 8:00	MQ	AMRO	5	175	0	14	32	8.000	8.078
7391	2020-4-28 8:00	MQ	AMRO	0	175	0	14	33	8.000	8.000
7392	2020-4-29 8:00	MQ	AMRO	0	175	0	14	34	8.000	8.000
7393	2020-4-30 8:00	MQ	AMRO	0	175	0	14	35	8.000	7.955
7394	2020-5-1 8:00	MQ	AMRO	3	178	0	14	36	7.865	7.895
7395	2020-5-2 8:00	MQ	AMRO	1	179	0	14	37	7.821	7.843
7396										
7397	2020-3-14 8:00	MR	AFRO	1	1	0	0			
7398	2020-3-15 8:00	MR	AFRO	0	1	0	0			
7399	2020-3-16 8:00	MR	AFRO	0	1	0	0			
7400	2020-3-17 8:00	MR	AFRO	0	1	0	0			
7401	2020-3-18 8:00	MR	AFRO	1	2	0	0			
7402	2020-3-19 8:00	MR	AFRO	0	2	0	0			
7403	2020-3-20 8:00	MR	AFRO	0	2	0	0			
7404	2020-3-21 8:00	MR	AFRO	0	2	0	0			
7405	2020-3-22 8:00	MR	AFRO	0	2	0	0			
7406	2020-3-23 8:00	MR	AFRO	0	2	0	0			
7407	2020-3-24 8:00	MR	AFRO	0	2	0	0			
7408	2020-3-25 8:00	MR	AFRO	0	2	0	0			
7409	2020-3-26 8:00	MR	AFRO	0	2	0	0			
7410	2020-3-27 8:00	MR	AFRO	1	3	0	0			
7411	2020-3-28 8:00	MR	AFRO	2	5	0	0			
7412	2020-3-29 8:00	MR	AFRO	0	5	0	0			
7413	2020-3-30 8:00	MR	AFRO	0	5	0	0			
7414	2020-3-31 8:00	MR	AFRO	0	5	0	0			
7415	2020-4-1 8:00	MR	AFRO	0	5	0	0			
7416	2020-4-2 8:00	MR	AFRO	0	5	0	0			

Table S1 Daily confirmed cases and daily deaths of COVID-19 in 214 nations from WHO

ID	Date and time	Country or region	Continent	Confirmed patients	Cumulative Confirmed patients	Daily dead patients	Daily cumulative dead patients	Lag days	Daily CFR	3DMA CFR
7417	2020-4-3 8:00	MR	AFRO	0	5	0	0			
7418	2020-4-4 8:00	MR	AFRO	1	6	1	1	0	16.667	16.667
7419	2020-4-5 8:00	MR	AFRO	0	6	0	1	1	16.667	16.667
7420	2020-4-6 8:00	MR	AFRO	0	6	0	1	2	16.667	16.667
7421	2020-4-7 8:00	MR	AFRO	0	6	0	1	3	16.667	16.667
7422	2020-4-8 8:00	MR	AFRO	0	6	0	1	4	16.667	16.667
7423	2020-4-9 8:00	MR	AFRO	0	6	0	1	5	16.667	16.667
7424	2020-4-10 8:00	MR	AFRO	0	6	0	1	6	16.667	15.873
7425	2020-4-11 8:00	MR	AFRO	1	7	0	1	7	14.286	15.079
7426	2020-4-12 8:00	MR	AFRO	0	7	0	1	8	14.286	14.286
7427	2020-4-13 8:00	MR	AFRO	0	7	0	1	9	14.286	14.286
7428	2020-4-14 8:00	MR	AFRO	0	7	0	1	10	14.286	14.286
7429	2020-4-15 8:00	MR	AFRO	0	7	0	1	11	14.286	14.286
7430	2020-4-16 8:00	MR	AFRO	0	7	0	1	12	14.286	14.286
7431	2020-4-17 8:00	MR	AFRO	0	7	0	1	13	14.286	14.286
7432	2020-4-18 8:00	MR	AFRO	0	7	0	1	14	14.286	14.286
7433	2020-4-19 8:00	MR	AFRO	0	7	0	1	15	14.286	14.286
7434	2020-4-20 8:00	MR	AFRO	0	7	0	1	16	14.286	14.286
7435	2020-4-21 8:00	MR	AFRO	0	7	0	1	17	14.286	14.286
7436	2020-4-22 8:00	MR	AFRO	0	7	0	1	18	14.286	14.286
7437	2020-4-23 8:00	MR	AFRO	0	7	0	1	19	14.286	14.286
7438	2020-4-24 8:00	MR	AFRO	0	7	0	1	20	14.286	14.286
7439	2020-4-25 8:00	MR	AFRO	0	7	0	1	21	14.286	14.286
7440	2020-4-26 8:00	MR	AFRO	0	7	0	1	22	14.286	14.286
7441	2020-4-27 8:00	MR	AFRO	0	7	0	1	23	14.286	14.286
7442	2020-4-28 8:00	MR	AFRO	0	7	0	1	24	14.286	14.286
7443	2020-4-29 8:00	MR	AFRO	0	7	0	1	25	14.286	14.286
7444	2020-4-30 8:00	MR	AFRO	0	7	0	1	26	14.286	13.690
7445	2020-5-1 8:00	MR	AFRO	1	8	0	1	27	12.500	13.095
7446	2020-5-2 8:00	MR	AFRO	0	8	0	1	28	12.500	12.500
7447										
7448	2020-3-18 8:00	MU	AFRO	3	3	0	0			
7449	2020-3-19 8:00	MU	AFRO	4	7	0	0			
7450	2020-3-20 8:00	MU	AFRO	5	12	0	0			
7451	2020-3-21 8:00	MU	AFRO	0	12	0	0			
7452	2020-3-22 8:00	MU	AFRO	0	12	0	0			

Table S1 Daily confirmed cases and daily deaths of COVID-19 in 214 nations from WHO

ID	Date and time	Country or region	Continent	Confirmed patients	Cumulative Confirmed patients	Daily dead patients	Daily cumulative dead patients	Lag days	Daily CFR	3DMA CFR
7453	2020-3-23 8:00	MU	AFRO	0	12	0	0			
7454	2020-3-24 8:00	MU	AFRO	24	36	0	0			
7455	2020-3-25 8:00	MU	AFRO	6	42	2	2	0	4.762	4.509
7456	2020-3-26 8:00	MU	AFRO	5	47	0	2	1	4.255	3.829
7457	2020-3-27 8:00	MU	AFRO	34	81	0	2	2	2.469	2.936
7458	2020-3-28 8:00	MU	AFRO	15	96	0	2	3	2.083	2.171
7459	2020-3-29 8:00	MU	AFRO	6	102	0	2	4	1.961	2.002
7460	2020-3-30 8:00	MU	AFRO	0	102	0	2	5	1.961	1.828
7461	2020-3-31 8:00	MU	AFRO	26	128	0	2	6	1.563	2.340
7462	2020-4-1 8:00	MU	AFRO	15	143	3	5	7	3.497	2.769
7463	2020-4-2 8:00	MU	AFRO	11	154	0	5	8	3.247	3.628
7464	2020-4-3 8:00	MU	AFRO	15	169	2	7	9	4.142	3.717
7465	2020-4-4 8:00	MU	AFRO	17	186	0	7	10	3.763	3.826
7466	2020-4-5 8:00	MU	AFRO	10	196	0	7	11	3.571	3.473
7467	2020-4-6 8:00	MU	AFRO	31	227	0	7	12	3.084	3.175
7468	2020-4-7 8:00	MU	AFRO	17	244	0	7	13	2.869	2.855
7469	2020-4-8 8:00	MU	AFRO	24	268	0	7	14	2.612	2.682
7470	2020-4-9 8:00	MU	AFRO	5	273	0	7	15	2.564	2.468
7471	2020-4-10 8:00	MU	AFRO	41	314	0	7	16	2.229	2.541
7472	2020-4-11 8:00	MU	AFRO	4	318	2	9	17	2.830	2.627
7473	2020-4-12 8:00	MU	AFRO	1	319	0	9	18	2.821	2.810
7474	2020-4-13 8:00	MU	AFRO	5	324	0	9	19	2.778	2.792
7475	2020-4-14 8:00	MU	AFRO	0	324	0	9	20	2.778	2.778
7476	2020-4-15 8:00	MU	AFRO	0	324	0	9	21	2.778	2.778
7477	2020-4-16 8:00	MU	AFRO	0	324	0	9	22	2.778	2.778
7478	2020-4-17 8:00	MU	AFRO	0	324	0	9	23	2.778	2.778
7479	2020-4-18 8:00	MU	AFRO	0	324	0	9	24	2.778	2.775
7480	2020-4-19 8:00	MU	AFRO	1	325	0	9	25	2.769	2.764
7481	2020-4-20 8:00	MU	AFRO	3	328	0	9	26	2.744	2.752
7482	2020-4-21 8:00	MU	AFRO	0	328	0	9	27	2.744	2.744
7483	2020-4-22 8:00	MU	AFRO	0	328	0	9	28	2.744	2.741
7484	2020-4-23 8:00	MU	AFRO	1	329	0	9	29	2.736	2.733
7485	2020-4-24 8:00	MU	AFRO	2	331	0	9	30	2.719	2.725
7486	2020-4-25 8:00	MU	AFRO	0	331	0	9	31	2.719	2.719
7487	2020-4-26 8:00	MU	AFRO	0	331	0	9	32	2.719	2.716
7488	2020-4-27 8:00	MU	AFRO	1	332	0	9	33	2.711	2.714

Table S1 Daily confirmed cases and daily deaths of COVID-19 in 214 nations from WHO

ID	Date and time	Country or region	Continent	Confirmed patients	Cumulative Confirmed patients	Daily dead patients	Daily cumulative dead patients	Lag days	Daily CFR	3DMA CFR
7489	2020-4-28 8:00	MU	AFRO	0	332	0	9	34	2.711	2.811
7490	2020-4-29 8:00	MU	AFRO	0	332	1	10	35	3.012	2.912
7491	2020-4-30 8:00	MU	AFRO	0	332	0	10	36	3.012	3.012
7492	2020-5-1 8:00	MU	AFRO	0	332	0	10	37	3.012	3.012
7493	2020-5-2 8:00	MU	AFRO	0	332	0	10	38	3.012	3.012
7494										
7495	2020-3-14 8:00	YT	AFRO	1	1	0	0			
7496	2020-3-15 8:00	YT	AFRO	0	1	0	0			
7497	2020-3-16 8:00	YT	AFRO	0	1	0	0			
7498	2020-3-17 8:00	YT	AFRO	0	1	0	0			
7499	2020-3-18 8:00	YT	AFRO	2	3	0	0			
7500	2020-3-19 8:00	YT	AFRO	1	4	0	0			
7501	2020-3-20 8:00	YT	AFRO	0	4	0	0			
7502	2020-3-21 8:00	YT	AFRO	7	11	0	0			
7503	2020-3-22 8:00	YT	AFRO	0	11	0	0			
7504	2020-3-23 8:00	YT	AFRO	3	14	0	0			
7505	2020-3-24 8:00	YT	AFRO	10	24	0	0			
7506	2020-3-25 8:00	YT	AFRO	6	30	0	0			
7507	2020-3-26 8:00	YT	AFRO	5	35	0	0			
7508	2020-3-27 8:00	YT	AFRO	15	50	0	0			
7509	2020-3-28 8:00	YT	AFRO	0	50	0	0			
7510	2020-3-29 8:00	YT	AFRO	0	50	0	0			
7511	2020-3-30 8:00	YT	AFRO	32	82	0	0			
7512	2020-3-31 8:00	YT	AFRO	0	82	0	0			
7513	2020-4-1 8:00	YT	AFRO	19	101	0	0			
7514	2020-4-2 8:00	YT	AFRO	15	116	2	2	0	1.724	1.643
7515	2020-4-3 8:00	YT	AFRO	12	128	0	2	1	1.563	1.616
7516	2020-4-4 8:00	YT	AFRO	0	128	0	2	2	1.563	1.495
7517	2020-4-5 8:00	YT	AFRO	19	147	0	2	3	1.361	1.428
7518	2020-4-6 8:00	YT	AFRO	0	147	0	2	4	1.361	1.314
7519	2020-4-7 8:00	YT	AFRO	17	164	0	2	5	1.220	1.222
7520	2020-4-8 8:00	YT	AFRO	20	184	0	2	6	1.087	1.127
7521	2020-4-9 8:00	YT	AFRO	2	186	0	2	7	1.075	1.070
7522	2020-4-10 8:00	YT	AFRO	5	191	0	2	8	1.047	1.057
7523	2020-4-11 8:00	YT	AFRO	0	191	0	2	9	1.047	1.047
7524	2020-4-12 8:00	YT	AFRO	0	191	0	2	10	1.047	1.181

Table S1 Daily confirmed cases and daily deaths of COVID-19 in 214 nations from WHO

ID	Date and time	Country or region	Continent	Confirmed patients	Cumulative Confirmed patients	Daily dead patients	Daily cumulative dead patients	Lag days	Daily CFR	3DMA CFR
7525	2020-4-13 8:00	YT	AFRO	16	207	1	3	11	1.449	1.315
7526	2020-4-14 8:00	YT	AFRO	0	207	0	3	12	1.449	1.427
7527	2020-4-15 8:00	YT	AFRO	10	217	0	3	13	1.382	1.396
7528	2020-4-16 8:00	YT	AFRO	4	221	0	3	14	1.357	1.343
7529	2020-4-17 8:00	YT	AFRO	12	233	0	3	15	1.288	1.426
7530	2020-4-18 8:00	YT	AFRO	12	245	1	4	16	1.633	1.518
7531	2020-4-19 8:00	YT	AFRO	0	245	0	4	17	1.633	1.558
7532	2020-4-20 8:00	YT	AFRO	39	284	0	4	18	1.408	1.483
7533	2020-4-21 8:00	YT	AFRO	0	284	0	4	19	1.408	1.368
7534	2020-4-22 8:00	YT	AFRO	27	311	0	4	20	1.286	1.307
7535	2020-4-23 8:00	YT	AFRO	15	326	0	4	21	1.227	1.231
7536	2020-4-24 8:00	YT	AFRO	13	339	0	4	22	1.180	1.179
7537	2020-4-25 8:00	YT	AFRO	15	354	0	4	23	1.130	1.147
7538	2020-4-26 8:00	YT	AFRO	0	354	0	4	24	1.130	1.086
7539	2020-4-27 8:00	YT	AFRO	47	401	0	4	25	0.998	1.017
7540	2020-4-28 8:00	YT	AFRO	32	433	0	4	26	0.924	0.930
7541	2020-4-29 8:00	YT	AFRO	27	460	0	4	27	0.870	0.888
7542	2020-4-30 8:00	YT	AFRO	0	460	0	4	28	0.870	0.827
7543	2020-5-1 8:00	YT	AFRO	79	539	0	4	29	0.742	0.785
7544	2020-5-2 8:00	YT	AFRO	0	539	0	4	30	0.742	0.742
7545										
7546	2020-2-28 8:00	MX	AMRO	2	2	0	0			
7547	2020-2-29 8:00	MX	AMRO	0	2	0	0			
7548	2020-3-1 8:00	MX	AMRO	0	2	0	0			
7549	2020-3-2 8:00	MX	AMRO	3	5	0	0			
7550	2020-3-3 8:00	MX	AMRO	0	5	0	0			
7551	2020-3-4 8:00	MX	AMRO	0	5	0	0			
7552	2020-3-5 8:00	MX	AMRO	0	5	0	0			
7553	2020-3-6 8:00	MX	AMRO	0	5	0	0			
7554	2020-3-7 8:00	MX	AMRO	0	5	0	0			
7555	2020-3-8 8:00	MX	AMRO	2	7	0	0			
7556	2020-3-9 8:00	MX	AMRO	0	7	0	0			
7557	2020-3-10 8:00	MX	AMRO	0	7	0	0			
7558	2020-3-11 8:00	MX	AMRO	0	7	0	0			
7559	2020-3-12 8:00	MX	AMRO	4	11	0	0			
7560	2020-3-13 8:00	MX	AMRO	1	12	0	0			

Table S1 Daily confirmed cases and daily deaths of COVID-19 in 214 nations from WHO

ID	Date and time	Country or region	Continent	Confirmed patients	Cumulative Confirmed patients	Daily dead patients	Daily cumulative dead patients	Lag days	Daily CFR	3DMA CFR
7561	2020-3-14 8:00	MX	AMRO	14	26	0	0			
7562	2020-3-15 8:00	MX	AMRO	15	41	0	0			
7563	2020-3-16 8:00	MX	AMRO	12	53	0	0			
7564	2020-3-17 8:00	MX	AMRO	29	82	0	0			
7565	2020-3-18 8:00	MX	AMRO	11	93	0	0			
7566	2020-3-19 8:00	MX	AMRO	25	118	1	1	0	0.847	0.729
7567	2020-3-20 8:00	MX	AMRO	46	164	0	1	1	0.610	0.689
7568	2020-3-21 8:00	MX	AMRO	0	164	0	1	2	0.610	0.610
7569	2020-3-22 8:00	MX	AMRO	0	164	0	1	3	0.610	0.672
7570	2020-3-23 8:00	MX	AMRO	87	251	1	2	4	0.797	0.829
7571	2020-3-24 8:00	MX	AMRO	119	370	2	4	5	1.081	0.986
7572	2020-3-25 8:00	MX	AMRO	0	370	0	4	6	1.081	1.069
7573	2020-3-26 8:00	MX	AMRO	108	478	1	5	7	1.046	1.058
7574	2020-3-27 8:00	MX	AMRO	0	478	0	5	8	1.046	1.150
7575	2020-3-28 8:00	MX	AMRO	111	589	3	8	9	1.358	1.359
7576	2020-3-29 8:00	MX	AMRO	128	717	4	12	10	1.674	1.640
7577	2020-3-30 8:00	MX	AMRO	131	848	4	16	11	1.887	1.858
7578	2020-3-31 8:00	MX	AMRO	145	993	4	20	12	2.014	2.153
7579	2020-4-1 8:00	MX	AMRO	101	1094	8	28	13	2.559	2.320
7580	2020-4-2 8:00	MX	AMRO	121	1215	1	29	14	2.387	2.544
7581	2020-4-3 8:00	MX	AMRO	163	1378	8	37	15	2.685	2.794
7582	2020-4-4 8:00	MX	AMRO	132	1510	13	50	16	3.311	3.184
7583	2020-4-5 8:00	MX	AMRO	178	1688	10	60	17	3.555	3.682
7584	2020-4-6 8:00	MX	AMRO	202	1890	19	79	18	4.180	4.040
7585	2020-4-7 8:00	MX	AMRO	253	2143	15	94	19	4.386	4.564
7586	2020-4-8 8:00	MX	AMRO	296	2439	31	125	20	5.125	4.858
7587	2020-4-9 8:00	MX	AMRO	346	2785	16	141	21	5.063	5.219
7588	2020-4-10 8:00	MX	AMRO	396	3181	33	174	22	5.470	5.390
7589	2020-4-11 8:00	MX	AMRO	260	3441	20	194	23	5.638	5.723
7590	2020-4-12 8:00	MX	AMRO	403	3844	39	233	24	6.061	6.057
7591	2020-4-13 8:00	MX	AMRO	375	4219	40	273	25	6.471	6.294
7592	2020-4-14 8:00	MX	AMRO	442	4661	23	296	26	6.351	6.481
7593	2020-4-15 8:00	MX	AMRO	353	5014	36	332	27	6.621	6.831
7594	2020-4-16 8:00	MX	AMRO	385	5399	74	406	28	7.520	7.274
7595	2020-4-17 8:00	MX	AMRO	448	5847	43	449	29	7.679	7.639
7596	2020-4-18 8:00	MX	AMRO	450	6297	37	486	30	7.718	7.780

Table S1 Daily confirmed cases and daily deaths of COVID-19 in 214 nations from WHO

ID	Date and time	Country or region	Continent	Confirmed patients	Cumulative Confirmed patients	Daily dead patients	Daily cumulative dead patients	Lag days	Daily CFR	3DMA CFR
7597	2020-4-19 8:00	MX	AMRO	578	6875	60	546	31	7.942	8.110
7598	2020-4-20 8:00	MX	AMRO	622	7497	104	650	32	8.670	8.305
7599	2020-4-21 8:00	MX	AMRO	764	8261	36	686	33	8.304	8.364
7600	2020-4-22 8:00	MX	AMRO	511	8772	26	712	34	8.117	8.480
7601	2020-4-23 8:00	MX	AMRO	729	9501	145	857	35	9.020	8.779
7602	2020-4-24 8:00	MX	AMRO	1043	10544	113	970	36	9.200	9.136
7603	2020-4-25 8:00	MX	AMRO	1089	11633	99	1069	37	9.189	9.292
7604	2020-4-26 8:00	MX	AMRO	1239	12872	152	1221	38	9.486	9.368
7605	2020-4-27 8:00	MX	AMRO	970	13842	84	1305	39	9.428	9.373
7606	2020-4-28 8:00	MX	AMRO	835	14677	46	1351	40	9.205	9.289
7607	2020-4-29 8:00	MX	AMRO	852	15529	83	1434	41	9.234	9.268
7608	2020-4-30 8:00	MX	AMRO	1223	16752	135	1569	42	9.366	9.444
7609	2020-5-1 8:00	MX	AMRO	1047	17799	163	1732	43	9.731	9.589
7610	2020-5-2 8:00	MX	AMRO	1425	19224	127	1859	44	9.670	9.701
7611										
7612	2020-2-29 8:00	MC	EURO	1	1	0	0			
7613	2020-3-1 8:00	MC	EURO	0	1	0	0			
7614	2020-3-2 8:00	MC	EURO	0	1	0	0			
7615	2020-3-3 8:00	MC	EURO	0	1	0	0			
7616	2020-3-4 8:00	MC	EURO	0	1	0	0			
7617	2020-3-5 8:00	MC	EURO	0	1	0	0			
7618	2020-3-6 8:00	MC	EURO	0	1	0	0			
7619	2020-3-7 8:00	MC	EURO	0	1	0	0			
7620	2020-3-8 8:00	MC	EURO	0	1	0	0			
7621	2020-3-9 8:00	MC	EURO	0	1	0	0			
7622	2020-3-10 8:00	MC	EURO	0	1	0	0			
7623	2020-3-11 8:00	MC	EURO	0	1	0	0			
7624	2020-3-12 8:00	MC	EURO	0	1	0	0			
7625	2020-3-13 8:00	MC	EURO	1	2	0	0			
7626	2020-3-14 8:00	MC	EURO	0	2	0	0			
7627	2020-3-15 8:00	MC	EURO	0	2	0	0			
7628	2020-3-16 8:00	MC	EURO	7	9	0	0			
7629	2020-3-17 8:00	MC	EURO	0	9	0	0			
7630	2020-3-18 8:00	MC	EURO	0	9	0	0			
7631	2020-3-19 8:00	MC	EURO	0	9	0	0			
7632	2020-3-20 8:00	MC	EURO	3	12	0	0			

Table S1 Daily confirmed cases and daily deaths of COVID-19 in 214 nations from WHO

ID	Date and time	Country or region	Continent	Confirmed patients	Cumulative Confirmed patients	Daily dead patients	Daily cumulative dead patients	Lag days	Daily CFR	3DMA CFR
7633	2020-3-21 8:00	MC	EURO	6	18	0	0			
7634	2020-3-22 8:00	MC	EURO	0	18	0	0			
7635	2020-3-23 8:00	MC	EURO	1	19	0	0			
7636	2020-3-24 8:00	MC	EURO	0	19	0	0			
7637	2020-3-25 8:00	MC	EURO	0	19	0	0			
7638	2020-3-26 8:00	MC	EURO	0	19	0	0			
7639	2020-3-27 8:00	MC	EURO	0	19	0	0			
7640	2020-3-28 8:00	MC	EURO	0	19	0	0			
7641	2020-3-29 8:00	MC	EURO	0	19	0	0			
7642	2020-3-30 8:00	MC	EURO	12	31	0	0			
7643	2020-3-31 8:00	MC	EURO	3	34	0	0			
7644	2020-4-1 8:00	MC	EURO	3	37	0	0			
7645	2020-4-2 8:00	MC	EURO	0	37	0	0			
7646	2020-4-3 8:00	MC	EURO	0	37	0	0			
7647	2020-4-4 8:00	MC	EURO	0	37	0	0			
7648	2020-4-5 8:00	MC	EURO	0	37	0	0			
7649	2020-4-6 8:00	MC	EURO	0	37	0	0			
7650	2020-4-7 8:00	MC	EURO	3	40	0	0			
7651	2020-4-8 8:00	MC	EURO	0	40	0	0			
7652	2020-4-9 8:00	MC	EURO	14	54	0	0			
7653	2020-4-10 8:00	MC	EURO	0	54	0	0			
7654	2020-4-11 8:00	MC	EURO	0	54	0	0			
7655	2020-4-12 8:00	MC	EURO	0	54	0	0			
7656	2020-4-13 8:00	MC	EURO	0	54	0	0			
7657	2020-4-14 8:00	MC	EURO	14	68	0	0			
7658	2020-4-15 8:00	MC	EURO	0	68	0	0			
7659	2020-4-16 8:00	MC	EURO	0	68	0	0			
7660	2020-4-17 8:00	MC	EURO	0	68	1	1	0	1.471	1.471
7661	2020-4-18 8:00	MC	EURO	0	68	0	1	1	1.471	1.471
7662	2020-4-19 8:00	MC	EURO	0	68	0	1	2	1.471	1.471
7663	2020-4-20 8:00	MC	EURO	0	68	0	1	3	1.471	1.471
7664	2020-4-21 8:00	MC	EURO	0	68	0	1	4	1.471	1.471
7665	2020-4-22 8:00	MC	EURO	0	68	0	1	5	1.471	1.471
7666	2020-4-23 8:00	MC	EURO	0	68	0	1	6	1.471	1.471
7667	2020-4-24 8:00	MC	EURO	0	68	0	1	7	1.471	1.471
7668	2020-4-25 8:00	MC	EURO	0	68	0	1	8	1.471	1.471

Table S1 Daily confirmed cases and daily deaths of COVID-19 in 214 nations from WHO

ID	Date and time	Country or region	Continent	Confirmed patients	Cumulative Confirmed patients	Daily dead patients	Daily cumulative dead patients	Lag days	Daily CFR	3DMA CFR
7669	2020-4-26 8:00	MC	EURO	0	68	0	1	9	1.471	1.471
7670	2020-4-27 8:00	MC	EURO	0	68	0	1	10	1.471	1.471
7671	2020-4-28 8:00	MC	EURO	0	68	0	1	11	1.471	1.471
7672	2020-4-29 8:00	MC	EURO	0	68	0	1	12	1.471	1.471
7673	2020-4-30 8:00	MC	EURO	0	68	0	1	13	1.471	1.471
7674	2020-5-1 8:00	MC	EURO	0	68	0	1	14	1.471	1.471
7675	2020-5-2 8:00	MC	EURO	0	68	0	1	15	1.471	1.471
7676										
7677	2020-3-10 8:00	MN	WPRO	1	1	0	0			
7678	2020-3-11 8:00	MN	WPRO	0	1	0	0			
7679	2020-3-12 8:00	MN	WPRO	0	1	0	0			
7680	2020-3-13 8:00	MN	WPRO	0	1	0	0			
7681	2020-3-14 8:00	MN	WPRO	0	1	0	0			
7682	2020-3-15 8:00	MN	WPRO	0	1	0	0			
7683	2020-3-16 8:00	MN	WPRO	3	4	0	0			
7684	2020-3-17 8:00	MN	WPRO	0	4	0	0			
7685	2020-3-18 8:00	MN	WPRO	1	5	0	0			
7686	2020-3-19 8:00	MN	WPRO	1	6	0	0			
7687	2020-3-20 8:00	MN	WPRO	0	6	0	0			
7688	2020-3-21 8:00	MN	WPRO	4	10	0	0			
7689	2020-3-22 8:00	MN	WPRO	0	10	0	0			
7690	2020-3-23 8:00	MN	WPRO	0	10	0	0			
7691	2020-3-24 8:00	MN	WPRO	0	10	0	0			
7692	2020-3-25 8:00	MN	WPRO	0	10	0	0			
7693	2020-3-26 8:00	MN	WPRO	1	11	0	0			
7694	2020-3-27 8:00	MN	WPRO	0	11	0	0			
7695	2020-3-28 8:00	MN	WPRO	1	12	0	0			
7696	2020-3-29 8:00	MN	WPRO	0	12	0	0			
7697	2020-3-30 8:00	MN	WPRO	0	12	0	0			
7698	2020-3-31 8:00	MN	WPRO	0	12	0	0			
7699	2020-4-1 8:00	MN	WPRO	2	14	0	0			
7700	2020-4-2 8:00	MN	WPRO	0	14	0	0			
7701	2020-4-3 8:00	MN	WPRO	0	14	0	0			
7702	2020-4-4 8:00	MN	WPRO	0	14	0	0			
7703	2020-4-5 8:00	MN	WPRO	0	14	0	0			
7704	2020-4-6 8:00	MN	WPRO	1	15	0	0			

Table S1 Daily confirmed cases and daily deaths of COVID-19 in 214 nations from WHO

ID	Date and time	Country or region	Continent	Confirmed patients	Cumulative Confirmed patients	Daily dead patients	Daily cumulative dead patients	Lag days	Daily CFR	3DMA CFR
7705	2020-4-7 8:00	MN	WPRO	0	15	0	0			
7706	2020-4-8 8:00	MN	WPRO	1	16	0	0			
7707	2020-4-9 8:00	MN	WPRO	0	16	0	0			
7708	2020-4-10 8:00	MN	WPRO	0	16	0	0			
7709	2020-4-11 8:00	MN	WPRO	0	16	0	0			
7710	2020-4-12 8:00	MN	WPRO	0	16	0	0			
7711	2020-4-13 8:00	MN	WPRO	0	16	0	0			
7712	2020-4-14 8:00	MN	WPRO	14	30	0	0			
7713	2020-4-15 8:00	MN	WPRO	0	30	0	0			
7714	2020-4-16 8:00	MN	WPRO	1	31	0	0			
7715	2020-4-17 8:00	MN	WPRO	0	31	0	0			
7716	2020-4-18 8:00	MN	WPRO	0	31	0	0			
7717	2020-4-19 8:00	MN	WPRO	1	32	0	0			
7718	2020-4-20 8:00	MN	WPRO	1	33	0	0			
7719	2020-4-21 8:00	MN	WPRO	1	34	0	0			
7720	2020-4-22 8:00	MN	WPRO	1	35	0	0			
7721	2020-4-23 8:00	MN	WPRO	0	35	0	0			
7722	2020-4-24 8:00	MN	WPRO	1	36	0	0			
7723	2020-4-25 8:00	MN	WPRO	0	36	0	0			
7724	2020-4-26 8:00	MN	WPRO	1	37	0	0			
7725	2020-4-27 8:00	MN	WPRO	1	38	0	0			
7726	2020-4-28 8:00	MN	WPRO	0	38	0	0			
7727	2020-4-29 8:00	MN	WPRO	0	38	0	0			
7728	2020-4-30 8:00	MN	WPRO	0	38	0	0			
7729	2020-5-1 8:00	MN	WPRO	0	38	0	0			
7730	2020-5-2 8:00	MN	WPRO	0	38	0	0			
7731										
7732	2020-3-17 8:00	ME	EURO	2	2	0	0			
7733	2020-3-18 8:00	ME	EURO	0	2	0	0			
7734	2020-3-19 8:00	ME	EURO	8	10	0	0			
7735	2020-3-20 8:00	ME	EURO	4	14	0	0			
7736	2020-3-21 8:00	ME	EURO	0	14	0	0			
7737	2020-3-22 8:00	ME	EURO	0	14	0	0			
7738	2020-3-23 8:00	ME	EURO	7	21	0	0			
7739	2020-3-24 8:00	ME	EURO	1	22	0	0			
7740	2020-3-25 8:00	ME	EURO	7	29	0	0			

Table S1 Daily confirmed cases and daily deaths of COVID-19 in 214 nations from WHO

ID	Date and time	Country or region	Continent	Confirmed patients	Cumulative Confirmed patients	Daily dead patients	Daily cumulative dead patients	Lag days	Daily CFR	3DMA CFR
7741	2020-3-26 8:00	ME	EURO	23	52	1	1	0	1.923	1.708
7742	2020-3-27 8:00	ME	EURO	15	67	0	1	1	1.493	1.615
7743	2020-3-28 8:00	ME	EURO	3	70	0	1	2	1.429	1.380
7744	2020-3-29 8:00	ME	EURO	12	82	0	1	3	1.220	1.275
7745	2020-3-30 8:00	ME	EURO	3	85	0	1	4	1.176	1.165
7746	2020-3-31 8:00	ME	EURO	6	91	0	1	5	1.099	1.393
7747	2020-4-1 8:00	ME	EURO	14	105	1	2	6	1.905	1.557
7748	2020-4-2 8:00	ME	EURO	15	120	0	2	7	1.667	1.667
7749	2020-4-3 8:00	ME	EURO	20	140	0	2	8	1.429	1.448
7750	2020-4-4 8:00	ME	EURO	20	160	0	2	9	1.250	1.231
7751	2020-4-5 8:00	ME	EURO	37	197	0	2	10	1.015	1.083
7752	2020-4-6 8:00	ME	EURO	6	203	0	2	11	0.985	0.966
7753	2020-4-7 8:00	ME	EURO	20	223	0	2	12	0.897	0.896
7754	2020-4-8 8:00	ME	EURO	25	248	0	2	13	0.806	0.837
7755	2020-4-9 8:00	ME	EURO	0	248	0	2	14	0.806	0.802
7756	2020-4-10 8:00	ME	EURO	4	252	0	2	15	0.794	0.795
7757	2020-4-11 8:00	ME	EURO	3	255	0	2	16	0.784	0.780
7758	2020-4-12 8:00	ME	EURO	7	262	0	2	17	0.763	0.766
7759	2020-4-13 8:00	ME	EURO	5	267	0	2	18	0.749	0.991
7760	2020-4-14 8:00	ME	EURO	7	274	2	4	19	1.460	1.199
7761	2020-4-15 8:00	ME	EURO	14	288	0	4	20	1.389	1.413
7762	2020-4-16 8:00	ME	EURO	0	288	0	4	21	1.389	1.366
7763	2020-4-17 8:00	ME	EURO	15	303	0	4	22	1.320	1.449
7764	2020-4-18 8:00	ME	EURO	2	305	1	5	23	1.639	1.528
7765	2020-4-19 8:00	ME	EURO	3	308	0	5	24	1.623	1.629
7766	2020-4-20 8:00	ME	EURO	0	308	0	5	25	1.623	1.616
7767	2020-4-21 8:00	ME	EURO	4	312	0	5	26	1.603	1.608
7768	2020-4-22 8:00	ME	EURO	1	313	0	5	27	1.597	1.597
7769	2020-4-23 8:00	ME	EURO	1	314	0	5	28	1.592	1.591
7770	2020-4-24 8:00	ME	EURO	2	316	0	5	29	1.582	1.581
7771	2020-4-25 8:00	ME	EURO	3	319	0	5	30	1.567	1.677
7772	2020-4-26 8:00	ME	EURO	0	319	1	6	31	1.881	1.876
7773	2020-4-27 8:00	ME	EURO	2	321	1	7	32	2.181	2.081
7774	2020-4-28 8:00	ME	EURO	0	321	0	7	33	2.181	2.181
7775	2020-4-29 8:00	ME	EURO	0	321	0	7	34	2.181	2.178
7776	2020-4-30 8:00	ME	EURO	1	322	0	7	35	2.174	2.176

Table S1 Daily confirmed cases and daily deaths of COVID-19 in 214 nations from WHO

ID	Date and time	Country or region	Continent	Confirmed patients	Cumulative Confirmed patients	Daily dead patients	Daily cumulative dead patients	Lag days	Daily CFR	3DMA CFR
7777	2020-5-1 8:00	ME	EURO	0	322	0	7	36	2.174	2.174
7778	2020-5-2 8:00	ME	EURO	0	322	0	7	37	2.174	2.174
7779										
7780	2020-3-18 8:00	MS	AMRO	1	1	0	0			
7781	2020-3-19 8:00	MS	AMRO	0	1	0	0			
7782	2020-3-20 8:00	MS	AMRO	0	1	0	0			
7783	2020-3-21 8:00	MS	AMRO	0	1	0	0			
7784	2020-3-22 8:00	MS	AMRO	0	1	0	0			
7785	2020-3-23 8:00	MS	AMRO	0	1	0	0			
7786	2020-3-24 8:00	MS	AMRO	0	1	0	0			
7787	2020-3-25 8:00	MS	AMRO	0	1	0	0			
7788	2020-3-26 8:00	MS	AMRO	1	2	0	0			
7789	2020-3-27 8:00	MS	AMRO	0	2	0	0			
7790	2020-3-28 8:00	MS	AMRO	3	5	0	0			
7791	2020-3-29 8:00	MS	AMRO	0	5	0	0			
7792	2020-3-30 8:00	MS	AMRO	0	5	0	0			
7793	2020-3-31 8:00	MS	AMRO	0	5	0	0			
7794	2020-4-1 8:00	MS	AMRO	0	5	0	0			
7795	2020-4-2 8:00	MS	AMRO	0	5	0	0			
7796	2020-4-3 8:00	MS	AMRO	0	5	0	0			
7797	2020-4-4 8:00	MS	AMRO	0	5	0	0			
7798	2020-4-5 8:00	MS	AMRO	1	6	0	0			
7799	2020-4-6 8:00	MS	AMRO	0	6	0	0			
7800	2020-4-7 8:00	MS	AMRO	0	6	0	0			
7801	2020-4-8 8:00	MS	AMRO	0	6	0	0			
7802	2020-4-9 8:00	MS	AMRO	2	8	0	0			
7803	2020-4-10 8:00	MS	AMRO	0	8	0	0			
7804	2020-4-11 8:00	MS	AMRO	1	9	0	0			
7805	2020-4-12 8:00	MS	AMRO	0	9	0	0			
7806	2020-4-13 8:00	MS	AMRO	0	9	0	0			
7807	2020-4-14 8:00	MS	AMRO	2	11	0	0			
7808	2020-4-15 8:00	MS	AMRO	0	11	0	0			
7809	2020-4-16 8:00	MS	AMRO	0	11	0	0			
7810	2020-4-17 8:00	MS	AMRO	0	11	0	0			
7811	2020-4-18 8:00	MS	AMRO	0	11	0	0			
7812	2020-4-19 8:00	MS	AMRO	0	11	0	0			

Table S1 Daily confirmed cases and daily deaths of COVID-19 in 214 nations from WHO

ID	Date and time	Country or region	Continent	Confirmed patients	Cumulative Confirmed patients	Daily dead patients	Daily cumulative dead patients	Lag days	Daily CFR	3DMA CFR
7813	2020-4-20 8:00	MS	AMRO	0	11	0	0			
7814	2020-4-21 8:00	MS	AMRO	0	11	0	0			
7815	2020-4-22 8:00	MS	AMRO	0	11	0	0			
7816	2020-4-23 8:00	MS	AMRO	0	11	0	0			
7817	2020-4-24 8:00	MS	AMRO	0	11	0	0			
7818	2020-4-25 8:00	MS	AMRO	0	11	0	0			
7819	2020-4-26 8:00	MS	AMRO	0	11	1	1	0	9.091	9.091
7820	2020-4-27 8:00	MS	AMRO	0	11	0	1	1	9.091	9.091
7821	2020-4-28 8:00	MS	AMRO	0	11	0	1	2	9.091	9.091
7822	2020-4-29 8:00	MS	AMRO	0	11	0	1	3	9.091	9.091
7823	2020-4-30 8:00	MS	AMRO	0	11	0	1	4	9.091	9.091
7824	2020-5-1 8:00	MS	AMRO	0	11	0	1	5	9.091	9.091
7825	2020-5-2 8:00	MS	AMRO	0	11	0	1	6	9.091	9.091
7826										
7827	2020-3-2 8:00	MA	EMRO	1	1	0	0			
7828	2020-3-3 8:00	MA	EMRO	0	1	0	0			
7829	2020-3-4 8:00	MA	EMRO	0	1	0	0			
7830	2020-3-5 8:00	MA	EMRO	1	2	0	0			
7831	2020-3-6 8:00	MA	EMRO	0	2	0	0			
7832	2020-3-7 8:00	MA	EMRO	0	2	0	0			
7833	2020-3-8 8:00	MA	EMRO	0	2	0	0			
7834	2020-3-9 8:00	MA	EMRO	0	2	0	0			
7835	2020-3-10 8:00	MA	EMRO	1	3	1	1	0	33.333	26.667
7836	2020-3-11 8:00	MA	EMRO	2	5	0	1	1	20.000	23.333
7837	2020-3-12 8:00	MA	EMRO	1	6	0	1	2	16.667	17.778
7838	2020-3-13 8:00	MA	EMRO	0	6	0	1	3	16.667	12.963
7839	2020-3-14 8:00	MA	EMRO	12	18	0	1	4	5.556	8.598
7840	2020-3-15 8:00	MA	EMRO	10	28	0	1	5	3.571	4.192
7841	2020-3-16 8:00	MA	EMRO	1	29	0	1	6	3.448	4.094
7842	2020-3-17 8:00	MA	EMRO	9	38	1	2	7	5.263	4.264
7843	2020-3-18 8:00	MA	EMRO	11	49	0	2	8	4.082	4.208
7844	2020-3-19 8:00	MA	EMRO	12	61	0	2	9	3.279	3.805
7845	2020-3-20 8:00	MA	EMRO	13	74	1	3	10	4.054	3.607
7846	2020-3-21 8:00	MA	EMRO	12	86	0	3	11	3.488	3.432
7847	2020-3-22 8:00	MA	EMRO	23	109	0	3	12	2.752	3.075
7848	2020-3-23 8:00	MA	EMRO	25	134	1	4	13	2.985	2.845

Table S1 Daily confirmed cases and daily deaths of COVID-19 in 214 nations from WHO

ID	Date and time	Country or region	Continent	Confirmed patients	Cumulative Confirmed patients	Daily dead patients	Daily cumulative dead patients	Lag days	Daily CFR	3DMA CFR
7849	2020-3-24 8:00	MA	EMRO	9	143	0	4	14	2.797	2.908
7850	2020-3-25 8:00	MA	EMRO	27	170	1	5	15	2.941	2.802
7851	2020-3-26 8:00	MA	EMRO	55	225	1	6	16	2.667	3.081
7852	2020-3-27 8:00	MA	EMRO	50	275	4	10	17	3.636	4.243
7853	2020-3-28 8:00	MA	EMRO	83	358	13	23	18	6.425	5.280
7854	2020-3-29 8:00	MA	EMRO	92	450	3	26	19	5.778	5.941
7855	2020-3-30 8:00	MA	EMRO	66	516	3	29	20	5.620	5.716
7856	2020-3-31 8:00	MA	EMRO	58	574	4	33	21	5.749	5.723
7857	2020-4-1 8:00	MA	EMRO	64	638	4	37	22	5.799	5.822
7858	2020-4-2 8:00	MA	EMRO	38	676	3	40	23	5.917	6.037
7859	2020-4-3 8:00	MA	EMRO	59	735	7	47	24	6.395	6.046
7860	2020-4-4 8:00	MA	EMRO	123	858	3	50	25	5.828	6.366
7861	2020-4-5 8:00	MA	EMRO	102	960	16	66	26	6.875	6.361
7862	2020-4-6 8:00	MA	EMRO	153	1113	5	71	27	6.379	6.843
7863	2020-4-7 8:00	MA	EMRO	28	1141	12	83	28	7.274	6.993
7864	2020-4-8 8:00	MA	EMRO	101	1242	8	91	29	7.327	7.244
7865	2020-4-9 8:00	MA	EMRO	104	1346	5	96	30	7.132	7.266
7866	2020-4-10 8:00	MA	EMRO	85	1431	9	105	31	7.338	7.224
7867	2020-4-11 8:00	MA	EMRO	96	1527	5	110	32	7.204	7.176
7868	2020-4-12 8:00	MA	EMRO	90	1617	3	113	33	6.988	7.022
7869	2020-4-13 8:00	MA	EMRO	129	1746	7	120	34	6.873	6.905
7870	2020-4-14 8:00	MA	EMRO	92	1838	6	126	35	6.855	6.705
7871	2020-4-15 8:00	MA	EMRO	150	1988	1	127	36	6.388	6.310
7872	2020-4-16 8:00	MA	EMRO	263	2251	1	128	37	5.686	5.779
7873	2020-4-17 8:00	MA	EMRO	277	2528	5	133	38	5.261	5.360
7874	2020-4-18 8:00	MA	EMRO	142	2670	4	137	39	5.131	5.095
7875	2020-4-19 8:00	MA	EMRO	150	2820	1	138	40	4.894	4.936
7876	2020-4-20 8:00	MA	EMRO	170	2990	5	143	41	4.783	4.732
7877	2020-4-21 8:00	MA	EMRO	196	3186	1	144	42	4.520	4.572
7878	2020-4-22 8:00	MA	EMRO	191	3377	5	149	43	4.412	4.400
7879	2020-4-23 8:00	MA	EMRO	160	3537	2	151	44	4.269	4.293
7880	2020-4-24 8:00	MA	EMRO	155	3692	4	155	45	4.198	4.185
7881	2020-4-25 8:00	MA	EMRO	197	3889	4	159	46	4.088	4.080
7882	2020-4-26 8:00	MA	EMRO	158	4047	1	160	47	3.954	3.985
7883	2020-4-27 8:00	MA	EMRO	68	4115	1	161	48	3.913	3.902
7884	2020-4-28 8:00	MA	EMRO	131	4246	2	163	49	3.839	3.882

Table S1 Daily confirmed cases and daily deaths of COVID-19 in 214 nations from WHO

ID	Date and time	Country or region	Continent	Confirmed patients	Cumulative Confirmed patients	Daily dead patients	Daily cumulative dead patients	Lag days	Daily CFR	3DMA CFR
7885	2020-4-29 8:00	MA	EMRO	43	4289	4	167	50	3.894	3.862
7886	2020-4-30 8:00	MA	EMRO	70	4359	1	168	51	3.854	3.841
7887	2020-5-1 8:00	MA	EMRO	170	4529	3	171	52	3.776	3.791
7888	2020-5-2 8:00	MA	EMRO	40	4569	0	171	53	3.743	3.759
7889										
7890	2020-3-23 8:00	MZ	AFRO	1	1	0	0			
7891	2020-3-24 8:00	MZ	AFRO	0	1	0	0			
7892	2020-3-25 8:00	MZ	AFRO	2	3	0	0			
7893	2020-3-26 8:00	MZ	AFRO	2	5	0	0			
7894	2020-3-27 8:00	MZ	AFRO	0	5	0	0			
7895	2020-3-28 8:00	MZ	AFRO	2	7	0	0			
7896	2020-3-29 8:00	MZ	AFRO	1	8	0	0			
7897	2020-3-30 8:00	MZ	AFRO	0	8	0	0			
7898	2020-3-31 8:00	MZ	AFRO	0	8	0	0			
7899	2020-4-1 8:00	MZ	AFRO	0	8	0	0			
7900	2020-4-2 8:00	MZ	AFRO	2	10	0	0			
7901	2020-4-3 8:00	MZ	AFRO	0	10	0	0			
7902	2020-4-4 8:00	MZ	AFRO	0	10	0	0			
7903	2020-4-5 8:00	MZ	AFRO	0	10	0	0			
7904	2020-4-6 8:00	MZ	AFRO	0	10	0	0			
7905	2020-4-7 8:00	MZ	AFRO	0	10	0	0			
7906	2020-4-8 8:00	MZ	AFRO	0	10	0	0			
7907	2020-4-9 8:00	MZ	AFRO	7	17	0	0			
7908	2020-4-10 8:00	MZ	AFRO	0	17	0	0			
7909	2020-4-11 8:00	MZ	AFRO	3	20	0	0			
7910	2020-4-12 8:00	MZ	AFRO	0	20	0	0			
7911	2020-4-13 8:00	MZ	AFRO	0	20	0	0			
7912	2020-4-14 8:00	MZ	AFRO	1	21	0	0			
7913	2020-4-15 8:00	MZ	AFRO	7	28	0	0			
7914	2020-4-16 8:00	MZ	AFRO	0	28	0	0			
7915	2020-4-17 8:00	MZ	AFRO	1	29	0	0			
7916	2020-4-18 8:00	MZ	AFRO	2	31	0	0			
7917	2020-4-19 8:00	MZ	AFRO	0	31	0	0			
7918	2020-4-20 8:00	MZ	AFRO	4	35	0	0			
7919	2020-4-21 8:00	MZ	AFRO	0	35	0	0			
7920	2020-4-22 8:00	MZ	AFRO	4	39	0	0			

Table S1 Daily confirmed cases and daily deaths of COVID-19 in 214 nations from WHO

ID	Date and time	Country or region	Continent	Confirmed patients	Cumulative Confirmed patients	Daily dead patients	Daily cumulative dead patients	Lag days	Daily CFR	3DMA CFR
7921	2020-4-23 8:00	MZ	AFRO	2	41	0	0			
7922	2020-4-24 8:00	MZ	AFRO	0	41	0	0			
7923	2020-4-25 8:00	MZ	AFRO	24	65	0	0			
7924	2020-4-26 8:00	MZ	AFRO	5	70	0	0			
7925	2020-4-27 8:00	MZ	AFRO	6	76	0	0			
7926	2020-4-28 8:00	MZ	AFRO	0	76	0	0			
7927	2020-4-29 8:00	MZ	AFRO	0	76	0	0			
7928	2020-4-30 8:00	MZ	AFRO	0	76	0	0			
7929	2020-5-1 8:00	MZ	AFRO	0	76	0	0			
7930	2020-5-2 8:00	MZ	AFRO	3	79	0	0			
7931										
7932	2020-3-24 8:00	MM	SEARO	2	2	0	0			
7933	2020-3-25 8:00	MM	SEARO	1	3	0	0			
7934	2020-3-26 8:00	MM	SEARO	0	3	0	0			
7935	2020-3-27 8:00	MM	SEARO	2	5	0	0			
7936	2020-3-28 8:00	MM	SEARO	0	5	0	0			
7937	2020-3-29 8:00	MM	SEARO	3	8	0	0			
7938	2020-3-30 8:00	MM	SEARO	2	10	0	0			
7939	2020-3-31 8:00	MM	SEARO	4	14	0	0			
7940	2020-4-1 8:00	MM	SEARO	1	15	1	1	0	6.667	6.458
7941	2020-4-2 8:00	MM	SEARO	1	16	0	1	1	6.250	5.972
7942	2020-4-3 8:00	MM	SEARO	4	20	0	1	2	5.000	5.417
7943	2020-4-4 8:00	MM	SEARO	0	20	0	1	3	5.000	4.921
7944	2020-4-5 8:00	MM	SEARO	1	21	0	1	4	4.762	4.841
7945	2020-4-6 8:00	MM	SEARO	0	21	0	1	5	4.762	4.690
7946	2020-4-7 8:00	MM	SEARO	1	22	0	1	6	4.545	7.648
7947	2020-4-8 8:00	MM	SEARO	0	22	2	3	7	13.636	10.606
7948	2020-4-9 8:00	MM	SEARO	0	22	0	3	8	13.636	12.795
7949	2020-4-10 8:00	MM	SEARO	5	27	0	3	9	11.111	11.821
7950	2020-4-11 8:00	MM	SEARO	1	28	0	3	10	10.714	9.907
7951	2020-4-12 8:00	MM	SEARO	10	38	0	3	11	7.895	9.455
7952	2020-4-13 8:00	MM	SEARO	3	41	1	4	12	9.756	8.034
7953	2020-4-14 8:00	MM	SEARO	21	62	0	4	13	6.452	7.204
7954	2020-4-15 8:00	MM	SEARO	12	74	0	4	14	5.405	5.521
7955	2020-4-16 8:00	MM	SEARO	11	85	0	4	15	4.706	4.939
7956	2020-4-17 8:00	MM	SEARO	0	85	0	4	16	4.706	4.910

Table S1 Daily confirmed cases and daily deaths of COVID-19 in 214 nations from WHO

ID	Date and time	Country or region	Continent	Confirmed patients	Cumulative Confirmed patients	Daily dead patients	Daily cumulative dead patients	Lag days	Daily CFR	3DMA CFR
7957	2020-4-18 8:00	MM	SEARO	9	94	1	5	17	5.319	4.899
7958	2020-4-19 8:00	MM	SEARO	13	107	0	5	18	4.673	4.832
7959	2020-4-20 8:00	MM	SEARO	4	111	0	5	19	4.505	4.460
7960	2020-4-21 8:00	MM	SEARO	8	119	0	5	20	4.202	4.279
7961	2020-4-22 8:00	MM	SEARO	2	121	0	5	21	4.132	4.090
7962	2020-4-23 8:00	MM	SEARO	6	127	0	5	22	3.937	3.889
7963	2020-4-24 8:00	MM	SEARO	12	139	0	5	23	3.597	3.669
7964	2020-4-25 8:00	MM	SEARO	5	144	0	5	24	3.472	3.498
7965	2020-4-26 8:00	MM	SEARO	2	146	0	5	25	3.425	3.441
7966	2020-4-27 8:00	MM	SEARO	0	146	0	5	26	3.425	3.425
7967	2020-4-28 8:00	MM	SEARO	0	146	0	5	27	3.425	3.394
7968	2020-4-29 8:00	MM	SEARO	4	150	0	5	28	3.333	3.586
7969	2020-4-30 8:00	MM	SEARO	0	150	1	6	29	4.000	3.769
7970	2020-5-1 8:00	MM	SEARO	1	151	0	6	30	3.974	3.982
7971	2020-5-2 8:00	MM	SEARO	0	151	0	6	31	3.974	3.974
7972										
7973	2020-3-14 8:00	NA	AFRO	2	2	0	0			
7974	2020-3-15 8:00	NA	AFRO	0	2	0	0			
7975	2020-3-16 8:00	NA	AFRO	0	2	0	0			
7976	2020-3-17 8:00	NA	AFRO	0	2	0	0			
7977	2020-3-18 8:00	NA	AFRO	0	2	0	0			
7978	2020-3-19 8:00	NA	AFRO	1	3	0	0			
7979	2020-3-20 8:00	NA	AFRO	0	3	0	0			
7980	2020-3-21 8:00	NA	AFRO	0	3	0	0			
7981	2020-3-22 8:00	NA	AFRO	0	3	0	0			
7982	2020-3-23 8:00	NA	AFRO	0	3	0	0			
7983	2020-3-24 8:00	NA	AFRO	0	3	0	0			
7984	2020-3-25 8:00	NA	AFRO	1	4	0	0			
7985	2020-3-26 8:00	NA	AFRO	4	8	0	0			
7986	2020-3-27 8:00	NA	AFRO	0	8	0	0			
7987	2020-3-28 8:00	NA	AFRO	0	8	0	0			
7988	2020-3-29 8:00	NA	AFRO	3	11	0	0			
7989	2020-3-30 8:00	NA	AFRO	0	11	0	0			
7990	2020-3-31 8:00	NA	AFRO	0	11	0	0			
7991	2020-4-1 8:00	NA	AFRO	0	11	0	0			
7992	2020-4-2 8:00	NA	AFRO	2	13	0	0			

Table S1 Daily confirmed cases and daily deaths of COVID-19 in 214 nations from WHO

ID	Date and time	Country or region	Continent	Confirmed patients	Cumulative Confirmed patients	Daily dead patients	Daily cumulative dead patients	Lag days	Daily CFR	3DMA CFR
7993	2020-4-3 8:00	NA	AFRO	0	13	0	0			
7994	2020-4-4 8:00	NA	AFRO	0	13	0	0			
7995	2020-4-5 8:00	NA	AFRO	1	14	0	0			
7996	2020-4-6 8:00	NA	AFRO	2	16	0	0			
7997	2020-4-7 8:00	NA	AFRO	0	16	0	0			
7998	2020-4-8 8:00	NA	AFRO	0	16	0	0			
7999	2020-4-9 8:00	NA	AFRO	0	16	0	0			
8000	2020-4-10 8:00	NA	AFRO	0	16	0	0			
8001	2020-4-11 8:00	NA	AFRO	0	16	0	0			
8002	2020-4-12 8:00	NA	AFRO	0	16	0	0			
8003	2020-4-13 8:00	NA	AFRO	0	16	0	0			
8004	2020-4-14 8:00	NA	AFRO	0	16	0	0			
8005	2020-4-15 8:00	NA	AFRO	0	16	0	0			
8006	2020-4-16 8:00	NA	AFRO	0	16	0	0			
8007	2020-4-17 8:00	NA	AFRO	0	16	0	0			
8008	2020-4-18 8:00	NA	AFRO	0	16	0	0			
8009	2020-4-19 8:00	NA	AFRO	0	16	0	0			
8010	2020-4-20 8:00	NA	AFRO	0	16	0	0			
8011	2020-4-21 8:00	NA	AFRO	0	16	0	0			
8012	2020-4-22 8:00	NA	AFRO	0	16	0	0			
8013	2020-4-23 8:00	NA	AFRO	0	16	0	0			
8014	2020-4-24 8:00	NA	AFRO	0	16	0	0			
8015	2020-4-25 8:00	NA	AFRO	0	16	0	0			
8016	2020-4-26 8:00	NA	AFRO	0	16	0	0			
8017	2020-4-27 8:00	NA	AFRO	0	16	0	0			
8018	2020-4-28 8:00	NA	AFRO	0	16	0	0			
8019	2020-4-29 8:00	NA	AFRO	0	16	0	0			
8020	2020-4-30 8:00	NA	AFRO	0	16	0	0			
8021	2020-5-1 8:00	NA	AFRO	0	16	0	0			
8022	2020-5-2 8:00	NA	AFRO	0	16	0	0			
8023										
8024	2020-1-23 8:00	NP	SEARO	1	1	0	0			
8025	2020-1-24 8:00	NP	SEARO	0	1	0	0			
8026	2020-1-25 8:00	NP	SEARO	0	1	0	0			
8027	2020-1-26 8:00	NP	SEARO	0	1	0	0			
8028	2020-1-27 8:00	NP	SEARO	0	1	0	0			

Table S1 Daily confirmed cases and daily deaths of COVID-19 in 214 nations from WHO

ID	Date and time	Country or region	Continent	Confirmed patients	Cumulative Confirmed patients	Daily dead patients	Daily cumulative dead patients	Lag days	Daily CFR	3DMA CFR
8029	2020-1-28 8:00	NP	SEARO	0	1	0	0			
8030	2020-1-29 8:00	NP	SEARO	0	1	0	0			
8031	2020-1-30 8:00	NP	SEARO	0	1	0	0			
8032	2020-1-31 8:00	NP	SEARO	0	1	0	0			
8033	2020-2-1 8:00	NP	SEARO	0	1	0	0			
8034	2020-2-2 8:00	NP	SEARO	0	1	0	0			
8035	2020-2-3 8:00	NP	SEARO	0	1	0	0			
8036	2020-2-4 8:00	NP	SEARO	0	1	0	0			
8037	2020-2-5 8:00	NP	SEARO	0	1	0	0			
8038	2020-2-6 8:00	NP	SEARO	0	1	0	0			
8039	2020-2-7 8:00	NP	SEARO	0	1	0	0			
8040	2020-2-8 8:00	NP	SEARO	0	1	0	0			
8041	2020-2-9 8:00	NP	SEARO	0	1	0	0			
8042	2020-2-10 8:00	NP	SEARO	0	1	0	0			
8043	2020-2-11 8:00	NP	SEARO	0	1	0	0			
8044	2020-2-12 8:00	NP	SEARO	0	1	0	0			
8045	2020-2-13 8:00	NP	SEARO	0	1	0	0			
8046	2020-2-14 8:00	NP	SEARO	0	1	0	0			
8047	2020-2-15 8:00	NP	SEARO	0	1	0	0			
8048	2020-2-16 8:00	NP	SEARO	0	1	0	0			
8049	2020-2-17 8:00	NP	SEARO	0	1	0	0			
8050	2020-2-18 8:00	NP	SEARO	0	1	0	0			
8051	2020-2-19 8:00	NP	SEARO	0	1	0	0			
8052	2020-2-20 8:00	NP	SEARO	0	1	0	0			
8053	2020-2-21 8:00	NP	SEARO	0	1	0	0			
8054	2020-2-22 8:00	NP	SEARO	0	1	0	0			
8055	2020-2-23 8:00	NP	SEARO	0	1	0	0			
8056	2020-2-24 8:00	NP	SEARO	0	1	0	0			
8057	2020-2-25 8:00	NP	SEARO	0	1	0	0			
8058	2020-2-26 8:00	NP	SEARO	0	1	0	0			
8059	2020-2-27 8:00	NP	SEARO	0	1	0	0			
8060	2020-2-28 8:00	NP	SEARO	0	1	0	0			
8061	2020-2-29 8:00	NP	SEARO	0	1	0	0			
8062	2020-3-1 8:00	NP	SEARO	0	1	0	0			
8063	2020-3-2 8:00	NP	SEARO	0	1	0	0			
8064	2020-3-3 8:00	NP	SEARO	0	1	0	0			

Table S1 Daily confirmed cases and daily deaths of COVID-19 in 214 nations from WHO

ID	Date and time	Country or region	Continent	Confirmed patients	Cumulative Confirmed patients	Daily dead patients	Daily cumulative dead patients	Lag days	Daily CFR	3DMA CFR
8065	2020-3-4 8:00	NP	SEARO	0	1	0	0			
8066	2020-3-5 8:00	NP	SEARO	0	1	0	0			
8067	2020-3-6 8:00	NP	SEARO	0	1	0	0			
8068	2020-3-7 8:00	NP	SEARO	0	1	0	0			
8069	2020-3-8 8:00	NP	SEARO	0	1	0	0			
8070	2020-3-9 8:00	NP	SEARO	0	1	0	0			
8071	2020-3-10 8:00	NP	SEARO	0	1	0	0			
8072	2020-3-11 8:00	NP	SEARO	0	1	0	0			
8073	2020-3-12 8:00	NP	SEARO	0	1	0	0			
8074	2020-3-13 8:00	NP	SEARO	0	1	0	0			
8075	2020-3-14 8:00	NP	SEARO	0	1	0	0			
8076	2020-3-15 8:00	NP	SEARO	0	1	0	0			
8077	2020-3-16 8:00	NP	SEARO	0	1	0	0			
8078	2020-3-17 8:00	NP	SEARO	0	1	0	0			
8079	2020-3-18 8:00	NP	SEARO	0	1	0	0			
8080	2020-3-19 8:00	NP	SEARO	0	1	0	0			
8081	2020-3-20 8:00	NP	SEARO	0	1	0	0			
8082	2020-3-21 8:00	NP	SEARO	0	1	0	0			
8083	2020-3-22 8:00	NP	SEARO	0	1	0	0			
8084	2020-3-23 8:00	NP	SEARO	0	1	0	0			
8085	2020-3-24 8:00	NP	SEARO	1	2	0	0			
8086	2020-3-25 8:00	NP	SEARO	0	2	0	0			
8087	2020-3-26 8:00	NP	SEARO	1	3	0	0			
8088	2020-3-27 8:00	NP	SEARO	0	3	0	0			
8089	2020-3-28 8:00	NP	SEARO	0	3	0	0			
8090	2020-3-29 8:00	NP	SEARO	2	5	0	0			
8091	2020-3-30 8:00	NP	SEARO	0	5	0	0			
8092	2020-3-31 8:00	NP	SEARO	0	5	0	0			
8093	2020-4-1 8:00	NP	SEARO	0	5	0	0			
8094	2020-4-2 8:00	NP	SEARO	0	5	0	0			
8095	2020-4-3 8:00	NP	SEARO	1	6	0	0			
8096	2020-4-4 8:00	NP	SEARO	0	6	0	0			
8097	2020-4-5 8:00	NP	SEARO	3	9	0	0			
8098	2020-4-6 8:00	NP	SEARO	0	9	0	0			
8099	2020-4-7 8:00	NP	SEARO	0	9	0	0			
8100	2020-4-8 8:00	NP	SEARO	0	9	0	0			

Table S1 Daily confirmed cases and daily deaths of COVID-19 in 214 nations from WHO

ID	Date and time	Country or region	Continent	Confirmed patients	Cumulative Confirmed patients	Daily dead patients	Daily cumulative dead patients	Lag days	Daily CFR	3DMA CFR
8101	2020-4-9 8:00	NP	SEARO	0	9	0	0			
8102	2020-4-10 8:00	NP	SEARO	0	9	0	0			
8103	2020-4-11 8:00	NP	SEARO	0	9	0	0			
8104	2020-4-12 8:00	NP	SEARO	3	12	0	0			
8105	2020-4-13 8:00	NP	SEARO	2	14	0	0			
8106	2020-4-14 8:00	NP	SEARO	2	16	0	0			
8107	2020-4-15 8:00	NP	SEARO	0	16	0	0			
8108	2020-4-16 8:00	NP	SEARO	0	16	0	0			
8109	2020-4-17 8:00	NP	SEARO	0	16	0	0			
8110	2020-4-18 8:00	NP	SEARO	14	30	0	0			
8111	2020-4-19 8:00	NP	SEARO	1	31	0	0			
8112	2020-4-20 8:00	NP	SEARO	0	31	0	0			
8113	2020-4-21 8:00	NP	SEARO	1	32	0	0			
8114	2020-4-22 8:00	NP	SEARO	10	42	0	0			
8115	2020-4-23 8:00	NP	SEARO	3	45	0	0			
8116	2020-4-24 8:00	NP	SEARO	3	48	0	0			
8117	2020-4-25 8:00	NP	SEARO	1	49	0	0			
8118	2020-4-26 8:00	NP	SEARO	2	51	0	0			
8119	2020-4-27 8:00	NP	SEARO	1	52	0	0			
8120	2020-4-28 8:00	NP	SEARO	0	52	0	0			
8121	2020-4-29 8:00	NP	SEARO	5	57	0	0			
8122	2020-4-30 8:00	NP	SEARO	0	57	0	0			
8123	2020-5-1 8:00	NP	SEARO	2	59	0	0			
8124	2020-5-2 8:00	NP	SEARO	0	59	0	0			
8125										
8126	2020-2-27 8:00	NL	EURO	1	1	0	0			
8127	2020-2-28 8:00	NL	EURO	1	2	0	0			
8128	2020-2-29 8:00	NL	EURO	4	6	0	0			
8129	2020-3-1 8:00	NL	EURO	7	13	0	0			
8130	2020-3-2 8:00	NL	EURO	5	18	0	0			
8131	2020-3-3 8:00	NL	EURO	10	28	0	0			
8132	2020-3-4 8:00	NL	EURO	10	38	0	0			
8133	2020-3-5 8:00	NL	EURO	44	82	0	0			
8134	2020-3-6 8:00	NL	EURO	46	128	1	1	0	0.781	0.657
8135	2020-3-7 8:00	NL	EURO	60	188	0	1	1	0.532	0.815
8136	2020-3-8 8:00	NL	EURO	77	265	2	3	2	1.132	0.932

Table S1 Daily confirmed cases and daily deaths of COVID-19 in 214 nations from WHO

ID	Date and time	Country or region	Continent	Confirmed patients	Cumulative Confirmed patients	Daily dead patients	Daily cumulative dead patients	Lag days	Daily CFR	3DMA CFR
8137	2020-3-9 8:00	NL	EURO	0	265	0	3	3	1.132	1.104
8138	2020-3-10 8:00	NL	EURO	117	382	1	4	4	1.047	1.058
8139	2020-3-11 8:00	NL	EURO	121	503	1	5	5	0.994	0.952
8140	2020-3-12 8:00	NL	EURO	111	614	0	5	6	0.814	1.017
8141	2020-3-13 8:00	NL	EURO	190	804	5	10	7	1.244	1.103
8142	2020-3-14 8:00	NL	EURO	155	959	2	12	8	1.251	1.419
8143	2020-3-15 8:00	NL	EURO	176	1135	8	20	9	1.762	1.571
8144	2020-3-16 8:00	NL	EURO	278	1413	4	24	10	1.699	2.096
8145	2020-3-17 8:00	NL	EURO	638	2051	34	58	11	2.828	2.451
8146	2020-3-18 8:00	NL	EURO	0	2051	0	58	12	2.828	2.915
8147	2020-3-19 8:00	NL	EURO	409	2460	18	76	13	3.089	3.153
8148	2020-3-20 8:00	NL	EURO	534	2994	30	106	14	3.540	3.458
8149	2020-3-21 8:00	NL	EURO	637	3631	30	136	15	3.746	3.677
8150	2020-3-22 8:00	NL	EURO	0	3631	0	136	16	3.746	3.916
8151	2020-3-23 8:00	NL	EURO	573	4204	43	179	17	4.258	4.163
8152	2020-3-24 8:00	NL	EURO	545	4749	34	213	18	4.485	4.569
8153	2020-3-25 8:00	NL	EURO	811	5560	63	276	19	4.964	5.000
8154	2020-3-26 8:00	NL	EURO	852	6412	80	356	20	5.552	5.452
8155	2020-3-27 8:00	NL	EURO	1019	7431	78	434	21	5.840	5.913
8156	2020-3-28 8:00	NL	EURO	1172	8603	112	546	22	6.347	6.244
8157	2020-3-29 8:00	NL	EURO	1159	9762	93	639	23	6.546	6.663
8158	2020-3-30 8:00	NL	EURO	1104	10866	132	771	24	7.096	6.998
8159	2020-3-31 8:00	NL	EURO	884	11750	93	864	25	7.353	7.566
8160	2020-4-1 8:00	NL	EURO	845	12595	175	1039	26	8.249	8.073
8161	2020-4-2 8:00	NL	EURO	1019	13614	134	1173	27	8.616	8.659
8162	2020-4-3 8:00	NL	EURO	1083	14697	166	1339	28	9.111	9.061
8163	2020-4-4 8:00	NL	EURO	1026	15723	148	1487	29	9.457	9.499
8164	2020-4-5 8:00	NL	EURO	904	16627	164	1651	30	9.930	9.760
8165	2020-4-6 8:00	NL	EURO	1224	17851	115	1766	31	9.893	9.917
8166	2020-4-7 8:00	NL	EURO	952	18803	101	1867	32	9.929	10.184
8167	2020-4-8 8:00	NL	EURO	777	19580	234	2101	33	10.730	10.533
8168	2020-4-9 8:00	NL	EURO	969	20549	147	2248	34	10.940	10.893
8169	2020-4-10 8:00	NL	EURO	1213	21762	148	2396	35	11.010	10.940
8170	2020-4-11 8:00	NL	EURO	1335	23097	115	2511	36	10.872	10.903
8171	2020-4-12 8:00	NL	EURO	1316	24413	132	2643	37	10.826	10.798
8172	2020-4-13 8:00	NL	EURO	1174	25587	94	2737	38	10.697	10.718

Table S1 Daily confirmed cases and daily deaths of COVID-19 in 214 nations from WHO

ID	Date and time	Country or region	Continent	Confirmed patients	Cumulative Confirmed patients	Daily dead patients	Daily cumulative dead patients	Lag days	Daily CFR	3DMA CFR
8173	2020-4-14 8:00	NL	EURO	964	26551	86	2823	39	10.632	10.690
8174	2020-4-15 8:00	NL	EURO	868	27419	122	2945	40	10.741	10.835
8175	2020-4-16 8:00	NL	EURO	734	28153	189	3134	41	11.132	11.073
8176	2020-4-17 8:00	NL	EURO	1061	29214	181	3315	42	11.347	11.280
8177	2020-4-18 8:00	NL	EURO	1235	30449	144	3459	43	11.360	11.369
8178	2020-4-19 8:00	NL	EURO	1140	31589	142	3601	44	11.400	11.347
8179	2020-4-20 8:00	NL	EURO	1066	32655	83	3684	45	11.282	11.303
8180	2020-4-21 8:00	NL	EURO	750	33405	67	3751	46	11.229	11.328
8181	2020-4-22 8:00	NL	EURO	729	34134	165	3916	47	11.472	11.446
8182	2020-4-23 8:00	NL	EURO	708	34842	138	4054	48	11.635	11.600
8183	2020-4-24 8:00	NL	EURO	887	35729	123	4177	49	11.691	11.689
8184	2020-4-25 8:00	NL	EURO	806	36535	112	4289	50	11.739	11.762
8185	2020-4-26 8:00	NL	EURO	655	37190	120	4409	51	11.855	11.806
8186	2020-4-27 8:00	NL	EURO	655	37845	66	4475	52	11.825	11.831
8187	2020-4-28 8:00	NL	EURO	400	38245	43	4518	53	11.813	11.841
8188	2020-4-29 8:00	NL	EURO	171	38416	48	4566	54	11.886	11.947
8189	2020-4-30 8:00	NL	EURO	386	38802	145	4711	55	12.141	12.074
8190	2020-5-1 8:00	NL	EURO	514	39316	84	4795	56	12.196	12.211
8191	2020-5-2 8:00	NL	EURO	475	39791	98	4893	57	12.297	12.246
8192										
8193	2020-3-19 8:00	NC	WPRO	2	2	0	0			
8194	2020-3-20 8:00	NC	WPRO	0	2	0	0			
8195	2020-3-21 8:00	NC	WPRO	2	4	0	0			
8196	2020-3-22 8:00	NC	WPRO	0	4	0	0			
8197	2020-3-23 8:00	NC	WPRO	3	7	0	0			
8198	2020-3-24 8:00	NC	WPRO	2	9	0	0			
8199	2020-3-25 8:00	NC	WPRO	3	12	0	0			
8200	2020-3-26 8:00	NC	WPRO	2	14	0	0			
8201	2020-3-27 8:00	NC	WPRO	0	14	0	0			
8202	2020-3-28 8:00	NC	WPRO	1	15	0	0			
8203	2020-3-29 8:00	NC	WPRO	0	15	0	0			
8204	2020-3-30 8:00	NC	WPRO	0	15	0	0			
8205	2020-3-31 8:00	NC	WPRO	0	15	0	0			
8206	2020-4-1 8:00	NC	WPRO	1	16	0	0			
8207	2020-4-2 8:00	NC	WPRO	1	17	0	0			
8208	2020-4-3 8:00	NC	WPRO	1	18	0	0			

Table S1 Daily confirmed cases and daily deaths of COVID-19 in 214 nations from WHO

ID	Date and time	Country or region	Continent	Confirmed patients	Cumulative Confirmed patients	Daily dead patients	Daily cumulative dead patients	Lag days	Daily CFR	3DMA CFR
8209	2020-4-4 8:00	NC	WPRO	0	18	0	0			
8210	2020-4-5 8:00	NC	WPRO	0	18	0	0			
8211	2020-4-6 8:00	NC	WPRO	0	18	0	0			
8212	2020-4-7 8:00	NC	WPRO	0	18	0	0			
8213	2020-4-8 8:00	NC	WPRO	0	18	0	0			
8214	2020-4-9 8:00	NC	WPRO	0	18	0	0			
8215	2020-4-10 8:00	NC	WPRO	0	18	0	0			
8216	2020-4-11 8:00	NC	WPRO	0	18	0	0			
8217	2020-4-12 8:00	NC	WPRO	0	18	0	0			
8218	2020-4-13 8:00	NC	WPRO	0	18	0	0			
8219	2020-4-14 8:00	NC	WPRO	0	18	0	0			
8220	2020-4-15 8:00	NC	WPRO	0	18	0	0			
8221	2020-4-16 8:00	NC	WPRO	0	18	0	0			
8222	2020-4-17 8:00	NC	WPRO	0	18	0	0			
8223	2020-4-18 8:00	NC	WPRO	0	18	0	0			
8224	2020-4-19 8:00	NC	WPRO	0	18	0	0			
8225	2020-4-20 8:00	NC	WPRO	0	18	0	0			
8226	2020-4-21 8:00	NC	WPRO	0	18	0	0			
8227	2020-4-22 8:00	NC	WPRO	0	18	0	0			
8228	2020-4-23 8:00	NC	WPRO	0	18	0	0			
8229	2020-4-24 8:00	NC	WPRO	0	18	0	0			
8230	2020-4-25 8:00	NC	WPRO	0	18	0	0			
8231	2020-4-26 8:00	NC	WPRO	0	18	0	0			
8232	2020-4-27 8:00	NC	WPRO	0	18	0	0			
8233	2020-4-28 8:00	NC	WPRO	0	18	0	0			
8234	2020-4-29 8:00	NC	WPRO	0	18	0	0			
8235	2020-4-30 8:00	NC	WPRO	0	18	0	0			
8236	2020-5-1 8:00	NC	WPRO	0	18	0	0			
8237	2020-5-2 8:00	NC	WPRO	0	18	0	0			
8238										
8239	2020-2-28 8:00	NZ	WPRO	1	1	0	0			
8240	2020-2-29 8:00	NZ	WPRO	0	1	0	0			
8241	2020-3-1 8:00	NZ	WPRO	0	1	0	0			
8242	2020-3-2 8:00	NZ	WPRO	0	1	0	0			
8243	2020-3-3 8:00	NZ	WPRO	1	2	0	0			
8244	2020-3-4 8:00	NZ	WPRO	0	2	0	0			

Table S1 Daily confirmed cases and daily deaths of COVID-19 in 214 nations from WHO

ID	Date and time	Country or region	Continent	Confirmed patients	Cumulative Confirmed patients	Daily dead patients	Daily cumulative dead patients	Lag days	Daily CFR	3DMA CFR
8245	2020-3-5 8:00	NZ	WPRO	0	2	0	0			
8246	2020-3-6 8:00	NZ	WPRO	2	4	0	0			
8247	2020-3-7 8:00	NZ	WPRO	1	5	0	0			
8248	2020-3-8 8:00	NZ	WPRO	0	5	0	0			
8249	2020-3-9 8:00	NZ	WPRO	0	5	0	0			
8250	2020-3-10 8:00	NZ	WPRO	0	5	0	0			
8251	2020-3-11 8:00	NZ	WPRO	0	5	0	0			
8252	2020-3-12 8:00	NZ	WPRO	0	5	0	0			
8253	2020-3-13 8:00	NZ	WPRO	0	5	0	0			
8254	2020-3-14 8:00	NZ	WPRO	1	6	0	0			
8255	2020-3-15 8:00	NZ	WPRO	0	6	0	0			
8256	2020-3-16 8:00	NZ	WPRO	0	6	0	0			
8257	2020-3-17 8:00	NZ	WPRO	5	11	0	0			
8258	2020-3-18 8:00	NZ	WPRO	9	20	0	0			
8259	2020-3-19 8:00	NZ	WPRO	19	39	0	0			
8260	2020-3-20 8:00	NZ	WPRO	14	53	0	0			
8261	2020-3-21 8:00	NZ	WPRO	13	66	0	0			
8262	2020-3-22 8:00	NZ	WPRO	0	66	0	0			
8263	2020-3-23 8:00	NZ	WPRO	36	102	0	0			
8264	2020-3-24 8:00	NZ	WPRO	40	142	0	0			
8265	2020-3-25 8:00	NZ	WPRO	47	189	0	0			
8266	2020-3-26 8:00	NZ	WPRO	73	262	0	0			
8267	2020-3-27 8:00	NZ	WPRO	76	338	0	0			
8268	2020-3-28 8:00	NZ	WPRO	78	416	0	0			
8269	2020-3-29 8:00	NZ	WPRO	60	476	1	1	0	0.210	0.196
8270	2020-3-30 8:00	NZ	WPRO	76	552	0	1	1	0.181	0.186
8271	2020-3-31 8:00	NZ	WPRO	48	600	0	1	2	0.167	0.167
8272	2020-4-1 8:00	NZ	WPRO	47	647	0	1	3	0.155	0.153
8273	2020-4-2 8:00	NZ	WPRO	76	723	0	1	4	0.138	0.141
8274	2020-4-3 8:00	NZ	WPRO	51	774	0	1	5	0.129	0.130
8275	2020-4-4 8:00	NZ	WPRO	50	824	0	1	6	0.121	0.122
8276	2020-4-5 8:00	NZ	WPRO	48	872	0	1	7	0.115	0.115
8277	2020-4-6 8:00	NZ	WPRO	39	911	0	1	8	0.110	0.110
8278	2020-4-7 8:00	NZ	WPRO	32	943	0	1	9	0.106	0.106
8279	2020-4-8 8:00	NZ	WPRO	26	969	0	1	10	0.103	0.103
8280	2020-4-9 8:00	NZ	WPRO	23	992	0	1	11	0.101	0.101

Table S1 Daily confirmed cases and daily deaths of COVID-19 in 214 nations from WHO

ID	Date and time	Country or region	Continent	Confirmed patients	Cumulative Confirmed patients	Daily dead patients	Daily cumulative dead patients	Lag days	Daily CFR	3DMA CFR
8281	2020-4-10 8:00	NZ	WPRO	23	1015	0	1	12	0.099	0.195
8282	2020-4-11 8:00	NZ	WPRO	20	1035	3	4	13	0.386	0.289
8283	2020-4-12 8:00	NZ	WPRO	14	1049	0	4	14	0.381	0.413
8284	2020-4-13 8:00	NZ	WPRO	15	1064	1	5	15	0.470	0.564
8285	2020-4-14 8:00	NZ	WPRO	8	1072	4	9	16	0.840	0.715
8286	2020-4-15 8:00	NZ	WPRO	6	1078	0	9	17	0.835	0.835
8287	2020-4-16 8:00	NZ	WPRO	6	1084	0	9	18	0.830	0.893
8288	2020-4-17 8:00	NZ	WPRO	2	1086	2	11	19	1.013	0.950
8289	2020-4-18 8:00	NZ	WPRO	8	1094	0	11	20	1.005	1.007
8290	2020-4-19 8:00	NZ	WPRO	4	1098	0	11	21	1.002	1.031
8291	2020-4-20 8:00	NZ	WPRO	7	1105	1	12	22	1.086	1.087
8292	2020-4-21 8:00	NZ	WPRO	2	1107	1	13	23	1.174	1.173
8293	2020-4-22 8:00	NZ	WPRO	5	1112	1	14	24	1.259	1.291
8294	2020-4-23 8:00	NZ	WPRO	0	1112	2	16	25	1.439	1.408
8295	2020-4-24 8:00	NZ	WPRO	2	1114	1	17	26	1.526	1.525
8296	2020-4-25 8:00	NZ	WPRO	3	1117	1	18	27	1.611	1.581
8297	2020-4-26 8:00	NZ	WPRO	4	1121	0	18	28	1.606	1.637
8298	2020-4-27 8:00	NZ	WPRO	1	1122	1	19	29	1.693	1.663
8299	2020-4-28 8:00	NZ	WPRO	2	1124	0	19	30	1.690	1.690
8300	2020-4-29 8:00	NZ	WPRO	2	1126	0	19	31	1.687	1.687
8301	2020-4-30 8:00	NZ	WPRO	3	1129	0	19	32	1.683	1.683
8302	2020-5-1 8:00	NZ	WPRO	3	1132	0	19	33	1.678	1.708
8303	2020-5-2 8:00	NZ	WPRO	2	1134	1	20	34	1.764	1.721
8304										
8305	2020-3-19 8:00	NI	AMRO	1	1	0	0			
8306	2020-3-20 8:00	NI	AMRO	0	1	0	0			
8307	2020-3-21 8:00	NI	AMRO	1	2	0	0			
8308	2020-3-22 8:00	NI	AMRO	0	2	0	0			
8309	2020-3-23 8:00	NI	AMRO	0	2	0	0			
8310	2020-3-24 8:00	NI	AMRO	0	2	0	0			
8311	2020-3-25 8:00	NI	AMRO	0	2	0	0			
8312	2020-3-26 8:00	NI	AMRO	0	2	0	0			
8313	2020-3-27 8:00	NI	AMRO	0	2	0	0			
8314	2020-3-28 8:00	NI	AMRO	0	2	1	1	0	50.000	37.500
8315	2020-3-29 8:00	NI	AMRO	2	4	0	1	1	25.000	33.333
8316	2020-3-30 8:00	NI	AMRO	0	4	0	1	2	25.000	25.000

Table S1 Daily confirmed cases and daily deaths of COVID-19 in 214 nations from WHO

ID	Date and time	Country or region	Continent	Confirmed patients	Cumulative Confirmed patients	Daily dead patients	Daily cumulative dead patients	Lag days	Daily CFR	3DMA CFR
8317	2020-3-31 8:00	NI	AMRO	0	4	0	1	3	25.000	25.000
8318	2020-4-1 8:00	NI	AMRO	0	4	0	1	4	25.000	23.333
8319	2020-4-2 8:00	NI	AMRO	1	5	0	1	5	20.000	21.667
8320	2020-4-3 8:00	NI	AMRO	0	5	0	1	6	20.000	20.000
8321	2020-4-4 8:00	NI	AMRO	0	5	0	1	7	20.000	20.000
8322	2020-4-5 8:00	NI	AMRO	0	5	0	1	8	20.000	20.000
8323	2020-4-6 8:00	NI	AMRO	0	5	0	1	9	20.000	18.889
8324	2020-4-7 8:00	NI	AMRO	1	6	0	1	10	16.667	17.778
8325	2020-4-8 8:00	NI	AMRO	0	6	0	1	11	16.667	16.667
8326	2020-4-9 8:00	NI	AMRO	0	6	0	1	12	16.667	16.667
8327	2020-4-10 8:00	NI	AMRO	0	6	0	1	13	16.667	15.873
8328	2020-4-11 8:00	NI	AMRO	1	7	0	1	14	14.286	15.079
8329	2020-4-12 8:00	NI	AMRO	0	7	0	1	15	14.286	13.228
8330	2020-4-13 8:00	NI	AMRO	2	9	0	1	16	11.111	12.169
8331	2020-4-14 8:00	NI	AMRO	0	9	0	1	17	11.111	11.111
8332	2020-4-15 8:00	NI	AMRO	0	9	0	1	18	11.111	11.111
8333	2020-4-16 8:00	NI	AMRO	0	9	0	1	19	11.111	11.111
8334	2020-4-17 8:00	NI	AMRO	0	9	0	1	20	11.111	11.111
8335	2020-4-18 8:00	NI	AMRO	0	9	0	1	21	11.111	11.111
8336	2020-4-19 8:00	NI	AMRO	0	9	0	1	22	11.111	11.111
8337	2020-4-20 8:00	NI	AMRO	0	9	0	1	23	11.111	14.815
8338	2020-4-21 8:00	NI	AMRO	0	9	1	2	24	22.222	17.778
8339	2020-4-22 8:00	NI	AMRO	1	10	0	2	25	20.000	20.741
8340	2020-4-23 8:00	NI	AMRO	0	10	0	2	26	20.000	20.000
8341	2020-4-24 8:00	NI	AMRO	0	10	0	2	27	20.000	22.424
8342	2020-4-25 8:00	NI	AMRO	1	11	1	3	28	27.273	24.848
8343	2020-4-26 8:00	NI	AMRO	0	11	0	3	29	27.273	25.874
8344	2020-4-27 8:00	NI	AMRO	2	13	0	3	30	23.077	24.476
8345	2020-4-28 8:00	NI	AMRO	0	13	0	3	31	23.077	23.077
8346	2020-4-29 8:00	NI	AMRO	0	13	0	3	32	23.077	23.077
8347	2020-4-30 8:00	NI	AMRO	0	13	0	3	33	23.077	24.908
8348	2020-5-1 8:00	NI	AMRO	1	14	1	4	34	28.571	26.740
8349	2020-5-2 8:00	NI	AMRO	0	14	0	4	35	28.571	28.571
8350										
8351	2020-3-19 8:00	NE	AFRO	1	1	0	0			
8352	2020-3-20 8:00	NE	AFRO	0	1	0	0			

Table S1 Daily confirmed cases and daily deaths of COVID-19 in 214 nations from WHO

ID	Date and time	Country or region	Continent	Confirmed patients	Cumulative Confirmed patients	Daily dead patients	Daily cumulative dead patients	Lag days	Daily CFR	3DMA CFR
8353	2020-3-21 8:00	NE	AFRO	0	1	0	0			
8354	2020-3-22 8:00	NE	AFRO	0	1	0	0			
8355	2020-3-23 8:00	NE	AFRO	1	2	0	0			
8356	2020-3-24 8:00	NE	AFRO	0	2	0	0			
8357	2020-3-25 8:00	NE	AFRO	0	2	0	0			
8358	2020-3-26 8:00	NE	AFRO	0	2	0	0			
8359	2020-3-27 8:00	NE	AFRO	8	10	1	1	0	10.000	10.000
8360	2020-3-28 8:00	NE	AFRO	0	10	0	1	1	10.000	10.000
8361	2020-3-29 8:00	NE	AFRO	0	10	0	1	2	10.000	11.667
8362	2020-3-30 8:00	NE	AFRO	10	20	2	3	3	15.000	13.333
8363	2020-3-31 8:00	NE	AFRO	0	20	0	3	4	15.000	15.000
8364	2020-4-1 8:00	NE	AFRO	0	20	0	3	5	15.000	12.252
8365	2020-4-2 8:00	NE	AFRO	54	74	2	5	6	6.757	8.953
8366	2020-4-3 8:00	NE	AFRO	24	98	0	5	7	5.102	5.342
8367	2020-4-4 8:00	NE	AFRO	22	120	0	5	8	4.167	4.941
8368	2020-4-5 8:00	NE	AFRO	24	144	3	8	9	5.556	5.052
8369	2020-4-6 8:00	NE	AFRO	40	184	2	10	10	5.435	4.981
8370	2020-4-7 8:00	NE	AFRO	69	253	0	10	11	3.953	4.448
8371	2020-4-8 8:00	NE	AFRO	25	278	1	11	12	3.957	3.709
8372	2020-4-9 8:00	NE	AFRO	64	342	0	11	13	3.216	3.285
8373	2020-4-10 8:00	NE	AFRO	68	410	0	11	14	2.683	2.804
8374	2020-4-11 8:00	NE	AFRO	28	438	0	11	15	2.511	2.478
8375	2020-4-12 8:00	NE	AFRO	53	491	0	11	16	2.240	2.340
8376	2020-4-13 8:00	NE	AFRO	38	529	1	12	17	2.268	2.294
8377	2020-4-14 8:00	NE	AFRO	19	548	1	13	18	2.372	2.366
8378	2020-4-15 8:00	NE	AFRO	22	570	1	14	19	2.456	2.409
8379	2020-4-16 8:00	NE	AFRO	14	584	0	14	20	2.397	2.439
8380	2020-4-17 8:00	NE	AFRO	25	609	1	15	21	2.463	2.577
8381	2020-4-18 8:00	NE	AFRO	18	627	3	18	22	2.871	2.769
8382	2020-4-19 8:00	NE	AFRO	12	639	1	19	23	2.973	2.977
8383	2020-4-20 8:00	NE	AFRO	9	648	1	20	24	3.086	3.038
8384	2020-4-21 8:00	NE	AFRO	7	655	0	20	25	3.053	3.061
8385	2020-4-22 8:00	NE	AFRO	2	657	0	20	26	3.044	3.140
8386	2020-4-23 8:00	NE	AFRO	5	662	2	22	27	3.323	3.315
8387	2020-4-24 8:00	NE	AFRO	9	671	2	24	28	3.577	3.475
8388	2020-4-25 8:00	NE	AFRO	10	681	0	24	29	3.524	3.683

Table S1 Daily confirmed cases and daily deaths of COVID-19 in 214 nations from WHO

ID	Date and time	Country or region	Continent	Confirmed patients	Cumulative Confirmed patients	Daily dead patients	Daily cumulative dead patients	Lag days	Daily CFR	3DMA CFR
8389	2020-4-26 8:00	NE	AFRO	3	684	3	27	30	3.947	3.879
8390	2020-4-27 8:00	NE	AFRO	12	696	2	29	31	4.167	4.084
8391	2020-4-28 8:00	NE	AFRO	5	701	0	29	32	4.137	4.225
8392	2020-4-29 8:00	NE	AFRO	8	709	2	31	33	4.372	4.332
8393	2020-4-30 8:00	NE	AFRO	4	713	1	32	34	4.488	4.437
8394	2020-5-1 8:00	NE	AFRO	6	719	0	32	35	4.451	4.491
8395	2020-5-2 8:00	NE	AFRO	9	728	1	33	36	4.533	4.492
8396										
8397	2020-2-28 8:00	NG	AFRO	1	1	0	0			
8398	2020-2-29 8:00	NG	AFRO	0	1	0	0			
8399	2020-3-1 8:00	NG	AFRO	0	1	0	0			
8400	2020-3-2 8:00	NG	AFRO	0	1	0	0			
8401	2020-3-3 8:00	NG	AFRO	0	1	0	0			
8402	2020-3-4 8:00	NG	AFRO	0	1	0	0			
8403	2020-3-5 8:00	NG	AFRO	0	1	0	0			
8404	2020-3-6 8:00	NG	AFRO	0	1	0	0			
8405	2020-3-7 8:00	NG	AFRO	0	1	0	0			
8406	2020-3-8 8:00	NG	AFRO	0	1	0	0			
8407	2020-3-9 8:00	NG	AFRO	1	2	0	0			
8408	2020-3-10 8:00	NG	AFRO	0	2	0	0			
8409	2020-3-11 8:00	NG	AFRO	0	2	0	0			
8410	2020-3-12 8:00	NG	AFRO	0	2	0	0			
8411	2020-3-13 8:00	NG	AFRO	0	2	0	0			
8412	2020-3-14 8:00	NG	AFRO	0	2	0	0			
8413	2020-3-15 8:00	NG	AFRO	0	2	0	0			
8414	2020-3-16 8:00	NG	AFRO	0	2	0	0			
8415	2020-3-17 8:00	NG	AFRO	0	2	0	0			
8416	2020-3-18 8:00	NG	AFRO	6	8	0	0			
8417	2020-3-19 8:00	NG	AFRO	4	12	0	0			
8418	2020-3-20 8:00	NG	AFRO	0	12	0	0			
8419	2020-3-21 8:00	NG	AFRO	10	22	0	0			
8420	2020-3-22 8:00	NG	AFRO	0	22	0	0			
8421	2020-3-23 8:00	NG	AFRO	0	22	0	0			
8422	2020-3-24 8:00	NG	AFRO	0	22	0	0			
8423	2020-3-25 8:00	NG	AFRO	20	42	0	0			
8424	2020-3-26 8:00	NG	AFRO	4	46	1	1	0	2.174	1.856

Table S1 Daily confirmed cases and daily deaths of COVID-19 in 214 nations from WHO

ID	Date and time	Country or region	Continent	Confirmed patients	Cumulative Confirmed patients	Daily dead patients	Daily cumulative dead patients	Lag days	Daily CFR	3DMA CFR
8425	2020-3-27 8:00	NG	AFRO	19	65	0	1	1	1.538	1.750
8426	2020-3-28 8:00	NG	AFRO	0	65	0	1	2	1.538	1.538
8427	2020-3-29 8:00	NG	AFRO	0	65	0	1	3	1.538	1.538
8428	2020-3-30 8:00	NG	AFRO	0	65	0	1	4	1.538	1.326
8429	2020-3-31 8:00	NG	AFRO	46	111	0	1	5	0.901	1.293
8430	2020-4-1 8:00	NG	AFRO	28	139	1	2	6	1.439	1.163
8431	2020-4-2 8:00	NG	AFRO	35	174	0	2	7	1.149	1.214
8432	2020-4-3 8:00	NG	AFRO	16	190	0	2	8	1.053	1.375
8433	2020-4-4 8:00	NG	AFRO	18	208	2	4	9	1.923	1.633
8434	2020-4-5 8:00	NG	AFRO	0	208	0	4	10	1.923	2.000
8435	2020-4-6 8:00	NG	AFRO	24	232	1	5	11	2.155	2.078
8436	2020-4-7 8:00	NG	AFRO	0	232	0	5	12	2.155	2.224
8437	2020-4-8 8:00	NG	AFRO	22	254	1	6	13	2.362	2.230
8438	2020-4-9 8:00	NG	AFRO	22	276	0	6	14	2.174	2.322
8439	2020-4-10 8:00	NG	AFRO	12	288	1	7	15	2.431	2.300
8440	2020-4-11 8:00	NG	AFRO	17	305	0	7	16	2.295	2.623
8441	2020-4-12 8:00	NG	AFRO	13	318	3	10	17	3.145	2.845
8442	2020-4-13 8:00	NG	AFRO	5	323	0	10	18	3.096	3.052
8443	2020-4-14 8:00	NG	AFRO	20	343	0	10	19	2.915	2.976
8444	2020-4-15 8:00	NG	AFRO	0	343	0	10	20	2.915	2.927
8445	2020-4-16 8:00	NG	AFRO	30	373	1	11	21	2.949	2.938
8446	2020-4-17 8:00	NG	AFRO	0	373	0	11	22	2.949	2.949
8447	2020-4-18 8:00	NG	AFRO	0	373	0	11	23	2.949	2.949
8448	2020-4-19 8:00	NG	AFRO	0	373	0	11	24	2.949	3.137
8449	2020-4-20 8:00	NG	AFRO	168	541	8	19	25	3.512	3.324
8450	2020-4-21 8:00	NG	AFRO	0	541	0	19	26	3.512	3.512
8451	2020-4-22 8:00	NG	AFRO	0	541	0	19	27	3.512	3.512
8452	2020-4-23 8:00	NG	AFRO	0	541	0	19	28	3.512	3.395
8453	2020-4-24 8:00	NG	AFRO	440	981	12	31	29	3.160	3.198
8454	2020-4-25 8:00	NG	AFRO	114	1095	1	32	30	2.922	3.014
8455	2020-4-26 8:00	NG	AFRO	87	1182	3	35	31	2.961	3.009
8456	2020-4-27 8:00	NG	AFRO	91	1273	5	40	32	3.142	3.032
8457	2020-4-28 8:00	NG	AFRO	64	1337	0	40	33	2.992	3.042
8458	2020-4-29 8:00	NG	AFRO	0	1337	0	40	34	2.992	2.952
8459	2020-4-30 8:00	NG	AFRO	195	1532	4	44	35	2.872	2.955
8460	2020-5-1 8:00	NG	AFRO	400	1932	14	58	36	3.002	3.003

Table S1 Daily confirmed cases and daily deaths of COVID-19 in 214 nations from WHO

ID	Date and time	Country or region	Continent	Confirmed patients	Cumulative Confirmed patients	Daily dead patients	Daily cumulative dead patients	Lag days	Daily CFR	3DMA CFR
8461	2020-5-2 8:00	NG	AFRO	238	2170	10	68	37	3.134	3.068
8462										
8463	2020-2-26 8:00	MK	EURO	1	1	0	0			
8464	2020-2-27 8:00	MK	EURO	0	1	0	0			
8465	2020-2-28 8:00	MK	EURO	0	1	0	0			
8466	2020-2-29 8:00	MK	EURO	0	1	0	0			
8467	2020-3-1 8:00	MK	EURO	0	1	0	0			
8468	2020-3-2 8:00	MK	EURO	0	1	0	0			
8469	2020-3-3 8:00	MK	EURO	0	1	0	0			
8470	2020-3-4 8:00	MK	EURO	0	1	0	0			
8471	2020-3-5 8:00	MK	EURO	0	1	0	0			
8472	2020-3-6 8:00	MK	EURO	2	3	0	0			
8473	2020-3-7 8:00	MK	EURO	0	3	0	0			
8474	2020-3-8 8:00	MK	EURO	0	3	0	0			
8475	2020-3-9 8:00	MK	EURO	1	4	0	0			
8476	2020-3-10 8:00	MK	EURO	3	7	0	0			
8477	2020-3-11 8:00	MK	EURO	0	7	0	0			
8478	2020-3-12 8:00	MK	EURO	0	7	0	0			
8479	2020-3-13 8:00	MK	EURO	2	9	0	0			
8480	2020-3-14 8:00	MK	EURO	4	13	0	0			
8481	2020-3-15 8:00	MK	EURO	0	13	0	0			
8482	2020-3-16 8:00	MK	EURO	6	19	0	0			
8483	2020-3-17 8:00	MK	EURO	12	31	0	0			
8484	2020-3-18 8:00	MK	EURO	0	31	0	0			
8485	2020-3-19 8:00	MK	EURO	17	48	0	0			
8486	2020-3-20 8:00	MK	EURO	22	70	0	0			
8487	2020-3-21 8:00	MK	EURO	15	85	0	0			
8488	2020-3-22 8:00	MK	EURO	0	85	0	0			
8489	2020-3-23 8:00	MK	EURO	29	114	1	1	0	0.877	1.174
8490	2020-3-24 8:00	MK	EURO	22	136	1	2	1	1.471	1.233
8491	2020-3-25 8:00	MK	EURO	12	148	0	2	2	1.351	1.317
8492	2020-3-26 8:00	MK	EURO	29	177	0	2	3	1.130	1.325
8493	2020-3-27 8:00	MK	EURO	24	201	1	3	4	1.493	1.331
8494	2020-3-28 8:00	MK	EURO	18	219	0	3	5	1.370	1.507
8495	2020-3-29 8:00	MK	EURO	22	241	1	4	6	1.660	1.782
8496	2020-3-30 8:00	MK	EURO	18	259	2	6	7	2.317	2.144

Table S1 Daily confirmed cases and daily deaths of COVID-19 in 214 nations from WHO

ID	Date and time	Country or region	Continent	Confirmed patients	Cumulative Confirmed patients	Daily dead patients	Daily cumulative dead patients	Lag days	Daily CFR	3DMA CFR
8497	2020-3-31 8:00	MK	EURO	26	285	1	7	8	2.456	2.503
8498	2020-4-1 8:00	MK	EURO	44	329	2	9	9	2.736	2.766
8499	2020-4-2 8:00	MK	EURO	25	354	2	11	10	3.107	2.902
8500	2020-4-3 8:00	MK	EURO	30	384	0	11	11	2.865	2.921
8501	2020-4-4 8:00	MK	EURO	46	430	1	12	12	2.791	3.058
8502	2020-4-5 8:00	MK	EURO	53	483	5	17	13	3.520	3.185
8503	2020-4-6 8:00	MK	EURO	72	555	1	18	14	3.243	3.482
8504	2020-4-7 8:00	MK	EURO	15	570	3	21	15	3.684	3.812
8505	2020-4-8 8:00	MK	EURO	29	599	6	27	16	4.508	4.351
8506	2020-4-9 8:00	MK	EURO	18	617	3	30	17	4.862	4.632
8507	2020-4-10 8:00	MK	EURO	46	663	0	30	18	4.525	4.629
8508	2020-4-11 8:00	MK	EURO	48	711	2	32	19	4.501	4.377
8509	2020-4-12 8:00	MK	EURO	117	828	2	34	20	4.106	4.238
8510	2020-4-13 8:00	MK	EURO	0	828	0	34	21	4.106	4.221
8511	2020-4-14 8:00	MK	EURO	26	854	4	38	22	4.450	4.467
8512	2020-4-15 8:00	MK	EURO	54	908	6	44	23	4.846	4.639
8513	2020-4-16 8:00	MK	EURO	66	974	1	45	24	4.620	4.574
8514	2020-4-17 8:00	MK	EURO	107	1081	1	46	25	4.255	4.421
8515	2020-4-18 8:00	MK	EURO	36	1117	3	49	26	4.387	4.277
8516	2020-4-19 8:00	MK	EURO	53	1170	0	49	27	4.188	4.267
8517	2020-4-20 8:00	MK	EURO	37	1207	2	51	28	4.225	4.274
8518	2020-4-21 8:00	MK	EURO	18	1225	3	54	29	4.408	4.367
8519	2020-4-22 8:00	MK	EURO	6	1231	1	55	30	4.468	4.441
8520	2020-4-23 8:00	MK	EURO	28	1259	1	56	31	4.448	4.408
8521	2020-4-24 8:00	MK	EURO	41	1300	0	56	32	4.308	4.351
8522	2020-4-25 8:00	MK	EURO	26	1326	1	57	33	4.299	4.307
8523	2020-4-26 8:00	MK	EURO	41	1367	2	59	34	4.316	4.339
8524	2020-4-27 8:00	MK	EURO	19	1386	2	61	35	4.401	4.454
8525	2020-4-28 8:00	MK	EURO	13	1399	4	65	36	4.646	4.681
8526	2020-4-29 8:00	MK	EURO	22	1421	6	71	37	4.996	4.902
8527	2020-4-30 8:00	MK	EURO	21	1442	2	73	38	5.062	5.105
8528	2020-5-1 8:00	MK	EURO	23	1465	4	77	39	5.256	5.247
8529	2020-5-2 8:00	MK	EURO	29	1494	4	81	40	5.422	5.339
8530										
8531	2020-3-29 8:00	MP	WPRO	2	2	0	0			
8532	2020-3-30 8:00	MP	WPRO	0	2	0	0			

Table S1 Daily confirmed cases and daily deaths of COVID-19 in 214 nations from WHO

ID	Date and time	Country or region	Continent	Confirmed patients	Cumulative Confirmed patients	Daily dead patients	Daily cumulative dead patients	Lag days	Daily CFR	3DMA CFR
8533	2020-3-31 8:00	MP	WPRO	0	2	0	0			
8534	2020-4-1 8:00	MP	WPRO	4	6	0	0			
8535	2020-4-2 8:00	MP	WPRO	0	6	0	0			
8536	2020-4-3 8:00	MP	WPRO	2	8	1	1	0	12.500	12.500
8537	2020-4-4 8:00	MP	WPRO	0	8	0	1	1	12.500	12.500
8538	2020-4-5 8:00	MP	WPRO	0	8	0	1	2	12.500	12.500
8539	2020-4-6 8:00	MP	WPRO	0	8	0	1	3	12.500	12.500
8540	2020-4-7 8:00	MP	WPRO	0	8	0	1	4	12.500	14.394
8541	2020-4-8 8:00	MP	WPRO	3	11	1	2	5	18.182	16.288
8542	2020-4-9 8:00	MP	WPRO	0	11	0	2	6	18.182	18.182
8543	2020-4-10 8:00	MP	WPRO	0	11	0	2	7	18.182	18.182
8544	2020-4-11 8:00	MP	WPRO	0	11	0	2	8	18.182	18.182
8545	2020-4-12 8:00	MP	WPRO	0	11	0	2	9	18.182	18.182
8546	2020-4-13 8:00	MP	WPRO	0	11	0	2	10	18.182	18.182
8547	2020-4-14 8:00	MP	WPRO	0	11	0	2	11	18.182	17.249
8548	2020-4-15 8:00	MP	WPRO	2	13	0	2	12	15.385	16.317
8549	2020-4-16 8:00	MP	WPRO	0	13	0	2	13	15.385	15.385
8550	2020-4-17 8:00	MP	WPRO	0	13	0	2	14	15.385	15.018
8551	2020-4-18 8:00	MP	WPRO	1	14	0	2	15	14.286	14.652
8552	2020-4-19 8:00	MP	WPRO	0	14	0	2	16	14.286	14.286
8553	2020-4-20 8:00	MP	WPRO	0	14	0	2	17	14.286	14.286
8554	2020-4-21 8:00	MP	WPRO	0	14	0	2	18	14.286	14.286
8555	2020-4-22 8:00	MP	WPRO	0	14	0	2	19	14.286	14.286
8556	2020-4-23 8:00	MP	WPRO	0	14	0	2	20	14.286	14.286
8557	2020-4-24 8:00	MP	WPRO	0	14	0	2	21	14.286	14.286
8558	2020-4-25 8:00	MP	WPRO	0	14	0	2	22	14.286	14.286
8559	2020-4-26 8:00	MP	WPRO	0	14	0	2	23	14.286	14.286
8560	2020-4-27 8:00	MP	WPRO	0	14	0	2	24	14.286	14.286
8561	2020-4-28 8:00	MP	WPRO	0	14	0	2	25	14.286	14.286
8562	2020-4-29 8:00	MP	WPRO	0	14	0	2	26	14.286	14.286
8563	2020-4-30 8:00	MP	WPRO	0	14	0	2	27	14.286	14.286
8564	2020-5-1 8:00	MP	WPRO	0	14	0	2	28	14.286	14.286
8565	2020-5-2 8:00	MP	WPRO	0	14	0	2	29	14.286	14.286
8566										
8567	2020-2-26 8:00	NO	EURO	1	1	0	0			
8568	2020-2-27 8:00	NO	EURO	3	4	0	0			

Table S1 Daily confirmed cases and daily deaths of COVID-19 in 214 nations from WHO

ID	Date and time	Country or region	Continent	Confirmed patients	Cumulative Confirmed patients	Daily dead patients	Daily cumulative dead patients	Lag days	Daily CFR	3DMA CFR
8569	2020-2-28 8:00	NO	EURO	2	6	0	0			
8570	2020-2-29 8:00	NO	EURO	1	7	0	0			
8571	2020-3-1 8:00	NO	EURO	12	19	0	0			
8572	2020-3-2 8:00	NO	EURO	6	25	0	0			
8573	2020-3-3 8:00	NO	EURO	8	33	0	0			
8574	2020-3-4 8:00	NO	EURO	0	33	0	0			
8575	2020-3-5 8:00	NO	EURO	23	56	0	0			
8576	2020-3-6 8:00	NO	EURO	57	113	0	0			
8577	2020-3-7 8:00	NO	EURO	34	147	0	0			
8578	2020-3-8 8:00	NO	EURO	22	169	0	0			
8579	2020-3-9 8:00	NO	EURO	23	192	0	0			
8580	2020-3-10 8:00	NO	EURO	85	277	0	0			
8581	2020-3-11 8:00	NO	EURO	0	277	0	0			
8582	2020-3-12 8:00	NO	EURO	0	277	0	0			
8583	2020-3-13 8:00	NO	EURO	473	750	1	1	0	0.133	0.122
8584	2020-3-14 8:00	NO	EURO	157	907	0	1	1	0.110	0.112
8585	2020-3-15 8:00	NO	EURO	170	1077	0	1	2	0.093	0.153
8586	2020-3-16 8:00	NO	EURO	92	1169	2	3	3	0.257	0.193
8587	2020-3-17 8:00	NO	EURO	139	1308	0	3	4	0.229	0.232
8588	2020-3-18 8:00	NO	EURO	115	1423	0	3	5	0.211	0.276
8589	2020-3-19 8:00	NO	EURO	129	1552	3	6	6	0.387	0.333
8590	2020-3-20 8:00	NO	EURO	190	1742	1	7	7	0.402	0.384
8591	2020-3-21 8:00	NO	EURO	184	1926	0	7	8	0.363	0.376
8592	2020-3-22 8:00	NO	EURO	0	1926	0	7	9	0.363	0.352
8593	2020-3-23 8:00	NO	EURO	206	2132	0	7	10	0.328	0.343
8594	2020-3-24 8:00	NO	EURO	239	2371	1	8	11	0.337	0.352
8595	2020-3-25 8:00	NO	EURO	195	2566	2	10	12	0.390	0.380
8596	2020-3-26 8:00	NO	EURO	350	2916	2	12	13	0.412	0.415
8597	2020-3-27 8:00	NO	EURO	240	3156	2	14	14	0.444	0.434
8598	2020-3-28 8:00	NO	EURO	425	3581	2	16	15	0.447	0.470
8599	2020-3-29 8:00	NO	EURO	264	3845	4	20	16	0.520	0.501
8600	2020-3-30 8:00	NO	EURO	257	4102	2	22	17	0.536	0.557
8601	2020-3-31 8:00	NO	EURO	124	4226	4	26	18	0.615	0.594
8602	2020-4-1 8:00	NO	EURO	221	4447	2	28	19	0.630	0.644
8603	2020-4-2 8:00	NO	EURO	218	4665	4	32	20	0.686	0.722
8604	2020-4-3 8:00	NO	EURO	270	4935	10	42	21	0.851	0.794

Table S1 Daily confirmed cases and daily deaths of COVID-19 in 214 nations from WHO

ID	Date and time	Country or region	Continent	Confirmed patients	Cumulative Confirmed patients	Daily dead patients	Daily cumulative dead patients	Lag days	Daily CFR	3DMA CFR
8605	2020-4-4 8:00	NO	EURO	273	5208	2	44	22	0.845	0.868
8606	2020-4-5 8:00	NO	EURO	302	5510	6	50	23	0.907	0.927
8607	2020-4-6 8:00	NO	EURO	130	5640	8	58	24	1.028	0.987
8608	2020-4-7 8:00	NO	EURO	115	5755	1	59	25	1.025	1.077
8609	2020-4-8 8:00	NO	EURO	108	5863	10	69	26	1.177	1.178
8610	2020-4-9 8:00	NO	EURO	147	6010	11	80	27	1.331	1.312
8611	2020-4-10 8:00	NO	EURO	150	6160	8	88	28	1.429	1.411
8612	2020-4-11 8:00	NO	EURO	84	6244	4	92	29	1.473	1.484
8613	2020-4-12 8:00	NO	EURO	76	6320	6	98	30	1.551	1.543
8614	2020-4-13 8:00	NO	EURO	95	6415	5	103	31	1.606	1.638
8615	2020-4-14 8:00	NO	EURO	73	6488	11	114	32	1.757	1.766
8616	2020-4-15 8:00	NO	EURO	78	6566	13	127	33	1.934	1.879
8617	2020-4-16 8:00	NO	EURO	111	6677	3	130	34	1.947	1.961
8618	2020-4-17 8:00	NO	EURO	114	6791	6	136	35	2.003	1.984
8619	2020-4-18 8:00	NO	EURO	0	6791	0	136	36	2.003	2.041
8620	2020-4-19 8:00	NO	EURO	193	6984	12	148	37	2.119	2.100
8621	2020-4-20 8:00	NO	EURO	84	7068	6	154	38	2.179	2.154
8622	2020-4-21 8:00	NO	EURO	45	7113	0	154	39	2.165	2.206
8623	2020-4-22 8:00	NO	EURO	53	7166	9	163	40	2.275	2.257
8624	2020-4-23 8:00	NO	EURO	84	7250	6	169	41	2.331	2.352
8625	2020-4-24 8:00	NO	EURO	95	7345	11	180	42	2.451	2.453
8626	2020-4-25 8:00	NO	EURO	63	7408	11	191	43	2.578	2.538
8627	2020-4-26 8:00	NO	EURO	59	7467	2	193	44	2.585	2.578
8628	2020-4-27 8:00	NO	EURO	38	7505	0	193	45	2.572	2.573
8629	2020-4-28 8:00	NO	EURO	28	7533	0	193	46	2.562	2.566
8630	2020-4-29 8:00	NO	EURO	72	7605	2	195	47	2.564	2.587
8631	2020-4-30 8:00	NO	EURO	62	7667	7	202	48	2.635	2.615
8632	2020-5-1 8:00	NO	EURO	43	7710	2	204	49	2.646	2.637
8633	2020-5-2 8:00	NO	EURO	49	7759	0	204	50	2.629	2.638
8634										
8635	2020-3-5 8:00	PS	EMRO	7	7	0	0			
8636	2020-3-6 8:00	PS	EMRO	9	16	0	0			
8637	2020-3-7 8:00	PS	EMRO	0	16	0	0			
8638	2020-3-8 8:00	PS	EMRO	3	19	0	0			
8639	2020-3-9 8:00	PS	EMRO	6	25	0	0			
8640	2020-3-10 8:00	PS	EMRO	4	29	0	0			

Table S1 Daily confirmed cases and daily deaths of COVID-19 in 214 nations from WHO

ID	Date and time	Country or region	Continent	Confirmed patients	Cumulative Confirmed patients	Daily dead patients	Daily cumulative dead patients	Lag days	Daily CFR	3DMA CFR
8641	2020-3-11 8:00	PS	EMRO	1	30	0	0			
8642	2020-3-12 8:00	PS	EMRO	1	31	0	0			
8643	2020-3-13 8:00	PS	EMRO	4	35	0	0			
8644	2020-3-14 8:00	PS	EMRO	3	38	0	0			
8645	2020-3-15 8:00	PS	EMRO	0	38	0	0			
8646	2020-3-16 8:00	PS	EMRO	1	39	0	0			
8647	2020-3-17 8:00	PS	EMRO	2	41	0	0			
8648	2020-3-18 8:00	PS	EMRO	0	41	0	0			
8649	2020-3-19 8:00	PS	EMRO	6	47	0	0			
8650	2020-3-20 8:00	PS	EMRO	1	48	0	0			
8651	2020-3-21 8:00	PS	EMRO	4	52	0	0			
8652	2020-3-22 8:00	PS	EMRO	7	59	0	0			
8653	2020-3-23 8:00	PS	EMRO	0	59	0	0			
8654	2020-3-24 8:00	PS	EMRO	1	60	0	0			
8655	2020-3-25 8:00	PS	EMRO	2	62	0	0			
8656	2020-3-26 8:00	PS	EMRO	22	84	1	1	0	1.190	1.145
8657	2020-3-27 8:00	PS	EMRO	7	91	0	1	1	1.099	1.107
8658	2020-3-28 8:00	PS	EMRO	6	97	0	1	2	1.031	1.024
8659	2020-3-29 8:00	PS	EMRO	9	106	0	1	3	0.943	0.948
8660	2020-3-30 8:00	PS	EMRO	9	115	0	1	4	0.870	0.889
8661	2020-3-31 8:00	PS	EMRO	2	117	0	1	5	0.855	0.824
8662	2020-4-1 8:00	PS	EMRO	17	134	0	1	6	0.746	0.749
8663	2020-4-2 8:00	PS	EMRO	21	155	0	1	7	0.645	0.659
8664	2020-4-3 8:00	PS	EMRO	16	171	0	1	8	0.585	0.573
8665	2020-4-4 8:00	PS	EMRO	34	205	0	1	9	0.488	0.511
8666	2020-4-5 8:00	PS	EMRO	12	217	0	1	10	0.461	0.448
8667	2020-4-6 8:00	PS	EMRO	36	253	0	1	11	0.395	0.417
8668	2020-4-7 8:00	PS	EMRO	1	254	0	1	12	0.394	0.390
8669	2020-4-8 8:00	PS	EMRO	9	263	0	1	13	0.380	0.385
8670	2020-4-9 8:00	PS	EMRO	0	263	0	1	14	0.380	0.504
8671	2020-4-10 8:00	PS	EMRO	3	266	1	2	15	0.752	0.626
8672	2020-4-11 8:00	PS	EMRO	2	268	0	2	16	0.746	0.748
8673	2020-4-12 8:00	PS	EMRO	0	268	0	2	17	0.746	0.742
8674	2020-4-13 8:00	PS	EMRO	5	273	0	2	18	0.733	0.725
8675	2020-4-14 8:00	PS	EMRO	14	287	0	2	19	0.697	0.706
8676	2020-4-15 8:00	PS	EMRO	4	291	0	2	20	0.687	0.688

Table S1 Daily confirmed cases and daily deaths of COVID-19 in 214 nations from WHO

ID	Date and time	Country or region	Continent	Confirmed patients	Cumulative Confirmed patients	Daily dead patients	Daily cumulative dead patients	Lag days	Daily CFR	3DMA CFR
8677	2020-4-16 8:00	PS	EMRO	3	294	0	2	21	0.680	0.673
8678	2020-4-17 8:00	PS	EMRO	13	307	0	2	22	0.651	0.657
8679	2020-4-18 8:00	PS	EMRO	6	313	0	2	23	0.639	0.637
8680	2020-4-19 8:00	PS	EMRO	9	322	0	2	24	0.621	0.623
8681	2020-4-20 8:00	PS	EMRO	7	329	0	2	25	0.608	0.612
8682	2020-4-21 8:00	PS	EMRO	0	329	0	2	26	0.608	0.604
8683	2020-4-22 8:00	PS	EMRO	6	335	0	2	27	0.597	0.600
8684	2020-4-23 8:00	PS	EMRO	1	336	0	2	28	0.595	0.593
8685	2020-4-24 8:00	PS	EMRO	4	340	0	2	29	0.588	0.589
8686	2020-4-25 8:00	PS	EMRO	2	342	0	2	30	0.585	0.586
8687	2020-4-26 8:00	PS	EMRO	0	342	0	2	31	0.585	0.585
8688	2020-4-27 8:00	PS	EMRO	0	342	0	2	32	0.585	0.584
8689	2020-4-28 8:00	PS	EMRO	1	343	0	2	33	0.583	0.584
8690	2020-4-29 8:00	PS	EMRO	0	343	0	2	34	0.583	0.583
8691	2020-4-30 8:00	PS	EMRO	1	344	0	2	35	0.581	0.577
8692	2020-5-1 8:00	PS	EMRO	9	353	0	2	36	0.567	0.572
8693	2020-5-2 8:00	PS	EMRO	0	353	0	2	37	0.567	0.567
8694										
8695	2020-2-24 8:00	OM	EMRO	2	2	0	0			
8696	2020-2-25 8:00	OM	EMRO	2	4	0	0			
8697	2020-2-26 8:00	OM	EMRO	0	4	0	0			
8698	2020-2-27 8:00	OM	EMRO	2	6	0	0			
8699	2020-2-28 8:00	OM	EMRO	0	6	0	0			
8700	2020-2-29 8:00	OM	EMRO	0	6	0	0			
8701	2020-3-1 8:00	OM	EMRO	0	6	0	0			
8702	2020-3-2 8:00	OM	EMRO	0	6	0	0			
8703	2020-3-3 8:00	OM	EMRO	6	12	0	0			
8704	2020-3-4 8:00	OM	EMRO	3	15	0	0			
8705	2020-3-5 8:00	OM	EMRO	1	16	0	0			
8706	2020-3-6 8:00	OM	EMRO	0	16	0	0			
8707	2020-3-7 8:00	OM	EMRO	0	16	0	0			
8708	2020-3-8 8:00	OM	EMRO	0	16	0	0			
8709	2020-3-9 8:00	OM	EMRO	0	16	0	0			
8710	2020-3-10 8:00	OM	EMRO	2	18	0	0			
8711	2020-3-11 8:00	OM	EMRO	0	18	0	0			
8712	2020-3-12 8:00	OM	EMRO	0	18	0	0			

Table S1 Daily confirmed cases and daily deaths of COVID-19 in 214 nations from WHO

ID	Date and time	Country or region	Continent	Confirmed patients	Cumulative Confirmed patients	Daily dead patients	Daily cumulative dead patients	Lag days	Daily CFR	3DMA CFR
8713	2020-3-13 8:00	OM	EMRO	0	18	0	0			
8714	2020-3-14 8:00	OM	EMRO	2	20	0	0			
8715	2020-3-15 8:00	OM	EMRO	2	22	0	0			
8716	2020-3-16 8:00	OM	EMRO	0	22	0	0			
8717	2020-3-17 8:00	OM	EMRO	2	24	0	0			
8718	2020-3-18 8:00	OM	EMRO	9	33	0	0			
8719	2020-3-19 8:00	OM	EMRO	6	39	0	0			
8720	2020-3-20 8:00	OM	EMRO	9	48	0	0			
8721	2020-3-21 8:00	OM	EMRO	4	52	0	0			
8722	2020-3-22 8:00	OM	EMRO	3	55	0	0			
8723	2020-3-23 8:00	OM	EMRO	11	66	0	0			
8724	2020-3-24 8:00	OM	EMRO	18	84	0	0			
8725	2020-3-25 8:00	OM	EMRO	15	99	0	0			
8726	2020-3-26 8:00	OM	EMRO	10	109	0	0			
8727	2020-3-27 8:00	OM	EMRO	22	131	0	0			
8728	2020-3-28 8:00	OM	EMRO	21	152	0	0			
8729	2020-3-29 8:00	OM	EMRO	15	167	0	0			
8730	2020-3-30 8:00	OM	EMRO	12	179	0	0			
8731	2020-3-31 8:00	OM	EMRO	13	192	0	0			
8732	2020-4-1 8:00	OM	EMRO	18	210	1	1	0	0.476	0.455
8733	2020-4-2 8:00	OM	EMRO	21	231	0	1	1	0.433	0.435
8734	2020-4-3 8:00	OM	EMRO	21	252	0	1	2	0.397	0.397
8735	2020-4-4 8:00	OM	EMRO	25	277	0	1	3	0.361	0.476
8736	2020-4-5 8:00	OM	EMRO	21	298	1	2	4	0.671	0.545
8737	2020-4-6 8:00	OM	EMRO	33	331	0	2	5	0.604	0.605
8738	2020-4-7 8:00	OM	EMRO	40	371	0	2	6	0.539	0.540
8739	2020-4-8 8:00	OM	EMRO	48	419	0	2	7	0.477	0.485
8740	2020-4-9 8:00	OM	EMRO	38	457	0	2	8	0.438	0.512
8741	2020-4-10 8:00	OM	EMRO	27	484	1	3	9	0.620	0.536
8742	2020-4-11 8:00	OM	EMRO	62	546	0	3	10	0.549	0.612
8743	2020-4-12 8:00	OM	EMRO	53	599	1	4	11	0.668	0.589
8744	2020-4-13 8:00	OM	EMRO	128	727	0	4	12	0.550	0.570
8745	2020-4-14 8:00	OM	EMRO	86	813	0	4	13	0.492	0.494
8746	2020-4-15 8:00	OM	EMRO	97	910	0	4	14	0.440	0.441
8747	2020-4-16 8:00	OM	EMRO	109	1019	0	4	15	0.393	0.433
8748	2020-4-17 8:00	OM	EMRO	50	1069	1	5	16	0.468	0.456

Table S1 Daily confirmed cases and daily deaths of COVID-19 in 214 nations from WHO

ID	Date and time	Country or region	Continent	Confirmed patients	Cumulative Confirmed patients	Daily dead patients	Daily cumulative dead patients	Lag days	Daily CFR	3DMA CFR
8749	2020-4-18 8:00	OM	EMRO	111	1180	1	6	17	0.508	0.483
8750	2020-4-19 8:00	OM	EMRO	86	1266	0	6	18	0.474	0.493
8751	2020-4-20 8:00	OM	EMRO	144	1410	1	7	19	0.496	0.500
8752	2020-4-21 8:00	OM	EMRO	98	1508	1	8	20	0.531	0.508
8753	2020-4-22 8:00	OM	EMRO	106	1614	0	8	21	0.496	0.497
8754	2020-4-23 8:00	OM	EMRO	102	1716	0	8	22	0.466	0.488
8755	2020-4-24 8:00	OM	EMRO	74	1790	1	9	23	0.503	0.498
8756	2020-4-25 8:00	OM	EMRO	115	1905	1	10	24	0.525	0.509
8757	2020-4-26 8:00	OM	EMRO	93	1998	0	10	25	0.501	0.504
8758	2020-4-27 8:00	OM	EMRO	51	2049	0	10	26	0.488	0.486
8759	2020-4-28 8:00	OM	EMRO	82	2131	0	10	27	0.469	0.466
8760	2020-4-29 8:00	OM	EMRO	143	2274	0	10	28	0.440	0.459
8761	2020-4-30 8:00	OM	EMRO	74	2348	1	11	29	0.468	0.453
8762	2020-5-1 8:00	OM	EMRO	99	2447	0	11	30	0.450	0.454
8763	2020-5-2 8:00	OM	EMRO	36	2483	0	11	31	0.443	0.446
8764										
8765	2020-2-26 8:00	PK	EMRO	2	2	0	0			
8766	2020-2-27 8:00	PK	EMRO	0	2	0	0			
8767	2020-2-28 8:00	PK	EMRO	0	2	0	0			
8768	2020-2-29 8:00	PK	EMRO	2	4	0	0			
8769	2020-3-1 8:00	PK	EMRO	0	4	0	0			
8770	2020-3-2 8:00	PK	EMRO	1	5	0	0			
8771	2020-3-3 8:00	PK	EMRO	0	5	0	0			
8772	2020-3-4 8:00	PK	EMRO	0	5	0	0			
8773	2020-3-5 8:00	PK	EMRO	0	5	0	0			
8774	2020-3-6 8:00	PK	EMRO	0	5	0	0			
8775	2020-3-7 8:00	PK	EMRO	0	5	0	0			
8776	2020-3-8 8:00	PK	EMRO	1	6	0	0			
8777	2020-3-9 8:00	PK	EMRO	0	6	0	0			
8778	2020-3-10 8:00	PK	EMRO	10	16	0	0			
8779	2020-3-11 8:00	PK	EMRO	3	19	0	0			
8780	2020-3-12 8:00	PK	EMRO	1	20	0	0			
8781	2020-3-13 8:00	PK	EMRO	0	20	0	0			
8782	2020-3-14 8:00	PK	EMRO	8	28	0	0			
8783	2020-3-15 8:00	PK	EMRO	24	52	0	0			
8784	2020-3-16 8:00	PK	EMRO	1	53	0	0			

Table S1 Daily confirmed cases and daily deaths of COVID-19 in 214 nations from WHO

ID	Date and time	Country or region	Continent	Confirmed patients	Cumulative Confirmed patients	Daily dead patients	Daily cumulative dead patients	Lag days	Daily CFR	3DMA CFR
8785	2020-3-17 8:00	PK	EMRO	134	187	0	0			
8786	2020-3-18 8:00	PK	EMRO	54	241	0	0			
8787	2020-3-19 8:00	PK	EMRO	61	302	2	2	0	0.662	0.548
8788	2020-3-20 8:00	PK	EMRO	159	461	0	2	1	0.434	0.567
8789	2020-3-21 8:00	PK	EMRO	34	495	1	3	2	0.606	0.501
8790	2020-3-22 8:00	PK	EMRO	151	646	0	3	3	0.464	0.569
8791	2020-3-23 8:00	PK	EMRO	138	784	2	5	4	0.638	0.593
8792	2020-3-24 8:00	PK	EMRO	103	887	1	6	5	0.676	0.674
8793	2020-3-25 8:00	PK	EMRO	104	991	1	7	6	0.706	0.713
8794	2020-3-26 8:00	PK	EMRO	66	1057	1	8	7	0.757	0.738
8795	2020-3-27 8:00	PK	EMRO	140	1197	1	9	8	0.752	0.763
8796	2020-3-28 8:00	PK	EMRO	211	1408	2	11	9	0.781	0.795
8797	2020-3-29 8:00	PK	EMRO	118	1526	2	13	10	0.852	0.914
8798	2020-3-30 8:00	PK	EMRO	99	1625	5	18	11	1.108	1.100
8799	2020-3-31 8:00	PK	EMRO	240	1865	7	25	12	1.340	1.241
8800	2020-4-1 8:00	PK	EMRO	174	2039	1	26	13	1.275	1.323
8801	2020-4-2 8:00	PK	EMRO	252	2291	5	31	14	1.353	1.352
8802	2020-4-3 8:00	PK	EMRO	159	2450	4	35	15	1.429	1.403
8803	2020-4-4 8:00	PK	EMRO	0	2450	0	35	16	1.429	1.443
8804	2020-4-5 8:00	PK	EMRO	609	3059	10	45	17	1.471	1.453
8805	2020-4-6 8:00	PK	EMRO	365	3424	5	50	18	1.460	1.427
8806	2020-4-7 8:00	PK	EMRO	581	4005	4	54	19	1.348	1.398
8807	2020-4-8 8:00	PK	EMRO	178	4183	4	58	20	1.387	1.379
8808	2020-4-9 8:00	PK	EMRO	306	4489	5	63	21	1.403	1.399
8809	2020-4-10 8:00	PK	EMRO	206	4695	3	66	22	1.406	1.453
8810	2020-4-11 8:00	PK	EMRO	275	4970	11	77	23	1.549	1.551
8811	2020-4-12 8:00	PK	EMRO	213	5183	11	88	24	1.698	1.646
8812	2020-4-13 8:00	PK	EMRO	313	5496	5	93	25	1.692	1.678
8813	2020-4-14 8:00	PK	EMRO	341	5837	3	96	26	1.645	1.708
8814	2020-4-15 8:00	PK	EMRO	151	5988	11	107	27	1.787	1.761
8815	2020-4-16 8:00	PK	EMRO	931	6919	21	128	28	1.850	1.853
8816	2020-4-17 8:00	PK	EMRO	106	7025	7	135	29	1.922	1.881
8817	2020-4-18 8:00	PK	EMRO	613	7638	8	143	30	1.872	1.928
8818	2020-4-19 8:00	PK	EMRO	355	7993	16	159	31	1.989	1.984
8819	2020-4-20 8:00	PK	EMRO	425	8418	17	176	32	2.091	2.051
8820	2020-4-21 8:00	PK	EMRO	1087	9505	21	197	33	2.073	2.102

Table S1 Daily confirmed cases and daily deaths of COVID-19 in 214 nations from WHO

ID	Date and time	Country or region	Continent	Confirmed patients	Cumulative Confirmed patients	Daily dead patients	Daily cumulative dead patients	Lag days	Daily CFR	3DMA CFR
8821	2020-4-22 8:00	PK	EMRO	244	9749	12	209	34	2.144	2.108
8822	2020-4-23 8:00	PK	EMRO	1066	10815	19	228	35	2.108	2.126
8823	2020-4-24 8:00	PK	EMRO	340	11155	9	237	36	2.125	2.109
8824	2020-4-25 8:00	PK	EMRO	1072	12227	19	256	37	2.094	2.106
8825	2020-4-26 8:00	PK	EMRO	970	13197	21	277	38	2.099	2.097
8826	2020-4-27 8:00	PK	EMRO	718	13915	15	292	39	2.098	2.112
8827	2020-4-28 8:00	PK	EMRO	164	14079	9	301	40	2.138	2.143
8828	2020-4-29 8:00	PK	EMRO	1210	15289	34	335	41	2.191	2.183
8829	2020-4-30 8:00	PK	EMRO	828	16117	23	358	42	2.221	2.239
8830	2020-5-1 8:00	PK	EMRO	1494	17611	48	406	43	2.305	2.276
8831	2020-5-2 8:00	PK	EMRO	503	18114	11	417	44	2.302	2.304
8832										
8833	2020-3-10 8:00	PA	AMRO	1	1	0	0			
8834	2020-3-11 8:00	PA	AMRO	7	8	1	1	0	12.500	11.250
8835	2020-3-12 8:00	PA	AMRO	2	10	0	1	1	10.000	9.881
8836	2020-3-13 8:00	PA	AMRO	4	14	0	1	2	7.143	6.949
8837	2020-3-14 8:00	PA	AMRO	13	27	0	1	3	3.704	4.850
8838	2020-3-15 8:00	PA	AMRO	0	27	0	1	4	3.704	3.244
8839	2020-3-16 8:00	PA	AMRO	16	43	0	1	5	2.326	2.493
8840	2020-3-17 8:00	PA	AMRO	26	69	0	1	6	1.449	1.646
8841	2020-3-18 8:00	PA	AMRO	17	86	0	1	7	1.163	1.176
8842	2020-3-19 8:00	PA	AMRO	23	109	0	1	8	0.917	0.937
8843	2020-3-20 8:00	PA	AMRO	28	137	0	1	9	0.730	0.792
8844	2020-3-21 8:00	PA	AMRO	0	137	0	1	10	0.730	0.730
8845	2020-3-22 8:00	PA	AMRO	0	137	0	1	11	0.730	0.895
8846	2020-3-23 8:00	PA	AMRO	108	245	2	3	12	1.224	1.231
8847	2020-3-24 8:00	PA	AMRO	100	345	3	6	13	1.739	1.568
8848	2020-3-25 8:00	PA	AMRO	0	345	0	6	14	1.739	1.637
8849	2020-3-26 8:00	PA	AMRO	213	558	2	8	15	1.434	1.536
8850	2020-3-27 8:00	PA	AMRO	0	558	0	8	16	1.434	1.401
8851	2020-3-28 8:00	PA	AMRO	116	674	1	9	17	1.335	1.517
8852	2020-3-29 8:00	PA	AMRO	112	786	5	14	18	1.781	1.668
8853	2020-3-30 8:00	PA	AMRO	115	901	3	17	19	1.887	2.032
8854	2020-3-31 8:00	PA	AMRO	88	989	7	24	20	2.427	2.247
8855	2020-4-1 8:00	PA	AMRO	0	989	0	24	21	2.427	2.465
8856	2020-4-2 8:00	PA	AMRO	192	1181	6	30	22	2.540	2.466

Table S1 Daily confirmed cases and daily deaths of COVID-19 in 214 nations from WHO

ID	Date and time	Country or region	Continent	Confirmed patients	Cumulative Confirmed patients	Daily dead patients	Daily cumulative dead patients	Lag days	Daily CFR	3DMA CFR
8857	2020-4-3 8:00	PA	AMRO	136	1317	2	32	23	2.430	2.493
8858	2020-4-4 8:00	PA	AMRO	158	1475	5	37	24	2.508	2.463
8859	2020-4-5 8:00	PA	AMRO	198	1673	4	41	25	2.451	2.504
8860	2020-4-6 8:00	PA	AMRO	128	1801	5	46	26	2.554	2.574
8861	2020-4-7 8:00	PA	AMRO	187	1988	8	54	27	2.716	2.630
8862	2020-4-8 8:00	PA	AMRO	112	2100	1	55	28	2.619	2.653
8863	2020-4-9 8:00	PA	AMRO	149	2249	4	59	29	2.623	2.578
8864	2020-4-10 8:00	PA	AMRO	279	2528	4	63	30	2.492	2.505
8865	2020-4-11 8:00	PA	AMRO	224	2752	3	66	31	2.398	2.460
8866	2020-4-12 8:00	PA	AMRO	222	2974	8	74	32	2.488	2.443
8867	2020-4-13 8:00	PA	AMRO	260	3234	5	79	33	2.443	2.497
8868	2020-4-14 8:00	PA	AMRO	166	3400	8	87	34	2.559	2.570
8869	2020-4-15 8:00	PA	AMRO	72	3472	7	94	35	2.707	2.641
8870	2020-4-16 8:00	PA	AMRO	102	3574	1	95	36	2.658	2.704
8871	2020-4-17 8:00	PA	AMRO	177	3751	8	103	37	2.746	2.706
8872	2020-4-18 8:00	PA	AMRO	265	4016	6	109	38	2.714	2.731
8873	2020-4-19 8:00	PA	AMRO	194	4210	6	115	39	2.732	2.751
8874	2020-4-20 8:00	PA	AMRO	63	4273	5	120	40	2.808	2.787
8875	2020-4-21 8:00	PA	AMRO	194	4467	6	126	41	2.821	2.850
8876	2020-4-22 8:00	PA	AMRO	191	4658	10	136	42	2.920	2.888
8877	2020-4-23 8:00	PA	AMRO	163	4821	5	141	43	2.925	2.910
8878	2020-4-24 8:00	PA	AMRO	171	4992	3	144	44	2.885	2.878
8879	2020-4-25 8:00	PA	AMRO	174	5166	2	146	45	2.826	2.865
8880	2020-4-26 8:00	PA	AMRO	172	5338	8	154	46	2.885	2.861
8881	2020-4-27 8:00	PA	AMRO	200	5538	5	159	47	2.871	2.870
8882	2020-4-28 8:00	PA	AMRO	241	5779	6	165	48	2.855	2.833
8883	2020-4-29 8:00	PA	AMRO	242	6021	2	167	49	2.774	2.823
8884	2020-4-30 8:00	PA	AMRO	179	6200	9	176	50	2.839	2.801
8885	2020-5-1 8:00	PA	AMRO	178	6378	2	178	51	2.791	2.836
8886	2020-5-2 8:00	PA	AMRO	154	6532	10	188	52	2.878	2.834
8887										
8888	2020-3-20 8:00	PG	WPRO	1	1	0	0			
8889	2020-3-21 8:00	PG	WPRO	0	1	0	0			
8890	2020-3-22 8:00	PG	WPRO	0	1	0	0			
8891	2020-3-23 8:00	PG	WPRO	0	1	0	0			
8892	2020-3-24 8:00	PG	WPRO	0	1	0	0			

Table S1 Daily confirmed cases and daily deaths of COVID-19 in 214 nations from WHO

ID	Date and time	Country or region	Continent	Confirmed patients	Cumulative Confirmed patients	Daily dead patients	Daily cumulative dead patients	Lag days	Daily CFR	3DMA CFR
8893	2020-3-25 8:00	PG	WPRO	0	1	0	0			
8894	2020-3-26 8:00	PG	WPRO	0	1	0	0			
8895	2020-3-27 8:00	PG	WPRO	0	1	0	0			
8896	2020-3-28 8:00	PG	WPRO	0	1	0	0			
8897	2020-3-29 8:00	PG	WPRO	0	1	0	0			
8898	2020-3-30 8:00	PG	WPRO	0	1	0	0			
8899	2020-3-31 8:00	PG	WPRO	0	1	0	0			
8900	2020-4-1 8:00	PG	WPRO	0	1	0	0			
8901	2020-4-2 8:00	PG	WPRO	0	1	0	0			
8902	2020-4-3 8:00	PG	WPRO	0	1	0	0			
8903	2020-4-4 8:00	PG	WPRO	0	1	0	0			
8904	2020-4-5 8:00	PG	WPRO	0	1	0	0			
8905	2020-4-6 8:00	PG	WPRO	0	1	0	0			
8906	2020-4-7 8:00	PG	WPRO	0	1	0	0			
8907	2020-4-8 8:00	PG	WPRO	0	1	0	0			
8908	2020-4-9 8:00	PG	WPRO	1	2	0	0			
8909	2020-4-10 8:00	PG	WPRO	0	2	0	0			
8910	2020-4-11 8:00	PG	WPRO	0	2	0	0			
8911	2020-4-12 8:00	PG	WPRO	0	2	0	0			
8912	2020-4-13 8:00	PG	WPRO	0	2	0	0			
8913	2020-4-14 8:00	PG	WPRO	0	2	0	0			
8914	2020-4-15 8:00	PG	WPRO	0	2	0	0			
8915	2020-4-16 8:00	PG	WPRO	0	2	0	0			
8916	2020-4-17 8:00	PG	WPRO	5	7	0	0			
8917	2020-4-18 8:00	PG	WPRO	0	7	0	0			
8918	2020-4-19 8:00	PG	WPRO	0	7	0	0			
8919	2020-4-20 8:00	PG	WPRO	0	7	0	0			
8920	2020-4-21 8:00	PG	WPRO	0	7	0	0			
8921	2020-4-22 8:00	PG	WPRO	0	7	0	0			
8922	2020-4-23 8:00	PG	WPRO	1	8	0	0			
8923	2020-4-24 8:00	PG	WPRO	0	8	0	0			
8924	2020-4-25 8:00	PG	WPRO	0	8	0	0			
8925	2020-4-26 8:00	PG	WPRO	0	8	0	0			
8926	2020-4-27 8:00	PG	WPRO	0	8	0	0			
8927	2020-4-28 8:00	PG	WPRO	0	8	0	0			
8928	2020-4-29 8:00	PG	WPRO	0	8	0	0			

Table S1 Daily confirmed cases and daily deaths of COVID-19 in 214 nations from WHO

ID	Date and time	Country or region	Continent	Confirmed patients	Cumulative Confirmed patients	Daily dead patients	Daily cumulative dead patients	Lag days	Daily CFR	3DMA CFR
8929	2020-4-30 8:00	PG	WPRO	0	8	0	0			
8930	2020-5-1 8:00	PG	WPRO	0	8	0	0			
8931	2020-5-2 8:00	PG	WPRO	0	8	0	0			
8932										
8933	2020-3-8 8:00	PY	AMRO	1	1	0	0			
8934	2020-3-9 8:00	PY	AMRO	0	1	0	0			
8935	2020-3-10 8:00	PY	AMRO	0	1	0	0			
8936	2020-3-11 8:00	PY	AMRO	4	5	0	0			
8937	2020-3-12 8:00	PY	AMRO	0	5	0	0			
8938	2020-3-13 8:00	PY	AMRO	0	5	0	0			
8939	2020-3-14 8:00	PY	AMRO	1	6	0	0			
8940	2020-3-15 8:00	PY	AMRO	0	6	0	0			
8941	2020-3-16 8:00	PY	AMRO	2	8	0	0			
8942	2020-3-17 8:00	PY	AMRO	1	9	0	0			
8943	2020-3-18 8:00	PY	AMRO	2	11	0	0			
8944	2020-3-19 8:00	PY	AMRO	0	11	0	0			
8945	2020-3-20 8:00	PY	AMRO	2	13	0	0			
8946	2020-3-21 8:00	PY	AMRO	5	18	1	1	0	5.556	5.556
8947	2020-3-22 8:00	PY	AMRO	0	18	0	1	1	5.556	5.219
8948	2020-3-23 8:00	PY	AMRO	4	22	0	1	2	4.545	5.836
8949	2020-3-24 8:00	PY	AMRO	5	27	1	2	3	7.407	6.453
8950	2020-3-25 8:00	PY	AMRO	0	27	0	2	4	7.407	7.377
8951	2020-3-26 8:00	PY	AMRO	14	41	1	3	5	7.317	7.347
8952	2020-3-27 8:00	PY	AMRO	0	41	0	3	6	7.317	6.801
8953	2020-3-28 8:00	PY	AMRO	11	52	0	3	7	5.769	6.148
8954	2020-3-29 8:00	PY	AMRO	4	56	0	3	8	5.357	5.404
8955	2020-3-30 8:00	PY	AMRO	3	59	0	3	9	5.085	5.043
8956	2020-3-31 8:00	PY	AMRO	5	64	0	3	10	4.688	4.796
8957	2020-4-1 8:00	PY	AMRO	1	65	0	3	11	4.615	4.550
8958	2020-4-2 8:00	PY	AMRO	4	69	0	3	12	4.348	4.286
8959	2020-4-3 8:00	PY	AMRO	8	77	0	3	13	3.896	3.835
8960	2020-4-4 8:00	PY	AMRO	15	92	0	3	14	3.261	3.427
8961	2020-4-5 8:00	PY	AMRO	4	96	0	3	15	3.125	3.090
8962	2020-4-6 8:00	PY	AMRO	8	104	0	3	16	2.885	3.478
8963	2020-4-7 8:00	PY	AMRO	9	113	2	5	17	4.425	3.886
8964	2020-4-8 8:00	PY	AMRO	2	115	0	5	18	4.348	4.325

Table S1 Daily confirmed cases and daily deaths of COVID-19 in 214 nations from WHO

ID	Date and time	Country or region	Continent	Confirmed patients	Cumulative Confirmed patients	Daily dead patients	Daily cumulative dead patients	Lag days	Daily CFR	3DMA CFR
8965	2020-4-9 8:00	PY	AMRO	4	119	0	5	19	4.202	4.194
8966	2020-4-10 8:00	PY	AMRO	5	124	0	5	20	4.032	4.295
8967	2020-4-11 8:00	PY	AMRO	5	129	1	6	21	4.651	4.398
8968	2020-4-12 8:00	PY	AMRO	4	133	0	6	22	4.511	4.547
8969	2020-4-13 8:00	PY	AMRO	1	134	0	6	23	4.478	4.357
8970	2020-4-14 8:00	PY	AMRO	13	147	0	6	24	4.082	4.321
8971	2020-4-15 8:00	PY	AMRO	12	159	1	7	25	4.403	4.484
8972	2020-4-16 8:00	PY	AMRO	2	161	1	8	26	4.969	4.656
8973	2020-4-17 8:00	PY	AMRO	13	174	0	8	27	4.598	4.529
8974	2020-4-18 8:00	PY	AMRO	25	199	0	8	28	4.020	4.193
8975	2020-4-19 8:00	PY	AMRO	3	202	0	8	29	3.960	3.955
8976	2020-4-20 8:00	PY	AMRO	4	206	0	8	30	3.883	3.897
8977	2020-4-21 8:00	PY	AMRO	2	208	0	8	31	3.846	3.859
8978	2020-4-22 8:00	PY	AMRO	0	208	0	8	32	3.846	3.973
8979	2020-4-23 8:00	PY	AMRO	5	213	1	9	33	4.225	4.099
8980	2020-4-24 8:00	PY	AMRO	0	213	0	9	34	4.225	4.181
8981	2020-4-25 8:00	PY	AMRO	7	220	0	9	35	4.091	4.117
8982	2020-4-26 8:00	PY	AMRO	3	223	0	9	36	4.036	4.025
8983	2020-4-27 8:00	PY	AMRO	5	228	0	9	37	3.947	3.977
8984	2020-4-28 8:00	PY	AMRO	0	228	0	9	38	3.947	3.936
8985	2020-4-29 8:00	PY	AMRO	2	230	0	9	39	3.913	3.875
8986	2020-4-30 8:00	PY	AMRO	9	239	0	9	40	3.766	3.764
8987	2020-5-1 8:00	PY	AMRO	10	249	0	9	41	3.614	3.713
8988	2020-5-2 8:00	PY	AMRO	17	266	1	10	42	3.759	3.687
8989										
8990	2020-3-7 8:00	PE	AMRO	6	6	0	0			
8991	2020-3-8 8:00	PE	AMRO	0	6	0	0			
8992	2020-3-9 8:00	PE	AMRO	1	7	0	0			
8993	2020-3-10 8:00	PE	AMRO	2	9	0	0			
8994	2020-3-11 8:00	PE	AMRO	2	11	0	0			
8995	2020-3-12 8:00	PE	AMRO	6	17	0	0			
8996	2020-3-13 8:00	PE	AMRO	5	22	0	0			
8997	2020-3-14 8:00	PE	AMRO	6	28	0	0			
8998	2020-3-15 8:00	PE	AMRO	15	43	0	0			
8999	2020-3-16 8:00	PE	AMRO	28	71	0	0			
9000	2020-3-17 8:00	PE	AMRO	15	86	0	0			

Table S1 Daily confirmed cases and daily deaths of COVID-19 in 214 nations from WHO

ID	Date and time	Country or region	Continent	Confirmed patients	Cumulative Confirmed patients	Daily dead patients	Daily cumulative dead patients	Lag days	Daily CFR	3DMA CFR
9001	2020-3-18 8:00	PE	AMRO	59	145	0	0			
9002	2020-3-19 8:00	PE	AMRO	89	234	0	0			
9003	2020-3-20 8:00	PE	AMRO	0	234	2	2	0	0.855	0.742
9004	2020-3-21 8:00	PE	AMRO	84	318	0	2	1	0.629	0.704
9005	2020-3-22 8:00	PE	AMRO	0	318	0	2	2	0.629	0.629
9006	2020-3-23 8:00	PE	AMRO	0	318	0	2	3	0.629	0.820
9007	2020-3-24 8:00	PE	AMRO	98	416	3	5	4	1.202	1.011
9008	2020-3-25 8:00	PE	AMRO	0	416	0	5	5	1.202	1.319
9009	2020-3-26 8:00	PE	AMRO	164	580	4	9	6	1.552	1.435
9010	2020-3-27 8:00	PE	AMRO	0	580	0	9	7	1.552	1.552
9011	2020-3-28 8:00	PE	AMRO	0	580	0	9	8	1.552	1.612
9012	2020-3-29 8:00	PE	AMRO	55	635	2	11	9	1.732	1.641
9013	2020-3-30 8:00	PE	AMRO	36	671	0	11	10	1.639	1.554
9014	2020-3-31 8:00	PE	AMRO	181	852	0	11	11	1.291	1.728
9015	2020-4-1 8:00	PE	AMRO	213	1065	13	24	12	2.254	1.786
9016	2020-4-2 8:00	PE	AMRO	258	1323	0	24	13	1.814	2.389
9017	2020-4-3 8:00	PE	AMRO	0	1323	17	41	14	3.099	2.840
9018	2020-4-4 8:00	PE	AMRO	91	1414	10	51	15	3.607	3.510
9019	2020-4-5 8:00	PE	AMRO	181	1595	10	61	16	3.824	3.871
9020	2020-4-6 8:00	PE	AMRO	151	1746	12	73	17	4.181	3.881
9021	2020-4-7 8:00	PE	AMRO	535	2281	10	83	18	3.639	3.804
9022	2020-4-8 8:00	PE	AMRO	280	2561	9	92	19	3.592	3.618
9023	2020-4-9 8:00	PE	AMRO	393	2954	15	107	20	3.622	3.334
9024	2020-4-10 8:00	PE	AMRO	1388	4342	14	121	21	2.787	3.012
9025	2020-4-11 8:00	PE	AMRO	914	5256	17	138	22	2.626	2.759
9026	2020-4-12 8:00	PE	AMRO	641	5897	31	169	23	2.866	2.712
9027	2020-4-13 8:00	PE	AMRO	951	6848	12	181	24	2.643	2.692
9028	2020-4-14 8:00	PE	AMRO	671	7519	12	193	25	2.567	2.592
9029	2020-4-15 8:00	PE	AMRO	0	7519	0	193	26	2.567	2.455
9030	2020-4-16 8:00	PE	AMRO	2784	10303	37	230	27	2.232	2.338
9031	2020-4-17 8:00	PE	AMRO	1172	11475	24	254	28	2.214	2.223
9032	2020-4-18 8:00	PE	AMRO	2014	13489	46	300	29	2.224	2.221
9033	2020-4-19 8:00	PE	AMRO	0	13489	0	300	30	2.224	2.287
9034	2020-4-20 8:00	PE	AMRO	931	14420	48	348	31	2.413	2.399
9035	2020-4-21 8:00	PE	AMRO	1208	15628	52	400	32	2.560	2.566
9036	2020-4-22 8:00	PE	AMRO	697	16325	45	445	33	2.726	2.666

Table S1 Daily confirmed cases and daily deaths of COVID-19 in 214 nations from WHO

ID	Date and time	Country or region	Continent	Confirmed patients	Cumulative Confirmed patients	Daily dead patients	Daily cumulative dead patients	Lag days	Daily CFR	3DMA CFR
9037	2020-4-23 8:00	PE	AMRO	1512	17837	39	484	34	2.713	2.731
9038	2020-4-24 8:00	PE	AMRO	1413	19250	46	530	35	2.753	2.734
9039	2020-4-25 8:00	PE	AMRO	1664	20914	42	572	36	2.735	2.806
9040	2020-4-26 8:00	PE	AMRO	734	21648	62	634	37	2.929	2.809
9041	2020-4-27 8:00	PE	AMRO	3683	25331	66	700	38	2.763	2.779
9042	2020-4-28 8:00	PE	AMRO	2186	27517	28	728	39	2.646	2.711
9043	2020-4-29 8:00	PE	AMRO	1182	28699	54	782	40	2.725	2.703
9044	2020-4-30 8:00	PE	AMRO	2491	31190	72	854	41	2.738	2.747
9045	2020-5-1 8:00	PE	AMRO	2741	33931	89	943	42	2.779	2.787
9046	2020-5-2 8:00	PE	AMRO	3045	36976	108	1051	43	2.842	2.811
9047										
9048	2020-1-30 8:00	PH	WPRO	1	1	0	0			
9049	2020-1-31 8:00	PH	WPRO	0	1	0	0			
9050	2020-2-1 8:00	PH	WPRO	1	2	0	0			
9051	2020-2-2 8:00	PH	WPRO	0	2	1	1	0	50.000	50.000
9052	2020-2-3 8:00	PH	WPRO	0	2	0	1	1	50.000	44.444
9053	2020-2-4 8:00	PH	WPRO	1	3	0	1	2	33.333	38.889
9054	2020-2-5 8:00	PH	WPRO	0	3	0	1	3	33.333	33.333
9055	2020-2-6 8:00	PH	WPRO	0	3	0	1	4	33.333	33.333
9056	2020-2-7 8:00	PH	WPRO	0	3	0	1	5	33.333	33.333
9057	2020-2-8 8:00	PH	WPRO	0	3	0	1	6	33.333	33.333
9058	2020-2-9 8:00	PH	WPRO	0	3	0	1	7	33.333	33.333
9059	2020-2-10 8:00	PH	WPRO	0	3	0	1	8	33.333	33.333
9060	2020-2-11 8:00	PH	WPRO	0	3	0	1	9	33.333	33.333
9061	2020-2-12 8:00	PH	WPRO	0	3	0	1	10	33.333	33.333
9062	2020-2-13 8:00	PH	WPRO	0	3	0	1	11	33.333	33.333
9063	2020-2-14 8:00	PH	WPRO	0	3	0	1	12	33.333	33.333
9064	2020-2-15 8:00	PH	WPRO	0	3	0	1	13	33.333	33.333
9065	2020-2-16 8:00	PH	WPRO	0	3	0	1	14	33.333	33.333
9066	2020-2-17 8:00	PH	WPRO	0	3	0	1	15	33.333	33.333
9067	2020-2-18 8:00	PH	WPRO	0	3	0	1	16	33.333	33.333
9068	2020-2-19 8:00	PH	WPRO	0	3	0	1	17	33.333	33.333
9069	2020-2-20 8:00	PH	WPRO	0	3	0	1	18	33.333	33.333
9070	2020-2-21 8:00	PH	WPRO	0	3	0	1	19	33.333	33.333
9071	2020-2-22 8:00	PH	WPRO	0	3	0	1	20	33.333	33.333
9072	2020-2-23 8:00	PH	WPRO	0	3	0	1	21	33.333	33.333

Table S1 Daily confirmed cases and daily deaths of COVID-19 in 214 nations from WHO

ID	Date and time	Country or region	Continent	Confirmed patients	Cumulative Confirmed patients	Daily dead patients	Daily cumulative dead patients	Lag days	Daily CFR	3DMA CFR
9073	2020-2-24 8:00	PH	WPRO	0	3	0	1	22	33.333	33.333
9074	2020-2-25 8:00	PH	WPRO	0	3	0	1	23	33.333	33.333
9075	2020-2-26 8:00	PH	WPRO	0	3	0	1	24	33.333	33.333
9076	2020-2-27 8:00	PH	WPRO	0	3	0	1	25	33.333	33.333
9077	2020-2-28 8:00	PH	WPRO	0	3	0	1	26	33.333	33.333
9078	2020-2-29 8:00	PH	WPRO	0	3	0	1	27	33.333	33.333
9079	2020-3-1 8:00	PH	WPRO	0	3	0	1	28	33.333	33.333
9080	2020-3-2 8:00	PH	WPRO	0	3	0	1	29	33.333	33.333
9081	2020-3-3 8:00	PH	WPRO	0	3	0	1	30	33.333	33.333
9082	2020-3-4 8:00	PH	WPRO	0	3	0	1	31	33.333	33.333
9083	2020-3-5 8:00	PH	WPRO	0	3	0	1	32	33.333	28.889
9084	2020-3-6 8:00	PH	WPRO	2	5	0	1	33	20.000	23.333
9085	2020-3-7 8:00	PH	WPRO	1	6	0	1	34	16.667	15.556
9086	2020-3-8 8:00	PH	WPRO	4	10	0	1	35	10.000	10.278
9087	2020-3-9 8:00	PH	WPRO	14	24	0	1	36	4.167	5.732
9088	2020-3-10 8:00	PH	WPRO	9	33	0	1	37	3.030	3.079
9089	2020-3-11 8:00	PH	WPRO	16	49	0	1	38	2.041	2.972
9090	2020-3-12 8:00	PH	WPRO	3	52	1	2	39	3.846	3.244
9091	2020-3-13 8:00	PH	WPRO	0	52	0	2	40	3.846	3.606
9092	2020-3-14 8:00	PH	WPRO	12	64	0	2	41	3.125	5.181
9093	2020-3-15 8:00	PH	WPRO	76	140	10	12	42	8.571	6.716
9094	2020-3-16 8:00	PH	WPRO	2	142	0	12	43	8.451	7.813
9095	2020-3-17 8:00	PH	WPRO	45	187	0	12	44	6.417	7.451
9096	2020-3-18 8:00	PH	WPRO	0	187	2	14	45	7.487	7.246
9097	2020-3-19 8:00	PH	WPRO	30	217	3	17	46	7.834	7.716
9098	2020-3-20 8:00	PH	WPRO	13	230	1	18	47	7.826	7.283
9099	2020-3-21 8:00	PH	WPRO	77	307	1	19	48	6.189	6.865
9100	2020-3-22 8:00	PH	WPRO	73	380	6	25	49	6.579	7.034
9101	2020-3-23 8:00	PH	WPRO	16	396	8	33	50	8.333	7.352
9102	2020-3-24 8:00	PH	WPRO	66	462	0	33	51	7.143	7.150
9103	2020-3-25 8:00	PH	WPRO	174	636	5	38	52	5.975	6.494
9104	2020-3-26 8:00	PH	WPRO	71	707	7	45	53	6.365	6.235
9105	2020-3-27 8:00	PH	WPRO	0	707	0	45	54	6.365	6.352
9106	2020-3-28 8:00	PH	WPRO	368	1075	23	68	55	6.326	6.339
9107	2020-3-29 8:00	PH	WPRO	0	1075	0	68	56	6.326	5.899
9108	2020-3-30 8:00	PH	WPRO	471	1546	10	78	57	5.045	5.472

Table S1 Daily confirmed cases and daily deaths of COVID-19 in 214 nations from WHO

ID	Date and time	Country or region	Continent	Confirmed patients	Cumulative Confirmed patients	Daily dead patients	Daily cumulative dead patients	Lag days	Daily CFR	3DMA CFR
9109	2020-3-31 8:00	PH	WPRO	0	1546	0	78	58	5.045	4.748
9110	2020-4-1 8:00	PH	WPRO	765	2311	18	96	59	4.154	4.451
9111	2020-4-2 8:00	PH	WPRO	0	2311	0	96	60	4.154	4.124
9112	2020-4-3 8:00	PH	WPRO	322	2633	11	107	61	4.064	4.241
9113	2020-4-4 8:00	PH	WPRO	385	3018	29	136	62	4.506	4.408
9114	2020-4-5 8:00	PH	WPRO	76	3094	8	144	63	4.654	4.614
9115	2020-4-6 8:00	PH	WPRO	152	3246	8	152	64	4.683	4.597
9116	2020-4-7 8:00	PH	WPRO	414	3660	11	163	65	4.454	4.613
9117	2020-4-8 8:00	PH	WPRO	210	3870	19	182	66	4.703	4.712
9118	2020-4-9 8:00	PH	WPRO	206	4076	21	203	67	4.980	4.888
9119	2020-4-10 8:00	PH	WPRO	0	4076	0	203	68	4.980	5.180
9120	2020-4-11 8:00	PH	WPRO	352	4428	44	247	69	5.578	5.649
9121	2020-4-12 8:00	PH	WPRO	220	4648	50	297	70	6.390	6.118
9122	2020-4-13 8:00	PH	WPRO	284	4932	18	315	71	6.387	6.397
9123	2020-4-14 8:00	PH	WPRO	291	5223	20	335	72	6.414	6.405
9124	2020-4-15 8:00	PH	WPRO	0	5223	0	335	73	6.414	6.408
9125	2020-4-16 8:00	PH	WPRO	437	5660	27	362	74	6.396	6.402
9126	2020-4-17 8:00	PH	WPRO	0	5660	0	362	75	6.396	6.458
9127	2020-4-18 8:00	PH	WPRO	218	5878	25	387	76	6.584	6.501
9128	2020-4-19 8:00	PH	WPRO	209	6087	10	397	77	6.522	6.547
9129	2020-4-20 8:00	PH	WPRO	172	6259	12	409	78	6.535	6.561
9130	2020-4-21 8:00	PH	WPRO	200	6459	19	428	79	6.626	6.594
9131	2020-4-22 8:00	PH	WPRO	140	6599	9	437	80	6.622	6.632
9132	2020-4-23 8:00	PH	WPRO	111	6710	9	446	81	6.647	6.629
9133	2020-4-24 8:00	PH	WPRO	271	6981	16	462	82	6.618	6.632
9134	2020-4-25 8:00	PH	WPRO	211	7192	15	477	83	6.632	6.674
9135	2020-4-26 8:00	PH	WPRO	102	7294	17	494	84	6.773	6.672
9136	2020-4-27 8:00	PH	WPRO	285	7579	7	501	85	6.610	6.651
9137	2020-4-28 8:00	PH	WPRO	198	7777	10	511	86	6.571	6.614
9138	2020-4-29 8:00	PH	WPRO	181	7958	19	530	87	6.660	6.675
9139	2020-4-30 8:00	PH	WPRO	254	8212	28	558	88	6.795	6.716
9140	2020-5-1 8:00	PH	WPRO	276	8488	10	568	89	6.692	6.696
9141	2020-5-2 8:00	PH	WPRO	284	8772	11	579	90	6.601	6.646
9142										
9143	2020-3-5 8:00	PL	EURO	1	1	0	0			
9144	2020-3-6 8:00	PL	EURO	4	5	0	0			

Table S1 Daily confirmed cases and daily deaths of COVID-19 in 214 nations from WHO

ID	Date and time	Country or region	Continent	Confirmed patients	Cumulative Confirmed patients	Daily dead patients	Daily cumulative dead patients	Lag days	Daily CFR	3DMA CFR
9145	2020-3-7 8:00	PL	EURO	1	6	0	0			
9146	2020-3-8 8:00	PL	EURO	5	11	0	0			
9147	2020-3-9 8:00	PL	EURO	5	16	0	0			
9148	2020-3-10 8:00	PL	EURO	6	22	0	0			
9149	2020-3-11 8:00	PL	EURO	5	27	0	0			
9150	2020-3-12 8:00	PL	EURO	24	51	1	1	0	1.961	1.762
9151	2020-3-13 8:00	PL	EURO	13	64	0	1	1	1.563	1.695
9152	2020-3-14 8:00	PL	EURO	0	64	0	1	2	1.563	1.943
9153	2020-3-15 8:00	PL	EURO	47	111	2	3	3	2.703	2.088
9154	2020-3-16 8:00	PL	EURO	39	150	0	3	4	2.000	2.245
9155	2020-3-17 8:00	PL	EURO	96	246	2	5	5	2.033	1.925
9156	2020-3-18 8:00	PL	EURO	41	287	0	5	6	1.742	1.771
9157	2020-3-19 8:00	PL	EURO	38	325	0	5	7	1.538	1.486
9158	2020-3-20 8:00	PL	EURO	100	425	0	5	8	1.176	1.216
9159	2020-3-21 8:00	PL	EURO	111	536	0	5	9	0.933	1.014
9160	2020-3-22 8:00	PL	EURO	0	536	0	5	10	0.933	0.990
9161	2020-3-23 8:00	PL	EURO	98	634	2	7	11	1.104	1.035
9162	2020-3-24 8:00	PL	EURO	115	749	1	8	12	1.068	1.094
9163	2020-3-25 8:00	PL	EURO	152	901	2	10	13	1.110	1.170
9164	2020-3-26 8:00	PL	EURO	150	1051	4	14	14	1.332	1.251
9165	2020-3-27 8:00	PL	EURO	170	1221	2	16	15	1.310	1.265
9166	2020-3-28 8:00	PL	EURO	168	1389	0	16	16	1.152	1.187
9167	2020-3-29 8:00	PL	EURO	249	1638	2	18	17	1.099	1.144
9168	2020-3-30 8:00	PL	EURO	224	1862	4	22	18	1.182	1.263
9169	2020-3-31 8:00	PL	EURO	193	2055	9	31	19	1.509	1.373
9170	2020-4-1 8:00	PL	EURO	256	2311	2	33	20	1.428	1.540
9171	2020-4-2 8:00	PL	EURO	243	2554	10	43	21	1.684	1.682
9172	2020-4-3 8:00	PL	EURO	392	2946	14	57	22	1.935	1.906
9173	2020-4-4 8:00	PL	EURO	437	3383	14	71	23	2.099	2.071
9174	2020-4-5 8:00	PL	EURO	244	3627	8	79	24	2.178	2.189
9175	2020-4-6 8:00	PL	EURO	475	4102	15	94	25	2.292	2.298
9176	2020-4-7 8:00	PL	EURO	311	4413	13	107	26	2.425	2.459
9177	2020-4-8 8:00	PL	EURO	435	4848	22	129	27	2.661	2.713
9178	2020-4-9 8:00	PL	EURO	357	5205	30	159	28	3.055	2.946
9179	2020-4-10 8:00	PL	EURO	370	5575	15	174	29	3.121	3.072
9180	2020-4-11 8:00	PL	EURO	380	5955	7	181	30	3.039	3.144

Table S1 Daily confirmed cases and daily deaths of COVID-19 in 214 nations from WHO

ID	Date and time	Country or region	Continent	Confirmed patients	Cumulative Confirmed patients	Daily dead patients	Daily cumulative dead patients	Lag days	Daily CFR	3DMA CFR
9181	2020-4-12 8:00	PL	EURO	401	6356	27	208	31	3.272	3.263
9182	2020-4-13 8:00	PL	EURO	318	6674	24	232	32	3.476	3.427
9183	2020-4-14 8:00	PL	EURO	260	6934	13	245	33	3.533	3.554
9184	2020-4-15 8:00	PL	EURO	268	7202	18	263	34	3.652	3.652
9185	2020-4-16 8:00	PL	EURO	380	7582	23	286	35	3.772	3.797
9186	2020-4-17 8:00	PL	EURO	336	7918	28	314	36	3.966	3.900
9187	2020-4-18 8:00	PL	EURO	461	8379	18	332	37	3.962	3.966
9188	2020-4-19 8:00	PL	EURO	363	8742	15	347	38	3.969	3.936
9189	2020-4-20 8:00	PL	EURO	545	9287	13	360	39	3.876	3.936
9190	2020-4-21 8:00	PL	EURO	306	9593	20	380	40	3.961	3.969
9191	2020-4-22 8:00	PL	EURO	263	9856	21	401	41	4.069	4.073
9192	2020-4-23 8:00	PL	EURO	313	10169	25	426	42	4.189	4.192
9193	2020-4-24 8:00	PL	EURO	342	10511	28	454	43	4.319	4.348
9194	2020-4-25 8:00	PL	EURO	381	10892	40	494	44	4.535	4.501
9195	2020-4-26 8:00	PL	EURO	381	11273	30	524	45	4.648	4.596
9196	2020-4-27 8:00	PL	EURO	344	11617	11	535	46	4.605	4.658
9197	2020-4-28 8:00	PL	EURO	285	11902	27	562	47	4.722	4.735
9198	2020-4-29 8:00	PL	EURO	316	12218	34	596	48	4.878	4.846
9199	2020-4-30 8:00	PL	EURO	422	12640	28	624	49	4.937	4.939
9200	2020-5-1 8:00	PL	EURO	237	12877	20	644	50	5.001	4.968
9201	2020-5-2 8:00	PL	EURO	228	13105	7	651	51	4.968	4.984
9202										
9203	2020-3-2 8:00	PT	EURO	2	2	0	0			
9204	2020-3-3 8:00	PT	EURO	0	2	0	0			
9205	2020-3-4 8:00	PT	EURO	4	6	0	0			
9206	2020-3-5 8:00	PT	EURO	3	9	0	0			
9207	2020-3-6 8:00	PT	EURO	4	13	0	0			
9208	2020-3-7 8:00	PT	EURO	0	13	0	0			
9209	2020-3-8 8:00	PT	EURO	17	30	0	0			
9210	2020-3-9 8:00	PT	EURO	0	30	0	0			
9211	2020-3-10 8:00	PT	EURO	11	41	0	0			
9212	2020-3-11 8:00	PT	EURO	0	41	0	0			
9213	2020-3-12 8:00	PT	EURO	0	41	0	0			
9214	2020-3-13 8:00	PT	EURO	71	112	0	0			
9215	2020-3-14 8:00	PT	EURO	0	112	0	0			
9216	2020-3-15 8:00	PT	EURO	133	245	0	0			

Table S1 Daily confirmed cases and daily deaths of COVID-19 in 214 nations from WHO

ID	Date and time	Country or region	Continent	Confirmed patients	Cumulative Confirmed patients	Daily dead patients	Daily cumulative dead patients	Lag days	Daily CFR	3DMA CFR
9217	2020-3-16 8:00	PT	EURO	86	331	0	0			
9218	2020-3-17 8:00	PT	EURO	117	448	1	1	0	0.223	0.267
9219	2020-3-18 8:00	PT	EURO	194	642	1	2	1	0.312	0.306
9220	2020-3-19 8:00	PT	EURO	143	785	1	3	2	0.382	0.427
9221	2020-3-20 8:00	PT	EURO	235	1020	3	6	3	0.588	0.636
9222	2020-3-21 8:00	PT	EURO	260	1280	6	12	4	0.938	0.821
9223	2020-3-22 8:00	PT	EURO	0	1280	0	12	5	0.938	0.917
9224	2020-3-23 8:00	PT	EURO	320	1600	2	14	6	0.875	0.976
9225	2020-3-24 8:00	PT	EURO	460	2060	9	23	7	1.117	1.130
9226	2020-3-25 8:00	PT	EURO	302	2362	10	33	8	1.397	1.316
9227	2020-3-26 8:00	PT	EURO	633	2995	10	43	9	1.436	1.509
9228	2020-3-27 8:00	PT	EURO	549	3544	17	60	10	1.693	1.636
9229	2020-3-28 8:00	PT	EURO	724	4268	16	76	11	1.781	1.803
9230	2020-3-29 8:00	PT	EURO	902	5170	24	100	12	1.934	1.904
9231	2020-3-30 8:00	PT	EURO	792	5962	19	119	13	1.996	2.038
9232	2020-3-31 8:00	PT	EURO	446	6408	21	140	14	2.185	2.110
9233	2020-4-1 8:00	PT	EURO	1035	7443	20	160	15	2.150	2.200
9234	2020-4-2 8:00	PT	EURO	808	8251	27	187	16	2.266	2.243
9235	2020-4-3 8:00	PT	EURO	783	9034	22	209	17	2.313	2.356
9236	2020-4-4 8:00	PT	EURO	852	9886	37	246	18	2.488	2.443
9237	2020-4-5 8:00	PT	EURO	638	10524	20	266	19	2.528	2.544
9238	2020-4-6 8:00	PT	EURO	754	11278	29	295	20	2.616	2.598
9239	2020-4-7 8:00	PT	EURO	452	11730	16	311	21	2.651	2.680
9240	2020-4-8 8:00	PT	EURO	712	12442	34	345	22	2.773	2.772
9241	2020-4-9 8:00	PT	EURO	699	13141	35	380	23	2.892	2.865
9242	2020-4-10 8:00	PT	EURO	815	13956	29	409	24	2.931	2.878
9243	2020-4-11 8:00	PT	EURO	1516	15472	26	435	25	2.812	2.894
9244	2020-4-12 8:00	PT	EURO	515	15987	35	470	26	2.940	2.930
9245	2020-4-13 8:00	PT	EURO	598	16585	34	504	27	3.039	3.046
9246	2020-4-14 8:00	PT	EURO	349	16934	31	535	28	3.159	3.149
9247	2020-4-15 8:00	PT	EURO	514	17448	32	567	29	3.250	3.240
9248	2020-4-16 8:00	PT	EURO	643	18091	32	599	30	3.311	3.300
9249	2020-4-17 8:00	PT	EURO	750	18841	30	629	31	3.338	3.368
9250	2020-4-18 8:00	PT	EURO	181	19022	28	657	32	3.454	3.427
9251	2020-4-19 8:00	PT	EURO	663	19685	30	687	33	3.490	3.492
9252	2020-4-20 8:00	PT	EURO	521	20206	27	714	34	3.534	3.516

Table S1 Daily confirmed cases and daily deaths of COVID-19 in 214 nations from WHO

ID	Date and time	Country or region	Continent	Confirmed patients	Cumulative Confirmed patients	Daily dead patients	Daily cumulative dead patients	Lag days	Daily CFR	3DMA CFR
9253	2020-4-21 8:00	PT	EURO	657	20863	21	735	35	3.523	3.540
9254	2020-4-22 8:00	PT	EURO	516	21379	27	762	36	3.564	3.553
9255	2020-4-23 8:00	PT	EURO	603	21982	23	785	37	3.571	3.601
9256	2020-4-24 8:00	PT	EURO	371	22353	35	820	38	3.668	3.662
9257	2020-4-25 8:00	PT	EURO	444	22797	34	854	39	3.746	3.725
9258	2020-4-26 8:00	PT	EURO	595	23392	26	880	40	3.762	3.764
9259	2020-4-27 8:00	PT	EURO	472	23864	23	903	41	3.784	3.803
9260	2020-4-28 8:00	PT	EURO	163	24027	25	928	42	3.862	3.848
9261	2020-4-29 8:00	PT	EURO	295	24322	20	948	43	3.898	3.910
9262	2020-4-30 8:00	PT	EURO	183	24505	25	973	44	3.971	3.938
9263	2020-5-1 8:00	PT	EURO	551	25056	16	989	45	3.947	3.963
9264	2020-5-2 8:00	PT	EURO	295	25351	18	1007	46	3.972	3.960
9265										
9266	2020-3-14 8:00	PR	AMRO	3	3	0	0			
9267	2020-3-15 8:00	PR	AMRO	0	3	0	0			
9268	2020-3-16 8:00	PR	AMRO	0	3	0	0			
9269	2020-3-17 8:00	PR	AMRO	0	3	0	0			
9270	2020-3-18 8:00	PR	AMRO	2	5	0	0			
9271	2020-3-19 8:00	PR	AMRO	1	6	0	0			
9272	2020-3-20 8:00	PR	AMRO	8	14	0	0			
9273	2020-3-21 8:00	PR	AMRO	7	21	1	1	0	4.762	4.762
9274	2020-3-22 8:00	PR	AMRO	0	21	0	1	1	4.762	4.624
9275	2020-3-23 8:00	PR	AMRO	2	23	0	1	2	4.348	4.746
9276	2020-3-24 8:00	PR	AMRO	16	39	1	2	3	5.128	4.868
9277	2020-3-25 8:00	PR	AMRO	0	39	0	2	4	5.128	4.460
9278	2020-3-26 8:00	PR	AMRO	25	64	0	2	5	3.125	3.793
9279	2020-3-27 8:00	PR	AMRO	0	64	0	2	6	3.125	3.125
9280	2020-3-28 8:00	PR	AMRO	0	64	0	2	7	3.125	3.125
9281	2020-3-29 8:00	PR	AMRO	0	64	0	2	8	3.125	3.125
9282	2020-3-30 8:00	PR	AMRO	0	64	0	2	9	3.125	3.233
9283	2020-3-31 8:00	PR	AMRO	110	174	4	6	10	3.448	3.307
9284	2020-4-1 8:00	PR	AMRO	65	239	2	8	11	3.347	3.547
9285	2020-4-2 8:00	PR	AMRO	47	286	3	11	12	3.846	3.664
9286	2020-4-3 8:00	PR	AMRO	30	316	1	12	13	3.797	3.871
9287	2020-4-4 8:00	PR	AMRO	62	378	3	15	14	3.968	3.916
9288	2020-4-5 8:00	PR	AMRO	74	452	3	18	15	3.982	3.978

Table S1 Daily confirmed cases and daily deaths of COVID-19 in 214 nations from WHO

ID	Date and time	Country or region	Continent	Confirmed patients	Cumulative Confirmed patients	Daily dead patients	Daily cumulative dead patients	Lag days	Daily CFR	3DMA CFR
9289	2020-4-6 8:00	PR	AMRO	0	452	0	18	16	3.982	4.019
9290	2020-4-7 8:00	PR	AMRO	61	513	3	21	17	4.094	4.030
9291	2020-4-8 8:00	PR	AMRO	60	573	2	23	18	4.014	3.993
9292	2020-4-9 8:00	PR	AMRO	47	620	1	24	19	3.871	4.239
9293	2020-4-10 8:00	PR	AMRO	63	683	9	33	20	4.832	4.694
9294	2020-4-11 8:00	PR	AMRO	42	725	6	39	21	5.379	5.180
9295	2020-4-12 8:00	PR	AMRO	63	788	3	42	22	5.330	5.205
9296	2020-4-13 8:00	PR	AMRO	109	897	2	44	23	4.905	5.073
9297	2020-4-14 8:00	PR	AMRO	6	903	1	45	24	4.983	4.921
9298	2020-4-15 8:00	PR	AMRO	20	923	0	45	25	4.875	4.929
9299	2020-4-16 8:00	PR	AMRO	51	974	3	48	26	4.928	4.802
9300	2020-4-17 8:00	PR	AMRO	69	1043	0	48	27	4.602	4.675
9301	2020-4-18 8:00	PR	AMRO	25	1068	0	48	28	4.494	4.463
9302	2020-4-19 8:00	PR	AMRO	50	1118	0	48	29	4.293	4.248
9303	2020-4-20 8:00	PR	AMRO	95	1213	0	48	30	3.957	4.028
9304	2020-4-21 8:00	PR	AMRO	39	1252	0	48	31	3.834	3.851
9305	2020-4-22 8:00	PR	AMRO	24	1276	0	48	32	3.762	3.786
9306	2020-4-23 8:00	PR	AMRO	0	1276	0	48	33	3.762	3.762
9307	2020-4-24 8:00	PR	AMRO	0	1276	0	48	34	3.762	3.840
9308	2020-4-25 8:00	PR	AMRO	0	1276	3	51	35	3.997	3.971
9309	2020-4-26 8:00	PR	AMRO	0	1276	2	53	36	4.154	4.005
9310	2020-4-27 8:00	PR	AMRO	95	1371	0	53	37	3.866	3.945
9311	2020-4-28 8:00	PR	AMRO	18	1389	0	53	38	3.816	3.846
9312	2020-4-29 8:00	PR	AMRO	11	1400	1	54	39	3.857	3.814
9313	2020-4-30 8:00	PR	AMRO	33	1433	0	54	40	3.768	3.711
9314	2020-5-1 8:00	PR	AMRO	106	1539	0	54	41	3.509	3.569
9315	2020-5-2 8:00	PR	AMRO	36	1575	0	54	42	3.429	3.469
9316										
9317	2020-2-29 8:00	QA	EMRO	1	1	0	0			
9318	2020-3-1 8:00	QA	EMRO	2	3	0	0			
9319	2020-3-2 8:00	QA	EMRO	4	7	0	0			
9320	2020-3-3 8:00	QA	EMRO	1	8	0	0			
9321	2020-3-4 8:00	QA	EMRO	0	8	0	0			
9322	2020-3-5 8:00	QA	EMRO	0	8	0	0			
9323	2020-3-6 8:00	QA	EMRO	3	11	0	0			
9324	2020-3-7 8:00	QA	EMRO	1	12	0	0			

Table S1 Daily confirmed cases and daily deaths of COVID-19 in 214 nations from WHO

ID	Date and time	Country or region	Continent	Confirmed patients	Cumulative Confirmed patients	Daily dead patients	Daily cumulative dead patients	Lag days	Daily CFR	3DMA CFR
9325	2020-3-8 8:00	QA	EMRO	0	12	0	0			
9326	2020-3-9 8:00	QA	EMRO	3	15	0	0			
9327	2020-3-10 8:00	QA	EMRO	3	18	0	0			
9328	2020-3-11 8:00	QA	EMRO	244	262	0	0			
9329	2020-3-12 8:00	QA	EMRO	0	262	0	0			
9330	2020-3-13 8:00	QA	EMRO	0	262	0	0			
9331	2020-3-14 8:00	QA	EMRO	75	337	0	0			
9332	2020-3-15 8:00	QA	EMRO	0	337	0	0			
9333	2020-3-16 8:00	QA	EMRO	64	401	0	0			
9334	2020-3-17 8:00	QA	EMRO	41	442	0	0			
9335	2020-3-18 8:00	QA	EMRO	0	442	0	0			
9336	2020-3-19 8:00	QA	EMRO	10	452	0	0			
9337	2020-3-20 8:00	QA	EMRO	8	460	0	0			
9338	2020-3-21 8:00	QA	EMRO	10	470	0	0			
9339	2020-3-22 8:00	QA	EMRO	11	481	0	0			
9340	2020-3-23 8:00	QA	EMRO	13	494	0	0			
9341	2020-3-24 8:00	QA	EMRO	7	501	0	0			
9342	2020-3-25 8:00	QA	EMRO	25	526	0	0			
9343	2020-3-26 8:00	QA	EMRO	11	537	0	0			
9344	2020-3-27 8:00	QA	EMRO	12	549	0	0			
9345	2020-3-28 8:00	QA	EMRO	13	562	0	0			
9346	2020-3-29 8:00	QA	EMRO	28	590	0	0			
9347	2020-3-30 8:00	QA	EMRO	44	634	1	1	0	0.158	0.151
9348	2020-3-31 8:00	QA	EMRO	59	693	0	1	1	0.144	0.186
9349	2020-4-1 8:00	QA	EMRO	88	781	1	2	2	0.256	0.213
9350	2020-4-2 8:00	QA	EMRO	54	835	0	2	3	0.240	0.258
9351	2020-4-3 8:00	QA	EMRO	240	1075	1	3	4	0.279	0.266
9352	2020-4-4 8:00	QA	EMRO	0	1075	0	3	5	0.279	0.262
9353	2020-4-5 8:00	QA	EMRO	250	1325	0	3	6	0.226	0.252
9354	2020-4-6 8:00	QA	EMRO	279	1604	1	4	7	0.249	0.231
9355	2020-4-7 8:00	QA	EMRO	228	1832	0	4	8	0.218	0.246
9356	2020-4-8 8:00	QA	EMRO	378	2210	2	6	9	0.271	0.247
9357	2020-4-9 8:00	QA	EMRO	166	2376	0	6	10	0.253	0.254
9358	2020-4-10 8:00	QA	EMRO	136	2512	0	6	11	0.239	0.237
9359	2020-4-11 8:00	QA	EMRO	216	2728	0	6	12	0.220	0.231
9360	2020-4-12 8:00	QA	EMRO	251	2979	1	7	13	0.235	0.224

Table S1 Daily confirmed cases and daily deaths of COVID-19 in 214 nations from WHO

ID	Date and time	Country or region	Continent	Confirmed patients	Cumulative Confirmed patients	Daily dead patients	Daily cumulative dead patients	Lag days	Daily CFR	3DMA CFR
9361	2020-4-13 8:00	QA	EMRO	252	3231	0	7	14	0.217	0.219
9362	2020-4-14 8:00	QA	EMRO	197	3428	0	7	15	0.204	0.203
9363	2020-4-15 8:00	QA	EMRO	283	3711	0	7	16	0.189	0.188
9364	2020-4-16 8:00	QA	EMRO	392	4103	0	7	17	0.171	0.170
9365	2020-4-17 8:00	QA	EMRO	560	4663	0	7	18	0.150	0.160
9366	2020-4-18 8:00	QA	EMRO	345	5008	1	8	19	0.160	0.152
9367	2020-4-19 8:00	QA	EMRO	440	5448	0	8	20	0.147	0.152
9368	2020-4-20 8:00	QA	EMRO	567	6015	1	9	21	0.150	0.145
9369	2020-4-21 8:00	QA	EMRO	518	6533	0	9	22	0.138	0.142
9370	2020-4-22 8:00	QA	EMRO	608	7141	1	10	23	0.140	0.136
9371	2020-4-23 8:00	QA	EMRO	623	7764	0	10	24	0.129	0.129
9372	2020-4-24 8:00	QA	EMRO	761	8525	0	10	25	0.117	0.118
9373	2020-4-25 8:00	QA	EMRO	833	9358	0	10	26	0.107	0.107
9374	2020-4-26 8:00	QA	EMRO	929	10287	0	10	27	0.097	0.098
9375	2020-4-27 8:00	QA	EMRO	957	11244	0	10	28	0.089	0.090
9376	2020-4-28 8:00	QA	EMRO	677	11921	0	10	29	0.084	0.084
9377	2020-4-29 8:00	QA	EMRO	643	12564	0	10	30	0.080	0.079
9378	2020-4-30 8:00	QA	EMRO	845	13409	0	10	31	0.075	0.080
9379	2020-5-1 8:00	QA	EMRO	687	14096	2	12	32	0.085	0.082
9380	2020-5-2 8:00	QA	EMRO	0	14096	0	12	33	0.085	0.085
9381										
9382	2020-1-19 8:00	KR	WPRO	1	1	0	0			
9383	2020-1-20 8:00	KR	WPRO	0	1	0	0			
9384	2020-1-21 8:00	KR	WPRO	0	1	0	0			
9385	2020-1-22 8:00	KR	WPRO	0	1	0	0			
9386	2020-1-23 8:00	KR	WPRO	0	1	0	0			
9387	2020-1-24 8:00	KR	WPRO	1	2	0	0			
9388	2020-1-25 8:00	KR	WPRO	0	2	0	0			
9389	2020-1-26 8:00	KR	WPRO	1	3	0	0			
9390	2020-1-27 8:00	KR	WPRO	1	4	0	0			
9391	2020-1-28 8:00	KR	WPRO	0	4	0	0			
9392	2020-1-29 8:00	KR	WPRO	0	4	0	0			
9393	2020-1-30 8:00	KR	WPRO	2	6	0	0			
9394	2020-1-31 8:00	KR	WPRO	5	11	0	0			
9395	2020-2-1 8:00	KR	WPRO	1	12	0	0			
9396	2020-2-2 8:00	KR	WPRO	3	15	0	0			

Table S1 Daily confirmed cases and daily deaths of COVID-19 in 214 nations from WHO

ID	Date and time	Country or region	Continent	Confirmed patients	Cumulative Confirmed patients	Daily dead patients	Daily cumulative dead patients	Lag days	Daily CFR	3DMA CFR
9397	2020-2-3 8:00	KR	WPRO	0	15	0	0			
9398	2020-2-4 8:00	KR	WPRO	1	16	0	0			
9399	2020-2-5 8:00	KR	WPRO	2	18	0	0			
9400	2020-2-6 8:00	KR	WPRO	5	23	0	0			
9401	2020-2-7 8:00	KR	WPRO	1	24	0	0			
9402	2020-2-8 8:00	KR	WPRO	0	24	0	0			
9403	2020-2-9 8:00	KR	WPRO	3	27	0	0			
9404	2020-2-10 8:00	KR	WPRO	0	27	0	0			
9405	2020-2-11 8:00	KR	WPRO	1	28	0	0			
9406	2020-2-12 8:00	KR	WPRO	0	28	0	0			
9407	2020-2-13 8:00	KR	WPRO	0	28	0	0			
9408	2020-2-14 8:00	KR	WPRO	0	28	0	0			
9409	2020-2-15 8:00	KR	WPRO	0	28	0	0			
9410	2020-2-16 8:00	KR	WPRO	1	29	0	0			
9411	2020-2-17 8:00	KR	WPRO	1	30	0	0			
9412	2020-2-18 8:00	KR	WPRO	1	31	0	0			
9413	2020-2-19 8:00	KR	WPRO	20	51	0	0			
9414	2020-2-20 8:00	KR	WPRO	53	104	1	1	0	0.962	0.726
9415	2020-2-21 8:00	KR	WPRO	100	204	0	1	1	0.490	0.638
9416	2020-2-22 8:00	KR	WPRO	229	433	1	2	2	0.462	0.428
9417	2020-2-23 8:00	KR	WPRO	169	602	0	2	3	0.332	0.545
9418	2020-2-24 8:00	KR	WPRO	231	833	5	7	4	0.840	0.732
9419	2020-2-25 8:00	KR	WPRO	144	977	3	10	5	1.024	0.939
9420	2020-2-26 8:00	KR	WPRO	284	1261	2	12	6	0.952	0.904
9421	2020-2-27 8:00	KR	WPRO	505	1766	1	13	7	0.736	0.748
9422	2020-2-28 8:00	KR	WPRO	571	2337	0	13	8	0.556	0.611
9423	2020-2-29 8:00	KR	WPRO	813	3150	4	17	9	0.540	0.526
9424	2020-3-1 8:00	KR	WPRO	586	3736	1	18	10	0.482	0.515
9425	2020-3-2 8:00	KR	WPRO	476	4212	4	22	11	0.522	0.529
9426	2020-3-3 8:00	KR	WPRO	600	4812	6	28	12	0.582	0.568
9427	2020-3-4 8:00	KR	WPRO	516	5328	4	32	13	0.601	0.596
9428	2020-3-5 8:00	KR	WPRO	438	5766	3	35	14	0.607	0.625
9429	2020-3-6 8:00	KR	WPRO	518	6284	7	42	15	0.668	0.642
9430	2020-3-7 8:00	KR	WPRO	483	6767	2	44	16	0.650	0.673
9431	2020-3-8 8:00	KR	WPRO	367	7134	6	50	17	0.701	0.681
9432	2020-3-9 8:00	KR	WPRO	248	7382	1	51	18	0.691	0.703

Table S1 Daily confirmed cases and daily deaths of COVID-19 in 214 nations from WHO

ID	Date and time	Country or region	Continent	Confirmed patients	Cumulative Confirmed patients	Daily dead patients	Daily cumulative dead patients	Lag days	Daily CFR	3DMA CFR
9433	2020-3-10 8:00	KR	WPRO	131	7513	3	54	19	0.719	0.728
9434	2020-3-11 8:00	KR	WPRO	242	7755	6	60	20	0.774	0.773
9435	2020-3-12 8:00	KR	WPRO	224	7979	6	66	21	0.827	0.814
9436	2020-3-13 8:00	KR	WPRO	0	7979	1	67	22	0.840	0.852
9437	2020-3-14 8:00	KR	WPRO	107	8086	5	72	23	0.890	0.883
9438	2020-3-15 8:00	KR	WPRO	76	8162	3	75	24	0.919	0.907
9439	2020-3-16 8:00	KR	WPRO	74	8236	0	75	25	0.911	0.934
9440	2020-3-17 8:00	KR	WPRO	84	8320	6	81	26	0.974	0.961
9441	2020-3-18 8:00	KR	WPRO	93	8413	3	84	27	0.998	1.019
9442	2020-3-19 8:00	KR	WPRO	239	8652	10	94	28	1.086	1.081
9443	2020-3-20 8:00	KR	WPRO	147	8799	8	102	29	1.159	1.138
9444	2020-3-21 8:00	KR	WPRO	98	8897	2	104	30	1.169	1.166
9445	2020-3-22 8:00	KR	WPRO	0	8897	0	104	31	1.169	1.192
9446	2020-3-23 8:00	KR	WPRO	64	8961	7	111	32	1.239	1.245
9447	2020-3-24 8:00	KR	WPRO	76	9037	9	120	33	1.328	1.315
9448	2020-3-25 8:00	KR	WPRO	100	9137	6	126	34	1.379	1.375
9449	2020-3-26 8:00	KR	WPRO	104	9241	5	131	35	1.418	1.429
9450	2020-3-27 8:00	KR	WPRO	91	9332	8	139	36	1.489	1.475
9451	2020-3-28 8:00	KR	WPRO	146	9478	5	144	37	1.519	1.532
9452	2020-3-29 8:00	KR	WPRO	105	9583	8	152	38	1.586	1.580
9453	2020-3-30 8:00	KR	WPRO	78	9661	6	158	39	1.635	1.626
9454	2020-3-31 8:00	KR	WPRO	125	9786	4	162	40	1.655	1.653
9455	2020-4-1 8:00	KR	WPRO	101	9887	3	165	41	1.669	1.673
9456	2020-4-2 8:00	KR	WPRO	89	9976	4	169	42	1.694	1.697
9457	2020-4-3 8:00	KR	WPRO	86	10062	5	174	43	1.729	1.722
9458	2020-4-4 8:00	KR	WPRO	94	10156	3	177	44	1.743	1.753
9459	2020-4-5 8:00	KR	WPRO	81	10237	6	183	45	1.788	1.780
9460	2020-4-6 8:00	KR	WPRO	47	10284	3	186	46	1.809	1.818
9461	2020-4-7 8:00	KR	WPRO	47	10331	6	192	47	1.858	1.864
9462	2020-4-8 8:00	KR	WPRO	53	10384	8	200	48	1.926	1.914
9463	2020-4-9 8:00	KR	WPRO	39	10423	4	204	49	1.957	1.958
9464	2020-4-10 8:00	KR	WPRO	27	10450	4	208	50	1.990	1.987
9465	2020-4-11 8:00	KR	WPRO	30	10480	3	211	51	2.013	2.013
9466	2020-4-12 8:00	KR	WPRO	32	10512	3	214	52	2.036	2.036
9467	2020-4-13 8:00	KR	WPRO	25	10537	3	217	53	2.059	2.066
9468	2020-4-14 8:00	KR	WPRO	27	10564	5	222	54	2.101	2.095

Table S1 Daily confirmed cases and daily deaths of COVID-19 in 214 nations from WHO

ID	Date and time	Country or region	Continent	Confirmed patients	Cumulative Confirmed patients	Daily dead patients	Daily cumulative dead patients	Lag days	Daily CFR	3DMA CFR
9469	2020-4-15 8:00	KR	WPRO	27	10591	3	225	55	2.124	2.128
9470	2020-4-16 8:00	KR	WPRO	22	10613	4	229	56	2.158	2.148
9471	2020-4-17 8:00	KR	WPRO	22	10635	1	230	57	2.163	2.166
9472	2020-4-18 8:00	KR	WPRO	18	10653	2	232	58	2.178	2.178
9473	2020-4-19 8:00	KR	WPRO	8	10661	2	234	59	2.195	2.195
9474	2020-4-20 8:00	KR	WPRO	13	10674	2	236	60	2.211	2.208
9475	2020-4-21 8:00	KR	WPRO	9	10683	1	237	61	2.218	2.218
9476	2020-4-22 8:00	KR	WPRO	11	10694	1	238	62	2.226	2.229
9477	2020-4-23 8:00	KR	WPRO	8	10702	2	240	63	2.243	2.236
9478	2020-4-24 8:00	KR	WPRO	6	10708	0	240	64	2.241	2.241
9479	2020-4-25 8:00	KR	WPRO	10	10718	0	240	65	2.239	2.245
9480	2020-4-26 8:00	KR	WPRO	10	10728	2	242	66	2.256	2.253
9481	2020-4-27 8:00	KR	WPRO	10	10738	1	243	67	2.263	2.263
9482	2020-4-28 8:00	KR	WPRO	14	10752	1	244	68	2.269	2.273
9483	2020-4-29 8:00	KR	WPRO	9	10761	2	246	69	2.286	2.283
9484	2020-4-30 8:00	KR	WPRO	4	10765	1	247	70	2.294	2.294
9485	2020-5-1 8:00	KR	WPRO	9	10774	1	248	71	2.302	2.305
9486	2020-5-2 8:00	KR	WPRO	6	10780	2	250	72	2.319	2.310
9487										
9488	2020-3-7 8:00	MD	EURO	1	1	0	0			
9489	2020-3-8 8:00	MD	EURO	0	1	0	0			
9490	2020-3-9 8:00	MD	EURO	0	1	0	0			
9491	2020-3-10 8:00	MD	EURO	2	3	0	0			
9492	2020-3-11 8:00	MD	EURO	0	3	0	0			
9493	2020-3-12 8:00	MD	EURO	1	4	0	0			
9494	2020-3-13 8:00	MD	EURO	4	8	0	0			
9495	2020-3-14 8:00	MD	EURO	4	12	0	0			
9496	2020-3-15 8:00	MD	EURO	11	23	0	0			
9497	2020-3-16 8:00	MD	EURO	6	29	0	0			
9498	2020-3-17 8:00	MD	EURO	1	30	0	0			
9499	2020-3-18 8:00	MD	EURO	6	36	0	0			
9500	2020-3-19 8:00	MD	EURO	13	49	0	0			
9501	2020-3-20 8:00	MD	EURO	17	66	1	1	0	1.515	1.383
9502	2020-3-21 8:00	MD	EURO	14	80	0	1	1	1.250	1.338
9503	2020-3-22 8:00	MD	EURO	0	80	0	1	2	1.250	1.188
9504	2020-3-23 8:00	MD	EURO	14	94	0	1	3	1.064	1.077

Table S1 Daily confirmed cases and daily deaths of COVID-19 in 214 nations from WHO

ID	Date and time	Country or region	Continent	Confirmed patients	Cumulative Confirmed patients	Daily dead patients	Daily cumulative dead patients	Lag days	Daily CFR	3DMA CFR
9505	2020-3-24 8:00	MD	EURO	15	109	0	1	4	0.917	0.927
9506	2020-3-25 8:00	MD	EURO	16	125	0	1	5	0.800	0.796
9507	2020-3-26 8:00	MD	EURO	24	149	0	1	6	0.671	0.867
9508	2020-3-27 8:00	MD	EURO	28	177	1	2	7	1.130	0.935
9509	2020-3-28 8:00	MD	EURO	22	199	0	2	8	1.005	1.000
9510	2020-3-29 8:00	MD	EURO	32	231	0	2	9	0.866	0.877
9511	2020-3-30 8:00	MD	EURO	32	263	0	2	10	0.760	0.766
9512	2020-3-31 8:00	MD	EURO	35	298	0	2	11	0.671	0.760
9513	2020-4-1 8:00	MD	EURO	55	353	1	3	12	0.850	0.901
9514	2020-4-2 8:00	MD	EURO	70	423	2	5	13	1.182	1.129
9515	2020-4-3 8:00	MD	EURO	168	591	3	8	14	1.354	1.353
9516	2020-4-4 8:00	MD	EURO	0	591	1	9	15	1.523	1.491
9517	2020-4-5 8:00	MD	EURO	161	752	3	12	16	1.596	1.618
9518	2020-4-6 8:00	MD	EURO	112	864	3	15	17	1.736	1.767
9519	2020-4-7 8:00	MD	EURO	101	965	4	19	18	1.969	1.929
9520	2020-4-8 8:00	MD	EURO	91	1056	3	22	19	2.083	2.146
9521	2020-4-9 8:00	MD	EURO	118	1174	6	28	20	2.385	2.239
9522	2020-4-10 8:00	MD	EURO	115	1289	1	29	21	2.250	2.217
9523	2020-4-11 8:00	MD	EURO	149	1438	0	29	22	2.017	2.063
9524	2020-4-12 8:00	MD	EURO	122	1560	1	30	23	1.923	1.975
9525	2020-4-13 8:00	MD	EURO	102	1662	3	33	24	1.986	2.004
9526	2020-4-14 8:00	MD	EURO	50	1712	3	36	25	2.103	2.069
9527	2020-4-15 8:00	MD	EURO	222	1934	5	41	26	2.120	2.156
9528	2020-4-16 8:00	MD	EURO	115	2049	5	46	27	2.245	2.291
9529	2020-4-17 8:00	MD	EURO	105	2154	8	54	28	2.507	2.408
9530	2020-4-18 8:00	MD	EURO	110	2264	2	56	29	2.473	2.511
9531	2020-4-19 8:00	MD	EURO	87	2351	4	60	30	2.552	2.579
9532	2020-4-20 8:00	MD	EURO	121	2472	7	67	31	2.710	2.670
9533	2020-4-21 8:00	MD	EURO	76	2548	3	70	32	2.747	2.741
9534	2020-4-22 8:00	MD	EURO	93	2641	3	73	33	2.764	2.749
9535	2020-4-23 8:00	MD	EURO	137	2778	3	76	34	2.736	2.745
9536	2020-4-24 8:00	MD	EURO	148	2926	4	80	35	2.734	2.756
9537	2020-4-25 8:00	MD	EURO	184	3110	7	87	36	2.797	2.792
9538	2020-4-26 8:00	MD	EURO	194	3304	7	94	37	2.845	2.820
9539	2020-4-27 8:00	MD	EURO	104	3408	2	96	38	2.817	2.874
9540	2020-4-28 8:00	MD	EURO	73	3481	7	103	39	2.959	2.924

Table S1 Daily confirmed cases and daily deaths of COVID-19 in 214 nations from WHO

ID	Date and time	Country or region	Continent	Confirmed patients	Cumulative Confirmed patients	Daily dead patients	Daily cumulative dead patients	Lag days	Daily CFR	3DMA CFR
9541	2020-4-29 8:00	MD	EURO	157	3638	6	109	40	2.996	3.010
9542	2020-4-30 8:00	MD	EURO	133	3771	7	116	41	3.076	3.042
9543	2020-5-1 8:00	MD	EURO	126	3897	3	119	42	3.054	3.065
9544	2020-5-2 8:00	MD	EURO	83	3980	3	122	43	3.065	3.059
9545										
9546	2020-3-13 8:00	RE	AFRO	3	3	0	0			
9547	2020-3-14 8:00	RE	AFRO	3	6	0	0			
9548	2020-3-15 8:00	RE	AFRO	3	9	0	0			
9549	2020-3-16 8:00	RE	AFRO	0	9	0	0			
9550	2020-3-17 8:00	RE	AFRO	0	9	0	0			
9551	2020-3-18 8:00	RE	AFRO	3	12	0	0			
9552	2020-3-19 8:00	RE	AFRO	3	15	0	0			
9553	2020-3-20 8:00	RE	AFRO	13	28	0	0			
9554	2020-3-21 8:00	RE	AFRO	19	47	0	0			
9555	2020-3-22 8:00	RE	AFRO	0	47	0	0			
9556	2020-3-23 8:00	RE	AFRO	17	64	0	0			
9557	2020-3-24 8:00	RE	AFRO	7	71	0	0			
9558	2020-3-25 8:00	RE	AFRO	12	83	0	0			
9559	2020-3-26 8:00	RE	AFRO	11	94	0	0			
9560	2020-3-27 8:00	RE	AFRO	41	135	0	0			
9561	2020-3-28 8:00	RE	AFRO	8	143	0	0			
9562	2020-3-29 8:00	RE	AFRO	0	143	0	0			
9563	2020-3-30 8:00	RE	AFRO	64	207	0	0			
9564	2020-3-31 8:00	RE	AFRO	0	207	0	0			
9565	2020-4-1 8:00	RE	AFRO	40	247	0	0			
9566	2020-4-2 8:00	RE	AFRO	34	281	0	0			
9567	2020-4-3 8:00	RE	AFRO	27	308	0	0			
9568	2020-4-4 8:00	RE	AFRO	13	321	0	0			
9569	2020-4-5 8:00	RE	AFRO	13	334	0	0			
9570	2020-4-6 8:00	RE	AFRO	10	344	0	0			
9571	2020-4-7 8:00	RE	AFRO	5	349	0	0			
9572	2020-4-8 8:00	RE	AFRO	9	358	0	0			
9573	2020-4-9 8:00	RE	AFRO	4	362	0	0			
9574	2020-4-10 8:00	RE	AFRO	14	376	0	0			
9575	2020-4-11 8:00	RE	AFRO	6	382	0	0			
9576	2020-4-12 8:00	RE	AFRO	6	388	0	0			

Table S1 Daily confirmed cases and daily deaths of COVID-19 in 214 nations from WHO

ID	Date and time	Country or region	Continent	Confirmed patients	Cumulative Confirmed patients	Daily dead patients	Daily cumulative dead patients	Lag days	Daily CFR	3DMA CFR
9577	2020-4-13 8:00	RE	AFRO	1	389	0	0			
9578	2020-4-14 8:00	RE	AFRO	1	390	0	0			
9579	2020-4-15 8:00	RE	AFRO	0	390	0	0			
9580	2020-4-16 8:00	RE	AFRO	0	390	0	0			
9581	2020-4-17 8:00	RE	AFRO	4	394	0	0			
9582	2020-4-18 8:00	RE	AFRO	8	402	0	0			
9583	2020-4-19 8:00	RE	AFRO	5	407	0	0			
9584	2020-4-20 8:00	RE	AFRO	1	408	0	0			
9585	2020-4-21 8:00	RE	AFRO	0	408	0	0			
9586	2020-4-22 8:00	RE	AFRO	2	410	0	0			
9587	2020-4-23 8:00	RE	AFRO	0	410	0	0			
9588	2020-4-24 8:00	RE	AFRO	1	411	0	0			
9589	2020-4-25 8:00	RE	AFRO	0	411	0	0			
9590	2020-4-26 8:00	RE	AFRO	0	411	0	0			
9591	2020-4-27 8:00	RE	AFRO	5	416	0	0			
9592	2020-4-28 8:00	RE	AFRO	1	417	0	0			
9593	2020-4-29 8:00	RE	AFRO	0	417	0	0			
9594	2020-4-30 8:00	RE	AFRO	3	420	0	0			
9595	2020-5-1 8:00	RE	AFRO	0	420	0	0			
9596	2020-5-2 8:00	RE	AFRO	0	420	0	0			
9597										
9598	2020-2-26 8:00	RO	EURO	1	1	0	0			
9599	2020-2-27 8:00	RO	EURO	0	1	0	0			
9600	2020-2-28 8:00	RO	EURO	2	3	0	0			
9601	2020-2-29 8:00	RO	EURO	0	3	0	0			
9602	2020-3-1 8:00	RO	EURO	0	3	0	0			
9603	2020-3-2 8:00	RO	EURO	0	3	0	0			
9604	2020-3-3 8:00	RO	EURO	1	4	0	0			
9605	2020-3-4 8:00	RO	EURO	0	4	0	0			
9606	2020-3-5 8:00	RO	EURO	2	6	0	0			
9607	2020-3-6 8:00	RO	EURO	1	7	0	0			
9608	2020-3-7 8:00	RO	EURO	2	9	0	0			
9609	2020-3-8 8:00	RO	EURO	6	15	0	0			
9610	2020-3-9 8:00	RO	EURO	0	15	0	0			
9611	2020-3-10 8:00	RO	EURO	2	17	0	0			
9612	2020-3-11 8:00	RO	EURO	14	31	0	0			

Table S1 Daily confirmed cases and daily deaths of COVID-19 in 214 nations from WHO

ID	Date and time	Country or region	Continent	Confirmed patients	Cumulative Confirmed patients	Daily dead patients	Daily cumulative dead patients	Lag days	Daily CFR	3DMA CFR
9613	2020-3-12 8:00	RO	EURO	18	49	0	0			
9614	2020-3-13 8:00	RO	EURO	15	64	0	0			
9615	2020-3-14 8:00	RO	EURO	49	113	0	0			
9616	2020-3-15 8:00	RO	EURO	10	123	0	0			
9617	2020-3-16 8:00	RO	EURO	35	158	0	0			
9618	2020-3-17 8:00	RO	EURO	26	184	0	0			
9619	2020-3-18 8:00	RO	EURO	62	246	0	0			
9620	2020-3-19 8:00	RO	EURO	14	260	0	0			
9621	2020-3-20 8:00	RO	EURO	48	308	0	0			
9622	2020-3-21 8:00	RO	EURO	59	367	0	0			
9623	2020-3-22 8:00	RO	EURO	0	367	0	0			
9624	2020-3-23 8:00	RO	EURO	66	433	2	2	0	0.462	0.839
9625	2020-3-24 8:00	RO	EURO	143	576	5	7	1	1.215	1.040
9626	2020-3-25 8:00	RO	EURO	186	762	4	11	2	1.444	1.365
9627	2020-3-26 8:00	RO	EURO	144	906	2	13	3	1.435	1.510
9628	2020-3-27 8:00	RO	EURO	123	1029	4	17	4	1.652	1.648
9629	2020-3-28 8:00	RO	EURO	263	1292	7	24	5	1.858	1.836
9630	2020-3-29 8:00	RO	EURO	160	1452	5	29	6	1.997	2.043
9631	2020-3-30 8:00	RO	EURO	308	1760	11	40	7	2.273	2.175
9632	2020-3-31 8:00	RO	EURO	192	1952	4	44	8	2.254	2.533
9633	2020-4-1 8:00	RO	EURO	293	2245	25	69	9	3.073	2.928
9634	2020-4-2 8:00	RO	EURO	215	2460	16	85	10	3.455	3.321
9635	2020-4-3 8:00	RO	EURO	278	2738	9	94	11	3.433	3.689
9636	2020-4-4 8:00	RO	EURO	445	3183	39	133	12	4.178	3.838
9637	2020-4-5 8:00	RO	EURO	430	3613	8	141	13	3.903	3.970
9638	2020-4-6 8:00	RO	EURO	251	3864	7	148	14	3.830	3.868
9639	2020-4-7 8:00	RO	EURO	193	4057	9	157	15	3.870	3.940
9640	2020-4-8 8:00	RO	EURO	360	4417	25	182	16	4.120	4.127
9641	2020-4-9 8:00	RO	EURO	344	4761	27	209	17	4.390	4.304
9642	2020-4-10 8:00	RO	EURO	441	5202	20	229	18	4.402	4.498
9643	2020-4-11 8:00	RO	EURO	265	5467	28	257	19	4.701	4.604
9644	2020-4-12 8:00	RO	EURO	523	5990	25	282	20	4.708	4.755
9645	2020-4-13 8:00	RO	EURO	310	6300	24	306	21	4.857	4.786
9646	2020-4-14 8:00	RO	EURO	333	6633	12	318	22	4.794	4.884
9647	2020-4-15 8:00	RO	EURO	246	6879	26	344	23	5.001	4.983
9648	2020-4-16 8:00	RO	EURO	337	7216	28	372	24	5.155	5.059

Table S1 Daily confirmed cases and daily deaths of COVID-19 in 214 nations from WHO

ID	Date and time	Country or region	Continent	Confirmed patients	Cumulative Confirmed patients	Daily dead patients	Daily cumulative dead patients	Lag days	Daily CFR	3DMA CFR
9649	2020-4-17 8:00	RO	EURO	491	7707	15	387	25	5.021	5.045
9650	2020-4-18 8:00	RO	EURO	360	8067	13	400	26	4.958	4.978
9651	2020-4-19 8:00	RO	EURO	351	8418	17	417	27	4.954	4.958
9652	2020-4-20 8:00	RO	EURO	328	8746	17	434	28	4.962	4.988
9653	2020-4-21 8:00	RO	EURO	190	8936	17	451	29	5.047	5.078
9654	2020-4-22 8:00	RO	EURO	306	9242	32	483	30	5.226	5.168
9655	2020-4-23 8:00	RO	EURO	468	9710	25	508	31	5.232	5.226
9656	2020-4-24 8:00	RO	EURO	386	10096	19	527	32	5.220	5.250
9657	2020-4-25 8:00	RO	EURO	321	10417	25	552	33	5.299	5.309
9658	2020-4-26 8:00	RO	EURO	218	10635	23	575	34	5.407	5.405
9659	2020-4-27 8:00	RO	EURO	401	11036	33	608	35	5.509	5.494
9660	2020-4-28 8:00	RO	EURO	303	11339	23	631	36	5.565	5.557
9661	2020-4-29 8:00	RO	EURO	277	11616	19	650	37	5.596	5.599
9662	2020-4-30 8:00	RO	EURO	362	11978	25	675	38	5.635	5.696
9663	2020-5-1 8:00	RO	EURO	262	12240	42	717	39	5.858	5.804
9664	2020-5-2 8:00	RO	EURO	327	12567	27	744	40	5.920	5.889
9665										
9666	2020-1-31 8:00	RU	EURO	2	2	0	0			
9667	2020-2-1 8:00	RU	EURO	0	2	0	0			
9668	2020-2-2 8:00	RU	EURO	0	2	0	0			
9669	2020-2-3 8:00	RU	EURO	0	2	0	0			
9670	2020-2-4 8:00	RU	EURO	0	2	0	0			
9671	2020-2-5 8:00	RU	EURO	0	2	0	0			
9672	2020-2-6 8:00	RU	EURO	0	2	0	0			
9673	2020-2-7 8:00	RU	EURO	0	2	0	0			
9674	2020-2-8 8:00	RU	EURO	0	2	0	0			
9675	2020-2-9 8:00	RU	EURO	0	2	0	0			
9676	2020-2-10 8:00	RU	EURO	0	2	0	0			
9677	2020-2-11 8:00	RU	EURO	0	2	0	0			
9678	2020-2-12 8:00	RU	EURO	0	2	0	0			
9679	2020-2-13 8:00	RU	EURO	0	2	0	0			
9680	2020-2-14 8:00	RU	EURO	0	2	0	0			
9681	2020-2-15 8:00	RU	EURO	0	2	0	0			
9682	2020-2-16 8:00	RU	EURO	0	2	0	0			
9683	2020-2-17 8:00	RU	EURO	0	2	0	0			
9684	2020-2-18 8:00	RU	EURO	0	2	0	0			

Table S1 Daily confirmed cases and daily deaths of COVID-19 in 214 nations from WHO

ID	Date and time	Country or region	Continent	Confirmed patients	Cumulative Confirmed patients	Daily dead patients	Daily cumulative dead patients	Lag days	Daily CFR	3DMA CFR
9685	2020-2-19 8:00	RU	EURO	0	2	0	0			
9686	2020-2-20 8:00	RU	EURO	0	2	0	0			
9687	2020-2-21 8:00	RU	EURO	0	2	0	0			
9688	2020-2-22 8:00	RU	EURO	0	2	0	0			
9689	2020-2-23 8:00	RU	EURO	0	2	0	0			
9690	2020-2-24 8:00	RU	EURO	0	2	0	0			
9691	2020-2-25 8:00	RU	EURO	0	2	0	0			
9692	2020-2-26 8:00	RU	EURO	0	2	0	0			
9693	2020-2-27 8:00	RU	EURO	0	2	0	0			
9694	2020-2-28 8:00	RU	EURO	0	2	0	0			
9695	2020-2-29 8:00	RU	EURO	0	2	0	0			
9696	2020-3-1 8:00	RU	EURO	0	2	0	0			
9697	2020-3-2 8:00	RU	EURO	1	3	0	0			
9698	2020-3-3 8:00	RU	EURO	0	3	0	0			
9699	2020-3-4 8:00	RU	EURO	0	3	0	0			
9700	2020-3-5 8:00	RU	EURO	1	4	0	0			
9701	2020-3-6 8:00	RU	EURO	3	7	0	0			
9702	2020-3-7 8:00	RU	EURO	0	7	0	0			
9703	2020-3-8 8:00	RU	EURO	0	7	0	0			
9704	2020-3-9 8:00	RU	EURO	0	7	0	0			
9705	2020-3-10 8:00	RU	EURO	0	7	0	0			
9706	2020-3-11 8:00	RU	EURO	0	7	0	0			
9707	2020-3-12 8:00	RU	EURO	27	34	0	0			
9708	2020-3-13 8:00	RU	EURO	0	34	0	0			
9709	2020-3-14 8:00	RU	EURO	0	34	0	0			
9710	2020-3-15 8:00	RU	EURO	0	34	0	0			
9711	2020-3-16 8:00	RU	EURO	59	93	0	0			
9712	2020-3-17 8:00	RU	EURO	0	93	0	0			
9713	2020-3-18 8:00	RU	EURO	54	147	0	0			
9714	2020-3-19 8:00	RU	EURO	52	199	0	0			
9715	2020-3-20 8:00	RU	EURO	54	253	0	0			
9716	2020-3-21 8:00	RU	EURO	53	306	0	0			
9717	2020-3-22 8:00	RU	EURO	0	306	0	0			
9718	2020-3-23 8:00	RU	EURO	132	438	0	0			
9719	2020-3-24 8:00	RU	EURO	0	438	0	0			
9720	2020-3-25 8:00	RU	EURO	220	658	0	0			

Table S1 Daily confirmed cases and daily deaths of COVID-19 in 214 nations from WHO

ID	Date and time	Country or region	Continent	Confirmed patients	Cumulative Confirmed patients	Daily dead patients	Daily cumulative dead patients	Lag days	Daily CFR	3DMA CFR
9721	2020-3-26 8:00	RU	EURO	182	840	2	2	0	0.238	0.264
9722	2020-3-27 8:00	RU	EURO	196	1036	1	3	1	0.290	0.281
9723	2020-3-28 8:00	RU	EURO	228	1264	1	4	2	0.316	0.376
9724	2020-3-29 8:00	RU	EURO	270	1534	4	8	3	0.522	0.475
9725	2020-3-30 8:00	RU	EURO	0	1534	1	9	4	0.587	0.533
9726	2020-3-31 8:00	RU	EURO	303	1837	0	9	5	0.490	0.601
9727	2020-4-1 8:00	RU	EURO	500	2337	8	17	6	0.727	0.694
9728	2020-4-2 8:00	RU	EURO	440	2777	7	24	7	0.864	0.812
9729	2020-4-3 8:00	RU	EURO	771	3548	6	30	8	0.846	0.843
9730	2020-4-4 8:00	RU	EURO	601	4149	4	34	9	0.819	0.858
9731	2020-4-5 8:00	RU	EURO	582	4731	9	43	10	0.909	0.854
9732	2020-4-6 8:00	RU	EURO	658	5389	2	45	11	0.835	0.828
9733	2020-4-7 8:00	RU	EURO	954	6343	2	47	12	0.741	0.783
9734	2020-4-8 8:00	RU	EURO	1154	7497	11	58	13	0.774	0.755
9735	2020-4-9 8:00	RU	EURO	2634	10131	18	76	14	0.750	0.771
9736	2020-4-10 8:00	RU	EURO	1786	11917	18	94	15	0.789	0.773
9737	2020-4-11 8:00	RU	EURO	1667	13584	12	106	16	0.780	0.798
9738	2020-4-12 8:00	RU	EURO	2186	15770	24	130	17	0.824	0.804
9739	2020-4-13 8:00	RU	EURO	2558	18328	18	148	18	0.808	0.812
9740	2020-4-14 8:00	RU	EURO	2774	21102	22	170	19	0.806	0.807
9741	2020-4-15 8:00	RU	EURO	3388	24490	28	198	20	0.808	0.815
9742	2020-4-16 8:00	RU	EURO	3448	27938	34	232	21	0.830	0.831
9743	2020-4-17 8:00	RU	EURO	4070	32008	41	273	22	0.853	0.845
9744	2020-4-18 8:00	RU	EURO	4785	36793	40	313	23	0.851	0.849
9745	2020-4-19 8:00	RU	EURO	6060	42853	48	361	24	0.842	0.845
9746	2020-4-20 8:00	RU	EURO	0	42853	0	361	25	0.842	0.850
9747	2020-4-21 8:00	RU	EURO	9910	52763	95	456	26	0.864	0.864
9748	2020-4-22 8:00	RU	EURO	5236	57999	57	513	27	0.884	0.878
9749	2020-4-23 8:00	RU	EURO	4774	62773	42	555	28	0.884	0.888
9750	2020-4-24 8:00	RU	EURO	5849	68622	60	615	29	0.896	0.892
9751	2020-4-25 8:00	RU	EURO	0	68622	0	615	30	0.896	0.902
9752	2020-4-26 8:00	RU	EURO	5966	74588	66	681	31	0.913	0.907
9753	2020-4-27 8:00	RU	EURO	12559	87147	113	794	32	0.911	0.917
9754	2020-4-28 8:00	RU	EURO	6411	93558	73	867	33	0.927	0.939
9755	2020-4-29 8:00	RU	EURO	5841	99399	105	972	34	0.978	0.971
9756	2020-4-30 8:00	RU	EURO	7099	106498	101	1073	35	1.008	1.002

Table S1 Daily confirmed cases and daily deaths of COVID-19 in 214 nations from WHO

ID	Date and time	Country or region	Continent	Confirmed patients	Cumulative Confirmed patients	Daily dead patients	Daily cumulative dead patients	Lag days	Daily CFR	3DMA CFR
9757	2020-5-1 8:00	RU	EURO	7933	114431	96	1169	36	1.022	1.005
9758	2020-5-2 8:00	RU	EURO	9623	124054	53	1222	37	0.985	1.003
9759										
9760	2020-3-15 8:00	RW	AFRO	5	5	0	0			
9761	2020-3-16 8:00	RW	AFRO	0	5	0	0			
9762	2020-3-17 8:00	RW	AFRO	2	7	0	0			
9763	2020-3-18 8:00	RW	AFRO	4	11	0	0			
9764	2020-3-19 8:00	RW	AFRO	0	11	0	0			
9765	2020-3-20 8:00	RW	AFRO	0	11	0	0			
9766	2020-3-21 8:00	RW	AFRO	6	17	0	0			
9767	2020-3-22 8:00	RW	AFRO	0	17	0	0			
9768	2020-3-23 8:00	RW	AFRO	0	17	0	0			
9769	2020-3-24 8:00	RW	AFRO	19	36	0	0			
9770	2020-3-25 8:00	RW	AFRO	4	40	0	0			
9771	2020-3-26 8:00	RW	AFRO	1	41	0	0			
9772	2020-3-27 8:00	RW	AFRO	9	50	0	0			
9773	2020-3-28 8:00	RW	AFRO	4	54	0	0			
9774	2020-3-29 8:00	RW	AFRO	6	60	0	0			
9775	2020-3-30 8:00	RW	AFRO	10	70	0	0			
9776	2020-3-31 8:00	RW	AFRO	0	70	0	0			
9777	2020-4-1 8:00	RW	AFRO	5	75	0	0			
9778	2020-4-2 8:00	RW	AFRO	7	82	0	0			
9779	2020-4-3 8:00	RW	AFRO	2	84	0	0			
9780	2020-4-4 8:00	RW	AFRO	5	89	0	0			
9781	2020-4-5 8:00	RW	AFRO	13	102	0	0			
9782	2020-4-6 8:00	RW	AFRO	0	102	0	0			
9783	2020-4-7 8:00	RW	AFRO	2	104	0	0			
9784	2020-4-8 8:00	RW	AFRO	1	105	0	0			
9785	2020-4-9 8:00	RW	AFRO	5	110	0	0			
9786	2020-4-10 8:00	RW	AFRO	3	113	0	0			
9787	2020-4-11 8:00	RW	AFRO	5	118	0	0			
9788	2020-4-12 8:00	RW	AFRO	2	120	0	0			
9789	2020-4-13 8:00	RW	AFRO	6	126	0	0			
9790	2020-4-14 8:00	RW	AFRO	1	127	0	0			
9791	2020-4-15 8:00	RW	AFRO	7	134	0	0			
9792	2020-4-16 8:00	RW	AFRO	2	136	0	0			

Table S1 Daily confirmed cases and daily deaths of COVID-19 in 214 nations from WHO

ID	Date and time	Country or region	Continent	Confirmed patients	Cumulative Confirmed patients	Daily dead patients	Daily cumulative dead patients	Lag days	Daily CFR	3DMA CFR
9793	2020-4-17 8:00	RW	AFRO	2	138	0	0			
9794	2020-4-18 8:00	RW	AFRO	0	138	0	0			
9795	2020-4-19 8:00	RW	AFRO	6	144	0	0			
9796	2020-4-20 8:00	RW	AFRO	3	147	0	0			
9797	2020-4-21 8:00	RW	AFRO	0	147	0	0			
9798	2020-4-22 8:00	RW	AFRO	3	150	0	0			
9799	2020-4-23 8:00	RW	AFRO	3	153	0	0			
9800	2020-4-24 8:00	RW	AFRO	1	154	0	0			
9801	2020-4-25 8:00	RW	AFRO	22	176	0	0			
9802	2020-4-26 8:00	RW	AFRO	7	183	0	0			
9803	2020-4-27 8:00	RW	AFRO	8	191	0	0			
9804	2020-4-28 8:00	RW	AFRO	16	207	0	0			
9805	2020-4-29 8:00	RW	AFRO	5	212	0	0			
9806	2020-4-30 8:00	RW	AFRO	13	225	0	0			
9807	2020-5-1 8:00	RW	AFRO	18	243	0	0			
9808	2020-5-2 8:00	RW	AFRO	6	249	0	0			
9809										
9810	2020-3-2 8:00	BL	AMRO	1	1	0	0			
9811	2020-3-3 8:00	BL	AMRO	0	1	0	0			
9812	2020-3-4 8:00	BL	AMRO	0	1	0	0			
9813	2020-3-5 8:00	BL	AMRO	0	1	0	0			
9814	2020-3-6 8:00	BL	AMRO	0	1	0	0			
9815	2020-3-7 8:00	BL	AMRO	0	1	0	0			
9816	2020-3-8 8:00	BL	AMRO	0	1	0	0			
9817	2020-3-9 8:00	BL	AMRO	0	1	0	0			
9818	2020-3-10 8:00	BL	AMRO	0	1	0	0			
9819	2020-3-11 8:00	BL	AMRO	0	1	0	0			
9820	2020-3-12 8:00	BL	AMRO	0	1	0	0			
9821	2020-3-13 8:00	BL	AMRO	0	1	0	0			
9822	2020-3-14 8:00	BL	AMRO	0	1	0	0			
9823	2020-3-15 8:00	BL	AMRO	2	3	0	0			
9824	2020-3-16 8:00	BL	AMRO	0	3	0	0			
9825	2020-3-17 8:00	BL	AMRO	0	3	0	0			
9826	2020-3-18 8:00	BL	AMRO	0	3	0	0			
9827	2020-3-19 8:00	BL	AMRO	0	3	0	0			
9828	2020-3-20 8:00	BL	AMRO	0	3	0	0			

Table S1 Daily confirmed cases and daily deaths of COVID-19 in 214 nations from WHO

ID	Date and time	Country or region	Continent	Confirmed patients	Cumulative Confirmed patients	Daily dead patients	Daily cumulative dead patients	Lag days	Daily CFR	3DMA CFR
9829	2020-3-21 8:00	BL	AMRO	0	3	0	0			
9830	2020-3-22 8:00	BL	AMRO	0	3	0	0			
9831	2020-3-23 8:00	BL	AMRO	0	3	0	0			
9832	2020-3-24 8:00	BL	AMRO	0	3	0	0			
9833	2020-3-25 8:00	BL	AMRO	0	3	0	0			
9834	2020-3-26 8:00	BL	AMRO	0	3	0	0			
9835	2020-3-27 8:00	BL	AMRO	0	3	0	0			
9836	2020-3-28 8:00	BL	AMRO	2	5	0	0			
9837	2020-3-29 8:00	BL	AMRO	0	5	0	0			
9838	2020-3-30 8:00	BL	AMRO	0	5	0	0			
9839	2020-3-31 8:00	BL	AMRO	1	6	0	0			
9840	2020-4-1 8:00	BL	AMRO	0	6	0	0			
9841	2020-4-2 8:00	BL	AMRO	0	6	0	0			
9842	2020-4-3 8:00	BL	AMRO	0	6	0	0			
9843	2020-4-4 8:00	BL	AMRO	0	6	0	0			
9844	2020-4-5 8:00	BL	AMRO	0	6	0	0			
9845	2020-4-6 8:00	BL	AMRO	0	6	0	0			
9846	2020-4-7 8:00	BL	AMRO	0	6	0	0			
9847	2020-4-8 8:00	BL	AMRO	0	6	0	0			
9848	2020-4-9 8:00	BL	AMRO	0	6	0	0			
9849	2020-4-10 8:00	BL	AMRO	0	6	0	0			
9850	2020-4-11 8:00	BL	AMRO	0	6	0	0			
9851	2020-4-12 8:00	BL	AMRO	0	6	0	0			
9852	2020-4-13 8:00	BL	AMRO	0	6	0	0			
9853	2020-4-14 8:00	BL	AMRO	0	6	0	0			
9854	2020-4-15 8:00	BL	AMRO	0	6	0	0			
9855	2020-4-16 8:00	BL	AMRO	0	6	0	0			
9856	2020-4-17 8:00	BL	AMRO	0	6	0	0			
9857	2020-4-18 8:00	BL	AMRO	0	6	0	0			
9858	2020-4-19 8:00	BL	AMRO	0	6	0	0			
9859	2020-4-20 8:00	BL	AMRO	0	6	0	0			
9860	2020-4-21 8:00	BL	AMRO	0	6	0	0			
9861	2020-4-22 8:00	BL	AMRO	0	6	0	0			
9862	2020-4-23 8:00	BL	AMRO	0	6	0	0			
9863	2020-4-24 8:00	BL	AMRO	0	6	0	0			
9864	2020-4-25 8:00	BL	AMRO	0	6	0	0			

Table S1 Daily confirmed cases and daily deaths of COVID-19 in 214 nations from WHO

ID	Date and time	Country or region	Continent	Confirmed patients	Cumulative Confirmed patients	Daily dead patients	Daily cumulative dead patients	Lag days	Daily CFR	3DMA CFR
9865	2020-4-26 8:00	BL	AMRO	0	6	0	0			
9866	2020-4-27 8:00	BL	AMRO	0	6	0	0			
9867	2020-4-28 8:00	BL	AMRO	0	6	0	0			
9868	2020-4-29 8:00	BL	AMRO	0	6	0	0			
9869	2020-4-30 8:00	BL	AMRO	0	6	0	0			
9870	2020-5-1 8:00	BL	AMRO	0	6	0	0			
9871	2020-5-2 8:00	BL	AMRO	0	6	0	0			
9872										
9873	2020-3-26 8:00	KN	AMRO	2	2	0	0			
9874	2020-3-27 8:00	KN	AMRO	0	2	0	0			
9875	2020-3-28 8:00	KN	AMRO	0	2	0	0			
9876	2020-3-29 8:00	KN	AMRO	0	2	0	0			
9877	2020-3-30 8:00	KN	AMRO	0	2	0	0			
9878	2020-3-31 8:00	KN	AMRO	0	2	0	0			
9879	2020-4-1 8:00	KN	AMRO	6	8	0	0			
9880	2020-4-2 8:00	KN	AMRO	0	8	0	0			
9881	2020-4-3 8:00	KN	AMRO	0	8	0	0			
9882	2020-4-4 8:00	KN	AMRO	0	8	0	0			
9883	2020-4-5 8:00	KN	AMRO	1	9	0	0			
9884	2020-4-6 8:00	KN	AMRO	0	9	0	0			
9885	2020-4-7 8:00	KN	AMRO	1	10	0	0			
9886	2020-4-8 8:00	KN	AMRO	0	10	0	0			
9887	2020-4-9 8:00	KN	AMRO	1	11	0	0			
9888	2020-4-10 8:00	KN	AMRO	0	11	0	0			
9889	2020-4-11 8:00	KN	AMRO	0	11	0	0			
9890	2020-4-12 8:00	KN	AMRO	1	12	0	0			
9891	2020-4-13 8:00	KN	AMRO	0	12	0	0			
9892	2020-4-14 8:00	KN	AMRO	0	12	0	0			
9893	2020-4-15 8:00	KN	AMRO	0	12	0	0			
9894	2020-4-16 8:00	KN	AMRO	2	14	0	0			
9895	2020-4-17 8:00	KN	AMRO	0	14	0	0			
9896	2020-4-18 8:00	KN	AMRO	0	14	0	0			
9897	2020-4-19 8:00	KN	AMRO	0	14	0	0			
9898	2020-4-20 8:00	KN	AMRO	0	14	0	0			
9899	2020-4-21 8:00	KN	AMRO	1	15	0	0			
9900	2020-4-22 8:00	KN	AMRO	0	15	0	0			

Table S1 Daily confirmed cases and daily deaths of COVID-19 in 214 nations from WHO

ID	Date and time	Country or region	Continent	Confirmed patients	Cumulative Confirmed patients	Daily dead patients	Daily cumulative dead patients	Lag days	Daily CFR	3DMA CFR
9901	2020-4-23 8:00	KN	AMRO	0	15	0	0			
9902	2020-4-24 8:00	KN	AMRO	0	15	0	0			
9903	2020-4-25 8:00	KN	AMRO	0	15	0	0			
9904	2020-4-26 8:00	KN	AMRO	0	15	0	0			
9905	2020-4-27 8:00	KN	AMRO	0	15	0	0			
9906	2020-4-28 8:00	KN	AMRO	0	15	0	0			
9907	2020-4-29 8:00	KN	AMRO	0	15	0	0			
9908	2020-4-30 8:00	KN	AMRO	0	15	0	0			
9909	2020-5-1 8:00	KN	AMRO	0	15	0	0			
9910	2020-5-2 8:00	KN	AMRO	0	15	0	0			
9911										
9912	2020-3-15 8:00	LC	AMRO	2	2	0	0			
9913	2020-3-16 8:00	LC	AMRO	0	2	0	0			
9914	2020-3-17 8:00	LC	AMRO	0	2	0	0			
9915	2020-3-18 8:00	LC	AMRO	0	2	0	0			
9916	2020-3-19 8:00	LC	AMRO	0	2	0	0			
9917	2020-3-20 8:00	LC	AMRO	0	2	0	0			
9918	2020-3-21 8:00	LC	AMRO	0	2	0	0			
9919	2020-3-22 8:00	LC	AMRO	0	2	0	0			
9920	2020-3-23 8:00	LC	AMRO	0	2	0	0			
9921	2020-3-24 8:00	LC	AMRO	1	3	0	0			
9922	2020-3-25 8:00	LC	AMRO	0	3	0	0			
9923	2020-3-26 8:00	LC	AMRO	0	3	0	0			
9924	2020-3-27 8:00	LC	AMRO	0	3	0	0			
9925	2020-3-28 8:00	LC	AMRO	0	3	0	0			
9926	2020-3-29 8:00	LC	AMRO	0	3	0	0			
9927	2020-3-30 8:00	LC	AMRO	1	4	0	0			
9928	2020-3-31 8:00	LC	AMRO	5	9	0	0			
9929	2020-4-1 8:00	LC	AMRO	0	9	0	0			
9930	2020-4-2 8:00	LC	AMRO	4	13	0	0			
9931	2020-4-3 8:00	LC	AMRO	0	13	0	0			
9932	2020-4-4 8:00	LC	AMRO	0	13	0	0			
9933	2020-4-5 8:00	LC	AMRO	0	13	0	0			
9934	2020-4-6 8:00	LC	AMRO	1	14	0	0			
9935	2020-4-7 8:00	LC	AMRO	0	14	0	0			
9936	2020-4-8 8:00	LC	AMRO	0	14	0	0			

Table S1 Daily confirmed cases and daily deaths of COVID-19 in 214 nations from WHO

ID	Date and time	Country or region	Continent	Confirmed patients	Cumulative Confirmed patients	Daily dead patients	Daily cumulative dead patients	Lag days	Daily CFR	3DMA CFR
9937	2020-4-9 8:00	LC	AMRO	0	14	0	0			
9938	2020-4-10 8:00	LC	AMRO	0	14	0	0			
9939	2020-4-11 8:00	LC	AMRO	0	14	0	0			
9940	2020-4-12 8:00	LC	AMRO	1	15	0	0			
9941	2020-4-13 8:00	LC	AMRO	0	15	0	0			
9942	2020-4-14 8:00	LC	AMRO	0	15	0	0			
9943	2020-4-15 8:00	LC	AMRO	0	15	0	0			
9944	2020-4-16 8:00	LC	AMRO	0	15	0	0			
9945	2020-4-17 8:00	LC	AMRO	0	15	0	0			
9946	2020-4-18 8:00	LC	AMRO	0	15	0	0			
9947	2020-4-19 8:00	LC	AMRO	0	15	0	0			
9948	2020-4-20 8:00	LC	AMRO	0	15	0	0			
9949	2020-4-21 8:00	LC	AMRO	0	15	0	0			
9950	2020-4-22 8:00	LC	AMRO	0	15	0	0			
9951	2020-4-23 8:00	LC	AMRO	0	15	0	0			
9952	2020-4-24 8:00	LC	AMRO	0	15	0	0			
9953	2020-4-25 8:00	LC	AMRO	0	15	0	0			
9954	2020-4-26 8:00	LC	AMRO	0	15	0	0			
9955	2020-4-27 8:00	LC	AMRO	0	15	0	0			
9956	2020-4-28 8:00	LC	AMRO	0	15	0	0			
9957	2020-4-29 8:00	LC	AMRO	0	15	0	0			
9958	2020-4-30 8:00	LC	AMRO	2	17	0	0			
9959	2020-5-1 8:00	LC	AMRO	0	17	0	0			
9960	2020-5-2 8:00	LC	AMRO	0	17	0	0			
9961										
9962	2020-3-2 8:00	MF	AMRO	2	2	0	0			
9963	2020-3-3 8:00	MF	AMRO	0	2	0	0			
9964	2020-3-4 8:00	MF	AMRO	0	2	0	0			
9965	2020-3-5 8:00	MF	AMRO	0	2	0	0			
9966	2020-3-6 8:00	MF	AMRO	0	2	0	0			
9967	2020-3-7 8:00	MF	AMRO	0	2	0	0			
9968	2020-3-8 8:00	MF	AMRO	0	2	0	0			
9969	2020-3-9 8:00	MF	AMRO	0	2	0	0			
9970	2020-3-10 8:00	MF	AMRO	0	2	0	0			
9971	2020-3-11 8:00	MF	AMRO	0	2	0	0			
9972	2020-3-12 8:00	MF	AMRO	0	2	0	0			

Table S1 Daily confirmed cases and daily deaths of COVID-19 in 214 nations from WHO

ID	Date and time	Country or region	Continent	Confirmed patients	Cumulative Confirmed patients	Daily dead patients	Daily cumulative dead patients	Lag days	Daily CFR	3DMA CFR
9973	2020-3-13 8:00	MF	AMRO	0	2	0	0			
9974	2020-3-14 8:00	MF	AMRO	0	2	0	0			
9975	2020-3-15 8:00	MF	AMRO	0	2	0	0			
9976	2020-3-16 8:00	MF	AMRO	0	2	0	0			
9977	2020-3-17 8:00	MF	AMRO	0	2	0	0			
9978	2020-3-18 8:00	MF	AMRO	2	4	0	0			
9979	2020-3-19 8:00	MF	AMRO	0	4	0	0			
9980	2020-3-20 8:00	MF	AMRO	0	4	0	0			
9981	2020-3-21 8:00	MF	AMRO	0	4	0	0			
9982	2020-3-22 8:00	MF	AMRO	0	4	0	0			
9983	2020-3-23 8:00	MF	AMRO	1	5	0	0			
9984	2020-3-24 8:00	MF	AMRO	3	8	0	0			
9985	2020-3-25 8:00	MF	AMRO	0	8	0	0			
9986	2020-3-26 8:00	MF	AMRO	3	11	0	0			
9987	2020-3-27 8:00	MF	AMRO	0	11	0	0			
9988	2020-3-28 8:00	MF	AMRO	1	12	0	0			
9989	2020-3-29 8:00	MF	AMRO	0	12	0	0			
9990	2020-3-30 8:00	MF	AMRO	0	12	0	0			
9991	2020-3-31 8:00	MF	AMRO	6	18	1	1	0	5.556	8.333
9992	2020-4-1 8:00	MF	AMRO	0	18	1	2	1	11.111	8.730
9993	2020-4-2 8:00	MF	AMRO	3	21	0	2	2	9.524	9.909
9994	2020-4-3 8:00	MF	AMRO	1	22	0	2	3	9.091	9.235
9995	2020-4-4 8:00	MF	AMRO	0	22	0	2	4	9.091	8.838
9996	2020-4-5 8:00	MF	AMRO	2	24	0	2	5	8.333	8.107
9997	2020-4-6 8:00	MF	AMRO	5	29	0	2	6	6.897	7.227
9998	2020-4-7 8:00	MF	AMRO	2	31	0	2	7	6.452	6.600
9999	2020-4-8 8:00	MF	AMRO	0	31	0	2	8	6.452	6.452
10000	2020-4-9 8:00	MF	AMRO	0	31	0	2	9	6.452	6.384
10001	2020-4-10 8:00	MF	AMRO	1	32	0	2	10	6.250	6.317
10002	2020-4-11 8:00	MF	AMRO	0	32	0	2	11	6.250	6.187
10003	2020-4-12 8:00	MF	AMRO	1	33	0	2	12	6.061	6.124
10004	2020-4-13 8:00	MF	AMRO	0	33	0	2	13	6.061	6.061
10005	2020-4-14 8:00	MF	AMRO	0	33	0	2	14	6.061	5.945
10006	2020-4-15 8:00	MF	AMRO	2	35	0	2	15	5.714	5.830
10007	2020-4-16 8:00	MF	AMRO	0	35	0	2	16	5.714	5.714
10008	2020-4-17 8:00	MF	AMRO	0	35	0	2	17	5.714	5.714

Table S1 Daily confirmed cases and daily deaths of COVID-19 in 214 nations from WHO

ID	Date and time	Country or region	Continent	Confirmed patients	Cumulative Confirmed patients	Daily dead patients	Daily cumulative dead patients	Lag days	Daily CFR	3DMA CFR
10009	2020-4-18 8:00	MF	AMRO	0	35	0	2	18	5.714	5.611
10010	2020-4-19 8:00	MF	AMRO	2	37	0	2	19	5.405	5.508
10011	2020-4-20 8:00	MF	AMRO	0	37	0	2	20	5.405	5.405
10012	2020-4-21 8:00	MF	AMRO	0	37	0	2	21	5.405	5.405
10013	2020-4-22 8:00	MF	AMRO	0	37	0	2	22	5.405	5.358
10014	2020-4-23 8:00	MF	AMRO	1	38	0	2	23	5.263	5.311
10015	2020-4-24 8:00	MF	AMRO	0	38	0	2	24	5.263	5.263
10016	2020-4-25 8:00	MF	AMRO	0	38	0	2	25	5.263	6.140
10017	2020-4-26 8:00	MF	AMRO	0	38	1	3	26	7.895	7.018
10018	2020-4-27 8:00	MF	AMRO	0	38	0	3	27	7.895	7.895
10019	2020-4-28 8:00	MF	AMRO	0	38	0	3	28	7.895	7.895
10020	2020-4-29 8:00	MF	AMRO	0	38	0	3	29	7.895	7.895
10021	2020-4-30 8:00	MF	AMRO	0	38	0	3	30	7.895	7.895
10022	2020-5-1 8:00	MF	AMRO	0	38	0	3	31	7.895	7.895
10023	2020-5-2 8:00	MF	AMRO	0	38	0	3	32	7.895	7.895
10024										
10025	2020-4-8 8:00	PM	AMRO	1	1	0	0			
10026	2020-4-9 8:00	PM	AMRO	0	1	0	0			
10027	2020-4-10 8:00	PM	AMRO	0	1	0	0			
10028	2020-4-11 8:00	PM	AMRO	0	1	0	0			
10029	2020-4-12 8:00	PM	AMRO	0	1	0	0			
10030	2020-4-13 8:00	PM	AMRO	0	1	0	0			
10031	2020-4-14 8:00	PM	AMRO	0	1	0	0			
10032	2020-4-15 8:00	PM	AMRO	0	1	0	0			
10033	2020-4-16 8:00	PM	AMRO	0	1	0	0			
10034	2020-4-17 8:00	PM	AMRO	0	1	0	0			
10035	2020-4-18 8:00	PM	AMRO	0	1	0	0			
10036	2020-4-19 8:00	PM	AMRO	0	1	0	0			
10037	2020-4-20 8:00	PM	AMRO	0	1	0	0			
10038	2020-4-21 8:00	PM	AMRO	0	1	0	0			
10039	2020-4-22 8:00	PM	AMRO	0	1	0	0			
10040	2020-4-23 8:00	PM	AMRO	0	1	0	0			
10041	2020-4-24 8:00	PM	AMRO	0	1	0	0			
10042	2020-4-25 8:00	PM	AMRO	0	1	0	0			
10043	2020-4-26 8:00	PM	AMRO	0	1	0	0			
10044	2020-4-27 8:00	PM	AMRO	0	1	0	0			

Table S1 Daily confirmed cases and daily deaths of COVID-19 in 214 nations from WHO

ID	Date and time	Country or region	Continent	Confirmed patients	Cumulative Confirmed patients	Daily dead patients	Daily cumulative dead patients	Lag days	Daily CFR	3DMA CFR
10045	2020-4-28 8:00	PM	AMRO	0	1	0	0			
10046	2020-4-29 8:00	PM	AMRO	0	1	0	0			
10047	2020-4-30 8:00	PM	AMRO	0	1	0	0			
10048	2020-5-1 8:00	PM	AMRO	0	1	0	0			
10049	2020-5-2 8:00	PM	AMRO	0	1	0	0			
10050										
10051	2020-3-13 8:00	VC	AMRO	1	1	0	0			
10052	2020-3-14 8:00	VC	AMRO	0	1	0	0			
10053	2020-3-15 8:00	VC	AMRO	0	1	0	0			
10054	2020-3-16 8:00	VC	AMRO	0	1	0	0			
10055	2020-3-17 8:00	VC	AMRO	0	1	0	0			
10056	2020-3-18 8:00	VC	AMRO	0	1	0	0			
10057	2020-3-19 8:00	VC	AMRO	0	1	0	0			
10058	2020-3-20 8:00	VC	AMRO	0	1	0	0			
10059	2020-3-21 8:00	VC	AMRO	0	1	0	0			
10060	2020-3-22 8:00	VC	AMRO	0	1	0	0			
10061	2020-3-23 8:00	VC	AMRO	0	1	0	0			
10062	2020-3-24 8:00	VC	AMRO	0	1	0	0			
10063	2020-3-25 8:00	VC	AMRO	0	1	0	0			
10064	2020-3-26 8:00	VC	AMRO	0	1	0	0			
10065	2020-3-27 8:00	VC	AMRO	0	1	0	0			
10066	2020-3-28 8:00	VC	AMRO	0	1	0	0			
10067	2020-3-29 8:00	VC	AMRO	0	1	0	0			
10068	2020-3-30 8:00	VC	AMRO	0	1	0	0			
10069	2020-3-31 8:00	VC	AMRO	0	1	0	0			
10070	2020-4-1 8:00	VC	AMRO	0	1	0	0			
10071	2020-4-2 8:00	VC	AMRO	0	1	0	0			
10072	2020-4-3 8:00	VC	AMRO	1	2	0	0			
10073	2020-4-4 8:00	VC	AMRO	1	3	0	0			
10074	2020-4-5 8:00	VC	AMRO	0	3	0	0			
10075	2020-4-6 8:00	VC	AMRO	0	3	0	0			
10076	2020-4-7 8:00	VC	AMRO	4	7	0	0			
10077	2020-4-8 8:00	VC	AMRO	0	7	0	0			
10078	2020-4-9 8:00	VC	AMRO	1	8	0	0			
10079	2020-4-10 8:00	VC	AMRO	0	8	0	0			
10080	2020-4-11 8:00	VC	AMRO	4	12	0	0			

Table S1 Daily confirmed cases and daily deaths of COVID-19 in 214 nations from WHO

ID	Date and time	Country or region	Continent	Confirmed patients	Cumulative Confirmed patients	Daily dead patients	Daily cumulative dead patients	Lag days	Daily CFR	3DMA CFR
10081	2020-4-12 8:00	VC	AMRO	0	12	0	0			
10082	2020-4-13 8:00	VC	AMRO	0	12	0	0			
10083	2020-4-14 8:00	VC	AMRO	0	12	0	0			
10084	2020-4-15 8:00	VC	AMRO	0	12	0	0			
10085	2020-4-16 8:00	VC	AMRO	0	12	0	0			
10086	2020-4-17 8:00	VC	AMRO	0	12	0	0			
10087	2020-4-18 8:00	VC	AMRO	0	12	0	0			
10088	2020-4-19 8:00	VC	AMRO	0	12	0	0			
10089	2020-4-20 8:00	VC	AMRO	0	12	0	0			
10090	2020-4-21 8:00	VC	AMRO	0	12	0	0			
10091	2020-4-22 8:00	VC	AMRO	0	12	0	0			
10092	2020-4-23 8:00	VC	AMRO	1	13	0	0			
10093	2020-4-24 8:00	VC	AMRO	0	13	0	0			
10094	2020-4-25 8:00	VC	AMRO	1	14	0	0			
10095	2020-4-26 8:00	VC	AMRO	0	14	0	0			
10096	2020-4-27 8:00	VC	AMRO	0	14	0	0			
10097	2020-4-28 8:00	VC	AMRO	1	15	0	0			
10098	2020-4-29 8:00	VC	AMRO	0	15	0	0			
10099	2020-4-30 8:00	VC	AMRO	1	16	0	0			
10100	2020-5-1 8:00	VC	AMRO	0	16	0	0			
10101	2020-5-2 8:00	VC	AMRO	0	16	0	0			
10102										
10103	2020-2-28 8:00	SM	EURO	1	1	0	0			
10104	2020-2-29 8:00	SM	EURO	0	1	0	0			
10105	2020-3-1 8:00	SM	EURO	0	1	0	0			
10106	2020-3-2 8:00	SM	EURO	7	8	0	0			
10107	2020-3-3 8:00	SM	EURO	0	8	0	0			
10108	2020-3-4 8:00	SM	EURO	8	16	0	0			
10109	2020-3-5 8:00	SM	EURO	5	21	0	0			
10110	2020-3-6 8:00	SM	EURO	3	24	0	0			
10111	2020-3-7 8:00	SM	EURO	3	27	1	1	0	3.704	3.203
10112	2020-3-8 8:00	SM	EURO	10	37	0	1	1	2.703	3.496
10113	2020-3-9 8:00	SM	EURO	12	49	1	2	2	4.082	3.320
10114	2020-3-10 8:00	SM	EURO	14	63	0	2	3	3.175	3.868
10115	2020-3-11 8:00	SM	EURO	6	69	1	3	4	4.348	4.672
10116	2020-3-12 8:00	SM	EURO	8	77	2	5	5	6.494	5.622

Table S1 Daily confirmed cases and daily deaths of COVID-19 in 214 nations from WHO

ID	Date and time	Country or region	Continent	Confirmed patients	Cumulative Confirmed patients	Daily dead patients	Daily cumulative dead patients	Lag days	Daily CFR	3DMA CFR
10117	2020-3-13 8:00	SM	EURO	6	83	0	5	6	6.024	6.181
10118	2020-3-14 8:00	SM	EURO	0	83	0	5	7	6.024	5.828
10119	2020-3-15 8:00	SM	EURO	9	92	0	5	8	5.435	6.761
10120	2020-3-16 8:00	SM	EURO	10	102	4	9	9	8.824	8.278
10121	2020-3-17 8:00	SM	EURO	2	104	2	11	10	10.577	10.748
10122	2020-3-18 8:00	SM	EURO	5	109	3	14	11	12.844	11.511
10123	2020-3-19 8:00	SM	EURO	17	126	0	14	12	11.111	11.076
10124	2020-3-20 8:00	SM	EURO	25	151	0	14	13	9.272	11.209
10125	2020-3-21 8:00	SM	EURO	0	151	6	20	14	13.245	11.921
10126	2020-3-22 8:00	SM	EURO	0	151	0	20	15	13.245	13.245
10127	2020-3-23 8:00	SM	EURO	0	151	0	20	16	13.245	12.395
10128	2020-3-24 8:00	SM	EURO	36	187	0	20	17	10.695	11.723
10129	2020-3-25 8:00	SM	EURO	0	187	1	21	18	11.230	10.674
10130	2020-3-26 8:00	SM	EURO	21	208	0	21	19	10.096	10.320
10131	2020-3-27 8:00	SM	EURO	10	218	0	21	20	9.633	9.647
10132	2020-3-28 8:00	SM	EURO	10	228	0	21	21	9.211	9.498
10133	2020-3-29 8:00	SM	EURO	0	228	1	22	22	9.649	9.780
10134	2020-3-30 8:00	SM	EURO	1	229	2	24	23	10.480	10.333
10135	2020-3-31 8:00	SM	EURO	1	230	1	25	24	10.870	10.789
10136	2020-4-1 8:00	SM	EURO	6	236	1	26	25	11.017	11.250
10137	2020-4-2 8:00	SM	EURO	0	236	2	28	26	11.864	11.709
10138	2020-4-3 8:00	SM	EURO	9	245	2	30	27	12.245	12.269
10139	2020-4-4 8:00	SM	EURO	7	252	2	32	28	12.698	12.433
10140	2020-4-5 8:00	SM	EURO	7	259	0	32	29	12.355	12.361
10141	2020-4-6 8:00	SM	EURO	7	266	0	32	30	12.030	11.979
10142	2020-4-7 8:00	SM	EURO	11	277	0	32	31	11.552	11.923
10143	2020-4-8 8:00	SM	EURO	2	279	2	34	32	12.186	11.593
10144	2020-4-9 8:00	SM	EURO	29	308	0	34	33	11.039	11.036
10145	2020-4-10 8:00	SM	EURO	36	344	0	34	34	9.884	10.269
10146	2020-4-11 8:00	SM	EURO	0	344	0	34	35	9.884	9.866
10147	2020-4-12 8:00	SM	EURO	12	356	1	35	36	9.831	9.849
10148	2020-4-13 8:00	SM	EURO	0	356	0	35	37	9.831	9.789
10149	2020-4-14 8:00	SM	EURO	15	371	1	36	38	9.704	9.737
10150	2020-4-15 8:00	SM	EURO	1	372	0	36	39	9.677	9.514
10151	2020-4-16 8:00	SM	EURO	21	393	0	36	40	9.160	9.253
10152	2020-4-17 8:00	SM	EURO	33	426	2	38	41	8.920	9.000

Table S1 Daily confirmed cases and daily deaths of COVID-19 in 214 nations from WHO

ID	Date and time	Country or region	Continent	Confirmed patients	Cumulative Confirmed patients	Daily dead patients	Daily cumulative dead patients	Lag days	Daily CFR	3DMA CFR
10153	2020-4-18 8:00	SM	EURO	0	426	0	38	42	8.920	8.804
10154	2020-4-19 8:00	SM	EURO	29	455	1	39	43	8.571	8.650
10155	2020-4-20 8:00	SM	EURO	6	461	0	39	44	8.460	8.491
10156	2020-4-21 8:00	SM	EURO	1	462	0	39	45	8.442	8.435
10157	2020-4-22 8:00	SM	EURO	14	476	1	40	46	8.403	8.347
10158	2020-4-23 8:00	SM	EURO	12	488	0	40	47	8.197	8.195
10159	2020-4-24 8:00	SM	EURO	13	501	0	40	48	7.984	7.993
10160	2020-4-25 8:00	SM	EURO	12	513	0	40	49	7.797	7.860
10161	2020-4-26 8:00	SM	EURO	0	513	0	40	50	7.797	7.738
10162	2020-4-27 8:00	SM	EURO	25	538	1	41	51	7.621	7.680
10163	2020-4-28 8:00	SM	EURO	0	538	0	41	52	7.621	7.552
10164	2020-4-29 8:00	SM	EURO	15	553	0	41	53	7.414	7.439
10165	2020-4-30 8:00	SM	EURO	10	563	0	41	54	7.282	7.301
10166	2020-5-1 8:00	SM	EURO	6	569	0	41	55	7.206	7.186
10167	2020-5-2 8:00	SM	EURO	11	580	0	41	56	7.069	7.137
10168										
10169	2020-4-6 8:00	ST	AFRO	4	4	0	0			
10170	2020-4-7 8:00	ST	AFRO	0	4	0	0			
10171	2020-4-8 8:00	ST	AFRO	0	4	0	0			
10172	2020-4-9 8:00	ST	AFRO	0	4	0	0			
10173	2020-4-10 8:00	ST	AFRO	0	4	0	0			
10174	2020-4-11 8:00	ST	AFRO	0	4	0	0			
10175	2020-4-12 8:00	ST	AFRO	0	4	0	0			
10176	2020-4-13 8:00	ST	AFRO	0	4	0	0			
10177	2020-4-14 8:00	ST	AFRO	0	4	0	0			
10178	2020-4-15 8:00	ST	AFRO	0	4	0	0			
10179	2020-4-16 8:00	ST	AFRO	0	4	0	0			
10180	2020-4-17 8:00	ST	AFRO	0	4	0	0			
10181	2020-4-18 8:00	ST	AFRO	0	4	0	0			
10182	2020-4-19 8:00	ST	AFRO	0	4	0	0			
10183	2020-4-20 8:00	ST	AFRO	0	4	0	0			
10184	2020-4-21 8:00	ST	AFRO	0	4	0	0			
10185	2020-4-22 8:00	ST	AFRO	0	4	0	0			
10186	2020-4-23 8:00	ST	AFRO	3	7	0	0			
10187	2020-4-24 8:00	ST	AFRO	1	8	0	0			
10188	2020-4-25 8:00	ST	AFRO	0	8	0	0			

Table S1 Daily confirmed cases and daily deaths of COVID-19 in 214 nations from WHO

ID	Date and time	Country or region	Continent	Confirmed patients	Cumulative Confirmed patients	Daily dead patients	Daily cumulative dead patients	Lag days	Daily CFR	3DMA CFR
10189	2020-4-26 8:00	ST	AFRO	0	8	0	0			
10190	2020-4-27 8:00	ST	AFRO	0	8	0	0			
10191	2020-4-28 8:00	ST	AFRO	0	8	0	0			
10192	2020-4-29 8:00	ST	AFRO	3	11	0	0			
10193	2020-4-30 8:00	ST	AFRO	0	11	0	0			
10194	2020-5-1 8:00	ST	AFRO	5	16	1	1	0	6.250	6.250
10195	2020-5-2 8:00	ST	AFRO	0	16	0	1	1	6.250	6.250
10196										
10197	2020-3-2 8:00	SA	EMRO	1	1	0	0			
10198	2020-3-3 8:00	SA	EMRO	0	1	0	0			
10199	2020-3-4 8:00	SA	EMRO	1	2	0	0			
10200	2020-3-5 8:00	SA	EMRO	3	5	0	0			
10201	2020-3-6 8:00	SA	EMRO	0	5	0	0			
10202	2020-3-7 8:00	SA	EMRO	2	7	0	0			
10203	2020-3-8 8:00	SA	EMRO	4	11	0	0			
10204	2020-3-9 8:00	SA	EMRO	4	15	0	0			
10205	2020-3-10 8:00	SA	EMRO	5	20	0	0			
10206	2020-3-11 8:00	SA	EMRO	1	21	0	0			
10207	2020-3-12 8:00	SA	EMRO	0	21	0	0			
10208	2020-3-13 8:00	SA	EMRO	0	21	0	0			
10209	2020-3-14 8:00	SA	EMRO	82	103	0	0			
10210	2020-3-15 8:00	SA	EMRO	0	103	0	0			
10211	2020-3-16 8:00	SA	EMRO	30	133	0	0			
10212	2020-3-17 8:00	SA	EMRO	38	171	0	0			
10213	2020-3-18 8:00	SA	EMRO	67	238	0	0			
10214	2020-3-19 8:00	SA	EMRO	0	238	0	0			
10215	2020-3-20 8:00	SA	EMRO	36	274	0	0			
10216	2020-3-21 8:00	SA	EMRO	118	392	0	0			
10217	2020-3-22 8:00	SA	EMRO	119	511	0	0			
10218	2020-3-23 8:00	SA	EMRO	51	562	0	0			
10219	2020-3-24 8:00	SA	EMRO	205	767	1	1	0	0.130	0.176
10220	2020-3-25 8:00	SA	EMRO	133	900	1	2	1	0.222	0.216
10221	2020-3-26 8:00	SA	EMRO	112	1012	1	3	2	0.296	0.263
10222	2020-3-27 8:00	SA	EMRO	92	1104	0	3	3	0.272	0.300
10223	2020-3-28 8:00	SA	EMRO	99	1203	1	4	4	0.333	0.407
10224	2020-3-29 8:00	SA	EMRO	96	1299	4	8	5	0.616	0.500

Table S1 Daily confirmed cases and daily deaths of COVID-19 in 214 nations from WHO

ID	Date and time	Country or region	Continent	Confirmed patients	Cumulative Confirmed patients	Daily dead patients	Daily cumulative dead patients	Lag days	Daily CFR	3DMA CFR
10225	2020-3-30 8:00	SA	EMRO	154	1453	0	8	6	0.551	0.602
10226	2020-3-31 8:00	SA	EMRO	110	1563	2	10	7	0.640	0.707
10227	2020-4-1 8:00	SA	EMRO	157	1720	6	16	8	0.930	0.895
10228	2020-4-2 8:00	SA	EMRO	165	1885	5	21	9	1.114	1.090
10229	2020-4-3 8:00	SA	EMRO	154	2039	4	25	10	1.226	1.224
10230	2020-4-4 8:00	SA	EMRO	140	2179	4	29	11	1.331	1.328
10231	2020-4-5 8:00	SA	EMRO	206	2385	5	34	12	1.426	1.421
10232	2020-4-6 8:00	SA	EMRO	138	2523	4	38	13	1.506	1.466
10233	2020-4-7 8:00	SA	EMRO	272	2795	3	41	14	1.467	1.457
10234	2020-4-8 8:00	SA	EMRO	137	2932	0	41	15	1.398	1.401
10235	2020-4-9 8:00	SA	EMRO	355	3287	3	44	16	1.339	1.341
10236	2020-4-10 8:00	SA	EMRO	364	3651	3	47	17	1.287	1.305
10237	2020-4-11 8:00	SA	EMRO	382	4033	5	52	18	1.289	1.300
10238	2020-4-12 8:00	SA	EMRO	429	4462	7	59	19	1.322	1.310
10239	2020-4-13 8:00	SA	EMRO	472	4934	6	65	20	1.317	1.333
10240	2020-4-14 8:00	SA	EMRO	435	5369	8	73	21	1.360	1.342
10241	2020-4-15 8:00	SA	EMRO	493	5862	6	79	22	1.348	1.336
10242	2020-4-16 8:00	SA	EMRO	518	6380	4	83	23	1.301	1.289
10243	2020-4-17 8:00	SA	EMRO	762	7142	4	87	24	1.218	1.210
10244	2020-4-18 8:00	SA	EMRO	1132	8274	5	92	25	1.112	1.122
10245	2020-4-19 8:00	SA	EMRO	1088	9362	5	97	26	1.036	1.043
10246	2020-4-20 8:00	SA	EMRO	1122	10484	6	103	27	0.982	0.985
10247	2020-4-21 8:00	SA	EMRO	1147	11631	6	109	28	0.937	0.937
10248	2020-4-22 8:00	SA	EMRO	1141	12772	5	114	29	0.893	0.899
10249	2020-4-23 8:00	SA	EMRO	1158	13930	7	121	30	0.869	0.867
10250	2020-4-24 8:00	SA	EMRO	1172	15102	6	127	31	0.841	0.848
10251	2020-4-25 8:00	SA	EMRO	1197	16299	9	136	32	0.834	0.823
10252	2020-4-26 8:00	SA	EMRO	1223	17522	3	139	33	0.793	0.798
10253	2020-4-27 8:00	SA	EMRO	1289	18811	5	144	34	0.766	0.772
10254	2020-4-28 8:00	SA	EMRO	1266	20077	8	152	35	0.757	0.752
10255	2020-4-29 8:00	SA	EMRO	1325	21402	5	157	36	0.734	0.734
10256	2020-4-30 8:00	SA	EMRO	1351	22753	5	162	37	0.712	0.716
10257	2020-5-1 8:00	SA	EMRO	1344	24097	7	169	38	0.701	0.705
10258	2020-5-2 8:00	SA	EMRO	0	24097	0	169	39	0.701	0.701
10259										
10260	2020-3-2 8:00	SN	AFRO	1	1	0	0			

Table S1 Daily confirmed cases and daily deaths of COVID-19 in 214 nations from WHO

ID	Date and time	Country or region	Continent	Confirmed patients	Cumulative Confirmed patients	Daily dead patients	Daily cumulative dead patients	Lag days	Daily CFR	3DMA CFR
10261	2020-3-3 8:00	SN	AFRO	0	1	0	0			
10262	2020-3-4 8:00	SN	AFRO	3	4	0	0			
10263	2020-3-5 8:00	SN	AFRO	0	4	0	0			
10264	2020-3-6 8:00	SN	AFRO	0	4	0	0			
10265	2020-3-7 8:00	SN	AFRO	0	4	0	0			
10266	2020-3-8 8:00	SN	AFRO	0	4	0	0			
10267	2020-3-9 8:00	SN	AFRO	0	4	0	0			
10268	2020-3-10 8:00	SN	AFRO	0	4	0	0			
10269	2020-3-11 8:00	SN	AFRO	0	4	0	0			
10270	2020-3-12 8:00	SN	AFRO	6	10	0	0			
10271	2020-3-13 8:00	SN	AFRO	0	10	0	0			
10272	2020-3-14 8:00	SN	AFRO	11	21	0	0			
10273	2020-3-15 8:00	SN	AFRO	5	26	0	0			
10274	2020-3-16 8:00	SN	AFRO	0	26	0	0			
10275	2020-3-17 8:00	SN	AFRO	1	27	0	0			
10276	2020-3-18 8:00	SN	AFRO	9	36	0	0			
10277	2020-3-19 8:00	SN	AFRO	2	38	0	0			
10278	2020-3-20 8:00	SN	AFRO	0	38	0	0			
10279	2020-3-21 8:00	SN	AFRO	18	56	0	0			
10280	2020-3-22 8:00	SN	AFRO	0	56	0	0			
10281	2020-3-23 8:00	SN	AFRO	11	67	0	0			
10282	2020-3-24 8:00	SN	AFRO	12	79	0	0			
10283	2020-3-25 8:00	SN	AFRO	7	86	0	0			
10284	2020-3-26 8:00	SN	AFRO	13	99	0	0			
10285	2020-3-27 8:00	SN	AFRO	6	105	0	0			
10286	2020-3-28 8:00	SN	AFRO	14	119	0	0			
10287	2020-3-29 8:00	SN	AFRO	11	130	0	0			
10288	2020-3-30 8:00	SN	AFRO	12	142	0	0			
10289	2020-3-31 8:00	SN	AFRO	20	162	0	0			
10290	2020-4-1 8:00	SN	AFRO	13	175	0	0			
10291	2020-4-2 8:00	SN	AFRO	15	190	1	1	0	0.526	0.520
10292	2020-4-3 8:00	SN	AFRO	5	195	0	1	1	0.513	0.651
10293	2020-4-4 8:00	SN	AFRO	24	219	1	2	2	0.913	0.780
10294	2020-4-5 8:00	SN	AFRO	0	219	0	2	3	0.913	0.904
10295	2020-4-6 8:00	SN	AFRO	7	226	0	2	4	0.885	0.894
10296	2020-4-7 8:00	SN	AFRO	0	226	0	2	5	0.885	0.871

Table S1 Daily confirmed cases and daily deaths of COVID-19 in 214 nations from WHO

ID	Date and time	Country or region	Continent	Confirmed patients	Cumulative Confirmed patients	Daily dead patients	Daily cumulative dead patients	Lag days	Daily CFR	3DMA CFR
10297	2020-4-8 8:00	SN	AFRO	11	237	0	2	6	0.844	0.850
10298	2020-4-9 8:00	SN	AFRO	7	244	0	2	7	0.820	0.821
10299	2020-4-10 8:00	SN	AFRO	6	250	0	2	8	0.800	0.791
10300	2020-4-11 8:00	SN	AFRO	15	265	0	2	9	0.755	0.758
10301	2020-4-12 8:00	SN	AFRO	13	278	0	2	10	0.719	0.729
10302	2020-4-13 8:00	SN	AFRO	2	280	0	2	11	0.714	0.707
10303	2020-4-14 8:00	SN	AFRO	11	291	0	2	12	0.687	0.690
10304	2020-4-15 8:00	SN	AFRO	8	299	0	2	13	0.669	0.664
10305	2020-4-16 8:00	SN	AFRO	15	314	0	2	14	0.637	0.634
10306	2020-4-17 8:00	SN	AFRO	21	335	0	2	15	0.597	0.704
10307	2020-4-18 8:00	SN	AFRO	7	342	1	3	16	0.877	0.777
10308	2020-4-19 8:00	SN	AFRO	8	350	0	3	17	0.857	0.851
10309	2020-4-20 8:00	SN	AFRO	17	367	0	3	18	0.817	1.000
10310	2020-4-21 8:00	SN	AFRO	10	377	2	5	19	1.326	1.119
10311	2020-4-22 8:00	SN	AFRO	35	412	0	5	20	1.214	1.299
10312	2020-4-23 8:00	SN	AFRO	30	442	1	6	21	1.357	1.275
10313	2020-4-24 8:00	SN	AFRO	37	479	0	6	22	1.253	1.298
10314	2020-4-25 8:00	SN	AFRO	66	545	1	7	23	1.284	1.226
10315	2020-4-26 8:00	SN	AFRO	69	614	0	7	24	1.140	1.255
10316	2020-4-27 8:00	SN	AFRO	57	671	2	9	25	1.341	1.235
10317	2020-4-28 8:00	SN	AFRO	64	735	0	9	26	1.224	1.220
10318	2020-4-29 8:00	SN	AFRO	88	823	0	9	27	1.094	1.113
10319	2020-4-30 8:00	SN	AFRO	59	882	0	9	28	1.020	1.026
10320	2020-5-1 8:00	SN	AFRO	51	933	0	9	29	0.965	0.955
10321	2020-5-2 8:00	SN	AFRO	91	1024	0	9	30	0.879	0.922
10322										
10323	2020-3-6 8:00	RS	EURO	1	1	0	0			
10324	2020-3-7 8:00	RS	EURO	0	1	0	0			
10325	2020-3-8 8:00	RS	EURO	0	1	0	0			
10326	2020-3-9 8:00	RS	EURO	0	1	0	0			
10327	2020-3-10 8:00	RS	EURO	4	5	0	0			
10328	2020-3-11 8:00	RS	EURO	7	12	0	0			
10329	2020-3-12 8:00	RS	EURO	7	19	0	0			
10330	2020-3-13 8:00	RS	EURO	12	31	0	0			
10331	2020-3-14 8:00	RS	EURO	10	41	0	0			
10332	2020-3-15 8:00	RS	EURO	0	41	0	0			

Table S1 Daily confirmed cases and daily deaths of COVID-19 in 214 nations from WHO

ID	Date and time	Country or region	Continent	Confirmed patients	Cumulative Confirmed patients	Daily dead patients	Daily cumulative dead patients	Lag days	Daily CFR	3DMA CFR
10333	2020-3-16 8:00	RS	EURO	16	57	0	0			
10334	2020-3-17 8:00	RS	EURO	15	72	0	0			
10335	2020-3-18 8:00	RS	EURO	10	82	0	0			
10336	2020-3-19 8:00	RS	EURO	41	123	0	0			
10337	2020-3-20 8:00	RS	EURO	12	135	1	1	0	0.741	0.659
10338	2020-3-21 8:00	RS	EURO	38	173	0	1	1	0.578	0.632
10339	2020-3-22 8:00	RS	EURO	0	173	0	1	2	0.578	0.538
10340	2020-3-23 8:00	RS	EURO	46	219	0	1	3	0.457	0.613
10341	2020-3-24 8:00	RS	EURO	30	249	1	2	4	0.803	0.750
10342	2020-3-25 8:00	RS	EURO	54	303	1	3	5	0.990	0.945
10343	2020-3-26 8:00	RS	EURO	81	384	1	4	6	1.042	1.188
10344	2020-3-27 8:00	RS	EURO	73	457	3	7	7	1.532	1.300
10345	2020-3-28 8:00	RS	EURO	71	528	0	7	8	1.326	1.458
10346	2020-3-29 8:00	RS	EURO	131	659	3	10	9	1.517	1.533
10347	2020-3-30 8:00	RS	EURO	82	741	3	13	10	1.754	1.643
10348	2020-3-31 8:00	RS	EURO	44	785	0	13	11	1.656	1.618
10349	2020-4-1 8:00	RS	EURO	115	900	0	13	12	1.444	1.442
10350	2020-4-2 8:00	RS	EURO	160	1060	0	13	13	1.226	1.260
10351	2020-4-3 8:00	RS	EURO	111	1171	0	13	14	1.110	1.660
10352	2020-4-4 8:00	RS	EURO	305	1476	26	39	15	2.642	2.051
10353	2020-4-5 8:00	RS	EURO	148	1624	0	39	16	2.401	2.572
10354	2020-4-6 8:00	RS	EURO	284	1908	12	51	17	2.673	2.570
10355	2020-4-7 8:00	RS	EURO	292	2200	7	58	18	2.636	2.601
10356	2020-4-8 8:00	RS	EURO	247	2447	3	61	19	2.493	2.522
10357	2020-4-9 8:00	RS	EURO	219	2666	4	65	20	2.438	2.399
10358	2020-4-10 8:00	RS	EURO	201	2867	0	65	21	2.267	2.331
10359	2020-4-11 8:00	RS	EURO	238	3105	6	71	22	2.287	2.248
10360	2020-4-12 8:00	RS	EURO	275	3380	3	74	23	2.189	2.227
10361	2020-4-13 8:00	RS	EURO	250	3630	6	80	24	2.204	2.163
10362	2020-4-14 8:00	RS	EURO	424	4054	5	85	25	2.097	2.135
10363	2020-4-15 8:00	RS	EURO	411	4465	9	94	26	2.105	2.078
10364	2020-4-16 8:00	RS	EURO	408	4873	5	99	27	2.032	2.025
10365	2020-4-17 8:00	RS	EURO	445	5318	4	103	28	1.937	1.967
10366	2020-4-18 8:00	RS	EURO	372	5690	7	110	29	1.933	1.941
10367	2020-4-19 8:00	RS	EURO	304	5994	7	117	30	1.952	1.939
10368	2020-4-20 8:00	RS	EURO	324	6318	5	122	31	1.931	1.923

Table S1 Daily confirmed cases and daily deaths of COVID-19 in 214 nations from WHO

ID	Date and time	Country or region	Continent	Confirmed patients	Cumulative Confirmed patients	Daily dead patients	Daily cumulative dead patients	Lag days	Daily CFR	3DMA CFR
10369	2020-4-21 8:00	RS	EURO	312	6630	3	125	32	1.885	1.901
10370	2020-4-22 8:00	RS	EURO	260	6890	5	130	33	1.887	1.885
10371	2020-4-23 8:00	RS	EURO	224	7114	4	134	34	1.884	1.894
10372	2020-4-24 8:00	RS	EURO	162	7276	5	139	35	1.910	1.906
10373	2020-4-25 8:00	RS	EURO	207	7483	5	144	36	1.924	1.925
10374	2020-4-26 8:00	RS	EURO	296	7779	7	151	37	1.941	1.935
10375	2020-4-27 8:00	RS	EURO	263	8042	5	156	38	1.940	1.946
10376	2020-4-28 8:00	RS	EURO	233	8275	6	162	39	1.958	1.958
10377	2020-4-29 8:00	RS	EURO	222	8497	6	168	40	1.977	1.973
10378	2020-4-30 8:00	RS	EURO	227	8724	5	173	41	1.983	1.982
10379	2020-5-1 8:00	RS	EURO	285	9009	6	179	42	1.987	1.993
10380	2020-5-2 8:00	RS	EURO	196	9205	6	185	43	2.010	1.998
10381										
10382	2020-3-15 8:00	SC	AFRO	2	2	0	0			
10383	2020-3-16 8:00	SC	AFRO	2	4	0	0			
10384	2020-3-17 8:00	SC	AFRO	0	4	0	0			
10385	2020-3-18 8:00	SC	AFRO	2	6	0	0			
10386	2020-3-19 8:00	SC	AFRO	0	6	0	0			
10387	2020-3-20 8:00	SC	AFRO	0	6	0	0			
10388	2020-3-21 8:00	SC	AFRO	1	7	0	0			
10389	2020-3-22 8:00	SC	AFRO	0	7	0	0			
10390	2020-3-23 8:00	SC	AFRO	0	7	0	0			
10391	2020-3-24 8:00	SC	AFRO	0	7	0	0			
10392	2020-3-25 8:00	SC	AFRO	0	7	0	0			
10393	2020-3-26 8:00	SC	AFRO	0	7	0	0			
10394	2020-3-27 8:00	SC	AFRO	0	7	0	0			
10395	2020-3-28 8:00	SC	AFRO	0	7	0	0			
10396	2020-3-29 8:00	SC	AFRO	1	8	0	0			
10397	2020-3-30 8:00	SC	AFRO	0	8	0	0			
10398	2020-3-31 8:00	SC	AFRO	0	8	0	0			
10399	2020-4-1 8:00	SC	AFRO	0	8	0	0			
10400	2020-4-2 8:00	SC	AFRO	2	10	0	0			
10401	2020-4-3 8:00	SC	AFRO	0	10	0	0			
10402	2020-4-4 8:00	SC	AFRO	0	10	0	0			
10403	2020-4-5 8:00	SC	AFRO	0	10	0	0			
10404	2020-4-6 8:00	SC	AFRO	0	10	0	0			

Table S1 Daily confirmed cases and daily deaths of COVID-19 in 214 nations from WHO

ID	Date and time	Country or region	Continent	Confirmed patients	Cumulative Confirmed patients	Daily dead patients	Daily cumulative dead patients	Lag days	Daily CFR	3DMA CFR
10405	2020-4-7 8:00	SC	AFRO	1	11	0	0			
10406	2020-4-8 8:00	SC	AFRO	0	11	0	0			
10407	2020-4-9 8:00	SC	AFRO	0	11	0	0			
10408	2020-4-10 8:00	SC	AFRO	0	11	0	0			
10409	2020-4-11 8:00	SC	AFRO	0	11	0	0			
10410	2020-4-12 8:00	SC	AFRO	0	11	0	0			
10411	2020-4-13 8:00	SC	AFRO	0	11	0	0			
10412	2020-4-14 8:00	SC	AFRO	0	11	0	0			
10413	2020-4-15 8:00	SC	AFRO	0	11	0	0			
10414	2020-4-16 8:00	SC	AFRO	0	11	0	0			
10415	2020-4-17 8:00	SC	AFRO	0	11	0	0			
10416	2020-4-18 8:00	SC	AFRO	0	11	0	0			
10417	2020-4-19 8:00	SC	AFRO	0	11	0	0			
10418	2020-4-20 8:00	SC	AFRO	0	11	0	0			
10419	2020-4-21 8:00	SC	AFRO	0	11	0	0			
10420	2020-4-22 8:00	SC	AFRO	0	11	0	0			
10421	2020-4-23 8:00	SC	AFRO	0	11	0	0			
10422	2020-4-24 8:00	SC	AFRO	0	11	0	0			
10423	2020-4-25 8:00	SC	AFRO	0	11	0	0			
10424	2020-4-26 8:00	SC	AFRO	0	11	0	0			
10425	2020-4-27 8:00	SC	AFRO	0	11	0	0			
10426	2020-4-28 8:00	SC	AFRO	0	11	0	0			
10427	2020-4-29 8:00	SC	AFRO	0	11	0	0			
10428	2020-4-30 8:00	SC	AFRO	0	11	0	0			
10429	2020-5-1 8:00	SC	AFRO	0	11	0	0			
10430	2020-5-2 8:00	SC	AFRO	0	11	0	0			
10431										
10432	2020-4-1 8:00	SL	AFRO	1	1	0	0			
10433	2020-4-2 8:00	SL	AFRO	1	2	0	0			
10434	2020-4-3 8:00	SL	AFRO	0	2	0	0			
10435	2020-4-4 8:00	SL	AFRO	0	2	0	0			
10436	2020-4-5 8:00	SL	AFRO	2	4	0	0			
10437	2020-4-6 8:00	SL	AFRO	2	6	0	0			
10438	2020-4-7 8:00	SL	AFRO	0	6	0	0			
10439	2020-4-8 8:00	SL	AFRO	0	6	0	0			
10440	2020-4-9 8:00	SL	AFRO	1	7	0	0			

Table S1 Daily confirmed cases and daily deaths of COVID-19 in 214 nations from WHO

ID	Date and time	Country or region	Continent	Confirmed patients	Cumulative Confirmed patients	Daily dead patients	Daily cumulative dead patients	Lag days	Daily CFR	3DMA CFR
10441	2020-4-10 8:00	SL	AFRO	0	7	0	0			
10442	2020-4-11 8:00	SL	AFRO	0	7	0	0			
10443	2020-4-12 8:00	SL	AFRO	1	8	0	0			
10444	2020-4-13 8:00	SL	AFRO	2	10	0	0			
10445	2020-4-14 8:00	SL	AFRO	0	10	0	0			
10446	2020-4-15 8:00	SL	AFRO	1	11	0	0			
10447	2020-4-16 8:00	SL	AFRO	2	13	0	0			
10448	2020-4-17 8:00	SL	AFRO	2	15	0	0			
10449	2020-4-18 8:00	SL	AFRO	11	26	0	0			
10450	2020-4-19 8:00	SL	AFRO	4	30	0	0			
10451	2020-4-20 8:00	SL	AFRO	5	35	0	0			
10452	2020-4-21 8:00	SL	AFRO	8	43	0	0			
10453	2020-4-22 8:00	SL	AFRO	7	50	0	0			
10454	2020-4-23 8:00	SL	AFRO	11	61	1	1	0	1.639	1.601
10455	2020-4-24 8:00	SL	AFRO	3	64	0	1	1	1.563	2.287
10456	2020-4-25 8:00	SL	AFRO	18	82	2	3	2	3.659	2.903
10457	2020-4-26 8:00	SL	AFRO	4	86	0	3	3	3.488	3.816
10458	2020-4-27 8:00	SL	AFRO	7	93	1	4	4	4.301	3.943
10459	2020-4-28 8:00	SL	AFRO	6	99	0	4	5	4.040	4.063
10460	2020-4-29 8:00	SL	AFRO	5	104	0	4	6	3.846	4.066
10461	2020-4-30 8:00	SL	AFRO	12	116	1	5	7	4.310	4.601
10462	2020-5-1 8:00	SL	AFRO	8	124	2	7	8	5.645	5.279
10463	2020-5-2 8:00	SL	AFRO	12	136	1	8	9	5.882	5.764
10464										
10465	2020-1-23 8:00	SG	WPRO	1	1	0	0			
10466	2020-1-24 8:00	SG	WPRO	2	3	0	0			
10467	2020-1-25 8:00	SG	WPRO	0	3	0	0			
10468	2020-1-26 8:00	SG	WPRO	1	4	0	0			
10469	2020-1-27 8:00	SG	WPRO	1	5	0	0			
10470	2020-1-28 8:00	SG	WPRO	2	7	0	0			
10471	2020-1-29 8:00	SG	WPRO	3	10	0	0			
10472	2020-1-30 8:00	SG	WPRO	3	13	0	0			
10473	2020-1-31 8:00	SG	WPRO	3	16	0	0			
10474	2020-2-1 8:00	SG	WPRO	2	18	0	0			
10475	2020-2-2 8:00	SG	WPRO	0	18	0	0			
10476	2020-2-3 8:00	SG	WPRO	0	18	0	0			

Table S1 Daily confirmed cases and daily deaths of COVID-19 in 214 nations from WHO

ID	Date and time	Country or region	Continent	Confirmed patients	Cumulative Confirmed patients	Daily dead patients	Daily cumulative dead patients	Lag days	Daily CFR	3DMA CFR
10477	2020-2-4 8:00	SG	WPRO	6	24	0	0			
10478	2020-2-5 8:00	SG	WPRO	4	28	0	0			
10479	2020-2-6 8:00	SG	WPRO	2	30	0	0			
10480	2020-2-7 8:00	SG	WPRO	3	33	0	0			
10481	2020-2-8 8:00	SG	WPRO	7	40	0	0			
10482	2020-2-9 8:00	SG	WPRO	3	43	0	0			
10483	2020-2-10 8:00	SG	WPRO	2	45	0	0			
10484	2020-2-11 8:00	SG	WPRO	2	47	0	0			
10485	2020-2-12 8:00	SG	WPRO	3	50	0	0			
10486	2020-2-13 8:00	SG	WPRO	8	58	0	0			
10487	2020-2-14 8:00	SG	WPRO	9	67	0	0			
10488	2020-2-15 8:00	SG	WPRO	5	72	0	0			
10489	2020-2-16 8:00	SG	WPRO	3	75	0	0			
10490	2020-2-17 8:00	SG	WPRO	2	77	0	0			
10491	2020-2-18 8:00	SG	WPRO	4	81	0	0			
10492	2020-2-19 8:00	SG	WPRO	3	84	0	0			
10493	2020-2-20 8:00	SG	WPRO	1	85	0	0			
10494	2020-2-21 8:00	SG	WPRO	1	86	0	0			
10495	2020-2-22 8:00	SG	WPRO	3	89	0	0			
10496	2020-2-23 8:00	SG	WPRO	0	89	0	0			
10497	2020-2-24 8:00	SG	WPRO	1	90	0	0			
10498	2020-2-25 8:00	SG	WPRO	1	91	0	0			
10499	2020-2-26 8:00	SG	WPRO	2	93	0	0			
10500	2020-2-27 8:00	SG	WPRO	3	96	0	0			
10501	2020-2-28 8:00	SG	WPRO	2	98	0	0			
10502	2020-2-29 8:00	SG	WPRO	4	102	0	0			
10503	2020-3-1 8:00	SG	WPRO	4	106	0	0			
10504	2020-3-2 8:00	SG	WPRO	2	108	0	0			
10505	2020-3-3 8:00	SG	WPRO	2	110	0	0			
10506	2020-3-4 8:00	SG	WPRO	0	110	0	0			
10507	2020-3-5 8:00	SG	WPRO	7	117	0	0			
10508	2020-3-6 8:00	SG	WPRO	13	130	0	0			
10509	2020-3-7 8:00	SG	WPRO	8	138	0	0			
10510	2020-3-8 8:00	SG	WPRO	12	150	0	0			
10511	2020-3-9 8:00	SG	WPRO	10	160	0	0			
10512	2020-3-10 8:00	SG	WPRO	6	166	0	0			

Table S1 Daily confirmed cases and daily deaths of COVID-19 in 214 nations from WHO

ID	Date and time	Country or region	Continent	Confirmed patients	Cumulative Confirmed patients	Daily dead patients	Daily cumulative dead patients	Lag days	Daily CFR	3DMA CFR
10513	2020-3-11 8:00	SG	WPRO	12	178	0	0			
10514	2020-3-12 8:00	SG	WPRO	9	187	0	0			
10515	2020-3-13 8:00	SG	WPRO	13	200	0	0			
10516	2020-3-14 8:00	SG	WPRO	12	212	0	0			
10517	2020-3-15 8:00	SG	WPRO	14	226	0	0			
10518	2020-3-16 8:00	SG	WPRO	17	243	0	0			
10519	2020-3-17 8:00	SG	WPRO	23	266	0	0			
10520	2020-3-18 8:00	SG	WPRO	47	313	0	0			
10521	2020-3-19 8:00	SG	WPRO	32	345	0	0			
10522	2020-3-20 8:00	SG	WPRO	40	385	2	2	0	0.519	0.491
10523	2020-3-21 8:00	SG	WPRO	47	432	0	2	1	0.463	0.482
10524	2020-3-22 8:00	SG	WPRO	0	432	0	2	2	0.463	0.455
10525	2020-3-23 8:00	SG	WPRO	23	455	0	2	3	0.440	0.432
10526	2020-3-24 8:00	SG	WPRO	52	507	0	2	4	0.394	0.397
10527	2020-3-25 8:00	SG	WPRO	51	558	0	2	5	0.358	0.357
10528	2020-3-26 8:00	SG	WPRO	73	631	0	2	6	0.317	0.323
10529	2020-3-27 8:00	SG	WPRO	52	683	0	2	7	0.293	0.294
10530	2020-3-28 8:00	SG	WPRO	49	732	0	2	8	0.273	0.272
10531	2020-3-29 8:00	SG	WPRO	70	802	0	2	9	0.249	0.293
10532	2020-3-30 8:00	SG	WPRO	42	844	1	3	10	0.355	0.315
10533	2020-3-31 8:00	SG	WPRO	35	879	0	3	11	0.341	0.340
10534	2020-4-1 8:00	SG	WPRO	47	926	0	3	12	0.324	0.322
10535	2020-4-2 8:00	SG	WPRO	74	1000	0	3	13	0.300	0.335
10536	2020-4-3 8:00	SG	WPRO	49	1049	1	4	14	0.381	0.377
10537	2020-4-4 8:00	SG	WPRO	65	1114	1	5	15	0.449	0.417
10538	2020-4-5 8:00	SG	WPRO	75	1189	0	5	16	0.421	0.443
10539	2020-4-6 8:00	SG	WPRO	120	1309	1	6	17	0.458	0.438
10540	2020-4-7 8:00	SG	WPRO	66	1375	0	6	18	0.436	0.433
10541	2020-4-8 8:00	SG	WPRO	106	1481	0	6	19	0.405	0.404
10542	2020-4-9 8:00	SG	WPRO	142	1623	0	6	20	0.370	0.380
10543	2020-4-10 8:00	SG	WPRO	287	1910	1	7	21	0.366	0.356
10544	2020-4-11 8:00	SG	WPRO	198	2108	0	7	22	0.332	0.349
10545	2020-4-12 8:00	SG	WPRO	191	2299	1	8	23	0.348	0.332
10546	2020-4-13 8:00	SG	WPRO	233	2532	0	8	24	0.316	0.324
10547	2020-4-14 8:00	SG	WPRO	386	2918	1	9	25	0.308	0.311
10548	2020-4-15 8:00	SG	WPRO	334	3252	1	10	26	0.308	0.295

Table S1 Daily confirmed cases and daily deaths of COVID-19 in 214 nations from WHO

ID	Date and time	Country or region	Continent	Confirmed patients	Cumulative Confirmed patients	Daily dead patients	Daily cumulative dead patients	Lag days	Daily CFR	3DMA CFR
10549	2020-4-16 8:00	SG	WPRO	447	3699	0	10	27	0.270	0.268
10550	2020-4-17 8:00	SG	WPRO	728	4427	0	10	28	0.226	0.238
10551	2020-4-18 8:00	SG	WPRO	623	5050	1	11	29	0.218	0.209
10552	2020-4-19 8:00	SG	WPRO	942	5992	0	11	30	0.184	0.189
10553	2020-4-20 8:00	SG	WPRO	596	6588	0	11	31	0.167	0.163
10554	2020-4-21 8:00	SG	WPRO	1426	8014	0	11	32	0.137	0.142
10555	2020-4-22 8:00	SG	WPRO	1111	9125	0	11	33	0.121	0.125
10556	2020-4-23 8:00	SG	WPRO	1016	10141	1	12	34	0.118	0.115
10557	2020-4-24 8:00	SG	WPRO	1037	11178	0	12	35	0.107	0.108
10558	2020-4-25 8:00	SG	WPRO	897	12075	0	12	36	0.099	0.100
10559	2020-4-26 8:00	SG	WPRO	618	12693	0	12	37	0.095	0.094
10560	2020-4-27 8:00	SG	WPRO	931	13624	0	12	38	0.088	0.093
10561	2020-4-28 8:00	SG	WPRO	799	14423	2	14	39	0.097	0.093
10562	2020-4-29 8:00	SG	WPRO	528	14951	0	14	40	0.094	0.093
10563	2020-4-30 8:00	SG	WPRO	690	15641	0	14	41	0.090	0.092
10564	2020-5-1 8:00	SG	WPRO	528	16169	1	15	42	0.093	0.092
10565	2020-5-2 8:00	SG	WPRO	932	17101	1	16	43	0.094	0.093
10566										
10567	2020-3-18 8:00	SX	AMRO	1	1	0	0			
10568	2020-3-19 8:00	SX	AMRO	0	1	0	0			
10569	2020-3-20 8:00	SX	AMRO	0	1	0	0			
10570	2020-3-21 8:00	SX	AMRO	0	1	0	0			
10571	2020-3-22 8:00	SX	AMRO	0	1	0	0			
10572	2020-3-23 8:00	SX	AMRO	0	1	0	0			
10573	2020-3-24 8:00	SX	AMRO	1	2	0	0			
10574	2020-3-25 8:00	SX	AMRO	0	2	0	0			
10575	2020-3-26 8:00	SX	AMRO	0	2	0	0			
10576	2020-3-27 8:00	SX	AMRO	0	2	0	0			
10577	2020-3-28 8:00	SX	AMRO	1	3	0	0			
10578	2020-3-29 8:00	SX	AMRO	0	3	0	0			
10579	2020-3-30 8:00	SX	AMRO	0	3	0	0			
10580	2020-3-31 8:00	SX	AMRO	3	6	0	0			
10581	2020-4-1 8:00	SX	AMRO	0	6	0	0			
10582	2020-4-2 8:00	SX	AMRO	0	6	0	0			
10583	2020-4-3 8:00	SX	AMRO	12	18	1	1	0	5.556	7.126
10584	2020-4-4 8:00	SX	AMRO	5	23	1	2	1	8.696	7.649

Table S1 Daily confirmed cases and daily deaths of COVID-19 in 214 nations from WHO

ID	Date and time	Country or region	Continent	Confirmed patients	Cumulative Confirmed patients	Daily dead patients	Daily cumulative dead patients	Lag days	Daily CFR	3DMA CFR
10585	2020-4-5 8:00	SX	AMRO	0	23	0	2	2	8.696	8.696
10586	2020-4-6 8:00	SX	AMRO	0	23	0	2	3	8.696	11.203
10587	2020-4-7 8:00	SX	AMRO	14	37	4	6	4	16.216	14.971
10588	2020-4-8 8:00	SX	AMRO	3	40	2	8	5	20.000	18.739
10589	2020-4-9 8:00	SX	AMRO	0	40	0	8	6	20.000	19.535
10590	2020-4-10 8:00	SX	AMRO	3	43	0	8	7	18.605	18.868
10591	2020-4-11 8:00	SX	AMRO	7	50	1	9	8	18.000	18.202
10592	2020-4-12 8:00	SX	AMRO	0	50	0	9	9	18.000	18.000
10593	2020-4-13 8:00	SX	AMRO	0	50	0	9	10	18.000	17.769
10594	2020-4-14 8:00	SX	AMRO	2	52	0	9	11	17.308	17.538
10595	2020-4-15 8:00	SX	AMRO	0	52	0	9	12	17.308	17.199
10596	2020-4-16 8:00	SX	AMRO	1	53	0	9	13	16.981	16.693
10597	2020-4-17 8:00	SX	AMRO	4	57	0	9	14	15.789	16.187
10598	2020-4-18 8:00	SX	AMRO	0	57	0	9	15	15.789	15.789
10599	2020-4-19 8:00	SX	AMRO	0	57	0	9	16	15.789	15.214
10600	2020-4-20 8:00	SX	AMRO	7	64	0	9	17	14.063	14.926
10601	2020-4-21 8:00	SX	AMRO	3	67	1	10	18	14.925	14.565
10602	2020-4-22 8:00	SX	AMRO	1	68	0	10	19	14.706	15.041
10603	2020-4-23 8:00	SX	AMRO	3	71	1	11	20	15.493	15.546
10604	2020-4-24 8:00	SX	AMRO	2	73	1	12	21	16.438	16.123
10605	2020-4-25 8:00	SX	AMRO	0	73	0	12	22	16.438	16.438
10606	2020-4-26 8:00	SX	AMRO	0	73	0	12	23	16.438	16.815
10607	2020-4-27 8:00	SX	AMRO	1	74	1	13	24	17.568	17.191
10608	2020-4-28 8:00	SX	AMRO	0	74	0	13	25	17.568	17.489
10609	2020-4-29 8:00	SX	AMRO	1	75	0	13	26	17.333	17.411
10610	2020-4-30 8:00	SX	AMRO	0	75	0	13	27	17.333	17.257
10611	2020-5-1 8:00	SX	AMRO	1	76	0	13	28	17.105	17.181
10612	2020-5-2 8:00	SX	AMRO	0	76	0	13	29	17.105	17.105
10613										
10614	2020-3-6 8:00	SK	EURO	1	1	0	0			
10615	2020-3-7 8:00	SK	EURO	2	3	0	0			
10616	2020-3-8 8:00	SK	EURO	2	5	0	0			
10617	2020-3-9 8:00	SK	EURO	0	5	0	0			
10618	2020-3-10 8:00	SK	EURO	2	7	0	0			
10619	2020-3-11 8:00	SK	EURO	3	10	0	0			
10620	2020-3-12 8:00	SK	EURO	11	21	0	0			

Table S1 Daily confirmed cases and daily deaths of COVID-19 in 214 nations from WHO

ID	Date and time	Country or region	Continent	Confirmed patients	Cumulative Confirmed patients	Daily dead patients	Daily cumulative dead patients	Lag days	Daily CFR	3DMA CFR
10621	2020-3-13 8:00	SK	EURO	11	32	0	0			
10622	2020-3-14 8:00	SK	EURO	12	44	0	0			
10623	2020-3-15 8:00	SK	EURO	17	61	0	0			
10624	2020-3-16 8:00	SK	EURO	11	72	0	0			
10625	2020-3-17 8:00	SK	EURO	25	97	0	0			
10626	2020-3-18 8:00	SK	EURO	10	107	0	0			
10627	2020-3-19 8:00	SK	EURO	16	123	0	0			
10628	2020-3-20 8:00	SK	EURO	14	137	0	0			
10629	2020-3-21 8:00	SK	EURO	41	178	0	0			
10630	2020-3-22 8:00	SK	EURO	0	178	0	0			
10631	2020-3-23 8:00	SK	EURO	7	185	0	0			
10632	2020-3-24 8:00	SK	EURO	6	191	0	0			
10633	2020-3-25 8:00	SK	EURO	13	204	0	0			
10634	2020-3-26 8:00	SK	EURO	12	216	0	0			
10635	2020-3-27 8:00	SK	EURO	10	226	0	0			
10636	2020-3-28 8:00	SK	EURO	69	295	0	0			
10637	2020-3-29 8:00	SK	EURO	0	295	0	0			
10638	2020-3-30 8:00	SK	EURO	41	336	0	0			
10639	2020-3-31 8:00	SK	EURO	0	336	0	0			
10640	2020-4-1 8:00	SK	EURO	27	363	0	0			
10641	2020-4-2 8:00	SK	EURO	37	400	0	0			
10642	2020-4-3 8:00	SK	EURO	26	426	0	0			
10643	2020-4-4 8:00	SK	EURO	24	450	0	0			
10644	2020-4-5 8:00	SK	EURO	21	471	0	0			
10645	2020-4-6 8:00	SK	EURO	14	485	0	0			
10646	2020-4-7 8:00	SK	EURO	49	534	2	2	0	0.375	0.354
10647	2020-4-8 8:00	SK	EURO	64	598	0	2	1	0.334	0.334
10648	2020-4-9 8:00	SK	EURO	84	682	0	2	2	0.293	0.304
10649	2020-4-10 8:00	SK	EURO	19	701	0	2	3	0.285	0.286
10650	2020-4-11 8:00	SK	EURO	14	715	0	2	4	0.280	0.280
10651	2020-4-12 8:00	SK	EURO	13	728	0	2	5	0.275	0.275
10652	2020-4-13 8:00	SK	EURO	14	742	0	2	6	0.270	0.268
10653	2020-4-14 8:00	SK	EURO	27	769	0	2	7	0.260	0.256
10654	2020-4-15 8:00	SK	EURO	66	835	0	2	8	0.240	0.398
10655	2020-4-16 8:00	SK	EURO	28	863	4	6	9	0.695	0.585
10656	2020-4-17 8:00	SK	EURO	114	977	2	8	10	0.819	0.791

Table S1 Daily confirmed cases and daily deaths of COVID-19 in 214 nations from WHO

ID	Date and time	Country or region	Continent	Confirmed patients	Cumulative Confirmed patients	Daily dead patients	Daily cumulative dead patients	Lag days	Daily CFR	3DMA CFR
10657	2020-4-18 8:00	SK	EURO	72	1049	1	9	11	0.858	0.896
10658	2020-4-19 8:00	SK	EURO	40	1089	2	11	12	1.010	0.967
10659	2020-4-20 8:00	SK	EURO	72	1161	1	12	13	1.034	1.051
10660	2020-4-21 8:00	SK	EURO	12	1173	1	13	14	1.108	1.103
10661	2020-4-22 8:00	SK	EURO	26	1199	1	14	15	1.168	1.134
10662	2020-4-23 8:00	SK	EURO	45	1244	0	14	16	1.125	1.142
10663	2020-4-24 8:00	SK	EURO	81	1325	1	15	17	1.132	1.169
10664	2020-4-25 8:00	SK	EURO	35	1360	2	17	18	1.250	1.207
10665	2020-4-26 8:00	SK	EURO	13	1373	0	17	19	1.238	1.264
10666	2020-4-27 8:00	SK	EURO	6	1379	1	18	20	1.305	1.282
10667	2020-4-28 8:00	SK	EURO	2	1381	0	18	21	1.303	1.351
10668	2020-4-29 8:00	SK	EURO	3	1384	2	20	22	1.445	1.443
10669	2020-4-30 8:00	SK	EURO	7	1391	2	22	23	1.582	1.558
10670	2020-5-1 8:00	SK	EURO	5	1396	1	23	24	1.648	1.623
10671	2020-5-2 8:00	SK	EURO	7	1403	0	23	25	1.639	1.643
10672										
10673	2020-3-4 8:00	SI	EURO	1	1	0	0			
10674	2020-3-5 8:00	SI	EURO	4	5	0	0			
10675	2020-3-6 8:00	SI	EURO	4	9	0	0			
10676	2020-3-7 8:00	SI	EURO	3	12	0	0			
10677	2020-3-8 8:00	SI	EURO	4	16	0	0			
10678	2020-3-9 8:00	SI	EURO	0	16	0	0			
10679	2020-3-10 8:00	SI	EURO	7	23	0	0			
10680	2020-3-11 8:00	SI	EURO	34	57	0	0			
10681	2020-3-12 8:00	SI	EURO	0	57	0	0			
10682	2020-3-13 8:00	SI	EURO	84	141	0	0			
10683	2020-3-14 8:00	SI	EURO	0	141	0	0			
10684	2020-3-15 8:00	SI	EURO	78	219	0	0			
10685	2020-3-16 8:00	SI	EURO	34	253	0	0			
10686	2020-3-17 8:00	SI	EURO	22	275	1	1	0	0.364	0.357
10687	2020-3-18 8:00	SI	EURO	11	286	0	1	1	0.350	0.342
10688	2020-3-19 8:00	SI	EURO	33	319	0	1	2	0.313	0.319
10689	2020-3-20 8:00	SI	EURO	22	341	0	1	3	0.293	0.289
10690	2020-3-21 8:00	SI	EURO	42	383	0	1	4	0.261	0.272
10691	2020-3-22 8:00	SI	EURO	0	383	0	1	5	0.261	0.255
10692	2020-3-23 8:00	SI	EURO	31	414	0	1	6	0.242	0.243

Table S1 Daily confirmed cases and daily deaths of COVID-19 in 214 nations from WHO

ID	Date and time	Country or region	Continent	Confirmed patients	Cumulative Confirmed patients	Daily dead patients	Daily cumulative dead patients	Lag days	Daily CFR	3DMA CFR
10693	2020-3-24 8:00	SI	EURO	28	442	0	1	7	0.226	0.364
10694	2020-3-25 8:00	SI	EURO	38	480	2	3	8	0.625	0.536
10695	2020-3-26 8:00	SI	EURO	48	528	1	4	9	0.758	0.750
10696	2020-3-27 8:00	SI	EURO	49	577	1	5	10	0.867	1.016
10697	2020-3-28 8:00	SI	EURO	55	632	4	9	11	1.424	1.198
10698	2020-3-29 8:00	SI	EURO	59	691	0	9	12	1.302	1.411
10699	2020-3-30 8:00	SI	EURO	39	730	2	11	13	1.507	1.417
10700	2020-3-31 8:00	SI	EURO	33	763	0	11	14	1.442	1.515
10701	2020-4-1 8:00	SI	EURO	51	814	2	13	15	1.597	1.607
10702	2020-4-2 8:00	SI	EURO	27	841	2	15	16	1.784	1.721
10703	2020-4-3 8:00	SI	EURO	56	897	1	16	17	1.784	1.903
10704	2020-4-4 8:00	SI	EURO	37	934	4	20	18	2.141	2.059
10705	2020-4-5 8:00	SI	EURO	43	977	2	22	19	2.252	2.401
10706	2020-4-6 8:00	SI	EURO	20	997	6	28	20	2.808	2.666
10707	2020-4-7 8:00	SI	EURO	24	1021	2	30	21	2.938	3.053
10708	2020-4-8 8:00	SI	EURO	34	1055	6	36	22	3.412	3.339
10709	2020-4-9 8:00	SI	EURO	36	1091	4	40	23	3.666	3.635
10710	2020-4-10 8:00	SI	EURO	33	1124	3	43	24	3.826	3.790
10711	2020-4-11 8:00	SI	EURO	36	1160	2	45	25	3.879	3.971
10712	2020-4-12 8:00	SI	EURO	28	1188	5	50	26	4.209	4.162
10713	2020-4-13 8:00	SI	EURO	17	1205	3	53	27	4.398	4.382
10714	2020-4-14 8:00	SI	EURO	7	1212	2	55	28	4.538	4.509
10715	2020-4-15 8:00	SI	EURO	8	1220	1	56	29	4.590	4.672
10716	2020-4-16 8:00	SI	EURO	28	1248	5	61	30	4.888	4.763
10717	2020-4-17 8:00	SI	EURO	20	1268	0	61	31	4.811	4.920
10718	2020-4-18 8:00	SI	EURO	36	1304	5	66	32	5.061	5.062
10719	2020-4-19 8:00	SI	EURO	13	1317	4	70	33	5.315	5.313
10720	2020-4-20 8:00	SI	EURO	13	1330	4	74	34	5.564	5.474
10721	2020-4-21 8:00	SI	EURO	5	1335	0	74	35	5.543	5.618
10722	2020-4-22 8:00	SI	EURO	5	1340	3	77	36	5.746	5.709
10723	2020-4-23 8:00	SI	EURO	13	1353	2	79	37	5.839	5.789
10724	2020-4-24 8:00	SI	EURO	13	1366	0	79	38	5.783	5.816
10725	2020-4-25 8:00	SI	EURO	7	1373	1	80	39	5.827	5.815
10726	2020-4-26 8:00	SI	EURO	15	1388	1	81	40	5.836	5.830
10727	2020-4-27 8:00	SI	EURO	19	1407	1	82	41	5.828	5.854
10728	2020-4-28 8:00	SI	EURO	0	1407	1	83	42	5.899	5.945

Table S1 Daily confirmed cases and daily deaths of COVID-19 in 214 nations from WHO

ID	Date and time	Country or region	Continent	Confirmed patients	Cumulative Confirmed patients	Daily dead patients	Daily cumulative dead patients	Lag days	Daily CFR	3DMA CFR
10729	2020-4-29 8:00	SI	EURO	1	1408	3	86	43	6.108	6.094
10730	2020-4-30 8:00	SI	EURO	10	1418	3	89	44	6.276	6.251
10731	2020-5-1 8:00	SI	EURO	11	1429	2	91	45	6.368	6.377
10732	2020-5-2 8:00	SI	EURO	5	1434	2	93	46	6.485	6.427
10733										
10734	2020-3-16 8:00	SO	EMRO	1	1	0	0			
10735	2020-3-17 8:00	SO	EMRO	0	1	0	0			
10736	2020-3-18 8:00	SO	EMRO	0	1	0	0			
10737	2020-3-19 8:00	SO	EMRO	0	1	0	0			
10738	2020-3-20 8:00	SO	EMRO	0	1	0	0			
10739	2020-3-21 8:00	SO	EMRO	0	1	0	0			
10740	2020-3-22 8:00	SO	EMRO	0	1	0	0			
10741	2020-3-23 8:00	SO	EMRO	0	1	0	0			
10742	2020-3-24 8:00	SO	EMRO	0	1	0	0			
10743	2020-3-25 8:00	SO	EMRO	0	1	0	0			
10744	2020-3-26 8:00	SO	EMRO	1	2	0	0			
10745	2020-3-27 8:00	SO	EMRO	1	3	0	0			
10746	2020-3-28 8:00	SO	EMRO	0	3	0	0			
10747	2020-3-29 8:00	SO	EMRO	0	3	0	0			
10748	2020-3-30 8:00	SO	EMRO	0	3	0	0			
10749	2020-3-31 8:00	SO	EMRO	0	3	0	0			
10750	2020-4-1 8:00	SO	EMRO	2	5	0	0			
10751	2020-4-2 8:00	SO	EMRO	0	5	0	0			
10752	2020-4-3 8:00	SO	EMRO	0	5	0	0			
10753	2020-4-4 8:00	SO	EMRO	2	7	0	0			
10754	2020-4-5 8:00	SO	EMRO	0	7	0	0			
10755	2020-4-6 8:00	SO	EMRO	0	7	0	0			
10756	2020-4-7 8:00	SO	EMRO	0	7	0	0			
10757	2020-4-8 8:00	SO	EMRO	1	8	0	0			
10758	2020-4-9 8:00	SO	EMRO	4	12	1	1	0	8.333	8.333
10759	2020-4-10 8:00	SO	EMRO	0	12	0	1	1	8.333	7.143
10760	2020-4-11 8:00	SO	EMRO	9	21	0	1	2	4.762	5.952
10761	2020-4-12 8:00	SO	EMRO	0	21	0	1	3	4.762	5.841
10762	2020-4-13 8:00	SO	EMRO	4	25	1	2	4	8.000	5.365
10763	2020-4-14 8:00	SO	EMRO	35	60	0	2	5	3.333	4.889
10764	2020-4-15 8:00	SO	EMRO	0	60	0	2	6	3.333	4.306

Table S1 Daily confirmed cases and daily deaths of COVID-19 in 214 nations from WHO

ID	Date and time	Country or region	Continent	Confirmed patients	Cumulative Confirmed patients	Daily dead patients	Daily cumulative dead patients	Lag days	Daily CFR	3DMA CFR
10765	2020-4-16 8:00	SO	EMRO	20	80	3	5	7	6.250	5.278
10766	2020-4-17 8:00	SO	EMRO	0	80	0	5	8	6.250	5.603
10767	2020-4-18 8:00	SO	EMRO	36	116	0	5	9	4.310	5.249
10768	2020-4-19 8:00	SO	EMRO	19	135	2	7	10	5.185	4.588
10769	2020-4-20 8:00	SO	EMRO	29	164	0	7	11	4.268	4.276
10770	2020-4-21 8:00	SO	EMRO	73	237	1	8	12	3.376	4.180
10771	2020-4-22 8:00	SO	EMRO	49	286	6	14	13	4.895	4.383
10772	2020-4-23 8:00	SO	EMRO	42	328	2	16	14	4.878	4.884
10773	2020-4-24 8:00	SO	EMRO	0	328	0	16	15	4.878	4.790
10774	2020-4-25 8:00	SO	EMRO	62	390	2	18	16	4.615	4.923
10775	2020-4-26 8:00	SO	EMRO	46	436	5	23	17	5.275	5.102
10776	2020-4-27 8:00	SO	EMRO	44	480	3	26	18	5.417	5.332
10777	2020-4-28 8:00	SO	EMRO	48	528	2	28	19	5.303	5.177
10778	2020-4-29 8:00	SO	EMRO	54	582	0	28	20	4.811	4.924
10779	2020-4-30 8:00	SO	EMRO	19	601	0	28	21	4.659	4.710
10780	2020-5-1 8:00	SO	EMRO	0	601	0	28	22	4.659	4.659
10781	2020-5-2 8:00	SO	EMRO	0	601	0	28	23	4.659	4.659
10782										
10783	2020-3-5 8:00	ZA	AFRO	1	1	0	0			
10784	2020-3-6 8:00	ZA	AFRO	0	1	0	0			
10785	2020-3-7 8:00	ZA	AFRO	1	2	0	0			
10786	2020-3-8 8:00	ZA	AFRO	1	3	0	0			
10787	2020-3-9 8:00	ZA	AFRO	0	3	0	0			
10788	2020-3-10 8:00	ZA	AFRO	4	7	0	0			
10789	2020-3-11 8:00	ZA	AFRO	0	7	0	0			
10790	2020-3-12 8:00	ZA	AFRO	10	17	0	0			
10791	2020-3-13 8:00	ZA	AFRO	0	17	0	0			
10792	2020-3-14 8:00	ZA	AFRO	21	38	0	0			
10793	2020-3-15 8:00	ZA	AFRO	0	38	0	0			
10794	2020-3-16 8:00	ZA	AFRO	13	51	0	0			
10795	2020-3-17 8:00	ZA	AFRO	11	62	0	0			
10796	2020-3-18 8:00	ZA	AFRO	54	116	0	0			
10797	2020-3-19 8:00	ZA	AFRO	34	150	0	0			
10798	2020-3-20 8:00	ZA	AFRO	55	205	0	0			
10799	2020-3-21 8:00	ZA	AFRO	35	240	0	0			
10800	2020-3-22 8:00	ZA	AFRO	0	240	0	0			

Table S1 Daily confirmed cases and daily deaths of COVID-19 in 214 nations from WHO

ID	Date and time	Country or region	Continent	Confirmed patients	Cumulative Confirmed patients	Daily dead patients	Daily cumulative dead patients	Lag days	Daily CFR	3DMA CFR
10801	2020-3-23 8:00	ZA	AFRO	34	274	0	0			
10802	2020-3-24 8:00	ZA	AFRO	128	402	0	0			
10803	2020-3-25 8:00	ZA	AFRO	152	554	0	0			
10804	2020-3-26 8:00	ZA	AFRO	373	927	0	0			
10805	2020-3-27 8:00	ZA	AFRO	0	927	0	0			
10806	2020-3-28 8:00	ZA	AFRO	243	1170	1	1	0	0.085	0.085
10807	2020-3-29 8:00	ZA	AFRO	17	1187	0	1	1	0.084	0.083
10808	2020-3-30 8:00	ZA	AFRO	93	1280	0	1	2	0.078	0.130
10809	2020-3-31 8:00	ZA	AFRO	46	1326	2	3	3	0.226	0.225
10810	2020-4-1 8:00	ZA	AFRO	27	1353	2	5	4	0.370	0.319
10811	2020-4-2 8:00	ZA	AFRO	27	1380	0	5	5	0.362	0.358
10812	2020-4-3 8:00	ZA	AFRO	82	1462	0	5	6	0.342	0.390
10813	2020-4-4 8:00	ZA	AFRO	43	1505	2	7	7	0.465	0.458
10814	2020-4-5 8:00	ZA	AFRO	80	1585	2	9	8	0.568	0.566
10815	2020-4-6 8:00	ZA	AFRO	70	1655	2	11	9	0.665	0.648
10816	2020-4-7 8:00	ZA	AFRO	31	1686	1	12	10	0.712	0.707
10817	2020-4-8 8:00	ZA	AFRO	63	1749	1	13	11	0.743	0.810
10818	2020-4-9 8:00	ZA	AFRO	96	1845	5	18	12	0.976	0.883
10819	2020-4-10 8:00	ZA	AFRO	89	1934	0	18	13	0.931	1.035
10820	2020-4-11 8:00	ZA	AFRO	69	2003	6	24	14	1.198	1.121
10821	2020-4-12 8:00	ZA	AFRO	25	2028	1	25	15	1.233	1.194
10822	2020-4-13 8:00	ZA	AFRO	145	2173	0	25	16	1.150	1.191
10823	2020-4-14 8:00	ZA	AFRO	99	2272	2	27	17	1.188	1.152
10824	2020-4-15 8:00	ZA	AFRO	143	2415	0	27	18	1.118	1.221
10825	2020-4-16 8:00	ZA	AFRO	91	2506	7	34	19	1.357	1.439
10826	2020-4-17 8:00	ZA	AFRO	99	2605	14	48	20	1.843	1.665
10827	2020-4-18 8:00	ZA	AFRO	178	2783	2	50	21	1.797	1.784
10828	2020-4-19 8:00	ZA	AFRO	251	3034	2	52	22	1.714	1.740
10829	2020-4-20 8:00	ZA	AFRO	124	3158	2	54	23	1.710	1.727
10830	2020-4-21 8:00	ZA	AFRO	142	3300	4	58	24	1.758	1.714
10831	2020-4-22 8:00	ZA	AFRO	165	3465	0	58	25	1.674	1.740
10832	2020-4-23 8:00	ZA	AFRO	170	3635	7	65	26	1.788	1.786
10833	2020-4-24 8:00	ZA	AFRO	318	3953	10	75	27	1.897	1.853
10834	2020-4-25 8:00	ZA	AFRO	267	4220	4	79	28	1.872	1.914
10835	2020-4-26 8:00	ZA	AFRO	141	4361	7	86	29	1.972	1.919
10836	2020-4-27 8:00	ZA	AFRO	185	4546	1	87	30	1.914	1.921

Table S1 Daily confirmed cases and daily deaths of COVID-19 in 214 nations from WHO

ID	Date and time	Country or region	Continent	Confirmed patients	Cumulative Confirmed patients	Daily dead patients	Daily cumulative dead patients	Lag days	Daily CFR	3DMA CFR
10837	2020-4-28 8:00	ZA	AFRO	247	4793	3	90	31	1.878	1.884
10838	2020-4-29 8:00	ZA	AFRO	203	4996	3	93	32	1.861	1.888
10839	2020-4-30 8:00	ZA	AFRO	354	5350	10	103	33	1.925	1.870
10840	2020-5-1 8:00	ZA	AFRO	297	5647	0	103	34	1.824	1.899
10841	2020-5-2 8:00	ZA	AFRO	304	5951	13	116	35	1.949	1.887
10842										
10843	2020-4-6 8:00	SS	AFRO	1	1	0	0			
10844	2020-4-7 8:00	SS	AFRO	0	1	0	0			
10845	2020-4-8 8:00	SS	AFRO	0	1	0	0			
10846	2020-4-9 8:00	SS	AFRO	0	1	0	0			
10847	2020-4-10 8:00	SS	AFRO	2	3	0	0			
10848	2020-4-11 8:00	SS	AFRO	0	3	0	0			
10849	2020-4-12 8:00	SS	AFRO	1	4	0	0			
10850	2020-4-13 8:00	SS	AFRO	0	4	0	0			
10851	2020-4-14 8:00	SS	AFRO	0	4	0	0			
10852	2020-4-15 8:00	SS	AFRO	0	4	0	0			
10853	2020-4-16 8:00	SS	AFRO	0	4	0	0			
10854	2020-4-17 8:00	SS	AFRO	0	4	0	0			
10855	2020-4-18 8:00	SS	AFRO	0	4	0	0			
10856	2020-4-19 8:00	SS	AFRO	0	4	0	0			
10857	2020-4-20 8:00	SS	AFRO	0	4	0	0			
10858	2020-4-21 8:00	SS	AFRO	0	4	0	0			
10859	2020-4-22 8:00	SS	AFRO	0	4	0	0			
10860	2020-4-23 8:00	SS	AFRO	0	4	0	0			
10861	2020-4-24 8:00	SS	AFRO	0	4	0	0			
10862	2020-4-25 8:00	SS	AFRO	1	5	0	0			
10863	2020-4-26 8:00	SS	AFRO	0	5	0	0			
10864	2020-4-27 8:00	SS	AFRO	1	6	0	0			
10865	2020-4-28 8:00	SS	AFRO	0	6	0	0			
10866	2020-4-29 8:00	SS	AFRO	28	34	0	0			
10867	2020-4-30 8:00	SS	AFRO	0	34	0	0			
10868	2020-5-1 8:00	SS	AFRO	1	35	0	0			
10869	2020-5-2 8:00	SS	AFRO	10	45	0	0			
10870										
10871	2020-1-31 8:00	ES	EURO	1	1	0	0			
10872	2020-2-1 8:00	ES	EURO	0	1	0	0			

Table S1 Daily confirmed cases and daily deaths of COVID-19 in 214 nations from WHO

ID	Date and time	Country or region	Continent	Confirmed patients	Cumulative Confirmed patients	Daily dead patients	Daily cumulative dead patients	Lag days	Daily CFR	3DMA CFR
10873	2020-2-2 8:00	ES	EURO	0	1	0	0			
10874	2020-2-3 8:00	ES	EURO	0	1	0	0			
10875	2020-2-4 8:00	ES	EURO	0	1	0	0			
10876	2020-2-5 8:00	ES	EURO	0	1	0	0			
10877	2020-2-6 8:00	ES	EURO	0	1	0	0			
10878	2020-2-7 8:00	ES	EURO	0	1	0	0			
10879	2020-2-8 8:00	ES	EURO	0	1	0	0			
10880	2020-2-9 8:00	ES	EURO	1	2	0	0			
10881	2020-2-10 8:00	ES	EURO	0	2	0	0			
10882	2020-2-11 8:00	ES	EURO	0	2	0	0			
10883	2020-2-12 8:00	ES	EURO	0	2	0	0			
10884	2020-2-13 8:00	ES	EURO	0	2	0	0			
10885	2020-2-14 8:00	ES	EURO	0	2	0	0			
10886	2020-2-15 8:00	ES	EURO	0	2	0	0			
10887	2020-2-16 8:00	ES	EURO	0	2	0	0			
10888	2020-2-17 8:00	ES	EURO	0	2	0	0			
10889	2020-2-18 8:00	ES	EURO	0	2	0	0			
10890	2020-2-19 8:00	ES	EURO	0	2	0	0			
10891	2020-2-20 8:00	ES	EURO	0	2	0	0			
10892	2020-2-21 8:00	ES	EURO	0	2	0	0			
10893	2020-2-22 8:00	ES	EURO	0	2	0	0			
10894	2020-2-23 8:00	ES	EURO	0	2	0	0			
10895	2020-2-24 8:00	ES	EURO	0	2	0	0			
10896	2020-2-25 8:00	ES	EURO	0	2	0	0			
10897	2020-2-26 8:00	ES	EURO	10	12	0	0			
10898	2020-2-27 8:00	ES	EURO	13	25	0	0			
10899	2020-2-28 8:00	ES	EURO	7	32	0	0			
10900	2020-2-29 8:00	ES	EURO	13	45	0	0			
10901	2020-3-1 8:00	ES	EURO	0	45	0	0			
10902	2020-3-2 8:00	ES	EURO	69	114	0	0			
10903	2020-3-3 8:00	ES	EURO	37	151	0	0			
10904	2020-3-4 8:00	ES	EURO	47	198	1	1	0	0.505	0.836
10905	2020-3-5 8:00	ES	EURO	59	257	2	3	1	1.167	1.003
10906	2020-3-6 8:00	ES	EURO	117	374	2	5	2	1.337	1.222
10907	2020-3-7 8:00	ES	EURO	56	430	0	5	3	1.163	1.399
10908	2020-3-8 8:00	ES	EURO	159	589	5	10	4	1.698	1.865

Table S1 Daily confirmed cases and daily deaths of COVID-19 in 214 nations from WHO

ID	Date and time	Country or region	Continent	Confirmed patients	Cumulative Confirmed patients	Daily dead patients	Daily cumulative dead patients	Lag days	Daily CFR	3DMA CFR
10909	2020-3-9 8:00	ES	EURO	435	1024	18	28	5	2.734	2.210
10910	2020-3-10 8:00	ES	EURO	615	1639	8	36	6	2.196	2.376
10911	2020-3-11 8:00	ES	EURO	501	2140	11	47	7	2.196	2.409
10912	2020-3-12 8:00	ES	EURO	825	2965	37	84	8	2.833	2.622
10913	2020-3-13 8:00	ES	EURO	1266	4231	36	120	9	2.836	2.678
10914	2020-3-14 8:00	ES	EURO	1522	5753	16	136	10	2.364	2.972
10915	2020-3-15 8:00	ES	EURO	2000	7753	152	288	11	3.715	3.147
10916	2020-3-16 8:00	ES	EURO	1438	9191	21	309	12	3.362	3.823
10917	2020-3-17 8:00	ES	EURO	1987	11178	182	491	13	4.393	4.038
10918	2020-3-18 8:00	ES	EURO	2538	13716	107	598	14	4.360	4.409
10919	2020-3-19 8:00	ES	EURO	3431	17147	169	767	15	4.473	4.616
10920	2020-3-20 8:00	ES	EURO	2833	19980	235	1002	16	5.015	4.936
10921	2020-3-21 8:00	ES	EURO	4946	24926	324	1326	17	5.320	5.218
10922	2020-3-22 8:00	ES	EURO	0	24926	0	1326	18	5.320	5.553
10923	2020-3-23 8:00	ES	EURO	3646	28572	394	1720	19	6.020	5.978
10924	2020-3-24 8:00	ES	EURO	4517	33089	462	2182	20	6.594	6.470
10925	2020-3-25 8:00	ES	EURO	6584	39673	514	2696	21	6.796	6.868
10926	2020-3-26 8:00	ES	EURO	7937	47610	738	3434	22	7.213	7.095
10927	2020-3-27 8:00	ES	EURO	8578	56188	655	4089	23	7.277	7.358
10928	2020-3-28 8:00	ES	EURO	7871	64059	769	4858	24	7.584	7.579
10929	2020-3-29 8:00	ES	EURO	8189	72248	832	5690	25	7.876	7.915
10930	2020-3-30 8:00	ES	EURO	6549	78797	838	6528	26	8.285	8.259
10931	2020-3-31 8:00	ES	EURO	6398	85195	812	7340	27	8.616	8.524
10932	2020-4-1 8:00	ES	EURO	9222	94417	849	8189	28	8.673	8.717
10933	2020-4-2 8:00	ES	EURO	7719	102136	864	9053	29	8.864	8.870
10934	2020-4-3 8:00	ES	EURO	8102	110238	950	10003	30	9.074	9.076
10935	2020-4-4 8:00	ES	EURO	7472	117710	932	10935	31	9.290	9.260
10936	2020-4-5 8:00	ES	EURO	7026	124736	809	11744	32	9.415	9.401
10937	2020-4-6 8:00	ES	EURO	6023	130759	674	12418	33	9.497	9.527
10938	2020-4-7 8:00	ES	EURO	4273	135032	637	13055	34	9.668	9.662
10939	2020-4-8 8:00	ES	EURO	5478	140510	743	13798	35	9.820	9.803
10940	2020-4-9 8:00	ES	EURO	6180	146690	757	14555	36	9.922	9.913
10941	2020-4-10 8:00	ES	EURO	5756	152446	683	15238	37	9.996	10.003
10942	2020-4-11 8:00	ES	EURO	4576	157022	605	15843	38	10.090	10.063
10943	2020-4-12 8:00	ES	EURO	4830	161852	510	16353	39	10.104	10.139
10944	2020-4-13 8:00	ES	EURO	4167	166019	619	16972	40	10.223	10.215

Table S1 Daily confirmed cases and daily deaths of COVID-19 in 214 nations from WHO

ID	Date and time	Country or region	Continent	Confirmed patients	Cumulative Confirmed patients	Daily dead patients	Daily cumulative dead patients	Lag days	Daily CFR	3DMA CFR
10945	2020-4-14 8:00	ES	EURO	3477	169496	517	17489	41	10.318	10.266
10946	2020-4-15 8:00	ES	EURO	6538	176034	567	18056	42	10.257	10.298
10947	2020-4-16 8:00	ES	EURO	4040	180074	523	18579	43	10.317	10.315
10948	2020-4-17 8:00	ES	EURO	4400	184474	551	19130	44	10.370	10.339
10949	2020-4-18 8:00	ES	EURO	4104	188578	348	19478	45	10.329	10.442
10950	2020-4-19 8:00	ES	EURO	0	188578	565	20043	46	10.628	10.552
10951	2020-4-20 8:00	ES	EURO	2585	191163	410	20453	47	10.699	10.683
10952	2020-4-21 8:00	ES	EURO	3352	194515	399	20852	48	10.720	10.738
10953	2020-4-22 8:00	ES	EURO	2627	197142	430	21282	49	10.795	10.788
10954	2020-4-23 8:00	ES	EURO	3052	200194	435	21717	50	10.848	10.853
10955	2020-4-24 8:00	ES	EURO	2767	202961	440	22157	51	10.917	10.901
10956	2020-4-25 8:00	ES	EURO	2944	205905	367	22524	52	10.939	10.932
10957	2020-4-26 8:00	ES	EURO	0	205905	0	22524	53	10.939	11.016
10958	2020-4-27 8:00	ES	EURO	1729	207634	666	23190	54	11.169	11.060
10959	2020-4-28 8:00	ES	EURO	1831	209465	0	23190	55	11.071	11.181
10960	2020-4-29 8:00	ES	EURO	1308	210773	632	23822	56	11.302	11.258
10961	2020-4-30 8:00	ES	EURO	2144	212917	453	24275	57	11.401	11.401
10962	2020-5-1 8:00	ES	EURO	518	213435	268	24543	58	11.499	11.478
10963	2020-5-2 8:00	ES	EURO	1781	215216	281	24824	59	11.534	11.517
10964										
10965	2020-1-27 8:00	LK	SEARO	1	1	0	0			
10966	2020-1-28 8:00	LK	SEARO	0	1	0	0			
10967	2020-1-29 8:00	LK	SEARO	0	1	0	0			
10968	2020-1-30 8:00	LK	SEARO	0	1	0	0			
10969	2020-1-31 8:00	LK	SEARO	0	1	0	0			
10970	2020-2-1 8:00	LK	SEARO	0	1	0	0			
10971	2020-2-2 8:00	LK	SEARO	0	1	0	0			
10972	2020-2-3 8:00	LK	SEARO	0	1	0	0			
10973	2020-2-4 8:00	LK	SEARO	0	1	0	0			
10974	2020-2-5 8:00	LK	SEARO	0	1	0	0			
10975	2020-2-6 8:00	LK	SEARO	0	1	0	0			
10976	2020-2-7 8:00	LK	SEARO	0	1	0	0			
10977	2020-2-8 8:00	LK	SEARO	0	1	0	0			
10978	2020-2-9 8:00	LK	SEARO	0	1	0	0			
10979	2020-2-10 8:00	LK	SEARO	0	1	0	0			
10980	2020-2-11 8:00	LK	SEARO	0	1	0	0			

Table S1 Daily confirmed cases and daily deaths of COVID-19 in 214 nations from WHO

ID	Date and time	Country or region	Continent	Confirmed patients	Cumulative Confirmed patients	Daily dead patients	Daily cumulative dead patients	Lag days	Daily CFR	3DMA CFR
10981	2020-2-12 8:00	LK	SEARO	0	1	0	0			
10982	2020-2-13 8:00	LK	SEARO	0	1	0	0			
10983	2020-2-14 8:00	LK	SEARO	0	1	0	0			
10984	2020-2-15 8:00	LK	SEARO	0	1	0	0			
10985	2020-2-16 8:00	LK	SEARO	0	1	0	0			
10986	2020-2-17 8:00	LK	SEARO	0	1	0	0			
10987	2020-2-18 8:00	LK	SEARO	0	1	0	0			
10988	2020-2-19 8:00	LK	SEARO	0	1	0	0			
10989	2020-2-20 8:00	LK	SEARO	0	1	0	0			
10990	2020-2-21 8:00	LK	SEARO	0	1	0	0			
10991	2020-2-22 8:00	LK	SEARO	0	1	0	0			
10992	2020-2-23 8:00	LK	SEARO	0	1	0	0			
10993	2020-2-24 8:00	LK	SEARO	0	1	0	0			
10994	2020-2-25 8:00	LK	SEARO	0	1	0	0			
10995	2020-2-26 8:00	LK	SEARO	0	1	0	0			
10996	2020-2-27 8:00	LK	SEARO	0	1	0	0			
10997	2020-2-28 8:00	LK	SEARO	0	1	0	0			
10998	2020-2-29 8:00	LK	SEARO	0	1	0	0			
10999	2020-3-1 8:00	LK	SEARO	0	1	0	0			
11000	2020-3-2 8:00	LK	SEARO	0	1	0	0			
11001	2020-3-3 8:00	LK	SEARO	0	1	0	0			
11002	2020-3-4 8:00	LK	SEARO	0	1	0	0			
11003	2020-3-5 8:00	LK	SEARO	0	1	0	0			
11004	2020-3-6 8:00	LK	SEARO	0	1	0	0			
11005	2020-3-7 8:00	LK	SEARO	0	1	0	0			
11006	2020-3-8 8:00	LK	SEARO	0	1	0	0			
11007	2020-3-9 8:00	LK	SEARO	0	1	0	0			
11008	2020-3-10 8:00	LK	SEARO	0	1	0	0			
11009	2020-3-11 8:00	LK	SEARO	1	2	0	0			
11010	2020-3-12 8:00	LK	SEARO	1	3	0	0			
11011	2020-3-13 8:00	LK	SEARO	0	3	0	0			
11012	2020-3-14 8:00	LK	SEARO	3	6	0	0			
11013	2020-3-15 8:00	LK	SEARO	5	11	0	0			
11014	2020-3-16 8:00	LK	SEARO	8	19	0	0			
11015	2020-3-17 8:00	LK	SEARO	10	29	0	0			
11016	2020-3-18 8:00	LK	SEARO	13	42	0	0			

Table S1 Daily confirmed cases and daily deaths of COVID-19 in 214 nations from WHO

ID	Date and time	Country or region	Continent	Confirmed patients	Cumulative Confirmed patients	Daily dead patients	Daily cumulative dead patients	Lag days	Daily CFR	3DMA CFR
11017	2020-3-19 8:00	LK	SEARO	17	59	0	0			
11018	2020-3-20 8:00	LK	SEARO	0	59	0	0			
11019	2020-3-21 8:00	LK	SEARO	13	72	0	0			
11020	2020-3-22 8:00	LK	SEARO	0	72	0	0			
11021	2020-3-23 8:00	LK	SEARO	22	94	0	0			
11022	2020-3-24 8:00	LK	SEARO	3	97	0	0			
11023	2020-3-25 8:00	LK	SEARO	5	102	0	0			
11024	2020-3-26 8:00	LK	SEARO	0	102	0	0			
11025	2020-3-27 8:00	LK	SEARO	4	106	0	0			
11026	2020-3-28 8:00	LK	SEARO	0	106	0	0			
11027	2020-3-29 8:00	LK	SEARO	9	115	1	1	0	0.870	0.851
11028	2020-3-30 8:00	LK	SEARO	5	120	0	1	1	0.833	1.114
11029	2020-3-31 8:00	LK	SEARO	2	122	1	2	2	1.639	1.290
11030	2020-4-1 8:00	LK	SEARO	21	143	0	2	3	1.399	1.688
11031	2020-4-2 8:00	LK	SEARO	5	148	1	3	4	2.027	2.025
11032	2020-4-3 8:00	LK	SEARO	3	151	1	4	5	2.649	2.607
11033	2020-4-4 8:00	LK	SEARO	8	159	1	5	6	3.145	2.935
11034	2020-4-5 8:00	LK	SEARO	7	166	0	5	7	3.012	2.999
11035	2020-4-6 8:00	LK	SEARO	10	176	0	5	8	2.841	3.062
11036	2020-4-7 8:00	LK	SEARO	4	180	1	6	9	3.333	3.133
11037	2020-4-8 8:00	LK	SEARO	6	186	0	6	10	3.226	3.421
11038	2020-4-9 8:00	LK	SEARO	3	189	1	7	11	3.704	3.538
11039	2020-4-10 8:00	LK	SEARO	1	190	0	7	12	3.684	3.647
11040	2020-4-11 8:00	LK	SEARO	7	197	0	7	13	3.553	3.585
11041	2020-4-12 8:00	LK	SEARO	2	199	0	7	14	3.518	3.468
11042	2020-4-13 8:00	LK	SEARO	11	210	0	7	15	3.333	3.354
11043	2020-4-14 8:00	LK	SEARO	8	218	0	7	16	3.211	3.183
11044	2020-4-15 8:00	LK	SEARO	15	233	0	7	17	3.004	3.052
11045	2020-4-16 8:00	LK	SEARO	5	238	0	7	18	2.941	2.962
11046	2020-4-17 8:00	LK	SEARO	0	238	0	7	19	2.941	2.917
11047	2020-4-18 8:00	LK	SEARO	6	244	0	7	20	2.869	2.855
11048	2020-4-19 8:00	LK	SEARO	10	254	0	7	21	2.756	2.736
11049	2020-4-20 8:00	LK	SEARO	17	271	0	7	22	2.583	2.547
11050	2020-4-21 8:00	LK	SEARO	33	304	0	7	23	2.303	2.381
11051	2020-4-22 8:00	LK	SEARO	6	310	0	7	24	2.258	2.227
11052	2020-4-23 8:00	LK	SEARO	20	330	0	7	25	2.121	2.094

Table S1 Daily confirmed cases and daily deaths of COVID-19 in 214 nations from WHO

ID	Date and time	Country or region	Continent	Confirmed patients	Cumulative Confirmed patients	Daily dead patients	Daily cumulative dead patients	Lag days	Daily CFR	3DMA CFR
11053	2020-4-24 8:00	LK	SEARO	38	368	0	7	26	1.902	1.897
11054	2020-4-25 8:00	LK	SEARO	52	420	0	7	27	1.667	1.697
11055	2020-4-26 8:00	LK	SEARO	40	460	0	7	28	1.522	1.509
11056	2020-4-27 8:00	LK	SEARO	63	523	0	7	29	1.338	1.350
11057	2020-4-28 8:00	LK	SEARO	65	588	0	7	30	1.190	1.220
11058	2020-4-29 8:00	LK	SEARO	31	619	0	7	31	1.131	1.133
11059	2020-4-30 8:00	LK	SEARO	30	649	0	7	32	1.079	1.087
11060	2020-5-1 8:00	LK	SEARO	16	665	0	7	33	1.053	1.049
11061	2020-5-2 8:00	LK	SEARO	25	690	0	7	34	1.014	1.034
11062										
11063	2020-3-14 8:00	SD	EMRO	1	1	0	0			
11064	2020-3-15 8:00	SD	EMRO	0	1	1	1	0	100.000	100.000
11065	2020-3-16 8:00	SD	EMRO	0	1	0	1	1	100.000	100.000
11066	2020-3-17 8:00	SD	EMRO	0	1	0	1	2	100.000	100.000
11067	2020-3-18 8:00	SD	EMRO	0	1	0	1	3	100.000	100.000
11068	2020-3-19 8:00	SD	EMRO	0	1	0	1	4	100.000	100.000
11069	2020-3-20 8:00	SD	EMRO	0	1	0	1	5	100.000	83.333
11070	2020-3-21 8:00	SD	EMRO	1	2	0	1	6	50.000	66.667
11071	2020-3-22 8:00	SD	EMRO	0	2	0	1	7	50.000	50.000
11072	2020-3-23 8:00	SD	EMRO	0	2	0	1	8	50.000	44.444
11073	2020-3-24 8:00	SD	EMRO	1	3	0	1	9	33.333	38.889
11074	2020-3-25 8:00	SD	EMRO	0	3	0	1	10	33.333	33.333
11075	2020-3-26 8:00	SD	EMRO	0	3	0	1	11	33.333	33.333
11076	2020-3-27 8:00	SD	EMRO	0	3	0	1	12	33.333	28.889
11077	2020-3-28 8:00	SD	EMRO	2	5	0	1	13	20.000	24.444
11078	2020-3-29 8:00	SD	EMRO	0	5	0	1	14	20.000	24.444
11079	2020-3-30 8:00	SD	EMRO	1	6	1	2	15	33.333	27.302
11080	2020-3-31 8:00	SD	EMRO	1	7	0	2	16	28.571	30.159
11081	2020-4-1 8:00	SD	EMRO	0	7	0	2	17	28.571	28.571
11082	2020-4-2 8:00	SD	EMRO	0	7	0	2	18	28.571	25.714
11083	2020-4-3 8:00	SD	EMRO	3	10	0	2	19	20.000	22.857
11084	2020-4-4 8:00	SD	EMRO	0	10	0	2	20	20.000	18.889
11085	2020-4-5 8:00	SD	EMRO	2	12	0	2	21	16.667	17.778
11086	2020-4-6 8:00	SD	EMRO	0	12	0	2	22	16.667	15.873
11087	2020-4-7 8:00	SD	EMRO	2	14	0	2	23	14.286	15.079
11088	2020-4-8 8:00	SD	EMRO	0	14	0	2	24	14.286	14.286

Table S1 Daily confirmed cases and daily deaths of COVID-19 in 214 nations from WHO

ID	Date and time	Country or region	Continent	Confirmed patients	Cumulative Confirmed patients	Daily dead patients	Daily cumulative dead patients	Lag days	Daily CFR	3DMA CFR
11089	2020-4-9 8:00	SD	EMRO	0	14	0	2	25	14.286	13.968
11090	2020-4-10 8:00	SD	EMRO	1	15	0	2	26	13.333	12.715
11091	2020-4-11 8:00	SD	EMRO	4	19	0	2	27	10.526	11.462
11092	2020-4-12 8:00	SD	EMRO	0	19	0	2	28	10.526	10.526
11093	2020-4-13 8:00	SD	EMRO	0	19	0	2	29	10.526	11.615
11094	2020-4-14 8:00	SD	EMRO	10	29	2	4	30	13.793	13.315
11095	2020-4-15 8:00	SD	EMRO	3	32	1	5	31	15.625	15.014
11096	2020-4-16 8:00	SD	EMRO	0	32	0	5	32	15.625	15.625
11097	2020-4-17 8:00	SD	EMRO	0	32	0	5	33	15.625	15.467
11098	2020-4-18 8:00	SD	EMRO	34	66	5	10	34	15.152	15.309
11099	2020-4-19 8:00	SD	EMRO	0	66	0	10	35	15.152	14.449
11100	2020-4-20 8:00	SD	EMRO	26	92	2	12	36	13.043	13.137
11101	2020-4-21 8:00	SD	EMRO	15	107	0	12	37	11.215	11.181
11102	2020-4-22 8:00	SD	EMRO	33	140	1	13	38	9.286	9.508
11103	2020-4-23 8:00	SD	EMRO	22	162	0	13	39	8.025	8.835
11104	2020-4-24 8:00	SD	EMRO	12	174	3	16	40	9.195	8.400
11105	2020-4-25 8:00	SD	EMRO	39	213	1	17	41	7.981	8.679
11106	2020-4-26 8:00	SD	EMRO	24	237	4	21	42	8.861	8.281
11107	2020-4-27 8:00	SD	EMRO	38	275	1	22	43	8.000	8.241
11108	2020-4-28 8:00	SD	EMRO	43	318	3	25	44	7.862	7.776
11109	2020-4-29 8:00	SD	EMRO	57	375	3	28	45	7.467	7.598
11110	2020-4-30 8:00	SD	EMRO	0	375	0	28	46	7.467	7.316
11111	2020-5-1 8:00	SD	EMRO	67	442	3	31	47	7.014	7.078
11112	2020-5-2 8:00	SD	EMRO	91	533	5	36	48	6.754	6.884
11113										
11114	2020-3-15 8:00	SR	AMRO	1	1	0	0			
11115	2020-3-16 8:00	SR	AMRO	0	1	0	0			
11116	2020-3-17 8:00	SR	AMRO	0	1	0	0			
11117	2020-3-18 8:00	SR	AMRO	0	1	0	0			
11118	2020-3-19 8:00	SR	AMRO	0	1	0	0			
11119	2020-3-20 8:00	SR	AMRO	1	2	0	0			
11120	2020-3-21 8:00	SR	AMRO	0	2	0	0			
11121	2020-3-22 8:00	SR	AMRO	0	2	0	0			
11122	2020-3-23 8:00	SR	AMRO	0	2	0	0			
11123	2020-3-24 8:00	SR	AMRO	4	6	0	0			
11124	2020-3-25 8:00	SR	AMRO	0	6	0	0			

Table S1 Daily confirmed cases and daily deaths of COVID-19 in 214 nations from WHO

ID	Date and time	Country or region	Continent	Confirmed patients	Cumulative Confirmed patients	Daily dead patients	Daily cumulative dead patients	Lag days	Daily CFR	3DMA CFR
11125	2020-3-26 8:00	SR	AMRO	1	7	0	0			
11126	2020-3-27 8:00	SR	AMRO	0	7	0	0			
11127	2020-3-28 8:00	SR	AMRO	1	8	0	0			
11128	2020-3-29 8:00	SR	AMRO	0	8	0	0			
11129	2020-3-30 8:00	SR	AMRO	0	8	0	0			
11130	2020-3-31 8:00	SR	AMRO	0	8	0	0			
11131	2020-4-1 8:00	SR	AMRO	0	8	0	0			
11132	2020-4-2 8:00	SR	AMRO	0	8	0	0			
11133	2020-4-3 8:00	SR	AMRO	0	8	0	0			
11134	2020-4-4 8:00	SR	AMRO	2	10	0	0			
11135	2020-4-5 8:00	SR	AMRO	0	10	0	0			
11136	2020-4-6 8:00	SR	AMRO	0	10	0	0			
11137	2020-4-7 8:00	SR	AMRO	0	10	1	1	0	10.000	10.000
11138	2020-4-8 8:00	SR	AMRO	0	10	0	1	1	10.000	10.000
11139	2020-4-9 8:00	SR	AMRO	0	10	0	1	2	10.000	10.000
11140	2020-4-10 8:00	SR	AMRO	0	10	0	1	3	10.000	10.000
11141	2020-4-11 8:00	SR	AMRO	0	10	0	1	4	10.000	10.000
11142	2020-4-12 8:00	SR	AMRO	0	10	0	1	5	10.000	10.000
11143	2020-4-13 8:00	SR	AMRO	0	10	0	1	6	10.000	10.000
11144	2020-4-14 8:00	SR	AMRO	0	10	0	1	7	10.000	10.000
11145	2020-4-15 8:00	SR	AMRO	0	10	0	1	8	10.000	10.000
11146	2020-4-16 8:00	SR	AMRO	0	10	0	1	9	10.000	10.000
11147	2020-4-17 8:00	SR	AMRO	0	10	0	1	10	10.000	10.000
11148	2020-4-18 8:00	SR	AMRO	0	10	0	1	11	10.000	10.000
11149	2020-4-19 8:00	SR	AMRO	0	10	0	1	12	10.000	10.000
11150	2020-4-20 8:00	SR	AMRO	0	10	0	1	13	10.000	10.000
11151	2020-4-21 8:00	SR	AMRO	0	10	0	1	14	10.000	10.000
11152	2020-4-22 8:00	SR	AMRO	0	10	0	1	15	10.000	10.000
11153	2020-4-23 8:00	SR	AMRO	0	10	0	1	16	10.000	10.000
11154	2020-4-24 8:00	SR	AMRO	0	10	0	1	17	10.000	10.000
11155	2020-4-25 8:00	SR	AMRO	0	10	0	1	18	10.000	10.000
11156	2020-4-26 8:00	SR	AMRO	0	10	0	1	19	10.000	10.000
11157	2020-4-27 8:00	SR	AMRO	0	10	0	1	20	10.000	10.000
11158	2020-4-28 8:00	SR	AMRO	0	10	0	1	21	10.000	10.000
11159	2020-4-29 8:00	SR	AMRO	0	10	0	1	22	10.000	10.000
11160	2020-4-30 8:00	SR	AMRO	0	10	0	1	23	10.000	10.000

Table S1 Daily confirmed cases and daily deaths of COVID-19 in 214 nations from WHO

ID	Date and time	Country or region	Continent	Confirmed patients	Cumulative Confirmed patients	Daily dead patients	Daily cumulative dead patients	Lag days	Daily CFR	3DMA CFR
11161	2020-5-1 8:00	SR	AMRO	0	10	0	1	24	10.000	10.000
11162	2020-5-2 8:00	SR	AMRO	0	10	0	1	25	10.000	10.000
11163										
11164	2020-1-31 8:00	SE	EURO	1	1	0	0			
11165	2020-2-1 8:00	SE	EURO	0	1	0	0			
11166	2020-2-2 8:00	SE	EURO	0	1	0	0			
11167	2020-2-3 8:00	SE	EURO	0	1	0	0			
11168	2020-2-4 8:00	SE	EURO	0	1	0	0			
11169	2020-2-5 8:00	SE	EURO	0	1	0	0			
11170	2020-2-6 8:00	SE	EURO	0	1	0	0			
11171	2020-2-7 8:00	SE	EURO	0	1	0	0			
11172	2020-2-8 8:00	SE	EURO	0	1	0	0			
11173	2020-2-9 8:00	SE	EURO	0	1	0	0			
11174	2020-2-10 8:00	SE	EURO	0	1	0	0			
11175	2020-2-11 8:00	SE	EURO	0	1	0	0			
11176	2020-2-12 8:00	SE	EURO	0	1	0	0			
11177	2020-2-13 8:00	SE	EURO	0	1	0	0			
11178	2020-2-14 8:00	SE	EURO	0	1	0	0			
11179	2020-2-15 8:00	SE	EURO	0	1	0	0			
11180	2020-2-16 8:00	SE	EURO	0	1	0	0			
11181	2020-2-17 8:00	SE	EURO	0	1	0	0			
11182	2020-2-18 8:00	SE	EURO	0	1	0	0			
11183	2020-2-19 8:00	SE	EURO	0	1	0	0			
11184	2020-2-20 8:00	SE	EURO	0	1	0	0			
11185	2020-2-21 8:00	SE	EURO	0	1	0	0			
11186	2020-2-22 8:00	SE	EURO	0	1	0	0			
11187	2020-2-23 8:00	SE	EURO	0	1	0	0			
11188	2020-2-24 8:00	SE	EURO	0	1	0	0			
11189	2020-2-25 8:00	SE	EURO	0	1	0	0			
11190	2020-2-26 8:00	SE	EURO	1	2	0	0			
11191	2020-2-27 8:00	SE	EURO	5	7	0	0			
11192	2020-2-28 8:00	SE	EURO	4	11	0	0			
11193	2020-2-29 8:00	SE	EURO	2	13	0	0			
11194	2020-3-1 8:00	SE	EURO	1	14	0	0			
11195	2020-3-2 8:00	SE	EURO	1	15	0	0			
11196	2020-3-3 8:00	SE	EURO	9	24	0	0			

Table S1 Daily confirmed cases and daily deaths of COVID-19 in 214 nations from WHO

ID	Date and time	Country or region	Continent	Confirmed patients	Cumulative Confirmed patients	Daily dead patients	Daily cumulative dead patients	Lag days	Daily CFR	3DMA CFR
11197	2020-3-4 8:00	SE	EURO	11	35	0	0			
11198	2020-3-5 8:00	SE	EURO	26	61	0	0			
11199	2020-3-6 8:00	SE	EURO	76	137	0	0			
11200	2020-3-7 8:00	SE	EURO	24	161	0	0			
11201	2020-3-8 8:00	SE	EURO	42	203	0	0			
11202	2020-3-9 8:00	SE	EURO	45	248	0	0			
11203	2020-3-10 8:00	SE	EURO	78	326	0	0			
11204	2020-3-11 8:00	SE	EURO	135	461	0	0			
11205	2020-3-12 8:00	SE	EURO	159	620	0	0			
11206	2020-3-13 8:00	SE	EURO	155	775	0	0			
11207	2020-3-14 8:00	SE	EURO	149	924	0	0			
11208	2020-3-15 8:00	SE	EURO	68	992	3	3	0	0.302	0.293
11209	2020-3-16 8:00	SE	EURO	67	1059	0	3	1	0.283	0.281
11210	2020-3-17 8:00	SE	EURO	108	1167	0	3	2	0.257	0.258
11211	2020-3-18 8:00	SE	EURO	112	1279	0	3	3	0.235	0.234
11212	2020-3-19 8:00	SE	EURO	144	1423	0	3	4	0.211	0.477
11213	2020-3-20 8:00	SE	EURO	200	1623	13	16	5	0.986	0.781
11214	2020-3-21 8:00	SE	EURO	123	1746	4	20	6	1.145	1.092
11215	2020-3-22 8:00	SE	EURO	0	1746	0	20	7	1.145	1.131
11216	2020-3-23 8:00	SE	EURO	160	1906	1	21	8	1.102	1.162
11217	2020-3-24 8:00	SE	EURO	110	2016	4	25	9	1.240	1.309
11218	2020-3-25 8:00	SE	EURO	256	2272	11	36	10	1.585	1.499
11219	2020-3-26 8:00	SE	EURO	238	2510	6	42	11	1.673	1.870
11220	2020-3-27 8:00	SE	EURO	296	2806	24	66	12	2.352	2.349
11221	2020-3-28 8:00	SE	EURO	240	3046	26	92	13	3.020	2.777
11222	2020-3-29 8:00	SE	EURO	401	3447	10	102	14	2.959	2.984
11223	2020-3-30 8:00	SE	EURO	253	3700	8	110	15	2.973	3.186
11224	2020-3-31 8:00	SE	EURO	328	4028	36	146	16	3.625	3.552
11225	2020-4-1 8:00	SE	EURO	407	4435	34	180	17	4.059	4.171
11226	2020-4-2 8:00	SE	EURO	512	4947	59	239	18	4.831	4.683
11227	2020-4-3 8:00	SE	EURO	519	5466	43	282	19	5.159	5.156
11228	2020-4-4 8:00	SE	EURO	612	6078	51	333	20	5.479	5.476
11229	2020-4-5 8:00	SE	EURO	365	6443	40	373	21	5.789	5.713
11230	2020-4-6 8:00	SE	EURO	387	6830	28	401	22	5.871	6.093
11231	2020-4-7 8:00	SE	EURO	376	7206	76	477	23	6.619	6.724
11232	2020-4-8 8:00	SE	EURO	487	7693	114	591	24	7.682	7.487

Table S1 Daily confirmed cases and daily deaths of COVID-19 in 214 nations from WHO

ID	Date and time	Country or region	Continent	Confirmed patients	Cumulative Confirmed patients	Daily dead patients	Daily cumulative dead patients	Lag days	Daily CFR	3DMA CFR
11233	2020-4-9 8:00	SE	EURO	726	8419	96	687	25	8.160	8.173
11234	2020-4-10 8:00	SE	EURO	722	9141	106	793	26	8.675	8.606
11235	2020-4-11 8:00	SE	EURO	544	9685	77	870	27	8.983	8.799
11236	2020-4-12 8:00	SE	EURO	466	10151	17	887	28	8.738	8.766
11237	2020-4-13 8:00	SE	EURO	332	10483	12	899	29	8.576	8.569
11238	2020-4-14 8:00	SE	EURO	465	10948	20	919	30	8.394	8.665
11239	2020-4-15 8:00	SE	EURO	497	11445	114	1033	31	9.026	9.169
11240	2020-4-16 8:00	SE	EURO	482	11927	170	1203	32	10.086	9.914
11241	2020-4-17 8:00	SE	EURO	613	12540	130	1333	33	10.630	10.437
11242	2020-4-18 8:00	SE	EURO	676	13216	67	1400	34	10.593	10.718
11243	2020-4-19 8:00	SE	EURO	606	13822	111	1511	35	10.932	10.744
11244	2020-4-20 8:00	SE	EURO	563	14385	29	1540	36	10.706	10.777
11245	2020-4-21 8:00	SE	EURO	392	14777	40	1580	37	10.692	10.972
11246	2020-4-22 8:00	SE	EURO	545	15322	185	1765	38	11.519	11.438
11247	2020-4-23 8:00	SE	EURO	682	16004	172	1937	39	12.103	11.895
11248	2020-4-24 8:00	SE	EURO	751	16755	84	2021	40	12.062	12.139
11249	2020-4-25 8:00	SE	EURO	812	17567	131	2152	41	12.250	12.124
11250	2020-4-26 8:00	SE	EURO	610	18177	40	2192	42	12.059	12.027
11251	2020-4-27 8:00	SE	EURO	463	18640	2	2194	43	11.770	11.948
11252	2020-4-28 8:00	SE	EURO	286	18926	80	2274	44	12.015	11.929
11253	2020-4-29 8:00	SE	EURO	695	19621	81	2355	45	12.002	12.048
11254	2020-4-30 8:00	SE	EURO	681	20302	107	2462	46	12.127	12.130
11255	2020-5-1 8:00	SE	EURO	790	21092	124	2586	47	12.261	12.239
11256	2020-5-2 8:00	SE	EURO	428	21520	67	2653	48	12.328	12.294
11257										
11258	2020-2-25 8:00	CH	EURO	1	1	0	0			
11259	2020-2-26 8:00	CH	EURO	0	1	0	0			
11260	2020-2-27 8:00	CH	EURO	5	6	0	0			
11261	2020-2-28 8:00	CH	EURO	4	10	0	0			
11262	2020-2-29 8:00	CH	EURO	8	18	0	0			
11263	2020-3-1 8:00	CH	EURO	6	24	0	0			
11264	2020-3-2 8:00	CH	EURO	6	30	0	0			
11265	2020-3-3 8:00	CH	EURO	7	37	0	0			
11266	2020-3-4 8:00	CH	EURO	20	57	0	0			
11267	2020-3-5 8:00	CH	EURO	29	86	1	1	0	1.163	0.821
11268	2020-3-6 8:00	CH	EURO	123	209	0	1	1	0.478	0.800

Table S1 Daily confirmed cases and daily deaths of COVID-19 in 214 nations from WHO

ID	Date and time	Country or region	Continent	Confirmed patients	Cumulative Confirmed patients	Daily dead patients	Daily cumulative dead patients	Lag days	Daily CFR	3DMA CFR
11269	2020-3-7 8:00	CH	EURO	55	264	1	2	2	0.758	0.613
11270	2020-3-8 8:00	CH	EURO	68	332	0	2	3	0.602	0.654
11271	2020-3-9 8:00	CH	EURO	0	332	0	2	4	0.602	0.605
11272	2020-3-10 8:00	CH	EURO	159	491	1	3	5	0.611	0.611
11273	2020-3-11 8:00	CH	EURO	154	645	1	4	6	0.620	0.566
11274	2020-3-12 8:00	CH	EURO	213	858	0	4	7	0.466	0.570
11275	2020-3-13 8:00	CH	EURO	267	1125	3	7	8	0.622	0.633
11276	2020-3-14 8:00	CH	EURO	234	1359	4	11	9	0.809	0.674
11277	2020-3-15 8:00	CH	EURO	841	2200	2	13	10	0.591	0.679
11278	2020-3-16 8:00	CH	EURO	0	2200	1	14	11	0.636	0.648
11279	2020-3-17 8:00	CH	EURO	450	2650	5	19	12	0.717	0.684
11280	2020-3-18 8:00	CH	EURO	360	3010	2	21	13	0.698	0.756
11281	2020-3-19 8:00	CH	EURO	853	3863	12	33	14	0.854	0.813
11282	2020-3-20 8:00	CH	EURO	977	4840	10	43	15	0.888	0.888
11283	2020-3-21 8:00	CH	EURO	1237	6077	13	56	16	0.922	0.910
11284	2020-3-22 8:00	CH	EURO	0	6077	0	56	17	0.922	0.901
11285	2020-3-23 8:00	CH	EURO	894	6971	4	60	18	0.861	0.869
11286	2020-3-24 8:00	CH	EURO	1044	8015	6	66	19	0.823	0.888
11287	2020-3-25 8:00	CH	EURO	774	8789	20	86	20	0.978	0.954
11288	2020-3-26 8:00	CH	EURO	925	9714	17	103	21	1.060	1.181
11289	2020-3-27 8:00	CH	EURO	1000	10714	58	161	22	1.503	1.397
11290	2020-3-28 8:00	CH	EURO	1390	12104	36	197	23	1.628	1.639
11291	2020-3-29 8:00	CH	EURO	1048	13152	38	235	24	1.787	1.738
11292	2020-3-30 8:00	CH	EURO	1122	14274	22	257	25	1.800	1.834
11293	2020-3-31 8:00	CH	EURO	1138	15412	38	295	26	1.914	2.010
11294	2020-4-1 8:00	CH	EURO	696	16108	78	373	27	2.316	2.148
11295	2020-4-2 8:00	CH	EURO	962	17070	5	378	28	2.214	2.327
11296	2020-4-3 8:00	CH	EURO	1774	18844	84	462	29	2.452	2.457
11297	2020-4-4 8:00	CH	EURO	862	19706	71	533	30	2.705	2.682
11298	2020-4-5 8:00	CH	EURO	783	20489	59	592	31	2.889	2.879
11299	2020-4-6 8:00	CH	EURO	576	21065	49	641	32	3.043	2.968
11300	2020-4-7 8:00	CH	EURO	509	21574	0	641	33	2.971	2.969
11301	2020-4-8 8:00	CH	EURO	590	22164	0	641	34	2.892	2.989
11302	2020-4-9 8:00	CH	EURO	546	22710	64	705	35	3.104	3.071
11303	2020-4-10 8:00	CH	EURO	785	23495	51	756	36	3.218	3.215
11304	2020-4-11 8:00	CH	EURO	733	24228	49	805	37	3.323	3.296

Table S1 Daily confirmed cases and daily deaths of COVID-19 in 214 nations from WHO

ID	Date and time	Country or region	Continent	Confirmed patients	Cumulative Confirmed patients	Daily dead patients	Daily cumulative dead patients	Lag days	Daily CFR	3DMA CFR
11305	2020-4-12 8:00	CH	EURO	592	24820	26	831	38	3.348	3.358
11306	2020-4-13 8:00	CH	EURO	400	25220	27	858	39	3.402	3.407
11307	2020-4-14 8:00	CH	EURO	279	25499	27	885	40	3.471	3.456
11308	2020-4-15 8:00	CH	EURO	254	25753	15	900	41	3.495	3.553
11309	2020-4-16 8:00	CH	EURO	583	26336	73	973	42	3.695	3.667
11310	2020-4-17 8:00	CH	EURO	315	26651	43	1016	43	3.812	3.809
11311	2020-4-18 8:00	CH	EURO	346	26997	42	1058	44	3.919	3.931
11312	2020-4-19 8:00	CH	EURO	325	27322	52	1110	45	4.063	4.027
11313	2020-4-20 8:00	CH	EURO	336	27658	24	1134	46	4.100	4.086
11314	2020-4-21 8:00	CH	EURO	204	27862	7	1141	47	4.095	4.145
11315	2020-4-22 8:00	CH	EURO	119	27981	45	1186	48	4.239	4.216
11316	2020-4-23 8:00	CH	EURO	205	28186	30	1216	49	4.314	4.337
11317	2020-4-24 8:00	CH	EURO	228	28414	51	1267	50	4.459	4.449
11318	2020-4-25 8:00	CH	EURO	181	28595	41	1308	51	4.574	4.548
11319	2020-4-26 8:00	CH	EURO	383	28978	28	1336	52	4.610	4.598
11320	2020-4-27 8:00	CH	EURO	0	28978	0	1336	53	4.610	4.623
11321	2020-4-28 8:00	CH	EURO	103	29081	16	1352	54	4.649	4.662
11322	2020-4-29 8:00	CH	EURO	100	29181	27	1379	55	4.726	4.724
11323	2020-4-30 8:00	CH	EURO	143	29324	28	1407	56	4.798	4.781
11324	2020-5-1 8:00	CH	EURO	179	29503	15	1422	57	4.820	4.820
11325	2020-5-2 8:00	CH	EURO	119	29622	12	1434	58	4.841	4.830
11326										
11327	2020-3-23 8:00	SY	EMRO	1	1	0	0			
11328	2020-3-24 8:00	SY	EMRO	0	1	0	0			
11329	2020-3-25 8:00	SY	EMRO	0	1	0	0			
11330	2020-3-26 8:00	SY	EMRO	4	5	0	0			
11331	2020-3-27 8:00	SY	EMRO	0	5	0	0			
11332	2020-3-28 8:00	SY	EMRO	0	5	0	0			
11333	2020-3-29 8:00	SY	EMRO	4	9	1	1	0	11.111	11.111
11334	2020-3-30 8:00	SY	EMRO	0	9	0	1	1	11.111	14.074
11335	2020-3-31 8:00	SY	EMRO	1	10	1	2	2	20.000	17.037
11336	2020-4-1 8:00	SY	EMRO	0	10	0	2	3	20.000	20.000
11337	2020-4-2 8:00	SY	EMRO	0	10	0	2	4	20.000	17.500
11338	2020-4-3 8:00	SY	EMRO	6	16	0	2	5	12.500	15.000
11339	2020-4-4 8:00	SY	EMRO	0	16	0	2	6	12.500	11.842
11340	2020-4-5 8:00	SY	EMRO	3	19	0	2	7	10.526	11.184

Table S1 Daily confirmed cases and daily deaths of COVID-19 in 214 nations from WHO

ID	Date and time	Country or region	Continent	Confirmed patients	Cumulative Confirmed patients	Daily dead patients	Daily cumulative dead patients	Lag days	Daily CFR	3DMA CFR
11341	2020-4-6 8:00	SY	EMRO	0	19	0	2	8	10.526	10.526
11342	2020-4-7 8:00	SY	EMRO	0	19	0	2	9	10.526	10.526
11343	2020-4-8 8:00	SY	EMRO	0	19	0	2	10	10.526	10.526
11344	2020-4-9 8:00	SY	EMRO	0	19	0	2	11	10.526	10.526
11345	2020-4-10 8:00	SY	EMRO	0	19	0	2	12	10.526	9.684
11346	2020-4-11 8:00	SY	EMRO	6	25	0	2	13	8.000	8.842
11347	2020-4-12 8:00	SY	EMRO	0	25	0	2	14	8.000	8.000
11348	2020-4-13 8:00	SY	EMRO	0	25	0	2	15	8.000	8.000
11349	2020-4-14 8:00	SY	EMRO	0	25	0	2	16	8.000	7.632
11350	2020-4-15 8:00	SY	EMRO	4	29	0	2	17	6.897	6.986
11351	2020-4-16 8:00	SY	EMRO	4	33	0	2	18	6.061	6.339
11352	2020-4-17 8:00	SY	EMRO	0	33	0	2	19	6.061	5.795
11353	2020-4-18 8:00	SY	EMRO	5	38	0	2	20	5.263	5.529
11354	2020-4-19 8:00	SY	EMRO	0	38	0	2	21	5.263	6.073
11355	2020-4-20 8:00	SY	EMRO	1	39	1	3	22	7.692	6.883
11356	2020-4-21 8:00	SY	EMRO	0	39	0	3	23	7.692	7.509
11357	2020-4-22 8:00	SY	EMRO	3	42	0	3	24	7.143	7.326
11358	2020-4-23 8:00	SY	EMRO	0	42	0	3	25	7.143	7.143
11359	2020-4-24 8:00	SY	EMRO	0	42	0	3	26	7.143	7.143
11360	2020-4-25 8:00	SY	EMRO	0	42	0	3	27	7.143	7.143
11361	2020-4-26 8:00	SY	EMRO	0	42	0	3	28	7.143	7.087
11362	2020-4-27 8:00	SY	EMRO	1	43	0	3	29	6.977	7.032
11363	2020-4-28 8:00	SY	EMRO	0	43	0	3	30	6.977	6.977
11364	2020-4-29 8:00	SY	EMRO	0	43	0	3	31	6.977	6.977
11365	2020-4-30 8:00	SY	EMRO	0	43	0	3	32	6.977	6.977
11366	2020-5-1 8:00	SY	EMRO	0	43	0	3	33	6.977	6.924
11367	2020-5-2 8:00	SY	EMRO	1	44	0	3	34	6.818	6.897
11368										
11369	2020-5-1 8:00	TJ	EURO	15	15	0	0			
11370	2020-5-2 8:00	TJ	EURO	17	32	0	0			
11371										
11372	2020-1-13 8:00	TH	SEARO	1	1	0	0			
11373	2020-1-14 8:00	TH	SEARO	0	1	0	0			
11374	2020-1-15 8:00	TH	SEARO	0	1	0	0			
11375	2020-1-16 8:00	TH	SEARO	0	1	0	0			
11376	2020-1-17 8:00	TH	SEARO	1	2	0	0			

Table S1 Daily confirmed cases and daily deaths of COVID-19 in 214 nations from WHO

ID	Date and time	Country or region	Continent	Confirmed patients	Cumulative Confirmed patients	Daily dead patients	Daily cumulative dead patients	Lag days	Daily CFR	3DMA CFR
11377	2020-1-18 8:00	TH	SEARO	1	3	0	0			
11378	2020-1-19 8:00	TH	SEARO	0	3	0	0			
11379	2020-1-20 8:00	TH	SEARO	0	3	0	0			
11380	2020-1-21 8:00	TH	SEARO	0	3	0	0			
11381	2020-1-22 8:00	TH	SEARO	0	3	0	0			
11382	2020-1-23 8:00	TH	SEARO	0	3	0	0			
11383	2020-1-24 8:00	TH	SEARO	1	4	0	0			
11384	2020-1-25 8:00	TH	SEARO	0	4	0	0			
11385	2020-1-26 8:00	TH	SEARO	1	5	0	0			
11386	2020-1-27 8:00	TH	SEARO	3	8	0	0			
11387	2020-1-28 8:00	TH	SEARO	6	14	0	0			
11388	2020-1-29 8:00	TH	SEARO	0	14	0	0			
11389	2020-1-30 8:00	TH	SEARO	0	14	0	0			
11390	2020-1-31 8:00	TH	SEARO	5	19	0	0			
11391	2020-2-1 8:00	TH	SEARO	0	19	0	0			
11392	2020-2-2 8:00	TH	SEARO	0	19	0	0			
11393	2020-2-3 8:00	TH	SEARO	0	19	0	0			
11394	2020-2-4 8:00	TH	SEARO	6	25	0	0			
11395	2020-2-5 8:00	TH	SEARO	0	25	0	0			
11396	2020-2-6 8:00	TH	SEARO	0	25	0	0			
11397	2020-2-7 8:00	TH	SEARO	0	25	0	0			
11398	2020-2-8 8:00	TH	SEARO	7	32	0	0			
11399	2020-2-9 8:00	TH	SEARO	0	32	0	0			
11400	2020-2-10 8:00	TH	SEARO	0	32	0	0			
11401	2020-2-11 8:00	TH	SEARO	1	33	0	0			
11402	2020-2-12 8:00	TH	SEARO	0	33	0	0			
11403	2020-2-13 8:00	TH	SEARO	0	33	0	0			
11404	2020-2-14 8:00	TH	SEARO	0	33	0	0			
11405	2020-2-15 8:00	TH	SEARO	1	34	0	0			
11406	2020-2-16 8:00	TH	SEARO	0	34	0	0			
11407	2020-2-17 8:00	TH	SEARO	1	35	0	0			
11408	2020-2-18 8:00	TH	SEARO	0	35	0	0			
11409	2020-2-19 8:00	TH	SEARO	0	35	0	0			
11410	2020-2-20 8:00	TH	SEARO	0	35	0	0			
11411	2020-2-21 8:00	TH	SEARO	0	35	0	0			
11412	2020-2-22 8:00	TH	SEARO	0	35	0	0			

Table S1 Daily confirmed cases and daily deaths of COVID-19 in 214 nations from WHO

ID	Date and time	Country or region	Continent	Confirmed patients	Cumulative Confirmed patients	Daily dead patients	Daily cumulative dead patients	Lag days	Daily CFR	3DMA CFR
11413	2020-2-23 8:00	TH	SEARO	0	35	0	0			
11414	2020-2-24 8:00	TH	SEARO	0	35	0	0			
11415	2020-2-25 8:00	TH	SEARO	2	37	0	0			
11416	2020-2-26 8:00	TH	SEARO	3	40	0	0			
11417	2020-2-27 8:00	TH	SEARO	0	40	0	0			
11418	2020-2-28 8:00	TH	SEARO	1	41	0	0			
11419	2020-2-29 8:00	TH	SEARO	1	42	0	0			
11420	2020-3-1 8:00	TH	SEARO	0	42	1	1	0	2.381	2.353
11421	2020-3-2 8:00	TH	SEARO	1	43	0	1	1	2.326	2.344
11422	2020-3-3 8:00	TH	SEARO	0	43	0	1	2	2.326	2.326
11423	2020-3-4 8:00	TH	SEARO	0	43	0	1	3	2.326	2.260
11424	2020-3-5 8:00	TH	SEARO	4	47	0	1	4	2.128	2.179
11425	2020-3-6 8:00	TH	SEARO	1	48	0	1	5	2.083	2.070
11426	2020-3-7 8:00	TH	SEARO	2	50	0	1	6	2.000	2.028
11427	2020-3-8 8:00	TH	SEARO	0	50	0	1	7	2.000	2.000
11428	2020-3-9 8:00	TH	SEARO	0	50	0	1	8	2.000	1.962
11429	2020-3-10 8:00	TH	SEARO	3	53	0	1	9	1.887	1.861
11430	2020-3-11 8:00	TH	SEARO	6	59	0	1	10	1.695	1.670
11431	2020-3-12 8:00	TH	SEARO	11	70	0	1	11	1.429	1.486
11432	2020-3-13 8:00	TH	SEARO	5	75	0	1	12	1.333	1.365
11433	2020-3-14 8:00	TH	SEARO	0	75	0	1	13	1.333	1.181
11434	2020-3-15 8:00	TH	SEARO	39	114	0	1	14	0.877	0.964
11435	2020-3-16 8:00	TH	SEARO	33	147	0	1	15	0.680	0.707
11436	2020-3-17 8:00	TH	SEARO	30	177	0	1	16	0.565	0.572
11437	2020-3-18 8:00	TH	SEARO	35	212	0	1	17	0.472	0.449
11438	2020-3-19 8:00	TH	SEARO	110	322	0	1	18	0.311	0.364
11439	2020-3-20 8:00	TH	SEARO	0	322	0	1	19	0.311	0.288
11440	2020-3-21 8:00	TH	SEARO	89	411	0	1	20	0.243	0.240
11441	2020-3-22 8:00	TH	SEARO	188	599	0	1	21	0.167	0.183
11442	2020-3-23 8:00	TH	SEARO	122	721	0	1	22	0.139	0.263
11443	2020-3-24 8:00	TH	SEARO	106	827	3	4	23	0.484	0.350
11444	2020-3-25 8:00	TH	SEARO	107	934	0	4	24	0.428	0.432
11445	2020-3-26 8:00	TH	SEARO	111	1045	0	4	25	0.383	0.417
11446	2020-3-27 8:00	TH	SEARO	91	1136	1	5	26	0.440	0.421
11447	2020-3-28 8:00	TH	SEARO	0	1136	0	5	27	0.440	0.462
11448	2020-3-29 8:00	TH	SEARO	252	1388	2	7	28	0.504	0.512

Table S1 Daily confirmed cases and daily deaths of COVID-19 in 214 nations from WHO

ID	Date and time	Country or region	Continent	Confirmed patients	Cumulative Confirmed patients	Daily dead patients	Daily cumulative dead patients	Lag days	Daily CFR	3DMA CFR
11449	2020-3-30 8:00	TH	SEARO	136	1524	2	9	29	0.591	0.567
11450	2020-3-31 8:00	TH	SEARO	127	1651	1	10	30	0.606	0.625
11451	2020-4-1 8:00	TH	SEARO	120	1771	2	12	31	0.678	0.694
11452	2020-4-2 8:00	TH	SEARO	104	1875	3	15	32	0.800	0.813
11453	2020-4-3 8:00	TH	SEARO	103	1978	4	19	33	0.961	0.909
11454	2020-4-4 8:00	TH	SEARO	89	2067	1	20	34	0.968	0.996
11455	2020-4-5 8:00	TH	SEARO	102	2169	3	23	35	1.060	1.066
11456	2020-4-6 8:00	TH	SEARO	51	2220	3	26	36	1.171	1.142
11457	2020-4-7 8:00	TH	SEARO	38	2258	1	27	37	1.196	1.211
11458	2020-4-8 8:00	TH	SEARO	111	2369	3	30	38	1.266	1.261
11459	2020-4-9 8:00	TH	SEARO	54	2423	2	32	39	1.321	1.307
11460	2020-4-10 8:00	TH	SEARO	50	2473	1	33	40	1.334	1.348
11461	2020-4-11 8:00	TH	SEARO	45	2518	2	35	41	1.390	1.405
11462	2020-4-12 8:00	TH	SEARO	33	2551	3	38	42	1.490	1.477
11463	2020-4-13 8:00	TH	SEARO	28	2579	2	40	43	1.551	1.537
11464	2020-4-14 8:00	TH	SEARO	34	2613	1	41	44	1.569	1.582
11465	2020-4-15 8:00	TH	SEARO	30	2643	2	43	45	1.627	1.639
11466	2020-4-16 8:00	TH	SEARO	29	2672	3	46	46	1.722	1.696
11467	2020-4-17 8:00	TH	SEARO	28	2700	1	47	47	1.741	1.727
11468	2020-4-18 8:00	TH	SEARO	33	2733	0	47	48	1.720	1.720
11469	2020-4-19 8:00	TH	SEARO	32	2765	0	47	49	1.700	1.701
11470	2020-4-20 8:00	TH	SEARO	27	2792	0	47	50	1.683	1.697
11471	2020-4-21 8:00	TH	SEARO	19	2811	1	48	51	1.708	1.708
11472	2020-4-22 8:00	TH	SEARO	15	2826	1	49	52	1.734	1.734
11473	2020-4-23 8:00	TH	SEARO	13	2839	1	50	53	1.761	1.749
11474	2020-4-24 8:00	TH	SEARO	15	2854	0	50	54	1.752	1.756
11475	2020-4-25 8:00	TH	SEARO	53	2907	1	51	55	1.754	1.751
11476	2020-4-26 8:00	TH	SEARO	15	2922	0	51	56	1.745	1.758
11477	2020-4-27 8:00	TH	SEARO	9	2931	1	52	57	1.774	1.786
11478	2020-4-28 8:00	TH	SEARO	7	2938	2	54	58	1.838	1.815
11479	2020-4-29 8:00	TH	SEARO	9	2947	0	54	59	1.832	1.833
11480	2020-4-30 8:00	TH	SEARO	7	2954	0	54	60	1.828	1.828
11481	2020-5-1 8:00	TH	SEARO	6	2960	0	54	61	1.824	1.824
11482	2020-5-2 8:00	TH	SEARO	6	2966	0	54	62	1.821	1.822
11483										
11484	2020-1-31 8:00	GB	EURO	2	2	0	0			

Table S1 Daily confirmed cases and daily deaths of COVID-19 in 214 nations from WHO

ID	Date and time	Country or region	Continent	Confirmed patients	Cumulative Confirmed patients	Daily dead patients	Daily cumulative dead patients	Lag days	Daily CFR	3DMA CFR
11485	2020-2-1 8:00	GB	EURO	0	2	0	0			
11486	2020-2-2 8:00	GB	EURO	0	2	0	0			
11487	2020-2-3 8:00	GB	EURO	0	2	0	0			
11488	2020-2-4 8:00	GB	EURO	0	2	0	0			
11489	2020-2-5 8:00	GB	EURO	0	2	0	0			
11490	2020-2-6 8:00	GB	EURO	1	3	0	0			
11491	2020-2-7 8:00	GB	EURO	0	3	0	0			
11492	2020-2-8 8:00	GB	EURO	0	3	0	0			
11493	2020-2-9 8:00	GB	EURO	1	4	0	0			
11494	2020-2-10 8:00	GB	EURO	4	8	0	0			
11495	2020-2-11 8:00	GB	EURO	0	8	0	0			
11496	2020-2-12 8:00	GB	EURO	1	9	0	0			
11497	2020-2-13 8:00	GB	EURO	0	9	0	0			
11498	2020-2-14 8:00	GB	EURO	0	9	0	0			
11499	2020-2-15 8:00	GB	EURO	0	9	0	0			
11500	2020-2-16 8:00	GB	EURO	0	9	0	0			
11501	2020-2-17 8:00	GB	EURO	0	9	0	0			
11502	2020-2-18 8:00	GB	EURO	0	9	0	0			
11503	2020-2-19 8:00	GB	EURO	0	9	0	0			
11504	2020-2-20 8:00	GB	EURO	0	9	0	0			
11505	2020-2-21 8:00	GB	EURO	0	9	0	0			
11506	2020-2-22 8:00	GB	EURO	0	9	0	0			
11507	2020-2-23 8:00	GB	EURO	4	13	0	0			
11508	2020-2-24 8:00	GB	EURO	0	13	0	0			
11509	2020-2-25 8:00	GB	EURO	0	13	0	0			
11510	2020-2-26 8:00	GB	EURO	0	13	0	0			
11511	2020-2-27 8:00	GB	EURO	3	16	0	0			
11512	2020-2-28 8:00	GB	EURO	4	20	0	0			
11513	2020-2-29 8:00	GB	EURO	3	23	0	0			
11514	2020-3-1 8:00	GB	EURO	13	36	0	0			
11515	2020-3-2 8:00	GB	EURO	3	39	0	0			
11516	2020-3-3 8:00	GB	EURO	12	51	0	0			
11517	2020-3-4 8:00	GB	EURO	38	89	0	0			
11518	2020-3-5 8:00	GB	EURO	29	118	0	0			
11519	2020-3-6 8:00	GB	EURO	49	167	1	1	0	0.599	0.776
11520	2020-3-7 8:00	GB	EURO	43	210	1	2	1	0.952	0.758

Table S1 Daily confirmed cases and daily deaths of COVID-19 in 214 nations from WHO

ID	Date and time	Country or region	Continent	Confirmed patients	Cumulative Confirmed patients	Daily dead patients	Daily cumulative dead patients	Lag days	Daily CFR	3DMA CFR
11521	2020-3-8 8:00	GB	EURO	67	277	0	2	2	0.722	0.868
11522	2020-3-9 8:00	GB	EURO	46	323	1	3	3	0.929	1.086
11523	2020-3-10 8:00	GB	EURO	50	373	3	6	4	1.609	1.281
11524	2020-3-11 8:00	GB	EURO	87	460	0	6	5	1.304	1.420
11525	2020-3-12 8:00	GB	EURO	134	594	2	8	6	1.347	1.299
11526	2020-3-13 8:00	GB	EURO	208	802	2	10	7	1.247	1.476
11527	2020-3-14 8:00	GB	EURO	342	1144	11	21	8	1.836	1.864
11528	2020-3-15 8:00	GB	EURO	251	1395	14	35	9	2.509	2.633
11529	2020-3-16 8:00	GB	EURO	152	1547	20	55	10	3.555	3.045
11530	2020-3-17 8:00	GB	EURO	407	1954	5	60	11	3.071	3.514
11531	2020-3-18 8:00	GB	EURO	676	2630	43	103	12	3.916	3.794
11532	2020-3-19 8:00	GB	EURO	647	3277	41	144	13	4.394	4.251
11533	2020-3-20 8:00	GB	EURO	706	3983	33	177	14	4.444	4.494
11534	2020-3-21 8:00	GB	EURO	1035	5018	56	233	15	4.643	4.577
11535	2020-3-22 8:00	GB	EURO	0	5018	0	233	16	4.643	4.743
11536	2020-3-23 8:00	GB	EURO	669	5687	48	281	17	4.941	4.873
11537	2020-3-24 8:00	GB	EURO	967	6654	54	335	18	5.035	5.066
11538	2020-3-25 8:00	GB	EURO	1427	8081	87	422	19	5.222	5.038
11539	2020-3-26 8:00	GB	EURO	1452	9533	41	463	20	4.857	5.012
11540	2020-3-27 8:00	GB	EURO	2129	11662	115	578	21	4.956	5.010
11541	2020-3-28 8:00	GB	EURO	2885	14547	181	759	22	5.218	5.378
11542	2020-3-29 8:00	GB	EURO	2546	17093	260	1019	23	5.962	5.823
11543	2020-3-30 8:00	GB	EURO	2433	19526	209	1228	24	6.289	6.203
11544	2020-3-31 8:00	GB	EURO	2619	22145	180	1408	25	6.358	6.586
11545	2020-4-1 8:00	GB	EURO	3009	25154	381	1789	26	7.112	7.353
11546	2020-4-2 8:00	GB	EURO	4324	29478	743	2532	27	8.589	8.121
11547	2020-4-3 8:00	GB	EURO	4244	33722	389	2921	28	8.662	8.899
11548	2020-4-4 8:00	GB	EURO	4450	38172	684	3605	29	9.444	9.466
11549	2020-4-5 8:00	GB	EURO	3735	41907	708	4313	30	10.292	10.019
11550	2020-4-6 8:00	GB	EURO	5903	47810	621	4934	31	10.320	10.341
11551	2020-4-7 8:00	GB	EURO	3802	51612	439	5373	32	10.410	10.626
11552	2020-4-8 8:00	GB	EURO	3634	55246	786	6159	33	11.148	11.081
11553	2020-4-9 8:00	GB	EURO	5491	60737	938	7097	34	11.685	11.697
11554	2020-4-10 8:00	GB	EURO	4344	65081	881	7978	35	12.259	12.230
11555	2020-4-11 8:00	GB	EURO	5195	70276	980	8958	36	12.747	12.502
11556	2020-4-12 8:00	GB	EURO	8719	78995	917	9875	37	12.501	12.613

Table S1 Daily confirmed cases and daily deaths of COVID-19 in 214 nations from WHO

ID	Date and time	Country or region	Continent	Confirmed patients	Cumulative Confirmed patients	Daily dead patients	Daily cumulative dead patients	Lag days	Daily CFR	3DMA CFR
11557	2020-4-13 8:00	GB	EURO	5288	84283	737	10612	38	12.591	12.625
11558	2020-4-14 8:00	GB	EURO	4342	88625	717	11329	39	12.783	12.757
11559	2020-4-15 8:00	GB	EURO	5252	93877	778	12107	40	12.897	12.915
11560	2020-4-16 8:00	GB	EURO	4603	98480	761	12868	41	13.067	13.093
11561	2020-4-17 8:00	GB	EURO	4617	103097	861	13729	42	13.317	13.264
11562	2020-4-18 8:00	GB	EURO	5599	108696	847	14576	43	13.410	13.422
11563	2020-4-19 8:00	GB	EURO	5525	114221	888	15464	44	13.539	13.441
11564	2020-4-20 8:00	GB	EURO	5850	120071	596	16060	45	13.375	13.383
11565	2020-4-21 8:00	GB	EURO	4676	124747	449	16509	46	13.234	13.348
11566	2020-4-22 8:00	GB	EURO	4301	129048	828	17337	47	13.435	13.409
11567	2020-4-23 8:00	GB	EURO	4451	133499	763	18100	48	13.558	13.521
11568	2020-4-24 8:00	GB	EURO	4583	138082	638	18738	49	13.570	13.575
11569	2020-4-25 8:00	GB	EURO	5386	143468	768	19506	50	13.596	13.620
11570	2020-4-26 8:00	GB	EURO	4913	148381	813	20319	51	13.694	13.618
11571	2020-4-27 8:00	GB	EURO	4463	152844	413	20732	52	13.564	13.560
11572	2020-4-28 8:00	GB	EURO	4309	157153	360	21092	53	13.421	13.479
11573	2020-4-29 8:00	GB	EURO	3996	161149	586	21678	54	13.452	14.223
11574	2020-4-30 8:00	GB	EURO	4076	165225	4419	26097	55	15.795	14.960
11575	2020-5-1 8:00	GB	EURO	6032	171257	674	26771	56	15.632	15.643
11576	2020-5-2 8:00	GB	EURO	6201	177458	739	27510	57	15.502	15.567
11577										
11578	2020-3-20 8:00	TL	SEARO	1	1	0	0			
11579	2020-3-21 8:00	TL	SEARO	0	1	0	0			
11580	2020-3-22 8:00	TL	SEARO	0	1	0	0			
11581	2020-3-23 8:00	TL	SEARO	0	1	0	0			
11582	2020-3-24 8:00	TL	SEARO	0	1	0	0			
11583	2020-3-25 8:00	TL	SEARO	0	1	0	0			
11584	2020-3-26 8:00	TL	SEARO	0	1	0	0			
11585	2020-3-27 8:00	TL	SEARO	0	1	0	0			
11586	2020-3-28 8:00	TL	SEARO	0	1	0	0			
11587	2020-3-29 8:00	TL	SEARO	0	1	0	0			
11588	2020-3-30 8:00	TL	SEARO	0	1	0	0			
11589	2020-3-31 8:00	TL	SEARO	0	1	0	0			
11590	2020-4-1 8:00	TL	SEARO	0	1	0	0			
11591	2020-4-2 8:00	TL	SEARO	0	1	0	0			
11592	2020-4-3 8:00	TL	SEARO	0	1	0	0			

Table S1 Daily confirmed cases and daily deaths of COVID-19 in 214 nations from WHO

ID	Date and time	Country or region	Continent	Confirmed patients	Cumulative Confirmed patients	Daily dead patients	Daily cumulative dead patients	Lag days	Daily CFR	3DMA CFR
11593	2020-4-4 8:00	TL	SEARO	0	1	0	0			
11594	2020-4-5 8:00	TL	SEARO	0	1	0	0			
11595	2020-4-6 8:00	TL	SEARO	0	1	0	0			
11596	2020-4-7 8:00	TL	SEARO	0	1	0	0			
11597	2020-4-8 8:00	TL	SEARO	0	1	0	0			
11598	2020-4-9 8:00	TL	SEARO	0	1	0	0			
11599	2020-4-10 8:00	TL	SEARO	0	1	0	0			
11600	2020-4-11 8:00	TL	SEARO	1	2	0	0			
11601	2020-4-12 8:00	TL	SEARO	0	2	0	0			
11602	2020-4-13 8:00	TL	SEARO	2	4	0	0			
11603	2020-4-14 8:00	TL	SEARO	2	6	0	0			
11604	2020-4-15 8:00	TL	SEARO	0	6	0	0			
11605	2020-4-16 8:00	TL	SEARO	12	18	0	0			
11606	2020-4-17 8:00	TL	SEARO	0	18	0	0			
11607	2020-4-18 8:00	TL	SEARO	0	18	0	0			
11608	2020-4-19 8:00	TL	SEARO	1	19	0	0			
11609	2020-4-20 8:00	TL	SEARO	3	22	0	0			
11610	2020-4-21 8:00	TL	SEARO	1	23	0	0			
11611	2020-4-22 8:00	TL	SEARO	0	23	0	0			
11612	2020-4-23 8:00	TL	SEARO	0	23	0	0			
11613	2020-4-24 8:00	TL	SEARO	1	24	0	0			
11614	2020-4-25 8:00	TL	SEARO	0	24	0	0			
11615	2020-4-26 8:00	TL	SEARO	0	24	0	0			
11616	2020-4-27 8:00	TL	SEARO	0	24	0	0			
11617	2020-4-28 8:00	TL	SEARO	0	24	0	0			
11618	2020-4-29 8:00	TL	SEARO	0	24	0	0			
11619	2020-4-30 8:00	TL	SEARO	0	24	0	0			
11620	2020-5-1 8:00	TL	SEARO	0	24	0	0			
11621	2020-5-2 8:00	TL	SEARO	0	24	0	0			
11622										
11623	2020-3-7 8:00	TG	AFRO	1	1	0	0			
11624	2020-3-8 8:00	TG	AFRO	0	1	0	0			
11625	2020-3-9 8:00	TG	AFRO	0	1	0	0			
11626	2020-3-10 8:00	TG	AFRO	0	1	0	0			
11627	2020-3-11 8:00	TG	AFRO	0	1	0	0			
11628	2020-3-12 8:00	TG	AFRO	0	1	0	0			

Table S1 Daily confirmed cases and daily deaths of COVID-19 in 214 nations from WHO

ID	Date and time	Country or region	Continent	Confirmed patients	Cumulative Confirmed patients	Daily dead patients	Daily cumulative dead patients	Lag days	Daily CFR	3DMA CFR
11629	2020-3-13 8:00	TG	AFRO	0	1	0	0			
11630	2020-3-14 8:00	TG	AFRO	0	1	0	0			
11631	2020-3-15 8:00	TG	AFRO	0	1	0	0			
11632	2020-3-16 8:00	TG	AFRO	0	1	0	0			
11633	2020-3-17 8:00	TG	AFRO	0	1	0	0			
11634	2020-3-18 8:00	TG	AFRO	0	1	0	0			
11635	2020-3-19 8:00	TG	AFRO	8	9	0	0			
11636	2020-3-20 8:00	TG	AFRO	0	9	0	0			
11637	2020-3-21 8:00	TG	AFRO	6	15	0	0			
11638	2020-3-22 8:00	TG	AFRO	0	15	0	0			
11639	2020-3-23 8:00	TG	AFRO	1	16	0	0			
11640	2020-3-24 8:00	TG	AFRO	2	18	0	0			
11641	2020-3-25 8:00	TG	AFRO	2	20	0	0			
11642	2020-3-26 8:00	TG	AFRO	3	23	0	0			
11643	2020-3-27 8:00	TG	AFRO	1	24	0	0			
11644	2020-3-28 8:00	TG	AFRO	1	25	0	0			
11645	2020-3-29 8:00	TG	AFRO	3	28	1	1	0	3.571	3.571
11646	2020-3-30 8:00	TG	AFRO	0	28	0	1	1	3.571	3.361
11647	2020-3-31 8:00	TG	AFRO	6	34	0	1	2	2.941	3.151
11648	2020-4-1 8:00	TG	AFRO	0	34	0	1	3	2.941	3.813
11649	2020-4-2 8:00	TG	AFRO	2	36	1	2	4	5.556	4.542
11650	2020-4-3 8:00	TG	AFRO	3	39	0	2	5	5.128	5.271
11651	2020-4-4 8:00	TG	AFRO	0	39	0	2	6	5.128	5.919
11652	2020-4-5 8:00	TG	AFRO	1	40	1	3	7	7.500	6.482
11653	2020-4-6 8:00	TG	AFRO	4	44	0	3	8	6.818	7.045
11654	2020-4-7 8:00	TG	AFRO	0	44	0	3	9	6.818	6.270
11655	2020-4-8 8:00	TG	AFRO	14	58	0	3	10	5.172	5.425
11656	2020-4-9 8:00	TG	AFRO	12	70	0	3	11	4.286	4.523
11657	2020-4-10 8:00	TG	AFRO	3	73	0	3	12	4.110	4.168
11658	2020-4-11 8:00	TG	AFRO	0	73	0	3	13	4.110	4.110
11659	2020-4-12 8:00	TG	AFRO	0	73	0	3	14	4.110	4.056
11660	2020-4-13 8:00	TG	AFRO	3	76	0	3	15	3.947	4.001
11661	2020-4-14 8:00	TG	AFRO	0	76	0	3	16	3.947	3.930
11662	2020-4-15 8:00	TG	AFRO	1	77	0	3	17	3.896	3.913
11663	2020-4-16 8:00	TG	AFRO	0	77	0	3	18	3.896	4.655
11664	2020-4-17 8:00	TG	AFRO	4	81	2	5	19	6.173	5.364

Table S1 Daily confirmed cases and daily deaths of COVID-19 in 214 nations from WHO

ID	Date and time	Country or region	Continent	Confirmed patients	Cumulative Confirmed patients	Daily dead patients	Daily cumulative dead patients	Lag days	Daily CFR	3DMA CFR
11665	2020-4-18 8:00	TG	AFRO	2	83	0	5	20	6.024	6.074
11666	2020-4-19 8:00	TG	AFRO	0	83	0	5	21	6.024	6.024
11667	2020-4-20 8:00	TG	AFRO	0	83	0	5	22	6.024	6.000
11668	2020-4-21 8:00	TG	AFRO	1	84	0	5	23	5.952	6.318
11669	2020-4-22 8:00	TG	AFRO	2	86	1	6	24	6.977	6.582
11670	2020-4-23 8:00	TG	AFRO	2	88	0	6	25	6.818	6.871
11671	2020-4-24 8:00	TG	AFRO	0	88	0	6	26	6.818	6.768
11672	2020-4-25 8:00	TG	AFRO	2	90	0	6	27	6.667	6.578
11673	2020-4-26 8:00	TG	AFRO	6	96	0	6	28	6.250	6.346
11674	2020-4-27 8:00	TG	AFRO	2	98	0	6	29	6.122	6.144
11675	2020-4-28 8:00	TG	AFRO	1	99	0	6	30	6.061	6.081
11676	2020-4-29 8:00	TG	AFRO	0	99	0	6	31	6.061	6.181
11677	2020-4-30 8:00	TG	AFRO	10	109	1	7	32	6.422	6.747
11678	2020-5-1 8:00	TG	AFRO	7	116	2	9	33	7.759	7.166
11679	2020-5-2 8:00	TG	AFRO	7	123	0	9	34	7.317	7.538
11680										
11681	2020-3-13 8:00	TT	AMRO	1	1	0	0			
11682	2020-3-14 8:00	TT	AMRO	0	1	0	0			
11683	2020-3-15 8:00	TT	AMRO	1	2	0	0			
11684	2020-3-16 8:00	TT	AMRO	0	2	0	0			
11685	2020-3-17 8:00	TT	AMRO	3	5	0	0			
11686	2020-3-18 8:00	TT	AMRO	2	7	0	0			
11687	2020-3-19 8:00	TT	AMRO	2	9	0	0			
11688	2020-3-20 8:00	TT	AMRO	0	9	0	0			
11689	2020-3-21 8:00	TT	AMRO	0	9	0	0			
11690	2020-3-22 8:00	TT	AMRO	0	9	0	0			
11691	2020-3-23 8:00	TT	AMRO	41	50	0	0			
11692	2020-3-24 8:00	TT	AMRO	7	57	0	0			
11693	2020-3-25 8:00	TT	AMRO	0	57	0	0			
11694	2020-3-26 8:00	TT	AMRO	4	61	1	1	0	1.639	1.639
11695	2020-3-27 8:00	TT	AMRO	0	61	0	1	1	1.639	2.103
11696	2020-3-28 8:00	TT	AMRO	5	66	1	2	2	3.030	2.457
11697	2020-3-29 8:00	TT	AMRO	8	74	0	2	3	2.703	3.227
11698	2020-3-30 8:00	TT	AMRO	2	76	1	3	4	3.947	3.436
11699	2020-3-31 8:00	TT	AMRO	6	82	0	3	5	3.659	3.712
11700	2020-4-1 8:00	TT	AMRO	3	85	0	3	6	3.529	4.269

Table S1 Daily confirmed cases and daily deaths of COVID-19 in 214 nations from WHO

ID	Date and time	Country or region	Continent	Confirmed patients	Cumulative Confirmed patients	Daily dead patients	Daily cumulative dead patients	Lag days	Daily CFR	3DMA CFR
11701	2020-4-2 8:00	TT	AMRO	4	89	2	5	7	5.618	4.901
11702	2020-4-3 8:00	TT	AMRO	1	90	0	5	8	5.556	5.786
11703	2020-4-4 8:00	TT	AMRO	7	97	1	6	9	6.186	5.914
11704	2020-4-5 8:00	TT	AMRO	3	100	0	6	10	6.000	6.004
11705	2020-4-6 8:00	TT	AMRO	3	103	0	6	11	5.825	6.481
11706	2020-4-7 8:00	TT	AMRO	2	105	2	8	12	7.619	6.997
11707	2020-4-8 8:00	TT	AMRO	1	106	0	8	13	7.547	7.548
11708	2020-4-9 8:00	TT	AMRO	1	107	0	8	14	7.477	7.454
11709	2020-4-10 8:00	TT	AMRO	2	109	0	8	15	7.339	7.385
11710	2020-4-11 8:00	TT	AMRO	0	109	0	8	16	7.339	7.339
11711	2020-4-12 8:00	TT	AMRO	0	109	0	8	17	7.339	7.274
11712	2020-4-13 8:00	TT	AMRO	3	112	0	8	18	7.143	7.187
11713	2020-4-14 8:00	TT	AMRO	1	113	0	8	19	7.080	7.101
11714	2020-4-15 8:00	TT	AMRO	0	113	0	8	20	7.080	7.059
11715	2020-4-16 8:00	TT	AMRO	1	114	0	8	21	7.018	7.038
11716	2020-4-17 8:00	TT	AMRO	0	114	0	8	22	7.018	7.018
11717	2020-4-18 8:00	TT	AMRO	0	114	0	8	23	7.018	7.018
11718	2020-4-19 8:00	TT	AMRO	0	114	0	8	24	7.018	7.018
11719	2020-4-20 8:00	TT	AMRO	0	114	0	8	25	7.018	7.018
11720	2020-4-21 8:00	TT	AMRO	0	114	0	8	26	7.018	7.018
11721	2020-4-22 8:00	TT	AMRO	0	114	0	8	27	7.018	6.997
11722	2020-4-23 8:00	TT	AMRO	1	115	0	8	28	6.957	6.977
11723	2020-4-24 8:00	TT	AMRO	0	115	0	8	29	6.957	6.957
11724	2020-4-25 8:00	TT	AMRO	0	115	0	8	30	6.957	6.957
11725	2020-4-26 8:00	TT	AMRO	0	115	0	8	31	6.957	6.957
11726	2020-4-27 8:00	TT	AMRO	0	115	0	8	32	6.957	6.937
11727	2020-4-28 8:00	TT	AMRO	1	116	0	8	33	6.897	6.917
11728	2020-4-29 8:00	TT	AMRO	0	116	0	8	34	6.897	6.897
11729	2020-4-30 8:00	TT	AMRO	0	116	0	8	35	6.897	6.897
11730	2020-5-1 8:00	TT	AMRO	0	116	0	8	36	6.897	6.897
11731	2020-5-2 8:00	TT	AMRO	0	116	0	8	37	6.897	6.897
11732										
11733	2020-3-2 8:00	TN	EMRO	1	1	0	0			
11734	2020-3-3 8:00	TN	EMRO	0	1	0	0			
11735	2020-3-4 8:00	TN	EMRO	0	1	0	0			
11736	2020-3-5 8:00	TN	EMRO	0	1	0	0			

Table S1 Daily confirmed cases and daily deaths of COVID-19 in 214 nations from WHO

ID	Date and time	Country or region	Continent	Confirmed patients	Cumulative Confirmed patients	Daily dead patients	Daily cumulative dead patients	Lag days	Daily CFR	3DMA CFR
11737	2020-3-6 8:00	TN	EMRO	0	1	0	0			
11738	2020-3-7 8:00	TN	EMRO	0	1	0	0			
11739	2020-3-8 8:00	TN	EMRO	1	2	0	0			
11740	2020-3-9 8:00	TN	EMRO	0	2	0	0			
11741	2020-3-10 8:00	TN	EMRO	4	6	0	0			
11742	2020-3-11 8:00	TN	EMRO	0	6	0	0			
11743	2020-3-12 8:00	TN	EMRO	1	7	0	0			
11744	2020-3-13 8:00	TN	EMRO	0	7	0	0			
11745	2020-3-14 8:00	TN	EMRO	9	16	0	0			
11746	2020-3-15 8:00	TN	EMRO	2	18	0	0			
11747	2020-3-16 8:00	TN	EMRO	2	20	0	0			
11748	2020-3-17 8:00	TN	EMRO	4	24	0	0			
11749	2020-3-18 8:00	TN	EMRO	5	29	0	0			
11750	2020-3-19 8:00	TN	EMRO	10	39	0	0			
11751	2020-3-20 8:00	TN	EMRO	15	54	1	1	0	1.852	1.759
11752	2020-3-21 8:00	TN	EMRO	6	60	0	1	1	1.667	2.506
11753	2020-3-22 8:00	TN	EMRO	15	75	2	3	2	4.000	3.012
11754	2020-3-23 8:00	TN	EMRO	14	89	0	3	3	3.371	3.334
11755	2020-3-24 8:00	TN	EMRO	25	114	0	3	4	2.632	2.964
11756	2020-3-25 8:00	TN	EMRO	59	173	2	5	5	2.890	2.804
11757	2020-3-26 8:00	TN	EMRO	0	173	0	5	6	2.890	2.808
11758	2020-3-27 8:00	TN	EMRO	54	227	1	6	7	2.643	2.726
11759	2020-3-28 8:00	TN	EMRO	0	227	0	6	8	2.643	2.721
11760	2020-3-29 8:00	TN	EMRO	51	278	2	8	9	2.878	2.695
11761	2020-3-30 8:00	TN	EMRO	34	312	0	8	10	2.564	2.643
11762	2020-3-31 8:00	TN	EMRO	50	362	1	9	11	2.486	2.529
11763	2020-4-1 8:00	TN	EMRO	32	394	1	10	12	2.538	2.627
11764	2020-4-2 8:00	TN	EMRO	61	455	3	13	13	2.857	3.011
11765	2020-4-3 8:00	TN	EMRO	40	495	5	18	14	3.636	3.377
11766	2020-4-4 8:00	TN	EMRO	0	495	0	18	15	3.636	3.570
11767	2020-4-5 8:00	TN	EMRO	58	553	1	19	16	3.436	3.635
11768	2020-4-6 8:00	TN	EMRO	21	574	3	22	17	3.833	3.653
11769	2020-4-7 8:00	TN	EMRO	22	596	0	22	18	3.691	3.739
11770	2020-4-8 8:00	TN	EMRO	27	623	1	23	19	3.692	3.735
11771	2020-4-9 8:00	TN	EMRO	5	628	1	24	20	3.822	3.800
11772	2020-4-10 8:00	TN	EMRO	15	643	1	25	21	3.888	3.812

Table S1 Daily confirmed cases and daily deaths of COVID-19 in 214 nations from WHO

ID	Date and time	Country or region	Continent	Confirmed patients	Cumulative Confirmed patients	Daily dead patients	Daily cumulative dead patients	Lag days	Daily CFR	3DMA CFR
11773	2020-4-11 8:00	TN	EMRO	28	671	0	25	22	3.726	3.900
11774	2020-4-12 8:00	TN	EMRO	14	685	3	28	23	4.088	4.066
11775	2020-4-13 8:00	TN	EMRO	22	707	3	31	24	4.385	4.385
11776	2020-4-14 8:00	TN	EMRO	19	726	3	34	25	4.683	4.540
11777	2020-4-15 8:00	TN	EMRO	21	747	0	34	26	4.552	4.574
11778	2020-4-16 8:00	TN	EMRO	33	780	1	35	27	4.487	4.513
11779	2020-4-17 8:00	TN	EMRO	42	822	2	37	28	4.501	4.424
11780	2020-4-18 8:00	TN	EMRO	42	864	0	37	29	4.282	4.352
11781	2020-4-19 8:00	TN	EMRO	2	866	0	37	30	4.273	4.293
11782	2020-4-20 8:00	TN	EMRO	13	879	1	38	31	4.323	4.298
11783	2020-4-21 8:00	TN	EMRO	5	884	0	38	32	4.299	4.280
11784	2020-4-22 8:00	TN	EMRO	17	901	0	38	33	4.218	4.232
11785	2020-4-23 8:00	TN	EMRO	8	909	0	38	34	4.180	4.179
11786	2020-4-24 8:00	TN	EMRO	9	918	0	38	35	4.139	4.147
11787	2020-4-25 8:00	TN	EMRO	4	922	0	38	36	4.121	4.103
11788	2020-4-26 8:00	TN	EMRO	17	939	0	38	37	4.047	4.058
11789	2020-4-27 8:00	TN	EMRO	10	949	0	38	38	4.004	4.028
11790	2020-4-28 8:00	TN	EMRO	18	967	1	39	39	4.033	4.047
11791	2020-4-29 8:00	TN	EMRO	8	975	1	40	40	4.103	4.072
11792	2020-4-30 8:00	TN	EMRO	5	980	0	40	41	4.082	4.103
11793	2020-5-1 8:00	TN	EMRO	14	994	1	41	42	4.125	4.105
11794	2020-5-2 8:00	TN	EMRO	4	998	0	41	43	4.108	4.116
11795										
11796	2020-3-11 8:00	TR	EURO	1	1	0	0			
11797	2020-3-12 8:00	TR	EURO	0	1	0	0			
11798	2020-3-13 8:00	TR	EURO	4	5	0	0			
11799	2020-3-14 8:00	TR	EURO	0	5	0	0			
11800	2020-3-15 8:00	TR	EURO	0	5	0	0			
11801	2020-3-16 8:00	TR	EURO	42	47	0	0			
11802	2020-3-17 8:00	TR	EURO	0	47	0	0			
11803	2020-3-18 8:00	TR	EURO	144	191	2	2	0	1.047	1.047
11804	2020-3-19 8:00	TR	EURO	0	191	0	2	1	1.047	1.146
11805	2020-3-20 8:00	TR	EURO	479	670	7	9	2	1.343	1.536
11806	2020-3-21 8:00	TR	EURO	277	947	12	21	3	2.218	1.926
11807	2020-3-22 8:00	TR	EURO	0	947	0	21	4	2.218	2.287
11808	2020-3-23 8:00	TR	EURO	289	1236	9	30	5	2.427	2.355

Table S1 Daily confirmed cases and daily deaths of COVID-19 in 214 nations from WHO

ID	Date and time	Country or region	Continent	Confirmed patients	Cumulative Confirmed patients	Daily dead patients	Daily cumulative dead patients	Lag days	Daily CFR	3DMA CFR
11809	2020-3-24 8:00	TR	EURO	293	1529	7	37	6	2.420	2.399
11810	2020-3-25 8:00	TR	EURO	343	1872	7	44	7	2.350	2.398
11811	2020-3-26 8:00	TR	EURO	561	2433	15	59	8	2.425	2.281
11812	2020-3-27 8:00	TR	EURO	1196	3629	16	75	9	2.067	2.035
11813	2020-3-28 8:00	TR	EURO	2069	5698	17	92	10	1.615	1.713
11814	2020-3-29 8:00	TR	EURO	1704	7402	16	108	11	1.459	1.496
11815	2020-3-30 8:00	TR	EURO	1869	9271	23	131	12	1.413	1.475
11816	2020-3-31 8:00	TR	EURO	1556	10827	37	168	13	1.552	1.515
11817	2020-4-1 8:00	TR	EURO	2704	13531	46	214	14	1.582	1.633
11818	2020-4-2 8:00	TR	EURO	2148	15679	63	277	15	1.767	1.770
11819	2020-4-3 8:00	TR	EURO	2456	18135	79	356	16	1.963	1.920
11820	2020-4-4 8:00	TR	EURO	2786	20921	69	425	17	2.031	2.029
11821	2020-4-5 8:00	TR	EURO	3013	23934	76	501	18	2.093	2.082
11822	2020-4-6 8:00	TR	EURO	3135	27069	73	574	19	2.121	2.121
11823	2020-4-7 8:00	TR	EURO	3148	30217	75	649	20	2.148	2.131
11824	2020-4-8 8:00	TR	EURO	3892	34109	76	725	21	2.126	2.133
11825	2020-4-9 8:00	TR	EURO	4117	38226	87	812	22	2.124	2.132
11826	2020-4-10 8:00	TR	EURO	4056	42282	96	908	23	2.147	2.137
11827	2020-4-11 8:00	TR	EURO	4747	47029	98	1006	24	2.139	2.132
11828	2020-4-12 8:00	TR	EURO	5138	52167	95	1101	25	2.111	2.118
11829	2020-4-13 8:00	TR	EURO	4789	56956	97	1198	26	2.103	2.112
11830	2020-4-14 8:00	TR	EURO	4093	61049	98	1296	27	2.123	2.127
11831	2020-4-15 8:00	TR	EURO	4062	65111	107	1403	28	2.155	2.155
11832	2020-4-16 8:00	TR	EURO	4281	69392	115	1518	29	2.188	2.186
11833	2020-4-17 8:00	TR	EURO	4801	74193	125	1643	30	2.214	2.218
11834	2020-4-18 8:00	TR	EURO	4353	78546	126	1769	31	2.252	2.254
11835	2020-4-19 8:00	TR	EURO	3783	82329	121	1890	32	2.296	2.295
11836	2020-4-20 8:00	TR	EURO	3977	86306	127	2017	33	2.337	2.328
11837	2020-4-21 8:00	TR	EURO	4674	90980	123	2140	34	2.352	2.351
11838	2020-4-22 8:00	TR	EURO	4611	95591	119	2259	35	2.363	2.374
11839	2020-4-23 8:00	TR	EURO	3083	98674	117	2376	36	2.408	2.406
11840	2020-4-24 8:00	TR	EURO	3116	101790	115	2491	37	2.447	2.444
11841	2020-4-25 8:00	TR	EURO	3122	104912	109	2600	38	2.478	2.479
11842	2020-4-26 8:00	TR	EURO	2861	107773	106	2706	39	2.511	2.512
11843	2020-4-27 8:00	TR	EURO	2357	110130	99	2805	40	2.547	2.547
11844	2020-4-28 8:00	TR	EURO	2131	112261	95	2900	41	2.583	2.580

Table S1 Daily confirmed cases and daily deaths of COVID-19 in 214 nations from WHO

ID	Date and time	Country or region	Continent	Confirmed patients	Cumulative Confirmed patients	Daily dead patients	Daily cumulative dead patients	Lag days	Daily CFR	3DMA CFR
11845	2020-4-29 8:00	TR	EURO	2392	114653	92	2992	42	2.610	2.604
11846	2020-4-30 8:00	TR	EURO	2936	117589	89	3081	43	2.620	2.623
11847	2020-5-1 8:00	TR	EURO	2615	120204	93	3174	44	2.641	2.641
11848	2020-5-2 8:00	TR	EURO	2188	122392	84	3258	45	2.662	2.651
11849										
11850	2020-3-24 8:00	TC	AMRO	1	1	0	0			
11851	2020-3-25 8:00	TC	AMRO	0	1	0	0			
11852	2020-3-26 8:00	TC	AMRO	1	2	0	0			
11853	2020-3-27 8:00	TC	AMRO	0	2	0	0			
11854	2020-3-28 8:00	TC	AMRO	0	2	0	0			
11855	2020-3-29 8:00	TC	AMRO	0	2	0	0			
11856	2020-3-30 8:00	TC	AMRO	2	4	0	0			
11857	2020-3-31 8:00	TC	AMRO	1	5	0	0			
11858	2020-4-1 8:00	TC	AMRO	0	5	0	0			
11859	2020-4-2 8:00	TC	AMRO	0	5	0	0			
11860	2020-4-3 8:00	TC	AMRO	0	5	0	0			
11861	2020-4-4 8:00	TC	AMRO	0	5	0	0			
11862	2020-4-5 8:00	TC	AMRO	0	5	0	0			
11863	2020-4-6 8:00	TC	AMRO	0	5	1	1	0	20.000	20.000
11864	2020-4-7 8:00	TC	AMRO	0	5	0	1	1	20.000	17.500
11865	2020-4-8 8:00	TC	AMRO	3	8	0	1	2	12.500	15.000
11866	2020-4-9 8:00	TC	AMRO	0	8	0	1	3	12.500	12.500
11867	2020-4-10 8:00	TC	AMRO	0	8	0	1	4	12.500	12.500
11868	2020-4-11 8:00	TC	AMRO	0	8	0	1	5	12.500	12.500
11869	2020-4-12 8:00	TC	AMRO	0	8	0	1	6	12.500	12.037
11870	2020-4-13 8:00	TC	AMRO	1	9	0	1	7	11.111	11.574
11871	2020-4-14 8:00	TC	AMRO	0	9	0	1	8	11.111	10.741
11872	2020-4-15 8:00	TC	AMRO	1	10	0	1	9	10.000	10.370
11873	2020-4-16 8:00	TC	AMRO	0	10	0	1	10	10.000	9.697
11874	2020-4-17 8:00	TC	AMRO	1	11	0	1	11	9.091	9.394
11875	2020-4-18 8:00	TC	AMRO	0	11	0	1	12	9.091	9.091
11876	2020-4-19 8:00	TC	AMRO	0	11	0	1	13	9.091	9.091
11877	2020-4-20 8:00	TC	AMRO	0	11	0	1	14	9.091	9.091
11878	2020-4-21 8:00	TC	AMRO	0	11	0	1	15	9.091	9.091
11879	2020-4-22 8:00	TC	AMRO	0	11	0	1	16	9.091	9.091
11880	2020-4-23 8:00	TC	AMRO	0	11	0	1	17	9.091	9.091

Table S1 Daily confirmed cases and daily deaths of COVID-19 in 214 nations from WHO

ID	Date and time	Country or region	Continent	Confirmed patients	Cumulative Confirmed patients	Daily dead patients	Daily cumulative dead patients	Lag days	Daily CFR	3DMA CFR
11881	2020-4-24 8:00	TC	AMRO	0	11	0	1	18	9.091	9.091
11882	2020-4-25 8:00	TC	AMRO	0	11	0	1	19	9.091	9.091
11883	2020-4-26 8:00	TC	AMRO	0	11	0	1	20	9.091	9.091
11884	2020-4-27 8:00	TC	AMRO	0	11	0	1	21	9.091	8.838
11885	2020-4-28 8:00	TC	AMRO	1	12	0	1	22	8.333	8.586
11886	2020-4-29 8:00	TC	AMRO	0	12	0	1	23	8.333	8.333
11887	2020-4-30 8:00	TC	AMRO	0	12	0	1	24	8.333	8.333
11888	2020-5-1 8:00	TC	AMRO	0	12	0	1	25	8.333	8.333
11889	2020-5-2 8:00	TC	AMRO	0	12	0	1	26	8.333	8.333
11890										
11891	2020-3-21 8:00	UG	AFRO	1	1	0	0			
11892	2020-3-22 8:00	UG	AFRO	0	1	0	0			
11893	2020-3-23 8:00	UG	AFRO	0	1	0	0			
11894	2020-3-24 8:00	UG	AFRO	8	9	0	0			
11895	2020-3-25 8:00	UG	AFRO	0	9	0	0			
11896	2020-3-26 8:00	UG	AFRO	5	14	0	0			
11897	2020-3-27 8:00	UG	AFRO	0	14	0	0			
11898	2020-3-28 8:00	UG	AFRO	0	14	0	0			
11899	2020-3-29 8:00	UG	AFRO	16	30	0	0			
11900	2020-3-30 8:00	UG	AFRO	3	33	0	0			
11901	2020-3-31 8:00	UG	AFRO	0	33	0	0			
11902	2020-4-1 8:00	UG	AFRO	0	33	0	0			
11903	2020-4-2 8:00	UG	AFRO	11	44	0	0			
11904	2020-4-3 8:00	UG	AFRO	1	45	0	0			
11905	2020-4-4 8:00	UG	AFRO	3	48	0	0			
11906	2020-4-5 8:00	UG	AFRO	0	48	0	0			
11907	2020-4-6 8:00	UG	AFRO	0	48	0	0			
11908	2020-4-7 8:00	UG	AFRO	4	52	0	0			
11909	2020-4-8 8:00	UG	AFRO	0	52	0	0			
11910	2020-4-9 8:00	UG	AFRO	1	53	0	0			
11911	2020-4-10 8:00	UG	AFRO	0	53	0	0			
11912	2020-4-11 8:00	UG	AFRO	0	53	0	0			
11913	2020-4-12 8:00	UG	AFRO	0	53	0	0			
11914	2020-4-13 8:00	UG	AFRO	1	54	0	0			
11915	2020-4-14 8:00	UG	AFRO	0	54	0	0			
11916	2020-4-15 8:00	UG	AFRO	0	54	0	0			

Table S1 Daily confirmed cases and daily deaths of COVID-19 in 214 nations from WHO

ID	Date and time	Country or region	Continent	Confirmed patients	Cumulative Confirmed patients	Daily dead patients	Daily cumulative dead patients	Lag days	Daily CFR	3DMA CFR
11917	2020-4-16 8:00	UG	AFRO	1	55	0	0			
11918	2020-4-17 8:00	UG	AFRO	0	55	0	0			
11919	2020-4-18 8:00	UG	AFRO	0	55	0	0			
11920	2020-4-19 8:00	UG	AFRO	0	55	0	0			
11921	2020-4-20 8:00	UG	AFRO	0	55	0	0			
11922	2020-4-21 8:00	UG	AFRO	0	55	0	0			
11923	2020-4-22 8:00	UG	AFRO	1	56	0	0			
11924	2020-4-23 8:00	UG	AFRO	5	61	0	0			
11925	2020-4-24 8:00	UG	AFRO	2	63	0	0			
11926	2020-4-25 8:00	UG	AFRO	12	75	0	0			
11927	2020-4-26 8:00	UG	AFRO	0	75	0	0			
11928	2020-4-27 8:00	UG	AFRO	0	75	0	0			
11929	2020-4-28 8:00	UG	AFRO	4	79	0	0			
11930	2020-4-29 8:00	UG	AFRO	0	79	0	0			
11931	2020-4-30 8:00	UG	AFRO	0	79	0	0			
11932	2020-5-1 8:00	UG	AFRO	2	81	0	0			
11933	2020-5-2 8:00	UG	AFRO	2	83	0	0			
11934										
11935	2020-3-3 8:00	UA	EURO	1	1	0	0			
11936	2020-3-4 8:00	UA	EURO	0	1	0	0			
11937	2020-3-5 8:00	UA	EURO	0	1	0	0			
11938	2020-3-6 8:00	UA	EURO	0	1	0	0			
11939	2020-3-7 8:00	UA	EURO	0	1	0	0			
11940	2020-3-8 8:00	UA	EURO	0	1	0	0			
11941	2020-3-9 8:00	UA	EURO	0	1	0	0			
11942	2020-3-10 8:00	UA	EURO	0	1	0	0			
11943	2020-3-11 8:00	UA	EURO	0	1	0	0			
11944	2020-3-12 8:00	UA	EURO	0	1	0	0			
11945	2020-3-13 8:00	UA	EURO	2	3	1	1	0	33.333	33.333
11946	2020-3-14 8:00	UA	EURO	0	3	0	1	1	33.333	33.333
11947	2020-3-15 8:00	UA	EURO	0	3	0	1	2	33.333	28.889
11948	2020-3-16 8:00	UA	EURO	2	5	0	1	3	20.000	22.540
11949	2020-3-17 8:00	UA	EURO	9	14	1	2	4	14.286	15.595
11950	2020-3-18 8:00	UA	EURO	2	16	0	2	5	12.500	13.095
11951	2020-3-19 8:00	UA	EURO	0	16	0	2	6	12.500	12.179
11952	2020-3-20 8:00	UA	EURO	10	26	1	3	7	11.538	10.140

Table S1 Daily confirmed cases and daily deaths of COVID-19 in 214 nations from WHO

ID	Date and time	Country or region	Continent	Confirmed patients	Cumulative Confirmed patients	Daily dead patients	Daily cumulative dead patients	Lag days	Daily CFR	3DMA CFR
11953	2020-3-21 8:00	UA	EURO	21	47	0	3	8	6.383	8.101
11954	2020-3-22 8:00	UA	EURO	0	47	0	3	9	6.383	6.383
11955	2020-3-23 8:00	UA	EURO	0	47	0	3	10	6.383	5.446
11956	2020-3-24 8:00	UA	EURO	37	84	0	3	11	3.571	4.498
11957	2020-3-25 8:00	UA	EURO	29	113	1	4	12	3.540	3.439
11958	2020-3-26 8:00	UA	EURO	43	156	1	5	13	3.205	3.013
11959	2020-3-27 8:00	UA	EURO	62	218	0	5	14	2.294	2.690
11960	2020-3-28 8:00	UA	EURO	93	311	3	8	15	2.572	2.340
11961	2020-3-29 8:00	UA	EURO	107	418	1	9	16	2.153	2.339
11962	2020-3-30 8:00	UA	EURO	62	480	2	11	17	2.292	2.271
11963	2020-3-31 8:00	UA	EURO	69	549	2	13	18	2.368	2.400
11964	2020-4-1 8:00	UA	EURO	120	669	4	17	19	2.541	2.466
11965	2020-4-2 8:00	UA	EURO	135	804	3	20	20	2.488	2.453
11966	2020-4-3 8:00	UA	EURO	183	987	3	23	21	2.330	2.458
11967	2020-4-4 8:00	UA	EURO	109	1096	5	28	22	2.555	2.481
11968	2020-4-5 8:00	UA	EURO	155	1251	4	32	23	2.558	2.665
11969	2020-4-6 8:00	UA	EURO	68	1319	6	38	24	2.881	2.839
11970	2020-4-7 8:00	UA	EURO	143	1462	7	45	25	3.078	3.025
11971	2020-4-8 8:00	UA	EURO	206	1668	7	52	26	3.118	3.069
11972	2020-4-9 8:00	UA	EURO	224	1892	5	57	27	3.013	3.087
11973	2020-4-10 8:00	UA	EURO	311	2203	12	69	28	3.132	3.017
11974	2020-4-11 8:00	UA	EURO	308	2511	4	73	29	2.907	3.009
11975	2020-4-12 8:00	UA	EURO	266	2777	10	83	30	2.989	2.965
11976	2020-4-13 8:00	UA	EURO	325	3102	10	93	31	2.998	2.964
11977	2020-4-14 8:00	UA	EURO	270	3372	5	98	32	2.906	2.925
11978	2020-4-15 8:00	UA	EURO	392	3764	10	108	33	2.869	2.846
11979	2020-4-16 8:00	UA	EURO	398	4162	7	115	34	2.763	2.771
11980	2020-4-17 8:00	UA	EURO	500	4662	10	125	35	2.681	2.683
11981	2020-4-18 8:00	UA	EURO	444	5106	8	133	36	2.605	2.625
11982	2020-4-19 8:00	UA	EURO	343	5449	8	141	37	2.588	2.612
11983	2020-4-20 8:00	UA	EURO	261	5710	10	151	38	2.644	2.620
11984	2020-4-21 8:00	UA	EURO	415	6125	10	161	39	2.629	2.638
11985	2020-4-22 8:00	UA	EURO	467	6592	13	174	40	2.640	2.625
11986	2020-4-23 8:00	UA	EURO	578	7170	13	187	41	2.608	2.591
11987	2020-4-24 8:00	UA	EURO	477	7647	6	193	42	2.524	2.535
11988	2020-4-25 8:00	UA	EURO	478	8125	8	201	43	2.474	2.474

Table S1 Daily confirmed cases and daily deaths of COVID-19 in 214 nations from WHO

ID	Date and time	Country or region	Continent	Confirmed patients	Cumulative Confirmed patients	Daily dead patients	Daily cumulative dead patients	Lag days	Daily CFR	3DMA CFR
11989	2020-4-26 8:00	UA	EURO	492	8617	8	209	44	2.425	2.447
11990	2020-4-27 8:00	UA	EURO	392	9009	11	220	45	2.442	2.469
11991	2020-4-28 8:00	UA	EURO	401	9410	19	239	46	2.540	2.505
11992	2020-4-29 8:00	UA	EURO	456	9866	11	250	47	2.534	2.527
11993	2020-4-30 8:00	UA	EURO	540	10406	11	261	48	2.508	2.515
11994	2020-5-1 8:00	UA	EURO	455	10861	11	272	49	2.504	2.486
11995	2020-5-2 8:00	UA	EURO	550	11411	7	279	50	2.445	2.475
11996										
11997	2020-1-29 8:00	AE	EMRO	4	4	0	0			
11998	2020-1-30 8:00	AE	EMRO	0	4	0	0			
11999	2020-1-31 8:00	AE	EMRO	0	4	0	0			
12000	2020-2-1 8:00	AE	EMRO	1	5	0	0			
12001	2020-2-2 8:00	AE	EMRO	0	5	0	0			
12002	2020-2-3 8:00	AE	EMRO	0	5	0	0			
12003	2020-2-4 8:00	AE	EMRO	0	5	0	0			
12004	2020-2-5 8:00	AE	EMRO	0	5	0	0			
12005	2020-2-6 8:00	AE	EMRO	0	5	0	0			
12006	2020-2-7 8:00	AE	EMRO	0	5	0	0			
12007	2020-2-8 8:00	AE	EMRO	2	7	0	0			
12008	2020-2-9 8:00	AE	EMRO	0	7	0	0			
12009	2020-2-10 8:00	AE	EMRO	1	8	0	0			
12010	2020-2-11 8:00	AE	EMRO	0	8	0	0			
12011	2020-2-12 8:00	AE	EMRO	0	8	0	0			
12012	2020-2-13 8:00	AE	EMRO	0	8	0	0			
12013	2020-2-14 8:00	AE	EMRO	0	8	0	0			
12014	2020-2-15 8:00	AE	EMRO	0	8	0	0			
12015	2020-2-16 8:00	AE	EMRO	1	9	0	0			
12016	2020-2-17 8:00	AE	EMRO	0	9	0	0			
12017	2020-2-18 8:00	AE	EMRO	0	9	0	0			
12018	2020-2-19 8:00	AE	EMRO	0	9	0	0			
12019	2020-2-20 8:00	AE	EMRO	0	9	0	0			
12020	2020-2-21 8:00	AE	EMRO	2	11	0	0			
12021	2020-2-22 8:00	AE	EMRO	2	13	0	0			
12022	2020-2-23 8:00	AE	EMRO	0	13	0	0			
12023	2020-2-24 8:00	AE	EMRO	0	13	0	0			
12024	2020-2-25 8:00	AE	EMRO	0	13	0	0			

Table S1 Daily confirmed cases and daily deaths of COVID-19 in 214 nations from WHO

ID	Date and time	Country or region	Continent	Confirmed patients	Cumulative Confirmed patients	Daily dead patients	Daily cumulative dead patients	Lag days	Daily CFR	3DMA CFR
12025	2020-2-26 8:00	AE	EMRO	0	13	0	0			
12026	2020-2-27 8:00	AE	EMRO	6	19	0	0			
12027	2020-2-28 8:00	AE	EMRO	0	19	0	0			
12028	2020-2-29 8:00	AE	EMRO	0	19	0	0			
12029	2020-3-1 8:00	AE	EMRO	2	21	0	0			
12030	2020-3-2 8:00	AE	EMRO	0	21	0	0			
12031	2020-3-3 8:00	AE	EMRO	6	27	0	0			
12032	2020-3-4 8:00	AE	EMRO	0	27	0	0			
12033	2020-3-5 8:00	AE	EMRO	0	27	0	0			
12034	2020-3-6 8:00	AE	EMRO	18	45	0	0			
12035	2020-3-7 8:00	AE	EMRO	0	45	0	0			
12036	2020-3-8 8:00	AE	EMRO	0	45	0	0			
12037	2020-3-9 8:00	AE	EMRO	14	59	0	0			
12038	2020-3-10 8:00	AE	EMRO	15	74	0	0			
12039	2020-3-11 8:00	AE	EMRO	0	74	0	0			
12040	2020-3-12 8:00	AE	EMRO	11	85	0	0			
12041	2020-3-13 8:00	AE	EMRO	0	85	0	0			
12042	2020-3-14 8:00	AE	EMRO	0	85	0	0			
12043	2020-3-15 8:00	AE	EMRO	13	98	0	0			
12044	2020-3-16 8:00	AE	EMRO	0	98	0	0			
12045	2020-3-17 8:00	AE	EMRO	0	98	0	0			
12046	2020-3-18 8:00	AE	EMRO	15	113	0	0			
12047	2020-3-19 8:00	AE	EMRO	27	140	0	0			
12048	2020-3-20 8:00	AE	EMRO	0	140	0	0			
12049	2020-3-21 8:00	AE	EMRO	13	153	2	2	0	1.307	1.307
12050	2020-3-22 8:00	AE	EMRO	0	153	0	2	1	1.307	1.208
12051	2020-3-23 8:00	AE	EMRO	45	198	0	2	2	1.010	1.041
12052	2020-3-24 8:00	AE	EMRO	50	248	0	2	3	0.806	0.806
12053	2020-3-25 8:00	AE	EMRO	85	333	0	2	4	0.601	0.669
12054	2020-3-26 8:00	AE	EMRO	0	333	0	2	5	0.601	0.601
12055	2020-3-27 8:00	AE	EMRO	0	333	0	2	6	0.601	0.543
12056	2020-3-28 8:00	AE	EMRO	135	468	0	2	7	0.427	0.485
12057	2020-3-29 8:00	AE	EMRO	0	468	0	2	8	0.427	0.558
12058	2020-3-30 8:00	AE	EMRO	143	611	3	5	9	0.818	0.716
12059	2020-3-31 8:00	AE	EMRO	53	664	1	6	10	0.904	0.875
12060	2020-4-1 8:00	AE	EMRO	0	664	0	6	11	0.904	0.930

Table S1 Daily confirmed cases and daily deaths of COVID-19 in 214 nations from WHO

ID	Date and time	Country or region	Continent	Confirmed patients	Cumulative Confirmed patients	Daily dead patients	Daily cumulative dead patients	Lag days	Daily CFR	3DMA CFR
12061	2020-4-2 8:00	AE	EMRO	150	814	2	8	12	0.983	0.889
12062	2020-4-3 8:00	AE	EMRO	210	1024	0	8	13	0.781	0.810
12063	2020-4-4 8:00	AE	EMRO	481	1505	2	10	14	0.664	0.703
12064	2020-4-5 8:00	AE	EMRO	0	1505	0	10	15	0.664	0.620
12065	2020-4-6 8:00	AE	EMRO	571	2076	1	11	16	0.530	0.575
12066	2020-4-7 8:00	AE	EMRO	0	2076	0	11	17	0.530	0.504
12067	2020-4-8 8:00	AE	EMRO	583	2659	1	12	18	0.451	0.477
12068	2020-4-9 8:00	AE	EMRO	0	2659	0	12	19	0.451	0.457
12069	2020-4-10 8:00	AE	EMRO	331	2990	2	14	20	0.468	0.485
12070	2020-4-11 8:00	AE	EMRO	746	3736	6	20	21	0.535	0.513
12071	2020-4-12 8:00	AE	EMRO	0	3736	0	20	22	0.535	0.559
12072	2020-4-13 8:00	AE	EMRO	387	4123	5	25	23	0.606	0.565
12073	2020-4-14 8:00	AE	EMRO	398	4521	0	25	24	0.553	0.576
12074	2020-4-15 8:00	AE	EMRO	412	4933	3	28	25	0.568	0.579
12075	2020-4-16 8:00	AE	EMRO	432	5365	5	33	26	0.615	0.595
12076	2020-4-17 8:00	AE	EMRO	460	5825	2	35	27	0.601	0.601
12077	2020-4-18 8:00	AE	EMRO	477	6302	2	37	28	0.587	0.598
12078	2020-4-19 8:00	AE	EMRO	479	6781	4	41	29	0.605	0.595
12079	2020-4-20 8:00	AE	EMRO	484	7265	2	43	30	0.592	0.597
12080	2020-4-21 8:00	AE	EMRO	490	7755	3	46	31	0.593	0.605
12081	2020-4-22 8:00	AE	EMRO	483	8238	6	52	32	0.631	0.621
12082	2020-4-23 8:00	AE	EMRO	518	8756	4	56	33	0.640	0.653
12083	2020-4-24 8:00	AE	EMRO	525	9281	8	64	34	0.690	0.673
12084	2020-4-25 8:00	AE	EMRO	0	9281	0	64	35	0.690	0.705
12085	2020-4-26 8:00	AE	EMRO	1068	10349	12	76	36	0.734	0.719
12086	2020-4-27 8:00	AE	EMRO	0	10349	0	76	37	0.734	0.750
12087	2020-4-28 8:00	AE	EMRO	1031	11380	13	89	38	0.782	0.766
12088	2020-4-29 8:00	AE	EMRO	0	11380	0	89	39	0.782	0.802
12089	2020-4-30 8:00	AE	EMRO	1101	12481	16	105	40	0.841	0.825
12090	2020-5-1 8:00	AE	EMRO	557	13038	6	111	41	0.851	0.848
12091	2020-5-2 8:00	AE	EMRO	0	13038	0	111	42	0.851	0.851
12092										
12093	2020-3-17 8:00	TZ	AFRO	1	1	0	0			
12094	2020-3-18 8:00	TZ	AFRO	2	3	0	0			
12095	2020-3-19 8:00	TZ	AFRO	3	6	0	0			
12096	2020-3-20 8:00	TZ	AFRO	0	6	0	0			

Table S1 Daily confirmed cases and daily deaths of COVID-19 in 214 nations from WHO

ID	Date and time	Country or region	Continent	Confirmed patients	Cumulative Confirmed patients	Daily dead patients	Daily cumulative dead patients	Lag days	Daily CFR	3DMA CFR
12097	2020-3-21 8:00	TZ	AFRO	0	6	0	0			
12098	2020-3-22 8:00	TZ	AFRO	0	6	0	0			
12099	2020-3-23 8:00	TZ	AFRO	6	12	0	0			
12100	2020-3-24 8:00	TZ	AFRO	0	12	0	0			
12101	2020-3-25 8:00	TZ	AFRO	0	12	0	0			
12102	2020-3-26 8:00	TZ	AFRO	1	13	0	0			
12103	2020-3-27 8:00	TZ	AFRO	0	13	0	0			
12104	2020-3-28 8:00	TZ	AFRO	0	13	0	0			
12105	2020-3-29 8:00	TZ	AFRO	1	14	0	0			
12106	2020-3-30 8:00	TZ	AFRO	5	19	0	0			
12107	2020-3-31 8:00	TZ	AFRO	0	19	0	0			
12108	2020-4-1 8:00	TZ	AFRO	1	20	1	1	0	5.000	5.000
12109	2020-4-2 8:00	TZ	AFRO	0	20	0	1	1	5.000	5.000
12110	2020-4-3 8:00	TZ	AFRO	0	20	0	1	2	5.000	5.000
12111	2020-4-4 8:00	TZ	AFRO	0	20	0	1	3	5.000	5.000
12112	2020-4-5 8:00	TZ	AFRO	0	20	0	1	4	5.000	4.848
12113	2020-4-6 8:00	TZ	AFRO	2	22	0	1	5	4.545	4.571
12114	2020-4-7 8:00	TZ	AFRO	2	24	0	1	6	4.167	4.293
12115	2020-4-8 8:00	TZ	AFRO	0	24	0	1	7	4.167	4.111
12116	2020-4-9 8:00	TZ	AFRO	1	25	0	1	8	4.000	4.056
12117	2020-4-10 8:00	TZ	AFRO	0	25	0	1	9	4.000	5.792
12118	2020-4-11 8:00	TZ	AFRO	7	32	2	3	10	9.375	7.583
12119	2020-4-12 8:00	TZ	AFRO	0	32	0	3	11	9.375	9.375
12120	2020-4-13 8:00	TZ	AFRO	0	32	0	3	12	9.375	8.291
12121	2020-4-14 8:00	TZ	AFRO	17	49	0	3	13	6.122	7.053
12122	2020-4-15 8:00	TZ	AFRO	4	53	0	3	14	5.660	5.443
12123	2020-4-16 8:00	TZ	AFRO	35	88	1	4	15	4.545	4.820
12124	2020-4-17 8:00	TZ	AFRO	6	94	0	4	16	4.255	4.060
12125	2020-4-18 8:00	TZ	AFRO	54	148	1	5	17	3.378	3.671
12126	2020-4-19 8:00	TZ	AFRO	0	148	0	5	18	3.378	3.617
12127	2020-4-20 8:00	TZ	AFRO	23	171	2	7	19	4.094	3.798
12128	2020-4-21 8:00	TZ	AFRO	84	255	3	10	20	3.922	3.979
12129	2020-4-22 8:00	TZ	AFRO	0	255	0	10	21	3.922	3.784
12130	2020-4-23 8:00	TZ	AFRO	30	285	0	10	22	3.509	3.646
12131	2020-4-24 8:00	TZ	AFRO	0	285	0	10	23	3.509	3.450
12132	2020-4-25 8:00	TZ	AFRO	15	300	0	10	24	3.333	3.392

Table S1 Daily confirmed cases and daily deaths of COVID-19 in 214 nations from WHO

ID	Date and time	Country or region	Continent	Confirmed patients	Cumulative Confirmed patients	Daily dead patients	Daily cumulative dead patients	Lag days	Daily CFR	3DMA CFR
12133	2020-4-26 8:00	TZ	AFRO	0	300	0	10	25	3.333	3.333
12134	2020-4-27 8:00	TZ	AFRO	0	300	0	10	26	3.333	3.333
12135	2020-4-28 8:00	TZ	AFRO	0	300	0	10	27	3.333	3.333
12136	2020-4-29 8:00	TZ	AFRO	0	300	0	10	28	3.333	3.333
12137	2020-4-30 8:00	TZ	AFRO	180	480	6	16	29	3.333	3.403
12138	2020-5-1 8:00	TZ	AFRO	0	480	1	17	30	3.542	3.472
12139	2020-5-2 8:00	TZ	AFRO	0	480	0	17	31	3.542	3.542
12140										
12141	2020-1-20 8:00	US	AMRO	1	1	0	0			
12142	2020-1-21 8:00	US	AMRO	0	1	0	0			
12143	2020-1-22 8:00	US	AMRO	0	1	0	0			
12144	2020-1-23 8:00	US	AMRO	0	1	0	0			
12145	2020-1-24 8:00	US	AMRO	1	2	0	0			
12146	2020-1-25 8:00	US	AMRO	0	2	0	0			
12147	2020-1-26 8:00	US	AMRO	3	5	0	0			
12148	2020-1-27 8:00	US	AMRO	0	5	0	0			
12149	2020-1-28 8:00	US	AMRO	0	5	0	0			
12150	2020-1-29 8:00	US	AMRO	0	5	0	0			
12151	2020-1-30 8:00	US	AMRO	1	6	0	0			
12152	2020-1-31 8:00	US	AMRO	1	7	0	0			
12153	2020-2-1 8:00	US	AMRO	1	8	0	0			
12154	2020-2-2 8:00	US	AMRO	0	8	0	0			
12155	2020-2-3 8:00	US	AMRO	3	11	0	0			
12156	2020-2-4 8:00	US	AMRO	0	11	0	0			
12157	2020-2-5 8:00	US	AMRO	0	11	0	0			
12158	2020-2-6 8:00	US	AMRO	1	12	0	0			
12159	2020-2-7 8:00	US	AMRO	0	12	0	0			
12160	2020-2-8 8:00	US	AMRO	0	12	0	0			
12161	2020-2-9 8:00	US	AMRO	0	12	0	0			
12162	2020-2-10 8:00	US	AMRO	0	12	0	0			
12163	2020-2-11 8:00	US	AMRO	1	13	0	0			
12164	2020-2-12 8:00	US	AMRO	0	13	0	0			
12165	2020-2-13 8:00	US	AMRO	2	15	0	0			
12166	2020-2-14 8:00	US	AMRO	0	15	0	0			
12167	2020-2-15 8:00	US	AMRO	0	15	0	0			
12168	2020-2-16 8:00	US	AMRO	0	15	0	0			

Table S1 Daily confirmed cases and daily deaths of COVID-19 in 214 nations from WHO

ID	Date and time	Country or region	Continent	Confirmed patients	Cumulative Confirmed patients	Daily dead patients	Daily cumulative dead patients	Lag days	Daily CFR	3DMA CFR
12169	2020-2-17 8:00	US	AMRO	0	15	0	0			
12170	2020-2-18 8:00	US	AMRO	0	15	0	0			
12171	2020-2-19 8:00	US	AMRO	0	15	0	0			
12172	2020-2-20 8:00	US	AMRO	0	15	0	0			
12173	2020-2-21 8:00	US	AMRO	19	34	0	0			
12174	2020-2-22 8:00	US	AMRO	1	35	0	0			
12175	2020-2-23 8:00	US	AMRO	0	35	0	0			
12176	2020-2-24 8:00	US	AMRO	18	53	0	0			
12177	2020-2-25 8:00	US	AMRO	0	53	0	0			
12178	2020-2-26 8:00	US	AMRO	6	59	0	0			
12179	2020-2-27 8:00	US	AMRO	0	59	0	0			
12180	2020-2-28 8:00	US	AMRO	3	62	0	0			
12181	2020-2-29 8:00	US	AMRO	0	62	0	0			
12182	2020-3-1 8:00	US	AMRO	0	62	0	0			
12183	2020-3-2 8:00	US	AMRO	2	64	0	0			
12184	2020-3-3 8:00	US	AMRO	44	108	2	2	0	1.852	2.864
12185	2020-3-4 8:00	US	AMRO	21	129	3	5	1	3.876	4.162
12186	2020-3-5 8:00	US	AMRO	19	148	5	10	2	6.757	5.266
12187	2020-3-6 8:00	US	AMRO	65	213	1	11	3	5.164	5.695
12188	2020-3-7 8:00	US	AMRO	0	213	0	11	4	5.164	5.164
12189	2020-3-8 8:00	US	AMRO	0	213	0	11	5	5.164	4.785
12190	2020-3-9 8:00	US	AMRO	259	472	8	19	6	4.025	4.261
12191	2020-3-10 8:00	US	AMRO	224	696	6	25	7	3.592	3.736
12192	2020-3-11 8:00	US	AMRO	0	696	0	25	8	3.592	3.374
12193	2020-3-12 8:00	US	AMRO	291	987	4	29	9	2.938	3.126
12194	2020-3-13 8:00	US	AMRO	277	1264	7	36	10	2.848	2.743
12195	2020-3-14 8:00	US	AMRO	414	1678	5	41	11	2.443	2.561
12196	2020-3-15 8:00	US	AMRO	36	1714	0	41	12	2.392	2.409
12197	2020-3-16 8:00	US	AMRO	0	1714	0	41	13	2.392	2.141
12198	2020-3-17 8:00	US	AMRO	1822	3536	17	58	14	1.640	1.814
12199	2020-3-18 8:00	US	AMRO	3551	7087	42	100	15	1.411	1.496
12200	2020-3-19 8:00	US	AMRO	3355	10442	50	150	16	1.437	1.389
12201	2020-3-20 8:00	US	AMRO	4777	15219	51	201	17	1.321	1.359
12202	2020-3-21 8:00	US	AMRO	0	15219	0	201	18	1.321	1.321
12203	2020-3-22 8:00	US	AMRO	0	15219	0	201	19	1.321	1.305
12204	2020-3-23 8:00	US	AMRO	16354	31573	201	402	20	1.273	1.297

Table S1 Daily confirmed cases and daily deaths of COVID-19 in 214 nations from WHO

ID	Date and time	Country or region	Continent	Confirmed patients	Cumulative Confirmed patients	Daily dead patients	Daily cumulative dead patients	Lag days	Daily CFR	3DMA CFR
12205	2020-3-24 8:00	US	AMRO	20341	51914	271	673	21	1.296	1.289
12206	2020-3-25 8:00	US	AMRO	0	51914	0	673	22	1.296	1.348
12207	2020-3-26 8:00	US	AMRO	16420	68334	318	991	23	1.450	1.399
12208	2020-3-27 8:00	US	AMRO	0	68334	0	991	24	1.450	1.453
12209	2020-3-28 8:00	US	AMRO	16894	85228	252	1243	25	1.458	1.508
12210	2020-3-29 8:00	US	AMRO	18093	103321	425	1668	26	1.614	1.598
12211	2020-3-30 8:00	US	AMRO	19332	122653	444	2112	27	1.722	1.680
12212	2020-3-31 8:00	US	AMRO	17987	140640	286	2398	28	1.705	1.724
12213	2020-4-1 8:00	US	AMRO	22559	163199	452	2850	29	1.746	1.835
12214	2020-4-2 8:00	US	AMRO	24103	187302	996	3846	30	2.053	2.015
12215	2020-4-3 8:00	US	AMRO	26298	213600	947	4793	31	2.244	2.240
12216	2020-4-4 8:00	US	AMRO	28103	241703	1061	5854	32	2.422	2.410
12217	2020-4-5 8:00	US	AMRO	32105	273808	1166	7020	33	2.564	2.568
12218	2020-4-6 8:00	US	AMRO	33510	307318	1338	8358	34	2.720	2.716
12219	2020-4-7 8:00	US	AMRO	26493	333811	1201	9559	35	2.864	2.856
12220	2020-4-8 8:00	US	AMRO	29510	363321	1286	10845	36	2.985	3.025
12221	2020-4-9 8:00	US	AMRO	31709	395030	1895	12740	37	3.225	3.218
12222	2020-4-10 8:00	US	AMRO	30859	425889	1925	14665	38	3.443	3.422
12223	2020-4-11 8:00	US	AMRO	35386	461275	1931	16596	39	3.598	3.599
12224	2020-4-12 8:00	US	AMRO	31606	492881	1920	18516	40	3.757	3.751
12225	2020-4-13 8:00	US	AMRO	31633	524514	1928	20444	41	3.898	3.874
12226	2020-4-14 8:00	US	AMRO	29308	553822	1528	21972	42	3.967	3.975
12227	2020-4-15 8:00	US	AMRO	24446	578268	1504	23476	43	4.060	4.103
12228	2020-4-16 8:00	US	AMRO	25802	604070	2395	25871	44	4.283	4.267
12229	2020-4-17 8:00	US	AMRO	28711	632781	2350	28221	45	4.460	4.436
12230	2020-4-18 8:00	US	AMRO	32549	665330	2163	30384	46	4.567	4.563
12231	2020-4-19 8:00	US	AMRO	30023	695353	2043	32427	47	4.663	4.652
12232	2020-4-20 8:00	US	AMRO	28252	723605	1776	34203	48	4.727	4.722
12233	2020-4-21 8:00	US	AMRO	27668	751273	1681	35884	49	4.776	4.781
12234	2020-4-22 8:00	US	AMRO	25634	776907	1718	37602	50	4.840	4.873
12235	2020-4-23 8:00	US	AMRO	24019	800926	2471	40073	51	5.003	4.980
12236	2020-4-24 8:00	US	AMRO	29127	830053	2238	42311	52	5.097	5.073
12237	2020-4-25 8:00	US	AMRO	30719	860772	1742	44053	53	5.118	5.118
12238	2020-4-26 8:00	US	AMRO	38509	899281	2151	46204	54	5.138	5.135
12239	2020-4-27 8:00	US	AMRO	32417	931698	1776	47980	55	5.150	5.135
12240	2020-4-28 8:00	US	AMRO	29218	960916	1190	49170	56	5.117	5.134

Table S1 Daily confirmed cases and daily deaths of COVID-19 in 214 nations from WHO

ID	Date and time	Country or region	Continent	Confirmed patients	Cumulative Confirmed patients	Daily dead patients	Daily cumulative dead patients	Lag days	Daily CFR	3DMA CFR
12241	2020-4-29 8:00	US	AMRO	22541	983457	1322	50492	57	5.134	5.158
12242	2020-4-30 8:00	US	AMRO	20517	1003974	1936	52428	58	5.222	5.234
12243	2020-5-1 8:00	US	AMRO	31379	1035353	2909	55337	59	5.345	5.315
12244	2020-5-2 8:00	US	AMRO	31774	1067127	2069	57406	60	5.379	5.362
12245										
12246	2020-3-13 8:00	VI	AMRO	1	1	0	0			
12247	2020-3-14 8:00	VI	AMRO	0	1	0	0			
12248	2020-3-15 8:00	VI	AMRO	0	1	0	0			
12249	2020-3-16 8:00	VI	AMRO	0	1	0	0			
12250	2020-3-17 8:00	VI	AMRO	1	2	0	0			
12251	2020-3-18 8:00	VI	AMRO	1	3	0	0			
12252	2020-3-19 8:00	VI	AMRO	0	3	0	0			
12253	2020-3-20 8:00	VI	AMRO	0	3	0	0			
12254	2020-3-21 8:00	VI	AMRO	3	6	0	0			
12255	2020-3-22 8:00	VI	AMRO	0	6	0	0			
12256	2020-3-23 8:00	VI	AMRO	0	6	0	0			
12257	2020-3-24 8:00	VI	AMRO	11	17	0	0			
12258	2020-3-25 8:00	VI	AMRO	0	17	0	0			
12259	2020-3-26 8:00	VI	AMRO	0	17	0	0			
12260	2020-3-27 8:00	VI	AMRO	0	17	0	0			
12261	2020-3-28 8:00	VI	AMRO	0	17	0	0			
12262	2020-3-29 8:00	VI	AMRO	0	17	0	0			
12263	2020-3-30 8:00	VI	AMRO	5	22	0	0			
12264	2020-3-31 8:00	VI	AMRO	8	30	0	0			
12265	2020-4-1 8:00	VI	AMRO	0	30	0	0			
12266	2020-4-2 8:00	VI	AMRO	0	30	0	0			
12267	2020-4-3 8:00	VI	AMRO	3	33	0	0			
12268	2020-4-4 8:00	VI	AMRO	4	37	0	0			
12269	2020-4-5 8:00	VI	AMRO	3	40	0	0			
12270	2020-4-6 8:00	VI	AMRO	2	42	0	0			
12271	2020-4-7 8:00	VI	AMRO	1	43	1	1	0	2.326	2.326
12272	2020-4-8 8:00	VI	AMRO	0	43	0	1	1	2.326	2.291
12273	2020-4-9 8:00	VI	AMRO	2	45	0	1	2	2.222	2.241
12274	2020-4-10 8:00	VI	AMRO	1	46	0	1	3	2.174	2.119
12275	2020-4-11 8:00	VI	AMRO	5	51	0	1	4	1.961	2.032
12276	2020-4-12 8:00	VI	AMRO	0	51	0	1	5	1.961	1.961

Table S1 Daily confirmed cases and daily deaths of COVID-19 in 214 nations from WHO

ID	Date and time	Country or region	Continent	Confirmed patients	Cumulative Confirmed patients	Daily dead patients	Daily cumulative dead patients	Lag days	Daily CFR	3DMA CFR
12277	2020-4-13 8:00	VI	AMRO	0	51	0	1	6	1.961	1.961
12278	2020-4-14 8:00	VI	AMRO	0	51	0	1	7	1.961	1.961
12279	2020-4-15 8:00	VI	AMRO	0	51	0	1	8	1.961	1.961
12280	2020-4-16 8:00	VI	AMRO	0	51	0	1	9	1.961	1.961
12281	2020-4-17 8:00	VI	AMRO	0	51	0	1	10	1.961	2.614
12282	2020-4-18 8:00	VI	AMRO	0	51	1	2	11	3.922	3.848
12283	2020-4-19 8:00	VI	AMRO	2	53	1	3	12	5.660	5.081
12284	2020-4-20 8:00	VI	AMRO	0	53	0	3	13	5.660	5.660
12285	2020-4-21 8:00	VI	AMRO	0	53	0	3	14	5.660	5.625
12286	2020-4-22 8:00	VI	AMRO	1	54	0	3	15	5.556	5.590
12287	2020-4-23 8:00	VI	AMRO	0	54	0	3	16	5.556	5.556
12288	2020-4-24 8:00	VI	AMRO	0	54	0	3	17	5.556	5.556
12289	2020-4-25 8:00	VI	AMRO	0	54	0	3	18	5.556	5.522
12290	2020-4-26 8:00	VI	AMRO	1	55	0	3	19	5.455	5.488
12291	2020-4-27 8:00	VI	AMRO	0	55	0	3	20	5.455	5.896
12292	2020-4-28 8:00	VI	AMRO	4	59	1	4	21	6.780	6.338
12293	2020-4-29 8:00	VI	AMRO	0	59	0	4	22	6.780	6.670
12294	2020-4-30 8:00	VI	AMRO	3	62	0	4	23	6.452	6.431
12295	2020-5-1 8:00	VI	AMRO	4	66	0	4	24	6.061	6.191
12296	2020-5-2 8:00	VI	AMRO	0	66	0	4	25	6.061	6.061
12297										
12298	2020-3-15 8:00	UY	AMRO	4	4	0	0			
12299	2020-3-16 8:00	UY	AMRO	0	4	0	0			
12300	2020-3-17 8:00	UY	AMRO	2	6	0	0			
12301	2020-3-18 8:00	UY	AMRO	23	29	0	0			
12302	2020-3-19 8:00	UY	AMRO	50	79	0	0			
12303	2020-3-20 8:00	UY	AMRO	15	94	0	0			
12304	2020-3-21 8:00	UY	AMRO	0	94	0	0			
12305	2020-3-22 8:00	UY	AMRO	0	94	0	0			
12306	2020-3-23 8:00	UY	AMRO	41	135	0	0			
12307	2020-3-24 8:00	UY	AMRO	27	162	0	0			
12308	2020-3-25 8:00	UY	AMRO	0	162	0	0			
12309	2020-3-26 8:00	UY	AMRO	55	217	0	0			
12310	2020-3-27 8:00	UY	AMRO	0	217	0	0			
12311	2020-3-28 8:00	UY	AMRO	21	238	0	0			
12312	2020-3-29 8:00	UY	AMRO	36	274	0	0			

Table S1 Daily confirmed cases and daily deaths of COVID-19 in 214 nations from WHO

ID	Date and time	Country or region	Continent	Confirmed patients	Cumulative Confirmed patients	Daily dead patients	Daily cumulative dead patients	Lag days	Daily CFR	3DMA CFR
12313	2020-3-30 8:00	UY	AMRO	29	303	0	0			
12314	2020-3-31 8:00	UY	AMRO	6	309	0	0			
12315	2020-4-1 8:00	UY	AMRO	11	320	1	1	0	0.313	0.452
12316	2020-4-2 8:00	UY	AMRO	18	338	1	2	1	0.592	0.682
12317	2020-4-3 8:00	UY	AMRO	12	350	2	4	2	1.143	0.940
12318	2020-4-4 8:00	UY	AMRO	19	369	0	4	3	1.084	1.088
12319	2020-4-5 8:00	UY	AMRO	17	386	0	4	4	1.036	1.123
12320	2020-4-6 8:00	UY	AMRO	14	400	1	5	5	1.250	1.255
12321	2020-4-7 8:00	UY	AMRO	6	406	1	6	6	1.478	1.391
12322	2020-4-8 8:00	UY	AMRO	9	415	0	6	7	1.446	1.525
12323	2020-4-9 8:00	UY	AMRO	9	424	1	7	8	1.651	1.555
12324	2020-4-10 8:00	UY	AMRO	22	446	0	7	9	1.570	1.589
12325	2020-4-11 8:00	UY	AMRO	7	453	0	7	10	1.545	1.540
12326	2020-4-12 8:00	UY	AMRO	12	465	0	7	11	1.505	1.511
12327	2020-4-13 8:00	UY	AMRO	7	472	0	7	12	1.483	1.479
12328	2020-4-14 8:00	UY	AMRO	11	483	0	7	13	1.449	1.530
12329	2020-4-15 8:00	UY	AMRO	0	483	1	8	14	1.656	1.577
12330	2020-4-16 8:00	UY	AMRO	9	492	0	8	15	1.626	1.703
12331	2020-4-17 8:00	UY	AMRO	1	493	1	9	16	1.826	1.748
12332	2020-4-18 8:00	UY	AMRO	9	502	0	9	17	1.793	1.797
12333	2020-4-19 8:00	UY	AMRO	6	508	0	9	18	1.772	1.768
12334	2020-4-20 8:00	UY	AMRO	9	517	0	9	19	1.741	1.802
12335	2020-4-21 8:00	UY	AMRO	11	528	1	10	20	1.894	1.835
12336	2020-4-22 8:00	UY	AMRO	7	535	0	10	21	1.869	1.991
12337	2020-4-23 8:00	UY	AMRO	8	543	2	12	22	2.210	2.088
12338	2020-4-24 8:00	UY	AMRO	6	549	0	12	23	2.186	2.183
12339	2020-4-25 8:00	UY	AMRO	8	557	0	12	24	2.154	2.157
12340	2020-4-26 8:00	UY	AMRO	6	563	0	12	25	2.131	2.212
12341	2020-4-27 8:00	UY	AMRO	33	596	2	14	26	2.349	2.319
12342	2020-4-28 8:00	UY	AMRO	10	606	1	15	27	2.475	2.415
12343	2020-4-29 8:00	UY	AMRO	14	620	0	15	28	2.419	2.432
12344	2020-4-30 8:00	UY	AMRO	5	625	0	15	29	2.400	2.400
12345	2020-5-1 8:00	UY	AMRO	5	630	0	15	30	2.381	2.475
12346	2020-5-2 8:00	UY	AMRO	13	643	2	17	31	2.644	2.512
12347										
12348	2020-3-16 8:00	UZ	EURO	4	4	0	0			

Table S1 Daily confirmed cases and daily deaths of COVID-19 in 214 nations from WHO

ID	Date and time	Country or region	Continent	Confirmed patients	Cumulative Confirmed patients	Daily dead patients	Daily cumulative dead patients	Lag days	Daily CFR	3DMA CFR
12349	2020-3-17 8:00	UZ	EURO	12	16	0	0			
12350	2020-3-18 8:00	UZ	EURO	0	16	0	0			
12351	2020-3-19 8:00	UZ	EURO	5	21	0	0			
12352	2020-3-20 8:00	UZ	EURO	12	33	0	0			
12353	2020-3-21 8:00	UZ	EURO	0	33	0	0			
12354	2020-3-22 8:00	UZ	EURO	0	33	0	0			
12355	2020-3-23 8:00	UZ	EURO	13	46	0	0			
12356	2020-3-24 8:00	UZ	EURO	0	46	0	0			
12357	2020-3-25 8:00	UZ	EURO	4	50	0	0			
12358	2020-3-26 8:00	UZ	EURO	15	65	0	0			
12359	2020-3-27 8:00	UZ	EURO	18	83	0	0			
12360	2020-3-28 8:00	UZ	EURO	21	104	1	1	0	0.962	1.233
12361	2020-3-29 8:00	UZ	EURO	29	133	1	2	1	1.504	1.282
12362	2020-3-30 8:00	UZ	EURO	12	145	0	2	2	1.379	1.408
12363	2020-3-31 8:00	UZ	EURO	4	149	0	2	3	1.342	1.293
12364	2020-4-1 8:00	UZ	EURO	24	173	0	2	4	1.156	1.184
12365	2020-4-2 8:00	UZ	EURO	17	190	0	2	5	1.053	1.038
12366	2020-4-3 8:00	UZ	EURO	31	221	0	2	6	0.905	0.929
12367	2020-4-4 8:00	UZ	EURO	20	241	0	2	7	0.830	0.802
12368	2020-4-5 8:00	UZ	EURO	57	298	0	2	8	0.671	0.671
12369	2020-4-6 8:00	UZ	EURO	92	390	0	2	9	0.513	0.536
12370	2020-4-7 8:00	UZ	EURO	82	472	0	2	10	0.424	0.499
12371	2020-4-8 8:00	UZ	EURO	62	534	1	3	11	0.562	0.509
12372	2020-4-9 8:00	UZ	EURO	21	555	0	3	12	0.541	0.528
12373	2020-4-10 8:00	UZ	EURO	69	624	0	3	13	0.481	0.501
12374	2020-4-11 8:00	UZ	EURO	0	624	0	3	14	0.481	0.446
12375	2020-4-12 8:00	UZ	EURO	172	796	0	3	15	0.377	0.435
12376	2020-4-13 8:00	UZ	EURO	100	896	1	4	16	0.446	0.401
12377	2020-4-14 8:00	UZ	EURO	158	1054	0	4	17	0.380	0.385
12378	2020-4-15 8:00	UZ	EURO	160	1214	0	4	18	0.329	0.335
12379	2020-4-16 8:00	UZ	EURO	135	1349	0	4	19	0.297	0.305
12380	2020-4-17 8:00	UZ	EURO	31	1380	0	4	20	0.290	0.287
12381	2020-4-18 8:00	UZ	EURO	70	1450	0	4	21	0.276	0.300
12382	2020-4-19 8:00	UZ	EURO	45	1495	1	5	22	0.334	0.310
12383	2020-4-20 8:00	UZ	EURO	70	1565	0	5	23	0.319	0.319
12384	2020-4-21 8:00	UZ	EURO	92	1657	0	5	24	0.302	0.328

Table S1 Daily confirmed cases and daily deaths of COVID-19 in 214 nations from WHO

ID	Date and time	Country or region	Continent	Confirmed patients	Cumulative Confirmed patients	Daily dead patients	Daily cumulative dead patients	Lag days	Daily CFR	3DMA CFR
12385	2020-4-22 8:00	UZ	EURO	0	1657	1	6	25	0.362	0.357
12386	2020-4-23 8:00	UZ	EURO	59	1716	1	7	26	0.408	0.388
12387	2020-4-24 8:00	UZ	EURO	62	1778	0	7	27	0.394	0.412
12388	2020-4-25 8:00	UZ	EURO	58	1836	1	8	28	0.436	0.419
12389	2020-4-26 8:00	UZ	EURO	29	1865	0	8	29	0.429	0.430
12390	2020-4-27 8:00	UZ	EURO	22	1887	0	8	30	0.424	0.423
12391	2020-4-28 8:00	UZ	EURO	37	1924	0	8	31	0.416	0.416
12392	2020-4-29 8:00	UZ	EURO	31	1955	0	8	32	0.409	0.423
12393	2020-4-30 8:00	UZ	EURO	76	2031	1	9	33	0.443	0.431
12394	2020-5-1 8:00	UZ	EURO	15	2046	0	9	34	0.440	0.438
12395	2020-5-2 8:00	UZ	EURO	48	2094	0	9	35	0.430	0.435
12396										
12397	2020-3-14 8:00	VE	AMRO	2	2	0	0			
12398	2020-3-15 8:00	VE	AMRO	15	17	0	0			
12399	2020-3-16 8:00	VE	AMRO	0	17	0	0			
12400	2020-3-17 8:00	VE	AMRO	16	33	0	0			
12401	2020-3-18 8:00	VE	AMRO	3	36	0	0			
12402	2020-3-19 8:00	VE	AMRO	0	36	0	0			
12403	2020-3-20 8:00	VE	AMRO	0	36	0	0			
12404	2020-3-21 8:00	VE	AMRO	0	36	0	0			
12405	2020-3-22 8:00	VE	AMRO	0	36	0	0			
12406	2020-3-23 8:00	VE	AMRO	34	70	0	0			
12407	2020-3-24 8:00	VE	AMRO	7	77	0	0			
12408	2020-3-25 8:00	VE	AMRO	0	77	0	0			
12409	2020-3-26 8:00	VE	AMRO	14	91	0	0			
12410	2020-3-27 8:00	VE	AMRO	0	91	0	0			
12411	2020-3-28 8:00	VE	AMRO	15	106	0	0			
12412	2020-3-29 8:00	VE	AMRO	7	113	2	2	0	1.770	1.770
12413	2020-3-30 8:00	VE	AMRO	0	113	0	2	1	1.770	1.955
12414	2020-3-31 8:00	VE	AMRO	16	129	1	3	2	2.326	2.140
12415	2020-4-1 8:00	VE	AMRO	0	129	0	3	3	2.326	2.250
12416	2020-4-2 8:00	VE	AMRO	14	143	0	3	4	2.098	2.169
12417	2020-4-3 8:00	VE	AMRO	1	144	0	3	5	2.083	2.088
12418	2020-4-4 8:00	VE	AMRO	0	144	0	3	6	2.083	2.083
12419	2020-4-5 8:00	VE	AMRO	0	144	0	3	7	2.083	2.083
12420	2020-4-6 8:00	VE	AMRO	0	144	0	3	8	2.083	2.018

Table S1 Daily confirmed cases and daily deaths of COVID-19 in 214 nations from WHO

ID	Date and time	Country or region	Continent	Confirmed patients	Cumulative Confirmed patients	Daily dead patients	Daily cumulative dead patients	Lag days	Daily CFR	3DMA CFR
12421	2020-4-7 8:00	VE	AMRO	15	159	0	3	9	1.887	2.738
12422	2020-4-8 8:00	VE	AMRO	6	165	4	7	10	4.242	3.449
12423	2020-4-9 8:00	VE	AMRO	1	166	0	7	11	4.217	4.225
12424	2020-4-10 8:00	VE	AMRO	0	166	0	7	12	4.217	4.566
12425	2020-4-11 8:00	VE	AMRO	5	171	2	9	13	5.263	4.914
12426	2020-4-12 8:00	VE	AMRO	0	171	0	9	14	5.263	5.263
12427	2020-4-13 8:00	VE	AMRO	0	171	0	9	15	5.263	5.166
12428	2020-4-14 8:00	VE	AMRO	10	181	0	9	16	4.972	5.069
12429	2020-4-15 8:00	VE	AMRO	0	181	0	9	17	4.972	4.869
12430	2020-4-16 8:00	VE	AMRO	12	193	0	9	18	4.663	4.735
12431	2020-4-17 8:00	VE	AMRO	4	197	0	9	19	4.569	4.548
12432	2020-4-18 8:00	VE	AMRO	7	204	0	9	20	4.412	4.464
12433	2020-4-19 8:00	VE	AMRO	0	204	0	9	21	4.412	4.257
12434	2020-4-20 8:00	VE	AMRO	24	228	0	9	22	3.947	3.958
12435	2020-4-21 8:00	VE	AMRO	28	256	0	9	23	3.516	3.657
12436	2020-4-22 8:00	VE	AMRO	29	285	1	10	24	3.509	3.499
12437	2020-4-23 8:00	VE	AMRO	3	288	0	10	25	3.472	3.446
12438	2020-4-24 8:00	VE	AMRO	10	298	0	10	26	3.356	3.324
12439	2020-4-25 8:00	VE	AMRO	20	318	0	10	27	3.145	3.215
12440	2020-4-26 8:00	VE	AMRO	0	318	0	10	28	3.145	3.128
12441	2020-4-27 8:00	VE	AMRO	5	323	0	10	29	3.096	3.106
12442	2020-4-28 8:00	VE	AMRO	2	325	0	10	30	3.077	3.071
12443	2020-4-29 8:00	VE	AMRO	4	329	0	10	31	3.040	3.052
12444	2020-4-30 8:00	VE	AMRO	0	329	0	10	32	3.040	3.033
12445	2020-5-1 8:00	VE	AMRO	2	331	0	10	33	3.021	3.021
12446	2020-5-2 8:00	VE	AMRO	2	333	0	10	34	3.003	3.012
12447										
12448	2020-1-24 8:00	VN	WPRO	2	2	0	0			
12449	2020-1-25 8:00	VN	WPRO	0	2	0	0			
12450	2020-1-26 8:00	VN	WPRO	0	2	0	0			
12451	2020-1-27 8:00	VN	WPRO	0	2	0	0			
12452	2020-1-28 8:00	VN	WPRO	0	2	0	0			
12453	2020-1-29 8:00	VN	WPRO	0	2	0	0			
12454	2020-1-30 8:00	VN	WPRO	3	5	0	0			
12455	2020-1-31 8:00	VN	WPRO	0	5	0	0			
12456	2020-2-1 8:00	VN	WPRO	1	6	0	0			

Table S1 Daily confirmed cases and daily deaths of COVID-19 in 214 nations from WHO

ID	Date and time	Country or region	Continent	Confirmed patients	Cumulative Confirmed patients	Daily dead patients	Daily cumulative dead patients	Lag days	Daily CFR	3DMA CFR
12457	2020-2-2 8:00	VN	WPRO	1	7	0	0			
12458	2020-2-3 8:00	VN	WPRO	1	8	0	0			
12459	2020-2-4 8:00	VN	WPRO	2	10	0	0			
12460	2020-2-5 8:00	VN	WPRO	0	10	0	0			
12461	2020-2-6 8:00	VN	WPRO	2	12	0	0			
12462	2020-2-7 8:00	VN	WPRO	0	12	0	0			
12463	2020-2-8 8:00	VN	WPRO	1	13	0	0			
12464	2020-2-9 8:00	VN	WPRO	1	14	0	0			
12465	2020-2-10 8:00	VN	WPRO	0	14	0	0			
12466	2020-2-11 8:00	VN	WPRO	1	15	0	0			
12467	2020-2-12 8:00	VN	WPRO	0	15	0	0			
12468	2020-2-13 8:00	VN	WPRO	1	16	0	0			
12469	2020-2-14 8:00	VN	WPRO	0	16	0	0			
12470	2020-2-15 8:00	VN	WPRO	0	16	0	0			
12471	2020-2-16 8:00	VN	WPRO	0	16	0	0			
12472	2020-2-17 8:00	VN	WPRO	0	16	0	0			
12473	2020-2-18 8:00	VN	WPRO	0	16	0	0			
12474	2020-2-19 8:00	VN	WPRO	0	16	0	0			
12475	2020-2-20 8:00	VN	WPRO	0	16	0	0			
12476	2020-2-21 8:00	VN	WPRO	0	16	0	0			
12477	2020-2-22 8:00	VN	WPRO	0	16	0	0			
12478	2020-2-23 8:00	VN	WPRO	0	16	0	0			
12479	2020-2-24 8:00	VN	WPRO	0	16	0	0			
12480	2020-2-25 8:00	VN	WPRO	0	16	0	0			
12481	2020-2-26 8:00	VN	WPRO	0	16	0	0			
12482	2020-2-27 8:00	VN	WPRO	0	16	0	0			
12483	2020-2-28 8:00	VN	WPRO	0	16	0	0			
12484	2020-2-29 8:00	VN	WPRO	0	16	0	0			
12485	2020-3-1 8:00	VN	WPRO	0	16	0	0			
12486	2020-3-2 8:00	VN	WPRO	0	16	0	0			
12487	2020-3-3 8:00	VN	WPRO	0	16	0	0			
12488	2020-3-4 8:00	VN	WPRO	0	16	0	0			
12489	2020-3-5 8:00	VN	WPRO	0	16	0	0			
12490	2020-3-6 8:00	VN	WPRO	1	17	0	0			
12491	2020-3-7 8:00	VN	WPRO	3	20	0	0			
12492	2020-3-8 8:00	VN	WPRO	10	30	0	0			

Table S1 Daily confirmed cases and daily deaths of COVID-19 in 214 nations from WHO

ID	Date and time	Country or region	Continent	Confirmed patients	Cumulative Confirmed patients	Daily dead patients	Daily cumulative dead patients	Lag days	Daily CFR	3DMA CFR
12493	2020-3-9 8:00	VN	WPRO	1	31	0	0			
12494	2020-3-10 8:00	VN	WPRO	0	31	0	0			
12495	2020-3-11 8:00	VN	WPRO	4	35	0	0			
12496	2020-3-12 8:00	VN	WPRO	4	39	0	0			
12497	2020-3-13 8:00	VN	WPRO	0	39	0	0			
12498	2020-3-14 8:00	VN	WPRO	9	48	0	0			
12499	2020-3-15 8:00	VN	WPRO	5	53	0	0			
12500	2020-3-16 8:00	VN	WPRO	4	57	0	0			
12501	2020-3-17 8:00	VN	WPRO	4	61	0	0			
12502	2020-3-18 8:00	VN	WPRO	5	66	0	0			
12503	2020-3-19 8:00	VN	WPRO	19	85	0	0			
12504	2020-3-20 8:00	VN	WPRO	6	91	0	0			
12505	2020-3-21 8:00	VN	WPRO	3	94	0	0			
12506	2020-3-22 8:00	VN	WPRO	0	94	0	0			
12507	2020-3-23 8:00	VN	WPRO	24	118	0	0			
12508	2020-3-24 8:00	VN	WPRO	5	123	0	0			
12509	2020-3-25 8:00	VN	WPRO	11	134	0	0			
12510	2020-3-26 8:00	VN	WPRO	14	148	0	0			
12511	2020-3-27 8:00	VN	WPRO	5	153	0	0			
12512	2020-3-28 8:00	VN	WPRO	16	169	0	0			
12513	2020-3-29 8:00	VN	WPRO	10	179	0	0			
12514	2020-3-30 8:00	VN	WPRO	15	194	0	0			
12515	2020-3-31 8:00	VN	WPRO	9	203	0	0			
12516	2020-4-1 8:00	VN	WPRO	9	212	0	0			
12517	2020-4-2 8:00	VN	WPRO	10	222	0	0			
12518	2020-4-3 8:00	VN	WPRO	11	233	0	0			
12519	2020-4-4 8:00	VN	WPRO	6	239	0	0			
12520	2020-4-5 8:00	VN	WPRO	1	240	0	0			
12521	2020-4-6 8:00	VN	WPRO	1	241	0	0			
12522	2020-4-7 8:00	VN	WPRO	4	245	0	0			
12523	2020-4-8 8:00	VN	WPRO	6	251	0	0			
12524	2020-4-9 8:00	VN	WPRO	0	251	0	0			
12525	2020-4-10 8:00	VN	WPRO	4	255	0	0			
12526	2020-4-11 8:00	VN	WPRO	2	257	0	0			
12527	2020-4-12 8:00	VN	WPRO	1	258	0	0			
12528	2020-4-13 8:00	VN	WPRO	4	262	0	0			

Table S1 Daily confirmed cases and daily deaths of COVID-19 in 214 nations from WHO

ID	Date and time	Country or region	Continent	Confirmed patients	Cumulative Confirmed patients	Daily dead patients	Daily cumulative dead patients	Lag days	Daily CFR	3DMA CFR
12529	2020-4-14 8:00	VN	WPRO	3	265	0	0			
12530	2020-4-15 8:00	VN	WPRO	2	267	0	0			
12531	2020-4-16 8:00	VN	WPRO	0	267	0	0			
12532	2020-4-17 8:00	VN	WPRO	1	268	0	0			
12533	2020-4-18 8:00	VN	WPRO	0	268	0	0			
12534	2020-4-19 8:00	VN	WPRO	0	268	0	0			
12535	2020-4-20 8:00	VN	WPRO	0	268	0	0			
12536	2020-4-21 8:00	VN	WPRO	0	268	0	0			
12537	2020-4-22 8:00	VN	WPRO	0	268	0	0			
12538	2020-4-23 8:00	VN	WPRO	0	268	0	0			
12539	2020-4-24 8:00	VN	WPRO	0	268	0	0			
12540	2020-4-25 8:00	VN	WPRO	2	270	0	0			
12541	2020-4-26 8:00	VN	WPRO	0	270	0	0			
12542	2020-4-27 8:00	VN	WPRO	0	270	0	0			
12543	2020-4-28 8:00	VN	WPRO	0	270	0	0			
12544	2020-4-29 8:00	VN	WPRO	0	270	0	0			
12545	2020-4-30 8:00	VN	WPRO	0	270	0	0			
12546	2020-5-1 8:00	VN	WPRO	0	270	0	0			
12547	2020-5-2 8:00	VN	WPRO	0	270	0	0			
12548										
12549	2020-4-10 8:00	YE	EMRO	1	1	0	0			
12550	2020-4-11 8:00	YE	EMRO	0	1	0	0			
12551	2020-4-12 8:00	YE	EMRO	0	1	0	0			
12552	2020-4-13 8:00	YE	EMRO	0	1	0	0			
12553	2020-4-14 8:00	YE	EMRO	0	1	0	0			
12554	2020-4-15 8:00	YE	EMRO	0	1	0	0			
12555	2020-4-16 8:00	YE	EMRO	0	1	0	0			
12556	2020-4-17 8:00	YE	EMRO	0	1	0	0			
12557	2020-4-18 8:00	YE	EMRO	0	1	0	0			
12558	2020-4-19 8:00	YE	EMRO	0	1	0	0			
12559	2020-4-20 8:00	YE	EMRO	0	1	0	0			
12560	2020-4-21 8:00	YE	EMRO	0	1	0	0			
12561	2020-4-22 8:00	YE	EMRO	0	1	0	0			
12562	2020-4-23 8:00	YE	EMRO	0	1	0	0			
12563	2020-4-24 8:00	YE	EMRO	0	1	0	0			
12564	2020-4-25 8:00	YE	EMRO	0	1	0	0			

Table S1 Daily confirmed cases and daily deaths of COVID-19 in 214 nations from WHO

ID	Date and time	Country or region	Continent	Confirmed patients	Cumulative Confirmed patients	Daily dead patients	Daily cumulative dead patients	Lag days	Daily CFR	3DMA CFR
12565	2020-4-26 8:00	YE	EMRO	0	1	0	0			
12566	2020-4-27 8:00	YE	EMRO	0	1	0	0			
12567	2020-4-28 8:00	YE	EMRO	0	1	0	0			
12568	2020-4-29 8:00	YE	EMRO	5	6	0	0			
12569	2020-4-30 8:00	YE	EMRO	0	6	0	0			
12570	2020-5-1 8:00	YE	EMRO	0	6	2	2	0	33.333	30.952
12571	2020-5-2 8:00	YE	EMRO	1	7	0	2	1	28.571	30.952
12572										
12573	2020-3-18 8:00	ZM	AFRO	2	2	0	0			
12574	2020-3-19 8:00	ZM	AFRO	0	2	0	0			
12575	2020-3-20 8:00	ZM	AFRO	0	2	0	0			
12576	2020-3-21 8:00	ZM	AFRO	0	2	0	0			
12577	2020-3-22 8:00	ZM	AFRO	0	2	0	0			
12578	2020-3-23 8:00	ZM	AFRO	1	3	0	0			
12579	2020-3-24 8:00	ZM	AFRO	0	3	0	0			
12580	2020-3-25 8:00	ZM	AFRO	0	3	0	0			
12581	2020-3-26 8:00	ZM	AFRO	0	3	0	0			
12582	2020-3-27 8:00	ZM	AFRO	10	13	0	0			
12583	2020-3-28 8:00	ZM	AFRO	0	13	0	0			
12584	2020-3-29 8:00	ZM	AFRO	3	16	0	0			
12585	2020-3-30 8:00	ZM	AFRO	19	35	0	0			
12586	2020-3-31 8:00	ZM	AFRO	0	35	0	0			
12587	2020-4-1 8:00	ZM	AFRO	0	35	0	0			
12588	2020-4-2 8:00	ZM	AFRO	1	36	0	0			
12589	2020-4-3 8:00	ZM	AFRO	3	39	1	1	0	2.564	2.564
12590	2020-4-4 8:00	ZM	AFRO	0	39	0	1	1	2.564	2.564
12591	2020-4-5 8:00	ZM	AFRO	0	39	0	1	2	2.564	2.564
12592	2020-4-6 8:00	ZM	AFRO	0	39	0	1	3	2.564	2.564
12593	2020-4-7 8:00	ZM	AFRO	0	39	0	1	4	2.564	2.564
12594	2020-4-8 8:00	ZM	AFRO	0	39	0	1	5	2.564	2.564
12595	2020-4-9 8:00	ZM	AFRO	0	39	0	1	6	2.564	2.564
12596	2020-4-10 8:00	ZM	AFRO	0	39	0	1	7	2.564	3.376
12597	2020-4-11 8:00	ZM	AFRO	1	40	1	2	8	5.000	4.188
12598	2020-4-12 8:00	ZM	AFRO	0	40	0	2	9	5.000	4.884
12599	2020-4-13 8:00	ZM	AFRO	3	43	0	2	10	4.651	4.699
12600	2020-4-14 8:00	ZM	AFRO	2	45	0	2	11	4.444	4.513

Table S1 Daily confirmed cases and daily deaths of COVID-19 in 214 nations from WHO

ID	Date and time	Country or region	Continent	Confirmed patients	Cumulative Confirmed patients	Daily dead patients	Daily cumulative dead patients	Lag days	Daily CFR	3DMA CFR
12601	2020-4-15 8:00	ZM	AFRO	0	45	0	2	12	4.444	4.352
12602	2020-4-16 8:00	ZM	AFRO	3	48	0	2	13	4.167	4.259
12603	2020-4-17 8:00	ZM	AFRO	0	48	0	2	14	4.167	4.060
12604	2020-4-18 8:00	ZM	AFRO	4	52	0	2	15	3.846	3.841
12605	2020-4-19 8:00	ZM	AFRO	5	57	0	2	16	3.509	4.091
12606	2020-4-20 8:00	ZM	AFRO	4	61	1	3	17	4.918	4.347
12607	2020-4-21 8:00	ZM	AFRO	4	65	0	3	18	4.615	4.606
12608	2020-4-22 8:00	ZM	AFRO	5	70	0	3	19	4.286	4.318
12609	2020-4-23 8:00	ZM	AFRO	4	74	0	3	20	4.054	4.096
12610	2020-4-24 8:00	ZM	AFRO	2	76	0	3	21	3.947	3.858
12611	2020-4-25 8:00	ZM	AFRO	8	84	0	3	22	3.571	3.697
12612	2020-4-26 8:00	ZM	AFRO	0	84	0	3	23	3.571	3.517
12613	2020-4-27 8:00	ZM	AFRO	4	88	0	3	24	3.409	3.450
12614	2020-4-28 8:00	ZM	AFRO	1	89	0	3	25	3.371	3.313
12615	2020-4-29 8:00	ZM	AFRO	6	95	0	3	26	3.158	3.207
12616	2020-4-30 8:00	ZM	AFRO	2	97	0	3	27	3.093	3.027
12617	2020-5-1 8:00	ZM	AFRO	9	106	0	3	28	2.830	2.892
12618	2020-5-2 8:00	ZM	AFRO	3	109	0	3	29	2.752	2.791
12619										
12620	2020-3-20 8:00	ZW	AFRO	1	1	0	0			
12621	2020-3-21 8:00	ZW	AFRO	1	2	0	0			
12622	2020-3-22 8:00	ZW	AFRO	0	2	0	0			
12623	2020-3-23 8:00	ZW	AFRO	0	2	0	0			
12624	2020-3-24 8:00	ZW	AFRO	0	2	1	1	0	50.000	50.000
12625	2020-3-25 8:00	ZW	AFRO	0	2	0	1	1	50.000	50.000
12626	2020-3-26 8:00	ZW	AFRO	0	2	0	1	2	50.000	40.000
12627	2020-3-27 8:00	ZW	AFRO	3	5	0	1	3	20.000	30.000
12628	2020-3-28 8:00	ZW	AFRO	0	5	0	1	4	20.000	20.000
12629	2020-3-29 8:00	ZW	AFRO	0	5	0	1	5	20.000	20.000
12630	2020-3-30 8:00	ZW	AFRO	0	5	0	1	6	20.000	17.500
12631	2020-3-31 8:00	ZW	AFRO	3	8	0	1	7	12.500	15.000
12632	2020-4-1 8:00	ZW	AFRO	0	8	0	1	8	12.500	12.500
12633	2020-4-2 8:00	ZW	AFRO	0	8	0	1	9	12.500	12.037
12634	2020-4-3 8:00	ZW	AFRO	1	9	0	1	10	11.111	11.574
12635	2020-4-4 8:00	ZW	AFRO	0	9	0	1	11	11.111	11.111
12636	2020-4-5 8:00	ZW	AFRO	0	9	0	1	12	11.111	11.111

Table S1 Daily confirmed cases and daily deaths of COVID-19 in 214 nations from WHO

ID	Date and time	Country or region	Continent	Confirmed patients	Cumulative Confirmed patients	Daily dead patients	Daily cumulative dead patients	Lag days	Daily CFR	3DMA CFR
12637	2020-4-6 8:00	ZW	AFRO	0	9	0	1	13	11.111	11.111
12638	2020-4-7 8:00	ZW	AFRO	0	9	0	1	14	11.111	13.468
12639	2020-4-8 8:00	ZW	AFRO	2	11	1	2	15	18.182	18.855
12640	2020-4-9 8:00	ZW	AFRO	0	11	1	3	16	27.273	24.242
12641	2020-4-10 8:00	ZW	AFRO	0	11	0	3	17	27.273	27.273
12642	2020-4-11 8:00	ZW	AFRO	0	11	0	3	18	27.273	25.874
12643	2020-4-12 8:00	ZW	AFRO	2	13	0	3	19	23.077	23.926
12644	2020-4-13 8:00	ZW	AFRO	1	14	0	3	20	21.429	20.718
12645	2020-4-14 8:00	ZW	AFRO	3	17	0	3	21	17.647	18.581
12646	2020-4-15 8:00	ZW	AFRO	1	18	0	3	22	16.667	15.786
12647	2020-4-16 8:00	ZW	AFRO	5	23	0	3	23	13.043	14.251
12648	2020-4-17 8:00	ZW	AFRO	0	23	0	3	24	13.043	12.862
12649	2020-4-18 8:00	ZW	AFRO	1	24	0	3	25	12.500	12.514
12650	2020-4-19 8:00	ZW	AFRO	1	25	0	3	26	12.000	12.167
12651	2020-4-20 8:00	ZW	AFRO	0	25	0	3	27	12.000	12.000
12652	2020-4-21 8:00	ZW	AFRO	0	25	0	3	28	12.000	11.571
12653	2020-4-22 8:00	ZW	AFRO	3	28	0	3	29	10.714	12.333
12654	2020-4-23 8:00	ZW	AFRO	0	28	1	4	30	14.286	13.095
12655	2020-4-24 8:00	ZW	AFRO	0	28	0	4	31	14.286	14.122
12656	2020-4-25 8:00	ZW	AFRO	1	29	0	4	32	13.793	13.661
12657	2020-4-26 8:00	ZW	AFRO	2	31	0	4	33	12.903	13.200
12658	2020-4-27 8:00	ZW	AFRO	0	31	0	4	34	12.903	12.903
12659	2020-4-28 8:00	ZW	AFRO	0	31	0	4	35	12.903	12.769
12660	2020-4-29 8:00	ZW	AFRO	1	32	0	4	36	12.500	12.634
12661	2020-4-30 8:00	ZW	AFRO	0	32	0	4	37	12.500	12.500
12662	2020-5-1 8:00	ZW	AFRO	0	32	0	4	38	12.500	12.255
12663	2020-5-2 8:00	ZW	AFRO	2	34	0	4	39	11.765	12.132
